# Supplementary material for: Consequences of adaptation of TAL effectors on host susceptibility to Xanthomonas
Source: PLoS Genet. 2021 Jan 19;17(1):e1009310. doi: 10.1371/journal.pgen.1009310 (PMC7845958; doi:10.1371/journal.pgen.1009310)
Supplement: S1 Fig — The RVD variants of LOB1 targeting TALEs of Xcc and Xca (Table 1) were analyzed using QueTAL (http://bioinfo-web.mpl.ird.fr/cgi-bin2/quetal/quetal.cgi). A. Phylogenetic relationship between LOB1 targeting TALEs was analyzed using DisTAL v1.1. B. Functional relationship between LOB1 targeting TALEs was analyzed using FuncTAL v1.1. (PDF) [file pgen.1009310.s001.pdf]

A

DisTAL analysis

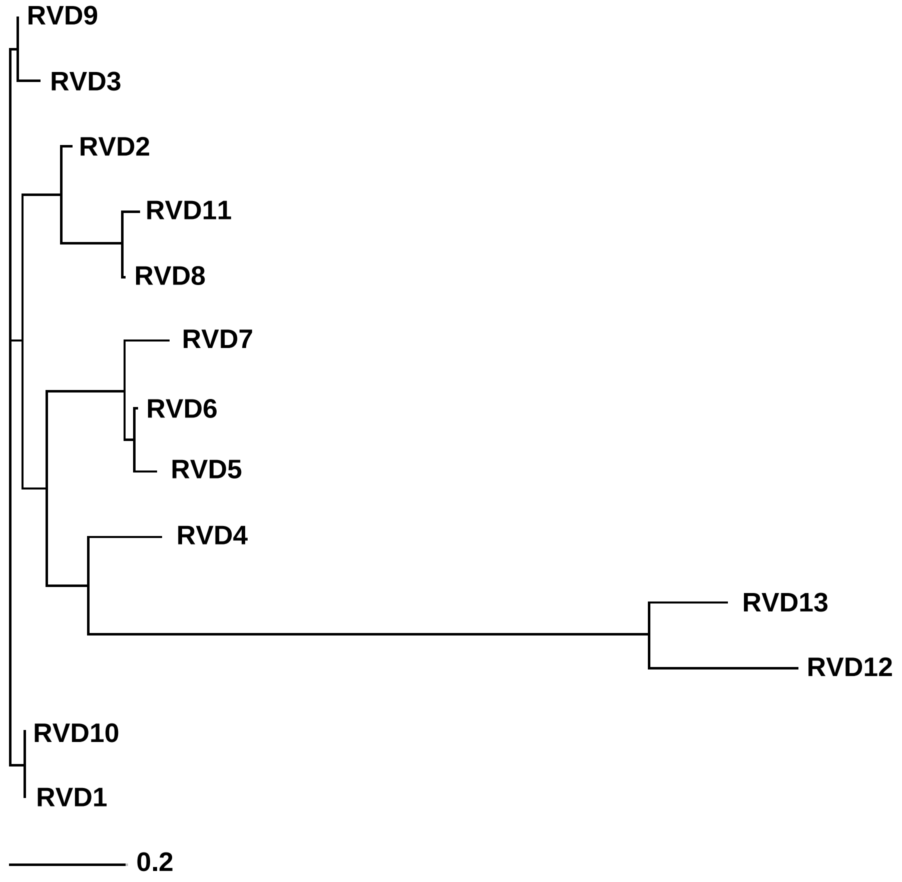

B

FuncTAL analysis

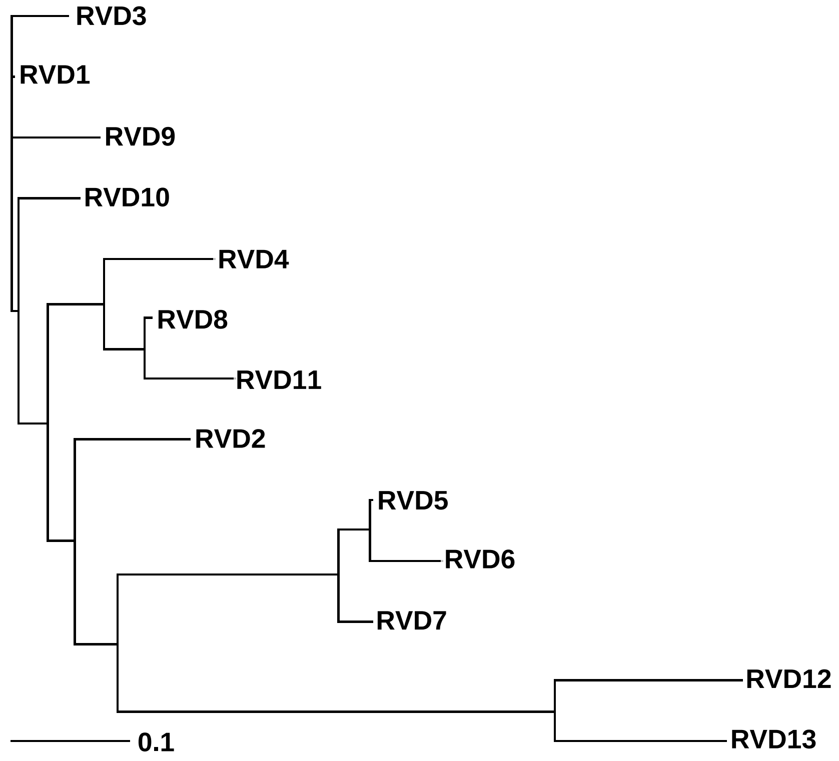

S1 Fig. Phylogenetic and functional relationships between *LOB1* targeting TALEs. The RVD variants of *LOB1* targeting TALEs of *Xcc* and *Xca* (Table 1) were analyzed using QueTAL (http://bioinfo-web.mpl.ird.fr/cgi-bin2/quetal/quetal.cgi). A. Phylogenetic relationship between *LOB1* targeting TALEs was analyzed using DisTAL v1.1. B. Functional relationship between *LOB1* targeting TALEs was analyzed using FuncTAL v1.1.
