# Supplementary material for: Consequences of adaptation of TAL effectors on host susceptibility to Xanthomonas
Source: PLoS Genet. 2021 Jan 19;17(1):e1009310. doi: 10.1371/journal.pgen.1009310 (PMC7845958; doi:10.1371/journal.pgen.1009310)
Supplement: S2 Fig — Sweet orange leaves were syringe-infiltrated with suspensions (1 × 10^8^ CFU/mL) of Xcc pthA4:Tn5 or Xcc pthA4:Tn5 transformed with the dTALEs depicted in Fig 2A. A. Inoculated leaves were photographed at 7 days post inoculation. B. The expression of CsLOB1 was quantified at 96 h post inoculation. The GAPDH gene was used as an endogenous control. Values are means ± SE of three biological replicates. Asterisks indicate a significant difference (Student’s t-test, P-value < 0.05) compared to Xcc pthA4:Tn5. The experiments were repeated three times with similar results. (PDF) [file pgen.1009310.s002.pdf]

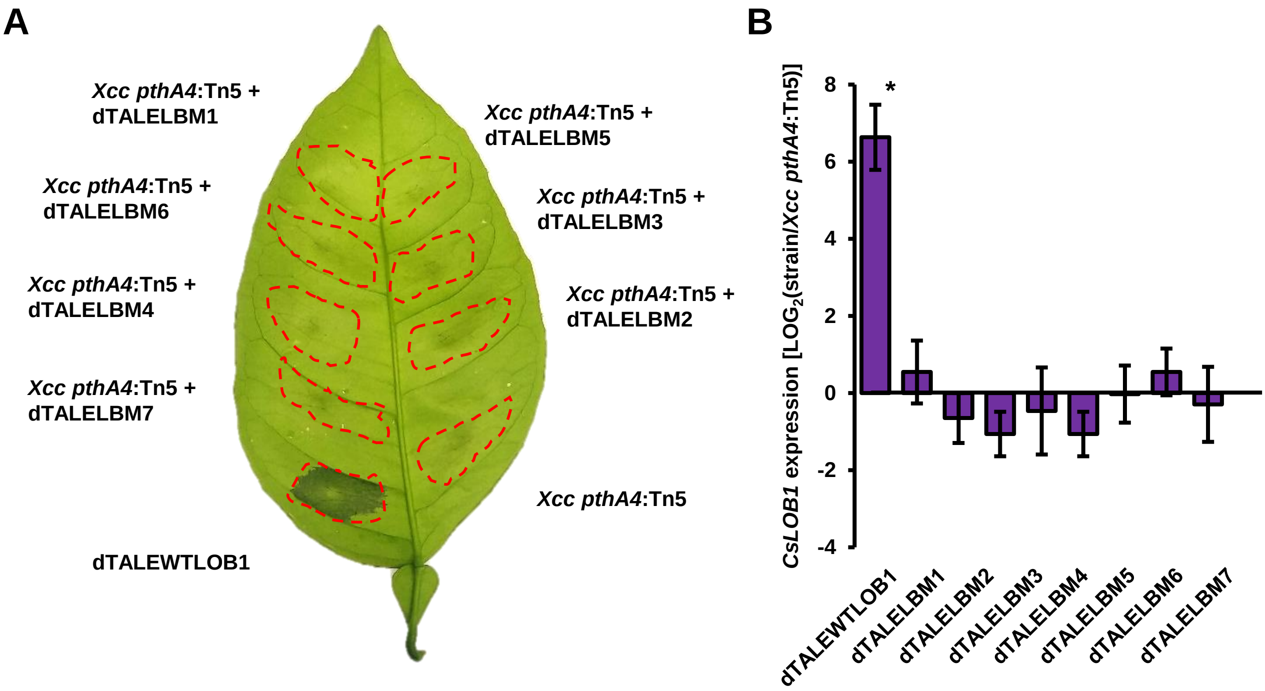

S2 Fig. Contribution of dTALEs to development of canker symptoms and expression of *CsLOB1*. Sweet orange leaves were syringe-infiltrated with suspensions (1 × 10^8 CFU/mL) of *Xcc pthA4*:Tn5 or *Xcc pthA4*:Tn5 transformed with the dTALEs depicted in Figure 2A. A. Inoculated leaves were photographed at 7 days post inoculation. B. The expression of *CsLOB1* was quantified at 96 h post inoculation. The *GAPDH* gene was used as an endogenous control. Values are means ± SE of three biological replicates. Asterisks indicate a significant difference (Student's *t*-test, *P*-value < 0.05) compared to *Xcc pthA4*:Tn5. The experiments were repeated three times with similar results.
