# Supplementary material for: Consequences of adaptation of TAL effectors on host susceptibility to Xanthomonas
Source: PLoS Genet. 2021 Jan 19;17(1):e1009310. doi: 10.1371/journal.pgen.1009310 (PMC7845958; doi:10.1371/journal.pgen.1009310)
Supplement: S4 Fig — Total protein was extracted from overnight cultures of Xcc pthA4:Tn5 [No vector control (NVC)], Xcc pthA4:Tn5 carrying pBBR1MCS-5 [Empty vector (EV)] and Xcc pthA4:Tn5 transformed with the parental and adapted dTALEs. Samples were separated by SDS-PAGE and immunoblotted with the anti-HA antibody (upper panel) or stained with coomassie blue (lower panel). (PDF) [file pgen.1009310.s004.pdf]

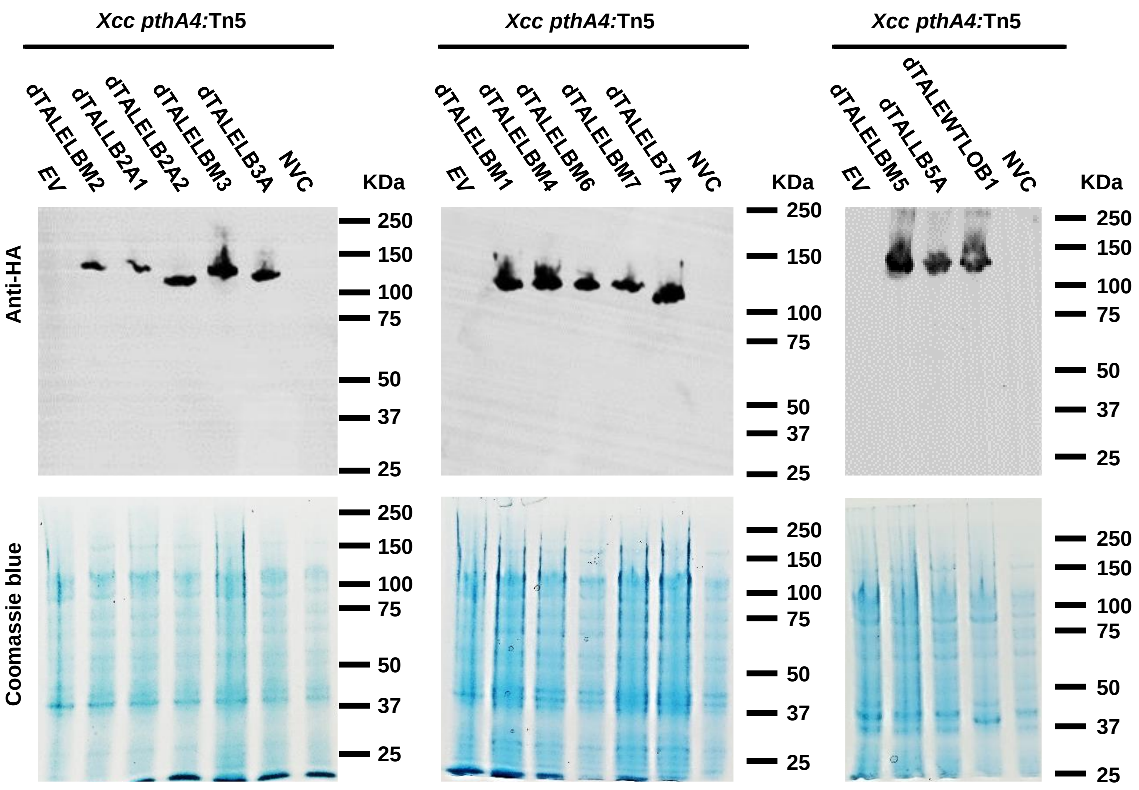

S4 Fig. Protein expression of dTALEs. Total protein was extracted from overnight cultures of *Xcc pthA4*:Tn5 [No vector control (NVC)], *Xcc pthA4*:Tn5 carrying pBBR1MCS-5 [Empty vector (EV)] and *Xcc pthA4*:Tn5 transformed with the parental and adapted dTALEs. Samples were separated by SDS-PAGE and immunoblotted with the anti-HA antibody (upper panel) or stained with coomassie blue (lower panel).
