# Supplementary material for: Consequences of adaptation of TAL effectors on host susceptibility to Xanthomonas
Source: PLoS Genet. 2021 Jan 19;17(1):e1009310. doi: 10.1371/journal.pgen.1009310 (PMC7845958; doi:10.1371/journal.pgen.1009310)
Supplement: S1 Table — (DOCX) [file pgen.1009310.s005.docx]

**S1 Table. Bacterial strains and plasmids used in this study**

| Strain or plasmid | Relevant characteristic | Reference |
| --- | --- | --- |
| **Strain** | | |
| ***Escherichia coli*** | | |
| HST08 | F–, endA1, supE44, thi-1, recA1, relA1, gyrA96, phoA, Φ80d lacZΔ M15, Δ (lacZYA - argF) U169, Δ (mrr - hsdRMS - mcrBC), ΔmcrA, λ– | TaKaRa Bio Inc. Kusatsu, Japan |
| ***Xanthomonas citri* subsp *citri*** | | |
| 306 | Wild-type | [1] |
| *Xcc* *pthA4*:Tn5 | Tn5 insertion mutant in XACb0065 (clone 320E11) in the background of *Xcc* 306, Tn5 was inserted in forward orientation in position 770 bp within the ORF of *pthA4*, Kn^R*^ | [2] |
| ***Agrobacterium tumefaciens*** | | |
| GV2260 | C58 background, carries Ti plasmid pGV2260 (pTiB6S3DT-DNA), Rif^R^, Amp^R^ | [3] |
| **Plasmid** | | |
| pBBR1MCS-5 | Broad host expression vector. Gn^R^ | [4] |
| pBBRNPth | pBBR1MCS-5 derivative for expression of -24 to +444 N-terminal coding fragment of PthA4 (XACb0065) and a HA tag. Used as backbone for construction of dTALEs. Gn^R^ | [5] |
| pTAL2 | Intermediate destination vector for assembled dTALEs. Ap^R^ | [6] |
| dTALEWTLOB1 | pBBR1MCS-5 expressing designer TALE (RVD repeat array: NI NG NI NI NI HD HD NG HD NG NG NG NG NN HD HD NG NG NN) fused to HA tag. Gn^R^ | [5] |
| dTALELBM1 | pBBR1MCS-5 expressing designer TALE (RVD repeat array: NI NH NG NN NI NI NG NG NI NH NG NG NG NN HD HD NG NG NN | This study |
| dTALELBM2 | pBBR1MCS-5 expressing designer TALE (RVD repeat array: NG NI NI NI NI HD HD NG HD NG NG NG NG NN HD HD NG NG NN) fused to HA tag. Gn^R^ | This study |
| dTALELB2A1 | Adapted variant of dTALELBM2 (RVD repeat array: NG NG NI NI NI HD HD NG HD NG NG NG NG NN HD HD NG NG NN) fused to HA tag. Gn^R^ | This study |
| dTALELB2A2 | Adapted variant of dTALELBM2 (RVD repeat array: NI NG NI NI NI HD HD NG HD NG NG NG NG NN) fused to HA tag. Gn^R^ | This study |
| dTALELBM3 | pBBR1MCS-5 expressing designer TALE (RVD repeat array: NG NI NG NI NI HD HD NG HD NG NG NG NG NN HD HD NG NG NN) fused to HA tag. Gn^R^ | This study |
| dTALELB3A | Adapted variant of dTALELBM3 (RVD repeat array: NG NI NI HD HD NG HD NG NG NG NG NN HD HD NG NG NN) fused to HA tag. Gn^R^ | This study |
| dTALELBM4 | pBBR1MCS-5 expressing designer TALE (RVD repeat array: HD NG HD NI NG NG NG HD NI NI NG NG NG NN HD HD NG NG NN) fused to HA tag. Gn^R^ | This study |
| dTALELBM5 | pBBR1MCS-5 expressing designer TALE (RVD repeat array: NI NG NI NI NI HD HD NG HD NG NG NI NI NN HD HD NG NG NN) fused to HA tag. Gn^R^ | This study |
| dTALELB5A | Adapted variant of dTALELBM5 (RVD repeat array: NI NG NI NI NI HD HD NG HD NG NG NI NG NN HD HD NG NG NN) fused to HA tag. Gn^R^ | This study |
| dTALELBM6 | pBBR1MCS-5 expressing designer TALE (RVD repeat array: HD HD NG NG NG HD NI NI NG HD NG NG NG NN HD HD NG NG NN) fused to HA tag. Gn^R^ | This study |
| dTALELBM7 | pBBR1MCS-5 expressing designer TALE (RVD repeat array: NI NG NI NG NI NI NI NI HD HD NG NG NG NN HD HD NG NG NN) fused to HA tag. Gn^R^ | [5] |
| dTALELB7A | Adapted variant of dTALELBM7 (RVD repeat array: NI NG NI NG NI NI NI HD HD HD HD HD NG NG NN) fused to HA tag. Gn^R^ | This study |
| pGEM-T | T vector for cloning LOB1 EBE PCR fragments from citrus. Ap^R^ | Promega, Madison, WI |
| pER8b | pER8 [7] derivative containing alternative MCS. Binary vector for 17β-estradiol inducible expression driven by the XVE system. Sp^R^ | [8] |
| pER8:PthA4 | For estradiol inducible expression of His-PthA4. Sp^R^ | This study |
| p1380-35S-GUS | Binary vector expression for overexpression of β‐Glucuronidase (*gus*) reporter. Kn^R^ | [9] |
| p1380-LOB1_sweet orange_-GUS | Binary vector expression for expression of β‐Glucuronidase (*gus*) reporter under the control of sweet orange (*Citrus sinensis*) *CsLOB1* promoter. Kn^R^ | This study |
| p1380-LOB1_swingle_ -GUS | Binary vector expression for expression of β‐Glucuronidase (*gus*) reporter under the control of Swingle citrumelo (*Poncirus trifoliata x Citrus paradisi*) *LOB1* promoter. Kn^R^ | This study |

*Kn^R^, Gn^R^ , Sp^R^ and Ap^R^ indicate resistance to kanamycin, gentamicin, spectinomycinand ampicillin, respectively.

**References:**

1. Da Silva ACR, Ferro JA, Reinach FC, Farah CS, Furlan LR, Quaggio RB, et al. Comparison of the genomes of two Xanthomonas pathogens with differing host specificities. Nature. 2002;417: 459–463. doi:10.1038/417459a

2. Yan Q, Wang N. High-throughput screening and analysis of genes of Xanthomonas citri subsp. citri involved in citrus canker symptom development. Mol Plant Microbe Interact. 2011;25: 1–72. doi:10.1094/MPMI-05-11-0121

3. Deblaere R, Bytebier B, De Greve H, Deboeck F, Schell J, Van Montagu M, et al. Efficient octopine Ti plasmid-derived vectors for Agrobacterium-mediated gene transfer to plants. Nucleic Acids Res. 1985;13: 4777–88. Available: http://www.ncbi.nlm.nih.gov/pubmed/4022773

4. Kovach ME, Elzer PH, Steven Hill D, Robertson GT, Farris MA, Roop RM, et al. Four new derivatives of the broad-host-range cloning vector pBBR1MCS, carrying different antibiotic-resistance cassettes. Gene. 1995;166: 175–176. doi:10.1016/0378-1119(95)00584-1

5. Teper D, Xu J, Li J, Wang N. The immunity of Meiwa kumquat against Xanthomonas citri is associated with a known susceptibility gene induced by a transcription activator-like effector. PLoS Pathog. 2020;16: e1008886. doi:10.1371/journal.ppat.1008886

6. Cermak T, Doyle EL, Christian M, Wang L, Zhang Y, Schmidt C, et al. Efficient design and assembly of custom TALEN and other TAL effector-based constructs for DNA targeting. Nucleic Acids Res. 2011;39: e82. doi:10.1093/nar/gkr218

7. Zuo J, Niu QW, Chua NH. Technical advance: An estrogen receptor-based transactivator XVE mediates highly inducible gene expression in transgenic plants. Plant J. 2000;24: 265–73. Available: http://www.ncbi.nlm.nih.gov/pubmed/11069700

8. Popov G, Fraiture M, Brunner F, Sessa G. Multiple Xanthomonas euvesicatoria Type III Effectors Inhibit flg22-Triggered Immunity. Mol Plant Microbe Interact. 2016;29: 651–60. doi:10.1094/MPMI-07-16-0137-R

9. Jia H, Wang N. Xcc-facilitated agroinfiltration of citrus leaves: a tool for rapid functional analysis of transgenes in citrus leaves. Plant Cell Rep. 2014;33: 1993–2001. doi:10.1007/s00299-014-1673-9
