# Supplementary material for: Consequences of adaptation of TAL effectors on host susceptibility to Xanthomonas
Source: PLoS Genet. 2021 Jan 19;17(1):e1009310. doi: 10.1371/journal.pgen.1009310 (PMC7845958; doi:10.1371/journal.pgen.1009310)
Supplement: S2 Table — (DOCX) [file pgen.1009310.s006.docx]

**S2 Table. Primers used in this study**

| Primer name | Sequence (5’ to 3’) (Underline represents restriction sites) | Destination vector |
| --- | --- | --- |
| *For construction of pBBRNPthF* | | |
| NPthF | AAAGTCGACCGACCATGTAAAGAGGTATGCCTG | pBBR1MCS-5 |
| NPthR | CCCGAATTCCGAAGCGTCGGAGGGTTGCG | pBBR1MCS-5 |
| *For construction of p1380-LOB1_sweet orange_-GUS and p1380-LOB1_swingle_-GUS* | | |
| FLpLOB1F | AAACTGCAGAAGCTGAAAATGAGCCTCTTATTTTCAAGGTGC | p1380-35S-GUS |
| FLpLOB1R | CCCGGATCCCTACATTAATTTTGTGTTTGCATTCCATTTTTGAGAGAAG | p1380-35S-GUS |
| *For amplification of TALE EBE region in the promoters of Rutaceae LOB1* | | |
| LOB1EBEF | AATGACATCATCTAGTGGCTCGGTGAC | pGEM-T |
| LOB1EBER | TGAGAGAAGAAAACTGTTGGGTTGTAG | pGEM-T |
| *For sequencing of dTALE* | | |
| M13F(-21) | TGTAAAACGACGGCCAGT | |
| M13R Reverse | CAGGAAACAGCTATGAC | |
| TAL_Seq_5-1 | CATCGCGCAATGCACTGAC | |
| TAL_R3 | GGCTCAGCTGGGCCACAATG | |
| TALmidseq5 | TCAGCTATCAGCACATAATCACG | |
| TALmidseq3 | ACGTGCGTTCGCCAATAC | |
| *For qRT-PCR* | | |
| RTCsGAPDHF | GAAAGGTCTTGCCTGCTTTG | |
| RTCsGAPDHR | TCCTTCTCCAGCCTCACTGT | |
| RTCsLOB1F | TCCACCAACCGAACCATACA | |
| RTCsLOB1R | GGCACTTGCTTCATAGACCAT | |
