# Supplementary material for: Consequences of adaptation of TAL effectors on host susceptibility to Xanthomonas
Source: PLoS Genet. 2021 Jan 19;17(1):e1009310. doi: 10.1371/journal.pgen.1009310 (PMC7845958; doi:10.1371/journal.pgen.1009310)
Supplement: S1 Text — (DOCX) [file pgen.1009310.s007.docx]

**S1_Text. dTALEs used in this study**

The following DNA sequences represent original and adapted dTALEs used in this study. Start codons are underlined. The DNA sequences that correspond to the RVDs of each repeat are color-coded according the following color scheme:

Red: NI/AACATT

Green: NG/AACGGT

Yellow: HD/CACGAT

Blue: NN/AACAAT

Purple: NH/AATCAC

>dTALEWTLOB1

CGACCATGTAAAGAGGTATGCCTGATGGATCCCATTCGTTCGCGCACACCAAGTCCTGCCCGCGAGCTTCTGCCCGGACCCCAACCCGATGGGGTTCAGCCGACTGCAGATCGTGGGGTGTCTGCGCCTGCTGGCAGCCCTCTGGATGGCTTGCCCGCTCGGCGGACGGTGTCCCGGACCCGGCTGCCATCTCCCCCTGCGCCCTCACCTGCGTTCTCGGCGGGCAGCTTCAGCGATCTGCTCCGTCCGTTCGATCCGTCGCTTCTTGATACATCGCTTCTTGATTCGATGCCTGCCGTCGGCACGCCGCATACAGCGGCTGCCCCAGCAGAGTGGGATGAGGCGCAATCGGCTCTGCGTGCAGCCGATGACCCGCCACCCACCGTGCGTGTCGCTGTCACTGCCGCGCGGCCGCCGCGCGCCAAGCCGGCCCCGCGACGGCGTGCTGCGCAACCCTCCGACGCTTCGCCGGCCGCGCAGGTGGATCTACGCACGCTCGGCTACAGTCAGCAGCAGCAAGAGAAGATCAAACCGAAGGTGCGTTCGACAGTGGCGCAGCACCACGAGGCACTGGTGGGCCATGGGTTTACACACGCGCACATCGTTGCGCTCAGCCAACACCCGGCAGCGTTAGGGACCGTCGCTGTCACGTATCAGCACATAATCACGGCGTTGCCAGAGGCGACACACGAAGACATCGTTGGCGTCGGCAAACAGTGGTCCGGCGCACGCGCCCTGGAGGCCTTGCTCACGGATGCGGGGGAGTTGAGAGGTCCGCCGTTACAGTTGGACACAGGCCAACTTGTGAAGATTGCAAAACGTGGCGGCGTGACCGCAATGGAGGCAGTGCATGCATCGCGCAATGCACTGACGGGTGCCCCCCTGAACCTGACCCCGGACCAAGTGGTGGCTATCGCCAGCAACATTGGCGGCAAGCAAGCGCTCGAAACGGTGCAGCGGCTGTTGCCGGTGCTGTGCCAGGACCATGGCCTGACCCCGGACCAAGTGGTGGCTATCGCCAGCAACGGTGGCGGCAAGCAAGCGCTCGAAACGGTGCAGCGGCTGTTGCCGGTGCTGTGCCAGGACCATGGCCTGACCCCGGACCAAGTGGTGGCTATCGCCAGCAACATTGGCGGCAAGCAAGCGCTCGAAACGGTGCAGCGGCTGTTGCCGGTGCTGTGCCAGGACCATGGCCTGACCCCGGACCAAGTGGTGGCTATCGCCAGCAACATTGGCGGCAAGCAAGCGCTCGAAACGGTGCAGCGGCTGTTGCCGGTGCTGTGCCAGGACCATGGCCTGACCCCGGACCAAGTGGTGGCTATCGCCAGCAACATTGGCGGCAAGCAAGCGCTCGAAACGGTGCAGCGGCTGTTGCCGGTGCTGTGCCAGGACCATGGCCTGACTCCGGACCAAGTGGTGGCTATCGCCAGCCACGATGGCGGCAAGCAAGCGCTCGAAACGGTGCAGCGGCTGTTGCCGGTGCTGTGCCAGGACCATGGCCTGACTCCGGACCAAGTGGTGGCTATCGCCAGCCACGATGGCGGCAAGCAAGCGCTCGAAACGGTGCAGCGGCTGTTGCCGGTGCTGTGCCAGGACCATGGCCTGACCCCGGACCAAGTGGTGGCTATCGCCAGCAACGGTGGCGGCAAGCAAGCGCTCGAAACGGTGCAGCGGCTGTTGCCGGTGCTGTGCCAGGACCATGGCCTGACTCCGGACCAAGTGGTGGCTATCGCCAGCCACGATGGCGGCAAGCAAGCGCTCGAAACGGTGCAGCGGCTGTTGCCGGTGCTGTGCCAGGACCATGGCCTGACCCCGGACCAAGTGGTGGCTATCGCCAGCAACGGTGGCGGCAAGCAAGCGCTCGAAACGGTGCAGCGGCTGTTGCCGGTGCTGTGCCAGGACCATGGCCTGACCCCGGACCAAGTGGTGGCTATCGCCAGCAACGGTGGCGGCAAGCAAGCGCTCGAAACGGTGCAGCGGCTGTTGCCGGTGCTGTGCCAGGACCATGGCCTGACCCCGGACCAAGTGGTGGCTATCGCCAGCAACGGTGGCGGCAAGCAAGCGCTCGAAACGGTGCAGCGGCTGTTGCCGGTGCTGTGCCAGGACCATGGCCTGACCCCGGACCAAGTGGTGGCTATCGCCAGCAACGGTGGCGGCAAGCAAGCGCTCGAAACGGTGCAGCGGCTGTTGCCGGTGCTGTGCCAGGACCATGGCCTGACCCCGGACCAAGTGGTGGCTATCGCCAGCAACAATGGCGGCAAGCAAGCGCTCGAAACGGTGCAGCGGCTGTTGCCGGTGCTGTGCCAGGACCATGGCCTGACTCCGGACCAAGTGGTGGCTATCGCCAGCCACGATGGCGGCAAGCAAGCGCTCGAAACGGTGCAGCGGCTGTTGCCGGTGCTGTGCCAGGACCATGGCCTGACTCCGGACCAAGTGGTGGCTATCGCCAGCCACGATGGCGGCAAGCAAGCGCTCGAAACGGTGCAGCGGCTGTTGCCGGTGCTGTGCCAGGACCATGGCCTGACCCCGGACCAAGTGGTGGCTATCGCCAGCAACGGTGGCGGCAAGCAAGCGCTCGAAACGGTGCAGCGGCTGTTGCCGGTGCTGTGCCAGGACCATGGCCTGACCCCGGACCAAGTGGTGGCTATCGCCAGCAACGGTGGCGGCAAGCAAGCGCTCGAAACGGTGCAGCGGCTGTTGCCGGTGCTGTGCCAGGACCATGGCCTGACCCCGGACCAAGTGGTGGCTATCGCCAGCAACAATGGCGGCAAGCAAGCGCTCGAAAGCATTGTGGCCCAGCTGAGCCGGCCTGATCCGGCGTTGGCCGCGTTGACCAACGACCACCTCGTCGCCTTGGCCTGCCTCGGCGGACGTCCTGCCATGGATGCAGTGAAAAAGGGATTGCCGCACGCGCCGGAATTGATCAGAAGAGTCAATCGCCGTATTGGCGAACGCACGTCCCATCGCGTTGCCGACTACGCGCAAGTGGTTCGCGTGCTGGAGTTTTTCCAGTGCCACTCCCACCCAGCGTACGCATTTGATGAGGCCATGACGCAGTTCGGGATGAGCAGGAACGGGTTGGTACAGCTCTTTCGCAGAGTGGGCGTCACCGAACTCGAAGCCCGCGGTGGAACGCTCCCCCCAGCCTCGCAGCGTTGGGACCGTATCCTCCAGGCATCAGGGATGAAAAGGGCCAAACCGTCCCCTACTTCAGCTCAAACACCGGATCAGGCGTCTTTGCATGCATTCGCCGATTCGCTGGAGCGTGACCTTGATGCGCCCAGCCCAATGCACGAGGGAGATCAGACGCGGGCAAGCAGCCGTAAACGGTCCCGATCGGATCGTGCTGTCACCGGCCCCTCCGCACAGCAGGCTGTCGAGGTGCGCGTTCCCGAACAGCGCGATGCGCTGCATTTGCCCCTCAGCTGGAGGGTAAAACGCCCGCGTACCAGGATCTGGGGCGGCCTCCCGGATCCTGGTACGCCCATGGCTGCCGACCTGGCAGCGTCCAGCACCGTGATGTGGGAACAAGATGCGGACCCCTTCGCAGGGGCAGCGGATGATTTCCCGGCATTCAACGAAGAGGAACTCGCATGGTTGATGGAGCTATTGCCTCAGAAGGGCGAATTCAAGCTTTACCCATACGATGTTCCAGATTACGCTTGA

>dTALELBM1

CGACCATGTAAAGAGGTATGCCTGATGGATCCCATTCGTTCGCGCACACCAAGTCCTGCCCGCGAGCTTCTGCCCGGACCCCAACCCGATGGGGTTCAGCCGACTGCAGATCGTGGGGTGTCTGCGCCTGCTGGCAGCCCTCTGGATGGCTTGCCCGCTCGGCGGACGGTGTCCCGGACCCGGCTGCCATCTCCCCCTGCGCCCTCACCTGCGTTCTCGGCGGGCAGCTTCAGCGATCTGCTCCGTCCGTTCGATCCGTCGCTTCTTGATACATCGCTTCTTGATTCGATGCCTGCCGTCGGCACGCCGCATACAGCGGCTGCCCCAGCAGAGTGGGATGAGGCGCAATCGGCTCTGCGTGCAGCCGATGACCCGCCACCCACCGTGCGTGTCGCTGTCACTGCCGCGCGGCCGCCGCGCGCCAAGCCGGCCCCGCGACGGCGTGCTGCGCAACCCTCCGACGCTTCGCCGGCCGCGCAGGTGGATCTACGCACGCTCGGCTACAGTCAGCAGCAGCAAGAGAAGATCAAACCGAAGGTGCGTTCGACAGTGGCGCAGCACCACGAGGCACTGGTGGGCCATGGGTTTACACACGCGCACATCGTTGCGCTCAGCCAACACCCGGCAGCGTTAGGGACCGTCGCTGTCACGTATCAGCACATAATCACGGCGTTGCCAGAGGCGACACACGAAGACATCGTTGGCGTCGGCAAACAGTGGTCCGGCGCACGCGCCCTGGAGGCCTTGCTCACGGATGCGGGGGAGTTGAGAGGTCCGCCGTTACAGTTGGACACAGGCCAACTTGTGAAGATTGCAAAACGTGGCGGCGTGACCGCAATGGAGGCAGTGCATGCATCGCGCAATGCACTGACGGGTGCCCCCCTGAACCTGACCCCGGACCAAGTGGTGGCTATCGCCAGCAACATTGGCGGCAAGCAAGCGCTCGAAACGGTGCAGCGGCTGTTGCCGGTGCTGTGCCAGGACCATGGCCTGACCCCGGACCAAGTGGTGGCTATCGCCAGCAATCACGGCGGCAAGCAAGCGCTCGAAACGGTGCAGCGGCTGTTGCCGGTGCTGTGCCAGGACCATGGCCTGACCCCGGACCAAGTGGTGGCTATCGCCAGCAACGGTGGCGGCAAGCAAGCGCTCGAAACGGTGCAGCGGCTGTTGCCGGTGCTGTGCCAGGACCATGGCCTGACCCCGGACCAAGTGGTGGCTATCGCCAGCAACAATGGCGGCAAGCAAGCGCTCGAAACGGTGCAGCGGCTGTTGCCGGTGCTGTGCCAGGACCATGGCCTGACCCCGGACCAAGTGGTGGCTATCGCCAGCAACATTGGCGGCAAGCAAGCGCTCGAAACGGTGCAGCGGCTGTTGCCGGTGCTGTGCCAGGACCATGGCCTGACTCCGGACCAAGTGGTGGCTATCGCCAGCAACATTGGCGGCAAGCAAGCGCTCGAAACGGTGCAGCGGCTGTTGCCGGTGCTGTGCCAGGACCATGGCCTGACTCCGGACCAAGTGGTGGCTATCGCCAGCAACGGTGGCGGCAAGCAAGCGCTCGAAACGGTGCAGCGGCTGTTGCCGGTGCTGTGCCAGGACCATGGCCTGACCCCGGACCAAGTGGTGGCTATCGCCAGCAACGGTGGCGGCAAGCAAGCGCTCGAAACGGTGCAGCGGCTGTTGCCGGTGCTGTGCCAGGACCATGGCCTGACTCCGGACCAAGTGGTGGCTATCGCCAGCAACATTGGCGGCAAGCAAGCGCTCGAAACGGTGCAGCGGCTGTTGCCGGTGCTGTGCCAGGACCATGGCCTGACCCCGGACCAAGTGGTGGCTATCGCCAGCAATCACGGCGGCAAGCAAGCGCTCGAAACGGTGCAGCGGCTGTTGCCGGTGCTGTGCCAGGACCATGGCCTGACCCCGGACCAAGTGGTGGCTATCGCCAGCAACGGTGGCGGCAAGCAAGCGCTCGAAACGGTGCAGCGGCTGTTGCCGGTGCTGTGCCAGGACCATGGCCTGACCCCGGACCAAGTGGTGGCTATCGCCAGCAACGGTGGCGGCAAGCAAGCGCTCGAAACGGTGCAGCGGCTGTTGCCGGTGCTGTGCCAGGACCATGGCCTGACCCCGGACCAAGTGGTGGCTATCGCCAGCAACGGTGGCGGCAAGCAAGCGCTCGAAACGGTGCAGCGGCTGTTGCCGGTGCTGTGCCAGGACCATGGCCTGACCCCGGACCAAGTGGTGGCTATCGCCAGCAACAATGGCGGCAAGCAAGCGCTCGAAACGGTGCAGCGGCTGTTGCCGGTGCTGTGCCAGGACCATGGCCTGACTCCGGACCAAGTGGTGGCTATCGCCAGCCACGATGGCGGCAAGCAAGCGCTCGAAACGGTGCAGCGGCTGTTGCCGGTGCTGTGCCAGGACCATGGCCTGACTCCGGACCAAGTGGTGGCTATCGCCAGCCACGATGGCGGCAAGCAAGCGCTCGAAACGGTGCAGCGGCTGTTGCCGGTGCTGTGCCAGGACCATGGCCTGACCCCGGACCAAGTGGTGGCTATCGCCAGCAACGGTGGCGGCAAGCAAGCGCTCGAAACGGTGCAGCGGCTGTTGCCGGTGCTGTGCCAGGACCATGGCCTGACCCCGGACCAAGTGGTGGCTATCGCCAGCAACGGTGGCGGCAAGCAAGCGCTCGAAACGGTGCAGCGGCTGTTGCCGGTGCTGTGCCAGGACCATGGCCTGACCCCGGACCAAGTGGTGGCTATCGCCAGCAACAATGGCGGCAAGCAAGCGCTCGAAAGCATTGTGGCCCAGCTGAGCCGGCCTGATCCGGCGTTGGCCGCGTTGACCAACGACCACCTCGTCGCCTTGGCCTGCCTCGGCGGACGTCCTGCCATGGATGCAGTGAAAAAGGGATTGCCGCACGCGCCGGAATTGATCAGAAGAGTCAATCGCCGTATTGGCGAACGCACGTCCCATCGCGTTGCCGACTACGCGCAAGTGGTTCGCGTGCTGGAGTTTTTCCAGTGCCACTCCCACCCAGCGTACGCATTTGATGAGGCCATGACGCAGTTCGGGATGAGCAGGAACGGGTTGGTACAGCTCTTTCGCAGAGTGGGCGTCACCGAACTCGAAGCCCGCGGTGGAACGCTCCCCCCAGCCTCGCAGCGTTGGGACCGTATCCTCCAGGCATCAGGGATGAAAAGGGCCAAACCGTCCCCTACTTCAGCTCAAACACCGGATCAGGCGTCTTTGCATGCATTCGCCGATTCGCTGGAGCGTGACCTTGATGCGCCCAGCCCAATGCACGAGGGAGATCAGACGCGGGCAAGCAGCCGTAAACGGTCCCGATCGGATCGTGCTGTCACCGGCCCCTCCGCACAGCAGGCTGTCGAGGTGCGCGTTCCCGAACAGCGCGATGCGCTGCATTTGCCCCTCAGCTGGAGGGTAAAACGCCCGCGTACCAGGATCTGGGGCGGCCTCCCGGATCCTGGTACGCCCATGGCTGCCGACCTGGCAGCGTCCAGCACCGTGATGTGGGAACAAGATGCGGACCCCTTCGCAGGGGCAGCGGATGATTTCCCGGCATTCAACGAAGAGGAACTCGCATGGTTGATGGAGCTATTGCCTCAGAAGGGCGAATTCAAGCTTTACCCATACGATGTTCCAGATTACGCTTGA

>dTALELBM2

CGACCATGTAAAGAGGTATGCCTGATGGATCCCATTCGTTCGCGCACACCAAGTCCTGCCCGCGAGCTTCTGCCCGGACCCCAACCCGATGGGGTTCAGCCGACTGCAGATCGTGGGGTGTCTGCGCCTGCTGGCAGCCCTCTGGATGGCTTGCCCGCTCGGCGGACGGTGTCCCGGACCCGGCTGCCATCTCCCCCTGCGCCCTCACCTGCGTTCTCGGCGGGCAGCTTCAGCGATCTGCTCCGTCCGTTCGATCCGTCGCTTCTTGATACATCGCTTCTTGATTCGATGCCTGCCGTCGGCACGCCGCATACAGCGGCTGCCCCAGCAGAGTGGGATGAGGCGCAATCGGCTCTGCGTGCAGCCGATGACCCGCCACCCACCGTGCGTGTCGCTGTCACTGCCGCGCGGCCGCCGCGCGCCAAGCCGGCCCCGCGACGGCGTGCTGCGCAACCCTCCGACGCTTCGCCGGCCGCGCAGGTGGATCTACGCACGCTCGGCTACAGTCAGCAGCAGCAAGAGAAGATCAAACCGAAGGTGCGTTCGACAGTGGCGCAGCACCACGAGGCACTGGTGGGCCATGGGTTTACACACGCGCACATCGTTGCGCTCAGCCAACACCCGGCAGCGTTAGGGACCGTCGCTGTCACGTATCAGCACATAATCACGGCGTTGCCAGAGGCGACACACGAAGACATCGTTGGCGTCGGCAAACAGTGGTCCGGCGCACGCGCCCTGGAGGCCTTGCTCACGGATGCGGGGGAGTTGAGAGGTCCGCCGTTACAGTTGGACACAGGCCAACTTGTGAAGATTGCAAAACGTGGCGGCGTGACCGCAATGGAGGCAGTGCATGCATCGCGCAATGCACTGACGGGTGCCCCCCTGAACCTGACCCCGGACCAAGTGGTGGCTATCGCCAGCAACGGTGGCGGCAAGCAAGCGCTCGAAACGGTGCAGCGGCTGTTGCCGGTGCTGTGCCAGGACCATGGCCTGACCCCGGACCAAGTGGTGGCTATCGCCAGCAACATTGGCGGCAAGCAAGCGCTCGAAACGGTGCAGCGGCTGTTGCCGGTGCTGTGCCAGGACCATGGCCTGACCCCGGACCAAGTGGTGGCTATCGCCAGCAACATTGGCGGCAAGCAAGCGCTCGAAACGGTGCAGCGGCTGTTGCCGGTGCTGTGCCAGGACCATGGCCTGACCCCGGACCAAGTGGTGGCTATCGCCAGCAACATTGGCGGCAAGCAAGCGCTCGAAACGGTGCAGCGGCTGTTGCCGGTGCTGTGCCAGGACCATGGCCTGACCCCGGACCAAGTGGTGGCTATCGCCAGCAACATTGGCGGCAAGCAAGCGCTCGAAACGGTGCAGCGGCTGTTGCCGGTGCTGTGCCAGGACCATGGCCTGACTCCGGACCAAGTGGTGGCTATCGCCAGCCACGATGGCGGCAAGCAAGCGCTCGAAACGGTGCAGCGGCTGTTGCCGGTGCTGTGCCAGGACCATGGCCTGACTCCGGACCAAGTGGTGGCTATCGCCAGCCACGATGGCGGCAAGCAAGCGCTCGAAACGGTGCAGCGGCTGTTGCCGGTGCTGTGCCAGGACCATGGCCTGACCCCGGACCAAGTGGTGGCTATCGCCAGCAACGGTGGCGGCAAGCAAGCGCTCGAAACGGTGCAGCGGCTGTTGCCGGTGCTGTGCCAGGACCATGGCCTGACTCCGGACCAAGTGGTGGCTATCGCCAGCCACGATGGCGGCAAGCAAGCGCTCGAAACGGTGCAGCGGCTGTTGCCGGTGCTGTGCCAGGACCATGGCCTGACCCCGGACCAAGTGGTGGCTATCGCCAGCAACGGTGGCGGCAAGCAAGCGCTCGAAACGGTGCAGCGGCTGTTGCCGGTGCTGTGCCAGGACCATGGCCTGACCCCGGACCAAGTGGTGGCTATCGCCAGCAACGGTGGCGGCAAGCAAGCGCTCGAAACGGTGCAGCGGCTGTTGCCGGTGCTGTGCCAGGACCATGGCCTGACCCCGGACCAAGTGGTGGCTATCGCCAGCAACGGTGGCGGCAAGCAAGCGCTCGAAACGGTGCAGCGGCTGTTGCCGGTGCTGTGCCAGGACCATGGCCTGACCCCGGACCAAGTGGTGGCTATCGCCAGCAACGGTGGCGGCAAGCAAGCGCTCGAAACGGTGCAGCGGCTGTTGCCGGTGCTGTGCCAGGACCATGGCCTGACCCCGGACCAAGTGGTGGCTATCGCCAGCAACAATGGCGGCAAGCAAGCGCTCGAAACGGTGCAGCGGCTGTTGCCGGTGCTGTGCCAGGACCATGGCCTGACTCCGGACCAAGTGGTGGCTATCGCCAGCCACGATGGCGGCAAGCAAGCGCTCGAAACGGTGCAGCGGCTGTTGCCGGTGCTGTGCCAGGACCATGGCCTGACTCCGGACCAAGTGGTGGCTATCGCCAGCCACGATGGCGGCAAGCAAGCGCTCGAAACGGTGCAGCGGCTGTTGCCGGTGCTGTGCCAGGACCATGGCCTGACCCCGGACCAAGTGGTGGCTATCGCCAGCAACGGTGGCGGCAAGCAAGCGCTCGAAACGGTGCAGCGGCTGTTGCCGGTGCTGTGCCAGGACCATGGCCTGACCCCGGACCAAGTGGTGGCTATCGCCAGCAACGGTGGCGGCAAGCAAGCGCTCGAAACGGTGCAGCGGCTGTTGCCGGTGCTGTGCCAGGACCATGGCCTGACCCCGGACCAAGTGGTGGCTATCGCCAGCAACAATGGCGGCAAGCAAGCGCTCGAAAGCATTGTGGCCCAGCTGAGCCGGCCTGATCCGGCGTTGGCCGCGTTGACCAACGACCACCTCGTCGCCTTGGCCTGCCTCGGCGGACGTCCTGCCATGGATGCAGTGAAAAAGGGATTGCCGCACGCGCCGGAATTGATCAGAAGAGTCAATCGCCGTATTGGCGAACGCACGTCCCATCGCGTTGCCGACTACGCGCAAGTGGTTCGCGTGCTGGAGTTTTTCCAGTGCCACTCCCACCCAGCGTACGCATTTGATGAGGCCATGACGCAGTTCGGGATGAGCAGGAACGGGTTGGTACAGCTCTTTCGCAGAGTGGGCGTCACCGAACTCGAAGCCCGCGGTGGAACGCTCCCCCCAGCCTCGCAGCGTTGGGACCGTATCCTCCAGGCATCAGGGATGAAAAGGGCCAAACCGTCCCCTACTTCAGCTCAAACACCGGATCAGGCGTCTTTGCATGCATTCGCCGATTCGCTGGAGCGTGACCTTGATGCGCCCAGCCCAATGCACGAGGGAGATCAGACGCGGGCAAGCAGCCGTAAACGGTCCCGATCGGATCGTGCTGTCACCGGCCCCTCCGCACAGCAGGCTGTCGAGGTGCGCGTTCCCGAACAGCGCGATGCGCTGCATTTGCCCCTCAGCTGGAGGGTAAAACGCCCGCGTACCAGGATCTGGGGCGGCCTCCCGGATCCTGGTACGCCCATGGCTGCCGACCTGGCAGCGTCCAGCACCGTGATGTGGGAACAAGATGCGGACCCCTTCGCAGGGGCAGCGGATGATTTCCCGGCATTCAACGAAGAGGAACTCGCATGGTTGATGGAGCTATTGCCTCAGAAGGGCGAATTCAAGCTTTACCCATACGATGTTCCAGATTACGCTTGA

>dTALELBM3

CGACCATGTAAAGAGGTATGCCTGATGGATCCCATTCGTTCGCGCACACCAAGTCCTGCCCGCGAGCTTCTGCCCGGACCCCAACCCGATGGGGTTCAGCCGACTGCAGATCGTGGGGTGTCTGCGCCTGCTGGCAGCCCTCTGGATGGCTTGCCCGCTCGGCGGACGGTGTCCCGGACCCGGCTGCCATCTCCCCCTGCGCCCTCACCTGCGTTCTCGGCGGGCAGCTTCAGCGATCTGCTCCGTCCGTTCGATCCGTCGCTTCTTGATACATCGCTTCTTGATTCGATGCCTGCCGTCGGCACGCCGCATACAGCGGCTGCCCCAGCAGAGTGGGATGAGGCGCAATCGGCTCTGCGTGCAGCCGATGACCCGCCACCCACCGTGCGTGTCGCTGTCACTGCCGCGCGGCCGCCGCGCGCCAAGCCGGCCCCGCGACGGCGTGCTGCGCAACCCTCCGACGCTTCGCCGGCCGCGCAGGTGGATCTACGCACGCTCGGCTACAGTCAGCAGCAGCAAGAGAAGATCAAACCGAAGGTGCGTTCGACAGTGGCGCAGCACCACGAGGCACTGGTGGGCCATGGGTTTACACACGCGCACATCGTTGCGCTCAGCCAACACCCGGCAGCGTTAGGGACCGTCGCTGTCACGTATCAGCACATAATCACGGCGTTGCCAGAGGCGACACACGAAGACATCGTTGGCGTCGGCAAACAGTGGTCCGGCGCACGCGCCCTGGAGGCCTTGCTCACGGATGCGGGGGAGTTGAGAGGTCCGCCGTTACAGTTGGACACAGGCCAACTTGTGAAGATTGCAAAACGTGGCGGCGTGACCGCAATGGAGGCAGTGCATGCATCGCGCAATGCACTGACGGGTGCCCCCCTGAACCTGACCCCGGACCAAGTGGTGGCTATCGCCAGCAACGGTGGCGGCAAGCAAGCGCTCGAAACGGTGCAGCGGCTGTTGCCGGTGCTGTGCCAGGACCATGGCCTGACCCCGGACCAAGTGGTGGCTATCGCCAGCAACATTGGCGGCAAGCAAGCGCTCGAAACGGTGCAGCGGCTGTTGCCGGTGCTGTGCCAGGACCATGGCCTGACCCCGGACCAAGTGGTGGCTATCGCCAGCAACGGTGGCGGCAAGCAAGCGCTCGAAACGGTGCAGCGGCTGTTGCCGGTGCTGTGCCAGGACCATGGCCTGACCCCGGACCAAGTGGTGGCTATCGCCAGCAACATTGGCGGCAAGCAAGCGCTCGAAACGGTGCAGCGGCTGTTGCCGGTGCTGTGCCAGGACCATGGCCTGACCCCGGACCAAGTGGTGGCTATCGCCAGCAACATTGGCGGCAAGCAAGCGCTCGAAACGGTGCAGCGGCTGTTGCCGGTGCTGTGCCAGGACCATGGCCTGACTCCGGACCAAGTGGTGGCTATCGCCAGCCACGATGGCGGCAAGCAAGCGCTCGAAACGGTGCAGCGGCTGTTGCCGGTGCTGTGCCAGGACCATGGCCTGACTCCGGACCAAGTGGTGGCTATCGCCAGCCACGATGGCGGCAAGCAAGCGCTCGAAACGGTGCAGCGGCTGTTGCCGGTGCTGTGCCAGGACCATGGCCTGACCCCGGACCAAGTGGTGGCTATCGCCAGCAACGGTGGCGGCAAGCAAGCGCTCGAAACGGTGCAGCGGCTGTTGCCGGTGCTGTGCCAGGACCATGGCCTGACTCCGGACCAAGTGGTGGCTATCGCCAGCCACGATGGCGGCAAGCAAGCGCTCGAAACGGTGCAGCGGCTGTTGCCGGTGCTGTGCCAGGACCATGGCCTGACCCCGGACCAAGTGGTGGCTATCGCCAGCAACGGTGGCGGCAAGCAAGCGCTCGAAACGGTGCAGCGGCTGTTGCCGGTGCTGTGCCAGGACCATGGCCTGACCCCGGACCAAGTGGTGGCTATCGCCAGCAACGGTGGCGGCAAGCAAGCGCTCGAAACGGTGCAGCGGCTGTTGCCGGTGCTGTGCCAGGACCATGGCCTGACCCCGGACCAAGTGGTGGCTATCGCCAGCAACGGTGGCGGCAAGCAAGCGCTCGAAACGGTGCAGCGGCTGTTGCCGGTGCTGTGCCAGGACCATGGCCTGACCCCGGACCAAGTGGTGGCTATCGCCAGCAACGGTGGCGGCAAGCAAGCGCTCGAAACGGTGCAGCGGCTGTTGCCGGTGCTGTGCCAGGACCATGGCCTGACCCCGGACCAAGTGGTGGCTATCGCCAGCAACAATGGCGGCAAGCAAGCGCTCGAAACGGTGCAGCGGCTGTTGCCGGTGCTGTGCCAGGACCATGGCCTGACTCCGGACCAAGTGGTGGCTATCGCCAGCCACGATGGCGGCAAGCAAGCGCTCGAAACGGTGCAGCGGCTGTTGCCGGTGCTGTGCCAGGACCATGGCCTGACTCCGGACCAAGTGGTGGCTATCGCCAGCCACGATGGCGGCAAGCAAGCGCTCGAAACGGTGCAGCGGCTGTTGCCGGTGCTGTGCCAGGACCATGGCCTGACCCCGGACCAAGTGGTGGCTATCGCCAGCAACGGTGGCGGCAAGCAAGCGCTCGAAACGGTGCAGCGGCTGTTGCCGGTGCTGTGCCAGGACCATGGCCTGACCCCGGACCAAGTGGTGGCTATCGCCAGCAACGGTGGCGGCAAGCAAGCGCTCGAAACGGTGCAGCGGCTGTTGCCGGTGCTGTGCCAGGACCATGGCCTGACCCCGGACCAAGTGGTGGCTATCGCCAGCAACAATGGCGGCAAGCAAGCGCTCGAAAGCATTGTGGCCCAGCTGAGCCGGCCTGATCCGGCGTTGGCCGCGTTGACCAACGACCACCTCGTCGCCTTGGCCTGCCTCGGCGGACGTCCTGCCATGGATGCAGTGAAAAAGGGATTGCCGCACGCGCCGGAATTGATCAGAAGAGTCAATCGCCGTATTGGCGAACGCACGTCCCATCGCGTTGCCGACTACGCGCAAGTGGTTCGCGTGCTGGAGTTTTTCCAGTGCCACTCCCACCCAGCGTACGCATTTGATGAGGCCATGACGCAGTTCGGGATGAGCAGGAACGGGTTGGTACAGCTCTTTCGCAGAGTGGGCGTCACCGAACTCGAAGCCCGCGGTGGAACGCTCCCCCCAGCCTCGCAGCGTTGGGACCGTATCCTCCAGGCATCAGGGATGAAAAGGGCCAAACCGTCCCCTACTTCAGCTCAAACACCGGATCAGGCGTCTTTGCATGCATTCGCCGATTCGCTGGAGCGTGACCTTGATGCGCCCAGCCCAATGCACGAGGGAGATCAGACGCGGGCAAGCAGCCGTAAACGGTCCCGATCGGATCGTGCTGTCACCGGCCCCTCCGCACAGCAGGCTGTCGAGGTGCGCGTTCCCGAACAGCGCGATGCGCTGCATTTGCCCCTCAGCTGGAGGGTAAAACGCCCGCGTACCAGGATCTGGGGCGGCCTCCCGGATCCTGGTACGCCCATGGCTGCCGACCTGGCAGCGTCCAGCACCGTGATGTGGGAACAAGATGCGGACCCCTTCGCAGGGGCAGCGGATGATTTCCCGGCATTCAACGAAGAGGAACTCGCATGGTTGATGGAGCTATTGCCTCAGAAGGGCGAATTCAAGCTTTACCCATACGATGTTCCAGATTACGCTTGA

>dTALELBM4

CGACCATGTAAAGAGGTATGCCTGATGGATCCCATTCGTTCGCGCACACCAAGTCCTGCCCGCGAGCTTCTGCCCGGACCCCAACCCGATGGGGTTCAGCCGACTGCAGATCGTGGGGTGTCTGCGCCTGCTGGCAGCCCTCTGGATGGCTTGCCCGCTCGGCGGACGGTGTCCCGGACCCGGCTGCCATCTCCCCCTGCGCCCTCACCTGCGTTCTCGGCGGGCAGCTTCAGCGATCTGCTCCGTCCGTTCGATCCGTCGCTTCTTGATACATCGCTTCTTGATTCGATGCCTGCCGTCGGCACGCCGCATACAGCGGCTGCCCCAGCAGAGTGGGATGAGGCGCAATCGGCTCTGCGTGCAGCCGATGACCCGCCACCCACCGTGCGTGTCGCTGTCACTGCCGCGCGGCCGCCGCGCGCCAAGCCGGCCCCGCGACGGCGTGCTGCGCAACCCTCCGACGCTTCGCCGGCCGCGCAGGTGGATCTACGCACGCTCGGCTACAGTCAGCAGCAGCAAGAGAAGATCAAACCGAAGGTGCGTTCGACAGTGGCGCAGCACCACGAGGCACTGGTGGGCCATGGGTTTACACACGCGCACATCGTTGCGCTCAGCCAACACCCGGCAGCGTTAGGGACCGTCGCTGTCACGTATCAGCACATAATCACGGCGTTGCCAGAGGCGACACACGAAGACATCGTTGGCGTCGGCAAACAGTGGTCCGGCGCACGCGCCCTGGAGGCCTTGCTCACGGATGCGGGGGAGTTGAGAGGTCCGCCGTTACAGTTGGACACAGGCCAACTTGTGAAGATTGCAAAACGTGGCGGCGTGACCGCAATGGAGGCAGTGCATGCATCGCGCAATGCACTGACGGGTGCCCCCCTGAACCTGACCCCGGACCAAGTGGTGGCTATCGCCAGCCACGATGGCGGCAAGCAAGCGCTCGAAACGGTGCAGCGGCTGTTGCCGGTGCTGTGCCAGGACCATGGCCTGACCCCGGACCAAGTGGTGGCTATCGCCAGCAACGGTGGCGGCAAGCAAGCGCTCGAAACGGTGCAGCGGCTGTTGCCGGTGCTGTGCCAGGACCATGGCCTGACCCCGGACCAAGTGGTGGCTATCGCCAGCCACGATGGCGGCAAGCAAGCGCTCGAAACGGTGCAGCGGCTGTTGCCGGTGCTGTGCCAGGACCATGGCCTGACCCCGGACCAAGTGGTGGCTATCGCCAGCAACATTGGCGGCAAGCAAGCGCTCGAAACGGTGCAGCGGCTGTTGCCGGTGCTGTGCCAGGACCATGGCCTGACCCCGGACCAAGTGGTGGCTATCGCCAGCAACGGTGGCGGCAAGCAAGCGCTCGAAACGGTGCAGCGGCTGTTGCCGGTGCTGTGCCAGGACCATGGCCTGACTCCGGACCAAGTGGTGGCTATCGCCAGCAACGGTGGCGGCAAGCAAGCGCTCGAAACGGTGCAGCGGCTGTTGCCGGTGCTGTGCCAGGACCATGGCCTGACTCCGGACCAAGTGGTGGCTATCGCCAGCAACGGTGGCGGCAAGCAAGCGCTCGAAACGGTGCAGCGGCTGTTGCCGGTGCTGTGCCAGGACCATGGCCTGACCCCGGACCAAGTGGTGGCTATCGCCAGCCACGATGGCGGCAAGCAAGCGCTCGAAACGGTGCAGCGGCTGTTGCCGGTGCTGTGCCAGGACCATGGCCTGACTCCGGACCAAGTGGTGGCTATCGCCAGCAACATTGGCGGCAAGCAAGCGCTCGAAACGGTGCAGCGGCTGTTGCCGGTGCTGTGCCAGGACCATGGCCTGACCCCGGACCAAGTGGTGGCTATCGCCAGCAACATTGGCGGCAAGCAAGCGCTCGAAACGGTGCAGCGGCTGTTGCCGGTGCTGTGCCAGGACCATGGCCTGACCCCGGACCAAGTGGTGGCTATCGCCAGCAACGGTGGCGGCAAGCAAGCGCTCGAAACGGTGCAGCGGCTGTTGCCGGTGCTGTGCCAGGACCATGGCCTGACCCCGGACCAAGTGGTGGCTATCGCCAGCAACGGTGGCGGCAAGCAAGCGCTCGAAACGGTGCAGCGGCTGTTGCCGGTGCTGTGCCAGGACCATGGCCTGACCCCGGACCAAGTGGTGGCTATCGCCAGCAACGGTGGCGGCAAGCAAGCGCTCGAAACGGTGCAGCGGCTGTTGCCGGTGCTGTGCCAGGACCATGGCCTGACCCCGGACCAAGTGGTGGCTATCGCCAGCAACAATGGCGGCAAGCAAGCGCTCGAAACGGTGCAGCGGCTGTTGCCGGTGCTGTGCCAGGACCATGGCCTGACTCCGGACCAAGTGGTGGCTATCGCCAGCCACGATGGCGGCAAGCAAGCGCTCGAAACGGTGCAGCGGCTGTTGCCGGTGCTGTGCCAGGACCATGGCCTGACTCCGGACCAAGTGGTGGCTATCGCCAGCCACGATGGCGGCAAGCAAGCGCTCGAAACGGTGCAGCGGCTGTTGCCGGTGCTGTGCCAGGACCATGGCCTGACCCCGGACCAAGTGGTGGCTATCGCCAGCAACGGTGGCGGCAAGCAAGCGCTCGAAACGGTGCAGCGGCTGTTGCCGGTGCTGTGCCAGGACCATGGCCTGACCCCGGACCAAGTGGTGGCTATCGCCAGCAACGGTGGCGGCAAGCAAGCGCTCGAAACGGTGCAGCGGCTGTTGCCGGTGCTGTGCCAGGACCATGGCCTGACCCCGGACCAAGTGGTGGCTATCGCCAGCAACAATGGCGGCAAGCAAGCGCTCGAAAGCATTGTGGCCCAGCTGAGCCGGCCTGATCCGGCGTTGGCCGCGTTGACCAACGACCACCTCGTCGCCTTGGCCTGCCTCGGCGGACGTCCTGCCATGGATGCAGTGAAAAAGGGATTGCCGCACGCGCCGGAATTGATCAGAAGAGTCAATCGCCGTATTGGCGAACGCACGTCCCATCGCGTTGCCGACTACGCGCAAGTGGTTCGCGTGCTGGAGTTTTTCCAGTGCCACTCCCACCCAGCGTACGCATTTGATGAGGCCATGACGCAGTTCGGGATGAGCAGGAACGGGTTGGTACAGCTCTTTCGCAGAGTGGGCGTCACCGAACTCGAAGCCCGCGGTGGAACGCTCCCCCCAGCCTCGCAGCGTTGGGACCGTATCCTCCAGGCATCAGGGATGAAAAGGGCCAAACCGTCCCCTACTTCAGCTCAAACACCGGATCAGGCGTCTTTGCATGCATTCGCCGATTCGCTGGAGCGTGACCTTGATGCGCCCAGCCCAATGCACGAGGGAGATCAGACGCGGGCAAGCAGCCGTAAACGGTCCCGATCGGATCGTGCTGTCACCGGCCCCTCCGCACAGCAGGCTGTCGAGGTGCGCGTTCCCGAACAGCGCGATGCGCTGCATTTGCCCCTCAGCTGGAGGGTAAAACGCCCGCGTACCAGGATCTGGGGCGGCCTCCCGGATCCTGGTACGCCCATGGCTGCCGACCTGGCAGCGTCCAGCACCGTGATGTGGGAACAAGATGCGGACCCCTTCGCAGGGGCAGCGGATGATTTCCCGGCATTCAACGAAGAGGAACTCGCATGGTTGATGGAGCTATTGCCTCAGAAGGGCGAATTCAAGCTTTACCCATACGATGTTCCAGATTACGCTTGA

>dTALELBM5

CGACCATGTAAAGAGGTATGCCTGATGGATCCCATTCGTTCGCGCACACCAAGTCCTGCCCGCGAGCTTCTGCCCGGACCCCAACCCGATGGGGTTCAGCCGACTGCAGATCGTGGGGTGTCTGCGCCTGCTGGCAGCCCTCTGGATGGCTTGCCCGCTCGGCGGACGGTGTCCCGGACCCGGCTGCCATCTCCCCCTGCGCCCTCACCTGCGTTCTCGGCGGGCAGCTTCAGCGATCTGCTCCGTCCGTTCGATCCGTCGCTTCTTGATACATCGCTTCTTGATTCGATGCCTGCCGTCGGCACGCCGCATACAGCGGCTGCCCCAGCAGAGTGGGATGAGGCGCAATCGGCTCTGCGTGCAGCCGATGACCCGCCACCCACCGTGCGTGTCGCTGTCACTGCCGCGCGGCCGCCGCGCGCCAAGCCGGCCCCGCGACGGCGTGCTGCGCAACCCTCCGACGCTTCGCCGGCCGCGCAGGTGGATCTACGCACGCTCGGCTACAGTCAGCAGCAGCAAGAGAAGATCAAACCGAAGGTGCGTTCGACAGTGGCGCAGCACCACGAGGCACTGGTGGGCCATGGGTTTACACACGCGCACATCGTTGCGCTCAGCCAACACCCGGCAGCGTTAGGGACCGTCGCTGTCACGTATCAGCACATAATCACGGCGTTGCCAGAGGCGACACACGAAGACATCGTTGGCGTCGGCAAACAGTGGTCCGGCGCACGCGCCCTGGAGGCCTTGCTCACGGATGCGGGGGAGTTGAGAGGTCCGCCGTTACAGTTGGACACAGGCCAACTTGTGAAGATTGCAAAACGTGGCGGCGTGACCGCAATGGAGGCAGTGCATGCATCGCGCAATGCACTGACGGGTGCCCCCCTGAACCTGACCCCGGACCAAGTGGTGGCTATCGCCAGCAACATTGGCGGCAAGCAAGCGCTCGAAACGGTGCAGCGGCTGTTGCCGGTGCTGTGCCAGGACCATGGCCTGACCCCGGACCAAGTGGTGGCTATCGCCAGCAACGGTGGCGGCAAGCAAGCGCTCGAAACGGTGCAGCGGCTGTTGCCGGTGCTGTGCCAGGACCATGGCCTGACCCCGGACCAAGTGGTGGCTATCGCCAGCAACATTGGCGGCAAGCAAGCGCTCGAAACGGTGCAGCGGCTGTTGCCGGTGCTGTGCCAGGACCATGGCCTGACCCCGGACCAAGTGGTGGCTATCGCCAGCAACATTGGCGGCAAGCAAGCGCTCGAAACGGTGCAGCGGCTGTTGCCGGTGCTGTGCCAGGACCATGGCCTGACCCCGGACCAAGTGGTGGCTATCGCCAGCAACATTGGCGGCAAGCAAGCGCTCGAAACGGTGCAGCGGCTGTTGCCGGTGCTGTGCCAGGACCATGGCCTGACTCCGGACCAAGTGGTGGCTATCGCCAGCCACGATGGCGGCAAGCAAGCGCTCGAAACGGTGCAGCGGCTGTTGCCGGTGCTGTGCCAGGACCATGGCCTGACTCCGGACCAAGTGGTGGCTATCGCCAGCCACGATGGCGGCAAGCAAGCGCTCGAAACGGTGCAGCGGCTGTTGCCGGTGCTGTGCCAGGACCATGGCCTGACCCCGGACCAAGTGGTGGCTATCGCCAGCAACGGTGGCGGCAAGCAAGCGCTCGAAACGGTGCAGCGGCTGTTGCCGGTGCTGTGCCAGGACCATGGCCTGACTCCGGACCAAGTGGTGGCTATCGCCAGCCACGATGGCGGCAAGCAAGCGCTCGAAACGGTGCAGCGGCTGTTGCCGGTGCTGTGCCAGGACCATGGCCTGACCCCGGACCAAGTGGTGGCTATCGCCAGCAACGGTGGCGGCAAGCAAGCGCTCGAAACGGTGCAGCGGCTGTTGCCGGTGCTGTGCCAGGACCATGGCCTGACCCCGGACCAAGTGGTGGCTATCGCCAGCAACGGTGGCGGCAAGCAAGCGCTCGAAACGGTGCAGCGGCTGTTGCCGGTGCTGTGCCAGGACCATGGCCTGACCCCGGACCAAGTGGTGGCTATCGCCAGCAACATTGGCGGCAAGCAAGCGCTCGAAACGGTGCAGCGGCTGTTGCCGGTGCTGTGCCAGGACCATGGCCTGACCCCGGACCAAGTGGTGGCTATCGCCAGCAACATTGGCGGCAAGCAAGCGCTCGAAACGGTGCAGCGGCTGTTGCCGGTGCTGTGCCAGGACCATGGCCTGACCCCGGACCAAGTGGTGGCTATCGCCAGCAACAATGGCGGCAAGCAAGCGCTCGAAACGGTGCAGCGGCTGTTGCCGGTGCTGTGCCAGGACCATGGCCTGACTCCGGACCAAGTGGTGGCTATCGCCAGCCACGATGGCGGCAAGCAAGCGCTCGAAACGGTGCAGCGGCTGTTGCCGGTGCTGTGCCAGGACCATGGCCTGACTCCGGACCAAGTGGTGGCTATCGCCAGCCACGATGGCGGCAAGCAAGCGCTCGAAACGGTGCAGCGGCTGTTGCCGGTGCTGTGCCAGGACCATGGCCTGACCCCGGACCAAGTGGTGGCTATCGCCAGCAACGGTGGCGGCAAGCAAGCGCTCGAAACGGTGCAGCGGCTGTTGCCGGTGCTGTGCCAGGACCATGGCCTGACCCCGGACCAAGTGGTGGCTATCGCCAGCAACGGTGGCGGCAAGCAAGCGCTCGAAACGGTGCAGCGGCTGTTGCCGGTGCTGTGCCAGGACCATGGCCTGACCCCGGACCAAGTGGTGGCTATCGCCAGCAACAATGGCGGCAAGCAAGCGCTCGAAAGCATTGTGGCCCAGCTGAGCCGGCCTGATCCGGCGTTGGCCGCGTTGACCAACGACCACCTCGTCGCCTTGGCCTGCCTCGGCGGACGTCCTGCCATGGATGCAGTGAAAAAGGGATTGCCGCACGCGCCGGAATTGATCAGAAGAGTCAATCGCCGTATTGGCGAACGCACGTCCCATCGCGTTGCCGACTACGCGCAAGTGGTTCGCGTGCTGGAGTTTTTCCAGTGCCACTCCCACCCAGCGTACGCATTTGATGAGGCCATGACGCAGTTCGGGATGAGCAGGAACGGGTTGGTACAGCTCTTTCGCAGAGTGGGCGTCACCGAACTCGAAGCCCGCGGTGGAACGCTCCCCCCAGCCTCGCAGCGTTGGGACCGTATCCTCCAGGCATCAGGGATGAAAAGGGCCAAACCGTCCCCTACTTCAGCTCAAACACCGGATCAGGCGTCTTTGCATGCATTCGCCGATTCGCTGGAGCGTGACCTTGATGCGCCCAGCCCAATGCACGAGGGAGATCAGACGCGGGCAAGCAGCCGTAAACGGTCCCGATCGGATCGTGCTGTCACCGGCCCCTCCGCACAGCAGGCTGTCGAGGTGCGCGTTCCCGAACAGCGCGATGCGCTGCATTTGCCCCTCAGCTGGAGGGTAAAACGCCCGCGTACCAGGATCTGGGGCGGCCTCCCGGATCCTGGTACGCCCATGGCTGCCGACCTGGCAGCGTCCAGCACCGTGATGTGGGAACAAGATGCGGACCCCTTCGCAGGGGCAGCGGATGATTTCCCGGCATTCAACGAAGAGGAACTCGCATGGTTGATGGAGCTATTGCCTCAGAAGGGCGAATTCAAGCTTTACCCATACGATGTTCCAGATTACGCTTGA

>dTALELBM6

CGACCATGTAAAGAGGTATGCCTGATGGATCCCATTCGTTCGCGCACACCAAGTCCTGCCCGCGAGCTTCTGCCCGGACCCCAACCCGATGGGGTTCAGCCGACTGCAGATCGTGGGGTGTCTGCGCCTGCTGGCAGCCCTCTGGATGGCTTGCCCGCTCGGCGGACGGTGTCCCGGACCCGGCTGCCATCTCCCCCTGCGCCCTCACCTGCGTTCTCGGCGGGCAGCTTCAGCGATCTGCTCCGTCCGTTCGATCCGTCGCTTCTTGATACATCGCTTCTTGATTCGATGCCTGCCGTCGGCACGCCGCATACAGCGGCTGCCCCAGCAGAGTGGGATGAGGCGCAATCGGCTCTGCGTGCAGCCGATGACCCGCCACCCACCGTGCGTGTCGCTGTCACTGCCGCGCGGCCGCCGCGCGCCAAGCCGGCCCCGCGACGGCGTGCTGCGCAACCCTCCGACGCTTCGCCGGCCGCGCAGGTGGATCTACGCACGCTCGGCTACAGTCAGCAGCAGCAAGAGAAGATCAAACCGAAGGTGCGTTCGACAGTGGCGCAGCACCACGAGGCACTGGTGGGCCATGGGTTTACACACGCGCACATCGTTGCGCTCAGCCAACACCCGGCAGCGTTAGGGACCGTCGCTGTCACGTATCAGCACATAATCACGGCGTTGCCAGAGGCGACACACGAAGACATCGTTGGCGTCGGCAAACAGTGGTCCGGCGCACGCGCCCTGGAGGCCTTGCTCACGGATGCGGGGGAGTTGAGAGGTCCGCCGTTACAGTTGGACACAGGCCAACTTGTGAAGATTGCAAAACGTGGCGGCGTGACCGCAATGGAGGCAGTGCATGCATCGCGCAATGCACTGACGGGTGCCCCCCTGAACCTGACCCCGGACCAAGTGGTGGCTATCGCCAGCCACGATGGCGGCAAGCAAGCGCTCGAAACGGTGCAGCGGCTGTTGCCGGTGCTGTGCCAGGACCATGGCCTGACCCCGGACCAAGTGGTGGCTATCGCCAGCCACGATGGCGGCAAGCAAGCGCTCGAAACGGTGCAGCGGCTGTTGCCGGTGCTGTGCCAGGACCATGGCCTGACCCCGGACCAAGTGGTGGCTATCGCCAGCAACGGTGGCGGCAAGCAAGCGCTCGAAACGGTGCAGCGGCTGTTGCCGGTGCTGTGCCAGGACCATGGCCTGACCCCGGACCAAGTGGTGGCTATCGCCAGCAACGGTGGCGGCAAGCAAGCGCTCGAAACGGTGCAGCGGCTGTTGCCGGTGCTGTGCCAGGACCATGGCCTGACCCCGGACCAAGTGGTGGCTATCGCCAGCAACGGTGGCGGCAAGCAAGCGCTCGAAACGGTGCAGCGGCTGTTGCCGGTGCTGTGCCAGGACCATGGCCTGACTCCGGACCAAGTGGTGGCTATCGCCAGCCACGATGGCGGCAAGCAAGCGCTCGAAACGGTGCAGCGGCTGTTGCCGGTGCTGTGCCAGGACCATGGCCTGACTCCGGACCAAGTGGTGGCTATCGCCAGCAACATTGGCGGCAAGCAAGCGCTCGAAACGGTGCAGCGGCTGTTGCCGGTGCTGTGCCAGGACCATGGCCTGACCCCGGACCAAGTGGTGGCTATCGCCAGCAACATTGGCGGCAAGCAAGCGCTCGAAACGGTGCAGCGGCTGTTGCCGGTGCTGTGCCAGGACCATGGCCTGACTCCGGACCAAGTGGTGGCTATCGCCAGCAACGGTGGCGGCAAGCAAGCGCTCGAAACGGTGCAGCGGCTGTTGCCGGTGCTGTGCCAGGACCATGGCCTGACCCCGGACCAAGTGGTGGCTATCGCCAGCCACGATGGCGGCAAGCAAGCGCTCGAAACGGTGCAGCGGCTGTTGCCGGTGCTGTGCCAGGACCATGGCCTGACCCCGGACCAAGTGGTGGCTATCGCCAGCAACGGTGGCGGCAAGCAAGCGCTCGAAACGGTGCAGCGGCTGTTGCCGGTGCTGTGCCAGGACCATGGCCTGACCCCGGACCAAGTGGTGGCTATCGCCAGCAACGGTGGCGGCAAGCAAGCGCTCGAAACGGTGCAGCGGCTGTTGCCGGTGCTGTGCCAGGACCATGGCCTGACCCCGGACCAAGTGGTGGCTATCGCCAGCAACGGTGGCGGCAAGCAAGCGCTCGAAACGGTGCAGCGGCTGTTGCCGGTGCTGTGCCAGGACCATGGCCTGACCCCGGACCAAGTGGTGGCTATCGCCAGCAACAATGGCGGCAAGCAAGCGCTCGAAACGGTGCAGCGGCTGTTGCCGGTGCTGTGCCAGGACCATGGCCTGACTCCGGACCAAGTGGTGGCTATCGCCAGCCACGATGGCGGCAAGCAAGCGCTCGAAACGGTGCAGCGGCTGTTGCCGGTGCTGTGCCAGGACCATGGCCTGACTCCGGACCAAGTGGTGGCTATCGCCAGCCACGATGGCGGCAAGCAAGCGCTCGAAACGGTGCAGCGGCTGTTGCCGGTGCTGTGCCAGGACCATGGCCTGACCCCGGACCAAGTGGTGGCTATCGCCAGCAACGGTGGCGGCAAGCAAGCGCTCGAAACGGTGCAGCGGCTGTTGCCGGTGCTGTGCCAGGACCATGGCCTGACCCCGGACCAAGTGGTGGCTATCGCCAGCAACGGTGGCGGCAAGCAAGCGCTCGAAACGGTGCAGCGGCTGTTGCCGGTGCTGTGCCAGGACCATGGCCTGACCCCGGACCAAGTGGTGGCTATCGCCAGCAACAATGGCGGCAAGCAAGCGCTCGAAAGCATTGTGGCCCAGCTGAGCCGGCCTGATCCGGCGTTGGCCGCGTTGACCAACGACCACCTCGTCGCCTTGGCCTGCCTCGGCGGACGTCCTGCCATGGATGCAGTGAAAAAGGGATTGCCGCACGCGCCGGAATTGATCAGAAGAGTCAATCGCCGTATTGGCGAACGCACGTCCCATCGCGTTGCCGACTACGCGCAAGTGGTTCGCGTGCTGGAGTTTTTCCAGTGCCACTCCCACCCAGCGTACGCATTTGATGAGGCCATGACGCAGTTCGGGATGAGCAGGAACGGGTTGGTACAGCTCTTTCGCAGAGTGGGCGTCACCGAACTCGAAGCCCGCGGTGGAACGCTCCCCCCAGCCTCGCAGCGTTGGGACCGTATCCTCCAGGCATCAGGGATGAAAAGGGCCAAACCGTCCCCTACTTCAGCTCAAACACCGGATCAGGCGTCTTTGCATGCATTCGCCGATTCGCTGGAGCGTGACCTTGATGCGCCCAGCCCAATGCACGAGGGAGATCAGACGCGGGCAAGCAGCCGTAAACGGTCCCGATCGGATCGTGCTGTCACCGGCCCCTCCGCACAGCAGGCTGTCGAGGTGCGCGTTCCCGAACAGCGCGATGCGCTGCATTTGCCCCTCAGCTGGAGGGTAAAACGCCCGCGTACCAGGATCTGGGGCGGCCTCCCGGATCCTGGTACGCCCATGGCTGCCGACCTGGCAGCGTCCAGCACCGTGATGTGGGAACAAGATGCGGACCCCTTCGCAGGGGCAGCGGATGATTTCCCGGCATTCAACGAAGAGGAACTCGCATGGTTGATGGAGCTATTGCCTCAGAAGGGCGAATTCAAGCTTTACCCATACGATGTTCCAGATTACGCTTGA

>dTALELBM7

CGACCATGTAAAGAGGTATGCCTGATGGATCCCATTCGTTCGCGCACACCAAGTCCTGCCCGCGAGCTTCTGCCCGGACCCCAACCCGATGGGGTTCAGCCGACTGCAGATCGTGGGGTGTCTGCGCCTGCTGGCAGCCCTCTGGATGGCTTGCCCGCTCGGCGGACGGTGTCCCGGACCCGGCTGCCATCTCCCCCTGCGCCCTCACCTGCGTTCTCGGCGGGCAGCTTCAGCGATCTGCTCCGTCCGTTCGATCCGTCGCTTCTTGATACATCGCTTCTTGATTCGATGCCTGCCGTCGGCACGCCGCATACAGCGGCTGCCCCAGCAGAGTGGGATGAGGCGCAATCGGCTCTGCGTGCAGCCGATGACCCGCCACCCACCGTGCGTGTCGCTGTCACTGCCGCGCGGCCGCCGCGCGCCAAGCCGGCCCCGCGACGGCGTGCTGCGCAACCCTCCGACGCTTCGCCGGCCGCGCAGGTGGATCTACGCACGCTCGGCTACAGTCAGCAGCAGCAAGAGAAGATCAAACCGAAGGTGCGTTCGACAGTGGCGCAGCACCACGAGGCACTGGTGGGCCATGGGTTTACACACGCGCACATCGTTGCGCTCAGCCAACACCCGGCAGCGTTAGGGACCGTCGCTGTCACGTATCAGCACATAATCACGGCGTTGCCAGAGGCGACACACGAAGACATCGTTGGCGTCGGCAAACAGTGGTCCGGCGCACGCGCCCTGGAGGCCTTGCTCACGGATGCGGGGGAGTTGAGAGGTCCGCCGTTACAGTTGGACACAGGCCAACTTGTGAAGATTGCAAAACGTGGCGGCGTGACCGCAATGGAGGCAGTGCATGCATCGCGCAATGCACTGACGGGTGCCCCCCTGAACCTGACCCCGGACCAAGTGGTGGCTATCGCCAGCAACATTGGCGGCAAGCAAGCGCTCGAAACGGTGCAGCGGCTGTTGCCGGTGCTGTGCCAGGACCATGGCCTGACCCCGGACCAAGTGGTGGCTATCGCCAGCAACGGTGGCGGCAAGCAAGCGCTCGAAACGGTGCAGCGGCTGTTGCCGGTGCTGTGCCAGGACCATGGCCTGACCCCGGACCAAGTGGTGGCTATCGCCAGCAACATTGGCGGCAAGCAAGCGCTCGAAACGGTGCAGCGGCTGTTGCCGGTGCTGTGCCAGGACCATGGCCTGACCCCGGACCAAGTGGTGGCTATCGCCAGCAACGGTGGCGGCAAGCAAGCGCTCGAAACGGTGCAGCGGCTGTTGCCGGTGCTGTGCCAGGACCATGGCCTGACCCCGGACCAAGTGGTGGCTATCGCCAGCAACATTGGCGGCAAGCAAGCGCTCGAAACGGTGCAGCGGCTGTTGCCGGTGCTGTGCCAGGACCATGGCCTGACTCCGGACCAAGTGGTGGCTATCGCCAGCAACATTGGCGGCAAGCAAGCGCTCGAAACGGTGCAGCGGCTGTTGCCGGTGCTGTGCCAGGACCATGGCCTGACTCCGGACCAAGTGGTGGCTATCGCCAGCAACATTGGCGGCAAGCAAGCGCTCGAAACGGTGCAGCGGCTGTTGCCGGTGCTGTGCCAGGACCATGGCCTGACCCCGGACCAAGTGGTGGCTATCGCCAGCAACATTGGCGGCAAGCAAGCGCTCGAAACGGTGCAGCGGCTGTTGCCGGTGCTGTGCCAGGACCATGGCCTGACTCCGGACCAAGTGGTGGCTATCGCCAGCCACGATGGCGGCAAGCAAGCGCTCGAAACGGTGCAGCGGCTGTTGCCGGTGCTGTGCCAGGACCATGGCCTGACCCCGGACCAAGTGGTGGCTATCGCCAGCCACGATGGCGGCAAGCAAGCGCTCGAAACGGTGCAGCGGCTGTTGCCGGTGCTGTGCCAGGACCATGGCCTGACCCCGGACCAAGTGGTGGCTATCGCCAGCAACGGTGGCGGCAAGCAAGCGCTCGAAACGGTGCAGCGGCTGTTGCCGGTGCTGTGCCAGGACCATGGCCTGACCCCGGACCAAGTGGTGGCTATCGCCAGCAACGGTGGCGGCAAGCAAGCGCTCGAAACGGTGCAGCGGCTGTTGCCGGTGCTGTGCCAGGACCATGGCCTGACCCCGGACCAAGTGGTGGCTATCGCCAGCAACGGTGGCGGCAAGCAAGCGCTCGAAACGGTGCAGCGGCTGTTGCCGGTGCTGTGCCAGGACCATGGCCTGACCCCGGACCAAGTGGTGGCTATCGCCAGCAACAATGGCGGCAAGCAAGCGCTCGAAACGGTGCAGCGGCTGTTGCCGGTGCTGTGCCAGGACCATGGCCTGACTCCGGACCAAGTGGTGGCTATCGCCAGCCACGATGGCGGCAAGCAAGCGCTCGAAACGGTGCAGCGGCTGTTGCCGGTGCTGTGCCAGGACCATGGCCTGACTCCGGACCAAGTGGTGGCTATCGCCAGCCACGATGGCGGCAAGCAAGCGCTCGAAACGGTGCAGCGGCTGTTGCCGGTGCTGTGCCAGGACCATGGCCTGACCCCGGACCAAGTGGTGGCTATCGCCAGCAACGGTGGCGGCAAGCAAGCGCTCGAAACGGTGCAGCGGCTGTTGCCGGTGCTGTGCCAGGACCATGGCCTGACCCCGGACCAAGTGGTGGCTATCGCCAGCAACGGTGGCGGCAAGCAAGCGCTCGAAACGGTGCAGCGGCTGTTGCCGGTGCTGTGCCAGGACCATGGCCTGACCCCGGACCAAGTGGTGGCTATCGCCAGCAACAATGGCGGCAAGCAAGCGCTCGAAAGCATTGTGGCCCAGCTGAGCCGGCCTGATCCGGCGTTGGCCGCGTTGACCAACGACCACCTCGTCGCCTTGGCCTGCCTCGGCGGACGTCCTGCCATGGATGCAGTGAAAAAGGGATTGCCGCACGCGCCGGAATTGATCAGAAGAGTCAATCGCCGTATTGGCGAACGCACGTCCCATCGCGTTGCCGACTACGCGCAAGTGGTTCGCGTGCTGGAGTTTTTCCAGTGCCACTCCCACCCAGCGTACGCATTTGATGAGGCCATGACGCAGTTCGGGATGAGCAGGAACGGGTTGGTACAGCTCTTTCGCAGAGTGGGCGTCACCGAACTCGAAGCCCGCGGTGGAACGCTCCCCCCAGCCTCGCAGCGTTGGGACCGTATCCTCCAGGCATCAGGGATGAAAAGGGCCAAACCGTCCCCTACTTCAGCTCAAACACCGGATCAGGCGTCTTTGCATGCATTCGCCGATTCGCTGGAGCGTGACCTTGATGCGCCCAGCCCAATGCACGAGGGAGATCAGACGCGGGCAAGCAGCCGTAAACGGTCCCGATCGGATCGTGCTGTCACCGGCCCCTCCGCACAGCAGGCTGTCGAGGTGCGCGTTCCCGAACAGCGCGATGCGCTGCATTTGCCCCTCAGCTGGAGGGTAAAACGCCCGCGTACCAGGATCTGGGGCGGCCTCCCGGATCCTGGTACGCCCATGGCTGCCGACCTGGCAGCGTCCAGCACCGTGATGTGGGAACAAGATGCGGACCCCTTCGCAGGGGCAGCGGATGATTTCCCGGCATTCAACGAAGAGGAACTCGCATGGTTGATGGAGCTATTGCCTCAGAAGGGCGAATTCAAGCTTTACCCATACGATGTTCCAGATTACGCTTGA

>dTALELB2A1

CGACCATGTAAAGAGGTATGCCTGATGGATCCCATTCGTTCGCGCACACCAAGTCCTGCCCGCGAGCTTCTGCCCGGACCCCAACCCGATGGGGTTCAGCCGACTGCAGATCGTGGGGTGTCTGCGCCTGCTGGCAGCCCTCTGGATGGCTTGCCCGCTCGGCGGACGGTGTCCCGGACCCGGCTGCCATCTCCCCCTGCGCCCTCACCTGCGTTCTCGGCGGGCAGCTTCAGCGATCTGCTCCGTCCGTTCGATCCGTCGCTTCTTGATACATCGCTTCTTGATTCGATGCCTGCCGTCGGCACGCCGCATACAGCGGCTGCCCCAGCAGAGTGGGATGAGGCGCAATCGGCTCTGCGTGCAGCCGATGACCCGCCACCCACCGTGCGTGTCGCTGTCACTGCCGCGCGGCCGCCGCGCGCCAAGCCGGCCCCGCGACGGCGTGCTGCGCAACCCTCCGACGCTTCGCCGGCCGCGCAGGTGGATCTACGCACGCTCGGCTACAGTCAGCAGCAGCAAGAGAAGATCAAACCGAAGGTGCGTTCGACAGTGGCGCAGCACCACGAGGCACTGGTGGGCCATGGGTTTACACACGCGCACATCGTTGCGCTCAGCCAACACCCGGCAGCGTTAGGGACCGTCGCTGTCACGTATCAGCACATAATCACGGCGTTGCCAGAGGCGACACACGAAGACATCGTTGGCGTCGGCAAACAGTGGTCCGGCGCACGCGCCCTGGAGGCCTTGCTCACGGATGCGGGGGAGTTGAGAGGTCCGCCGTTACAGTTGGACACAGGCCAACTTGTGAAGATTGCAAAACGTGGCGGCGTGACCGCAATGGAGGCAGTGCATGCATCGCGCAATGCACTGACGGGTGCCCCCCTGAACCTGACCCCGGACCAAGTGGTGGCTATCGCCAGCAACGGTGGCGGCAAGCAAGCGCTCGAAACGGTGCAGCGGCTGTTGCCGGTGCTGTGCCAGGACCATGGCCTGACCCCGGACCAAGTGGTGGCTATCGCCAGCAACGGTGGCGGCAAGCAAGCGCTCGAAACGGTGCAGCGGCTGTTGCCGGTGCTGTGCCAGGACCATGGCCTGACCCCGGACCAAGTGGTGGCTATCGCCAGCAACATTGGCGGCAAGCAAGCGCTCGAAACGGTGCAGCGGCTGTTGCCGGTGCTGTGCCAGGACCATGGCCTGACCCCGGACCAAGTGGTGGCTATCGCCAGCAACATTGGCGGCAAGCAAGCGCTCGAAACGGTGCAGCGGCTGTTGCCGGTGCTGTGCCAGGACCATGGCCTGACCCCGGACCAAGTGGTGGCTATCGCCAGCAACATTGGCGGCAAGCAAGCGCTCGAAACGGTGCAGCGGCTGTTGCCGGTGCTGTGCCAGGACCATGGCCTGACTCCGGACCAAGTGGTGGCTATCGCCAGCCACGATGGCGGCAAGCAAGCGCTCGAAACGGTGCAGCGGCTGTTGCCGGTGCTGTGCCAGGACCATGGCCTGACTCCGGACCAAGTGGTGGCTATCGCCAGCCACGATGGCGGCAAGCAAGCGCTCGAAACGGTGCAGCGGCTGTTGCCGGTGCTGTGCCAGGACCATGGCCTGACCCCGGACCAAGTGGTGGCTATCGCCAGCAACGGTGGCGGCAAGCAAGCGCTCGAAACGGTGCAGCGGCTGTTGCCGGTGCTGTGCCAGGACCATGGCCTGACTCCGGACCAAGTGGTGGCTATCGCCAGCCACGATGGCGGCAAGCAAGCGCTCGAAACGGTGCAGCGGCTGTTGCCGGTGCTGTGCCAGGACCATGGCCTGACCCCGGACCAAGTGGTGGCTATCGCCAGCAACGGTGGCGGCAAGCAAGCGCTCGAAACGGTGCAGCGGCTGTTGCCGGTGCTGTGCCAGGACCATGGCCTGACCCCGGACCAAGTGGTGGCTATCGCCAGCAACGGTGGCGGCAAGCAAGCGCTCGAAACGGTGCAGCGGCTGTTGCCGGTGCTGTGCCAGGACCATGGCCTGACCCCGGACCAAGTGGTGGCTATCGCCAGCAACGGTGGCGGCAAGCAAGCGCTCGAAACGGTGCAGCGGCTGTTGCCGGTGCTGTGCCAGGACCATGGCCTGACCCCGGACCAAGTGGTGGCTATCGCCAGCAACGGTGGCGGCAAGCAAGCGCTCGAAACGGTGCAGCGGCTGTTGCCGGTGCTGTGCCAGGACCATGGCCTGACCCCGGACCAAGTGGTGGCTATCGCCAGCAACAATGGCGGCAAGCAAGCGCTCGAAACGGTGCAGCGGCTGTTGCCGGTGCTGTGCCAGGACCATGGCCTGACTCCGGACCAAGTGGTGGCTATCGCCAGCCACGATGGCGGCAAGCAAGCGCTCGAAACGGTGCAGCGGCTGTTGCCGGTGCTGTGCCAGGACCATGGCCTGACTCCGGACCAAGTGGTGGCTATCGCCAGCCACGATGGCGGCAAGCAAGCGCTCGAAACGGTGCAGCGGCTGTTGCCGGTGCTGTGCCAGGACCATGGCCTGACCCCGGACCAAGTGGTGGCTATCGCCAGCAACGGTGGCGGCAAGCAAGCGCTCGAAACGGTGCAGCGGCTGTTGCCGGTGCTGTGCCAGGACCATGGCCTGACCCCGGACCAAGTGGTGGCTATCGCCAGCAACGGTGGCGGCAAGCAAGCGCTCGAAACGGTGCAGCGGCTGTTGCCGGTGCTGTGCCAGGACCATGGCCTGACCCCGGACCAAGTGGTGGCTATCGCCAGCAACAATGGCGGCAAGCAAGCGCTCGAAAGCATTGTGGCCCAGCTGAGCCGGCCTGATCCGGCGTTGGCCGCGTTGACCAACGACCACCTCGTCGCCTTGGCCTGCCTCGGCGGACGTCCTGCCATGGATGCAGTGAAAAAGGGATTGCCGCACGCGCCGGAATTGATCAGAAGAGTCAATCGCCGTATTGGCGAACGCACGTCCCATCGCGTTGCCGACTACGCGCAAGTGGTTCGCGTGCTGGAGTTTTTCCAGTGCCACTCCCACCCAGCGTACGCATTTGATGAGGCCATGACGCAGTTCGGGATGAGCAGGAACGGGTTGGTACAGCTCTTTCGCAGAGTGGGCGTCACCGAACTCGAAGCCCGCGGTGGAACGCTCCCCCCAGCCTCGCAGCGTTGGGACCGTATCCTCCAGGCATCAGGGATGAAAAGGGCCAAACCGTCCCCTACTTCAGCTCAAACACCGGATCAGGCGTCTTTGCATGCATTCGCCGATTCGCTGGAGCGTGACCTTGATGCGCCCAGCCCAATGCACGAGGGAGATCAGACGCGGGCAAGCAGCCGTAAACGGTCCCGATCGGATCGTGCTGTCACCGGCCCCTCCGCACAGCAGGCTGTCGAGGTGCGCGTTCCCGAACAGCGCGATGCGCTGCATTTGCCCCTCAGCTGGAGGGTAAAACGCCCGCGTACCAGGATCTGGGGCGGCCTCCCGGATCCTGGTACGCCCATGGCTGCCGACCTGGCAGCGTCCAGCACCGTGATGTGGGAACAAGATGCGGACCCCTTCGCAGGGGCAGCGGATGATTTCCCGGCATTCAACGAAGAGGAACTCGCATGGTTGATGGAGCTATTGCCTCAGAAGGGCGAATTCAAGCTTTACCCATACGATGTTCCAGATTACGCTTGA

>dTALELB2A2

CGACCATGTAAAGAGGTATGCCTGATGGATCCCATTCGTTCGCGCACACCAAGTCCTGCCCGCGAGCTTCTGCCCGGACCCCAACCCGATGGGGTTCAGCCGACTGCAGATCGTGGGGTGTCTGCGCCTGCTGGCAGCCCTCTGGATGGCTTGCCCGCTCGGCGGACGGTGTCCCGGACCCGGCTGCCATCTCCCCCTGCGCCCTCACCTGCGTTCTCGGCGGGCAGCTTCAGCGATCTGCTCCGTCCGTTCGATCCGTCGCTTCTTGATACATCGCTTCTTGATTCGATGCCTGCCGTCGGCACGCCGCATACAGCGGCTGCCCCAGCAGAGTGGGATGAGGCGCAATCGGCTCTGCGTGCAGCCGATGACCCGCCACCCACCGTGCGTGTCGCTGTCACTGCCGCGCGGCCGCCGCGCGCCAAGCCGGCCCCGCGACGGCGTGCTGCGCAACCCTCCGACGCTTCGCCGGCCGCGCAGGTGGATCTACGCACGCTCGGCTACAGTCAGCAGCAGCAAGAGAAGATCAAACCGAAGGTGCGTTCGACAGTGGCGCAGCACCACGAGGCACTGGTGGGCCATGGGTTTACACACGCGCACATCGTTGCGCTCAGCCAACACCCGGCAGCGTTAGGGACCGTCGCTGTCACGTATCAGCACATAATCACGGCGTTGCCAGAGGCGACACACGAAGACATCGTTGGCGTCGGCAAACAGTGGTCCGGCGCACGCGCCCTGGAGGCCTTGCTCACGGATGCGGGGGAGTTGAGAGGTCCGCCGTTACAGTTGGACACAGGCCAACTTGTGAAGATTGCAAAACGTGGCGGCGTGACCGCAATGGAGGCAGTGCATGCATCGCGCAATGCACTGACGGGTGCCCCCCTGAACCTGACCCCGGACCAAGTGGTGGCTATCGCCAGCAACATTGGCGGCAAGCAAGCGCTCGAAACGGTGCAGCGGCTGTTGCCGGTGCTGTGCCAGGACCATGGCCTGACCCCGGACCAAGTGGTGGCTATCGCCAGCAACGGTGGCGGCAAGCAAGCGCTCGAAACGGTGCAGCGGCTGTTGCCGGTGCTGTGCCAGGACCATGGCCTGACCCCGGACCAAGTGGTGGCTATCGCCAGCAACATTGGCGGCAAGCAAGCGCTCGAAACGGTGCAGCGGCTGTTGCCGGTGCTGTGCCAGGACCATGGCCTGACCCCGGACCAAGTGGTGGCTATCGCCAGCAACATTGGCGGCAAGCAAGCGCTCGAAACGGTGCAGCGGCTGTTGCCGGTGCTGTGCCAGGACCATGGCCTGACCCCGGACCAAGTGGTGGCTATCGCCAGCAACATTGGCGGCAAGCAAGCGCTCGAAACGGTGCAGCGGCTGTTGCCGGTGCTGTGCCAGGACCATGGCCTGACTCCGGACCAAGTGGTGGCTATCGCCAGCCACGATGGCGGCAAGCAAGCGCTCGAAACGGTGCAGCGGCTGTTGCCGGTGCTGTGCCAGGACCATGGCCTGACTCCGGACCAAGTGGTGGCTATCGCCAGCCACGATGGCGGCAAGCAAGCGCTCGAAACGGTGCAGCGGCTGTTGCCGGTGCTGTGCCAGGACCATGGCCTGACCCCGGACCAAGTGGTGGCTATCGCCAGCAACGGTGGCGGCAAGCAAGCGCTCGAAACGGTGCAGCGGCTGTTGCCGGTGCTGTGCCAGGACCATGGCCTGACTCCGGACCAAGTGGTGGCTATCGCCAGCCACGATGGCGGCAAGCAAGCGCTCGAAACGGTGCAGCGGCTGTTGCCGGTGCTGTGCCAGGACCATGGCCTGACCCCGGACCAAGTGGTGGCTATCGCCAGCAACGGTGGCGGCAAGCAAGCGCTCGAAACGGTGCAGCGGCTGTTGCCGGTGCTGTGCCAGGACCATGGCCTGACCCCGGACCAAGTGGTGGCTATCGCCAGCAACGGTGGCGGCAAGCAAGCGCTCGAAACGGTGCAGCGGCTGTTGCCGGTGCTGTGCCAGGACCATGGCCTGACCCCGGACCAAGTGGTGGCTATCGCCAGCAACGGTGGCGGCAAGCAAGCGCTCGAAACGGTGCAGCGGCTGTTGCCGGTGCTGTGCCAGGACCATGGCCTGACCCCGGACCAAGTGGTGGCTATCGCCAGCAACGGTGGCGGCAAGCAAGCGCTCGAAACGGTGCAGCGGCTGTTGCCGGTGCTGTGCCAGGACCATGGCCTGACCCCGGACCAAGTGGTGGCTATCGCCAGCAACAATGGCGGCAAGCAAGCGCTCGAAAGCATTGTGGCCCAGCTGAGCCGGCCTGATCCGGCGTTGGCCGCGTTGACCAACGACCACCTCGTCGCCTTGGCCTGCCTCGGCGGACGTCCTGCCATGGATGCAGTGAAAAAGGGATTGCCGCACGCGCCGGAATTGATCAGAAGAGTCAATCGCCGTATTGGCGAACGCACGTCCCATCGCGTTGCCGACTACGCGCAAGTGGTTCGCGTGCTGGAGTTTTTCCAGTGCCACTCCCACCCAGCGTACGCATTTGATGAGGCCATGACGCAGTTCGGGATGAGCAGGAACGGGTTGGTACAGCTCTTTCGCAGAGTGGGCGTCACCGAACTCGAAGCCCGCGGTGGAACGCTCCCCCCAGCCTCGCAGCGTTGGGACCGTATCCTCCAGGCATCAGGGATGAAAAGGGCCAAACCGTCCCCTACTTCAGCTCAAACACCGGATCAGGCGTCTTTGCATGCATTCGCCGATTCGCTGGAGCGTGACCTTGATGCGCCCAGCCCAATGCACGAGGGAGATCAGACGCGGGCAAGCAGCCGTAAACGGTCCCGATCGGATCGTGCTGTCACCGGCCCCTCCGCACAGCAGGCTGTCGAGGTGCGCGTTCCCGAACAGCGCGATGCGCTGCATTTGCCCCTCAGCTGGAGGGTAAAACGCCCGCGTACCAGGATCTGGGGCGGCCTCCCGGATCCTGGTACGCCCATGGCTGCCGACCTGGCAGCGTCCAGCACCGTGATGTGGGAACAAGATGCGGACCCCTTCGCAGGGGCAGCGGATGATTTCCCGGCATTCAACGAAGAGGAACTCGCATGGTTGATGGAGCTATTGCCTCAGAAGGGCGAATTCAAGCTTTACCCATACGATGTTCCAGATTACGCTTGA

>dTALELB3A

CGACCATGTAAAGAGGTATGCCTGATGGATCCCATTCGTTCGCGCACACCAAGTCCTGCCCGCGAGCTTCTGCCCGGACCCCAACCCGATGGGGTTCAGCCGACTGCAGATCGTGGGGTGTCTGCGCCTGCTGGCAGCCCTCTGGATGGCTTGCCCGCTCGGCGGACGGTGTCCCGGACCCGGCTGCCATCTCCCCCTGCGCCCTCACCTGCGTTCTCGGCGGGCAGCTTCAGCGATCTGCTCCGTCCGTTCGATCCGTCGCTTCTTGATACATCGCTTCTTGATTCGATGCCTGCCGTCGGCACGCCGCATACAGCGGCTGCCCCAGCAGAGTGGGATGAGGCGCAATCGGCTCTGCGTGCAGCCGATGACCCGCCACCCACCGTGCGTGTCGCTGTCACTGCCGCGCGGCCGCCGCGCGCCAAGCCGGCCCCGCGACGGCGTGCTGCGCAACCCTCCGACGCTTCGCCGGCCGCGCAGGTGGATCTACGCACGCTCGGCTACAGTCAGCAGCAGCAAGAGAAGATCAAACCGAAGGTGCGTTCGACAGTGGCGCAGCACCACGAGGCACTGGTGGGCCATGGGTTTACACACGCGCACATCGTTGCGCTCAGCCAACACCCGGCAGCGTTAGGGACCGTCGCTGTCACGTATCAGCACATAATCACGGCGTTGCCAGAGGCGACACACGAAGACATCGTTGGCGTCGGCAAACAGTGGTCCGGCGCACGCGCCCTGGAGGCCTTGCTCACGGATGCGGGGGAGTTGAGAGGTCCGCCGTTACAGTTGGACACAGGCCAACTTGTGAAGATTGCAAAACGTGGCGGCGTGACCGCAATGGAGGCAGTGCATGCATCGCGCAATGCACTGACGGGTGCCCCCCTGAACCTGACCCCGGACCAAGTGGTGGCTATCGCCAGCAACGGTGGCGGCAAGCAAGCGCTCGAAACGGTGCAGCGGCTGTTGCCGGTGCTGTGCCAGGACCATGGCCTGACCCCGGACCAAGTGGTGGCTATCGCCAGCAACATTGGCGGCAAGCAAGCGCTCGAAACGGTGCAGCGGCTGTTGCCGGTGCTGTGCCAGGACCATGGCCTGACCCCGGACCAAGTGGTGGCTATCGCCAGCAACATTGGCGGCAAGCAAGCGCTCGAAACGGTGCAGCGGCTGTTGCCGGTGCTGTGCCAGGACCATGGCCTGACTCCGGACCAAGTGGTGGCTATCGCCAGCCACGATGGCGGCAAGCAAGCGCTCGAAACGGTGCAGCGGCTGTTGCCGGTGCTGTGCCAGGACCATGGCCTGACTCCGGACCAAGTGGTGGCTATCGCCAGCCACGATGGCGGCAAGCAAGCGCTCGAAACGGTGCAGCGGCTGTTGCCGGTGCTGTGCCAGGACCATGGCCTGACCCCGGACCAAGTGGTGGCTATCGCCAGCAACGGTGGCGGCAAGCAAGCGCTCGAAACGGTGCAGCGGCTGTTGCCGGTGCTGTGCCAGGACCATGGCCTGACTCCGGACCAAGTGGTGGCTATCGCCAGCCACGATGGCGGCAAGCAAGCGCTCGAAACGGTGCAGCGGCTGTTGCCGGTGCTGTGCCAGGACCATGGCCTGACCCCGGACCAAGTGGTGGCTATCGCCAGCAACGGTGGCGGCAAGCAAGCGCTCGAAACGGTGCAGCGGCTGTTGCCGGTGCTGTGCCAGGACCATGGCCTGACCCCGGACCAAGTGGTGGCTATCGCCAGCAACGGTGGCGGCAAGCAAGCGCTCGAAACGGTGCAGCGGCTGTTGCCGGTGCTGTGCCAGGACCATGGCCTGACCCCGGACCAAGTGGTGGCTATCGCCAGCAACGGTGGCGGCAAGCAAGCGCTCGAAACGGTGCAGCGGCTGTTGCCGGTGCTGTGCCAGGACCATGGCCTGACCCCGGACCAAGTGGTGGCTATCGCCAGCAACGGTGGCGGCAAGCAAGCGCTCGAAACGGTGCAGCGGCTGTTGCCGGTGCTGTGCCAGGACCATGGCCTGACCCCGGACCAAGTGGTGGCTATCGCCAGCAACAATGGCGGCAAGCAAGCGCTCGAAACGGTGCAGCGGCTGTTGCCGGTGCTGTGCCAGGACCATGGCCTGACTCCGGACCAAGTGGTGGCTATCGCCAGCCACGATGGCGGCAAGCAAGCGCTCGAAACGGTGCAGCGGCTGTTGCCGGTGCTGTGCCAGGACCATGGCCTGACTCCGGACCAAGTGGTGGCTATCGCCAGCCACGATGGCGGCAAGCAAGCGCTCGAAACGGTGCAGCGGCTGTTGCCGGTGCTGTGCCAGGACCATGGCCTGACCCCGGACCAAGTGGTGGCTATCGCCAGCAACGGTGGCGGCAAGCAAGCGCTCGAAACGGTGCAGCGGCTGTTGCCGGTGCTGTGCCAGGACCATGGCCTGACCCCGGACCAAGTGGTGGCTATCGCCAGCAACGGTGGCGGCAAGCAAGCGCTCGAAACGGTGCAGCGGCTGTTGCCGGTGCTGTGCCAGGACCATGGCCTGACCCCGGACCAAGTGGTGGCTATCGCCAGCAACAATGGCGGCAAGCAAGCGCTCGAAAGCATTGTGGCCCAGCTGAGCCGGCCTGATCCGGCGTTGGCCGCGTTGACCAACGACCACCTCGTCGCCTTGGCCTGCCTCGGCGGACGTCCTGCCATGGATGCAGTGAAAAAGGGATTGCCGCACGCGCCGGAATTGATCAGAAGAGTCAATCGCCGTATTGGCGAACGCACGTCCCATCGCGTTGCCGACTACGCGCAAGTGGTTCGCGTGCTGGAGTTTTTCCAGTGCCACTCCCACCCAGCGTACGCATTTGATGAGGCCATGACGCAGTTCGGGATGAGCAGGAACGGGTTGGTACAGCTCTTTCGCAGAGTGGGCGTCACCGAACTCGAAGCCCGCGGTGGAACGCTCCCCCCAGCCTCGCAGCGTTGGGACCGTATCCTCCAGGCATCAGGGATGAAAAGGGCCAAACCGTCCCCTACTTCAGCTCAAACACCGGATCAGGCGTCTTTGCATGCATTCGCCGATTCGCTGGAGCGTGACCTTGATGCGCCCAGCCCAATGCACGAGGGAGATCAGACGCGGGCAAGCAGCCGTAAACGGTCCCGATCGGATCGTGCTGTCACCGGCCCCTCCGCACAGCAGGCTGTCGAGGTGCGCGTTCCCGAACAGCGCGATGCGCTGCATTTGCCCCTCAGCTGGAGGGTAAAACGCCCGCGTACCAGGATCTGGGGCGGCCTCCCGGATCCTGGTACGCCCATGGCTGCCGACCTGGCAGCGTCCAGCACCGTGATGTGGGAACAAGATGCGGACCCCTTCGCAGGGGCAGCGGATGATTTCCCGGCATTCAACGAAGAGGAACTCGCATGGTTGATGGAGCTATTGCCTCAGAAGGGCGAATTCAAGCTTTACCCATACGATGTTCCAGATTACGCTTGA

>dTALELB5A

CGACCATGTAAAGAGGTATGCCTGATGGATCCCATTCGTTCGCGCACACCAAGTCCTGCCCGCGAGCTTCTGCCCGGACCCCAACCCGATGGGGTTCAGCCGACTGCAGATCGTGGGGTGTCTGCGCCTGCTGGCAGCCCTCTGGATGGCTTGCCCGCTCGGCGGACGGTGTCCCGGACCCGGCTGCCATCTCCCCCTGCGCCCTCACCTGCGTTCTCGGCGGGCAGCTTCAGCGATCTGCTCCGTCCGTTCGATCCGTCGCTTCTTGATACATCGCTTCTTGATTCGATGCCTGCCGTCGGCACGCCGCATACAGCGGCTGCCCCAGCAGAGTGGGATGAGGCGCAATCGGCTCTGCGTGCAGCCGATGACCCGCCACCCACCGTGCGTGTCGCTGTCACTGCCGCGCGGCCGCCGCGCGCCAAGCCGGCCCCGCGACGGCGTGCTGCGCAACCCTCCGACGCTTCGCCGGCCGCGCAGGTGGATCTACGCACGCTCGGCTACAGTCAGCAGCAGCAAGAGAAGATCAAACCGAAGGTGCGTTCGACAGTGGCGCAGCACCACGAGGCACTGGTGGGCCATGGGTTTACACACGCGCACATCGTTGCGCTCAGCCAACACCCGGCAGCGTTAGGGACCGTCGCTGTCACGTATCAGCACATAATCACGGCGTTGCCAGAGGCGACACACGAAGACATCGTTGGCGTCGGCAAACAGTGGTCCGGCGCACGCGCCCTGGAGGCCTTGCTCACGGATGCGGGGGAGTTGAGAGGTCCGCCGTTACAGTTGGACACAGGCCAACTTGTGAAGATTGCAAAACGTGGCGGCGTGACCGCAATGGAGGCAGTGCATGCATCGCGCAATGCACTGACGGGTGCCCCCCTGAACCTGACCCCGGACCAAGTGGTGGCTATCGCCAGCAACATTGGCGGCAAGCAAGCGCTCGAAACGGTGCAGCGGCTGTTGCCGGTGCTGTGCCAGGACCATGGCCTGACCCCGGACCAAGTGGTGGCTATCGCCAGCAACGGTGGCGGCAAGCAAGCGCTCGAAACGGTGCAGCGGCTGTTGCCGGTGCTGTGCCAGGACCATGGCCTGACCCCGGACCAAGTGGTGGCTATCGCCAGCAACATTGGCGGCAAGCAAGCGCTCGAAACGGTGCAGCGGCTGTTGCCGGTGCTGTGCCAGGACCATGGCCTGACCCCGGACCAAGTGGTGGCTATCGCCAGCAACATTGGCGGCAAGCAAGCGCTCGAAACGGTGCAGCGGCTGTTGCCGGTGCTGTGCCAGGACCATGGCCTGACCCCGGACCAAGTGGTGGCTATCGCCAGCAACATTGGCGGCAAGCAAGCGCTCGAAACGGTGCAGCGGCTGTTGCCGGTGCTGTGCCAGGACCATGGCCTGACTCCGGACCAAGTGGTGGCTATCGCCAGCCACGATGGCGGCAAGCAAGCGCTCGAAACGGTGCAGCGGCTGTTGCCGGTGCTGTGCCAGGACCATGGCCTGACTCCGGACCAAGTGGTGGCTATCGCCAGCCACGATGGCGGCAAGCAAGCGCTCGAAACGGTGCAGCGGCTGTTGCCGGTGCTGTGCCAGGACCATGGCCTGACCCCGGACCAAGTGGTGGCTATCGCCAGCAACGGTGGCGGCAAGCAAGCGCTCGAAACGGTGCAGCGGCTGTTGCCGGTGCTGTGCCAGGACCATGGCCTGACTCCGGACCAAGTGGTGGCTATCGCCAGCCACGATGGCGGCAAGCAAGCGCTCGAAACGGTGCAGCGGCTGTTGCCGGTGCTGTGCCAGGACCATGGCCTGACCCCGGACCAAGTGGTGGCTATCGCCAGCAACGGTGGCGGCAAGCAAGCGCTCGAAACGGTGCAGCGGCTGTTGCCGGTGCTGTGCCAGGACCATGGCCTGACCCCGGACCAAGTGGTGGCTATCGCCAGCAACGGTGGCGGCAAGCAAGCGCTCGAAACGGTGCAGCGGCTGTTGCCGGTGCTGTGCCAGGACCATGGCCTGACCCCGGACCAAGTGGTGGCTATCGCCAGCAACATTGGCGGCAAGCAAGCGCTCGAAACGGTGCAGCGGCTGTTGCCGGTGCTGTGCCAGGACCATGGCCTGACCCCGGACCAAGTGGTGGCTATCGCCAGCAACGGTGGCGGCAAGCAAGCGCTCGAAACGGTGCAGCGGCTGTTGCCGGTGCTGTGCCAGGACCATGGCCTGACCCCGGACCAAGTGGTGGCTATCGCCAGCAACAATGGCGGCAAGCAAGCGCTCGAAACGGTGCAGCGGCTGTTGCCGGTGCTGTGCCAGGACCATGGCCTGACTCCGGACCAAGTGGTGGCTATCGCCAGCCACGATGGCGGCAAGCAAGCGCTCGAAACGGTGCAGCGGCTGTTGCCGGTGCTGTGCCAGGACCATGGCCTGACTCCGGACCAAGTGGTGGCTATCGCCAGCCACGATGGCGGCAAGCAAGCGCTCGAAACGGTGCAGCGGCTGTTGCCGGTGCTGTGCCAGGACCATGGCCTGACCCCGGACCAAGTGGTGGCTATCGCCAGCAACGGTGGCGGCAAGCAAGCGCTCGAAACGGTGCAGCGGCTGTTGCCGGTGCTGTGCCAGGACCATGGCCTGACCCCGGACCAAGTGGTGGCTATCGCCAGCAACGGTGGCGGCAAGCAAGCGCTCGAAACGGTGCAGCGGCTGTTGCCGGTGCTGTGCCAGGACCATGGCCTGACCCCGGACCAAGTGGTGGCTATCGCCAGCAACAATGGCGGCAAGCAAGCGCTCGAAAGCATTGTGGCCCAGCTGAGCCGGCCTGATCCGGCGTTGGCCGCGTTGACCAACGACCACCTCGTCGCCTTGGCCTGCCTCGGCGGACGTCCTGCCATGGATGCAGTGAAAAAGGGATTGCCGCACGCGCCGGAATTGATCAGAAGAGTCAATCGCCGTATTGGCGAACGCACGTCCCATCGCGTTGCCGACTACGCGCAAGTGGTTCGCGTGCTGGAGTTTTTCCAGTGCCACTCCCACCCAGCGTACGCATTTGATGAGGCCATGACGCAGTTCGGGATGAGCAGGAACGGGTTGGTACAGCTCTTTCGCAGAGTGGGCGTCACCGAACTCGAAGCCCGCGGTGGAACGCTCCCCCCAGCCTCGCAGCGTTGGGACCGTATCCTCCAGGCATCAGGGATGAAAAGGGCCAAACCGTCCCCTACTTCAGCTCAAACACCGGATCAGGCGTCTTTGCATGCATTCGCCGATTCGCTGGAGCGTGACCTTGATGCGCCCAGCCCAATGCACGAGGGAGATCAGACGCGGGCAAGCAGCCGTAAACGGTCCCGATCGGATCGTGCTGTCACCGGCCCCTCCGCACAGCAGGCTGTCGAGGTGCGCGTTCCCGAACAGCGCGATGCGCTGCATTTGCCCCTCAGCTGGAGGGTAAAACGCCCGCGTACCAGGATCTGGGGCGGCCTCCCGGATCCTGGTACGCCCATGGCTGCCGACCTGGCAGCGTCCAGCACCGTGATGTGGGAACAAGATGCGGACCCCTTCGCAGGGGCAGCGGATGATTTCCCGGCATTCAACGAAGAGGAACTCGCATGGTTGATGGAGCTATTGCCTCAGAAGGGCGAATTCAAGCTTTACCCATACGATGTTCCAGATTACGCTTGA

>dTALELB7A

CGACCATGTAAAGAGGTATGCCTGATGGATCCCATTCGTTCGCGCACACCAAGTCCTGCCCGCGAGCTTCTGCCCGGACCCCAACCCGATGGGGTTCAGCCGACTGCAGATCGTGGGGTGTCTGCGCCTGCTGGCAGCCCTCTGGATGGCTTGCCCGCTCGGCGGACGGTGTCCCGGACCCGGCTGCCATCTCCCCCTGCGCCCTCACCTGCGTTCTCGGCGGGCAGCTTCAGCGATCTGCTCCGTCCGTTCGATCCGTCGCTTCTTGATACATCGCTTCTTGATTCGATGCCTGCCGTCGGCACGCCGCATACAGCGGCTGCCCCAGCAGAGTGGGATGAGGCGCAATCGGCTCTGCGTGCAGCCGATGACCCGCCACCCACCGTGCGTGTCGCTGTCACTGCCGCGCGGCCGCCGCGCGCCAAGCCGGCCCCGCGACGGCGTGCTGCGCAACCCTCCGACGCTTCGCCGGCCGCGCAGGTGGATCTACGCACGCTCGGCTACAGTCAGCAGCAGCAAGAGAAGATCAAACCGAAGGTGCGTTCGACAGTGGCGCAGCACCACGAGGCACTGGTGGGCCATGGGTTTACACACGCGCACATCGTTGCGCTCAGCCAACACCCGGCAGCGTTAGGGACCGTCGCTGTCACGTATCAGCACATAATCACGGCGTTGCCAGAGGCGACACACGAAGACATCGTTGGCGTCGGCAAACAGTGGTCCGGCGCACGCGCCCTGGAGGCCTTGCTCACGGATGCGGGGGAGTTGAGAGGTCCGCCGTTACAGTTGGACACAGGCCAACTTGTGAAGATTGCAAAACGTGGCGGCGTGACCGCAATGGAGGCAGTGCATGCATCGCGCAATGCACTGACGGGTGCCCCCCTGAACCTGACCCCGGACCAAGTGGTGGCTATCGCCAGCAACATTGGCGGCAAGCAAGCGCTCGAAACGGTGCAGCGGCTGTTGCCGGTGCTGTGCCAGGACCATGGCCTGACCCCGGACCAAGTGGTGGCTATCGCCAGCAACGGTGGCGGCAAGCAAGCGCTCGAAACGGTGCAGCGGCTGTTGCCGGTGCTGTGCCAGGACCATGGCCTGACCCCGGACCAAGTGGTGGCTATCGCCAGCAACATTGGCGGCAAGCAAGCGCTCGAAACGGTGCAGCGGCTGTTGCCGGTGCTGTGCCAGGACCATGGCCTGACCCCGGACCAAGTGGTGGCTATCGCCAGCAACGGTGGCGGCAAGCAAGCGCTCGAAACGGTGCAGCGGCTGTTGCCGGTGCTGTGCCAGGACCATGGCCTGACCCCGGACCAAGTGGTGGCTATCGCCAGCAACATTGGCGGCAAGCAAGCGCTCGAAACGGTGCAGCGGCTGTTGCCGGTGCTGTGCCAGGACCATGGCCTGACCCCGGACCAAGTGGTGGCTATCGCCAGCAACATTGGCGGCAAGCAAGCGCTCGAAACGGTGCAGCGGCTGTTGCCGGTGCTGTGCCAGGACCATGGCCTGACCCCGGACCAAGTGGTGGCTATCGCCAGCAACATTGGCGGCAAGCAAGCGCTCGAAACGGTGCAGCGGCTGTTGCCGGTGCTGTGCCAGGACCATGGCCTGACCCCGGACCAAGTGGTGGCTATCGCCAGCCACGATGGCGGCAAGCAAGCGCTCGAAACGGTGCAGCGGCTGTTGCCGGTGCTGTGCCAGGACCATGGCCTGACTCCGGACCAAGTGGTGGCTATCGCCAGCCACGATGGCGGCAAGCAAGCGCTCGAAACGGTGCAGCGGCTGTTGCCGGTGCTGTGCCAGGACCATGGCCTGACTCCGGACCAAGTGGTGGCTATCGCCAGCCACGATGGCGGCAAGCAAGCGCTCGAAACGGTGCAGCGGCTGTTGCCGGTGCTGTGCCAGGACCATGGCCTGACTCCGGACCAAGTGGTGGCTATCGCCAGCCACGATGGCGGCAAGCAAGCGCTCGAAACGGTGCAGCGGCTGTTGCCGGTGCTGTGCCAGGACCATGGCCTGACTCCGGACCAAGTGGTGGCTATCGCCAGCCACGATGGCGGCAAGCAAGCGCTCGAAACGGTGCAGCGGCTGTTGCCGGTGCTGTGCCAGGACCATGGCCTGACCCCGGACCAAGTGGTGGCTATCGCCAGCAACGGTGGCGGCAAGCAAGCGCTCGAAACGGTGCAGCGGCTGTTGCCGGTGCTGTGCCAGGACCATGGCCTGACCCCGGACCAAGTGGTGGCTATCGCCAGCAACGGTGGCGGCAAGCAAGCGCTCGAAACGGTGCAGCGGCTGTTGCCGGTGCTGTGCCAGGACCATGGCCTGACCCCGGACCAAGTGGTGGCTATCGCCAGCAACAATGGCGGCAAGCAAGCGCTCGAAAGCATTGTGGCCCAGCTGAGCCGGC**C**TGATCCGGCGTTGGCCGCGTTGACCAACGACCACCTCGTCGCCTTGG**C**CTGCCTCGGCGGACGTC**C**TGCCATGGATGCAGTGAAAAAGGGATTGCCGCACGCGCCGGAATTGATCAGAAGAGTCAATCGCCGTATTGGCGAACGCACGTCCCATCGCGTTGCCGACTACGCGCAAGTGGTTCGCGTGCTGGAGTTTTTCCAGTGCCACTCCCACCCAGCGTACGCATTTGATGAGGCCATGACGCAGTTCGGGATGAGCAGGAACGGGTTGGTACAGCTCTTTCGCAGAGTGGGCGTCACCGAACTCGAAGCCCGCGGTGGAACGCTCCCCCCAGCCTCGCAGCGTTGGGACCGTATCCTCCAGGCATCAGGGATGAAAAGGGCCAAACCGTCCCCTACTTCAGCTCAAACACCGGATCAGGCGTCTTTGCATGCATTCGCCGATTCGCTGGAGCGTGACCTTGATGCGCCCAGCCCAATGCACGAGGGAGATCAGACGCGGGCAAGCAGCCGTAAACGGTCCCGATCGGATCGTGCTGTCACCGGCCCCTCCGCACAGCAGGCTGTCGAGGTGCGCGTTCCCGAACAGCGCGATGCGCTGCATTTGCCCCTCAGCTGGAGGGTAAAACGCCCGCGTACCAGGATCTGGGGCGGCCTCCCGGATCCTGGTACGCCCATGGCTGCCGACCTGGCAGCGTCCAGCACCGTGATGTGGGAACAAGATGCGGACCCCTTCGCAGGGGCAGCGGATGATTTCCCGGCATTCAACGAAGAGGAACTCGCATGGTTGATGGAGCTATTGCCTCAGAAGGGCGAATTCAAGCTTTACCCATACGATGTTCCAGATTACGCTTGA
