# Supplementary material for: Consequences of adaptation of TAL effectors on host susceptibility to Xanthomonas
Source: PLoS Genet. 2021 Jan 19;17(1):e1009310. doi: 10.1371/journal.pgen.1009310 (PMC7845958; doi:10.1371/journal.pgen.1009310)
Supplement: S2 Text — (DOCX) [file pgen.1009310.s008.docx]

S2_Text. The 1 kb upstream sequences from the putative transcriptional start site of all genes in sweet orange

>Cs1g09430

TGTATTTTTAGGGCACCATCGATTTTTACCACATGACCACTCATTTAGATCATTAAAGAAGGTTTCAATAATAAGGCAGAATGGGATGAACCTTCAAAAACTTTGTCTACTCAAGAAATTTTTAAAACTGGTAGATGGTATTAACTATAAGAATAGTAAGCATAGAGGCAGTAACCGAAAGCGTGATGATTGTGATAATGATATTGACAAATTATTCAAAAGAAAATCAAATTTTTTTATTTAGAATATTGGCAACATTTATTAGTTCGCCATGTGTTAGATGTGATGCACATTGAGAAAAATATTTATGAGACATCTATGACACATTACTTCATCAACCTAGAAAAACAAAAGGTGGGGTCAAGGCTAGAATAGATCTAATTGAATTATAGATTAGGGGCAAATTAATTCATAGTGAAAATGATAAAAATTCCATATCTACAGCACCTTACACCTTGAGTAAAAAAGAAAAAAATGTTTTGTCAAACTTTGTTTGAAACCAAAGTTCCATAAGGTTATTCTTCCAACTTCAGAAGTCTTGTAAACTTGGATTATTATCGGCTTCAAGGTCTTAAATCTCATTACTGCCACACTTCAGGAGTATTTTACTTTAATGCATTGTGTAGTAAGGTGCTAGAAACAAAATCTCTTGACAAATTGGAAAAACAACATTACGTCACTATGTGCTTGTTTGAACAATTTTTCCAACCTTATTTTTTTTTATATAATGGTCCACTTGACGGTACATCTTGTTGACCAAGTCCGATTATGTGGACCTGTTCACCTTTGCTGGATGTATTCATTTGAGAGAAACATGAAATTGCTAAAGGGTTATGTTTTAAACCATTGTCGCCCTGAGGGATGCATTGCCGAATGTTATGTGGCAAAGGAAGCACTTGAATTTTGTGCTGAATAATTGTCCAATCATGATTTCATTGGACTGCCACCTAGTTGCATGGTAGATTATACAATTGAAAAGCCCTTAGGAGGAGGAAAAGCTA

>Cs1g01790

TTCAGGCTTTTGAGACTAAAATAGAGGAAAAGGGTAGCAAAACTAAAATAATTGACTCCAACTTCAAATTATAGTAAAACTGAAATAACTAACTCGAAATAAAGGAAATTTAAAGACTCTATATATTATTCTATATTTGTGCATATAATTGTAAACCTATAAACTAAAGTAATTAATTACTTGTTCAACAAGAATAAGTACAATCAAAGCCACCAAGGTCAAGCAATGCTTCTCTCCTCGAGTTTTATATTATATAAAATGATGAAATTATATTGATGCACATCAGTGGAGATATTTAGTATATTGAGGCAAATTCTTCTCATCTAGATTAGATTGATTCATAATTGAAAGAGATTACTATAAGTTCTTTCAAAATTGATAATTTGTCCCATTATTAGAGGTGTTCCAAAAATGACTGCAATCGAGTGAATAGCTACGAACTAATCAATCAGATTGAATTAGAAAAGGAAAAAATTTGTACACCCATAAATTTGAAATTGCAGCTGTCTAAGAACACTAAAAAATTATCAATCAAGGAACATTAGTTGTACTATCTTATCTGTTTGACTGTTAATTTTTAATTAAACCCAAAAAAATGTATTTTTCAGAGTGATATCTTATGAAATATACAGTTAAGAAATATGCGGCCATATTTATCATAAACTAAATAGTTAAAATATTATGGCAAAATAAAATAACTTTTATAAACAATTTGATCCTTTTACTCTATTAAGATAAAAATTAATTTATCCCTTTGTTCATTACCTTAATAATTAAAATGATGTCAAATTAACCTCAATTTTTTTTTAAATTTTACAAAGGCTTTACTACAACCAATAAAATGAGTATAATATGAGGGGCCAGCGGCCCACCGCATGTATTGTTTGGGCCTATTGTATAATTTTCTACCGAAACATTTATGGGCTTGTTACAATATTCGACCCAAATAATTCGGTTCGGATAAATGATACGGGCCAGCATCACGTCACGAAGCCGAGTCG

>Cs1g04290

ATATGTTAGAAGGATATAACGGTAATTACATATTAAAATTATCATTTCTGTTAAAACTAACGGTTTCTGTTAACAAATGAGGGTATGTATGTCTTTTTTGAAACATTAGGGGGTTTTGTGTCCCTTTACTAAAATTTGAGGGTGTCTGAGTCCTTTTCCCTATTTATAAAGGAGATTTTACCAAATTTTCATAAGAATTCTTACTAATTAGATTATTATCTATTCAGTTTTGATGCGATTAGATAGACTTGCCCTGAGGGTTTGGGTCGGGTCGTTTCACGCATGCACGCAAGCGCGTGCGCACACTCACACCGGCCCAAATACACAATCACACTAATCACCTAGCACATCATATATATATTTATGTCTATGTATGTAACTCCAATTTCAACAAAAAGTTAATTTCATATTCCATCCTATATACATGAAACATCAATTAAATAATTTTATTTCCCAGGGTTGGTGGGAAAGGGAAATGATCATGTGACCTCTTGTTTTCACTAGAAGGGGACAAACCAATTCGACTTATTCCGTCAGAGATACAAAAAGTTACTTCTTGCTAGTTAATATTCTGGTGACATTAAAATAAAATAGTAACTAGCATATTTATGGGCAAATCCAAAATATTTATGTTACTTTCATAAAATATTTAAGTGTTGTACAAATTTTATTTTACTCATTAGTTTCCCTTCTTTCTATATATTAAATACATGTTATTATATAACAAGTAACAAGAATTAGTAGAATTTGATATATCTCAATCTAATCTATTGACCCAGAGAGTGAGAATTTTTTTAATATTGCCTATCGTTGAAATCACCTCCATTTGTTGACTTTGTATATTTTCTAAGGGATTCGCTTTGATTTTCTTTATTATTTTGGAGATCCAATCCTTCAAGAAGCAATTATGCAAGACACTTATAAATATGATGAATTTGGTTGCACACTATATGCCACTCAACACTCACTTTGGTGAATAATTTCTCAAAGCCAGCAAAAGA

>Cs1g15920

AATAATAACATAAATAATTTTAAAGTTTATTTTATTTGGGTTTAATTGTCTTAGTAACACTACTTTTTGGCTGCAGGGGATCATCTTATTCAGGAGAAACAGCAGTTGCGTACAAATCAAATGAAAGAAAAAGGCTTGAGAAGAAAGGATACAGAATCATTGGAAACATCGGCGATCAGTGGAGCGATCTCTTGGGCACCAACGCCGGCAATCGGACGTTCAAGCTGCCTGATCCAATGTATTACATTAGCTGATTAATAAATAATGTTTACTTAGGATTTTGAATAAATAATGCAAGAGCCCCCATTTGTGCACCAAATTTGTACGCATGTTATGTTATAATCTCCAAAGTTAGAATAACAATGGTGGAAAGAACTTCAAATATGGGCTTCCAAGTCCCAACTCGGCCCAAATTGTCAAATCCCAAAACCTGGCCTAAATGCCAGACCAAAACCATAATCCCGATTCTAATTATGTTTCCAGATGAGAAACGGCCTGAGAAATCCTTTGTTGAGAGAGATGGTATTCAAGTAATTCTTTGATCTTCTCTCGGAAATAAATGAGAATTTTACTAAGGATAAAAAAAGCTTAAAATTTCAAGCCAGATTTTTTTGTAATTTCTTGCAAAAATAGATTCAGAATTGATTAAGGAAATCAAAGTCCACGCCATTTGAACCTAAAGGAAAGTGAGAAATTTCAGTTATTAAAATATCAATCTTTACCTCTTATTAGTACTCAAATTTTAAGAATAGTAGAAGCAAAACACATTTACAATCAGTCAAACTGTATCCACCAGATGAAACTCGATTCAATCATCCAAAGTCCACTAAGTTAAGGCAGATGCCTTAACAAGAAAATTTATTCGAAATTTTTAGTACTCTTACAAGAGGCACTTTCTATTTCAATGTCATGATACTTAATTTACATCATAATTGATGTGCCACCCACCCATCATTTATTGATAATTATCAATCAGAACTTTTGTATGAAAATGAAATAAA

>Cs1g04850

ATTAGTAAAATCCTCTCAATGGGCCTTTGGTCCAGTGGGAGAACTCTTACACTTACAATGTTGAGTGCAAATGTTCGACCCTCCATAAAAATATTTATGGGGGTTATCTACAATCCTCTTTCAATTATTAAATAATTGAGTGAAGTCAATCTATCCCTCACGAAATGTGAGTGGAGTGGACAGTTCTCAAATTTTTTAGAGGGTTTAAAGAATCTGGGCAGCGCTTCAGAGGAGTACATGCCATGAAGAATGTCCCAATTGTAGAATATGTTTGTAAATATTAATTGTAATACATGATGTATGTTAGTGGAGTGGACAGTTCTTGAATATTTGAGAGGGTTTAAAGAATCTTAGCAACGCTTCAGAGGAGTACATGTCATGAAGAAGGTCTCAATTGTAGAATCTGTTTATAAATATTAATTGAAATACCTGATGTAATATCAGTAACAGTACTGTATAACAATATATAAAAAAAAAAAGAATTAGTGAAATCCCACTGAAAAATAAATCTTACATCTCTAAAAAGAAAAAAATTTATCATACTTGAAAAATAAATAATCGCATCTAACCAATAGAATTATAATTAATAATTAGCAAGAAAATTAGAGTTATATATCAAATCTTAGGGTAAAATAAAACACAAAAGCTCATAATATACTTCACTTTTTCAGTTTATCACTATCATTTACTGTCTTAACATTTAGTATTAGCTGCAGACTGCAGTTGATCAAAAGATAATAATCATTTAATTAAAAACTTGACAGCAAGAGACTAAAGTAGTATTATTATTTTTTTTTTTTGGCCCTTTTGAAACAACCAATAACTTCATTATAATCAAAGATTAAAGTGGGAATTAATAATGAGCAGTTACGTGTTAATCAAGAAATAATTACCTGGGCCGCAGTCACCCATCATATCTATGAATAAACAGACCCAACCCAAGGCTCAAACCCAACATGATAAATCACACGTGTGCTACACACGTGCATTAGGGTTTTAGG

>Cs1g24790

AATGCCAAATTGTTTTGAATTCACAAGAGGGGCCCTTTTCCCCTTAGCCCTTTCACTCCCTAAGAAATAAAAGACCACATCTAACTCGGCCAAAAAACTAGAATAGGAATGAGATTCACAATGCCATCATTGTATGACCGATAGTAGTACATGGTTGATTTGGAAAAAAACTCTTTTCTTTTTTAAGTCGTTTGTTAAGCTTCCTCTCCTTATATAGAGCTTCCCTTCAAAAGTTCTCAATAAATTCACTTCTCTCATAGACAAGAAAAATATATGTATGCATAGAGTTGACAATTTAAGGGCTTGAAGTGTAAGGGTTGGGCTTAGACTTTTACTTATTTTTTCAAATTTTTTAAAAATTATTGTAAACTTGAAATGAATTAATAATAGTCAAGTTACTATAATCAACAATTAATTAAAGAAAATAAATATTTCAAAAATAAAATATAACTAAAGAAAATGAAATAAATAAAAGTTTATAAATTTAGTATTTTTATATTGAATTCTCTAATTCACGGACTCTCTTAATTAATTAATACTTAAAAAAGAGCTTAGCATTATAATTTTAAGTATTTTGATTCATTATTGATATATATATAAATTAAAGATTCAAGCTTTCTTTTTTTTTTATTATTTTTTAAATTTAAATTTATTATTCTAATTTATAAATTTATTCTTAAAAAAAAAGAGAGTTTGGGCTTAGCTCAGGTTTTTTCTCCTAAACCCTTGTCTTGACCCTGTGCAGAGGCCCGGGGCAAAACTCCTAGAAATGAATAGGACCAACCCGAGCCTTTTTTGCCATCCTTACACACCCATCCCAAATACGTAGGTGTTGATGACCATGATATTTTCAAATAAACAATTATTTTTTAAATTAAAACAAACAAAAATTTTAAAAAATAAAATAAATTAGCATAAATATTAAATAGACAGGGTGTTTTTGGTATTTAGGGGGTGGATTTATAAAATATATTCATTTTAAAATTTTTTATGGGGGTGGG

>Cs1g09740

ATTTTGTTAAAATCAAGCAAAATATTGTTTGTACAAAAAAATTATGGTTTGAGTATATTTATTTACTTAAGAGAACACTTGCATCCAATCATCGTATCAAATTAATCATTCTTATTTATTTGGTTGTGTGTTTGTTTGTGTTTGCGGGTTGAACAACTTAATCCTGGATGCTTAGTTGCATATTTCCCTCTACCTAAGCCTTTAGACAATTGTCTTGCAAATTTGAATAAGCTCAATTGAACATATATACGTATTTTTAGAAATTTATTTATCTATAGAACTTCTCAAGAGTGGACGTTAAAATATGATTTACGGATGAAACTAAAATAAAAATATACAGATTTTAGTATACTTTTCTCTTTTAATGTTTGGAGTCTGTATTTTCTGTCTTATAATTGCATGCGCACAAGATTCATATTTTATCATCCGCATTTGAGAAGCTCTGTATTTATTTGTTATTTATATGTAATGTGCATGATTAAGTGCAAGATATTTATCTATTTACATTTTCAAGACGTTCCAAGAAATACCCCAATATTTTGGCAAGATTTGTAAATTTATAAAATCTTTTAGAACTGAATAAATTGACTTTTCTTATTTTTTAAAAGATATTATTTTTTTGTTTTATTTACAAATCATTAGTTGCTTTGAATCTTATATTTAAACAAGAGTTCTGCTATTTACCCCGGGTCAAACCCCGAAAGCTAAAACAAAACGACGCCATTTCCTCTTCTTCATGAAACGCTACCGTTTCACCCTAGCAAAAGCAAACGCTTCTCTCCTCTTTGTTTTCCTCTTCCTCTGCTCTTCGTTTCACCAGGGAACGACATAACCCAATCCTCTTCCACTCCTCTTCGTATTGTTCGTTTTGTTTAAAATCCAAATTGGGGAAATTTCCTCTTAAACAAATCTTGTTTTGTATAAAAAGGATTTGAGGGCAAATTAAGATTTTCAACACATCCACAGAAAGAGAGTGAGAGTGGTGAGTGAGTTATCAAAAA

>Cs1g13180

ACAAATTTGGTGTCTAGGGTTGTCTTCATAATGTTAACAAAACCAAAAAATAAAATTTTGAAAATTTTGCAACACTTTGATAATTAGAATGCATGAACTTTTTAAACTTCATTAATAATGGCAAAATGAATTAATTATATGTATATAGAATTTTACAAATATCTTTTTTTTTAATAAATAATTTTTCATCATAATTTAGTGTGAAAGACTAGGATAATGTATTGATGATCTTGCAACTCTCGTATATCACAACTTTCAAATTTTTATTCACAATTTTCGAATGATCGATATAATTTTTCAAGTTACACGTCACGGGATAAAATTTCTTAAATATAACAACAATTTTAATGAACATAAGGAAAATATTAATGACAAATACTTAAATTTCAAGAATGATTCTACATTTATAAAGTTTTGATACAATTGTAACATCTCAGATTATGTGATATTTAACGTGTCATTCAATAATAAGCGTAAATAATTGTAAGATCAATTATAGTTTCACGTTTCATTCAATGACTAATGCAAATAATTGTCATATTAATCACATAAAAGTCAAATTTTATTTAATACATAAGTCAGTCATTAGTCTGTGATATGTTAGTCAAGATAAGAACTTTATATTCCAGTTTTAATATTATCATCTTTATTTAGCTAAAATGTGAACTATAATTTACAAGATTTGCAAACATGTTGAAACCTTATTTGATATTGAGGCTAATAACTATAGCAAAAAGTTACAATACTAAAACATTTAGTAAACATTAGTTATTTTAGTTCAAAAGTTATATTGATTTGATTTTATATTTATATGAGAATATTTTTATTATAATTTTTCATAATTTTTTAATTCTTCGGCAATTAAATCATAAATAAAAATATTTTGAATTGCCGTAAGTTACGGATGACGTAACTGTATTTAATTCAGAATTAAATACTGGGACGGGATGGGGTGTTTGTTGCTTAACACAGAATTTAATAAATCAATAAATTACCGATGA

>Cs1g26100

GCCCAAAAGGCTAAAAAAAGGTTTCGGTTTGCAAGTGGGCTTTCGCTTTTCATTTTTATTTGGACAAATAGTGAAGTTTCGCATAAAGTTTAGTTACTGATTGGTCGTGGCTAAGATACCAAACACCTGCGAATTGAGTTTTTAACTTTTGAGAGTCGGACATGGAATGTGTGATGGGCCTACCAACAAAACCTCGGTAGTTGGGATCTTTTTGAGGGAAAAAAAAATATTTGTTTTCTCGAAAGAATATTTCTTTTAACTGCCAATTACACTTTTTCTCGCCGAGGCTGATCAATTTTTTTCTTTTAGTATAATTCAAAGAAAAATTCACATATTCCCTATGTCTTGATACTTTTTTTTATTTTTTTAACAATTGTATCATTACCATTTGAAATTTCGAATATAACCACATCAAAAGATTTTATTAACTGAACCAATAATTTTATATGAAAAAAAGGTCTATCTGATATCTAGAATTCCAATATTACTATAACATACGTAAAATAAACTTATTTAAAACTACTTTAACCAAGTAATCACATAATTGCTGGTGTAATTAAAAAGGTAAGACAATGCGTAAACATGTTTATATAAAATAGTAACATATGTATTAGATTTTGTAGTATAATGGTGAAATTCACTCACTTCCTATGGCAGAATGAATTTGTTAACTTTTTTTTTTCCATCCAATAAACTTATGATTGTAAAATACAATCGTAATTCAATAAAGAAAAAAGAAAAGAAAGAAAGGAGCGTCAATGGCTCGGATTCAAAAAGAAAAAAAAAAAAAAGAACAACAACAACTCATTTTAAACCGTTGATCTCATTTCCTAGTAGTCATCGAGGGAAAGATGTGAAGCCTTGCACGCACTGCCGGGGACACTGGAACATGGAAGTGACGGTCAAACTCGAACACGCCAGCCAGCGTACCTATGCGGAGAGAAAAAAAAAACAAAAATAATAAAAAAAATAAGGGACAAGGAGGGTGAGTTAAACTCATT

>Cs1g22100

TTTAGTGAATATGCAAGATATTTTTTTCTTTCTTTTTCCGAAGGTGCTATTATTATTATTTTTATGTTCGGTATCCTTTTGCTCAAATGTCTTAAAAGATAAGTGTATTGCAAGGTAGTGTCTTACTTCATTAAAGATACAAACTTTTGTAACTCAATTTTGTAAACATATCATTAGTCTTTTGTTAAACGTGCAAGGCACCAATCTAATTGTATTCAAGCTTCACGAGGCATTGCTAGTGGACTTCCAGCTCAATAGTGGGCTGGGCTGGTGGTCTTAACAATATTATGACACGAAAAATGTGAAATAGAGATTTGTGTTTAGTGTGATTAGCGGGAGTAAATTTTTGATCTACCGTTGTTATATTAATCACTTGATAAAAACATATAAAAGTAAAATGGGACGTGTTCGGCTTATATATGTAATCGCTGGATGTCCATATTCATTTTTTTAAAATATAAATGAGAAAAAAATTATTGATAAGAAAAAGGAGAGAAAAATAACAAGTTAAATAACAAAATGATACCCAGACAATTCTCAACCATCTTTTAAAAGGTTTTTTTTTTTAATTGGAAATTGATGAATAATTCCAACAAGTGTAAGAATTACAAGATTCTAAGTTTGCCTAAGAGTCAACAACAGACTTTTCTCTTTCTTTAGTAAATAATGACAATTAATCCATCTATTGTTAAGAGGACAAGGAGGGTTAGAATATTAATCACGAGGCACAAAACCTCTCTAACCATACTCTAAATCAGCCTCCTTTATTAGAATGTGTAGTTATGATCGGCATACAAACGACGACACGCGACTAAGACAGAGAAGCGGAAAAGCCCATCCTGTTGGTCATGTAGCAGAGCTGAACTAAATGAATCTATGATCTGTTTGATGATATTATTTGAATAATATGATTATTTATATTTTAGAGAAGCCGTGAACCAACAGGAAACAGAAATATTATGAAAGTTGAAAGGTAGCAAGCGACTTGCCCAAAAGATTTT

>Cs1g22150

GGAAACTACTCAAAAAAATCTTTCTAGAATAGAGTTTAACATTCACTAAATGAGCATAAATTCTTTGGTAATGATAACTCCTAGCAATCTAGAGAAAGTAATGGTGTTTGAGAATCAGTGAATGTAAAGCCTACCCTATTGTACTGCCAAAATGGTCCACTTCTGATATTATTTGTACTACACCATATCTTTCATAGACCTTTTGAGGTGAGAACAGAGCATCAACTGTACTGTTTTATACTGGTATAAATTAGGAAACCCAACCTTTGAGAGATCAACACTGTGAAGCATATAGATGAGTGGTCCATAATTTTGCAAAATCCTGCATGGGAAGATTTGAGTACTGTGGTATGCGATAAATAGAATAGAGCACATGATGCTTGGGACCATTTATTCTAGTGATACTAGGTGTTTTATTGATGCAAGTTTTACGCCTTTAAATGGTTAACCAACGTGATTCTTTTGTAGGTAACTATGTTTTCTTGGGCCTGTTCTAAATCCTTGATTTGTACAATTGTTTACGAGTTACATGTTGAATTTAGATCATCAGAGTTTACAGCTCACCTTCACCATTAATCATTTATTCAATTTACTTGGTGTTCACTAATGTTGTTACGTAGTCTAAAGCGTGGCCTCTGAGGAAATTGATTGCAATTTATATAGTTGGACTGTTAATTGCTCTACTGTCAGTTAAGCTAAAGCTTCTGTTAAGAAATCATGTATGATGCCATAGAAGGTTCTTTATAAAGGTGAAAGTATGGAATCCATGTGTTGATATTGACCAATACTAAAAGTTGACATCAGCAATTCCCCTCAAATGATCAAGCATCACTTTGTGGGAACACCAGATAGAATTGAGAGTTGATGTTAATAATATCCAAGGCCACTTGCATTTCTTTTTAATGCTTTTCTTTCTGTCCATTAAACTCCTTAGTGATCTATCCCTATTTAAGAGCAGCACAATAAAAAACTCAACTTACATATACCCTTCTGATTGGAGA

>Cs1g21320

GAGAGCCTGCTGGAGAGTATGGTAAAGCTAGTTAAAGAGAAGTAATGAGGTTAAAGAGTTTTTTGCAGAGAAACTGGCATGGCATGGCATGGGTTAATGGTCACTTTATTGGCGGCAAGTTTTTTACAGTCTCTCTTTCTCTCAAAAAAGGAAAAGAAGCCAATTACCTTTCAAGTCTTATGCATACGGTGTAGCACAAACACTGCCTCTGTGGGCTATGGTCGCTCTCAGATGAGTTGCTATCCACAATGTCCTTTTTCTGATTCTATAATCTTATTTAGATTTTCTGTTCCCCATTTGAAATACCAAACAAACCCCATTCTTTGAAAAATTTATGTGCATTACTCACCTCTCAGGTTATTTAATATTATTTATTACATGTGTGTGTTTTTTTTTTTTAAATTTCCTTCTCTTTTCCTTATTGTAAAGAAAAGAAAAGGGGAAAGTTTTCTTACTTATTTGGGAAAATTGTTAAAACAAGACACGCCGAAGAACTCCTTTTCCATTAGGCTTCAGCAAAATGAGTCAAATTAATATGCGTCTTAATTTTGTTTACAATCCTTTATTCCCACATCATAGCCTGATGATCACACGCTTGATCGTCATTTACACACAACCACAATTATAAATGCGGTGCTGTATTTGGTCGGTCGTTTTCATTAATTTATTATTGAAGGCATCGCCCCATGTTTCCCATCGGGAGAATAGAAACAGCATGCTTTAAACCTTTTATAGCCGGTTCCTACTTGCAGTAAGCAGTGTCAGTTGGCATTTTGTGAGTAACCTAATTGCAGAGTCCAAATACAGAGATGGATTCACACTTGACATCTTTGCGACATCAATGGACTGGCCTGGCCGGAGCCGGCCAATTTTAATTGAGACAATAAAGCCCTTGAGCATATGCTAACGCATATACTTATTGGCGGATGTAACGCGGTAACTCTAATAGCTTATAAGGATGAAGAATCTTGCTGAACAAAATTTAACGAACAAGAAGAAAT

>Cs1g25820

AATTTGTCTACATACGATCATAAATAATTCAACGGTTGATTGCAACATAGCTATATTCATGCACTTAAGAAGCAGCTTATGATTATTTGTTTATTTATTTTGTTTTAATTACAATACACCCCACCTTTTCTCAATCCTGAACTAAATGTATCTTATTGGCCTCCGGTCTCTTACTTCAATAGTTCAATGTAGGAGATAAACAGTTTGCGGTGTCCTTCGGAAATTAAGCAATTTACTTGTAATGGAATAATGGATCATTTTTCTTTGTGCATACGTGTGGCTTCCCCAATTATCACAACCCCCCAAAAAACATGAATCTTCCCATCAGCTTTCACAAGTGAAATCGTGTCAACAAGTGAATGGAATTGAATTGTAAAAGGTGTTTTTTTTTTCTTCATGACGAAATCTGATGAGTCTTGTTCTAAGTCGTTTTCTAACACATAATTTATATTTGATAATATTAAAAACATGAACCCCCCAACTGCTTTTTTCTTTTCTTAATTATTATATCTATAATAATTATTAATTATTAAATTTATTGCAAGGGCCGAGGGACCATCCCATCCTCATTTATAGAAGCACATGCTAGGTTATCAAACTATATATTTTGGATTATTTTATTATGATTGAAGGGCCCATGTAGATTGTTATTAAGTCCTCCATCTGTTTTTATGGATTCTGGAACTATATTTGGGTCCAAGTGAGTTCTCTTTTTTTTTTTTTAATATCCCAATCCAGCCCATTTACTTTCATTCCTTAGTCCCCACCTTATCAAAGTGTTGCATTTGCTTTTGAAATAGTTAAATAGTTAATGCGTGCTGCTCAAAGCAAGGGTCCACTGGGGTTCCCCCACGAGGGACGACGTGTGCCTTCCAGATTCTTCCACGTACTCCTTCCTAGTCACAAGTGTCAGTATGCGACGCAACTTACACTCTCATCTTTCATCTTTTAAGTCATTAATATATATCTCCTTCAAATGATTAATGATGTCCTAGCATTAG

>Cs1g08780

AATTTATTCTTCGCTTGCCTTTCATGTTCTCTTTTTGTTTGTGGGAGTAGACAAGTAGCATTCAAGTTATAAATCTGTAACGTAGTTACCGGAAATGGATCGTTGATGGATTCATTAATTGAAGATGCATACCCATACCTATTCTGGCTAATAACATTAGTTGCATCTCTCAGAATTTGCTTGCACAAACGCGGAAATTTTCTTTTAAAACATGAAACCAGAGAAAAATGCTTACGATAGAGAACGGGGAGAAAGGGAGAAAAAAATAATAGGATTTTTGGGGAATCTCTTTTGAGTGCAGAGCATATGCAAATAGCTTGCAGTCAGATAAAGAAACAACACCCATTTGGGACTTCGCCATTGCCCACAGGTGACAAAAAGTGTACTCTTTATCTTTCATCTTTCTTCTGGGATCTCTGTTTCACTTTCCAATCTCCCGTTTCATCTCTCATCTTTCCCTATATCAAACCGTAAGGTAGAGTACTGGGCTTGGATGCTCTTAATATATAATAATATTGAGACTAGTTCTAAGTAGTTTCTGAATTATAATAAGATCGGAGAATAAAGCCAACATAAAAAAATTAACATTTCTACAAACAGTAGTGTCAATACTGACATATTATGAATTATTTGATTGTAATAGTGATTTGGCTTCTTAATTTTCTTTGTCGGTACTGCATTATTTCCCATTCAAAAAAGCTTTCAAGTGTTTTACAACATCATCAATCTTTTTCTAACAATATGTCTGCTATATTTCTCATTTATTTGTACTGGATAGTTCTGTAAATTTATGCAAACAGATTATCATCTAATTGATCTCTATATGGTTCATATTAAGCACTACTACTACTTCCAAACAAACGAGCTAGCTAGTTCTGAAAGTTTACTTTTAATTGTCAAATTATTTTCTTAGCACAGTCTACTAAAAAAAGAAACAAAAGAAATAAAAAAGAAGTAGCTTGTTAATGACTGCTCTGTAATGTCTATTTTTCATGTTAATT

>Cs1g16760

CTCTCCCCATGGGAGTCCCACTCCCACTCCCCACTCTAAAAATGAGTGGGGCCCACGAAGTAGGATTCTCAATCCTTCCCTAATTAGTTGAAGTAAACACTGGAGTGGGAGGAATCCACACTCCTCACTCCCACTCCAGAAAGTAAACACAACATTAGTGCAGTCAAGTCTTCCATTCTGAGATATGTTTGATTTAGGCTCTTGTAATTATTTTACTTGTTGGCATCATAGGGATGATCCTCCGTCAACAATTCACAAGTGTGATTGCAATCAAATAATCCAAGCATCGGTAAATTTAATTCATATTTAATATATATATATTATAAAGGATACGGTTATCATATTGAGTATACAATCCGTAAATCTAATTCATATTTAAATTTTTGAACACATTTCAAACAAATTATTACTACTAACAGTAACCAAAAGAATGATGCACTTTTTTATAAGTTCACATTTTCCCATGGTTTAAAAATATGCGTGGTATTTCAATTAAAATGAAAAAAAAAAGATATATTAAAAGCAAAACAACTAATCAATCCATATAAACAAAGTAATAAAGCCAAACTTATTTTATGGATGATATTATTCTTCTTATACTTTATTACAAACAAAGTAATAAAGCCATTATATATATTACAAACTATATAATAAATAAGAGTTACTATAATCCTCTTAGTAAAACTAATAATTCAGCTCTTACATTCTGACAATTTTGAAACAAATTAATCCATCAGACAATTTGATTTTGGGGTTATAACAATCAAATTATTGCACTGTATTAAAAAATTTTGACGAAATTCAATTGCAAGACGGTGTAAAAAATTTGCAAACTGTGGAATACTTATAATATTTAACAAAAAGAGGGATTCAAATGTTAATTAGTCCTTTAAGACTTGGGTTGGGCTGTGGGGTTGAACAAACCCACGAGCCCAAATTATGGGCTGGGTGCGTTTTGTTTACCCGCCCGTTTCCAGAAAATGGGACCAAAAGAAACTAGG

>Cs1g18590

GGTAGATTTATCAGGAGGTATTCGCAATCAAAGAACAGCTCGAAGCAAAGAGATGCAAGTAGGATCCATCTCCTATCAAACAAATTCAAGTGTTGAGATAGTCCTCTAGTCACAACACAACTCAACTAGGTCTCAGCATCAACAGCTACAATTTTTTTTTTTATTTCTTCAAGAAATACACATTTATATATGATTTTTAGGTGGTATAGACAATTTTTTCATTTGTTCCCCAAATTTTCTGTCAATGACATAAAAACAAATCTATAAAGCATATACCTATAGTAATCATGTCTTCTTTTTTTTAAACTTTTTTTTTTAATTTTTAACATATGCTAGGCAATAAATACAATTTTTTTATTAATTTATGTAATGTGAGTGTACAAAGAAATTGTATGCATGAAATAAAATTTAATAGTAGTACGTAGTGAATATAACTTTTAAATAATAATTTTGATAAAAATAGTAAAGATATTATAAATTTTTTTATTTTATGAGTGATTTGATTAAATCAACTTATCTTTAAACTACAATTAGTGTCTATTTATTTATATAGCTTTAGAGAAAAAAAAAATAGATATTAGACTTGGGAGTTGGGACTTCACTTTCCCTCACAACTAGTAGATTCCAATAGGCCTTCCGAGAAAATAGAAGATCAAAAAATAGAATTCAACGAGCAATACATTTTATATTAAAATGCACATTTTTAATAAATTAAAATTATAGATTCAATTTAGACGCAAACCAAAAAAATTAAAATTATATAAATTATCCGGAAAATTCGTGGTAATCCAACTCAAGCCGGCAGAAATACGACAATATTTTATATCAATTTAAAATTACTATTAATAGATGGACCGGGTAAATGATTCCTCGATTGGTAATGGTACCGTTTACTGACTGGTCTCTAAAAACTGAAACCGAAAATGCGGGAGCATGACATCACGTAAACACACCCCAAAAATTTGAGCTTCTCAAATCTTTCTGTGTCCAATTACTTGTTC

>Cs1g04640

AATAAAAATAACACAAATTTATCACAATAATGTTGTCAATTTTGGACTTTAATCTACATGATTGAAAAGTGTACAAGAAAAATATAGCTAATTTATTAAGATGATATTGTCAATTTCGGACTTCTAATCTATGCTATTCAAAAGAAAATTAAGTAACAAAGAAAAAGATATTAGTTAAAGTCAAAAATATTTTCTTTTTCCCATTTTTTTTAATAATATGAATAGAATGTGAGAATTAGGATTTATATTAGTAAACGATGAGTTCTTATAAATAAAAATTGAAAGAGAACAAAAAAGAGAAGGAAATAAAATGAAAAAAAAAATCTATCAATGATTTTTAAATTTAAATAATATAATTAAACATAAATTTTTTTTTAAGAAAAATTAATCTCAAGCGAGACAGTGGTCCCCACGACAACAAGGACGGACAGGTTTGGGCCTACCCAATTTACCGCCAATAAACTATCGCGAAGTCACAAACAAAAGGCACATTGACGCTCTCTTCTCCGTTTGGCGGGAAAATTGAAAAGAATAAAAAAACCAAAAATTCAAAACCCTAATTGCTTCCAAATTTGAAATGCAACATTTAATCTATTTAACCGGCCGCCCCTTCTCATTTCTTCTCGCGCACACTACATGTTCGATAAAATTCCTCAGTGAGCAAAAAAAGTCGATAACCAATCAAGAATCAAGATGTACGTCGTGAAGAGAGATGGAAAGCAAGAGGCGGTCCATTTTGACAAGATTACGGCGAGACTGAAGAAATTGAGCTACGGGCTAAGCATCGAGCACTGCGACCCGGTGCTTGTGTCCCAGAAGGTGTGTACCGGTGTCTACAAAGGTGTCACCACTAGCCAACTCGATGAATTGGCCGCTGAAACTGCAGCCGCCATGACCGCTAACCACCCCGATTATGCCTCTGTGAGATTCATTAACTTTACCTACTAATTGGGTTTGGTTTTACAATATTTTGTTTGTTAGTCAATTTAGTGAAAATGGCA

>Cs1g25430

TGCTAATAGCCATAGCCAACTCTTATGAAAGTTGCATTCGCATCGCCTTACGAATTTGACTAGTAGATCAATGCAGGCTTTTCATTCTCTTTTTCAACCATTCGGCTCCAAAGTCTTCTTAAGTAGCAGAAGAATGCTCTTGGGCTTTGAGTTTGACTATCAGTTGCGGTGACAGTCTGAAGACCGTCACAGTTTTGGTCACAGCCATGACCGTTGGCCTTTGACATGCGGAATGGTTGTCACGAGAACAACCTAAGCAAAGGTATCATGAGCTAATAGCGATGGATCCAAGTCGTTCCCTGTATTTTGTAAAAGCTCTCTTGGGAGACGGAGAGCGCATTTACATTGAGCTCTTCAGCGGAAATTGCACATATGTAAGAATGTCAAATCAGTATGTCCGGTGGTGAAATTGAGAAACAACACTAATTTGAGCATGATCACTGTACTCGGGACTAAATATTAGCTCATTTGTTCTTGCGAAAGTGAGCGTATAACATGGGGTAGAAAGAGTCCCGACAACTAGAGCATCAAAGTCGTGTCCGCTCATGCTTTTTTACAACTATAAGAGAATCCCACCCAAATTTTATTATTTTACTTTTATCCTCAATTAAAATATTATTACTATTTATCAAAAAAAATATTATTACTAAAAATGAGGACAACAACTTGTGCTTACCACAGCCACACCATTCTCTGCTGAGTCCCTTTTCTGTGTGGAGCTACTAACTTAACTAGTTGATTAATGAGTTTTTTAAAAATAGATTATAAAATTAATGAAATAAGATTATTAAATAAATAAATAATATTTTAAATATTTTAATATTTAAAAACAAATTTTAACTACTATTCTCAAAATTTAAAATTTAAATTTTTATTAATAAACACAAAATAATTTATTTAATCTTAAGAGTTCTACTTAACAAATAATCAATAATAATAACACTTATAATTATTGTAAGTCATTCCAAACAGTCGTAAGTTTAATATGATAATTTTAATAA

>Cs1g23110

TTAGCTTAGCAGTAAAATGACCCAATGTGTAGCACACGAAGGGCAATCATTTTGGCTCTTTAGTTAACAAAATTCAAGCCAAAAGTATCGGGCAGTGGCTTTCTAGTTGGGTGTAGGGACATCGACTTTACGCGTGATTCCTTGTGATTGGCCCTCTCCTTGAACTCGTAATCATGGTTTTGAACAGCATCTAATCTTCATCAAATAAACTCAAGCCCGGCCCCCAACAAAGATTATTCTTTTGGGAAACTTGATTACTAGTATCATTATATATGATGCTCACTACCGATCTACACTCTACGCTCTACAGTCTCCACCACTTTCAGTGCTTCATTTTATCAAATGATTTAACAAATTCCATTATAATGTAGTGCATCACCCACTGGAAGTCATCTTGTTTCAAACAAAGACACCCACAGGATACACTGGTATTGCAAACGAAAAATGATCTGATTTTTCGATCAACTAGCTCGTTCACAACATACACAATCACGAATAACGACATAGTTTTTACTTTAGTATAGATTAAATGGTAATTTACAGTTAATAAAAATGAAAAATTTAAAATCGATAGCACATATACAATTAAATCTGAAAAACAAATATGCATGGTAAGTTTTAATCACCAATATAATTGTTTAATATTCATTTTTTTTAAATGTAGATATTATGTAGATTTAAACTAAATTCTCTCAAATTTTAGTGCAATTAAGATTCAAACTCTAATGGGAATTTGCCAATTTGGTATTGTTTAATGCGATTATTCAATTTTTTTTTTTTGGAAGAATATTACCAGGTCTTTTCGAATTGGACTTCTCACAAACAAGGAAAAGAACAGTATGATTATGATCAGAAGGGTAGAATGGGAAATGGAGAAAAGGCCAATTACTTCCCTCCAAAAACCACTAACAGCAAAATTCCAAATCGAAGACCACTACAATTTTTTATATGGATTAATAAAGTGGCTTGCTGAGATTCTTTCACATAATTGTATTCCTGTA

>Cs1g23410

GAAAATGAAGCATTTATTTTGGTCAAAAGTACAAATTTAATTTCCAATAGTCAAAATCATAACATCCGGGTGAAAACCCTAACCTGATCTCCAAAGCACGGCTAAAGCACTCAGCAGCCTCGCCGTAATCACTTTCTTTCAAAGCATTGGTTCCTTTCTCCATCAACTCGTCTGCAAACTCTACCGTCTTCTCCCGTTCTCCGTCGGCGATGGCACCGGAAGTTTCACAGTTGTTGTTGCATGTCGACTCGGTACCGCTCACAGTCACAGATTCCATAGTGGCTTCGACCGAGGCTTGGGTGGTTCCTACTGTTTCTGTGGGTTGCGCAGTTTGCTCCGCCACCGTCTGTGAACCTTCTTCTTCCGCCATTTGCTTCGTTTCAAAATTTGTTCAAAACGGAATGGCCGAGTAGGAATAAATATTTTTATTAGACGCCGGATTTAGCGAAACGACATCGTCTCTTAAGGCTGTATATGTTAAAAAGTGATTATAATTTTATTTATATTACCCCGAGAGTGACAAAACATTAAGTTCTACTATTCCTAAGATCCTATTAAAAAATAATTGTTTTACGCAAATCTTTCTCTCTCTCCTTTATAGAAGTACTTCTTTGCACTATTTAGAACAACAGTTTGGGAATGAATTTGTAGTTTGAGAATTTAAGAATTTATTAGATTGATCAAACTGAAAAATGAAAATTAGGGTTTATAATTATATAATTGTATGTTGAATGAGTAATATATTTTTTGGAGTTATTGAATATAGTCCTCAATTGTAAACTATTATTGAGATATTTTAATAATATAAAATTTTATTAAAATATGAGTGTTCAAAATACTTTTTTTTTGTATGAACAGTGCCCCTCCTATTTAAATAATTGATTGATTTCTTTAAAAGATTACTCATATTAAATAAAAAAAAAAAGGTGGGGGGTGCAGGGATATTTGAAGGTTAAAATAGTCTCTTTGTTTGAAATACAAACATACAATCCTTTTTCCTT

>Cs1g25800

GGCTGGTAAAGCTGCACTGCATTGTTTATTCCAGTGCCAGAAAGTGAGAAAAGTTTGTTTCGTTTCCAGCTTGGGGTAACTTCTTGTGCGGGAAGATAGTTGTAGTGAAAGTTGTTAGAAATTGATGATATAACTTCTTTCATGGAGGGAACAAAAAATCGTGTACCACAGCTTCATTTTTTCTTATTTGAAAAAAAAAAAAAATAAAATAAGAATCTTATAGCATTTCAGAAATTTAGGTAATGTGTAAGGTTTCGATTTTTGCAGAAGGCTTGGACGATCTTGTAAGACTGCCTCTGAACCAGGGCTAAGCAAAGAAGGTACCTGGCTCCTAAAATGAAGAGATAGGAATGGTCTGGTCTGGTCTGGTAGTAGGAGAGGAGTAACCGTGTGTGTAGAAGAATCATGCGTGTGATGCTCCAGTTTTTGTTTTAATAAATTGGTGAAACGGTGCTTATGCATGTTTGTTCAATGGAGAAGGAGATTTGTATTTAAATATTAATTTTGAATAGAGCAAATAAATATTGTCAATATATTTTCCTTAACGTAGACAATTGAATGTCCAAAACCCCGAAGGCATTCAATGGCCTGCTATTTTCCTTCATTTTTACCACCGTGTTAAGAGAATATTGATATATTGCCACAAATTTCTCAGGCTTGTGCATTATTTAATTAGCTTTTGAATGATTTTGTTGCCCACATTAATAGAGAAGATACAAATTTACTTTTATATAAATAAGTTGGGAGGAAAAATGAGTCCCAATATTCCATGCTTTCAATTTACCGAATACCTTGAAAAGGTGGAATTTAACAACTTCATAAGAAATTAGTATAAAGTACAATTTGGCGTAAAGTACACGACCCAGAATCAACGGTCTAAGATTTATTCAGAGCTTACACGTCAAGCGCACAAGATCACGCCGATCCAGAGCGTCTAAAGGCAATTGAGGATCTTACTACGTATCAAGTCAAAACGCAACCTCGTTAACGAGAGAGAGAGA

>Cs1g19770

GGCCAAGAAAGTCAAAGCCGTGGGTATCAAAATCTTCTTCAAAACCGTGTTCATCAAAGCTTGGTGGGTGAAAACCGTGTTCAATTTTCAAAGCCATGTGCCAACCATTCTTGTCAAAGCTTCTAGATGCAGCCATGCCAACCATTCTTGTTTATTCAACACTTCTAGATGTAGTCAATTTTGACAAGAAGAACATATCACTGGGGATTTTTCGAAAGAGATATGAAAAATTGGAAATCGAATATATGAATGCTGAATGAAAGGGTGTTCTATTAAATAATCTATTAATATTTACATCAGATTTTTTTTAGGATCACTTTAAAATCAAATCTATTACTACACAATTTGTATCACACATCAATAGAAAGATGCCTATTGCTAACCAATTTTTAGTTGAAAGAGGGAACTATAAGAGTCCTTGAAATTAGACTCAAACACATACCGTGTTAAACGTAGTATTTTATTTAGAGTGGGACTATTAGCACTTTGTAATTTTATTTTCAAACTCCTTTTCTATCAAAACCTACTTTAATATCCTTTTTTATTTTTTTCAATTTTTTTAATTCAAAAAGAAAAACAATTCAGTTTCTTCCAAACAACTCTAATTACAAACATTGTTTTTATAAATTTTTGAATTATTAAATCATACATATTTTACTCAAAAAAAAATCAAATATATGTATGAATTAAGTAGAATTAAAGTGGACTCAAATTATTATTATTTATTTTTTCAATTAAAAATAATTTTAGAAAATTTTGTAAATTAAAAATAAATTTACAAAACTTATTTTTAGAAAGTTTTTAATTGCCAACATAGTAACCCGGCCGAATGCATACACCGATTTTGTCAATTATTCGTCAGGTTCTGTCCAGTCTCTTCATAATTATTGTCCAGTCTCTTCAATTATTACTTTTCATGTGAACCACACAAATTTCGGGTGTCACAATTTTATATGGTTTACATGAGAAGTGTAAGTTACAATGATCGTAACTTCTGAGAC

>Cs1g15500

NNNNNNNNNNNNNNNNNNNNNNNNNNNNNNNNNNNNNNNNNNNNNNNNNNNNNNNNNNNNNNNNNNNNNNNNNNNNNNNNNNNNNNNNNNNNNNNNNNNNNNNNNNNAAAAAAAAAAAAAAAAAAAAAAACTCCTCACAATGGTGTGTGTGTGGATTCCACGTTGTGCCCCGAATGTGGTTGGCGTTTAAACAACTAATATTTCCATGAAACGCAGGCCAAATGAAATTACAATAAACAAATACTCGTAGCAAATTCAACGAGCTTTTGATGATTTGAGTTAATCCACCCGATTTGAGGCCTAATTATGAGGACCTTGAACAGTGGTTTTGTTGTGTGATAGCGTCATGCATTTTTATCCACTAACTTCATTTCCTCCAAACATGGCCCATCTTCTTTACATTCATTTTTCATTTTCTGATCCAAAAATATCATAAATATTATATACGCAGCAATAGTTAGGAAAAATTTTCATTATATTCGTAATAATTTCAATATTTAAATGAACATATTAAATCCATTTTTTTAGTATTCATCTCATAGTATTAACATTTTAATAATCTTTACCCGTGTGTATTTTATTATAACGGTATACTATCACATTTTTTTTTAAAAATAAATAATATTAGTGTAGCATTCATCTAATGAATGATATAATAGAATGATAAATAATTTTATATTTATCATATAATAAATAAGTGATAAACAATATTGAAATTAAAGTTGGCACTCAAATGTTAGAATTTCAAACATTATTTGTAGAATACACATAAAAATAAGGTAAGCAATAATCCTTACCCTTATCATAATTCCTGATAACTTCATTGTAATTTTTTTTTTTTAAATATGAAACTCTTTGACCAAATATACATTTATTAATTATTCAAACAGTTATTGAATAAATTAACAAAAAAAAATTTCAAATTTTGAAAAAAACTATTAAAATAAAAATAAAGAGAAACCCCACGCTCAGAGCTTCCCTCGTATATAAAGAACAGAAAC

>Cs1g16270

TGGGTGTTTTTCTTTATTTAGCTTGCTACCGAGTTAAATTCATGAGGGATTGTATGAGTTAAAAAGTTATATTTGGCTTCTCCCAACCATATCCCTAAAATAATAATATTAGATTAACGATGTTACTAGTTAAAAAAAAATAATGTCACAAATGAATTTAGTTTAATCAAATTAATATTAGCCAAGTGCTAGCCATGATAGTTATTAACAACCTTAAAAGAAAAAAAAAAGGCTTAGTTTATGAATTATGATATCATCAATCACAATAACACTAATAGAAATAGATTAAAAATTATCATTACATTTATAATTGTAAATTGCAAGTACTTATATAAATTATTGTGACTTAAAAATAGTGAATAAATGTAATCATCAAAAAATACACATCGTTATGAATAAAGTAATTAATCTCACATAAATTTATATGAATTAACTTACACAAGAGATTTACGAGTATTATTTAAAAAATATCAAACATTAGATATTTTAGACTTGTCTAATATACAAATATTATAGGTCTACATATTTATAACGTATTGCACTGTCAAAAAATTGATAAAATATTTTGATAAAATTTTTATGTAAAACATCATTAACTTTATAATGTAATATTAAAAATTAATATTATTAATTTATTATTATTCTAATTAGTATAGAATTTTCATATAGTTTACATATATTTTAATATTATTCAGGAAGGAAAAAAAAGTAGTAGTATAGTTTCAAGTTAAATTGAGGGGGTCTTTCGTAGTATTCTAGTTTGAATTGATTAGTTTGATATTTTTTTTAAATGAGGGTGATTGTTTGATAATTACCTTTAATATTCTCTGTTTCACAATTGATAAGTGTGGCCGGTTACGTCCGCAATATGAAAAAAATAACATAAGGCAGCCGCATGACATGCCATGCTGCGCTGTATTACTATTGTTCGGCTCCCCTTCTATCGCTATATCTGAATATGATAATAAAAAAATATCAAAAAAGACAAAAAAGTTCTCTCC

>Cs1g21260

GTCTTTGCAACATTTGAATTAATCATCTCTGGGGAGGTCCCTTTTTTTGTAGTGTGCTATTGTTTTATACGCGTGCGTGTGATGGGTACTGTCTTTTTCCATTTTTTTCGTAAATAAATTTTGTTTATAAAAATAAAAGCTTTTGGCAAATTAAATACTACTTCCAAACCTTTCACCAAAAATAATAATAATAATAATAAATAAATAAATAAAACTACCTCGATTCGCTATCCCCTCGGTTGATTCCATCACCCAAGGCCAAGGTGAAGCTAGCAAACGCCAAACTCGAGTATATATGAATCAATCAAATAAATGATCATGGAATCATCGAAACAAATTTTGTCTTCTCTATTTTGTATTTGTCGTCTACATCGCCCATCAATCTCATTTAGTATAAAATCTAAATGTCATGATTGGCAGCACAATAACTCTACTTCTAGAGAACTGCATACGTCTGAATCATGAGAACTTTTCTGCTTAACACGGCACCAACGCTAAGTCATTCATTTTATTTCTCATATTCGCTTTGTTTTTTTGCACAAACCATTAATTATTCTCTTGACTTCCTTGCCTGCTCGATAAGAATGTACGTAATGCAAATGGCAATGCCAGACTACGTCATTTACCGGCTTCAACTTTCAATATAAACGACAAGCCGAACCACTGCCGTTCTGTGAGATAGTCGTCTTATGACTTGAAAAACGTCGTCCCTTTCTATCAGCCGCTATCAATCAGTCCCCGGCCAGGGCGCAATCGCTGCTATCCATTTCAACACTGGTCCCACTAAATCTAACGGCAGACCGTTCACTCGAAGGCCGTTACCATTTTCCACATCAAAATGAGTATCTTTGCCACTCTATAAAAAGAGGGGACGCCCCTCCAGATTCTTGGCTCCAATTTTCCAAATTCAGTTAAAACCACAGTGAACATCAATTTTATAAGTCTGTTAACTCTTTGGGGCGAAAAATATCACGAGAATGATGGCATGTTGCGGCGATTCA

>Cs1g14950

TTCTGATTTCGAGATTTTTATTTTCAGTTTGCTGTTTTAAAATTAAAACATATGTATTCAAAGTGTGAAGGGCAAGCTCATTGACTCGTGTCAGATAAAGATAACAGAGTCCAAATGACAAACAATAATTGGAAAAAAAAAAAATTGAACCCAAATAGTCAAACAAAGACAATACAAAATTGATTTCAGAATTCTATATTAGACGACTCATTTCAAACTTTTGATAAGATATATAAGACCGAGATTAAAACATGTGACTACAATCTTTTTTATTTTCTTTTCTTTGATATAATATTATACTGCACATATGACATGGAAAATCTATTCAATTTCTTAGATCAATTCCGGAATAATCAATCCTGATAAAAATTATCAAGTGCGAATGTCTAAATATAAATATGGTATTACTCTATTCATTGTAATAATTAATTGAACATAAACACTATAATAATTGACTCGAATTGTTGCCATGGACATTGGTTGATGATGATAGCATGCTCAACTTATCATAAAATATACTAGCACTATCTATCCCCATTATTTCACACGATAGGCACACATCACTTTACTTTAACTACAAATGATCGCATGAGGCTCCACTTTACTTGCACATCCAACATGTCACACGTCCCATTGGCACAAAAAGTGGCACATCCAGTGGGTGATGCGTCCAAATGTCCCATGGCCACACCAAATCAATAACTGTCCTTCTGCCGATGAAGCTGATAATATGACATCACTACGCCTACCAATCATGTAAAAATTTCGATCAAAATTCGGACCCACCACCATGCAAAATTCCAAAACCTTTGGTTCGTGAATCAGCGGTGGCCTTTGCGTCATCCTCTTGCACTGATAGCAAAATCAATTGGCAAAACCAAAAAGGTGGTGAAACACTAGGCTAAAAGCACCAAGAAGACACAGTGCCGAAACGTGGCAACATGCTACTGGTGGTTTGGAGTAGAAGAAGTTGGCCGTTTCCTCATTCACGCAACCCCCCA

>Cs1g26410

TTATGAGATGATTCAGAATGGCATGATTATTTTGGAGGTTTCCACTGTGGCCGTCCTAACCAGGTCGTTTCCCTGTTTGTGATTGAGCTTGTAATGTTGTTAAATGAATTTATTTTCTTTGCTACAGGAACAAGGCATAGAGTCAGATACAGAAACTTGTAATTATCCAAGGCTTATAATATTCGTATTCTGACATTTTGCCTGCTTGCTGCTTCTATCAGTCATTTTGGCTTAAAAAAGGGGGAAAAAAACTCGTAAATGCACAAAATTCTTGTTCGTTCTCAATATCAGCTCCTAGTGACTTGGATGAGTAGCCTAAAAAACTTTATTTGACTAGTTACTTAAGTTGGAATCCTGAGAGAAATTTCTGTTGGAGCACCTGCTATCCGGGATCAATTATTCGGATAAAGAAAAAGGATTGATTTGTTTATTCGATGGAAATAGACGTTTAAGAAAGAAAAGAAAAGAAAAAGGAAAAAGAAAAAAGAGGTGAATCGGCAGGAAATAAGTAATAAAAGTATTTGATGAACCGGCACATAGTGGTAGACGATAGACCCTACCCTAGAGTACCCTGCCCTACAGAGAGCGTAGACAATGCAAGTCGTATTAGTGCCGCACCTGACGCACCGTAAAAATACAAACCGCAGGAAAGCGAAACAAAACCAGCAATGAAATGATTGATATTTGATCGCAGCATCACACACACACACACACACACACACACACTCAATATAATTTGAAATCAGATGCGGCACGCCACATGCATTGCAAACCAAAGTTACCGTTGTACTTACACGAAGAGTCAAAGACAAAGTCATCGCCTCCCCACTCTAATCCAACGGTCACCTATCACTAGCCGTTGGTTTTGAAAATAATAATAATAAATCCAGACAAAGCATCCATTGTTTTATTTTCTCTCCTCCATTTTGTCTAAACACTTTAAATGTTCTTTTGAAGGAGCGGAGGGGCTGTGGTTTTAGAGGATTGTTTCTTCTGAACAT

>Cs1g01380

TTAATGTCCATTAAGATTAATGTACTATGATGTTTACTAATAAAAAGAGATCCAGATGTACATTTTTAAGGTAGTGGGGTTTAGCTATGTATGACTAAAACTATGGTGGTCTATGAGACAAATTTTTGGTCTCAGATTTCGTTTCCCCTCGTATTTAAGCTTTGACATATCATTATAGAAGTTATAACCTATCTTGATGCTCAAATCTTCACATATGATTTGATATTTTGGACTAAATTTCTCAAGTTGAAAATCAACGCCCAATAGCTGGCTTGGCCACAACGTGCATGCCAATCACTCTTTATAAATATATTCAAATTCTTTATCTTTAAGGGCATGTCCATTGTATTATTGGCTGTAAGCAAATTAAAGTTTTTTAATCTAACAGAACACTCTCGCTAAGACCAAATAACAATTAGCAAATCATTTCCTCTCCTTTAAATTTTCTTTCTAAAAATTTACTGATTCTCATTTATCCAAAGACCGGGTCTCTTGGTTCCCTCTGTGCACCACGCAAAGCAAACACAAAAAGTAAGAGGAAAAAAATTCAAAAAATTTCAAAATTCCATTACCTAACTAATTACAAATTTAATTGAAAAATTAGCACTCTTCTCTTTTTCTTTTTCTTTTTTTCTTTTTACCTTCAAAATAATTATGACATTTCTCATTGTCTTTTTAACCAAAAGAGAGAGAATAATTATTAAACAAAGAATCAATCTTTTTCTTCTTTAACAGCCACACTCCAGGAGGAAAGTTTTAACCAGCTTCCTTGCCTATCATCTCCTGGGGCTAAGGCCTGTTTCAAATTGATTTCCAATCCAAATCCAATCCCTTTTCTTAATTCTCAATCTTTGTGCTAATCATTTTGGGAATGCACCAATGTCTCGTCTATTTGTTTTATTAAAAATACATTGCAATTTTTCATAACAAGAGCTGCATTTTTCTCCAATTTTCTTGGGAAGCTACTTGTGCAACAAGCCTGAAGATTCAACATTAAAGCA

>Cs1g21200

TGATCATAAAATTCCCAACATTTGACCTATATAACACAATGCAAGAAATGGATTAGAATACCCTGTTGAGAAGATATTCCCAAAGTGGGGGTGGGGAGTCGGAAAAAAGGGAAAAAATGAAAAAAAACTCTATGGCAGTACTTTAATCTAATTGGGATAAGTCTCCCACAATAAGTCATGAGGAGATTTTTTTTGTCGTTAATTTTCATAAAAAATTTTAGTCAAATATCTCTATTAACGGATAATTCATAAAGAATACAAAAATTACAAAATAATATCCATAATCTATGTAATAGCACATTACAAATGTCAACTGACATGCTAATAATAATACTAAGGAGACAGTAACGGATATACATATATAACACCAACAAAAATAACTGAACGACAAAATCATATTAAAAAGGATGACTTAGATGTCTATGCGAGAAAATAAAGGATGACTTATATATTTATATATATATATATATATAAGCAGCCATGAATTTACGTAGTGAGTAGAAGAACCATTAATTTCAGCAGCTGATGGTGAATTGATATATGTATAAACAATATATGGTTCCATCAAATGAACGATCATGATCATCATCCATTCCCATCACGTAACCTAGCAGATGTTTGATGATCCTAATGATAAAAGCTAACGGAGCAGATTGGAGAGAGAGACGGAGTCAATGGCGACGGCGGATTATTAGCACACATGTACGTGTGTGTGTGTGTGTGTGTGTATTAGGATCAGTGTAGATTCTACACAAAGAAAGAAAGACAATGAGAAAAAAACTTCGTCTCCTTCTCTGGCATTTCTAATTTCCCCAATTATTTATTTATATTTATAATATTAAACATAAAGTTATCCTTTTTTTCTCAATCGACTTTTTGAAATCCTGTGACCGTTCCTTGTCTCTCCTTCTCCAGCTACTTATTCTCTCTCTCTCTCTCTCTCTCTCTTTTTGATTTCTTATATATATATATATATATAAACACACATACACAATAGAGCA

>Cs1g23080

AACACCACTTGAGATAGGAACAATAATAGATGGAGGCACATATGCACACATAGTAAATAAAGAACACAGTAAAATAGGATTAGTAAAATGGTCCGGCAATCTCACTTCGATATTTAAGTCAGCTAAAGTTTCATATCGTATACTCATTTTTCTTAAAATCTCCTAGATGTAATTTTACGTGTTTTAAAAAAATACATCTAAATAATTTTATTTAACTTACTTTAAAAAAAATTAAACTTATATTTATTTCAATCATTTCATAATAAAACGATACTATTACAATAAAATTTACTTAATACATGTAAGTTTTTAATTTTTTTTTAAATTGATTAAGCATCAGGTTACTGTATTACACAGAGATATTTACAAATGACAACATATCATTGCACTGATATTTACAATCAGATATATACCTGGGACTTACATTATTATATTTTTTATTCTATAAAGTACATGCCCAACCAAATACCTCTCATGGGAGAGAGATGTCTCTACTCCACTCGCATTTCGCAAGAGAGAAAGATATTATAAGCAATCTTCACACAATTATAATTAATTGAAAGAGTTTGAGTCCGTAGAGACTCGAACTATTACTTTCATAGTCATACTTAACTTTAAAGATAATGATTCACCACTGGGCCTAAAGCCCAAGTGGCTATGTAAGTTTTTAATTAGCAGCTTGTTTCACTGCAATCACAGTTTGGCCCTGAACAAATATTTACTCGACGTATATAAAGGGATTGTCAAAGGGCTTGTCGTATGCGTTGAATATCTAAAGCAGTGGCATAATACGGGATTGTCCAGGGGACTACGGCTACTTCAATAGATTGGACTCGTGTCAGAAATGGAAATGTGCCATTGCGAGATCATCTAACAAAACCAAAACGAGGTGCCACAACAGTGAGTGCATGGCGTCCACGTTACCTTCATACTTCCACACGTGTAACACTAGAAGAAAAGATTGAAAGCGTTCTCTCTAAATGAATTGGAAAATGGCGTTG

>Cs1g20500

ATAGATATAATTATAATACTAAATTATTTTAATAAATATTATTTAGAGTAGAGCTATTGACACCCCTCTTACATCCTATTAAAACACCGTTATTACGCGTATCCCATTAAATAATATTAAATTTTTTATCATAACTATTAACACCCTCTTTAATTCCAAAAATAACATTATTTCAATTATTCTAAATAACTCTCTCTCCTAAAATCCAAAATACAGGTATCTTGTTTCTCGTTTTCTTTCCATCAGTCTTCTCATCTTTAATTTCAATCAAATGTACATTTTAATTTAGAGTTTAAAACTAATCCTTAAGGAAATAATTGACTTATAAGAAAACTATTAAAAAAGAGAGATTATTATTTATGTTTATACACTGACAATTGGGTAATCATGATTCATGAGGTGTTGCAAATAAAAGATTTTTACTTCTGTGGCAGTTATTGTCGTGTAGACTAGGATAACCTACAAGTTTACAATTTTCAACTTTAATTTATGTGTTCCTTTCCTTCTTAATACCTTTCTTTAATAAAATTAAATTCATCAGGATTACAACAAATAATTATGAATAATAGAACAAATACATCTCAAAATTCTTAAAGCCAAACTCTTATTTTTGGATATCTATGAACCAAATTTGCAAGAGTGAATTGTAGATATCCTTACAAAAATTTGTGTGTATTTGACTTCAAAAAGTTTTTTGTTGGTTGCTAATTCTTAAGCTATTTGTATTCATCCAGTCTTGAGATTAGCAAGAGATTATATTAATACAATAACAGAATAGGATGGATGATGAATACAAAAGTTTTGGCCGAAAAAGAAAAGGTAAATTTCGCAGGAGTGGTGTAAAGAATTGGTGGAAAGAGAAATAATAATAGGTTTAATTTTTTTAAAAAATGTCCTTTAATATAATTTTATCTCAAAGGTGTTTTATTAGAACAATAAAGGGGTGCTAATGGCTCAACTCTATTATTTATTATAATTTTTAAAATTATATTCACCTAAAT

>Cs1g19860

ACAGCAGCTGTAGCTTAAATGCTACAACACTTAATCTCAAACAGGATCATAGAGTCTCTTCATATCACGTTGAGCTAGACTTTATCAAAACATTTGTCTACTTATAATTTTAATAATCTCCCTATAACATCAAAGAAAGTGCAGATTAATTCATGTTTGCTTTTCTTTGTTTTCAATGTTTGTATAGTATTTTACCCTAGCTTCATTTTTCATCAGCTAATCATTATAATTCCTCATTTGATAAGAAAATACCGCGCAAATGACTACAAGAAATAAGATTAAAACCCTCATTATTCTAAGATAGATGTTAAATCCCCTTCTATTAAGGAAATCCACCTTTTATTAAAATTAGAATGAAGTTAAATCATAGAAGCACGAGTTCTAAGTTGCTAAAGGACTGTAAACATTGTATTGAAACGTGCCTTTGCTATCTTTTAGTTTGATCAATAAAATGAGACATCTCTTATTGGAAGATTATTATTATTATTATTATTATTATTATTATTATTATTACTGCTAAATATAGTGAGAGGTTGCATACCTTAATCTAATGCGTATATTATTGTGTTAAAAAGAGTGGCTCTTGAACTGATCCTCCCTAAAAACGTGTGATGATAAATACCATTTTATGACCAAGTTAATATTGTACCAAAATTTATTTTTTTAAAAATCAAATTTCTTTCTATGCTTACTTGAACCTCATATAGGCTTTAAAAAAAAAAATCTTACATTATTTTGTTCAAAAAAAATTTTCCTAAGGGGGTTTGTGAAAGATGTATTTAGAACATGCAGCCTTTGACTAGAAGATAGAAATAATGTCACTGAAAATTAAGACAAATCATAACCAAACTCCCAAGTCAAAAAGGAACAAAGTTATAAAATAGATATACATATAATCCAAATTTGACTTTTGAAGTGACATGTGAAATCTCCAAAATTTCATAAAACTTTTAAACTATAAATTGAACAAAGTTTCTTTACTTAAGAGTTAGAGGCATCAA

>Cs1g12910

ATATGAGGATTCATAATAAGAACATATATTCAAGAATTCTCCACTATTAAACAAGTCTAAAGGGCTAGGATTTAATAAACCTGGAAAATGTTTTGGAAACATTCCAACACCAAACACAACTACCAAAAATCAACATAAAAAAACATATCACTTAAAATTAAAATGATATGTTTACCTATACAACATAACCATATAAGTATGTTTCATTATATTTTATCTAAAATCTACTGAATCAAATACAAGCAACACTTTTATCCTATTAAGTAAACATAATAGAAACCTGGAATCAATTATTAAGTAACATAACGGATGAATCAGGTTAAAGGCTGGCTTCTGGTGTAACCAAAGCTGTTGATCTGCAAAATGTAAGAGATTAAGAACTTGATAGACTCTGTCTTGGAGGCTTCTGTTCCAAAGACTTGGCTTCAGGCTACGACTTCGGAAAAAAGGTGGCCATGCTGACAGAACAAGTGATCCTATTGAAGAATGAATGAGGAGAAATCAAAGAAGTTGCTGAGATGACACCACAAGATGCAGCGGTTGAATTAGTTGTTGAGCAAATAGTGATGAAACAAAAACAAACTTTATGCCCTTAATCGTGCATAAAAATTATACTATAAGACAAAAACCCTGCACTTACCAACCCGTACTGATTAAAACCCCTCACCAACGCGTCTGCTCTCACCATCAATGCTGTCAGTGAATAAGCACATCACACCTTAATAATGACAGCTGGGTGTTATCATTTCGGTCAAAAAGGTAGACCCCCAAAAACAATGATAATAATTAACAGTCATAAATAAAAAATAATATTGGGTATGTTACCATTTTGGTCAAAAAGATGATTTCGCCAAAAAGATAATTTTGGATCTGTAATACGATTTTCGCCAAAAAGATAATTTTGCCGCTAATTATTTGGGTAGTCAGATAATAATAACAAAACATGTTATCTTTCGCCTATAACTTGAGGAGATAATAAGAGTTTTTCATTATACTCTACT

>Cs1g11100

ACTCATCTCAGGTAACTTTCAATGCCCATATCTCTTTCTCTATTAAATTCCTTCCATGTCAATTTTGCTGCACGGATTTGGTCCAGTAACGTGTTTGCAACTTGTTCTATTTTTCCAGGATAAGTAGCCATGGCAAAGCTGGTTCAAGAATAAATCTCTCAATTGATTTGAGCACCGAAGTAGATGAAAATAAATTAAATAATTAAATAACGTATTGTGTCAAAGTGACTTACTATTGTTGATTTGTATGTAAATATATTGCTCTACCAATTTCTTCTCATGTGTAATTAATTCATTTGATAAAAACCTGGTGAATTTCCAAATGTTACCTTCAAACAATTAGTGATTTGTCTTCATATTGGGTTTGCTTTTGCCTTAATAGTTGCCACTCAATTTGTCAACCTATCCTTATAATTGTTGCTGAATATTCACACACCGCATTACGTGTTTTGTGAGTGATTTTTATTTCTTTCATAAATATCTATTTATGTAATTATTCTTTTTTGAAAATGACAATTTTATTTAATATCATTTATTCTAGATAAGTAGGGTTATTGTTGTCTATTTGATGACATAAAAATTATATCTGACATAAAAATTATATCACAAAAATAATTTTATATTAACATGCTAATTATAACTGAGTAAATAGGAGAAACATTAGGAACTTTTTACAATAGTACTGTTAAAAAAATTTATAATCTGGGAAAGACTTTTTAAGAAATATAAGTTAATCCAAGAGATTATATTTTTTAATACAATTATAAATCTTTTATTTACGTGCATATAAAATTATAACAATGTCCTTGTCCAATGATCTAACCAAAAAAAAGACATAATTTTAAAAATTGTGGATAAATTCAACAGTCAATATCACTTTTGAACTCTTATCTAAAGGAAGAAAATTATTATTCTTGAATTTTTTAATTAAATTTTTATTTTTAAAAGAAAATAAAAAATCTTTTAAAAAATTCGCATCTCCAAATTGTCTTGTCATCGTC

>Cs1g04470

TGTGTACTATTTCTCAAATTAAATTGCACATATATTCTCTCTATTATGTCCTTTGTTCGTTTAATTTGTTCATAATCTTTTTATGCTTTTATTTAGATTTCTTCACCGTTAAAGTTAATGAATTCATGAGTAAATGTTAAATTTATTCCGCTTTTTTTTGCATTTTTTTAATATGCAAACTAAATCATATTGCTTTTCTATTATATGGTAATTGTCAATATATTTATAGTTTATTGTCGTAAATTTCACTGAATCTTCTCTTCTTTTATTTATTTATTTATATATTTAAGGATAGAAAGAGAAACTAGTTTCAAAACTCTTCCAGAAATGTACACAAGAGGATTATAGGTTCGTTTGTAAAGAGATATATTCAATATAATTATAAGATTACATACAGATATTATCTTACATAGAATTGGATCTGATTCTTAAATTAATTTTCCGCCGTTGGATTTGCATCGTCTGTTGCTCTCCTTTCAGCTTGCAAACCTAATATAAAAGGTTATAATTGATTTGAATAATGCTAATGGGTACCTGAGCATGCCTTTTTCAAAGAACTTTATTTGTTATATGGGTCACTCTATGGTGTTATTATAAAAGTTATAGGTTTATTGCAGGAGTTTAAACGAGTTATTTTGACTAATTAAATAAAAAAAGAATTGTAATAAGAACATATCTTATTTAAAAGAATTAGCAATCTTTTATGATATCTTATTTATTTAAACAAAAGTTGTTACCGTGATTGGATAGGATTATATAGATCTCGGAAAATTCTTAAAAATTTCGTTTCTTTATGTAAGTTAGAAAAATAGTTTCCGTACTGTATAAAGAGATACCTAATTAAAGTTGCGTGTATGAACTAATATAAAAAGAGTCGTCTCGTTGAAGGCTTGAAGCCTCTCTCACATAGGTGGGATTCAGCCATCAAGTACGATTAATTTTCCTCCCTCTATTGTTTTCGGGGTTGTACAGTGCTACGCACATACAGATACAGGAAAATA

>Cs1g02000

TAATTGCTCATGTAAAGTAACAATTAAATGCAATCACCATTACACATGCAAAGTTACTATGTACAGCTATGTAAATTTATTTGGTGTAAATTTAACCATTTGCTACACATCCATGGTTCATATGACTAAACCGGCTTCCTTTTCCCTCTTATGTGCAGTTCTTTGACAGGTACCGAAGCAGACGGGTGCCGGTGTAATTCTGCTATTAAAGGAAGTACAATGTAGCGATGAGTCCACTTGTAAAAATACTGCAGGATGAAAAGTAAAAGATTGAAAAAAAAAATCTTTAATTTTTGTTAGAGTGTTGATTGTAGAAATTGAATCTCACTTGACAGCAAACTAAACCTTCAACCAAATAGAGAAAATGGAAATGATGGAAATGATTTCAGATTTATTTTGTATTACTCTATGGTGGTTTTTCCTTTGACAGCCATTGCTGAATGGAAATAATTTTGTGTTTCAGTTACGATTTTTTTATATCATTATCAGTAATCCATTAAGACAAAATAGATAATTTCATTTTCTTTTCCTTTTGTTTTTGACAATTATTATCAATAAGATTTATAAAATTCTAATCTAAATTGTTGTTTTTTTGTTTGTTTGTTTAGCATGTAGTAACAGGAATGTCTACAAGAAGATGGATATCTAGGTGGGAAAATTTTTTTTAATCAAAAATATAAAAAATTTAGAACTATGAATCACAATTTTATTTATCACATTTTCTGGTTTTAAAAAACCTTTATCATATAAATATATATATATATATTTTTCATTTTCTTATTTTTACCATCATTCCCTCACGTTCTCGCATCACATGATGCTCTAATACCCAGTGTTCTCAATTCTTTAATTCCGTTAATACGACATCGTTTTCACCTTTCAGCTTGTGTTTCCATTTCGTTATTAAGCGGTGCCAACTGTTTACAGTGCGCCGCCGTTTAGACAAATGCCTCATGTCTCTCATTGTTTGCGCCAAATGCCCAAATCTTAACAGTCTTTCA

>Cs1g04080

AGGAAATTGCTATCTGCAATAGCTCATATAGGGATTTGTGATCGCATTGAAATGAAATAATATTTTGTTTCTGAAGTCTTTACTGAATAACCAGTCGTTGAAATTTGTGGCAGGGAATAATCTACAGTTTGTTGGATAAAAAGAAGGAGGCTACAGAACAGTTTGAGATTTATTGGAGCCTTGTACCTGATGAATTTCCACAACGAGGATTTCTCGATGACGTGGTTTTGACAGCAAAGACCAAATCTAGAGAACGGTCTCAGAAGAATTTGGATGCTGCATTCTCACACCGGAAATAAAGCACCAGCTCCTTTCTTTTCTTGCTCTTATTATTGATTTAGCTGCTTGCTTGTCATATTTTTAGGTTAGTAACTTTAGAAACAGAGAGAAAAGTTGCTAAATTATGTGATGGTAATGTACAGTGTAATTTTTGGGCAATATACAAGCTCTTTTGATACGATTGGTCTCAAAGAAATATCACAATTCACAACATTTCAGCCGCTGCTAGTTTTAGCCTTTTTCCCACTCTTTCCCCGGCGAACCCCAAATAATCAATTAAAATTCAAACAAAAATGAGCATTGGACATTGAATGGACATCAAAATCTCATGGTAGAGTAATTTAATTTGATGTTATAGTAGAGTAATGATTCAGTGGACGACAATATTTATCTTATGGTAAGTGATAATATTGGTAAGTGATAATATTATGTAGCACTATTTGATTATTTTATAGTTTTAATATTATTCACAAGAAGATATAAAACTACACCACGTGTTTAAATGTTCATCGTATGATAGGTAGATATAGAACTGCACCACGTGTTTAAATGTTCATCGTATGATAGGTATTAGTGGTCGCTAGATTATCATCATATGACAACTATATTTTAGAGAATTTTATAAAAAAAAAAAATTGAGAACTACTTATGTAATTAGCTATATCTAAAAGGACTAAAGAGGCAATTACGCTTTTATCTTAAAATAGAAAAAATAAAGTAAC

>Cs1g02450

AAACTTTATTGTCGAATGTATGAATGTATGGTAAAAAATTGATAGATTTTTTTTACAATCCAAAACTTCTAAAAACTTTCCCCGTGATTTTTGTATTTTCATTTTTACTATCAATAATACGCCTCTACAACTAGGCTCGTTCATATAAAAATAAAAATAAAAATCATAAATTATTAGCAAAAAAAACATCAATTATTCTATCCTTAAGATGTTAAGCTAAATGGTTTTCCAGCTGATGTATCACCAATTAATGACCAACATGTATTGAAAATAAATATTATTGCAATAATGCTATGCTTACTCAGTTGGAATCTAAAAACTATTACGATAATGTTATACTTACTTAGTTGGAATACTTAATTTGTATCCTAACTTTTGCAGCAAATAATGTAATACTATTTAATAATTGGCTCAAGTGATAGTTAGTTATTAAATCTTATCATTTTTCATTCAAATGATAATACTCTATTGTTAACACCTTAATTTGGGCACTTAAAGTAAGCATAGCATTTCTCAAACTATTATTCTATCACTAATGTGATGATTATCTTCCATGCTGAAGCTGCTGGAGGGTAGCCACGCCAAATTTCAGTAAGATCCCTACTTTTTTGGTTTATGCATTTATAGATGGTATAACCTGGTAAGCATTTTTGTTTGGTCGGTATAATCTCTTGCATTGTAATAAAAGGTTCCAGATTCAAATCAGTAATGTATTTCACTTTACATAAACGTAATATACATAAACAAAAAAAAAAGAAACTCAACTTGATTAATATAACAAGTCTAGTGAATCTAGAAGGGTTCTCCTAATCCTTTGAAGTCAAACATACATAAAAATATCATTTTTTCTTTTTTCAACTTTATTAAGCACCGCCAGCTTTAGAAGAAATTTGAAAACGCGGGAACCAGTATACGACAAAATGGAAGTTGTTGAGCCTTCCCGAAAATTATTTAGGAAAAATGCAAACAACTTCGGAACCTATTTGCAATTCCGAATAATT

>Cs1g10390

TCCCATTTGGGATCTGCAACCACCAGCGTTAGTATCTCACGTGAAAGATCTAGAAAGACAACTCAAGGAAATAGAGGATGGTATATATCATCTGCAATTGGAACTGGATTCAATAAAAGGACGAAGGTGAGTTATATTCCTTGTGTGTGTTTTTTGTTATTGTTCTTTTTCACTTTTGATTTGATTTTGCAGATTTGATTTATTTTGTTTTGATTCACTCTCCCATATAAATGGCGGATAACGGTACTCCGTGACTTTTTTAAGTCGGTATTCTCAGTTTCCCACAATAACTGAAACCTCAATGTCAAGTATGGAAGAAATACAACAAAATTTGCATCAACATTTTCCTTCTCTCCCTCAAAAGCTCGTTGTGAACTCCTGCATTTGTTAATGATCAATGGGATTCCTGTTGACATTCGCTGGTTAATTGAAGCAAAGGTCTGATTGGCAGGTGAGTTTTCTGATCCATTCATTTCCTACATGCCTGGTTTTGGTAAAAGTACATTTGCTAAGAAAAACGGCCAAGGCGGATGTACGGTACAGCAGAACGTGAAATTCAAAATTTATGGAAGTTGTTCCAAAAGGAAAGTGCACGGTTTAGTCTTGGGAATCTGACTCTAAAAGACCTTGTTTGCCAGTTTATAAGAAAATTGGATGAGAAGCCTATCCTCGACCCATAGAGGGCGTTCAAGCCGTTACTGGACGTAAAATCAGAGGAAGATGTGAGCCACAATCACAACTACCAGTAAATATCTTTTTATTTTACTGTTCTTTTATCTTTTGCATTATTATTATTATTGTGTGTCTTTTTTTATCTAATCACTGTTTACTTTTTCTTTTCACTGTTTGCCTTTGAATTATTAAGCTAAAAGCTTCACGCGTCATTTGACAAACACACCACCCCATTTACAGATGTATATCATTATGTCCGCCACCAAGATCCTTAAATAGGAACTTCTTTTCAAGCACTCACAAATTGTTTCTTTTTAGAATTGTTCTAA

>Cs1g03090

ACGAGAATATGTGATGAATAATGTGCTCCGGAAGATTGCCGAGTCTATTAATAGTAATTCCATTTTATCACTCCTCCTCCTCCTCCTCCTCTATTAATAGTAACAATTTATTATTATTATGAATAATAATAATAATGATTATAAAAATGATAATTATTATTTCAAAATGAGAATTAAATGATTATAAATAATCATAATAATAATGATTATTAAAGATAAAATGAGAATTTCAAAATTTAGGCGGAGGTAATATGGTCACTTTAAAGAAAGAGATTAATAATTATAAAAATGATAATTCTGATTTATAATCATTTAAGGTTAATTTAGTAATTTGTAATAATTCATTGCGATTTCTAGATATAGCAAATACATCAATGACAATACAGTTCATTCTAATTCTGATTCTTACAGTTGAAATAAACAATTTATTTTGATTCTCATTCTCTATTCCGATTCAAAATAAACAGTTTATTTTAATTCTTATTTCCTATTCCGATTCTAGCTCATTCTCATTCCCTATTTCGATTCAAAATAAACAATTTATTTTGATTATTATTTCTCATTTTGATTATACTCCACTATGATTTCGGCTCATTTCGACTATTGATTAATAAACGCACCAATAATGTTAAAGATACATTTCGGTGGGTCTCTATGAGTCTATTGTCTAGCCCCTTTTTTTTCCTTCAATTAAAGTAAGGGATTGGGAAATTCCGGCGACAGAAATTAGTATGGTGGTTGACCAAATTAATTAATTTCCATTCAAAACAAGAATTTAATTTATTTATTTTCGGGTATTTAAGTAATTTTAATTAAAAAGAATTTTACGTGGAATTTAAAGAGGAGTCAAAAGGACCCAGACCTATTTACATTCACCACTGGCTTTGAACAATGGAACTTACCGGCCCAGTTATTTCTTCTTCTAATTTCTAAAAAGTGAAATATCCAATCAAATGAAATAAAATAAACGTGCTAGCTTCTCCGATAGCACAAATTTCGCG

>Cs1g08030

AATATATTGACTTAGGACATGTCATTGATAACATGTATAATGAAATCAACAACTTTTTATTTAAAACTTAAAATTGAGTTGTAATTACCTTTTGCCCAAATTATTCACTCTAAATTGTTAATATACCCTTCGTAATGATATGATGATAAAAAATGATGAATAATTGGTAAAATTAAAATAAAATTTATCATTGGAAAGGGTGTGTTTGTAACTTAGAGTGAATAATTTACGCAAAAGGTAATTACATCTTAATTTTAAGTTTTAAATAAAAAATTGTTAATTTCATTATACCCATGACATGTCTTAAGTTAATATATTGAATTATTTATTATAGGGAAATAATAGTAATTATAAATTAAAAATAAATTTACTATTAAAATAAATAAAATTTAATAATAAAAATAAGAGTTATATGTCTTTTTTAGAATGTTAAGAAATTTTATGTCTTTTTTTTCAAACGTGAAGGCCTCATTGTCATTTTCCCTTATTTATTTATATATGAGAAATTAATAAAGTGGGGGCCAACTTCACGTGCATAGACCACACTCATGCATGACTATCAGTCAACTATCTCAGCGTCATTACTATTGTAACAGTGTTATCCTACAATTTTATTCGAACTCAGCTGATTAGTTAAATAAAAATCATTTTTAGAGCAGTATTGTCTTACACTTTTATTCAAATTCAGCTAATCAGTTAAATAAAAGTTATTTGAAAACACTTGACGATGCCTTTGGAAACTTATCAACCATTTGATGAACATCGCATCACATACCACATTATTTGTAATAAAATTTTGAGATAAAATTTAGAATATTTATTGTTGTTACACTTTCTATTTAGCCATCTCAATCTCTCTCTCTGTCATGGAAAACGACAAAGTAAGATTGACTATTTCTCTCAAAAATTTTCTCCCATTTGTAGGGCACTAGGGCCCTTCGTCTTAACTCTACATGACTTAATCCCTTGAAAATTTTTCAGAAAAGGAAAGTCGAAAAGAG

>Cs1g17020

GAGCAAGAAACACTTGTTCAACCCCAGTTTTGCAGATTTGGCGCATTGGTTCTTGCAAAGTTTCCCTTCTTGATATGTTTTCTGAATTTGGCAACAAGTTCTTGTCAGCTCTTCACAGAAAGATTTTGATCTTCTCTGGTAGTTGAGAGTCCATATGGTGTTCCAGCCATGAGAATCACCTGTTGAACTTGTTGGGAAGGCAGCAAACTTAAAGTTGAGAGCTAAGTGATAGCCACTCCTCACTGTGTATTGCCCTTTCTTATCACAATGCCACAGTAGCTTATCTTTTTGGGAAGTTCTTAGTAATGGGATTCTCTTTATAATCTCTGCATCCTCATTTGCAAATGCTGGTCAATCAAGCTGTCATTCCACAAATGATTGGGGTCAATTAGCACTGAGACTCTAGCAACTGCAGGTAAGGATGGAACAAAGTTAGGCCTAAATTATTATTGACCTAAGAATGCAATTACTTGCCATAATTGGAACATCGCCTCCTCTTCTCACTCTCCATCTTGTTCCCTTTTGAAGGACCTGTCTAGCCCACAAAATAATCCTCCACATAAAAGATGGGTTAGAGCTAAATTCATTTCCAGAAAATGAATTTGACAAAAGTATCTAACTCTCATTACTTCGGCCACCAAGAAGTCCGAATACTTTGCAACGAGAGCTTGATTGTGGTTGAAGCCGCCAACTCCAGCACATTAGTTAAGACTGCATCTGAAAAAATTCAAAACCACACAAGAGTTGAAATTCCAACTTTTTTCATTTTTTTCCCTGAATGTGGTTGAAATTCTAACTCGAACCAACAACCGAAGTTCATAACCTCATTGTTTGTGGGAAAATTTAAACAATTGCGTATTCTATATTTTCTTCGATTCATGTTGTTTGTCATGGCCCGCCAAAAGAGAGATTTTAACTGGCCTCGGTGGGACCCGCTCTCTTACGCCTACCAAACACATGATCGCAATGCCAGACACAAAATAGGATAAAAAATGTCCACC

>Cs1g10040

GGTCCATCCCGCATCCACAAATATTTGAAAGATTCAAATTCAAAAAATCTTGATCCACGTAATAAAAAATGTGGACCTAGATGTAGAAATTGAGGATTCATGTGGATCCATATTCATTTGCATTTGGAAAACTAAATATTCGGATTTGCATATTTTTTTGAAATAAATTCTCTGCACTATTTATTTTTTGTCATATGTATATTTAACCATCACAAAATTTTAACAAGTGATTTGCATCACTTTATTTCTAAACTTGTATCGATTAACTACTTTGACACCAACAAAATTTAGAATTCATTAAATAATTCAAATAAAATGATCATTTTACCCTTAAATAAATTCATAGACTATTTTATCCTATAAAATTTTAAAAAATAAAATTATAATTACAAAAATACCCCTGAATTTATTAAAACAAATAAATCTCAAAATCCAAAGTGTTAATTTTTGTAATTGATAAAATAAGCATATATTAATGCACCTATGACATATGTAATACAACTAATTAATACATAAATGACATGTGTAGTTTAGTTTTTTTTTTTTTTTGAGGAGGTGTAGTTTAGTTAAATATAGCTACCACTGACATAATATTTTAATTGTTGTACGATTGATATGAAATCTCTTATGTATTTTAAAATTTCTCTTAGTAAAAAACTTGGAAACCCCACTATTCCTTTTAGCCTCCCCCGTCCTCACCTGAGCTAGTAGCAACAAAAACAAGCATAGTCAATTGCAGTAGAATTTAGAGCAATAGAACTCAAGGAAATATCTAAAATGTATCATATAGTAAGACTAACTACCTCGAGAATTTGGTGAAGAAGGGAATTGTGATCTGGGCCCATTCCCTGAGTGCAACTAATGTAGAAATAAGGCTCGAGCCCATGAACCTTTCAACCAACACTAGGCCGTAACACATACCAAAAGCAAGAACTTGACCAAACATTATTACTATTATAATGACAGTGAAGTGACTCACTGATGCCCCGAGATTGGGACAA

>Cs1g06170

CCGCAGACATATGGATTGGATATGGATTGAGTCAAATTCGCATCCGATCTGCAGATGTATGAATTGGATTTGGATTAAGTTTAATCAATCCGTGGATTGGATTGGATTGAAAAATTATAATCTGTAAAATTATGGATCGGATATGGATTGATGTCTAATCCGTAAAATCCGATCCGCGAACACTCCTACATTTTAGGATGAGAAGAAGACAGCAAACTTGGCAGGGCCAATATTGATCCTTTCGTGCAAGTTCTTTTTGAACTTAGGTTATAATCTGTGGTTGGTTAAATGTTAGATTTAACTTCGCATATATGAACTATATAGACATCTTTGAACAGTTTTTTATTAGCCTCCTATAGAGCATTTGATAGATGATATTAGCCTAAAATGATACTTCAAGGTAAGACTTATTTACATGCTTTGGTAAGTTGTGTTTATATGAAAAATATCCGAAAACATACATCTTTTGGAAATTTTAAGGAAATTGCATTAAGATTTTTAAGAAAATATTTTCTTTATTATTATTCAAAAAATATATTATTTTTGGTCCCAAATGGTTTTCTTTTATAAGACCAAGATCAAAGATAATTTCACATTTACATGAAAATACTTTCGGCATTAGCATTAGAATTCAGAAACGTTTTTATTTAGATAAAAATATATTAAAACGCTTGAAGTGATATTATAAAATTATTTTATATCTACTTTTTTTTTTCCAACAAGGGTCTTTAAAACAAATACTTTACCATTATAGGTCACAGATAACCACATTGAAATAAATAACCTGAGGGAAATTACTACTAAGACCCCAGAAATTTTTCCCGCTTAATCTTAACGAACAAAGGAATATAACTGTCTAGAGAGAAAAATAAAGAAAGCTGCACAAAGAGAGAGAATCAAAGCAAGTCATAAAACCGAGCCTTAACTGAATCTTTCATTCAATTCCTGTAATTTCCTTTCTTTTTTTTCTTTTCTCTTAAGTTTCTTTTTTTCTCTTGAAA

>Cs1g12400

TTTAACTGGGCTTGCATTTGATTTATATAGAGGTGACGGTGGAGTTAATAAAATGGGTCAGTGTATTAATGAGATGAAGTGACAAAAAGAGAGGCCGTTGCATGGAATGAAGTGAAACTAGTTATGGCTTCACGAGAATTGGGGGGCTAGGAATTAATGCATTGGTGACGCCATCAGCATTAATGCGTTTCACGGGATTTTTGCCCATGTCGGGTTGGAGCTATCGGGTCGGTGTTGCTCGGCTGGGTTATAGTCGTGGATCAATCTTGTTTCTTATTGGGCCTTGTAATACCATTGTATTGGGCTAATGTCTCAAATGGGTACAGCTAAAAATTTTGGTTCGACTATTGACACCCTTCGTTAATCTCGCAAAACTCTTATTAAATTATAATTTTTTCCTCTCTTGTCGATCTATTTTACTTGTACATGTATATGAGCTTTTCTTTTCTCTCTCCTATCTATGCACAAACCTGAAACATTAATTTTTTTTTTTTGTTGGTACAGCAATGATAAATTATGTACTTAAATTTAAAGGACATATTAAAAGCCACTAAATATATCTTACACTAAGCTCATGCGCTAACAATTAAACAATTAAACAATGTAATAAATTATAAATTTGTTTTCTCTAAACTTCATAAATATTTGAAGCTCCTTCTATGAACATGCTCAAGGAGCAACTATTAGAGAAAGTATACATATACACATTAAGTTAGGTTTAAATCAAGTGAGACTAGTATGGAGTTTCATCTCTTTCTTTTACTTTCTTTTTTTTTTCATATTAATTATTTTTATTTTAGGGAAGTTACTATTTTTTTTTTGTTTTGCAGAAACAGAAAGTCTAAAGAGAAGAATAGAGATATCAAAATTGTAAACAAGATTTACTAATAAAAAAATTTCTGGAAGTTGAAAGATTTAAACATAAGAAGATTGGTTATTTTTTAAAATGTGTATTTTTAGTATTAAGACGAGTACAATAATTTTTATGAGAATTGTGGGAG

>Cs1g18920

GACCAGTGAATTGTGTTATAGCTTCTTTTATAGTATATTTCTTTATAAAAAAAATTAATTTTTGGTCTAGAATTCAATATGAATATCAAAATATTTGAAAAAAATTTATGTCTACTTGTTCTTCATAATTTTATCGGAGTTAAAAGTTTTGTATAGTATTTTTTTTTAAAAAAAAGAATCAATAATGATATAACCACGAATAAATGGACATGCTGAGTTGTAACTTATGCGTTATGAAGAAATTAGTTAGATGAAAGCATCATAAGGCCCAAACATAAGATCGAAATTCTCTCGTGATATCTTTTATTTGAACAAATACACTTACATAATTGGTAGTTAATGAATGAGAATATAATAATTAAGTAAATATTTATTATATATAAATTAAAGAGTATATGTATGAAAAGATTTTTCAGATCTTTTATCCTCAAACGTAATTGTATAACGTGACACCTTAATGATATAAGCAGTTGAGATTATAAAAAAAATGTAATAGTTGGAAGTAGCGGCGGAGCAAGCAAGTTTATTAGAGAGGGGCAACTCCAAATAACAGAATATTAAAAAAAAAAATATATTTTTTCATGGAATGTGGTCCATGTGGAGCATACTTCACATAATTCACATACAAACATAAGTAGTTAAATACAATTCTTGTAGATACATTAAGCTAATCGTTAAAAGAATTTTTGAAATGATGAAACTTATTTGATAAATTTTAAGTAGTTTTTTTCTCTGAAGGTGGTGCAGCTAAATATTTAAAAATGAAAAATGAGTGTACTATGGAGGCCGCTTAACTAACTTTTGATTGGAGGTATCTACAAAGAAAAATAGGAATTCTTGGAGGCCTCCGCCCTTAGCCCGATGATATGAATAACTCCTCAATGCATGAGACAATATGGTGTTAAATTAACCAAAACTCATCCCCGTGTTTGAATTTCTAAATATTAGCTTGAGTAAAGGAAAGAAAAACCCTGCTGGGTTTTTGCTGGCTTCAGCTGTGA

>Cs1g06310

TAAAAATACAGAATATCTCAGGTTAGGAGAGAATTCTAATCCTTTATTTAATATGAGTAATGATAGACATTATAATTTTTTTATCCCAAATTTCATCCCAAATAGTATGGTATTCAATAGTGTCATTAATTGAGTGGTTGATAATTCTTTACAATATCCAAGTGAATTAATTGTATTATCTCACCAACTACTCAATTAATGACACATCATTTGGGATATTTTTTAGGATAAAAAATTTGAGATGTGTAGCATTACTATTTTTTATAAGATTTCAGTGAATTAATTGTATTATTCTCACCAATCAATTGATGAATACCACGGTGTTTGAGATGGCGTTTGAAGATAAAAAATTTAGGATATTTAAAAGTGAAGTTCAATCTAAAAACCTATAAAAAATAAAAAGAATAAGAAAAATATATCTTATAAATAAAAAAGACTCGACCTCTCAACTTAAGAAATAGACGAAATCAACGTGCTTATTACATAAAAGTAATTTCCTGTGCTTTAGAACTAATTAACAGGTCAACAATTCAACATACGTTTCATATTCCTAATAAACAACAATGAGATTTCTAGCACTTAGCCGTTTCGACCTCAACCTCTGTCATTTTCCCTCACATTTGCATGGACCATTACAAACTGGACAAAAATTTACTCGTCTGATTGCATTTGGCCCGCCAACACATGGCAAGATCTCATTGGAGTACCACAAAGGGCGCGCCACTTGTACATCCCTATAGATAAAATTGGATCATATATACATAAAACACTTTGTAATTTTCCTCGTGCATGTAGCTCTATAATAATTCATGTACTAATGCAGCCATTGATCAATGCAATTTGATCAATCAAAAGTGTCCATAATTGTAATCAGAATCAACTTGCCCATAGGCATGACCCCACAGCATCTGTTGTTGCTGGCCACATACACAGAATCCAATACGTGTTTAATTGACCCACTACCACCACCAAGCTTTTATTCTCCAAGCAAACTCAATATA

>Cs1g22540

GTTTAAAGGCTGAACTAGTCAATTTAGTTCAACTAATTTTAGATGAACCTTTCCACGAAAAATGAAATTCGTAACAACCTCAAAATTTGTGCTAGAGAGAGAAAGAGCAACAATCATCGAATGTTGACAAAAGAACACAAATCAAATTATTTCTCCACATTATAAGATTAAGAGGAAATGTAATTTTTTTTTTAATTCTTATTTCATGCTCATTTCTAATTTTATTATGCATTGTATATTTAAATGTATAATTATTTATTAATACTTACACACAAGAGGAATATTATTAAAAGAGTAAAAGAAATCTTAGACACCTTAGAAGACCAAGTAATAGAAGTTTGAAACAACTACAACTGCAACGACCTTATCCATATAAATACGAAATGTCAGCCTGTGCCAAAAGAGAAATTGACCAAAACACGTCAAATTTTGCAATTTGACCACAAATTGCATTAGCCGTAAAGACAAATATAATTATCTTTTACCCCCACATCGGAGAAAAAATTAGCTTCATTTAATAATAATAATCTCATTTTACCCCATATCGGAGAAAAATTAGCTTCCTCAATTTTAATGACCTTCATTAAAAAAAAAAAAAAAGAGAGAAAATATCGTAATACAATTTTAGCATAATAATAATAATAATAATCTCATTTTACTCATATCCGAATATTCTCAAAATAATAAACAAAAGGCGTGGTTTATTTAAAAAAAGAAAAAAAAAATATCTGCAGGGATCCACTCCACCCAACAATATCAAAGGAAGGAAGGAAGGAATGAACCACGTTTCATATAACTGTGATCCCCCCAGTTTCCGTCCAAAGTCTGAAAACGAGTCCTAAGCAGGAAAATATCCTCGCCTTTCACGTGCATTAACTGTAGAGAGCTAGCAAATATTGAATCAAATACCAAAATTAAAAACTACTAATAAGTAGTAACTTTATTAAAAAAATATTAAATATTTTTTAATATATTAAAAAAAAAAAAAAAGAAGGAATCTC

>Cs1g02830

CAAAAACGAAGAAAAAAATTCGATTTCATCCCGTATTTAGTACAGTGGCGAAGAATCAAATTATAACTATATTTATACCTTTTTCTACTTCGTCTTCCAAGTGCAGGATAACCCCAATGGGTTGTGGGTCTTTTTTCTACCAAACAGAGTCCTTCCTTCACCACACCCATGGGGATGGTCTACAGGGTTCATAACTACTCTTACTACTAGACGCTTCCCTAGCCAACATTTAGATCTGGGTTTACCCAAACTTTTCTGGTTTAATCTGCTTTCTATGCATTTGGAGGTGTGTGAATCAAATTGTAACCTGTAGTGCCTACTTTGAATTTTTATTTTTGTTATTATTATTATTTTATAGATTTTTTGCATGAATGGATACCAAACCTTTCTGCATGGACCCCACCCATGTTAAACCCTGGACTCTTGTACCACCCAGGAGAGTTTAACTAACTGAACCAACTAGCACCCACAGGTGGTTGCCTTGTGCTGAATATATGTTTATGCATGAAATAGACTTCCTAATGCTGAGCTTTTAATTTTCTTCAGACCTCCCTCTCATGTTTTTTCTTGTTGTCTCTTATAAGGTTTGTAGAAGATGTAATTTGTTATCTAGACAGGAGTATGAACAATTTATCACTTATCAATGTTATTTTTTGTGTTTTTAATTAACAATGGCTAATTTCATTCTTGCCATAACTTATGGTTTTCATATAAAGATTCACTTCTGTATTTGCTGTTGTATTGCCATTTATCCCTTTTTAGGAAATACAACTTCTGGTTACTTTTTAAATCAAGATGATGGATGAATTGTGTATTTCTTTGTTTTTTAACCTGAAATGGTAAGAGTAAACATTGTACCAGTCGAGTTTATGAACTTACAGTGTTTAAAGTACTTTTACGATCCTTTTAGTAGGATACATAGACTTTTCTTTAACGGTATTTTGTTGTGTTTTTGTTATAGACTTTCTTTAATGGTACCTTTTTTGTGTTTTTATTATATC

>Cs1g16890

ATGATTTTTCCCTCTCTTCTCAATCTATATAATTGGTTTTCGCACAAACTTAAAATATTAATCTTTTTTTCTTTCTTTTTCATATTGATTATTTTTATTTTTGGGAAGTTTCTATTTTTTTAAGTTTTATTAAGAGAGAAAGTATAAAGAGATTAACAAAGATATCAAGATTGTAAAAAATTAAGTGTGAGAAAGTTACAAAAAAATAAAAGCTTTACGAATGGAAAAAGTGTTTACTAGGAAAAAAAAATTTTCCACATTACTGAAGTTTAATTGGAAAAAAGGTTTTATAAGAATTTTGCAGAGGTCGCCATAGACGTTCAATCCTTAATTCTCTTTTAAAATGAATTTCTCTTGACTAAAATTTAAGTGAGAAATTGACAACTAAGAATATAAAAATTATAAATAAATTAGTCTTAAGATTATCACTATTTAAACAAGTGGCATAATATTTTATTCGGGATGTCTAAGCATTATTATTTTATTTTTATTTTTATTTTTATTTTTGGGCGCTTTATTAGCATGGATTTGTTCATTTACGTGTGAACTAATTAACTCAAAGTCGAAAGAACCAGACGTCTCAGCCTCTAAAACTTGTCTGCACATGCCAACTTCGTTTCTCCGATATTTTGTGATTGCCCGTTAGAGTCATCAAGTCATTATCTATTTGGTTAACAAGAACCTCCGGAGGTTCTGTTGCTTTTATTTCAATTCTTGTTATCAATTTGTTCAAGAGAGGTTTTGTTTAATTTCTTTCAATGGCAGCTTCTTGTCTTCCTTGTACATGGCTTCCTGCTTTCGAGCTAGACCATGCTTTAGAGCCCCTTCTGTTGCTGTCTTCGAACAGACGTATGAAGCTCATATCGCCATCAAGTTGCAACAACAGCAACATTACTAGATTTGGTATTTGTAATGGCTTTTTAAGAGAAAGCAATATTAATATAATGAATGGCCGCCGTCTTTGGCCTTCTTTAATTATATTAATGCTTTCTCACTCTTTC

>Cs1g09810

AAGGCATGCCACTTTTCCCTTTTCAATTCCAGCACTACTAAACCTTAGCTCAACAACTTAACAAACTTCATCACTTAACAAAACGAAATCACTCTCAATAAAGTAACAAATTCCATCAAACAAGCATTGGATAATTGAAGATATAATTATTAATTTGGAGAGCTTCTTAAGGTTAGTTTATACTCTTACCATAACCTAAACTTTTAATCTATTGATTTTATCATTTGAGTTCGAAATTGAAGTGGGTTTTTATTGCAAACGTTGTTATTTTTCATTAACAAAACAATGTGACTTGTTAAAGTAGTTAGTTTGAGTTGAGAAATGAAGATCAATCATTGAAACCCCTGAAAAATCGAGCAAAACAGAAGCCCACAAAAAGTGAGACAGGCGTCTGCCTCACCAAAATATGTGCAACAGGCAAGTGGGACAGGCGCCTGTCGCACATATAAACCCACTGGTTTTATGTGAGACAGGCACCTGTCTCACTTGTTGCCACTGGTTTTATACGAGACAGGCTCCTGTCTCACTTGTTTCCACTGGTTTTATTTCTCACTTGTTCCAATTGATTTTATGTGAGACAGACACCTGTCCCACTGCCTGTCTCACTAAATTTCAACTATTTTTTGAAAGTCATAACTTGGGCTATATGTGTCCGTTTTAAGCGTATAAAATATCGTTTCGAAGCTTACAATGAGACGAATCAAATCCAAAATTGTATATTTCATTTCATCCACATAAATGTACTGCATATTCACTTTACTAGGTTTTGTAGCGTGTAATCTTATCTCTTTTGTTTTTTTTATAATTTTTAGGTTTAATGGATTCCATTGTACTTCAATTATGTTACAACGGTTGGTGGGAGACGTTAGCAGATGGTCGTACAGAGTACGTGAATGCGAAGAATGCAACATTTTTAGTTTGGAAAGATTGTACATTTGAGCAATTTTTGGCAAGGGTGTATGAAGTTTTACAGATAAATCCTAATTAATATAGTTTGACGA

>Cs1g13036

AATATAAAAAGCTATAATTGTCCAATCTCAATCAGAACCTGAGCAATTTAGCAAAAATATACTTTTATTCCATATTTTACTAATATCCTCACCATTATTCAGAGCACCAACCTTTAACTTTCTTCGATTCTGTTTGGTATAACTGTTTAAGTAGAGCTTTTACTAAAAAATAATGGTTTGGTTATCAATGAGAATTTTTATTAAAATTTATAAAATTACTTTAATAGGTAAATTTCTTAAAGTTATTTTATGAAATGAATAAAATAAAATTGTGAAATATATAAAGCATATTTTAGACAATATAATATTTAAAAAAACTTAACCTCTACTTTAAAAAATTTAAAATTTAAACTTTTACTAGTATAGATAAAATAACTTATTTAATTTTTAAAAATTTTACTCAAATTATATAAAAAATATTAAGTTTAAAAAAAGATTATAAGATTAAATAAATAAATTTGACCGAATATACACTAACTCTTAAAAAATAAATATTTTGCGCCACAGAGGTAAGGTCAATAATGTTGCAACTGAGGTATTGTACCATATATATATATATATATTGGTATTTAACAAAGTTCTTGGGTGCTTCTCTGTTCAATCCAGCGGCTGAAATTAATTATTGGATAATTGATGCAGCGACTGACAACCGTTGCAGCCAGTTGTATATTAATTTAAGAGTAATGATACAGCCACAAACTCTTGTACAAACTTATTTTGTACAAACTGACGTGGCATTAATTCATTGGTTGAATGAAAATATAAATTAATAAAAACAAATCATGTGGGCCAAGTGATATTTAATTCAATCAATCTTATCATGCCACATCAATTTATACAAAATAAATTTGTACAAAAATTTGTGGCTATATCATCACTCTTAATTTAATAGCAGTTCAGCAAGCGTAGGTTCCGAAAATTTCTTAGTCCTGATTAGGGTTTCACTAATTCCACCTGAAAATTGATCTCGCGACAGCAATTAAGACATAAAATTAGAATTA

>Cs1g13090

AATTTCAGATTGCTAATTGCTATGCTTTCACCCTTTTAACTGTGGACTTGTCTTTGGTTATTAATTATCAGTAAAATAGCGAAATAAAACATGTCTGATTCTCAGCTTCCTTAGTGCATATAACATATGCGTTTTTTTTAAAAAAAAAAAAAAAACACGAAAGGAACCTTGTAATTAAAGTCTGGGTCTATTAGCTGGTAAAATATTTTGAGTTGATACAACAAGTGTTTGATATATGATTTTATTTTTTATTTAATGATAGATCGTCATTGTAGATAGCTTGTTGTGTAACGTTTTTGAAATGCAATCGTACAATGTATTATGTTATTTTATTGCTTCACTACTCTTTATCCCGTGCAATGCATCAAACATGCTCTATGAATACTATATTTAGATCTTGAGTATCACATATTTGGTCAATGGAACAAGTGAGCATAAACAATTTTAGGATGCTAATATTTTACAATAAGATATATAAGGTTTGTTTAAGTGTCACTATCAATCTAAGTTGACGTAAACTTATTTATTTTATCACCATCAACTTGATCTGAATCGAATCATGCTGTTACTTCAACTTGGCAAATAAAAATTCTATCTTAGTTGTTTCAAAATATCTAACTTGTTTTACCCTTGTTATCTTCTTAGAGAGAATCTGAAGGGAGTCTAAATTAACTTGATGTGAATCATTGGAGGTGTTAGTTGATATTATTATCCTTCATTTGAGGCCAAGGTCTAACTCCTATGATAAGGAACGACTTTCCTAGAACTCGGGTTCTGGGTTCGAGCCTCACTAATGGGCTTGTTTAATTTAAATAAAAAAATTACCCGCCATTTTATTGCACTAATAATATCATAATATTTTATTGTACTAATAATATCATAATATTCTTTCAACTTATTGCCTCTTCTTAAATTAGCTATAATTCATTTCTTCCAAAACCTCACAAGCTCAACTTTTCATTGTTAAGTTATTCCTTCTTCTTTGATATCTCGCAGAAATT

>Cs1g18380

TGTTTGAGTAATGATTTTGTATTTTTAGTTGAATTTTGAACAATAAGTTTGTCTTTTTAATTGAGTTTTTGTCCTTAAAATAAAAAAATAAATTTAATTACACACTTAAATAAATTAATATTTGTTATTCCACCCCAATAGTTTAAATAGCCATTAGTACACATCCATACTATTGAGATATTTCATTAATTTAATTAAGAGAAGGGTAAATTTTTTTTAAATAATTACTCTATTTTAAATATTTATGTAATTATCTTTTAATTAAAATAAATTTAGCCAAATTTATGTACTAGCTAAATTTAGCCACATGCATGCACATGCTCAACAAGTACGGATTAATATATCTACAAAATATTAAACAGCTGATTCTTGTCGGCCACTAACTAAATTTAGCCACATGCATGCACATGCTCAACAAGTACTGATCATATTATCTACGAAATAGGAACAGCTCATTATTGAAGTATTGTGACTTGCAATATTTGGTTCATGCATTCTACTTTTTGGGTATTTCTTAATTTTAAACAAATCATAATATATAATGATGGTTTTAAACAAATCATAATATAGCCACATTCATCTTTCTGAAATCATGATCTCGTGAGATTTTGGAGGTTTTAGTATTTGATTTTGTAAGCACTTACGCAAGAAGTAGTGTCTGGTTAATTTAATTCTACACTTTAAATTAATAATAGTTCTTGCATTAATTACTGATTAACCATATGATTTTTTTTTTTTTTTTGTTGCAATGAGATAATCACTTTCTAGAAGAAGAGAAGAGACTTTGTCATTATAATTTTGTTCTATATGATTTGGTGATTACTTTTCCAGTATCCACTAATTATAAATAGGAAAAATAATCAAAACAGTCGATTTCAATGGACTTGGAAAAATTTGAAAGACATGACAAAAGAGGGGAAAAAGAATTAATATAAATTCATACTGATCCAAAACTTGGTTTTATCAAAAGATTAAATGCTAATATACTGCCATTTTGCAAT

>Cs1g06915

TAGGTTTATTGGGCCTTTTGAGATTCTGGAGAAAATTGGCACAATGGCTTTGCCACCGAGCCTTTCAAGGCTTGACGATGTGTTTCATGTGTCGGTGTTGAGGAAGTACATTGCTGATCCTTCTCATATCTTGGATTACCAACCTATTCGGATCTCGAAAGATATGTCATACAAGGAGCAATCGATGTAGATTTTGGACAAGAAGGAACAAGCTTTAAGAAAAAGAGTTATACCTTTAGCAAAAGTTTATTGGGCAAATCATTCTGTGGAAGAAGCTACTTGGGAACCAAGCAGAGATTAAAGAGAAGTACCCTCAGCTTTTTCAAGAATAAGATAAGGTTTTAAATCTCGAGTACGAAATTTCTCTTAAGGAGGGGAGAAGTTGTAAAACCTAAAATTTGTTTCATTTTTTTTTATATATACATATTTTATAGAATAGAGTATTTTATACTTATAATATGTAAGTGAATGATTAAAGTTTCATTTCGATTATTTTTCTTTTATATGTATTTTATAGAATATATTATTTTATATTAGTAAATATGTTGAATGATTAAAGTTTGGTTAATTGGTGGAAGTGGTGGGGTGTGTGAGCTCCATAAACAAAACAGTGCGGGCCTTGTTAAGGCCTAAAGCGAATAATATCTTGTCAGGTTTGTGTTGGGTCATGACAGTATTTTTTTAACCTGCATATGGTTGATGTCTTGTAAACTCGAGCAAGTCAAAATTGGCATGCAAATGTTGTTAATGAGCCTCAAGGCAATGCGGTTGCATGAAACCCAAGTTTGCTTTGTCGAGTTTCCTAGGTTTCCTTCCGCTATATAAAAATATAGCCAGCTAATTAAAAATAAAGTTAATATTATTAGCTGGGAACACAAACTGTCATGCAATGCATTGGCAGGCTCAATGCTCTTACTGACTGAGGGCAGGCTTTAGATATTTCTCTCAATGCTCAATGTTGAGACTGCTTATCACTGACTTTTCTCCTTCACATCTTGTAG

>Cs1g20620

GAACTAATATCGAAACGTAAGGAGTAACCTCAATACTAAAACATCGACTTTAGGGAGCTAATAATGGTTTTGTGAATAATATGTTTTGCATTAATCAATAATATTCACATTTTGAATGTTAAAATTGTTAATCAATATATTTGACCAATTGACTGCCCCCAAAAAGTTCGGCACCCGCAATTCGTTTTTTTTTTTTTTTTTTTTTTCTCTCCCTCTTTGTTCTGGTAATTAAGCATTTAGCAATAAGCACCCGCCAGTTAGGATGTTTCTACCGTACTATCGAATCTCGTAGGACTAAAATTTAATTTTTCTTTTATTTCAATGTGCCACTCGAGTGAAAATTATCCAAATATTTGAAGAAATTAAAATTTTAATCAGTTGTCTTGAACGTCACGTGATACTTTTCCCATGATAAAATTACCCAAATAACTTGGTCCAGAAGACTGACGTATCTCTCTGCTCTCGCAAAGAAAATTAAACCGCGAAACAAGAAAAGCAAAGGCAGTACCTTTGACATTATATTCTGCAATGAAGAAAAGAAATTTCCATATTTAAGAAATAGTCAAAAACGTTTAAAAAATTGAAAAAAAATTGAAAGAAGTTGGCAAGGAGCCGTTCTCTTCTTAGATGAAAGTTATATAAAGGCCATTCATTAGCCATTACAATATGCACGTTTTTGAAGCAATTAAGGTTCCTAGCAAGCACCATTATTTGGTAACGTTCACCAAGTAGAGCTAGCTAGACAACATCTTCTCTGTTACCTTCTTCATTTGCATTTACAAGGGTATGTCCATTTTCTCAATTACCATCATACTAAGAATTTGTTTTTTTTTTTCCTTTTATGGGGTTCTATAATTAATTACTTGCCACCTTGGCTTACCATTTTCTGCTTCTGAAAGTATATTATAAACGACTCCAAATTGCTATGTTTAAAGAGTGTTCTGTTCACGTTTGGTATGCATGTTGTTGTTTTCAGGCCTTCGTTCATTAGAAAATAGTTA

>Cs1g20760

ATCATGTTCAATGTAGTTGTACTACATCAATAAATTCATACATGATTAAGACAAATCATTCAATGAATTTATTACAGTCTATACCTAAATAAAGTGCCCAACTTTATTTATCAACTGCGAACTAAATTTATTTAATCATAAGATAACTTGTATTTATGTCTTCTGTGAATCCACATGGTGATCACATAAATACATATAATATGATTAAATGGACTTTAATAAAAATATTAATGTAATTAAGATATTTGAATAAAATACCTCATTAATTTTATTAATCAGAAAAAAAATTTATTACAGTTAAATAAACACATGCTTTTAAAGAGCATATTTTCCCAACACTAAGAAGTAATTATTAATTATATTTACCTATGCGATCTATGAGCATGCAACTGATGAAGGTGATTAGTGATTGCTTCTGATTAATCCCTTTTCAACTTCTTGAAAAATTAACATAAATAACTGCAGAAAAGTGGGCTGCCGGTCGCTGGGAACAAACGTTAAATTCGAGTTATATTTATAGAAATTTTTACTTTCTATCAAATCATGTTTCACTGTATTCATTAAACTTAATTAACCTGTAATTTATATGATCCATGCAACTTTGGAGTCTTGTTTATTAAACGTGACCAAACATGGATAAATTTGAATAAAACGTATGAATTGGAGTATTACTAAGAGAAGCTAAAAGCCACATTCATGATGTTGCATGCATGGTAACTGACAAGAAGAAAGACGAAAGTGGTCGAGGATGAAGGATTGCTCCGCCTTAATATTTTAAGTTTTTTTATAACGCTGATGATGACTACTTTCCACTACCGAAACAAAAATTAATAACACTATTAATAGCAATATTTTAATTAACATGTAAATTGATTATCAACTTTTTTGTATTATTCTAATTCCAACATCGCTCTCTCATCCTCCCACACTATACGCTCCGGTAATTTCCTAGCTTTTGCAAGTCAAACTCAAGTTTTCAAACAAAACCTTTCTGTTTTCTT

>Cs1g17470

AACTAATTTGTGCACTCAAATAAGAAACACAGTGATCAAATATTTTTAGGTGCGATAAAAAAATTAAATTCAGAGTAAAAGACATCCAGCTCTTTGAAGGTAAACATACGAATAACTAAATCCCGTCGTTTTAAAAATAGACAATTAGGTGTGTTTCATATAATCGACGTTCAAAACTCCGATAAATTATATATGTTTGGACAGGGTTAACGTAAGGGCATTAATATGCTATTGTCCTTCAAGTTTAATATACGAATCTTTATCTCTATCCATGTAGTTGAAATTTATGAACGAAAGGAGAGGTGGTTGCACGTTTTTAATATTGGGAATTTAGCTCACCATTTGGGCAAAGCCCTAACGGGTCCGACCGTCCGAGAGTCATTTTCTCTTGACTTTTCGTCGGCAGCCAGCACATGATGACAACAAATGCTACATCAAAATTCGGTTCGGGCATCCTACCGGCAGAGTAGTTTGTTTTTGTTGAATTGGGCTTCGGCTTGTGGTCCATCGTCTCTTTCACTCGATTTTATTTGGACTTGGCACTCATTAGACTAGTTTACCCATTCTTCATTTGGGCTTGGGCTTAGGTTCAATCCTAGTGGACTTCTAATGAGATATTAATCCCATAATACCTTAATAAAATGTTGTAATTCAACAACGATTGGTAAAGGCGTCCTGGTTATTGACGGACACGATGGTTTGTTTACTGTCTACTGAACATATTTTCAAAAGGAAAAAAAAAAAAAAAAACTTATTTTCACTCTATGATTCAGTGATAAACGTAGGCCCACTCAATCACACATCACAATCTCTGATGATTTCTTCTACTCAACAGATAAGATCTTGCCACGTGGGTGAATCCACCTTTTAATTTATGGAATCAAGAGCGTCCAATCACAGCCAATCGGTGCCTGAAAGTCTGTTTTCAACATTGAAAGGGTATTTTAAGCAATTTACTCCTGGTTTAAAAATCCCCTCTTGAACTCACTTCTTCCATTTTC

>Cs1g23900

TATCACTTTCTCCTGCAACTTCAGTTTGGAAAACAAGTTCATAAACAACATTCTTACGAAATCATTCTTCCCATAATCTATTCGAATAATAATCAATTTTCTACTGGAATCTCAATTCGGTGAAAATTTTCATAAATAAATTCCTTTCGAAATCATTAATGCTCGTAGATTTGAACAAAGATGATAGCGTTTTGCTTGAGTGAGGCTTTGAGCTTTCACATTTTAGGGTTTTGTCGATTGAGAGTGCATGAGGCCATGAGTAACGATGACTAGTGTGTTATGTGCTGAGAGTAAACACTTTGTAAAATTTTAAGTATTTTAGAGAAACTAACCCAAAACGATGTGCTTTTTTTTTTTTTCATTTTTTTTATCTGTTAAAATTTTAAAATTCGGTCAAGCATAGTTAAAAATTAATTTCTATCACAAAGTTTATGATAAAGAAATTTTCTATCACCACAATATTATACATATTTTATAGACAATAATTCTATCATAATTCTATTGTTAATTTTTTGATAGACCATAATTTTATCACGAGTCCATCATATTTTTATGATATAAGTGAATTTGTCATAAAGTATATGTCATTTATTATCAGTTTTCTTGTAATAGCACATGTCATAATATCATTTGTTTGTTATATAGCTAATATCATATCTCTTATGTATTTTAAAATTTTTCAAATAGTATATAAAATGTTAGAAATTTGAGATATAATCTATTCATATCAAGGAAAGTGTATTATATTATTCATCCTATGAAAAATCATACTAAGAAATATTAACTGGTACCTTAAAATAGAAACTTATAAAAAAAAAATCAAATCAAATCTTAGGTGTATATAAATCATGCCCACAAGACACCTAAAAAGAAGGAGAGCCCTTAGGCAACCATCAATCATTCTCTAACAAATTTGCATTTTTGTTTTTAACTCTTCATCGTCGATCTTTAACTTCTTGAATCTTTCTCTTCTTCTGCAAATCTTTTAGAATTTACGTGCA

>Cs1g21550

TTCTTCGGCAAGAGCCTGTTCCTGGGACGGTGTGAGTGGTGCTTGGGACTGGATTTTGGCTTGCTTCCGATTGTGTTTCTGGATTTCCTTTTTGCGGATGCTCCTGAGGATTTTGGGCGTCGGAAATACGCCGTGTTTGAGGAGTTTCTGTTTGATCTTTTCCATGTTGGGTTCGAATTGTGCTTGAGCCGGAGCCGGCAGTTGAAGAGGAGCTGGCTGTGCTTCCATGTCCACCAACCAACTACTGAAATCGTTGTTATTAGTTAGTATGTTTGGGCTTGGCAAGCAAAGCTGAGTACACTATCCGCTTAGAGGTTTTGGCTAATATTTTACGTGTGCCGCTGAGATTTTGGCTCAATGCCGAAGATGCACACAGTATTTCAGAATTAGTCTCAAGTACCCTCCCGTGATGTCATCATCAAATATTGCATAAGATATTTGTCCAAAAAAAAAATATATATTGCATAAGATAATGTTATTTTAGTTGTCTTTGCATGGGCGAAGTTGTCTCACCCTATTGGCACAACTCATTTATTCAAAGCTTTCTAATCATTGTTGAGATTTTTTTTTCTAAGTTAAATTGAATATGAGTAGATTTTTATCATAATAATATAGGTAGATATTATGTGGGATAGAAATTTGAAATGTACTAAACTGAATTTATTAAAGAAAATAATCTTAATACTTTTTATTATGAACATTAATAATATTTTAAGTGTAATATATTAATGTATGTGTGTGTGTATATAAAAGACTAATAATCTTCACGCTCACAATTTCACAATATGGGTTTGTTCGTAAGATTTTATATATATATATATTCAAGAAATCAGATTTGAATTTCAAATAAAGAACTCCTGAAGAGTAATTTACAGCCCTTTCAAAAGAGTTCACTTTTCATTTTTAGTTACTTTTTTAAAGGATCTACTCACTTGTAATAAGAGTAACTTATGGCCCATAAAAAAGAAAAAACCCCGCGGGCCGTAACTTAAAATTCTCAC

>Cs1g18420

ATTTGAGGAACTTTATTGCAAAAAATTTATTGTGAATGTAATAAATATTACTTTTTATTTTATTTAGATTATGTATAAGTATGAATTTTTTATCATAATTTAGGTAATAGATTATGAAGTTCTTTAGTTTTTTTAATTGAGTTTTTTTAATGTAATTTCATATATTTAAGAAAAAAAGATATGTCTAAACAATTTTTTAAACATACTATGAAAATAACAAGTAATTAAAAATTTATATCTATTTTGGTCATTATATAATAAAACAACACTTTCGTAATAAAATTTATTAAACACATGTACTTTACTTTTTAAACTCACAACAACCGTAACAACATAGTTTACCAAATACCAAGTTACTTTTTTTAATCAATAGCTTATTTCATCTATACAGCATAATCAACTTATTTCATCGGCCTCCACAATCTCAAACTGGCCCTAAGTCTAACAATAATTAAAATTTTTTTTTTAACAAAAACATTAAGGGATTTTTAATCAATTTGCTACTTTTATAGTTACGACAAGTCAGGGTTATTCACAATTCATACTTAAAACTATTATTTAACAATTTAAAGACATAACAATAATATTTTTATCTACCATTTGATATCTTTATTTGTGTATTTTTACTATAAAGGATCAAATTAATATTTTACTAATCAACTAATTAATCAACTAATAGTTTTAATTGGAAAGTTATAAAATAAAAGATTTTATAAGGTTATGGTAGTAAAATATACTTTTTACAGTCATACAATATTTTACAATTCTTTAAGTAGTGAAACAATGAGAATCTTTTAATAATCATACGTTTATCAAATTTATAAACTAAAAAAAAAAAAAAACTTATATGAAAACTTATCTTTATCAAATTTTCCATTTGATAAAGAAAATTTACAAGAACAAAGAAAACTTACAAGAATAAATTTTCAGAACTAAACAACAAATTATCTTTTTCTCAAAGTAATATCCTCATTCCAGCTAATTTTAGTTATATACATACA

>Cs1g15680

TTAATTAAACTCTATTGTAACATTTTATCATTTTCCGTAATTTTCATTAAAAAAGAGCTATGCATAGTTAGGAAGCACGGGTTTCAAAAGAATTTAAAAAGGGTGTTTAAAGCTTTTCTGTGGATTAAGGAATCGGCCCAAAACAAAAAGGCCATTACGCAAAACGACGAAAAAGCTGCTGAAGTAAAATATAAAATCGCCCACCGTGGGGCTCGAACCCACGACCACAAGGTTAAGAGCCTTGCGCTCTACCGACTGAGCTAGACGGGCACCAGTTGAAATGCTTACACTAAGAAAAATCTTATCGTTACTATTGCTTTAAATTTCTCTATTGTTTTTACATTGAGTGGTCAAAGTAAACTCAACCGACTGAGTCCGATCCAAGGGTTCAGATAAACTCAACCGCACCGCAAAGGCACAAACTCGCATGTACCATGAAACCACTCACTGCATTGCAACGCTTGTACCTTTTTACACTCATCCGTGGCTAGGCTTGTCAAAGCAAGAACTCCAACCCCCTTTTCGTCATTTCACTACTTTTCTATTTCATGCAGTTTAAACAAAATACCAAAAATTTCAACACCGTGCGTTTCCTCCACATCGACGACATACATCCCTCCACGCAATAATTTAAATTCATAAAAAATTCGCAATTAATTACAATGCCTCCTTAGTTTCTTCTTTTTCTCTTTTGTAAAGTAAGTACTCCTGTCTCTCTCTGTTTCTCTCTCCAGATTCTCTTCTAAACAGATCCCTAAATCACTAACCCTAATTCCTCCGACTGTGATCACTGATTCTTGAATTCTAAATCAAGATTCACGATTAATACCCTTTTGAGTTTTGCAGTGTGAAGGATTTCAATCTGTCGTTGGAAATTTAAAAAATTTGTTACCTTTTTCTATCGTGTGTTACAGTCCCGATTGATTGCTTGAGAATTAAGGTTTTTGTTTTGCAAATTTTTTTACGGCAAATAGTGATTATGTACATTGAATGATGAACGA

>Cs1g19450

ATACTAATGCAAGTTTCATAATCATACAACAACAATTATGAATACATTGATAGTTAAAAAATTAATTTTCAACATAATCGATCCAATTCTTATCTTGATTTAAGAATCGTTGAATTAATTTTTAATTTAATTACATTGGCAGTCAATAAATTCACAATAATTAATTTTTAAAAATATTATAAAATAATAACAAATAAATAATATATATTAATTTTTCAGTATGAGAAAATTGACAACCCCGTTTTGTAACTTAAAAAATAACAAAACTTTTTTCCCAAAATGGGCAATATAATAAAAAAAGAAAACAAAAGAACATTGAATAATTGCAAAAATCCAAGCAGTCCAAAATACAGACCATAGAAAAATGTCAGACTCAGACAAAGCAAGCTGTAATCGCTTATTGGAGAGAGTGAAAGAACTGGGGGAGGGGCGGGGGGCGCAAAAAATGTTAAAAAAAAAGGGATTTTTGATTTGAAATAGAAAATCTGAAAGTGATTCTAGAGGGAAGAGAAATAAATACACTCTTTTTTAATCTCTAAACGAAAATAGCGACTTTTTTCTTTTTCACGTTTTCTCAAATAATAACATAAATTATCCATATATATCACAAAGACCTAACTCCACATAATTGACCAAAAATAACTGTTAATTATGTTCGAAAAACTTATATGAGAAGGATACACTATTATTTTTTAGCAGTCATTTAATTAAATTAAAACTAAAGGATGAGATCTCTTATTGAAACGTAATTGTATATATAGTAATATTTTAATAAGAAATTCTTCTTTTAAAATAAAATAAGGAATCCTTTTAAAAGATGCAAAAAAAAAAAACAATTCAATATAATACACTATATTAAAATGTACATTTTCCTTATCTTTTAATTATCCATTACTTTAATAAAATAAGAATCATTATATAATATGATTGTATATATAATAGTTTGCATATAAATGAGAAACTTCTTCAGCAAAATACAAACTTTCTCCTCTTAATATTAT

>Cs1g02890

AAATCATTTGTTTCATTTAAATAAGTTCCACAGAGATTCCTCAATCCCATCACCTTTCTGGACATGCCAAGAGTGTGCGGAAAGCAATGTAGTGTTTCCGCACACGCGCGCAAGAATGAGATCATGCGACTATTATTAAATCATTAAAGAAGTTAAAATATAAGAAGATTATTAGGCTATTAATGATAATCTTTTTTTCTTTTTTGTCCCCTTTCTTTTCGATATGAAGGTATAATTATTCCTTAACTCAGGTTGTGAGCAAGTCATGCCTTTATTTATATATATGTATATATGATTATAGAATAATGTACCGCCTTGCTTCTTGCATAATCTGCTCATTCCTCCCTATTAGCAGCAACCTTGCCTGTCCTTTCTAGGAACTGGCGCTCGCTGCATTATTCTTATAATTTAATAAGTAACGAGTATTCTATCCCCCCCCCCCCAAAAAANNNNNNNNNNNNNNNNNNNNNNNNNNNNNNNNNNNNNNNNNNNNNNNNNNNNNNNNNNNNNNNNNNNNNNNNNNNNNNNNNNNNNNNNNNNNNNNNNNNNNNNNNNNNNNNNNNNNNNNNNNNNNNNNNNNNNNNNNNNNNNNNNNNNNNNNNNNNNNNNNNNNNNNNNNNNNNNNNNNNNNNNNNNNNNNNNNNNNNNNNNNNNNNNNNNNNNNNNNNNNNNNNNNNNNNNNNNNNNNNNNNNNNNNNNNNNNNNNNNNNNNNNNNNNNNNNNNNNNNNNNNNNNNNNNNNNNNNNNNNNNNNNNNNNNNNNNNNNNNNNNNNNNNNNNNNNNNNNNNNNNNNNNNNNNNNNNNNNNNNNNNNNNNNNNNNNNNNNNNNNNNNNNNNNNNNNNNNNNNNNNNNNNNNNNNNNNNNNNNNNNNNNNNNNNNNNNNNNNNNNNNNNNNNNNNNNNNNNNNNNNNNNNNNNNNNNNNNNNNNNNNNNNNNNNNNNNNNNNNNNNNTTTCCCCCCCCCCCCAAAAAAAAAAAAAATAACGATATTAAGTCAACCA

>Cs1g03270

GTAATCTATACCTGCATCTATTATTTTCGTATTTTTGTATGTCAACTACCATTTATAAATGTCCTGGTAAAAATAATTTTAATCTCGTTTATTTATCTTTCAATGAAGACTTTTGACGTATTTCTCTCAAGCACTTCGGAAATGAGACAAATAGCCCTTCAAGTTTGAAAGAGTTACAAAAGAAAAGAATGATAGTGATGAAGTACATTTCAAATTGTAAATTTATTACACTACATACGTACATACATACATATATATATGTATGTATGTACGTATGTAGTGCAATAAATTCAGTTAAAACCTGATAGAACAAATCATAGAAAATTTTTTAGTGCTCTTAAAGCATTATTTTTTTTTCCCCATCCGTTCCTTATTAATTACCACTTTTACGCTTCTCAAAAGTAATCACACGATATCGTCATTAATTATTTAAGGGTCGCCATTAATTATCAATTTAATGGGGAAAAAAATATGACGCTAAGGTAATGCAAAAATCTTATTAATATCTGTGAAGTCATCATACGGAAACATGCATGTTAGATTACAGATGGCCGCAAATATTCTGAATAAGCAAGGACAGCAATGTATGAATCATGCGATTTAGTGATTACTAATCAATCAAATTAATTAGAACTCACCCAAGATTAAAGAAAAATTTGAAAGAAACAAACAAACAAAAAGAGTGAAAATAAGCAAAAGTCTATCCACACAAAACATGCAATTGATTTGAACCAGTAGCCCTAAACGCACCTGCATGGATGCCAGTGCAGATACAATAGACAGCAGCGAATTTTCACAGAAACTTGCGTTAAGAGAAATAATTAAATAACATATTGGGGTGTCTGTAAATAAATTAGCCAAGGACCTCTCGATTAAATTGTCCACGGCCATTCAATTCGTTGAATTATATATAATACTTACAAAACACTCACTAATCCAGCAAGGAATCATTCTCAAAACTTAACTTTCACACACGAACACTCACATACAAAAAAAAAAAA

>Cs1g12700

ACTTTTTAAATTTGTGTGTAGATTAATAATAAAATTGAAAATAATTAAAAAAATAACACAATAATACTTTGAGTTTTAACAATAAAACAAATCAAATAAAAATTAAAAACTTAGAAAAATGTATATAAAAAAGGGGTATTTTCGGAATTAAAATGAATATAATTAAAAAGGGGGTTTTATGAGGAGATGAGAGAGGTGCCAATAGTCCAACTCTTAATATTATTATTAAGTTGTTACCTAAAAGATATAAAAGATAACCAATAATTTGAGGAGTGTTACCAAAAAAATTTTAGGAGACAATGGCTAGCATAAAGTTTTCAAAATACATGTTTTTCCTGAATCTGATTATTTGTTTTACAGTGTGAGAACATTAAAAAATAACTAAGTAAACAAATATTGAACTATTTCAATTACCGACATAAATATACCAACTTCTTGTTATTATTATTATTATTATTTTTAATACAACTCTTTACGTATAGAACTTATTCAGCACATATTTGCCGTTTTAGGAACCTCGAAACTGACCAAATGGAAGGTCGCATATCCCCTTCTGGAAGTTATTTAAATTTCTATGCTAAATTCATATTACAAACAGCTAAAATTTTGGAACACTAATCCTAGCAAATTTGTCTGAGGGGTAAAATACGAAAACCAACTAAAAAAAATCAATATCCACATCTAGGAGAATGATGTTGCAATATCGTATATGAATTACATTTTATAGAAAAAAGAAAATTGATAACCGAAAACAATGATATGAACGTTACAGAAAAGTTATTATTATTTTCAAAGATTCGACAATATAACCCGGCGGACTGCATATGGAACAATCTTTAGAAAGAGCGGTTTCATGAGCAATAAATCAAAAGCACCAAAAATAGCTTACCAGCCTAGCAAGACGTGCTCGTTGCCACCCAAATTCTATCCCCCCCCTCCCGTCTCCTTCTCTCTCTTCTGGTATTGTTCATCCTGCAATCTTTCTTGCCGGCAGGCCGCCA

>Cs1g20690

CACCCTAGCACAAACCCTCAGTCCACATTTTGCTTAACAAACAAACAATATGCCCTAGTGCTTTTAAAATTAACCTGAAACACAAGAATTTGGTCCATATGTTAGGTGCTCCTTGCAAGTTGGCAAATCTCGAAGCCCAACGAAGATAAATGAGTGATTTTATCCAGGCCGTGTGTGTAATTCCAGGCCCATATCAGTTGAGACTTTTCGTGAATAATAAGACATGGATCTAAAATTCACGCCCAGAAGACAGACTTTGAGTGAAAATAAAATGTCTGAGAAAGTGACACAATGCAGGAGTTTGAGACTCTGTGTTTGGTGGTAGAAGAACCGTTCAAATCAAAGTTTATGTGGTCACATTTGCCTCACAAGAACAAGATAGGATATCCCACATTCACTTTGTGGCTAAGAAACGGTGGAATTTGGATAGGGATTTCACGGCTGGTTTTTATAGGCCTTTTATCAATTTAGTTTTTTCAATCCGACCTTAGCTTTTCGAATTTTAGTTTCAGTATTTTCAATCAACATTGTAATCTTTATTGCCTAGCATTTTCCTAATAAATAAAGAATTGATGCATCGGACACCCATTCATGTACACACGATGATGATAAATTTAGAACCCATCTTAATCATCGCAAACCACACACATTTACGTATTTAATTTTTGTCATCTTCGATAATCGCGGAACCTAAAATTAAGAAATTTATGAACTAGAAATTATAGAATTTCGAAAGAACAATTTTATCGCCTTTACTTGATGTTTAGCTCAAAATTTATAAAGAATTTACTCGTTGTTGACTAGTCATTTATTTTTGGGTAAAATTAAAATTATAAATTTATACAGATGTAACTCGATTGATTGAATTAAGTATCTCTAAATTCATATGATTTGTTTTTATTACTCTTTATTTTTGTTATAGATCGGTGATTTACAATAATTGAGAATTATATAATCCTTAATTTATAGTTGCTAAATTTGGTGAATCCGTTGTAGGTGAG

>Cs1g09440

GATTGGGGTATCGAGCGAGTGTTTACTGTAAGTGTTGACAATGCAAGTCCAAATGAGGGTGCTCTTAGGTACTTGATTGATAGAGTGAAGACTTGGAGGGGTGATGGCTTAGTGTTGAATGGAGATTATTTGCATGTTCGTTGCTGTGCACACATATTGAATTTGATCATTACTGAAGGGTTGAAGAAATTAGAGCAGAGTATAGTTAGTGTTCAGAATGTGGCAAAGTATGTGAGATCTTCCACTGCTAGAATGCAAGCATTTCAAATTCGTGTGCAACAAGAGAAGATTAATTGCCGAGGAAGTGTGATTTTGGATTGTCCCACTAGGTGGAATTCTACATATTCCATGTTGAATACGGCATTAAAATTTAAGCCGGCATTTGATCGAATGGCACTTGAAGATAAGCTTTATGATGCTTATTTCAATGAAAAAGAGGGTGGGAAGAAGAAAAAGGAGGGGCCGCCATTATACAGTGATTGGGAAAATACTCAACGCATAGTCAAGTTTTTAAAGACGTTTCATGATGCGACTTTGCAGTTTTCTTCATCTTTGAAGGTAACATCAAATCTTTATTAATTTCTTTTAATTTTTTATATAACATTGTTTTTATTTATATACTTTAGCTTTATTAACATTGTTTTTATTTATATAATAGAATACAAAAGACAATTAAAAAAAAAAAAGAAATTCTATAAGCTATATTTTCTATGAGCTATATTTTCTATGAGCTATATTGTTTTTAATTTTTGATATATAATAATATGATGAAGTTTATTTTAGCTTTATTAACATTATTTTTTTTTAGGTGACATCAAATATTTGTTACAATTTGATAAGTCAAACTGAACAATCTTTGGGATCATTGTCCACTAGTAACGATAGTCTTTTGGGTTTCAAGGCAATTAAAATGAAGGAAAATTTTAATAAGTATTGGGATGGTTGTTTCAAGATAAATAAGTTGTTGATTGTTGCTTCCATACTTGATCCAAGGTGAAAAA

>Cs1g16520

CCATTTCCATAATGTTTTAAAGGAAGACTGATGAAAATTTTAAGTGCTTCCAGGGTTCAGATGATGATCTAAAAGAAATTCATATAAAGGAAGAGAGAGAGATTGATGCAAAGTTTTGTGTGACTATACGGGTATATTTGTAAGGCTAGATAATTTTGTCTTGAGCTTGAAATTGGAATATTAGATCATCTAGGATTATTTTTTAAAAATAAAAAATTGTTGCTATTAAACAAATCATTTTGGAAGAAGATCATGCAGGAAAGAGTGAAAATATGATAAAAGGAATTAATAATATTTAAAATTTAAAAGAAATTCATATAATAAATTAATTTTAACCATTAGATTGATTATGGTTTGAAGACTAAGGACATGTTTGAAATTGTTTTTAAAAAGTTCAAAAGTGATTTTGAAAAGCCAAAAGTTAATTTTGATATTTAATAATTTTTAATCTCTCTTATATTTAACACAATTCTATATATATCATAATATAAAAATATTCCAATCTAAGATCTTGAATTTGTTCAAGTCTGATAAATCTATTAAGTCATATAGTTAATAGTATAATGATATTATATTATTAAACCATATTACTTGATAACATTCCCAGATTTGATAAATGTATAAGTTCTTGAACCAAATTAGTACTCTACCCTCGTATATATTATTGAAATCCAACCTTATCCTCGTTAACTTTATGTTGCTCTCTCTGCTGTACCGTACATCCGCCTTGGCCGTATATTATTATTATTATTATTATTATTATTATTATTATATTGTGATAAAAAAGTAAAAAAAAATAATTGATAATATAAAATATTTGTTTTAATTATCAATTATTACATATACACAATTAATATATATATATATACACGTGTGTGCGCGCGCGTGTTTGAATCCGGGCCCGGGCCCAGGCCCAGGCCCAGGCCAACACCGCCTAGGCCGAATCCGAAATGGTTGATTCTGCCCTTTCCTCAGCCTTCAACTTTTATAATTGTATTAAA

>Cs1g08620

ACTTTTTTAGTTTTTATTTATTCTAATTATTTTAATTTTATTGGACTTTCAAATAATATAAAAATGTATTCACATAAATGGTTGTTGCACTCTTTTTTTTTTTTTCAACTTTTCTTGCTCATCATTTATAGTTAAAAATCTAAACTTGTGCTACATTTCTATCCTTGCTGAACTAAAATAATGGTACTCGATAATATGAGATTTTCCCATTTTAATGAAGTGCTGACTAAAATAAAAATATAAAATAAATACATACTCTAATATGAACTCTTATAACTTTACGTAGATTGTTTGCATCTTGTCATTCGGATGAGTTAAAATACAAAAAAATATGAACTCTAATAACTAAAACACAAAAATATATATACTAAAATGCGTATTTTTGTTGAATCTTAATTATATCCACCTTTTTATTTAAATATGAATGTCTACGATAGGTAATTTATCTATATATACTATTTACATCAAAATTCAAGATTAGATTTGGAGGAAATTATAAATGTATACGAATGAGGAGAAAAATGAAAAGAAAATATATAACACAATAAAAAAATTGAACTTAAGGGAAAAAGGCAACAACATCATCTCATAGTTTAGCAAAATAGTTGCAGAAACTCCTTGTCTAAAAAAATAATCACACTATATTGATTTAAGTGTATTTCATTTGGACATAATTATTTAAAAAGAAGTATTTGTAAATATTTATAAAAATAAGAAGTTATTAGGAACCATTTCATTTCATCAAATTATAAAATGGAGCTGTCTTTTTTTCCATTTTAACATAGAGCACGTGCGATTAACGTTAATTTAATTTGTATTGGTTGATGGCTTTGTCATGTTTTGTTTTTATATATATTTTTTCTGTAATTTTTCTCCCAATTTATATACCTGTATAGGTAATATTGGAAAAATTTTACAACTTGTCAAATCTATTGAATAATTAGTGTTAATTTCAACTCCATCAATCTCAGTATCTCACCAAAATTTTAACTTTTTCTCAC

>Cs1g14390

ATCTTAGCCAAAAGGCCGAGAAAGGTATGTTTTGTTAAGTGTCTCGATTCTGTTTTTATAGCTTCCGTCTGCAACTTGCTTAGGTTGCCCTGTCGATGTGGGAAAGAATTTGCTGCATCTGATATATAAGTTAAGGAGATCATGAGAATGGATTGTGTGACCTTTCGGCATTGAGCTGCAAGGTATTATCATGAACTGATTCAGTTATTTTAGCATTTTTAGCCCATGAGTAAAAAGCCTTTCACTCAACCTTCAACCTTTCGTTGTTGTTGTAACTCACATTAAGACGAAGCATAGATGGATCGTTTAAAAAACAAATTGATATTCTTGTTCTGACAAGGTAAGCCAACTTTCATTAGTACTTTACATTAAAGTGGGTATAGCGAAACCAACATCTTTTATGGCATGAATCAAGGCCCCATTATTATTATGTGGTGTCAAGCCAACCTTTTGCTTCATCACCAAGAAGCCGAAATGGCACTGGATAAGGGAACTTAAACGGTAAAAATTTGAAAGAAAAAGCTGCTTTATCAAACTGGAAAAGGATTCTTTTCCCGTCCTTCTCTATATGCAACAATGGGAAATATCAAATAATCGTGACTCAATTAATTGTAGCAATAATGATGTAGAAGATGATATGGGCTTAATTTGGGTGTGAGTTATACCAAGACTCATGCCATTACCAACAATGACGGAATCAAAACCATGGTAAAAATTATGAGTGCTCAAATTTTGACACCAGAAATTGTGTTTTCAAAACTGCAATTGATTTATCACTAGAGTTTAAGATCATCAGCCTATTATAGAAATTAGGGGTGGGCATTTGGTTCGGTTCAGTTTGAACCGAATTGAACTGAACTGTATTATAATAATTTGGTTCGGTTTGATTCGCATTAGAAAATCAAATAGTTTAAAATTTCTGAATCATTTGGTTTTTAATTTGGTTCGGTTTAAATCCGAACCAAACGGTTCAGTGAAAGAATTAAAAAAAAAAAAAAAGT

>Cs1g25660

TTTGGTAGATGTTTCCCAGGATACAACGGGCACGAATTTTCGGTCAAGTACAAACAAGTAATTCCGGCGAACCAGAACAGTGGCAAACTCGATTAGAAAAAAACAGATGAAAGGAGGAAAGGGAAAAATTGCAAGTGCTCTATGTGCTGGTTTTTTTGTTCGTTTAAAAAAAAAAAAGGGAAGGTGCCTTTTTATACTCATGTGGGTAGGTAACAGCAGCAAAAGAATTGAATTTCGAAATTCAATATGAGCAATTTAATTGCTGTAACAGCCAAAAATAATTGTAAAGTTGTTACAAATCTTTTGAATTCAAAGAGGTGTGAAACTGTTACAAAAAAAATTCAAAACGTGCCATGCAAGCTTGGATAATTTTGGGCTGACATTCGCGTGCGCACCCGAGTTCAACCAAATCGGTTTTGAATTTGTAACCCCCCGCGTACGCACGTGTGGAGAGAGCCTCTATCCTTATTCATGCATGGTTAAGTGTTTATATATAAACACTAATCCCTTGGCTTCTTTTTCCCATGTGAGATAAGTCTCATTTCCTTTCAAATTTCCAACTTCAAAGGTAAACTTTGAGAGACAATTTCTCATTCACTCAAGCACCATTTTGAGAAAATAAAATTATTCTTTTGAAGTATTAACGGAATAGAATCCGAACCTCACAAGATTTCTCACTTTTGTTAAGTGCAACCAACTCAAAATGTGCCTTAAAATAACATATATACATGCTATTTTTTCAACAGTTTAGTCACGGGTAAATATAGTCTATGCGAATATAATGGACTCTAGCTCTGAAAAGTTGCAAAAGTGAAGAAGCACAGAATTTAGCTGAGAATGTTGATAATGGCTTGCCGTTTTCTTTTTCACTGTCCATGCAAAATTTGGAAAAAAAAAAAAAAAAAAGCTCAAACATGGCACGAACTCCGAGCCACTGCGGACTTATTCACATCCTAAACATAAAATTTCAATTTGATTTATTTTTCTTGTAATCTAATCTC

>Cs1g22550

TATTTTATGAAATTATTAGAGGTTCTAATAATTTATTCATTGTACCATCTTTCTAGTAATTTATTAGAGTCATAATATATTTATGACATATAATATCTCTTATCTAAATTTAGCATCTTGAATTATATAAAAGAAAAAAAAATCTAATTTATGTCTTAAGAGAGACAAGAAATTATAAATGCAAATGCAAATACAAATATATATTTAAATTGTTATAAAAATATAGTAAAAATTTTAAAAATTTTAAAAATAGATATAAAACAAAATCATTTAGTTATAAAAAATTAATGAGAACTTATTTAAAAAATAAATAAATATATGATTCAAAATAAAATTTTCTGTAAATAGTCATTACACCCATACTTTATTTTTCGTTAAAATTATTTTCATCCATTAAATTAAAAAATATCAAAATCTAACTGCCCATATGTACTGACTGTTTGTTACAGCTGATGTAACATAAGCCTTGCCTAATGATTGTCATTCAGAAAGTTATCAATAAAAAAAGACTTTTTTAATGTGAAGAGTAAGCAGCAAGGTTCTGAAGAAAATTAATAGGAGACTGTACAGTGGATTTCAAGAGAGTGAAAAATTGTTATAAAAAAAAGACATAATCATAAATCAGGAGTTCTGATTTTATTTTTAATATAATTATTTTGAGCAATAGGAGAGTTTATTAAAAAGGGTATTATTTTAATCAAAGTAAATATAAGTGAAAAGGGAGTTCTGTGGAGGACACATGAGCGGCGAAAATAGTAGGTTCACTCATAAAGAATAGAGGACTGTGTAGTGTGCAGTGCGGCCCAATTCAATAATAACACGCCCGACCAGGCTCGTCCTTTAGGGCCCACCGTCCTGATTCTGACATGCAACCATGTGCTGTCTTACTCCCCAACTGCCTCTTGTAACAAATTAACAACAGTGACCAGGACTCATAAACATAAAGGCCTTGCCTATTTAATTACATCAACCATCACATTCAAACCTGTATCGCGCTCACT

>Cs1g21440

TATATCTTTCGATGCGTATCATATGAATTAAGTTTCTTTATTTTGTTCCTATATATGATATTTTAGGGCTTTTAAAAAAAAATTGAATCGATTTAAATTTAGTTGAAGGCTGAATAGGTTTAAATGCAGGTATGTGAATGATATTAATTTTTATGAATTAAAAATATTTGAAAGGTTGTGTTTTGATATTTATACTCTTATAATTATATATATATATAGAGTAATTTGATAATCAAAATTTAATAAAAGATTTTTGACATGCCAAACTCTATTTTAAATTAAAATTTATTTGGTAAAATTAAATAAATTAAACTCTTAGTTATACTTCTTAAGCTCTTTCTCTCTCACACTCAATCAGAGTAACTATTTAATATTTCAATTTAACAATTTCAATAAGAAATTGAAAATTTGTGAGGAATTCCTTTGGTTTTCACATTAACTAATTATTATCTCTAAATATCGAATTTAGTTCGTAAAAAAAATCAGTGTTAAATATAAAAAAAATTGAATGATAATTATAAATACAAAAATTAGAGTGTGATTATTTCTCCTTATATCGTAACTTGACTCTTAGTTTTGCAGTCATCCAAATTCAAATAATAATACAAAATAAAGGTTGGTGCTTATGAGGTTTCTCACAAAAATGACTACTTAAAAATAGTAGTATTTTTTAAAAACTAAAATTAGCATTAGTTGAAGTAATAGTATCTCACAAGTGATAAATTAAAAAAAAAAAAGATCTAACAATTGATTTTGAATATACGTTAAAACAATAAATAAATCAAAATATGTATTAAGTTAGACGTTCACAATTTTGTTTTTAAGTTGGAAATTCAAATTATATAGTAGGTGTGAATCGTGTGATTCATCCAAGCCCTTGTTCTCCAAGGAATCTGAAAGCAAAGCTGAAACGACCCTTCGTGGCAGTTAGACATTGTGAGCTGTGACCAAAGCTTGGTTTAGCCCAGTCTCCGGGAGTTTACGAATTGCGACTACGACCC

>Cs1g25980

CCTAACCATTTGTCATTCAATAAACTGATAAATAGGGTGTTCAGATTTTTTTGATTGGACATGGAGCATCATCTGAAATTGCATAACTGATTATGTCAGTGTTATGTTCATATTCCTGTCATTTTTTTCCCTGTTTTTTTTTTGGGGGGGGGGNNNNNNNNNNNNNNNNNNNNNNNNNNNNNNNNNNNNNNNNNNNNNNNNNNNNNNNNNNNNNNNNNNNNNNNNNNNNNNNNNNNNNNNNNNNNNNNNNNNNNNNNNNNNNNNNNNNNNNNNNNNNNNNNNNNNNNNNNNNNNNNNNNNNNNNNNNNNNNNNNNNNNNNNNNNNNNNNNNNNNNNNNNNNNNNNNNNGGGGGGGGGTGTTGCGTATTATATTCCTTTATATTATTTTGTGTATTTATTGTTTACAGTTGTGTAAAGTTGGTGAATTCTTGTGATAAATGTATGTTTGGTATATTTTGAAGTCATCCTAGTAGTTGGATGGAATCTAGTCACATTAAGTGTAACTGTTCTTATGTTCTATATTTCAAAAGCTTGACTGGGTAGCGTTTTTTACTTTCTTTCCTACAATTACACTAGGGATGTGCATGTGCTGAACTCTGATGATTTTTTAGGGCATGCCATGTATATAATGTGATCACATCTTGTTCCACGGATAGTTCTCCATGATTTCAGGTTTTGAGCATTCACCCTCATTTGACCGTCTGTTCAAGAAGCACTTTTTTATTGCTTGTACAGTGCCAGCTCTTAAAAGCTGTATTTTGTGTATGATTCATTATTGCATATTGCCAGCTTTATATAGCATATACATATATGGACGTTCAAAAGCAGTTATGCTGGTCTATATTATTGGGTGATATTCGAAGTTTTCAACACTTGCTGCGACAATACATACATATGCCTCATTAGAAATGGGGCGTAGTAGTGCTTGCCAAAAATGTGAGCTGGGGCATTCTTTCTCTTGTAAAAGAAGAAAGGCATAAAAATTCAACAGTACATATTGT

>Cs1g04970

ATCAAATGAAAAGACTAAATTACCCCTCTAGCGTTATCCTATTTAAACGACAAGTAACAGTTTGGTGGACGGCAGATACATTTTGTCAACTTAGGGTGGACGATTGATACACCATCAAAGTATTGTAGTATTTTTTATAACAGAGCATCTATAGGGTATATGAATGATAATTTCTCATATATATATATATATATATATATATATATTATTTAACCATTGACTACTTTGATCATATAGAATTTAATTATACCCTTATAAAAATTAAGGTAGAAAAATATTATTCACCCTCACAGTTGGTTCCAAAAATGTATTTCTCATTTTGGTTCTAATCATTATAAGTTAAAAAAAAAAAAGCATACAATCACCGCTAATTTTATTGTCATGATTACATATGAATTGCTCAAACTTTCTATTTCAACTATCGAATCTTTTAGCCGCTTCTAAACATGAAGAATTTAAAGCTATAATTTGTAGGTGCAATTGTATTTGCACATGCCGTGAGACAATACTCATTGACATTATTTTAATTTTTAATAGTTACTCTTTTCTTTTCTTTTTTTCTCTCTCCTCTCTCTCCCTCATTTCCCCCCTTCTTTCCTTCATTTTCTCAAATCTCAAACGCCCACTATAAAATATTTTTTTTTAGATTTTATTATCTTAGAGAATTATATTTTGTACATGATGAAATAACTTTAATGGGTAAAAACTAAAAATTAAAAGAAAAGACTATTTGAACCCATAAATGTATTTGTTATACGAGGATGGTTTAGAAATAAAAAAATATTAAATGAAAATGCATTTGAACTTTAAGGAGTGATTTTAGGGGTTAGAGTAATGAATTAACTGTAATTTTTTTTTTATCACAATAGGGTCAAAATATAATAAAATAGAGAGTAAGAAGTTAAGTGCAATTTGCCAATAACAAAAGACAAAAAGTGGGATATGCTGTGATAGCCGGACGGGCGGACCCCTGGATTTAGTTGTTAATTACTGAAGAAATT

>Cs1g23640

AATCACTTTCAAATTGAATTCAACAAACACTTTTTAAGTTTTAGATTGTCAAATAATTGATTATTTAACAATCTAAAGCAATCCCAAAACATACATGCAGTTGATTTTTGATATTTGCTAGCACAAACAAAGTGAAAATTGTAATTTTAATGAATATTTTATACAACTATTAGGATGATATATGACATTTTCTATTATCAATTTTGCCCATAATTAAAATGTGTTATTAATTTAATTCAATGACTAAGGATGTATACATAATTTTACTTGATTCATATATCCAAAATATTTAAGTATTACGGCTGATATTCTAATCAAACAATTATTTCCCCTAATATAATCTCCATCCCCATTGAGGAATATAGAATATAAATGATATTTGTAATTTGTCCTCCTCCATACGTAAAAAGAGATGGGATTTTTCAAAAACATATTTCAATTAAGCAAACCCTAGAGATTAACTAAATTTAAAAACAATAATTGCAATTAATTTATTTCCATAAAATAAGGTCTAAAATTGTTCTATTGATGGCAGCTCTATCTTAATTACAGAACGTTCCAGACATTTAAACCACCCGAACTGCGAAGTTAACTAACCGGGGAAGGTCATATCTATCTTGTCATTTATAACTAACGCCCACGCGGCAACACTACTGTGTGTAATTAATTAGCCTCTACGTAAAATAAAATCTCTCTCCGTCTTCAATGGTAATCAAACTTTGATAGATATGAACAATATGTGGCGACACGTTTGCGCAAACGTAAGACCAATCAATGACCAGTTCTCGCATAGCGGCCGTATGAGCGGCATTACACTACATGTAAACAAACAAGCCTATTATATTATATATTTTACTTTTGACCCTTCAAAATAAATAACCATCGCTCTCCAATCTCTCCCCCTCACACGTGTTAAACGTGTGATTTCCAACATCACCGGTTAGAGTCAACGTCAGTTGCCTACTCTGATCAATAAATTTTCATATGATCGGACGGTTGTG

>Cs1g24730

ACAGCTAGTGGGTTGGACAAAAATCAAAGAGATACGAGATCTAATGAGAGAAAAAAAAAAAAAAAAAAAAAGGAGATGGAGAACCTTCATTTGGGAAGATGAATTGAGACAAATATAGTCACTATGCAGTGCAAGATTTTTTTTTTATTTTTTTAATATGTGTGTATGTTTTGTAGAAGGAAAAAAAAAGGTTACTCAAAATCTGAAAGATTTAACTAAAGCACTTGGAAAAAATACAATTGAATTTTTTGATTGAGAGATAAAGCTGCTAGTGTATTCTAGCTTTATTGATAATTTTTTCCCCTTAAAAAAATAAATAAAAAGTAGAAAAAAGGAGATGGATTTGAGGGAAGGATGGTTGCGTAGGAGGACTCCAGCGTCCGTGTTCGGCTGGAAGTGTTAATTGTGGACCATTGAAAGACCCGAGCCAAGACAACAGCAGCAATTGAAGCGTTCCATCTAACATTTTTCTTGTTGTTACTATAATGCCCCTGGTCAGCGATCATATTGACGAGTCAAACCCCCTTCAAAATGAATCGTATAATATATGAAGACCACTGTCGGCTGCGGAGGCTTTCTGGAATAGTATTAGGTTAAATTTGCCCCTTATTGTTGATGCACATGTCCTGCTTTGGTGCTGAGCATCCGTGGTATGAAAAAGAGTATATATATATATATATATATATATGACACTTCGAATATTTAAAAAAAAAAGGGGTTAAAATTTGGAATACGGAAAACTTTAAAAATATAATTGTATTTAATGTTTTATAGCATTATTTACAGTGTAGATGAATCTTGTAGCATATACTTTTTGATAAACACTAGTTCATGCAAAGATTGATTAATTTCTGAGTTAATAAGAGAGAACCCAGATACCAAGATCATAAAAAACAGCCTCCCAAAGCATCCTTTAATTAGCTTTACTGATTTCTGTTAACGCGTGCCGGACACTCGCCAATAAGATCACAGGTTTCCTCTTAATCCGACTAAGAACAAGC

>Cs1g05560

GGACTAAAATTTGGTCCCATAGATGTAACCTTTTTGCCTCATCTGTTCTGTAATCAAAGTTTTGGTAGTGAAGTGGATTCTTTCTCACACGATTGTTCAAAAAGTAAGGCAAATGATTAGGTAGGAATGCTTTATCCTTCTCATGATTTATGTAAATTTTTCTGTTCCTCATAGTAAGGATATACCATTGTGGTTAAATGACACTGAGTTGCTCAGTAATTTAGTGAAATGATGAAAAATCTTCCCCTGTTTTCTCAAGTGTCATGTAGCTCTCTAATGGATGGATTGTTTGCCAGTCTTGAGTTCATGGATTTTGCCAAGTAAATGAAGATGCATCAGAAACGAGATGCCAATATTTTAGAGCAATCCCATATGAAAACCAGCTGATTGGCTAGAACTTGACCCCAAAATCATTTAAGTCTGCAAAAGCAAAGAACACTAAAGCTGTTCAATTTTATTGAATGATCAAGAAATATGGAATCTGATGAAATAAATTGAATTTTCTTCACGTATAATGATGATTTAAAAAGAATAATAATAATAATAATAATAATAAAGAACTTGTAAGAGACAAGAAAGGAAGTAGTTGATTTGGGGGAATCTTCTAGCCCTTCAACCTACCTCAGCTATCCTATGTTGAGGAGGTAAATGACTGCATGAACTTGAGAGGTGAGAGAGACACGTTTTAAGGAAGAATGTATGGCCACATCAATTTGGTCAATTTTGTAGAAAACAAACTCTCACATCAATCTGTAGCATCTTTTATTTTCAGGATAAATAAACAAATGCAGAGAAACTCTTTCGGGGGAAAACAGTACAAAAGAAGAAGTGTTTTTCAATGCAATCAATGACAACTCTTCAAAAACTTAGCCTGAGAGTCAAGTTACTTAAACCTTAGAAGATTTCAAAACATAAAACAATTGATACTCTTAGATCACGCGGTAAGTCAGCATTACAAATACGCATTAACAAATGCCATATTCACCATCAGAAGTTCAGCA

>Cs1g14520

GAACCGAAATGCATTGGAACTTTCTAAGAATTTAGAGAAGGCTAATTCTCAGATCTCTAACCTCGAAGATGAGAAGGCAGTGCTTTACAAGAGCCTAACGGAACAGAAGAGCATAGCCAATGAATCCCGAGAAAACATGGAAGATGCTCATAATCTAGTCATGAGGCTTGGCCAGGAAAGGAAGAGTTTGGACAAGAGATCAAAGAAACTTGAAGAGGAATTGGCATCCGCAAAAGGTGAAATACTGCGGCTAAGAAGTCAAATAAATTCATCAAAAACTCTTGTAAATGATGAGAATCCTCGGAAAGTTGAAGATGACAACAAGGTCCCAGTCACTGCAAAGAAAACTACTAGGAGGAGAAAGAGTAACTCACAATGAGACTGCAAACTAATTTTTTGTTCTCCATATTCCTAATGTTCATTTATTATCCAATGCCATTTGGCACTGAGACTTTGCATTCTCAGATTAATTTTTCGCGACGCATATGTATGATTATTGTATAAATGCCGAGGCATGAGATTAGGGAAATGGCTCTTTGTATGTAAGGATATAGTCTTTTCATTTCATTGTCTTCACTTTTCATGAATGTATAATGTCAAGTTGAGGTGCCTACTCAGTGTATTTACTCTTTTATTCTTCCTTTTTTCTGGGGTGAAGTATAGATTGCTGGTTTGCTGCAAACATTGTTGGCATAGTTTATTTTCAACATTGTGAAGTGTCATGCTTTCACATACATATAAAATACATATGCTAATTGTCCAACACAAAAGGGGAATTACACTCAAGCATTTTATGTTGCGCTATTACCAAAATTGATATAAAAAGTTTTCGACAAATCCTTTAATTTGTAAGATTAATAAGTTTATCATCTATAAATGTAATTTTTTAATACTGCCATTAAAAAAAAAAAACAAAAAAAAAAACGATAAACCCTTTGGCTGTACTCGAACCCGCAATCTTGAGAAAAGAAAATCAATCATGCTAAACCCCTTGGCCGCGA

>Cs1g22370

AACGCGCAAAACTCATCCATGACAACTACCCCTTTGTCATCTCGTAAATCTAGGAGCGATCCAGTACCCATGCCAGTTAACGGTATGGAAATTTCTCACCATTCGATGAGGATTTCAGTTCCCCGTCATTATTCTTCCGAAGGTGATGTCTTTCTTCCTTTTTCTTCTGTACAGTGTTTTTTGATAGTATTTGTTTTCTTGCTTTGCTGAGAAACCTTCTTTTTGATACAGAGCCGCCTCCCCCTGGTGTAACACTTGAAACTAATGGGTATGAGACACATCTCAATGTTACCAATTATGATTACAGTCGAAGGGTTTTTGGGGCAACATTGGATAGTATTAGTCGCTCTAGATCTGGCCACTATAATAGTAGTAATCGACATTACTGATGCTTATCATGTCACTTAGTCAAAAAGAACTCTTTTTCATGTAAAGTAAAAGTGTGGTACATTGTTATGGTTAAGGTAAGATAAGCTTGTTTCAGTTTCTAACTAGTTCCCGTAGGTGTGTCAACCCATGGCTGGACTTGAGAAAGCTGTACTTAATTTTTTATCGTAAAAAATTGTTTGTTGTTTTAATTGTAAAAGTAGCATCTGTCATTATTCTGGACTTTGAAACCTGATAGGAGGCAGTTATTTATTTTTAACACTTGTCCTGCAATTAATTGAAATGTCTTTTTGATTGTTGATATAGTTAAATTAATTTTATCATGTTGCTGCGGTATTGGTTTGTAGACTTATTATTATTATTATTTGGGAATGAACAGGAAAACTGATGATTTATGTATTTGTGTTTATTATATATATGTTTATTTGTGTGCTTTCATACATGAATAACCCCAATCTTAATTTGTAAACTTCTCCTGACTTCTTCTTTATAAATAGTAATATATCTATAGAAGTTTCTTCGTTGCTAAAGTAGTTGATATCTGGTTGAGATTGAGATTGAGATCTGACTTACTTGATAATGTGGAAGATTATAAACATATAGTAAACATGCAA

>Cs1g06460

AAAATATAACATATTAAGATTAATTGTTCAAATAAAGTTACAATAATATATTAATATAACACCGAAATTAATTGTACAAATAAAGCCACAATTAATTGAAATGGTGAAGTTTCTAAAACCAAACACAGTATGATCCTTCACCAGGGATATTGCACTTGTCGAAAAGTGGTGATTACGAAGTCGTCAACGATGAGAACTAAGAGAAGTACGAGTGGCGATTTTAAAGGTTGTGATAGTGCAATTGTTTGGTTGGAACCAATGATTGTGAAGTGGCTCGATGGACTTGGGAAAGTAGATGGCTTGCACTTGCTAGAATCACGCAGTCGACGTCCTTCAAATCCGTGTCGAGAGTGTATCGAATCAATATTGGGTGTTGCTAGTGCGTGTGACAACGAGGAAGCTTAGAGTTTTTTGTTTGCAAATTTTTGGTAACTTGACATTATGTTACTTTACTTTTGGGTGACGTAGCAAATTATTGGTAACTTGGCGTTATGTTACTTTACTTTTGCTAGTACGTGTGACTTTGCTTTTATTTTGTTTTACAATTTTTACAAGGTATATTTGTATATTTGATTATTTAATTATTTTTTATTTATTAGTATTATTGGGTAATGGCTAAGATATGTTATAATATTTTATGTAACTATGCAATGTCACATTTATAATATTCTTTAATTAAATTTATAATAATTTTTTAACTAAATAAATAAATATATATATATATATATAATTTTTTTTTTTGCGGATTCACGAATTTTTAAGGATTTACAGAAGTAAATCCATATCCAATCCATCAACTGCGGATTTTTAAATTTTCAATGCATATCCAATCCACAGATTTACGGATCGGATTTTCACGGATCAAATGGATTGAGCGGATGGGATTGGATTCTGAACACCCCTAGGTCACATACATAAAAATTATTTATAAAAAATTATTACTCCAGAGGTTTAACTAGGTGATTTTACAATAATTTTATGTAGTTTTTCTTTGTAGATTC

>Cs1g14710

TCAATCCAATAGTTTATTCAAAGTTTTTCTTTAAAAAAAAAAAATAAGGGCATCCAAGTCATCATTTAATATTAAATCTATGGATGAAAAATTAAATTGTCATTTGAAGCTAAAAATTTAAGGAACGCACTCAACTTATACGCGTAATTTTAGTAATAAATACATCTTGATTAATAAATTTGATTTAAATTCTTTAAATACTACATATTGCACTTAATTTATCAGTTTCAATTCTTTAAATAGTATAATAGATATCTTTTTATCCCAAATTTCATCTCAAAATGATGTGTTATTCATTAAGTGGTTGATAATTCATTACAAAATTCAAGTAAATTAATTGTATTATCTCAACAACTAATTGATGAATAACACATTATTTAATATAGTTTTTAGGAAAAAAATGAGAATGTGTAACATTACTTATTAATAAAATACCTTCAAGTGGTACTTAACACGAGGTTACAAACATTTTAGAAACCTAAAATCAATAGAGATTCCGGTATTAGTAATTAAACAGCCATAAACTGGTAAAATCACTTAGATTGTTTTCTTAATCAATTAAATGATTGAGATTTGAATGAACGAGTATCTGCTAATCAGAGAAGGTAAAGTATGAGAATAACACAAGGCCAAAGTTGAACCAATATATAACTTCCATGAAAACGAGATGCCTGCTTTGCTTTTTTTGGAAACGAATTACATGCTGTTGTGCCAGGGCAATTGAATAAAAAATTAAAAGGCCCAACATGATGCAGTAGGCCTGGCCCATTTACAAACTCAAGGGGCCACAAACATTAACACCATGACTCCATATTTAGAACATCATAAATTGAAATTAAATTTACTTATTGCAATTGTATTTTTAGCCTCAAAATAGAGTCCACCAATAAATAGTCACCGACTCACACTCAAGCCCTATTTGATAATATGTTCTTGTTCTATAAATTGAACCTCTCCCCAAATTAAAAATCACAAGAAATTTATCTAATTAGACAGCAGCA

>Cs1g09270

TATTTGATGACAAGGGTATTTTGGAAATTTTAGCAGTCCACTAACAGTCAACGTTACAAAATACTAACGCCAAGGGAGGTTTGTATAATTACGGTCATACATTCTCTCAGACTGTTAATACCCCAAACCTCAGGGGAGGTCCCTAAAAATTATCCCAATAAAAATTGAACATCGCAAAATCCATCAAGATTCAAGATAATTTTTGTGGATGGGAAAGCCGAAAGCCCGTTTGTGATAAACGCTTTTATCTAATCTTTGTCAACACAAGAAAAATTGAAAACTATTGATTAGTCGAGCCGCTTTTATCTATCTACCTCAGAGCATCTCTAAAAAGTTCTTTAAATTTTACTCTTCAAATTTTAATTTGCTTACTGATATGGCAAATAAGTGTATAGAAAAATATAATCCCCTCCAAAACACTCACCAAATAAGAATAAATTAATATTATTTTAATCAAATCTTTCCAACTATCAAATTGAATTGTTATTATATTTTTTAAAAAACATAAATAATAATTATTGTAGTCTTATCTTTTCAATAAATAATAATAAAATATTAATAGAAGAGGGAGAAACTCACTTGTCAATATTGAAGAGTGGGAAACAAATCTTATTTGAAAAATCTAAATTAACGTATCTTTTGGAGTATTTAATATTGATATAGCTCTTCAAATAAGATTTTACTTCTTATTTGAATAGCGTGTAGGAGATGCTCTAAGTTATCATTAAGAAAAAGAAAAAGGCCAAAAAGAAGAGTTCAATGTTCCATTACATAAAGGTTCTGTACATCGTTTACTCTGTTGTGATGTAGTCAAAATTCTTGGCCATTTCTGATCAAGCCGCGTAATTATTGCCCATACACCAATCGCCAATCCTTTCTTACGGGGGGCAACTTGTATAAAATTATTGACTCCCATTGTCCTACTCACCATCCGCATTCACATCATTTTCCCTTTACATATTATATGTAAACAACTCATTATTCAATACTTCGTTGTTTCC

>Cs1g25950

ACAGCCAATAATGGACTTGGATATGTCAGGATGTGCGCCGTTTACTTTCCAAGCCTACAACCTCCCAACGCCTTCTCTTACAAATTCATTTATGTCCCCCGAGCTCTTGTTTTGTTACATTTTGCTTAATACTTATGTTGTTTGTTATTCCGGATTTTTTCCCCCTATTTAAGAATCAAAGAGAATCATGGATTTCATTTCCTAGCTTTTAAAAGACTAGTAGAAGTCTAGAACATAGGATATGCTATTACCATTTAAGTTAGTCTTCGCCTCAATATTATTCCGAAATATATTTAGTGTATATATATATCTATTCTATCATAAATAAAATAAAAAATTCAAGCTTATTTATTGTATAGTCATTAATCTCGTCTCTTTACATTGTATTATTTAAACATATATATAACTTGATCGATTTAGTAATAAACTAATTTTATATAAGATATTATTTTTTTATTGAAGTAACTCGATAAATTTGTTAATCATAAAACTAATTATGTATTCAAAGATTACTTTTTATATTAAACTATTGAAATAAATTTGTTAATTAAAATAAGATATTTCTTTAAGATTCTAAAGCTACAAACTAAGACGTTACGCTAACTTGCAGGCTAAATAAAAAAAAAGTTGTTAATTAAATTCATTGACTTATCTAGTATCAACAACTAAAATTATTATATTATTACTACAATCACAAAGACTTTTGAAAAAATAAATAACGAAGATCATATGATCATTTTATTCAACTAATCATGTAGTTCTCAAATGAATAAAGTCATTGACAAATTCACACTACTAATTGGAGATTAAATTTTTTTTTTACTATTTATAAAAAAAAAAGTATTGAGGATGGAAAGAAGTGTTGGTAGATTTATTTTAGTACTCTTTAAAAGTATTTATTTGTTATTTTCTATATGAAAATATTCACATTACGTAGAGAAAAACTAAAATTGAAAGAAAGAAGGAAAAATGGGTAAAATTATAGAAAAAGAAGGGCCAAC

>Cs1g17890

AAATAACTAAGATGTTAAATTTTTAATTAAAATTTTTATCTTTGAGCTACTAAAAATCTTCAATAGAATTGTTATCGAGAGGGTTAAAAGTTATTTTAAGAAGTTAAACTTGACAATTTTTTTAAATTCACTTTTAAAAAACCATCTTATCTTAAAATAGATTCTGACGATATTTTTGTATATTTAAATGAACTCAAAAATTATATTTATTTAGTTAAAAAATAAAATCATTAAATTTAAAAAGACTTAGGATACATGCTTCTGGGATTTTTTTAAACTTACCATTTTTAACATTAAAATTTTGGATCAGGCTACGGTGCAATTGTGGCACCGTGCCACAGTTGCATCTAACCGTTGGATACAGTTGGAATGGTGGGGTTCACTAGCATCCAACGGCTAGATGCAACTGTGGCACGGTGCCACAGTTGCACCTAAGGTTCACCCTAAAATTTAATACTTATAACAACAATTATTTCTAAAACTATAACATTACTAAATTACTCTTAAAACCCTATTTCCATAAACGTATAAGAATTAATGAATGAAATCAATAAATGAAATGACTTTCACTTTTTGTTCAATCAAAACGCACTGCACAAGTACACAGCCATTTATATAGTACTGGTTTGATTTATGTCACCTGGTGATTATTCTCACAGCCTGTCATTCACGGCTGTTTCACCCATTTAGGGTAAGAAAGATCCCTCTTTACAACTTTTTTTTAACCGTTCTGTATGACTTTGAGAAAGAGAGAGATCGTGATTTGTGATTTGTGACGCACACAAAACACAACATCAGTTGTAAGAATTGTACAGTGGCTAGCTGTATGTTTGTACAACAGCAAATGCCATTATTGGACTGCCTGTGACTTGTGACATACCAGACAAGAAGACACGTGTTCCTCTTGTCTCACGAAAAACCAATGAAGTAGTTGTCTTCATGTAAGCAGCTACACTTGAACTGGCCCCGACCTATAGCAATTTTACTTCTTTATTTATTTA

>Cs1g26620

TACTGAACGGGTAATGGTGGCAAGAGCTGCACATCGATGAAAGGGCTATGCTGTCTACAGTTGTGCACTTGGCTGAAATGGAAAAATTTGGGGATCAGGCTAAATTGAACTGAATCCGAGACTCTTGAAACTCATTTTATTTATATTTTTTCATTTTGAGTTTGGTTCAGTTCGGTTTAATCTTAAAATTCGAACGGATTCAAAATTAGAAATAGACCAGTTTTTTTTTTTTTTTTAATTTCAGTTTGAGTAAACTCGACTGGGTTTGCCCGTTAAAACAAAGGGAAATAATAATAATAATAATAATAAGAACCCAAGCAATCCTTAATACACCCGGTCCTAACCTTAAGCTGTCACTTCCGTTTAGGCGTGTTGGCAACTTCTTATCGTTACTTGAGCTAATTTGCTTCTTCGTTCCATGCTTCCAACTGCTGTTGCTTCCTCTCTGCCTCCTTCTTCTTTCGCCGTTGTCAACTGCACAAGGTCAGGTTATACGTACGTGGCGCATGAATGGGACAGTTGTATTGTAATTTGTATAAAGTTTGTTTGTGTTTTGTAATTCAATTGAGGCAATAAACAATAGCTGATATTTATATTGTAATTTGTATGATGGTTGTTTGTATTTTTAAAAAAGACTTGTTCAGGAGGATGATGCTTGATAGTTGATTAATAGTATTTGTTTTTGACACAGTTAGTTGATTGGGATGTGATTTGAGATTTTAGAATTTTAGGTTACTACCTAGTGATTTTTGGAATTGAGTTAGTTGTAATTCGAATTAAAATTAAACTAGAATTTAGACCCGAATTTTTAAATCGAACTAATTCAAAATACGAACATAAACCACACCGAACCGAATAATTGATTGAAACTCAAAAAGGAAGAGTAAAATTTCCTTTTAAAATGTGGTATGGACTTTGAATTGAAGAGCTAGCAAAGCGGGAAAGAGCCTCCTCATTCCTCAACCTCTCCAGTTACCAAATCGAGCAGTCGCAGTCGCTCA

>Cs1g11580

ACTTTGTGGGAAGTTGGTGTCCAAATTTATTATGCATATAAACAAGAATTATTTACTCTACGAGCTGTCTTGTTGTGGACAATTAGTGATTTTCCTGCTTATGGGAACTTGTCTGGATGCTCAGTTAAAGGATATTTTGCATGTCCTATATGTGGGGAAGATACACAGTCCTGTAGACTTAAGAATGGGAAGAAAAATGTGTGCATGCGCCATAGGCGATATCTCCCAGAATCTCACTTGTTTAGAGATTTAACAAAGGCTTTTGATGGTAAACCAGAAAGAGACTTTCCTTCTAAGCCATTAAGTGGTGAAGAAACATTAAAAAAGGTTGAAGGGATTCAAAACTCATGGGGGAAGAAAACTAGAAAGCATAAAAAATTAGATTCTAATGAAACAATTTGCTGGAAAAAGAAATCAATATTTTTTTCTCTCGAGTATTGGAAACATTTGCATGTTCGCCATATGTTAGATGTAATGCACATTGAAAAAAATGTGTGCGAAAGTATATATGGTACATTATTGAACATTCCAGGAAAAGCAAAAGATGGACTTAATTCTCGGTTGGACCTTGTTGATTTCAATATTAGGAAAGAATTAGAACCTGTTGTTGAAGTGAATCACACTTATTTACCTGCTACATGTTATTCCTTGACTACAGTGGAAAAAGTAATGTTTTGCGAAACATTATTTAATCTGAAGGTTCCTGAAGGATACTGTTCAAACTTTAAAAATCTTGTATCAATGAGTGATTTAAAGCTTATTGGGTTGAAATCTCATGATTGTCATGCTTTGATGCAACAACTCCTACCATTGGCTATTCGAGGAATTTTGCCAGAACTTGTTAGATATGCCATCACTAGACTTTGCTTTTTCTTCAATGATTTTTGTAGCAAAGTTGTGGATGTAGAGAAGTTAAACCAAATACAAAAGGATCTTGTGGTCACAATTAATTTATTTGAAATGTATTTCCTTCCTGAATTTTTTGATATCATGGTTCATTT

>Cs1g23720

TTAAATTTTTTTGTGAGCTCATGACATTTGTATTTCACTAAAAAGCTAATAAATTGTAAGAGTCATTTAACAATAGATGAAAAATGAGAGAATAAATCATATTAATTATCGAGAGAAAATAAGAGGAAAGAGAAGAAGAAATACATAATAATATGTCATGGAGCTATTAATTTTAATTTTTTATCAATAAAAATAAAAATTAGTATTTAATTTTGTCTACTGATTTGAGTGGTATTGAAAGGTTAAGATTTTGTATATATTATTGTCTTCAAAAAAAGGCTCCCAATATTGATATATAACGTAAATGTTGTGTTATGTTAATATAGGAAACATGAGAGATACTACACCCATTAATATGGGTATGATACATGCAATTAAATTTTAAATAATCATAGTTAAATGTTGATTTTTCAAAGTAAACGCTGGCAAGTCATAAAATTATATTATTTGATTATTCTAGAATGTCTCGCAAAGTGGATTCTATGCAGTGCGAGTCGTGTTGTGTGGGGTGGAAAATATGAATGTCAAGTCAACATCCCTTGAATCTTCCCATGAGCAGATGAGATAGCACAATTACGTCAGTATTCTTGACTAACTGTTCGACTTTGATCTTTCGGTCGTCTGCGAGCTTCATTTTTTTTTTGTAGCAACTCTGCATCAATTATCAAACTGTCTACTTGACTGATTCAGACTAGTTTTTTTTTTCCTGAACCGGGAGATGGTCTTGAATGGACCTTAATTGTGAAAGATATCTTTAAGCCTATACCGCAATTCAGACTAATTTTTGGTGATATCGGGTCGGTCCGTAAGGGGTAAAGTACTAATTAAAGTGGTGACTCAATACGAGAATGAGACTTGAACCCTTGATCTCTCTTAAAGAATGAGAGTGCCGAATCACTCATACCAATAAACTTTGGTTATCCAAACTAGTTATTAACCATTGCAACTTAGTTATTGTCTCCATCCCAACATTAAATTGAGTCAATATTGGTTTACAGAAA

>Cs1g21000

CTAATTTGGCTGGACTGTAATTTACTGTAACATGGCCCTTAAAAAGGTAATAAGATACAAATATGATGTCACGATACAAATAAAATGTCATACTTCAGTTTTCTGTCCAAAGTAATGATAAGATTAATTAATAAAACTTTGTCGTGCGTATACATACGCATATATACAACTAATGTGTTATAAAATCTTATTTTCCCAGACAATAATTGAGGACGGAACTCAGCATATATATGTCGAGAGAAACATATATATCTATGTATTCACATCTAAGAAAAATATTATGTACAAGAATTTTTTAGTCACAAAAATCGGATTGCATTATTCGTAGACTTCTTTTGGGTCCAAGGAGAATGAAGGCCCTTTACCAACAAAGGACAGCATTTTCACACGTGTGATAACAACATTAATTAAGTATTAAACGCGGACGGTACCCACTATATAAAACATTGAAAATCATTCAATCCTGTCCAATTGGAATTGCACAGCTTCATCACTTCCTTTGCACAGAGGTAAAGCTTCCACTGACTTTTTACTCTTTCTCTTCTTTGATGAAATTCTGAATCCTGTGTATGATTGATTTGGGTACAGGTGTTGTACTCAAGTTAATTAGCATTAGAGCATATATATATGCTGCTTTTGTCCTTCAAATTTGAAGCTTCTTTCTTCGAGTTATAGTGAGAGATATCTTGGAATTAGTCTGAGAGAAAATTTGGTAATAACTATTTTAATTTCCCGGTGCCTCTGTCTAATATTTCAGGTTTAGTTCTAAAGTCCACCTCTTGCTTCAAATACACAACCAAAAGGGGGAAAAAAAAAGGCTCAGAACTATGGAATTGTCATCAGCTAAATGGGTTTCTGAAATGGTAAAATAAGACTATTATATATATGAAAGTTATTTACGCACTATATGAATTAAATGCGAAGAAGAGTATGGGTTATTTGTTATCATCAAAATTACTAATTTCTCTGATAAATTCTTTTAATTTTTTATTTATAGGAAA

>Cs1g17810

AGTATCTATAATGCAGCAGATTACTGGGAAATCTGTCTAACATATTACAACTGATTTCACTTCTATGCAAATCTGCCTTTCTATCTCCTTCTTTTCTTTTATACTTGATCCAATTGTTATCTGTCCTGTAATCACCCTTAGATTTCTTTATTCCCCTTCTGATTGTATTGTTGTCCCACGTACGGTATCATATCAGTTATGTATGTAAATTGATCCTCGCAGCTGTACTTGTCTCTACCATTGATTTAATATATTCATTAGCATGTACTGTTGCCAATCCTTTTCTAAAATAAGATATAGAAATTATATTTATCTATTTATTTATTTTTCGGAAAACAGTTATGTTGCGTTTTAGCTGTAATATTCTATTTCTTTTGGGTGCACACTTTGTATAATCTTTTGGATATTTTGGCGCATTTTTGCTGCTTTATTTAATTTTTTGTTTATCTTTTTAATGATACCCTTTTGCTGCTACAGGAGGAAGATAACGGTGTGCTTTATGCAAAGCTTTCAAGTATGGAAAATTATGGTTGGGGTGATATTGGAACAAATGCTGACAGTCCATGCAGTGATGCAATGCAGTGTGACAATATCTTTGGCATTGATCCATTTTACATAGAAAAAGGTGATCTGACAATTTTTTTAATAAGCATTAATATTTTTAAGTGTTAGCTTGTTATGGAGGCACAAGGTCTAGAAACTTGGAAATAATTTGACCAGATGGACCTCTGTTCCATCTCTCTATTATGTGTATGTGTGTGTGTGTGTACACAAAATAAATTCAATATTACTATTTTTATTCCCAAAATAAAAAATGAGATGGTGGCCATCAGTACTTTGGACAAAAGTGAAAATATGATAGTCTTGTATAGTTGCAATCTTTAACTTGTTTATTTCAACTGGATGTGATGGATGTATCATACTAATACTTATCAGGCAAGGGCTTCCACTCTTGTAATCATGTTTTCTAGTAACATATGGATTATGTCTATAGGTGATGA

>Cs1g26730

TAGAATCTTATAAGTTTCTCAAAAAAAATATATTTTAATTAAAATTTAAAATATAAATAGAAATTATGACATATGGTAGGTGCAAAAAATTTGTTGAAAGAATAATATATATATTTAATTGAATTTTCATTCAAAACAAAAAATAAATTATTTAAATAATTTCAATTAAAAAAGAGTTCTATGACAAATTTTAAGAAGTGTCGAAAAACCTACCCTATATAATAATGAAGATAGAGAGATCGGCTCTGGTGACTTCTTGATCCATACTCTCCACAATGTGGACTATTGGATGTTCAAACACTTTCTAAGACGTTTTTACCAATTGAGCAACTAACAATGAATGAAGTTGCACTTTTCAAGTAATACAATATGTAATAATAAATAATTTTAATTGAGTGACATTTTATTGAATGGTGAGATAAAATAATAAAATAGTTTAATAAATTTTGAATTGAATTGTCAAATGATTAATTTTCGGTGTATTTACGGTTATTTTTCTAAAATCAGAATGGATAAGAATTTGAATTGACTGAAATAAAAATAGATTAAAATCAGAATCAAAATAAGTAGTTTACTTGAGGTATATGAATCAAAATCAGAATGAGTTGTATTTTTATTGATATGTTTACTTTATCTTGTAATCGAAATAAATTATTACAAGTTAGTAAAATATCCTTAGTTGTAATTATATATTTGTTTAGTGAGAAAATAAAGAGTAAATATTTTTCTGTGATGATGATGATGTTTATTATTATTATTATAAAATTAATTATTACATGTACGGTACAGCAGCTTTATGGGAATAGAATTTTTACTATTATTTTTATATTTTTAAAACATAAATATTATTAATAAAAAATAAAAATTATTCACTAATTGAAGAAGTAAACACACCCCCAAATGTTAAATGAGCGCGTAAATTATTATTTTGGTAGCCTGCGCCTTGTGAAAAAGGAAATAACAATAAAAAAAAATATTATATACAGAGCCAGAGGTGTGGG

>Cs1g05430

TTACATCATTCTTTTCTTTCCCTTTTCTGTACAAACAAACACTTCTGGGATTAAATCTTTTTTACATGATTACAAAAGCTGTCAATCTCGCAGCAGTAGTACTCGCCTTGTATTGGAGGCCTGAGCCTAAATAAGTAAACAGCCTGATTTTGATGGCGATGAAATGCAGGCATTCTTGTATGACCTTAGTTAGACATAATGAGCTCCAATACATTTCGTATTACTTCATTTCGACAGGAGCATTAAGTATTGTTACCTCGGTTTTACAGATGCATTTGTTTTCTTTTCAGATCATTCTTGGTCTCCAAGTAGCGCATGCATGGAGACCACCAATACAGGGCTAATTCATGGCTTTTGATTCCCATATCAGTTGTAAAACAATGCTTGTTTGTGCATCACTCCAATCAGTGCCATCAATTCTAAATGTTGGACGCTATAACTGATATGTAGCATAAAAACTTGCAATTTTTTTTAAAAAAATTTTATATCTTAAATATCACGTACTTATAAAATAAATTTAAAAAAAAATTTTTTTTTCTAAGAAAATTGTCTCTGCACTACTTCAATTCTGTTGATGTAACTATTAAAGAATTATAACAATGTCTATGCAGCATCTAACTTTTTTTTTTCTATTATTTAGCCACTTCGTCATTACAGAAAATTTAATTTTAAAGAATTTATGGTGAAAGGCTAATTTGCCACTGGAATTTTATGGATATGATTTTACCAATGAAATGTAAAAAAATTATGAAGACAGTAAATTACCAATTACCCTTGCATTAAAAAATATAATGACAAACATGCCCGCAAAAGTAACTGTTGGAATTAAATAAATAAATAAAAAGCCCGACTACTTTTTAGTTACTTAAAATGTCTTGTTAATAAAAAACTGAAAGTTAAGTGCCGTGAAGATATTTTTAGAACTCTTGAGTGAGTGTATAAAATTTCAATGAAATTGAGGTGGTCCACAGATAATTTTTTTTCTATTAATACAAAATAAA

>Cs1g26470

TAGGATCAAGGGAGAAGACCTGAAAATTGCCTTTGACAGAGCTATTTGAGCTGTCTTTCGGTTAAACAAATTAGCTATGCTTACGTCACTATTTGATCTTCATGATTGTACAACAGGTCATTTATATGTGAAGAGTGATGTGTATGGATTTGGTGTTGTGCTGCTTGAACTGTTGACGGGTTTAAGGGCACTTGACACTAAACGCCCAACTGGACAGCAAAATTTGGTTGAGTGGTTGAAGCCAATGCTGTCTCAGAAAAAGAAGCTTAAAACTATAATGGATGCACGGATAGAGGGCCAGTATTCGTCTAACGCAGCGTTGCAGGCAGCACAGCTCACCCTAATGTGCCTAGAATCAGATCCTAAAAGGCGCCCCTCCATGAGAGAAGTTCTGGAAGTGTTGGAACAAATAGAAGCAGTGCCAATAGAAAAACCAAAGAACTCCATGTTTACCAGTTTGCATTCTGCACCTCATCGCCGTGCACAACGACCCATTCATCATCGCTCCCCACTTCATACAGGTCACCGTGGCGGCAGCGTCTGAGACGGAAACCGAATTTATGCTGACACAAAAGAGTATCGTTCCTATTTCTTAGAATGGAATTTGTTCTACCTACCATTATTCTTTCTTTAGTTGAATTAATCATGACAAGTTTTGAATTTTAGTCCTGGTTTGTGCTGAATGATGAGATGATGAAGTTGTAAAACTAGAGATTTCCCATTGTCTTGTCTGATGTAAATGTGACTGGACACTGGAGTGAAGTTGAATGGTCACGGCCAAGAAAATCTAATTAATCTAGAGTTAAAAGCAGAAGAACAAGGCAGCGCATTGTTGCTAATTTGCTGCAAAATTGAGAATATGAATCACATACATATTAACATACGTACGTACACTACTGTATCGAATACGAGATGTCTGGTCAATAATGACGTGAAGAAAGTCTTGAAATGGGCCTGCTCAGAAGAGTTGGACTTTGGAGCCCATACCATCATGAAGCGCT

>Cs1g04370

CTACATATATCTGTTAATGGTTTAATTTCTTCTAGTTTTAGAGTTCTCACAATCAAGTGCTTGGAAACAATGAGAGGTTGCATGAGTGGGGTCTGTGGGGATTGGGGAGATGGAAGTGGATTATCGCTAGGATCTTCTTTAAAGGGTACTTTAATCACCTTTTAACATTCGCATCAAATGTTTGTCATGTACGCTATCTATTAGTAAGTTATTTGTAAAATATTATTGGTACTTGTTTGTTAGTTTATTCGAGCCATCGTAAAGGGCCTTGAAAAGGACAAGGCAATTTGCAAATCACACATTAGACGGTTCTGCTTGAATTGCACGTCCAAAAGAGGAAGAAAAATGGAAAACGAAACCAATATTATATTCACTAAATGGGAGTCATATTTATTGGAATTTACTTTGTTGCAAGGATGGCAACAAATGTAATCATCGTTGATCTATGTTGAACAAAAATCTTCTACAATACATAATCTTATATGGAGTAGAGTATATGAAAATTCGTATTCGAATTTGAGTAGTTAGCTAAATAAAGTTATTAGATAATTATTTGGTCGGGCTTTTAGGAAAATTTGTTGTTGATAAAAAATTATTAAAAAATTACTATGTTAGAGATGTTCTAAGTCAGAGCGTTTTTCATAATTATTAGATTTAAAGTTGAAATGATTATTGGTTCTTTCAACTTTAGATCTAATGATTCTAATAGATGCTTTAATTTAGAGCATAACTAACGTAGCTAGACCCAAAATTGTATGTGTGAGGTTCCAATTTTACACACGGAAGGACTTTATGTTTATGAAGATATATTTCAAAAAGGTCCACTTTTTCCAAACTAAATGTGGATGTATGTAATTTAACTAAACGTTAAGTAGCTAAATGAAAATTTACCCACAAAGGCCCAAAACAACACTCACAAAGCCCGATTATATTGTCCGGCCCATGATTCTCCACTCAGTTATAAAATGGCGCACGCAAACGCGCCCTTATCCGTTTCGTAA

>Cs1g06130

TAAGAAGATGAAAAAGAAAAAGAAAAATAAAGGGGGCAGCAGTAGTGCTCGTCCGAGGCTTTTGGGTATTTTGAAGGCAGTTTTATTCGAAACTTCTTTGGTACGTATATAATATATAATAATAATAATTAGTTAATTTCCCTTTTGTTTATTTATCTTGCTCTGAATCAATAACAATAATCAGGGTTAAATTAAATTTGTAATCATTGCAGGCGAAGAGAATCAGAAAGAGGAAAATTAAACAGAAGACGTGCCAAACAAGCAGTGGATCATCAAATGAGGAATCGTCGATGCTCAAAGAATTTGATCAAGATGGAAGCATTACTTCATCTGCTTCGGCGTGTACTTCCTCGTCTGATCCTACGAACAAATCCCTTCGATTGGAGTGTAAACAAAAACAACAACGGGACGTTGTTGTTGCTGAAGCTCAACATCATCATCATCTTAATCAGCATGGGAGAGGGTGTTACGATTCTTACGTTGGCTTCTGTTTGATTTTAATTAGCCTATTGATACTGATCGTCTGGGGCAAGCTCTGCGCCATATTCTGTACTTCCACGTGGCTTTTCTTGGGGGCACGTTGGAACACCGGTAACCAATCATCGGAAAATATGCCTGTCGATTCCAAATCGTCATGGGAGGACTGGTAGAAAGGGACCAATCTCGCGGTCTATACACACCCATTTCGACATTTTGCTTTAATTTGCCAGTTTGGTACCTTTTTTTCAGATTTTTGGTTCATGATATGTATAGCTTTATATGTTATACTTATATGTAAGTAAATTAAAAAGGATTTAGGGTTCCTGTTTTGACTTTATCTTTTTGCAATCTTTTAAGTACGTGTGTATATATATGATATTGAGGAAATGAAAATTGTACAAACATTTTGGTGGTGGCACGAAAATTATTTGAATATTCAAAGAAGCAAAATGATGCTGCTGGTTAACAAACACTGGCACATGTTGTTTTCAGAACTTCGACTGTTTTGGTGGGACGATCAT

>Cs1g06230

GGCCTAAGATGTGGAATACAAGCACCGTAGTCCATGGATACAAATTTCATTATTACTGATTTATTAAACAATAATTGAGGGTCAAAGCCCTCCAATTGTGAAATGTGATCACATGAGAATTTGCCATTCTCACTTTGTAAAGCAGCCTGCTCTTAACTTCCTCCAACCGTCGACTGAACATATGCCGCTCGCATCACTAAATTCAAAATACGATTGTGACGCGTCACGCTATCATTGGTCCTTTCCCATTTATTCCCGCGATCTGCTTTAACGCTCATGAGCCCACAAATTCCCCCTTTTACATCTGGAATCAAATTATTAATAATAAACTATACTGGAAATTTATCACTCATTCCCCTTGAAATTTATGATATTCTCACAGAGATAGCATATTTCATTTAATAGTCGCTTAGCTCCCATTGAACAAAATTGAGGACACTTCAATAACTTACAATGTGCTCAAGATAAACACGCTTCTGACATCCAAAGTTAGCTTCTTTTTTTTTTTTTTTGAAAAATCAAAAGATTACGAACAAGAAATTGAATTCTACACTTTTATAATTTTCTTTGGTCTCAATTTATAATAGAAAAAGATTTATGAGTGCAGGATTGATGTTTTTTTTTATCAATCCAATCCATACAATCAGATGCCTATTGATATTTACATATGATATTTATTGAAACCACTTAGATTACTTTTATTATGAGATAATTCAAATTTATATCTCATATACTTTGAATGTTTTATTTCACTTATATTTTTTATTAGAAGAGGAAACAGTCTCTTACTGGACTCAAACTGCTACTCTCACAATTAATCCCACTCGGACACTTGACAATTCGACATGCATGAGAAGAAAAAAAAAATTATTTTGATAATTAATTAAAAAAAGGAAGTGAATAAATCCCGGCCATAGAAGTTTAAATCTGACACGTCTCATGAAAAAGATTAGAAAACATTCTTCCTCGCTGGTTTATTTTAATACAGATCTAGGGGAGCA

>Cs1g12760

AAAAATAAAAAATAAAAAAATCATAAAAATACAAAGAATTGAAGATTTTAAATAAAAAAACAAATTGAACTGGATCAATTTAGTTTGGTTTTGTTTGATTTGTTGGGCAAAAATTGATTGGTTCAGTTTGATTCATAACCTAATTAGGAAAAGGTTTAATAGCTTTTGAAATTGGTCTAAGTGATTATGAAATTGGAGAAAACAAATCATGATTTTAATTTAGAATAATTTTGTTTGTTTATTTTTTGGAATATTATATAATTAAAAAAGAAAAGAGGAAAAATGAAACTAATACCTTTATCTGAGAATGAGGTTAAAATATTGAAAGAGGTTTATTTCATAAGTTTAAACAAAAATAATGTTTGGTCAAATTTTAAAAAACTGCCTTTTTCATAAATTTATCTATTTTGATCATATTTCTTAAAAGTAGTTAATTAAATAAGATACCATATTTTTTTTAAAATTTTTTATTATAGTCTTAATATTCTTTTTTTTTAAAGGACAATTATATCTTACTTTTTTAGAACCCTAATACACAAATAAAAAAGTGATTTAAACTTATATTCATTATGGTAATTATATAATAAAATTTATTAAATATATATACTACACATTTTAAACTTATAACAATAGCAATAACATATTTTATCAAATACTAAATTACTTTTTAAATAAGTAATTTCTTTTATCCACACAACAAAAATACCTTATTTCAATTGCTCATCAGAATCCCAAACTGACCTTGAAACTATTTATGCTGGAGTTATTTATAGTTGGATAAAGAGGTTCATGAAAAGTGGTGCTGGAAATCGTGGGATGGTAACGTATTCATATAACCATTCGTTACAGCTTACATGACACATTTTTTAATATTAAAAATAGACTTATGATGTCATTTTATTCTTTATCTTTTTTATGTTTGGGGCATTATTTCACGTTAAGGGTATAACTAATGAAATGTAACAAAAAAAAAAAATTGTAAAAAAACTATTATATTTCGA

>Cs1g11550

TAGCCATTGTTTCTCTAATGGTTGGGAATTGGCAGCCAATTTTTTCCCCCTTTTCCACGTATTCATATGTAAATTTGATCAATGTGTTTGAAATGTTTCTTCCTCAACTTAACTTGTATCCTAACCCATCTACTCTATTGAATGAAGAAGCTCCTGGTGTGATACCGCAGTTGCACTACCTATGTTTAATTTTTATGTACTTAATTTAGATTATTCAGTAATGAGAGTAGGATATCTCGCATCTAATTTATTAAAGTATTTAGAATTTGTAATATATACTAATAATATTTAAATATGAAATGAAAGATTTTGTAATATAAATTGATTTTTTGGTTATTTTGTGGTGCCTTTTAATTTTGCGAATAAATCAATACGCAATGATCCAATTCAATATATTTTTTTAAATAAATTAAACAGTGATACCATGGTAACAGTTATCATAGACACTAACAAAATATCAGCAACTACTAACACTATGGTGTCGTAATATTGGTGATATTGTAGTGATAGTAATCATTGACATCGTATTCGCGACACCATAAACAAGTGTCACTATTTTTCTGACTGTCAGATTACTGACACAATTAACAAGTGTCACTGAAAGTAATTACTAACACTATTCTAATGTCGATAAAGACCCTTTCTTTAGTAGTGCTTTCCTCATGGATATGGATTTTCATTTTATAATTTATGGGAAAGTGAGTGAAATCCATGTTTGTCTAAACTAAGCATTAACCCCCTTGTGTTTCTCTTGGAGACAAACCTTTGCGCTTTGATTTTTAAAGAGAGAGAGGGGGAAAAAAGTTAAGCAAAGTTTGGGAGTATTTTGGACTTGAATAAAAATTGTTTTCAAATAATCAAATTATTTTTTTTCTTTTTGCAACTTTCATTGTTATGCTTGATTGAAGATTCTTTTTTAAAAATATCATTACATCTATAAAATCTATCACAAATTAATCAAAACGTGCAAACAAAAATATGTTGGGATTTATATATCTTGA

>Cs1g04270

GAAAGTTTATAGGTTCTAAGATGTTTTTCCGTTGGAAATGAAGATTTGACTCTTTCGGTGGAAATGATTTGAACATTAGAAGCGACACTTTTGATACTTTTTTTTTTTTTATGATTTGGACATTATTGGTGACCAATTTGGGAGCAGTTGGTCGGAAAATGTTTTTGAGAACCACTACCATCTCTTGCTGGCTTTGAGAAAATTTTCACCAAAGTGAGTGTTGAGTGGCCTACTGTGTGTGGTGCCTAATCTATCATATTTATTATAAGGTAGGTTTTTTTTTAAAAAAATTTAAATGAATTTATTTTTTAAAAAAATTTGAATAAGTCGGAATGCAAATATTTAAATGGCATATTTGCTGAGGTTCATATTCGTTGGTTGGTTAGATCAGGTGATAGTAGTTTCTGGTATGATTACTGGCTTGAATTTACACCTCTCTTTCATTTTAATCCTCCAGCAGCTTCTATGGCCCCTATATCTTCTTTTTGGTGTGGTGTGGATTGGGATAAGAAGAAGCTTTTATTAGTACTGTCATCATCAGTTGTTGAACAAATTGTGTTAGTACCTATTTCTGGTACTGGGCCAGATATACTCTGTTGTGCCATATCTAGGGATGAGAATTTTTGTCTAAGGAGTAGTTGGGAATTTGTACATGGGTCCCGTAGCCAGAATGAGGTTTTTTCTTTAATTTGGCAACGACATATTCCTTCGCAGGTTTCTTTTTTCTTGTGGCGTTTATTGAATGGTTTCTTGGCCACGGATGATGCTTAGTGCTCTAGGGGTTTTCATATGGTTTCTCGGTATCTTTGTAATCGAGATGTTGGGATTGTCAGACACTTATTTTTGAATTGCCCAGAGGTTCAACAGGTTTGGCGGCGATTTTACTCAATGATTGGTCGTCCGTATCTCCCTTTTTTGTCTCCCCATGCTTTGCTTAGTCACTGGCAACGGTGTTGCTGTTCTTCTAAACATATTCGAGTCATTCTTCCATGTTTCATTCT

>Cs1g14970

TAAAGCTTCTAATTTTATATAACTGCGATGTGTTCACAAACACATGCGTTGGTTCATACCTCTTTATTATTTATGCTCTTGTATTACCTAAGTCCCACTACTGTGTTACTGTTCATTTAAGAGTAGTAAGTTACAGCTAGCCGTAACTTAGTTGGATCCTTTAAACTAAGGTTAGTGTACCCTAAAAAAATAAAAATCATAAAATGAATGTGTTTTTGTGATGTAATCTTGTGAGAGAGAATGGTAAAACTCTTCCCATCATGTGGAGGGAATAAATTTGAGTTTCTAACAATATAAAGTTGATATTTTATTCGAATCAATTCGGTTTAGATAAGTTTTAGTTGGATTCATAAAATTCTGAATCATGGATTTTTTTTCTAATTAAGTTTTAAAAAGTAACTAAAATAAAATAAAATAAATTGACTAATTAAACTAATCAGCGAACCTAATTTGTATTGGTTCATTTTAGTTTTTTTATTTAAATTATTTCAAAGAATTCTAAACTGAACCTAAGGTTGTTTACCCTTGTACACGCTCTTTTGTAAATATTTATTCGTAGGCATGGCATAGATCGAAAATTACTGTGAAGTAAGATGGTAATAGGAAAGGTAGGTGATTAATTTTGAACTTCAACTATTAAAAATATTAACAAATAATATAAGGGGGGTTAATAAAATTAACCAACAGTAAAAACCACACGTATTTTGTGATTTTGTCTTTAAGTTTCGGTGTTCTGGCACTTATACCCTGGAACACTCAATTCCAAGACTTCATGCTCTTCAACGCTTGGAAAGGTGTCGAAATTTCTAGACTAATCAATCAATTCCCCACCGACTAACCCCACCTTCGAAGCTCTTCAGAACCTTCTCGAACTCGCCCCAATCCTCACCCTCCCTTCAATTTTATAAACCCTTTCTACAAAAAGAAACCCAGTACTCGATCAAACGATTCAATAACTACGCTTCTCGCATCAGTGAGACATTTTTACAAACGCCTTTGCA

>Cs1g20130

TATATTTCAAATTGGAAAAGAAAAGGAAAAAAAACCCCTCAAATCTCGTGATCCGTGGGTAGGGATGCCAACGGCTTAATTACTGTGCGAGAATGTATATGACTTGACTGTACCCGTATTTATTTAGTAGAAAATACCTTCACCATCAGTCTTTATCTGTCGTGGACTTAATGGATAAGTCCGAAATTTTTTGATAAAAATTATAAGTACACGGTTATCTTCGAGTTGTTATGAGGTAGAACACGAATTTTAATTATGTATATAAAGTGGTAAATAGTAAAAATATAGAGTAGTTTTAACATGGTCGGATCCAAATTATAATTACCGGTCTCGTCCCCTATCCCTATATTCCTTTTTTATTTTGTAACTTCAGCATATGTTTGAAAAAAAAAAGAATGATATCTTAACTATATAGCTATAGATGCATGCAACACTTTATTTTAAAATATTTAAAAAAAATTATTTTCAATCTCAGATAAATATTAATTATAATTTAATGATTGTTAAATTTAATTTTAATCAATGTATTATTAAATATTTAGATCGAAAGTTAAAGTCCCCACTTTAACATTTCTTTATTCAATTTCAACTAATCTTAATTTGACTAGAATAATTTTTTAGATGGTTTAATAAATATTTAATCCATTAAATAACTAAAAAATCTTTAGGCTGTTGTCGTGGACATTATTATATTGGTTTTTTCCCTTCAATGATTAATGAATGCTTCCCGTGCATGCCAGATGGGCTGTGAGTGGCACCTCGCTATTTTATTTTATTTTTTATATGACAAAAGAGATAGAACTCTAATTTGTTATGACCACAAAAGATATAACTCTATTTCAAAATTACATGTGGCTCGTTATAGTTTCTTCAATAATTTTAAAGGGATCTCCTAAGTTAAATTTTATGTATCTTATATTCATTTATATAAATAGTGCATAGTGTGAAACTTACTTATTATTTATATGAATGAGTATAAGATAGGTGAAATGTAAGTTAGA

>Cs1g05910

AACTCAATTGGTTCTTGTTCATCGTGCTTTGGATTATTTTCCACATATACCAGAACAGTCTCTGACTTTTCCTTTATAGTGCTATCATTTTTATCTTTCCTTTGTAATTGATCTTCTATAAAAATAACATCTCTGCTGATGATGATCTTGTGGGTAGTGGGGTCCCACAAACGATACTCCTTCACTCCATCAGCATATCCCAAAAAAATACTTCTTCTAAATTTTGGATCCAACTTTATTCTTTCTTGGGCATACATAGGAGAACTATACATAGGAGAACCAATTAAAAAGAACTAATTAAAACTAATTGTCCAAGGCCCCAAATATGATTAAGCCGGCCCTGGTCTACTCCACTTTCCTTGTACGTATTTCCCTTTAAGGGGGCGGTCTTTGAATTTTAAGTTATATCAAATAGAAATACTATTTCACCCTTAATTAAGATCCAACTTCTACAAAAGTAACGATACGGGTGCTTTGGGTAAATCTTAAAATATATGAAGTAATTAACTCGAATTGGAGAGTCCAAAGGAAAATTTATCCACATTTCACATTCCATGCACATTATGAGTTAAACGTTTAATAGAGTATTTTTTTCTTGAGTTCTTGTAGTTTGTGAAAAAAAAGAAAAAAGCCCGAAGATAAAAAATTCTTTAAATGCATCACTTAAACCTTTCGAAATTAAAATTCATTTCATTCGTCCTTTACTGAACTTTTATCGTAGTCCATTTCAAACTTGTTTACTGATTATTTTACAATTGTAAAAATCTAATCGCCTTCGCAGTAATTATCATCGCAATATCGTACGGTCGCAGATTGTCAATTAATTTACTGTTGATTTTTTTTGTCTTTATAGAGGAATCGCACCATCTTTTGACTTCACTTTTGTCCTTCTTTCGGCACATTTATCAGTTTCCTTAAAAAATTTGTCAATGTTTGAAATATCGCCACCCAGTTCACACTGGCGGACCCTCAAAATCGCTCCAGAATTAATCAAATACAAC

>Cs1g03250

ATGAGTATATAGATCGTCTCCATAGGAATTGATGTTATAGGATTTGGTACAACAATCTTAATAGTGCATTTCAATCTAAATTAAAATAAAATAATTAATTAAACTAATGAGCTAGCTAAATTAAAAAAAGCAAATAAAAAAATGCAACAAGAAAAATCTCTTACTTTAATAAAGTATGAGATTAGAAAATATAAAAAATACATACTCCAATGTAGTTTATTTTCTGTTATAGTGTATTGAAATTTGTGTTTTCTACCATTTAACTTACCGGTTACTTTATAAAGTAAGGAATTCCCTTACTAGAGGGACTACGTCACTAGTATTAGAAAGTCACACAATGATATAATGACAATTAAAAATATTTTCATAATTTTACTGTTTATTTATTTCACAATCATACTTATTATTTTTCTTTCATTAAATCAAATTGTTCAAATATATCGCATTCTCTATCGCTAAAATAAAGAATAATTTCATATTGTACAATTTTAATAATTTATATTATAAACTTATTTATTCTCATTCTTTTATTAAATACATGTATTTTTACTAAGCGATTTAATGCATCGTTGCTCTGTGTGTGTGTGTTTGCACGCGTGTGTGTTGTTTACTCTTTTATATGAACTGTCATTCACACAAATATTTTATATTTATCATAAATTCATAAACTAATCTAAGATAACATTAACAACCTGATTTATGTTTAAATTTTAATTGCTTATATTCTTTTTAAATCAATTATCTTTTTTAGATTATTCGATTAATGTCCCCGTGCTTCGATGTATAAGACATTAAAATTAGTTGCGATCACTTGGAGTGAAGGCAGAGGCGGCACATGGTCCAGGTCATCACTCACATAAAATAACACAATCCGATAGAAGTAAATGTTAACGTTATGCTTAGCTTGATAGATAGATAAACAAATAGTAAACGAGATCGACAAACACGTGAATAATAAATAACACCATTATTAATTAATTAGTGAACTATTTGGATACCCA

>Cs1g16860

CAATATGATTGCTGTGGTAAGAGACGGTGCAGTGGTGGAGTATGGCAGCCATGAGACGCTCTTAGCTTCCCATCTTAATGGTGTTTATGCTAGCTTGGTCAGAGCTGAAACTGAAGCCAATGCATTTTCATGAGTAGTTGATTTTTGGTCTAACCGTGTAATGGATCATATGACCTAAATATAAGCATTATGAATATATAACACCTCCATAATTTTGCTCATGCTAATAATTATATGTATCTAAAGAATAAGCAATTGTATAAGCTAGCATATAACTGAATGTAGATACTATTGACAAGGACTTTAAAAAATGGGATATTAAATGGCGTGGTCCAACTTGTGAAATTTGGCATGTTGTGAAATTGGTATGCTTCGAATCAAATCCTATCTACAGATGTAAAACTGGATTGGGTATACCAAAAGGTATAAGATAAGCTTTCTATTTTCTGTTGAGTGTGTCCACAGGAAAAATTCTAAGCTTCTACTTTTTGAATGTATCTCCGCCTATTAATCGTAAACTAAACAGCCAAAATTAAGTCCTTCTACCCGTAAGAACAATCCTTTCTATTATCTATTTTAAATGAGGCACATGCAAAAATTTTGGTCAAGTGAGAAATTGCAATTTGAATGCAGGAAAGGTACATTTCGACATTCTCACCTTTCCTTAAAACTCCACTCCCTCTAACTTAAAAGGGAAATGCGACCAGGACGTGACCCACATCACAATGACTTAAACACCCCTTTGAATATAAGAATAATGCTCTAACTTCAATAATCTTACACATGGGCATATATGGGCATATGTGTCTCTTCATGTCCCGCAAAGGTGGGCACAAAATTCTCTCAAGTAAACATAGTCTCTTCGCTTTCAATCTAAGAGAGGTTGATGGTTACGTGCCTGGCATGCAAAGCTATAGAGGTTAATTCATTGCTTCACCAACCAGCCTGTGCATTTTGTTTGCTGAAGCTAGCTGTTATTTTTCCATTCTTCTCTTCACAGA

>Cs1g18520

TATGTCTGTCATATGCTTTCGAAGAAATCAATCACTGTGGCCATTAATTCTTACCATTGTAGGCTATTAAGTGAATCCTACTATGCTTTTTGTTAACTGGAAATTCAATTGCATCTTTTCATATTGCCATATTCATTCAGTTAAGATCATTTTCAGGAAAACACTTTCAGGAGTGTTAATTAATCTTGTGGATAATTTATGAGAGAAGCTGACAAATTCTGAATACAGCAAAAGTAAAAGAGCACTAAATCTATGAATGGCCTTTCAATTTTCAAGATAATTCAAAAGCTAATTTCAATGGAGTGGTCTATTTCTATTTGATATATACCTTTTGCAGACAAGTTTCTGTGTATTACTTACTCATCAAAATTAAAATTGAAATTTTTTGGGCATAATATGCGTAGATTACAAATAATTATGTGTTAATCATGTAAGCAATCTGGGTTATAATTAAGTTCCTCATGTATATGTAAAACATAAGAAATAAAATGCAAAAAGTTATATAAATTTCCACCGTACGTCTATTCAATATACATAATTGGGTAAGTCTCAGATTACTCCACCCCTGTGAATTGATTATCTTTTCATATGTCATGTGCAATTTGAAAACAACCGGCGTGCTCTGACTTATTTCTGTGTACGCATGAATTTTTTAAAAAGAAGTCATACGAGGAGAGATCCTGATCGTGGTCGAGTTGATTGAGCAGAGATCCTAATCGTGGTCGAGAGGGTAACCAGAATCTCGCATCAATAAAGAACATATCTTTAATTAAAATTTAAAGAATAATACAAATTATGAAATAAGATAGTGTTAAATTTTTTTTCAAGGAATAATAATATTTAAATATTTACTTGAATTTCTTTGGGATAAAATTTTTAAAATATTTATCATTATTCATAAATTACCTCGATTATTTAAGCAGTTCGGAAATTTAAAGGGGGTGTCAAGAGTCGAGACTCAAAAGCCTATTTGATGACAAAAGATTGCGTCATCCGAAGTT

>Cs1g10280

TATATTTGCTTATCGCATTAGTCATTGCTTCAAATTCTTTAAAAAAAAATTGTAATCCTCGTCATTTATCACATTGGTTCAAATTCTTAATTTTTTTTTATTAAAAGTTCAAATTCTTAAAAATTTGTGTGACGTAAAATAAAAAAATTGTAACTCTCGTTAATTAAACAATTGAAATCCAAAATTTATATTATACATAAGTTAGTAGATGACATGATATCATCACATAGTTTCATTATGATAACTACTTATTAAATCAATTTAGATTAGTTTAATTGGGTCAAACGACAATATTACTCTTACATGGTTGAATACATATTTTGTATAGGTAATATAATCCACCGATCAAATGATGACACATCATATCCAACATTATTTGTGATACAAATTATTTATCCCAAGTACGTATATGTAGCATCGCTTGCAGCCAAGGATCATTTTGCTACCAACTATGGTGATTCTAGAAGATTTGGCAAATTAAATGGATCATTCTTTCATCTCTCTTTAATGAAATAAGCTATTTCTTAATAATTACTTCCCGTATGATTCTTTGCCTAGCCATCACTTGCAAGAGTCGTTTATTGTTGCCGTCACATAGTTTGAATTCTGAATATTTTATAAAGTCTATTGAAATAATCATATTGTTTGAAGATTTAACTATTTACCGCCCACTGTATCAATTTCTGAGTCGGCCTTGGAATGAAGAGTCATAATTTGACTAGTATGGGTCACACACGTTTTACGTTTCTTCTAATAATCAAACCAATTCTTGGAAACGCTTAGAATGCCAGGTCAATTTTGCCTCAACGGACCTTGCTCAAGAAGGAAAGTAGATGTCAATTATTCCAAGTCATGGCCAGAAATTATGTGCCACTATAAATTCCGCGAGGCATCTCACACAAAATGCAAGAGGAGGCCAGAGTCTTGACAAACACTCCAAAAAGCACTGTTTTTTATTTGCATCCATAGTTCCACTTTTCAATAGGCCAAAAATCAGAAAA

>Cs1g01170

TCACTAGTAAAATATTTCCTTCTGTCTTCGCCCGTGGATGTAGGCTAAAAGCCGAACCACGTAATTTCTGGTGTCCTCTATTGTGCTTGTTCTTTATTTTCTTCCAATTTTATTTTAGCTGCATGTCTGCTTCACCCACCAATTTCCTAACAGTGGTATCAGAGCTATTGGTTGTATTTTTGGAGTCGGGAACTGTTCACGTAAGGGGTACTATTCACGTATACGGCACTGTACACGTATAAGACACTATTCATGTATACTGTATTGTTCACATATACGGTACTGTTCACGTATACGGTACTATTCACGTGAAACGGTGGAAGCGATCCAAGAATTTTCCGGTGCAAAGCAGGGAATAGTAGTGTGAGAAAAGCAACTGTGGTTTTGTACATGTCTAGGAAAGTTCTGTCACAAAGGCGATAAGAGCTTAAGGAGTCTGGGTTTTAAGTGGGACCATTGTGACCCCTCCAGTCTTTCCTGGGAACTTTCCTGGTGTGCATTCTCACACATACTCACAACTATTCAAGGTGGTATACTAGCTTGTGTGCAGTATTTATCAACAAAATGGCGGCAAAGTATGAAATTGAGAAGTTTAACGAAAATAATTTTTCGTTGTGGAAAATGAAGATGAAAGTTGTATTGAGGAAAAATAATTGTTTGGCAGCAATTGGAGAAAGGCCCATGGAGATAACTGATGACAAGTGGAACGAGGTAGACGGTAACGCCATTTCTGATATACACTTGGCACTTGCGGATGGAGTATTATCCAGTGTGGCAGAGAAAAATACGGCGAAGGAAATATGGGATACTCTCACAAAATTGTATGAGGCCAAGTCACTACACAACAAAATTAAATGAGGAGAGCAGGCGGAAAAATAAGGAAAACAGACAAGTAAGTTCGCAGCAAGCGGAGGCGCTATCGGTGACGAGAGGGAGATCAACGGAACGTGGCCCCAGTGGGAGTAAAAATCAGGGTAGATCAAAATTCAGAAGTAAGAAGA

>Cs1g19130

TCTACATGATGAGCGATCACCTGATTATCGTGGAGGTACTCTTCCAAACTTAAAAAGTACAAAATTAATAAATTATACTTAAATTTGAGCACGTGTAGGTAATGTAATAAATTTACTCCACAACCACCAGTCAAACGCAATCTTTGTTTCCAATAGTACCATGCAAATATCACCCTCGTGCCACGCGCCTCCAAAGACGGTAATTTTACCCCCCGCTCAAACTCCACCGTTTGTCCGTACACAAGCTATTCATAATTGGGGATAAGAATATCAAGGTCCCCAAGTTCTTATTTTTTCTTAAATAAGATATAATTTGTTCTTTTATTTGTGATATGTATTTATTTTATAAGTTTTGGTACGAAAAAAAAAAAGAATTTATACTTACAACATAAATAAAAAAAATTTTAAATTTTATTTTAATTATAAGATGAGAAGTAGAATGTCACTAAATAAATGAATGATTCTCTCTTAAACTCTAATACTATAATGTGTCGTGGTTCGATATAATAAGGATACAAAATCGAACAGGCGAACACAGGGACTTATTTGGTATAGATGGATAAACTATATTCTAATTCGAGAAAATGAGAAAGTGTTCGGAAAGCCAAACGTTACAGCGTCCTACTTAGACAATTCTCACAGCGCACTTCAATATGCGCGTGGGGATTGGTTTCATCGTACTTGGAAGTAAAGAAAAGTAAAAACACTTCGGGTTTAAACAAATCTTATGCGGATTTTGCGAGAGAAGATTGGCCATGTTAAACATAAGTAACATGTGGTTATATATAAATCCAGATTACACTCAGACTCAGTCTTGCCTGTCTATATTTCCCCAAATAGCCGCTAAGATTTGGCCACGTCAGTATATTTCCGCAAGTTTCTTGGGGGATCTCTCTGTTGTTGGCACTGTAAAAGAAAGTACTGTAGAAGATAATTGAAGGGAGTCTCAGTTCGATTCTATCCACTTGGGTCCTACGTTGTCCATATCTCATTTGTTTAGC

>Cs1g24040

TAAAATTTTTATAAAATCTCTTTTCTATAATTTTAACCTCAATTAAACAAGGTTTAAAGTTGAGAGAAACAAAATCTAATTAAACCCCGATATAATCTTCCATCGATTAAAATAGGACTCTTAGTTGTGGGTGCCCAAATTTAAAAAATTCTCAAAAGAGCAAGAAGCTAATAATTTTTGTATTTTTTTAAAGCTCTAATCCGATTTTGCTGATGAAATTGAGAGGCAGATCACTTAGTGATAATGCCTCGCCTTCTTTTTCTTTCTCTTTGGTCAGAACCTTGCATGATTTTTATCAAGAGTTTCTTGCCTTCTGTAAGTTATTCTTTATTCAAAATTGAATTTGTTGGAGCCACAAACAAAGAATAAAGAGAATGAAATTTATGTAATAAGAGATTATGGAGCGATCTTATCTAGTTAATTATTCTTTTGAGAAAACTAAGCTAGTGAAAACGTTGCAAACTCCAATAAGAGATTATAGGGTTTTGTTGACAAATAAGAAGGTGAAGGTGTCTGTGAAATTGAAATGGGTGTACTCAAAGCAGCGTAATTTTCAGATATGTTTTTAAAAAGTTGTTTGTGCAAAAGGGGTTCTACAAGTACTTTAAACTGGTGCTAATAGCATCACCCGATGACAGTAGTAGTTGGGCCACCATGAAGACTGAAAAGTAAAAGAGAAAAGCTACACAGAATTACAGATCTCTCTATACATATCTTTGAGTCACGTGGCTACATTAAGTACATGCCATTTGTCTCGAAATGAGAAGACATGCAAATTTAACGCTACATCTTTTCACTCCATTGTCTTGACACATGGCATGGGTGTTGCTACGTGTAGAACCAAGGAGAAGCGAATTTTTGGTAGTATTGTCGTAAGAAGAGGATCAGAAACCAATTGATTTGCTAAATGTAGCCCTTGCAGTGGCATATTTTAACTGGTTTATCTCACAGTTAAATTTATATATATAAATACACGATCTCTAGAAACCGCAAGAATAAAA

>Cs1g08550

TGCTTATGGCCTTAAGCTTCAGATCAATCAATTTCAAAGACACTTACATTAATGATTATAATTTGATCACTGCTCATTAATGTTTCAATATTAAATTACGGTCTCCCTAAGTTATATCTAATCAAACTTACAGTAAGATAAAAATTCACTATATATTAGCCCACTTGGCCCTTTTGCTCCCTTAATTCTTTTTGTATTGCTTTACTTGATTGTAAGTTACAATCATTTTAACTCAGACAAACCCTAAAGTTAATTGCAATTGGATGCTTTTTATTAATATATATATATATAGCACGAAAATATAGTTGGATTGTGTGCTGTAATCTGAATTAAAACTCGTTTGTTCGAAACTTTTTATAGCTACTGTTGGTGAAGAAAACTCATTGTGCGGGGTTAGACTAAAGATTTTTAGATATTCAATCTAAAACACTAATTTATTAGGCGGAAGAATTTATTCTAATTATAAACAGTTTATTTGAAGGTAATTTTGTTTTTTAATTGATTTTGTCGGTAAGCTACACTACAGACTAAGTTACCAAAAACTGCAAGTTACGTAAATGATTGGATAGTGAATAACAAAGAAGAGTTAATTAATTAATTAACATTTATGAATTAATTAGACGTGAAGTTTTACCTGTAAATATTGAAATGAAAGAACGATAAGTATGAAAAGGACTCCTTCGTTAATTTTATAAACTTTTTATGGGAGAAATCAATACATTACCAGAAAGCAAGAAAACAGTTTGATGTTAGCTTTGAATTTATAACAATAACAATAAATACAGTACAAGCCCCATTTTGTTTTTCTTGCCATATCCGCCCACCGCTCTGTAATCTCTCTCTCTCTCTCTCTCTCTCTCTCTCTCTCTCTCTCTATCTATCTATTTGTTTATATATATATATATATGCACAGATGACATGTATATAAATAGATATAGGTAGATAGAGTAGAGATGATATGAAGAGTAGTGGTGTGTGGCCAAAATTCAGATAAAAAAAAA

>Cs1g03450

CCTCTTTCTCTCTCTTAGCGTTGTAAATATTTGTTATCGTTAAAAAAATAAAAAATAAAATGAAAAATGACAAAAAGTTGAATGCATCACTCTCTCTCTTGGCATTGTAATAATTTTATATTGTTGAAAATAATCTCATATTCCAAACCAAAATTCATCTTTATCGTCAAATAATTATAACCAAACTTTACAAAATTAACCCAAGAATGCAAAGAAAACAAAAAGTTTTCATTTTCTTTAATCACTGACCATAAACAATTGGGTTCTGTTATTTAAAACGGGTAGCGCTTTCGTGTTTGGTAGCTAGAGAGATTATGGAAGTGTGACAGGGAGCCGGAGAGTGAGGTCGGAGAGAGAGGGGAAGCGTGATGGGAAGCCGAAGGGAGTGAGGAAACATGATGGAGAGCCAAAGAGAAAGGTTGGAGAGAGTGAGAAAGCGCGATGGGGAGTAAGGATCCGAAGAAAGGAAGATAAATTCGAGACAGATAGAGAGTGATGCATGTGAATAGATTTAACTTTTTGTTATTTTCCACTTTTTATTTTTTTTAATAATAAAAAATATCTACAATGCTGAGAGAGGGAGAGAGGTGAAACAACAACACAAGTTGTCGTTTCTCTCAACCTTCTCTCGGCATTCTAAATATTTAGTACGACTCAATCATTAATACCTAATATGGGTTCTCTTGGAACTTTTATCAAACACGGCAAACAAATAAAATTCACCCTCGAAGGACCAAGACCTTGTTTACAAGGGAATACGAAGATAAATGAATCAAGCAAACAACACTTACTAAACTTGGCTCCGTAGTTCCTACGAATATGAGCGAGCGAGGGAGCGTACCATTTCATTTTCATTTTGGGCCTGGTCGAGCACGGGCTGATGCTTCATGTAACAAATTTGGCCCAATATAGCCAGCCCGGTCCAGGCTTCCCGTTCGACCTCTGCAAACCGGTCCTCATAAACCCGATCCACGATACGTGTGGTTTCAAGTGGCACGTGT

>Cs1g03510

ACGATTTAGTAGATTTTATTATTCTTGACGAATGAAAATATATACGTTTATATTTGCTGTATATAAGATAATGACATATACAGTACACTGTATGAAATATATAGAGAATCGAAATTAGTTAATATTTTGTTTCATGTGTCTGTGTGTTGCATCAATTGTCTTCTTTCTCTAGTTTCAGAACCCACATGGTTCATTTTTAGTGGCTGCCCGCTTGAGATCAGTTTATTAATTTTGAAGCTCTATTAATATGACTATTGTTATGTCTGGTGATCCCATTAGTCAGATTTTAATTGGTACATTTGCAATTTATATATATAACTTTTTATTAAACCCTAATGTGTAACGTGTGGCGCGGTTCTATGAACTCATCGCAGAACTCAGCGCATGGAAAATGAATTGTATAAAATTTGAAAATTTTTTATATCAACATAAGAGACAAAATCGATCCCAAAGCAGAGGCCAGATCAGTATTGATTCAAATATAATTTGTTCAAGTTGAAGATGACACATCAACAAGCAAAAGATACTTTGATTTTCGGGTAATAATTGAATTGATAAATAAAATTAAATGGAAGAGATCAAAGTTAAAAATAATAATATAGCATTCATGAATGCAAAAATTCAACAAGGTCGCCCTTGAGAAGCAACCATGTGAAATCGGTGGTGTTTTGATGGGATGGCCTAACCATCATCGGTTAGTTATTTCAACCAAAGAAGAAATTAGGAATATTGATTATATACATGTAATTTAACACAAGGCAACAGTGCATATTGACAGAAGAAGAAACCAAATCCATTTACAAATTAGTCAATACAAGTAACAAACGAATGAGTGAAGGATGGATGGCTCTCAAAGAAAATTAATTAAGCAATATTTTATTTTTCCACATGGGTAACAAATTAGCTGACCTGTCTAGTAATAGTACCCTCTTGGGTCATATATATGTATGGACGGAACCCCAACCTCCCCAAGCTTCTTTAATATAATCAATCGTATTGCT

>Cs1g25790

TTGTTGAAATGCATTACATTGTTGCACACCTTTTGTTGACTCCCGGATTCAATATGCATTATGTATTAAAAAGATTGGAATTTCCCAGTTCCCACTAAGAAGACCAACAACGTTTCCTTGCATGGACGTACCAAATATAACTTAGTATTGTTTTAAAGGCTTGGCCGCGTCTTCTGCGTAAAAGGTGAAAGAGAAAACACGAAAATAAAAAATAAAAAAATACATAATCAAGTCTTTAATTAAGATGCTTGTCAACCGTGAGAGTTCAATGAAAATCCTAATTATGTAATTAACAATGTGACCCCAATCGAAATGCTCCATCTAAGTTTTGAACTTATTATTATTAAATTAAGTGTCCCGTTGATTTTGAAATGTCTCAACTTACCCCGTGCGGTCTAAAAGCTCGATTCTTCGGAATTTGATTAGTCAGTTTGATTTTACAAAAGTTCATTACAAAGGTAAAAATATTTTAGCTTGTTGAACTTTAGTTGTTATTTCGAGTTGATTTTAGAAGAACACGATAATGGAAATGTCATTATCATATTAGCTTATGCTACCAGTCTGACAAGCAGCCCATGAGGATAATTCTATCAATAAGGAACTGTCGAGAATGGCAAATACATTTTTATTTAATTGTAGACAAGTCTCCGTCTCATTTCAGAATATAATATTGGCCTACGACAAGCTTTCCAAATTCCCAGGCTCCCCGACTGGGACATTGGGTCAAGCAGGCTTGGATTTTTTGTCAAACACTCGATTAAACTTCGATACATTCCATGGAGGGGCCACTTCTATTTTATTTTTATTTTTGAATAATTGCAGAAAGCAGATTCAACAGGCGGAATCAATGCGGACGCTGAGAAAAATGACTGTCTCCACCGTGAGTCCCGGGCCAATTGCGAATAAAACACCCCACTCCAAGCCATCACCGGTTGTGGGTTTGGCCTCCTGCATGCTGTTCTTCCTTATCTCATTTAAAGTCCATTCATCTTCATGATCTT

>Cs1g03040

CAAAAACGATTTACGTGTGCATATCAGAAAATATGCCCCCAACCAAAAGCTTTGCTCTCAAAGTAATTTGCAGCTGGAGTAGTGTTTTTTTTCCTCTCTCTTTTTTGGTCTGTGCACACTGCTTGCTGCCGATTAGCGTTACCTAACACAAGAAGCTTCGGCGCGAAAGGTTAAAATGCATGGATCTGACGGTGATTGCGGTTGAAACAGGAAACTAGTGGCTACGAAAGAACATATTATTTCCATCAACTCAACATGTGCTAGTTGATTGCGTATTTGTTTAATATAGTGGGGAAAAAAAGGGGTAAAATTACTTAAAATGTTTAGTGAACATCCAGAAAAAAAGGGGTAAAATTACTTAAAATGTTTAGTGAACGTCCAATTCTGTAGTTTGATAAGCACTAATTTGAAACTTTTATCTTATAAAAATTAATTTTTTATTCTTTGTACAATAAGTGTTGAAATGCCGATTTCTTAGAGAGCGAACTTCAATTTCACACTCACCTGAGTAGTTAAAATATGAGTAATGATATGGTTATAAATTCTTGTATAAATTTATTTTGTATAAATTGATGTGACATGATAAAATTAGTTGAATTAAATATCACTTGCCCCATATGATTTATTTATATTATTTTATATTTTCATTCAACCAATGAATTATTGTCACATCAATTTGTACAAGATAAGTTTATCCAAGAATTTATAACTGTATCATCACTCTTAAAATATTTAATAGGTACATTTAAAATTTAAATTAATGATAAAGATGCCCTAGAATTTTGTATTTATCAAGTTCATTGGTGTCTCAAGTGTATATAATTTTCCTTTAAATCTACTGCCCATATGCAGGAAATGCGACACGTGTCAAAAAGTACGTAGAATACATTAAACAAATCGAAGGATGTCAGTTGAGTTGACGTCAGCCTATGACAGGTCCATGCACTCAAAACAGAGTCCCTTTCGTTTTAAAAGCTCGCAGAATGAGGAGTGAGAATCTG

>Cs1g12820

AATTTTTAAATGATTTTGCGCTAACGGTTTAACAATTTTGCCTGTCTGACCAAACACGAGCAATGTAACAATTTGATACCATTTATGGTATAGAATTATAGAAAAATTTGTATACAATGTATCCATAAATCAAGAACTAACACAATAAATAGTTTGGTATGAGATGCTTCGTGCCATCCCTTCTCTTAATGGCTCAAAACAAAAAACATTATAAAAACATATCCCTTCTCTTAAGGTGCCCATATTGCATTGCAATGAAGGAGGAGGTTTGGCGCTTGACTCGGCAACAATGGCGTCTTTTATGGGCACCTTTCGGTCCCAACCCCAGCTACCATTTCTTATGCCGCACGTTTGCATTGAATTGTCTAATCCCACATGCGTGTGGGTATGAATTATTAGTAATTAGGTCTTCATTTTGCTAAAAAAAGTCAGCAAAAATATTATTTTTGGGCACTCCTCCACTTCTTTGAGTCAAGCCACGTGCCACAAGTCATTTTTTGCCGGCATTTTTATTGCTGCTTCTTTCAGTCTCTACACATTACAATAAGTCTTAGATATTTTCTATTGCATGCTTTTTCCTCCCTTGGATTCTTACACGCGCCAATTTTTTTTTTTTTTTAGTATTAAAAATATCAATATGTGTTATATACATCATATCCATCATATTAGTATGATAAATAATAGACAAAATTATTTTAAAAATATCAATAATAGTCGTTTTTCTGTCAATCGAACCTAATTTTATTGCTTCATACCGAATTACAATGAATAATAGAAAATTATTTTAATTTTGATCGTTAATTTCAAAACAAAGGTGATCTAAATGGGGTGCGAATTAAAGCAAATGCACTTTTGACCATACAGGCATATTCTTTTGAGATCCAATGGTTTCTTTATTCAAATAAATGCTCTTTTGTCAAGGAAACATCATTCACACCCCCATTTAAAAAATAAGAATTATTTGATGATCTCCCACACTATAAATATAGAAGCATGTTACC

>Cs1g08430

TCTCGTGCTTTATGGTGAAGCCTTATTATGGATTTGAGTGAACCCTTATTATGGTTGCCCAATTTTACCAACTAATAGCAAGGTGAACATAATGAGATTTTGAGTAAAGTCTTATTATGCTCACCCTATTTTATTACCCAAACATCTTTCTCTGTCCTTTGTCTTATTTCTATCATTAAATATTTACATTATACTATTTTACACTATGATGGATAATTATATCATCATTTTGTATACAATTATGCTAAGATAAGTTTATTAATGTCTTAATTTAATTTAATTTATTTTTCAATTGGACCTGCATAGAAACATAATCACTTAATAATAATACTCTTTATAAAATTATAATGTAGTAATTAATACAATACAAAAAACAAATCAAATATTAAGAAAGTGCACTTTAGAAGCTTGACCGAGCGGTTTTCAATCATTTTTATTTCGCAAGCTACTTGAATTCAAATTTCCAATGATAAAACAGGATAAAAAATGATTTGAACTTTGCTTACCCATAAATTGTGGCTCCAAAAAAGCTAACCTAACCATCGGTTTGCCCGATTTGGGTCCAATTTACAAACCAAGAAAAAAATAATCAATTTAATTAATTAAATAAAAAATCGACTTGAAAATTCGATAACCAAAAAAATAAATATTTTAAAAATTAAAAAAAATCGAACCCAATTAGAACCAAACCAATTTTGGTTTGGTTCATTTCATAATTTTAATTATGTTTATTCCGTTTAGCTTAGTTCAATAAAAATCGAACCGACCGGACCGAACCATATCACCTACTACCCCCTTTAACTTCTAATTAAAAAAAAAAAGGAAAAATTACATTGAGAAAGCCCTATGTGTAAAATGCTTCTTGGAATTTGTTCGGGTTCCAAAAAAGAAAAAAAAAAGTTAATAGCAATTCAATTAAAACAATCCATCTTTGAAATTCCTAATATTCTTGAACAGGCTTTTTTAAGGGCCTCAAACCATCTCTGAAAACAAAGGCTTGG

>Cs1g04910

ATGTTAAAAAAATTATTTTTTGAGATTTTGTTGAGTTTATTAATTAACTATTTGATAATATTCAGACCGTAAATATGATATCTTTTGGTACTAAAGACCCATTTGTGATAATTTTTAGAAGGTTTAAAAATGATTTTGAAAATTTAAAAGTTAATTTTACTGTTTAGTTAAAAAAAATCAAAACTACTTTTGAAAAAATCAACCTCTCTTACAGTTGATTTTGAAAAGTAGGAAATAAAATAGCTTTTAGATTCTAATTTTGAAAATCAATTTTATTCTTTAATACAATTTCATAAATATCCTTAAAATTATTACCTAAACCCAAAATTATCATTATTCAAATTAGAAATAAAATTAATATTTTAATAAATTTTTATTATAATTCATCAATATCAATTTATTGGTCGGATTATTATTAAATTTGTTTCACTATAATATAAAATTCATTATCAATTGTTTTAAGATAAAAATAAAAAAATAAAATCAATATATCATAAATTTTATTTTCAATAAAATTATTACAATACATAATTAAAAATCATGTTTTTATATATATAAATAAATTTTACCCTACACTATATACATTTTATTCATTTTTTTAATAGTTTAAAAATAAAATTTATCAAATGTTCATAATTATTTTTAAAACTCACAATGTTTTTGAAAACAATAATTATTTTAAAAGTTATAATATTTCCAAACTGACACTAAATTATTAGACATCTTAGACCGCCAAGTTATATATATTTTTTCTACAAATTATTGTAAAAAATTTACAAATAAACTTATAAAAATAATACTTGATAAAAAAGTTAAGACTTTTTCGGAAAATGGTTGGTTACAGATTTCCGTACGGTACTCAAAAACCGTTATATTTAGACGGATTCCAACGTTTGCAACACATTGCCTCAGAAATCACAAGCCAGCTTCTTCTATCATTATTACGCACATTTCATTGTTTAAGTGAGAGTGAAGGATAAAGGCCAGAGAGAGAGAAAGCA

>Cs1g07890

TCAGTACACAATGCTGTAGCAAAGCGTGGGTGGCTAGAGTTTTGTAACCATCCTCGAGACCCTATGCTGCCAGTTGTAAAAGAATTTTACGCCAATTTGGTAAGCCCTGGTCAGCACAATGCTTGGGTAAGAAACTCACTTGTTCCATTAGACTCTCGAGTTATAAATGCTTTCTATAATTTACCTGCTGAAATTAATTGTGAGTATGCCAAGTTGCTTGACAAATTGACCCCGCAAAGATGGAACACAATTTTCACGACACTTACAGTAAAGGGTACATCGTGGGCTAATGAGGAAGGGCATGTAATTAACAAGATAGATTTAAAACCCATTGCTAAGGTGTAGGTGAAATATTTGAAATCCAGGCTTATGCCAACCACCCACACCACTAATGTCTCATAAGAAAGGTTGGTATTGTTGTATGTTATTATTAGAGGGCTTCCCATAGATGTGGGAAGCATTATCGAAAAATAAAATTTGGGACTGTGCCATGAAGAACCACAAAGGTGCTGCCTTGCTATTTCCTTCACTCATCACCAGCATCTGTGTAGTATCAGGAGTTTGTCTTGATGCAAAAGATGAACATGTCAAGAATGATGGTGCCCTTACAGCACGCACTATTGAGAGAATTGCTGGTGAAACTGCTGGAGCCACATCTAAACCAGCTGCTGCAACATGAGCGAGACGAGCTATTGGGCTAGAAAATGAAGCTGTAGTAAACCCACAAGCAGAGGCTAACTCAGAAGTTGTTGAACAATCTGACCAACATGAGGAAGAGGGTGACAAGTCTGTTGGTGAACTAGATTTAGAGGAGGTCCTGACTAGGGTTTCTCACGTTCTTTCTCAGACTTTTCCTCTGCCTCTGTTGGTGAACTATCAGAGAATACAAGCACTGAGCACCAGCATCACTCAATGTACTGAGGCTTAGCAGAGGGAAAACGATCGGTTTTGGAGCTACTTACAGCACCTGGAAAGCCACTTACACTAATTTTCTCTATACA

>Cs1g19320

AGGAAAAAGAATTATAATTTTAACAAAAAGTATTTTAATAACTTGTAATAATATATTTTGATTATATATATAGGAATATAGATAATTTCGATTTCGATTCTAATTCTTAAAACTTAAATAACCGACCTATTATAATATTCTCCATTCTAATTTGATACCATCCCAATTTTATTTTAATTATAGTGCCTATTAGTACTGTGAAATATTGAGTTCCATCTTTCGAATGATCCTACATTGTAGAAAAGAGCATAGTAGACTAGGGACAAAGCTTTACCTAACGATTTTCGCACTCCGTGGTTCCAATTAAAAATGAATGAAAGGCTGACTAGGATTGGCACAGGCGTCGTGGATTCATGAATTTTAGCTCAAAGCGTGTCCTATCAGCGCACAACTTTAACAAATAAAATAGGAAAGCGGAATTAATGAAGACAAAATTGCTTTGCTTTGCTCCGAGTCATAGAAGCGATGGCAACTGGCAAGTTGGCAACATTGGCAACTTGTTTATTTTCGCTAGAAGCATCATCTCACAGTTAAATAAATATTTAAGAGAAAGGACATTTTACGCTTGAAGGGTTGTTCTCCCTCGCTGTTTTTACACATAATGACATCACCATTCAATAATAATAAAACGTATGATGAATGACATCAGTTAGTCTGCAAGTGATTGTATCATCACCAAATCAGTCCACGCAAAGCGCGAAAGATAAGAGTTTATTTATTTTAATCGAAGTAGTGGCCGACTAAAAAGGATAGATGCACCGTCCGCCGCTAGTAAAAAACGCACCGGAACAAAACCTTCGATCAAGACATTCGAATCTCTATCATTTATATGAAGAAGACAAATGCTCTTTCCCTTTCTGCCCAGGCGTGGAAAGTTGGATTTAGAAATTACAAGTCCAAAAGGAGGAAACTTTCTTTCAACTTCTCTGTCCCCACCACATTTTATATATCACATACATTCATGCACTGGGACTCCTCCCTTGACCCTTGGGATAATTTAC

>Cs1g15840

TGAAGCTGTGAGCTCTTGTGATTCCAAGATCTGAAAGTTTGGCCGACAGTGATAGTTCTATAGGTTTATATTTTTTCTTTTTCTGGTTATGATGATCCATCAATTTATCCTATTGCGCTTTGCTGTCTGTTGATAGATGGAGGTTGGCTGTGTTGTGTGGCAAGTTTAGGGGTTCTGGGATGATGTCTATTAATCATTGTGCTGACTTGTGTATACTTTTAGAAGGTTTCTGGCATGAGGGCTAGTGTGCGCTTAAAATCTACACAGCTTGTGTATACTTTTGTGATGTAATGTAATAAATTTATACTGGAACTCTCTCCCTCCCTCTGGATTGTTATGCCTTATTTTGTTCTCGTGTGACTTGGGGCTGGCTTTCGAATTATGATAGAATCGCTAACCATCAACTAAAAGTGGTAATTGCACAACTGACCAAAATCAAACCGTTTTCGAAGAAATAAGTTTTGTGAAGTTAGTTCACCGTCTCCCTCAAATAGTTTGAACTTCCCAAACCGGTTTATGTTTGATGGGGGTTAATTTCATTACAATTTTCTCATTGTCTTTAAAAGAGATAATGTAATGTCTTTTGTATGATTCGGTTCTGAACAGTAATTGTATTGTGACCATCCAAGCAGCATCTTTTAAAGATAAAGATTAAAAAAAAAAAAGTAAAGATAGAAAGAAATATTCCTAGGTGTCCAATATATTTTATTCCTTATCCTACCCAATTTTATCTAATCTTCACTGTCTAATTAAAATAGGATTGTTCAATTTTATGCGGATCAAACAGCTATTTCTTATTCCTTGACATTCAAGAAACTGTTTTGTACCCAATTTAAATCATCTAGATTGGATTGATTATTATTATTATTGTAGATAAATTAAGATTATTCCGTTAGAGATGGAGAACGGTAGAAAAGAACAGAAAGCACTTTAAGGACACGTGTAGTATAAAAGCGTCGTGCACTTCAGTAACACTGACACACAGCATTGAGTATAGAAGC

>Cs1g12040

GTTATACAATTCCTATGTGTGATAGAATCCTGTAAATTTCAACGAAGAGAATGAAGAAAACTGATGAGTAGAATACAAAGAATTTGCTTCTGGGCTTAGCAAAAGACAAGATGTTTCGATTTCTGTACTACAACGTTCATAAGCAAATGTACATAGTTGAATCAACCAAATCAGGGGGAAAATACAATACTTAATTTGAATTGCAAGCACGACTAACTACTTTGCAATTCAGCTCTGACTTGAATTTTTTAATAGGGAATCATTTGCATCAAAATGCTTAATTGATTTACGACAAACGATTAAGGCAATTGATAGTGACATATAATCAAACCTTCATAATCTTCATAGCATAATATTAAAACAAGCGTAGAATAATCATTTCATGTTTCTAGAATTTCTCTTTAAATAAGAAACAAGAAGTAATGATAACAATTTTAGGCAAAAGTTTCAAAATCATAGCTGTCAACATTTCTACATATTTCCTACATTTTTCTACAAACATAAATAATTGAATAAAAAAATGATCGGAAAACTAACCACTGGGGCAACTTCAATGCCACCTTGGTTTCTCTGGTATCCTAGAACTGTACATGGCTCTGAAGAATGAAACGAAGGTAACAGTACAAAATCCGCCATCTGGACAACTAATAAATCGTGCAAATCAGCGACAAAGCTGGCACGGTGGCTCCCATCATCAAGAATCGTTTAGTAATGGCGTTTCAATTATACAGAGATAGCTCATCATACCTTTGCTTTGCTCCTGCTCAAGTGAAAGGGGTGGTATTTCGTACTGTTGTCAATGGAAATAACATTTGTGCCACAAAATTGACTAAATAATATAAACTGAGGTCTTGTCCTCTGGCAAGTTTTGTTTTGTCTTGTCCTACTGACAAGTTTGCTTGATATATTTTAAGGATTGGTCGTCAACTTCTTCTTTTACTTGTTTTGGGGTGGATTCATTCTGTTAATCACAAGTGGTATGGCATCTAATAGCAATTCAA

>Cs1g24560

TGATTTATCATGGAGTTTTTACCTTTGTAATTGTACGTACAATGACACAATCTCCCTAGTGGCGGTTTCCTTTCCTCTTTGGCCTTTGCTTATTTGCTATGCATGCTCGCAGTGCGCTCTAGAGTCCCATAGCATTAACTTTGTCAGTCTTATCTGATCATTAAAGTTTCTCAGGGTTGCTTTCTTTCACATGTAAATTAATCTCGAGCTGTTGTGATGAATTCTTGGTATTACTTTTAGTCGAAATTACTCTTCACTGAAGAATAGAAACATGGCGTGGTTAGACTTCTAATAGAAGCCTTCATAGAGCAGCGGCTTAATTTAACTATAAGCCAGAACTGAAGAGTGATTAATGCATTACATAGTATGGTATCAACAATTGACTAGGCATGTTTATAATATATATAGTGAAATCATTACTCATCATGAAAAATAAGTGCCAACTGGATAAAATATTCTCGTTCATTTTTATCTTTTGGTGGGCAATTGTTGCGAAAATAATTCAATAGACTGATCAATGCAGTTGTTAATAAACGCATAAACTCACCATGTGTTTACAGTAGTCGAATTTGGAACAAGCCTCGCTAGATGTTAAAGCTATGCACACGCCTCCCTTTTGATAAAGATATGAATTAAGATGGGGCTTACTTCTGCAGCTAGACGGAGCTTTGTTTCAGTTCTGACATAAGGGTTATATAGGCTTTTAACTTTTTATTTTGTTTTTTCGTTTACTAATAGAGGTTCTAATTAAGAGTCCAGCAACGTAGTTTTGGCCTCTAACTAATAGGTGAAATATATAACCAGATAACATCCGGTAGTACGTAGTTACATATAGACATGCAGACCAAGTCCATGACACTTCCATCTCCCATCTTATCTAAGAGCATCCATTAATTGTAAAATACTAGAAATTAGAACACATGGGTATAAAATAAAAATGGCATTAGCGGGAGGGATAGTGCGTTGATTGGGGAAACTGGGGGTTCCCAAGGTAGTGTTCA

>Cs1g02990

CAAAAAAGAAAAAAATCAATTGATTAGAAAAAGATGCCACTGCACAAATACACTGTACACAAACATATTCCTGTGTCGTTTGTTATTCTTGATATGGAGTTTATTTGCTTTCCTCCCGTTAATGGGGTAATTCAAATTTGCCGAAAGTGGACCTAAGTGTTTGCTGGTCCATAATTGAGGCTTCCAAATTCAACCTTTCGGGTGCGTTTGGTGTGTGGTATTACATAGGATTAATTGTGGGCCCAACATAAATTTAAACCAATAACATGTTTGGCAGCAGCTGAATCTAAAATTTAAGACCTCTATATTTAGTTTTTTTAAGTACTAATAAATATTATTTTATAAATATATAATATAAATTAATCATAAAAAATTAATACAGTACAGTGCAACACCAAACATAATATAACTAAAAATTAATACAGTACAGTGCAGCACCAAACATTGTACAACATTATTATAATACAGTATTAGTGCAATACAACATAATACAATACACCAGCATAAAAATAATGCAATACAATATAATACAATACAGTGTACCAAACACACCCTTAATACCATTGCACGACCTTGGCAGCCCATGTTAATTTTCTCACTTTGATTAGTGTGGTTCGTAGAAGGAAGTCGCAAAGTTCAATCATTGCCCAGTTAGTTGAACGTCGCAGTCGCATTTTCAGCTGTGTGAGAGAGCGTACCTTACGTTAAACGTAAGGTAGGCAAATCCCTTATTATGTAGCTAGATTAAAGGTTGATTAAAGTAACTTTGTCTCCATTTGATTTTAAGTATTAATTAAAGATACAAAATTGAAAACCCGGTAAAAGAAAACACAGTGAAGTCAAATTTGATTCTTTAACGGGTCCATTATAAAAGAAAACCATAATTATGAACCCGAAATTAAGTATTTTGTCATTAGAATGTCAACGTTGCCGCACTAGCCACTAGACAAATTGGTATAAAATGCTTCGGAAGACAGGCAAAATGAAAAGGGCAAGCAAGT

>Cs1g04240

AATATGAAACTCTTGGACTTTTGAAAAATTAATCAGAAGGGAAAATGAAGGACAGATCGATCATTTCTGGTAAAAAAGTCTTGTGCGTGTCTTTAGATGATCCCAACAGTGTATACTCATTTTCCTTTATCATTAACCATCAATTATTACTGTATATTTGTTATCATAAAATTCGGATAAAATAATCGTTGATTCATTTCAATCAAACGGAAGCTTCCTTTGTATGTTTCTGTGGAAGTCAAATGATCATGTTGTTTTCACATGCGTATGCATTGAACCATTGTAATTAACAAAGTGTATCAATTTTCAATGAGTCAAGGTGGGACTGAAAAACTCTATTAAACTTGTGGGAGTGGGACAGATAATCTATTTTTGAAATTCATAACACTCTCAGTAATTTAGTTGAACAAATATTTAACTTCTTTTTATCTTATTTGTTATCTCATCAGCACTGCAGAAGTGGATTATTTCATAAGCAGCAACAATCCAAAAGTATCCCTAAATAAATAATTTTATTCAATATTAGGATTTGTTCGCCAAAGACTGACTTTAGATTTTTATAGTAAATAAAATTACTATAAAATTTAATTATATGTACGTTAAATTTTTATGAAATTTTAAAATCAATTTCAGGGGTTTGTATAATAAAAGCGTATAATAGTAAAAAAAAATAGTTGTCTTTAAAATAGTATTTCTTATTTGTATAATTGAGTAAATTTTATAGAGAGTTTAGATTTTTTTTTCGCGGTGCATCAACTTTCATTTTATGTGTCAAGTACAATTTGTATATTTTATTTGTATATTTGAGTAAATCTTATAGGGAGTTTAGATGTCTTTTTCGCGGTGCATCAACTTTCATTTTTACAATTTATATTTTTTTATTTAATTAACTCACACACATTAGTGAGGCTCGAACCTAAAACATGAGTCTTTGAGTGACCACTTTTTACCATTGAAACTTGACGTCGCCGTCTAATGTCAAATACAATACGAGATCAATA

>Cs1g11050

TATATATATATAATTTCAAACTTTCATCGCTTATCTTAAATTACGGATTACAATTTCAATTTTTCAAAAGGCCACACGTTCACAAAAGACACTCGTTAAAAAGGAAAACAATGAGGACTGCTCTTATTGTTTAAAAAAATTAAGCGAAATCTCTCTTATATTTGAAATTAAGTTTAAATGCTTCTATAGTTATATTAACTGTGTTAATTTTATATGTTAATTTTTTATTTTACTTCTAGTCTTGATGTAGATTATAAAATCTAAAAATTTTAAAAAATAGTAATTAAGTCAAATGATTTATATACCCTCAATGATTAAATTAATTTAATAATAAATTGTAAATTAAGAAAAAAAAATTAACTAACATAATATTTATGTAGCTACTAGCTTGCATTCATTATGATTCTTGACCCTTCTTTTTTCCTCGTTTCTTTTTTTTCCCGACAAATCACGAAGAGTAGTGCCATTCAGCAAAAGGTCATATTGTTTTGGCTTCTTCAGCCTTGATGCTTTCTTCCCCTTGACAAACATTTGAAGAAGCTACAGCAAGCCGGTTTATTCAGGTAATTAAGCTATTTTTTCTTTTAGATTATATTGCACTATTGATTTATTTTATCTGATATGCTGTTAGTTCTTAATTTGGTCATGTCAAATTGAGAAACATATTTTCATTCATTAATTGATCCATAACTCAAAATATTATTAATTATTACCAAATTCTTATTGGATTGCCTGGGATCCGTAACTCAAAATATGTGTTCAGATCATGTCTTTCACGGATTTGGCTCAGCATATTTTGTAAATCTTATGTAAGCATATTTGTTCCACATAAGTAAAACTTTTTTTAAGTTAATAAATAAGTCAGATGAAATTATTTATCTTTATTATTAGTAGTTATAATATTTAATGTAAAAAATTGATGCAATTTTGAACAAAAATTGCATTTGGCAGAAACCTTATGCTAATGCTATACTGATTAATTCTCTTCTAGGACCAGCCAA

>Cs1g26680

AAAACAGAAGATCAATGAAGGTGAGGGAGGGAATCGGAAATTTTTTACTAAAAGCGCCACTGCGCCAGGCCTACATATTTACCCATCACGTCGTTCAATAAAGATCCAGTAGTCAAAAGCGGGTGTACTGAGAAATATACAACCTACGTTCCATGGGTCTCTAAACAGGCCCAGATCTGGCTCAAAGAACCCATAAGATTCTCGCCAGATTACGATTTTTTTTTTAATTTTTTAATTTTTTAATTTTTTTTCGAAAATCGATTTTATTTTATTTTCTAAGATTCACCCGCCCCCACCATGCTCAACAGAACAACAGCATGACTGAGCTCTCCTTGATTTGAATCCTAATCTAACCTGAAGTCAGAATACAACAATATTTTACCAGTGTCGAATTGTGAGATGAGGAAATATTTTCTTTCTTTCGATAAATTAAGAAAAGATTTAGAACACATTTTTCAAAATAAAAATTAATTTGAACTACTCGCCCCTCTACTTTTTCAAAAAAAAAAAAAAAATTAACCAATTTATAGTTAGTACAAAAACATTTTATTTTGTATGCATTGAGCCATAAAATATGTAAAATTTATTTCCTTCGTAACTGAGGCATCAAAACATTAGTTAAATGCCAGCGACCCTTCTCGGGACACGTCTAAACTTTGGTTAATTACTTTTGATTTTTCTCCAGCACTGGGTTGTACCCTGTTGAGCTTTATGGGTAAATTTACCTGTACATTTAAAATATTAAAGAAAGGAAATTAGCTGGGGAAAATGCAAATTGCAGACACAAGACAAGCTGATGCTGATCCTAAAATTGGAAGATTAGAAGAAGCCAGGAATCTTCGAATCGGAACAGTCTTCATAAGAATGTTTTTGTCTTTTGACTTGACTTAAAGTTAAACCCCTCCAATCGATCAGGCCAAGCAAACCAATATCTCCTCTTCTGTCTTCTTCTCTTCTCTACATATAACAACGGTTTCAGTTTTGAATCCCCGCCATTTGCA

>Cs1g02090

TCTTATTATCAATTTATCACTAATTGGTTGTCTTGTTCATTGTGTTTATGATTTACATATTTTTTGTAGATGGAATTCCAGAGGAAGCAATACTTTCTACTGAGCAGTTGATTGATGAAAGTTGGATGAGTGAGCAACAATCAAATGAAGTGGATGATTGAGAGTTTAGTCTTTAGCACTAGTGGTTTGAAATTTGAAATTTAAATTGTAGAATTATGTTATGGATTTGTATTTGTGGATGTTTATTACTTTAAAATTTTGAATTTTAGATTCTATATTGTGTTGTATTTGTTTTAGAACCTGGATAAGTTATATGTTTTGTATTTGAAATTTTGTTGAGCTAAACTAAAGTAATCTGGATTCTGGAAGAAATTTGGTTCTGGTTGCTAAACTAAAGCAAAATGAATTTTGGAAGCAATTTGGTTTTGGTTTGTGTAATTGTGTTTTTTTTTTTCCTTTTTCCAGCAACTTAAGTAAAGTAATCTGGGTTCAAAACCCAGATATTTCCAAGTTGAACCCGGATGGAAACAAACCAGGTCGGGTCCGGTAATGAGAATTTAGTCTTCGGGTCCGGATTCGGAACGGCCAAACCCGGACCCGGACCCGGAAATGGTTATACGTGTGTATGCCAACCTCGTGCAATCGACTTGCCTGTGTTGTCAAAATAGAAGAAGAAGAAATATAGAAACTTCATATAATGAGCCAAGTTTAAAACAAATACACTCAAATAACCCAAAAAAAAGGCCAAAATTCAAAGTCTATGGACTAGCGATATTGCTTGCTTCCACTTGCTCTCATAATTGAGCTACGTAATTATGTTATCCACTTCCTGCATGGGCCTCTGAAATAGAACCGCTTATTTTGACTAACAACAGTCCACAAGGCCGTCCAGGCATATATGTATGTATAAACAATACTGCCTATATGTATATATACACGCTCCCGTTCTTTTTTTGATTTATCTGTTATGGTGCCCTCGTTTGCTCATGAGCTTATAAAAA

>Cs1g13900

GCTCAAAGCGGTCCGATTACGCAACTTTTTGCTTCCTGGAAGTTTGAAAAATCATCTGCGCACTATTGAAAGCAAATTAAGCATGCTATCAACGACAAAAGGAAGAAGGTTGGTCATAGTTTAAATAAATTTTTTAAGTTCTTATATCTTTGAGATTTCGCACTCGAATGGACAACCAAACAAAGGTGCCACAAAAAATTGAGTGTTTATAAAATTATTTCAGTATTATCTGGCAAGTATGAAAGAAGAAATTCAACATTTTCCACATTATATTCGCATTATTTCAATATTCATGATTTTTTTTGAAATATTCATGATAATTAAATATTAAATTTCCCATATCTAATTCGAATATATGCCAAAAAAGAAAAAAAGAAAAGAAAGAATCAGAGTCATAAAGAAAGTCAAATATTATTACCCCAACCCCACCCACAAGTCACGCCAAAACAATGATGAGAAAAAAATACGATTCTACAGATTACTTGAAAAGCTCTTTTGTTTTTGTTTTTAATTGAAATATATGAAAATAGCCCAATGGCTCACAAACGATGGGAGTGTCACAGCCCGCCTTTGTTCACTTATGGGGTCCATTAATCGCCGTGCATCCCATGGTGCTTCGGTGTGCAACTGCACATGGACCACCATGCAACGTCACAATTCTTGTTCAGCCACGAAAACAGATGTTCAAATTGACTGCATTTGACTTTTGTTATTTCTCAAATAAGTGACATTAGCAGAGTCTCTTTCTACCTTTGTTGAATAATGTGACAAAAAATAAATATAATGGAATAACTAATAATTGGAGAAAAAACTTCACAAATAAAAAGAGGAAGACTAAAAAGGCCAATGAGAGGTTGAAGTCAATTGAAATTGCTTATACTCTCATGGTGCTGAGTCAAATGTATCAACGGAGGGTGAAGAAAATATGGGAAAAGATTTAACACGCCTTACATTCGCAAAATAAAAGAAAGAGGAACTAATAAAGTTTGAGTGATTATATA

>Cs1g19110

AATATATGCAGGTGATCATGGGTTTAATAGGTAAAATCTCTAGAACCTCCCACTATTTCATAGTACCCCGAAATGCACCTATCATCCCAAAAATTTTGTTAACCATTCTTGTTCTTCTTATTATTATTTGCTTATTTTGCAGTTTCAACCCTCTAATTAGTGTGATGTCAGAGCTTTCTGGCCCCACTTTAAATGCCTAGGAATTTTTGTTTCAGAAAAAAATAAAAACAAAATAAAAAAATTTAAGACATTATATATAAAAATATTTCAAAAAACTCAATAGAAAAAGTCCATCTTGTAACAAATATATGCGCTAAGATCAATATCACAAATCATTGATTGGTTGTGTGGTGATCTATGAACACCTCCATACCTTATCAAGGATTTGAGTTTTATGTGGAAGTATATTTTATTTTATTCATAATTTTTTGTGATTAGTATATGTCAAGTCAAAATGATAAGAAATGTCTTATGATGAAAAGTCTTAATTGGTCGTCTTCCTACAAATACCTAAAGAATATGTCATTAATGTGGTTGAAATGTATGTGGCACATGAAACTTTCAAATTAAGTGGTCTAATCAAATTAAATCATGGAATTGGTGATAAATCAAGCTATCAGCATGAATTAAGAGACTAAAGAAAGGAGATTTTATCAACGGACGGAATAAATTAAGTAGATAAAGAGGTGACTCATTGACACTATATATCTAATTCTGTTTTAATTAATTACATCAGTTTATTAGTTTCCAATAAAAGAAAAGTCGTGAATAGAGACATATTAATTGGATGAACAAAATTATAGAATTTTTTTCTTTTTTTTTTCTTGTTATATAATTAAACATAGAGTTAGATATCCAAACATGCTACGTAATTGTATGATTCCTAACTTTACCTAGCATACAAACAAGATCAGTAGGAGAATAAATACTTGGCCCAAATGGTGAAAATTTTCATCAGAAAGTTAAATCAGAAAAAAAATTAAAATATTGAAGTTCGACAA

>Cs1g25010

GTTGTTTAAAATTAACCCAATTCAGGTATCCTTGTTCGACCACTGCTAGATCATTGGACTCGTTTGAAGCAACCCAAGAACTCGTTCTTTCTAACAATAAAGCCAACAAGAATAGCTTCAGCAACATTGTGAAAGAATGAGAGGAAAACGCGGGGCTGAAGATTTGAATTAGTAGTAGAAGATAAAGAGTGTAATATTAACAATGAGGACCAAAAGGCACAGGAATCTATCAGGCTATGGCTGTATCTACGTTGGCCTGTACAATCACATCCTTTAAACCCAGCATTGATTTTTCAGTAGAAGCACTGTTTCTTCTTGTCTCTTTTGGTGAACCGAGAATTATATCCATTTGTTATGCAATAAAATAGTGTTTTGGTGCAGCTTTTTCACAACTCTATAGACTCTGGTCATCAGATAATTATAAGGTGTTTCCGTGAATTTACTTAGCTTTTTCCAGCTTCCATTGACTATCAGTGGCAACTTGTCTTCGACAAAACAACACAAACTAATAAACAAAATTGAACATAAATGCATGATGGGTCCTCGTAGATGTAATAAAGATTAACTTGCTCTTCCTACCATTTTGCTTTTTTATCTTTTTGAGTTTTTCATATGTTAAAAGAAAAATTGCAATAGGTAGGCTACATTAGAGTATGGTCCCTTATAAATTACAATTTCAGTTTGTAGTGTGAGGAAGATTGCTTACCGATGTGACAGGAGAAACGACAAAGTGAAATAGTGGTTCATGATGGAGATACTACCCAAAAACGATGACGCATTGATCTCGGCTTCGTCATCCATTTTTATTTTTGATCACTTGGTTTAAAAATACATGTTAAACTTGGTCACGCGTTAAGCAGAGAATCCGTTGTAGTCTAGGTGGTTAGGATACTCGGCTCTCACCCGAGAGACCCGGGTTCAAGTCCCGGCAACGGAATTTTTTTATAAGGTAGCTCTTTACTTTTATTAGTATTTATTTATTTATTTTTTAACTTTCTCTG

>Cs1g21880

AAAAGTGATAAGTAAACATATTAGTGATTTTATTTCATGTTGATTAGAAGCCACAACACCTTAACATTACAGTAGCCAATTTCTAATAATTACTTATTTTGGTTGGTGTTTGGACTGGAATTTTTTTTAAAAAATTACTTTCGAGTACCATGCCCTGCGTAAAATAACATCTAGGGTGGCCATAATTGCTTGAAATGCAAGCCACTGTTCGCTGTTGTAGGTAGCCAAATCCTCCATAGACCGGGTGGTGTGATTAATGGGCCATAAAAGACTTTTGCTATGCATTATTGGATTGTGGACCCGATGGAACAATGAGATGTGCAATCACAATGTGGATTATTGATCAACTCAGAAATTTCGCGTAGTCAATTTTGTCGCAAGGGACTCATAAATTGCGCGATGTGAAGGGACATCCAAGGGAGGCCTGTGATGTTTACGAAAGCTATACATACATATATACATATGAAAGGGTTGATGGATAGTTAACATCTAAATTGATTTTTTAACCTTTATTTTTCTTAGGATTCGAATTCACTTGGCCAGTTTTTATTCTTTTCATTAACTTTGTATAAACATCATTTATTGAAGAAGAAATCCAAATATTGCACTATTTCTATATCTTTTATAATAATAGTAAAAGGGTAACCCAATTATCTATTGCCCTAATAGAAAGGCTTGTAATAATAATTGTTTCGCATAAGAATAGAGACCCACATTCCATTCAGATAGCATCTGAAAGTTACCTGCTTAGATGGCTTTTTCTTTCTTATTTTGCTAATCAATAAATATTCGACAACTTTCATTTTAATGCAGTGAAACAGATACGTACAAGGAGGATGGACAAACTGATGAGACTCTTCAAAATAAATCAAGTAAGCACGGGTTAGAGATATTGGATATTCAAGCCTCTTAAGAAGTTCGTTATGATGAAGATCTTTAATTTTGTGGGTGGGAATATGCGCCACGTGGAAACTCTTACCTTTGGCCGCCAATAAACATGC

>Cs1g09370

CATCTGTAATATATTCATCACAAGAAAATGGCTGTGGTTCCCATCAATCTCAGTTAATTTCTTTTCTTTCTTCTTTTTTCTTATTTGTATGTAGAAAATATATTGCATCTAATTCTGTATCACATATATTATTTGGTCAAGTATATGCAACACTTTTTAATCTTTTTTAATTTCACTTTTGACTTACCTATCGCTTTGACCATATTTTAATCTTTAATCAAAGCAACTACTTGACCTTAAATTATTTAAGTTTTGCTGCAAATACTAAGATAAAACATCGTCTGCACTCAAAGTAGAATATTTAGAGGCAAGTTTGTCTAGATTCAAAATTCATATAATTTTTCTATAAAACAAGAAAGGTTAGGCCAAAGAGGATACAGCAAGCCAGCAATTACCTTTAATTAGTTACACTCTGTGCTATTGGGAATACTACATACCTGAGACAGGATTGAATGATATGGGAGGAGTTCTATTCAAGTTTCTATATTATCCATATTCTGAATTATTAGTCAACCTTATTGGCTCGAAAGTTAGATTTAGGAATGTTAAAGCATGTTCTGATTGTCGTCTGAAATAAAGTCGGTAGGATAGTGTTGAAGAGGATTCAAATGATTCCACAAATCTCATGGTTCTGTAGATTGTTCATCTTAAATTGAATTGGTTTGTACTGCTCTGTAGATTCTTATTCTTTTCATTTTTTAACTTTGGTCGGTCAATTTGGAAATCAGAATTTCTTCGGATTTTTTTTTTTGGCTTATTTTTAGAGTATGCCTGTGGGGTGGCGTAGGAACAGTGAACTCTCACTGTTGGCTTCATAGCAATCAAGAGTTTCTTATTAAATTTTTTGATTAAATCTTAATCAAGTGCGAGTCACTTTATTGATTTAATTTTTGTTCTCAGATGAATCTCCTGTGCCACAAAGGACAAAGTGCAACAAGCCAATTTTTTGGCTCACTAAAGAATCATTAAATAGAGTTGGATCTTATGATGGAGAAGGTACA

>Cs1g17370

TCGAACCGAAAGGCACAAGTTCTCCCAACAAAGGCTGTGCACCATATTTATCATAAAGCTAATTTCGCGGCGGACTTCATGGCTAGTCATGCCCTCACTCTTCCTCTTGGCCTTCATCATTTTGCTACCTCTCCTCTGGGCATCGAGACTTAGTTACATAATGATTTGTTTAGGGACTGTGTTTTCTTATGTTGTTAAACCTTAGTTTTCGCTGAGGTTCTTTATTTAACAAAGAAAAAAACTAAATACCAGAGTTAATTTTTAATTTTTTAAAATCATTTTTAAGCCTACTAAAAATAATTTTAAATGAGTCCTAAGTCAAAGATACGAGCTCAGCGGAAGGCCAATGTCATAATAAACTTAATTGCAATATGACGTCTAATTATACTCACCATTTTATTTTTTAAATATTAAATTATAAGTATCCTATCTGTATGAACAAATACCTGCACTACAACAAATTTCATTTCACAAAAAATCTAAGTAATTCGTGTATAAAGCACCTCACATGGCTAGCTAGCATCACAAAGGTAGCTTAGTGGTCAAGTACAATAATCTCTTCTAATAATTAATTAAAAATTTAAATTTATTAATTGAACAATTAATAATATATTTTAGTGCCACGTTTCATGCCTTAAAACTGATGTCACTGTATACGTCATACACATTATCCTGAAACTAATAAATTGTAAATATAGGCCACCTTTCTTGAAAAATTGTCTCTTATCTCTTTGTTATATATTTTCTGATCAACAAAAAAAATAAAATTGAAGTTTTTTTTTTTTTTTTGAGTTATTGAAAAAAAAAAATACTTGCAATTGATCTTGAAAACAACTACTTTCTAATTCGCCTTAAATCACCAGAAGATGCTGTTTATGCATTAACCGAGGGGCCATGGGTTATCTTTGGGCATTATTTAACAGTGCAGCTGTGGACACCTCAGTTTGACAGCACCACTACTGATCTTGACTCTGCCATTGTTTGGATTAGGTTACCCGGCA

>Cs1g07190

ATATTCAAAAAAATGAGGCATTCCCAAAATATCAGACCTTAGGCGAGTACCTTGTTTGCTTATACTTAAAGTCGCCACCAGAGATTTCTATCTCTCTCATTTATCTATAAAGGATGCACATTAGTTTTCTCAGTTCCCTCCAATATTTTATATAGAAGTGATGAGAATTAAAGTTTGGGGGAAACTATAAAATACTGGTGGGAAATAATTATATTTTCCTTATAAATTAAATAATTACATTAGACATTAATAAATAAAAACTAATTTGTAATCAAATTGAAAATTTAATGCTGTATGGTATTATATGTTTATAATTGTAACATTAGTAGTTTATATTATACTATATATTTATATTATATTATTATTTTATTTATAATTATAACATTATAATATTATATTATTCGATAAAAGTATATTATTAAATTATTCTAATGTTATAAAAATTAATATATAGAAAATTTAGTTATTTAATAATTAATTTAAAAAATTAATAATATAACACTAAAATATTAATAAATTTAAATAATATTAAGTTAATTTTAATATTAATATACAATTATTTATAATGCAGTCTATAGTAAACCAAACATTGTGTTGCATTACAATTTAATACAATATATTATAACACAATACAATTAATATAATATAATACAATGCAGTGCATCAAACTCAATCTTAAAAAAATCTAAAATAAGAATTATCAAAAATATTTTAGTTATCAAATAATTTATTTAAATATCACCCAAATATTGAGGAGTTAAATTTCAAAACTCGTCCAGATAAAATCTACCGTGCAGCCAACAAGAAGAACGATTTCTTTAAAATAGCCGAATTTCTAAAAATCCAAAATTGAAAAACCCTAAACCATTGGGAGTCGGGAGCCCTTTAATTAGTTTTTAATTTTTGTGAAACTAAACAAGTAAACATCACAAAAACGACAACCAAAATGAAGATTGACCGTCGTTTCATCTCCCTCTCAGTCTCAAATTAATTCAACTCCG

>Cs1g10920

NNNNNNNNNNNNNNNNNNNNNNNNNNNNNNNNNNNNNNNNNNNNAAGATTATGTAAATGTGTGTTTGGTAAGTAAGAAAATCAACGAGAAAATGAAGAAGAATCAATCTGGAATTTATTAGAATTAAAACTGTATTATACAAAAATGTTTTAAGCTCGGTATTTATACATTGTAAAGAAGCTGACTAGAGTTTGTTATAACGCCTATACACGCTTAATGAGTTTGTTAACAATAATGCTGACTCAGCTAACTAACTGTTACACGTCAGCTAGAAGATAATCAAGTGTGCTCATCCTCACAGCTCTGATCATGCTCTGTAACAGTTTCGGTCCAGTAATGTTTCTGCAATTTGTACTATTTTTGCAGGACGAGTAGCCATGGCCAAAAGTTGGTTCCAGAACGGATCTCTCAATTGATTTGAGCACCAAAGTAGATGGAAACAAATTAAATAATTAAATAATGCATTGCGTCAAAGAGACTTATTAAAGTTTATTTGTATTTAAATATATTGTTTTTCCAAGTTCTTCTCATTTGTAATTACTACACATCCTTTGATAAAAAAAAAAAAAAAAAAACTCGTGAATTTGTAAATGTTACCTTTAAATAATTATTTATTTGTCTTCATATTGGATTTACTTTTGCCTTAATAGTTGCCGCCAAATATTCACACACAAATAACAAAGGTTTCACCGCAATATGTATCCTGTGAGATTTTTTTTCCTGTAAATATTGGTTAGAGTAATATAATTATTCTTTTTTATCATATATATATTATTATTCTTTGTTAAAAATGACAATTTTATTTAATATCATTTCTTCTATTGATAACTAGTGCTGACTAATGTTTGTTTGTTTGATTATCCAAAACTCATACAACAAGCTCAAGAATCAAAACTTTCCAATCCTTAACGACATAGATAAAACACAATAACTCTAAGTTTATACTTTAACTTGAACATTCATGGCCGACCAAAGGTGGAAGAAAAAGTTAACTCTTATAA

>Cs1g09910

GCCTTCAAAAAATATAGGGTCTTAGGCACCTTCATATGTTCTTTTTAATCTATGAATTATTTACACAAATATTTATCATAAATTATAAATAAGTTCGAGTGATAAAATGTCTTAGGTCTGCCTCTGTCCTCATGGATTGGCTTAAGACTTTAAACAATGAAATTAAACAAACCACGTGGAGGTAGCCAAATTTTAGGCAGAGTTGGCGCATTATATTCTGGAAGCAACCTTATATAGTTGTTTATATATTTGTATGATTGTATCCATGGCCCAGTCATATGCCTTTAGATAGCATATCAACTTGAATCAAGTTAATTCAATAAACTTTCTTAATAATATATTAATAGAAGGTGACGGTATATTATTTTAGACTTTAATAAAACTCAGGTGATGAGTTATCTTCTTTAATTCTCACATAATTAACAAGTATTTGGGTAAATCTGCAGTATCGCACGAATTATCTGTAAAAGAATAATAGAAAACATTTGATTAATGATCAGTCTCATTCTCTTTTGAAAAGTCTTAAATCTCAGTTCTTACTTAAATTAAAAAATCCACTCATGATATTAGATTATCTTCTTGTTTATGCATCATACTTTACAGATTTTTCATCATGTATGAAGGGTTCATAATCAAATCTACTTTTTTTGTATTGATAATAAATTTTTTTATCCCACTTGTCAATCTTTTGAGGTTTTTGATGATATAATTATTCTAGTTTTCATTTTAAAAAAAAAATTAGATAGTTCACCCCAAAATGAAATTCTTAAATTTCCTAATTAATATTATTTCAAAATTAATTTTCTCATAAAAAAACAGAAAAAAAAAGAAAAACTCTACTCCCTCAATTAATTTTCACATAAATTTTCCCTCGCACAAGACCCTCTATTTTAGTGGAAAGCTTGCCATGCAAAATAAAAAAATTATATACATATTGGTTAATGCTTTTAAATCACGTTGAGTTAAGTGGTGAACAAAAGAAAGATGGTTTTGAAGTTTCC

>Cs1g15460

TTTTCGATTCGGATAAGGATTCTGCGACAACAAAAGACAAGTGAGTACACTTTGTGTAATGAAAAAGGTGACGTGTTAATTCCCGCTCACAAGTTTATTTAATTAATTTAATATTTGTAGTACCAACCATTGGGCCTGGCCATAGGAAATCGAGGCAAAGCCCATTTCGAAGACCGGTGAAGCATCAACGTTTGTAAATATATGTTATATGTGAATTAATCTTTCAACGTATAAATGAAATTGGATAATTATTGAAACTCTATAATAATTTTGATTCTTTCTGGGAATAATTTTAAGAAGTCTTAAGAATGGAAAATTCCTTGTTACGAAAACAATAAAGGGGTTCGTTACTATTTTTGTTTGAATTTCGCAAAATCATAATATATTTTGTAAATATTTTGGTAATAAACGAAAATAATAAGGTGGTTCATTACTATTTTGTTTGAAGTTTGCAAAATTATAATATATTATATACATGTGGCTAAGTTACAAAATATTATTTCTTACATCAACCTAAAAATTATGGTTAATAACCTAAAAGTATTTGACAACTAAACTAAGAGTTTGTTTAGGCAACTGCACTGCCGCATTTGCTTTGTTTAGAACAATTCTTATATTAATGTATGTTAAATTATTATGTAACTTAGTAAAACCTACTTTATACATATTTTAGTATTAATAATTTTACCGATCTGAACATTTGTGTTCAACTTAAATCCTAATATTATAAATAATTTATTTATTATATAATGAACATAAATTACTATTCTACCATTAAATAAATAAACACCACATCATTTGATAATTTTCCTAACCGTATCCTTTTTACTAAATCACTTCATTTTTACACATACCATGCTTAAATTACGTAATATATATATACACACACACACCCTGAGTGACTATAAACTATGAAGCTTTTTTAAATTTAAATGATTATTAAAATAATAAAGCAAAATGATGACTAATATATTTTTCTATAAAAGAAAAGAAAAATGCTA

>Cs1g11240

GACTGAAAATCCACCTTTAGAGAAAAAAAAAATGATGCTCATTAAAATGAAGGAGAATTTTTATTGAAGAAAAAACTCTCAAACAAATCGAAGGAGGTACAAAATGCGCCAAACCTCCTAACAAAGCAAAAGCACTAAAGTGGTGAAAACTGAAAATAAAATAGCAACCAATAAATGTAAACCAGTAATAAGGAAATATATTAAAATTAATATTTCCTATACACACACACATACATATATATAGCGACTGCTGTGTGGAAAGACTTCACAAGTCAAGCAACTCGATAACATAATACTCGCTAGCAGCTCTAACCTTAGGCGATTGGATAATTGCCCAGGCCCCACGTAGAAAATATATTATCTACTTATTTTTAAAGAAGAAGAGTACAATTACTTTTTATCTTAAAAATTGAATAATATCTCACTAAACTGTATCTTTAAAGGCCTCATTTTAGTCAAAGTCCTATTAAAACTGTGCAATAATTACTTCATAATTTAAAAATCAAACTTTTACCTTGATAGCTATTTTTCCAAAATGAACTTCAATCTAACTATCACACCAACTTTTTCAACTCAACCAAAACCACTTCAAGATAAGTATTTTTAAAAAATTAAAATTAAATTTTTTTACTTAAAAATATGAAAAAAAAAATTATGCCTAGACCACAGCGAGCTTATTATTTTTTCATTCAAACATTGTGTGTGTATATTACAATTATAAATCTATAAGAACTATTTTAAAATTAATTTTATTATAATACAATCTAAATTTATATCCTCACAAATATTCAAAAAATATTTATTTTACCTACATTAATGAAAGGAAAATTTATTCCTAACCAGTTATATAATTACAAGGGGAGTAGAGTCTCAACATCTAGCCACTTAATTTGAGCTCATGGTCCAACCTTATCGTTCGGCTGCCCTTGAAAGCTAATATATAAATAATATATTTAATCATGTGCGTCAAAGTTCACACGGCCATAAACTTTGGAAAAGTC

>Cs1g26550

TGGGTTATTATAATATGATATATATCATAATTTGATTCACATACAAAAATGTAAGAATGCCGCATACTTTGGTGGAAGATATAAAAATTACTCAAAATTTAATATTTGAGTATCAATAAGGTAACGGCTATGTTCTCTAAAACACCTAGGGGCCTCGGCCACCTTTTGCATTGCATTTAATCTTGCTGCTGCTTTTGCCCCGTTGCGCGTTAGGGAATGTTTGGGATTGCCGTCAGAAGCACTCACCACTGCTTCATTAGCTGATCACGTAACCGCAGCACTGTTTCCCACCTTTAAAATCCCAGTCATTTTCATTTTTTTTTTTTTTTGGCTTAGTTGGTGTTGCTTTAAGAAAAATATCGTTACTTCCAAGTAATTGATTACTTTCGTTTTGAAGGTTGGGTTATTATAATATGATATATATCATAATTTGATTCACATACAAAAATGTAAGAATGCCGCATACTTTGGTGGAAGATATAAAAATTACTCAAAATTTAATATTTGAGTATCAATAATTTTTTTTTTTTTTTTTTTTTTTTTTTTTTTTGGGGGGGGGGGGGGGGGGGGGTTTGGTATGGTTTGGTTTTCTATATAAAAAAGGAAATATAATTGAAACGAAACAATTAAATTAAATATGGTAATCAAGAAAATTGCCGGTAGAAAAATAAGGATTAAAATATTGATATTTCCAAAAACGGTTAATGGCTTCAATGTTCACGGTTTATGACCGTTAAGTGAAACGAGACTATTTATTACATTTTGAAGAACGAGAGGCCCCCAACCACGAGAGAGAAAGGAAGATAAATTGAAGAAGAAGAAGAAGAGGAGAAGACAAAAAACAGAGCAGGAGAGCTAATGCTAAAATACGCAGGAGGGGACACACAATTCCACAACCCCGGCCCATAATAATAATAATCGATCGATCAACTTTCTCACACGCCAACTACTCTCTTTTATTTTATTTTATTTATTTATTTATTTATTTATTTATTTATTTA

>Cs1g16360

TGGATACGTTACTAGACTGTTGTAGTGTGATAGATACTTTACTGGACTATGGTCGTGTAATGCATACTGTACTGGACTGTAATTGTGTAATGTATACTTTATTGGACTGTAGTCGTGTAAAGGATACTTTACTGGACTGTTGTCGTGTAATGGATACTTGGTATTATTGGTTTGGTTTTTGTTGTTAAACAATATTTTGAGTCATGTTGATCCTATAGATGCTAGTTGTGAGAAAGCCATGTTGTCTTGTATATGGAACATTTTTAAGTTGCAAAAAAAGTTCTCATATTTGTTGCTTCTATATTGCGTGATCATCTCGACAATTGATAATAAATTATAGCAATGATAATAAATTTGAAGTATTATTTTTATTACAAACTAAAATTGATGATCTAGATACACATTAGAAGTTTAGACTATGGGCTAGCTCTTGGAACATTTTAGAAAGATATGGTTTTTCATTATGGTGACAATTTAACTAGAATTCATTACTTTGCATTTTCTCCCAAGATAAAGAATCATTTACTTTGTGTATATTGGGTTATGGTTGTGTAATGAATTCTAGCCTCCAATGCAGTCATGTAAAGAATTTTTTACCCGACTTCTACCGGACTGTAGCCGTGTAAAGAATTCTTTACTCGACAACAGTTGTGTAAAGAATTCTTTACACAACTGCAGTTGTGTGAAGAATACTTTGCTCAATAGTAGTCGGGTATGAACACCTCACTCTACTATATTCGTGTATTAGTTCTTTACTCGACTGCAGTGTGTAAAGAATTATTCACTTGACATGCGTCGGGTATGAACACTTTATTTTCAATGTTACAAAGAATCTATGGTTTATATAGTGGACTGTAGTTCAATGCATGCTTTACTCGACTGTAGTCGTGCAATGAATGATTTACTTGACTGGTATGTCGTGTAATTTTTATGATTGGTTTAAAATATATTATTATGTAACTTTTCAATCATTTTATATATTGATTGATAAATTTTTTTTT

>Cs1g15170

GTTATGGGGTTTATAGGAAAAACCAAATATACCCATCCTCATATGGCTTAATAAACATTGCTTTAAAACCCAAAGCTTTTTGAATAATTTGCAGGAAAGTAAAGTCGAGTATCCAGAAAGAATTTCTCCGTGAGATCAAGAGAAAAAAGAGACCCCCTTGGCGTGGTTTTCTCGGGGTTTTTATAAACACCCCACACCCACGACCTTAGATCGTGCCAATAACCATTAATTGATCAAATTCGTGCCCTGATTTTTCGGAGGAGCTTTGTGGTGGTTTATTTTAATTGAGTCTTTATTCCTGGTTATCACCTGGTCGAGGTGATCTGCTCAAGTTTAGACCACCTGGTCGAGGTGATCTGGTCAAGCTCATAGCACATGGTCGAGGTGATCTGCTCGAGCTCATAGCACCTGATCGAGGTGATCTGCTCCACCTCATAGCACCTGGTCGAGGTGATCTGCTCCACCTCACAGAACATGGTCGAGGTGATTATTGTGATTAGCCACTTCGGAATTTCTCTTCACTATTTTTCTCACATTTCACATTATTTATATAAATATTTGTATTTTAAATAATGAATCCACATCTTAATTAATTAATGATCAATATAATTGAAAATGGATTAACCTTGAATACTAAAGATATACTCTGACTTATTGTCATTTTGTTTTGAATGTAAGTATTAGCAGTATCACTTTAATCCAAAATCTTAAGTCCAGCTCAATATATTTAACCAGACAAGTTCAAAAGATCGTCTAAAAGTCTAAAAGGTTTCTTTTATATTTATTGTTATTTTACTATTCTAGCTAGTGATTTTACTTTTCTCCTGAGCCATTTTTTATATCCAGCTGGGACATTTCAGAAAACGTCACTGAACCACAACAATTTGTTAGAATATCAAGGAAGGATAGGCTTATCCAGCTCTCGTCCACATGTCACCGCCTAAATGGGATTCCATATCTCTCAAAACAAAACCCAGTTCGCCACGTGTCCCGCTGACATC

>Cs1g10440

NNNNNNNNNNNNNNNNNNNNNNNNNNNNNNNNNNNNNNNNNNNNNNNNNNNNNNNNNNNNNNNNNNNNNNNNNNNNNNNNNNNNNNNNNNNNNNNNNNNNNNNNNNNNNNNNNNNNNNNNNNNNNNNNNNNNNNNNNNNNNNNNNNNNNNNNNNNNNNNNNNNNNNNNNNNNNNNNNNNNNNNNNNNNNNNNNNNNNNNNNNNNNNNNNNNNNNNNNNNNNNNNNNNNNNNNNNNNNNNNNNNNNNNNNNNNNNNNNNNNNNNNNNNNNNNNNNNNNNNNNNNNNNNNNNNNNNNNNNNNNNNNNNNNNNNNNNNNNNNNNNNNNNNNNNNNNNNNNNNNNNNNNNNNNNNNNNNNNNNNNNNNNNNNNNNNNNNNNNNNNNNNNNNNNNNNNNNNNNNNNNNNNNNNNNNNNNNNNNNNNNNNNNNNNNNNNNNNNNNNNNNNNNNNNNNNNNNNNNNNNNNNNNNNNNNNNNNNNNNNNNNNNNNNNNNNNNNNNNNNNNNNNNNNNNNNNNNNNNNNNNNNNNNNNNNNNNNNNNNNNNNNNNNNNNNNNNNNNNNNNNNNNNNNNNNNNNNNNNNNNNNNNNNNNNNNNNNNNNNNNNNNNNNNNNNNNNNNNNNNNNNNNNNNNNNNNNNNNNNNNNNNNNNNNNNNNNNNNNNNNNNNNNNNNNNNNNNNNNNNNNNNNNNNNNNNNNNNNNNNNNNNNNNNNNNNNNNNNNNNNNNNNNNNNNNNNNNNNNNNNNNNNNNNNNNNNNNNNNNNNNNNNNNNNNNNNNNNNNNNNNNNNNNNNNNNNNNNNNNNNNNNNNNNNNNNNNNNNNNNNNNNNNNNNNNNNNNNNNNNNNNNNNNNNNNNNNNNNNNNNNNNNNNNNNNNNNNNNNNNNNNNNNNNNNNNNNNNNNNNNNNNNNNNNNNNNNNNNNNNNNNNNNNNNNNNNNNNNNNNNNNNNNNNNNNNNNNNNNNNNNNNNNNNNNNNNNNNNNNNNNNNNNNNNNNNNNNNGCTAG

>Cs1g02650

GATTCTGTTAAAGAAAAGGGACGCCTACCGATTGTTGTTAGTATATTCTCTTTACAATTAGGAGGATTTTGATTGTTTTGTCTTTTTCCACATTTTGTTGTTAGTAGAATTTTTATGACTAGAGTTTTTATTTAAAAATTTTAATTGTTTGATTGTGAATTTTTAAAAGTTATATTATAAAAAATAAAATAAAATAATGCTGTACGGCCAAGGCGGATGTACGGTACAGCAGTTTTATTAGTAGAGGTAAAATAATTTATTTAATTTTTAAAAGTTCTATTAAAAAAATAATTAAATAATAATAAAAATTTTATTAAGAGTTTTTAAAATTATATAAGCATTTAGGCTCTATTTGGTATATCTTTTCAAGTAGAGTATATTTAAGTAAAGTTTTTATTAGTAGAGTTTTTACAAGTAAAATTTTTATTTAAAAATTTTTAGTTGTTTGATTGTTAATAACAACTTTTATTAAAAATTATAAAATTATTTTAATAGGTAAGTTTTTTTAGTTACGTTATAAAAAAATAAAATAAGATTGTTCAATAAATAGAGGATATTTTAAAATTTAAAAAAAATTTCAACCTCTACTTCTAAAATCCCCAAAAATCTTAAAATTTAAATTTTTACTAGTTGAAGTAAAATAATTTATTTAAATTTCAAAATCTTCATTAAAAACTCAACCAAATAATAATAAAAATTTTCATAATATCTTATAGAATTAAATAAACACTTATGACTATTAAATAAATACTTATAATTATTAGATAAGACATATTAAATAGATAATTAACGTCAAATATTAAAATTACTTAATAATGAATGGTATTATTAGAATATATTGGGTTGGTTGATGAGTGGTCGACAGGTTAAAAGCTCAATTAAATAATAACAAAAATTTTGATAAGTATTTATAAAATTAAATAAATACTTATTAGTATTAAATAAATCACGTCAAAAACTATTAAATAAGCCATAAAAAAGGGTAGGTGATTACGTCAAAT

>Cs1g09256

GGTATAGTCAAGACGAAATTGAAAGTGGTTTGAAATGGGAGAATGGTCAGAGAGAGAACCTAAGAGAAGAATTTTTTTTTTTCCAAAAGTATTAACAAATGAAGGTTATTAAGAAAGTACAACAAATAAATGTTAGTTTAAGAACTGATAAGAATATATACGAAAAGGGATTTGAAAAAAGGCTAGTGTTATGTTACGTTAAACGTAACATACACGCACTCACAACAACAGTTCTAATGGTGGGGCCCGCTATTGTTGCTGTGAGTGTGTGTACGTTACGTTTAACATAACATAGGGTGATCTTAGAAAAGAATGTTAGAGGTGTGCTAATAATTCAACTCTTTTTTATTTTTATTTTTGGCCACGTAAATGCGTTATTAAACTATATAGTTTAATAGAGTTTAGAGTCGGAAAAAACGCTTGGGCCTACCCTCCACATGAGGTCTTGGACAAGGGTTTAGGAAAAAAAGTATGGGCTAGATTTGATCCTCTATTTAAAATTTAAAATAATAAATTAATTTCTAAAAATAATAGAAAAATAAAACTTAAATTCTGAATTTATAAATGAATGACGATTCAACCTAAAGTATAATACTAAACTCTTTCTTAAGCCTTAATTAATTAAAAGAGTGCTTGAATTAGAACATTCAGTACTAAGAAATTAAATATGTAAGCTTTTATTTACTTCATTTTTTATTTATTATTTTATTTTAAAATAATTATTTTTATTAATTCATTATTAATTATAACAACTTGATAATTATTAACTTATATAAATTTACAAACCCAAGCTTAAGCTGCAATTTGTTATCTTTAATAGAGTTAGTTCAGACACACTACACTAATTCTATCAAATCATACTAATAATGTATTCGCATGAAAAAAAAAAATTGAGTGAGCTCGCACATAAGAATTTTTATATTATTATAAACGACTATTTAAGAAATCTACTAATGATATTTGTAGCCGTGTACACGAGCCACTCTCATAATTGCTTTCAT

>Cs1g21960

CCCAACCCAATCCGTAAAAAACTCATACGGGTCGGGTCGGTTTCACAGATTGAGCGAGTTGATTCCCACCCTGTTATTTTTATGAATAAAGAGATTCTGTCGCAAATTTCATTTCTTTATCACAAGTAGGTCGTAGACAAGAGAAATGAAAAGGCAAGGTGCTCTGGTTCATTCTTATTGCAGTACCCATCCCCTTTGTGAATTTTCCATATACATGATGGCCACATACCAGCCATTCCACGCACTCTTTTCCCTTGGTCGTATGTTCCCCCTTGTACAGTGAACAGGTAACAAAAGAGTGGGTCTTTTGACAGCCATCAAAACTCTTTATAGAGCCCCTTTCTAATTGAAATTAACTAAATAACTAAATATAATTTAATTCATTTTTTTGTTTTTTGAATGCAAATTTAATTAAAAAACCTAACTAAATTATTATTTTAATTAAAATTTCACACCCACCCTATTTCATAATTTATAATTATATTTTTATAATATTTTAAAAAATTATGAGATTTTAGAGTGTATAATAAATATAAACTACATAATAAGTATAAAAATTTTAACAATATTGGGATAAAAATTTTATGGAAAAATGACTTATTTTAATATATGGAATTTTATATTATAAAATTAAAATGGGATATTTTGAGAAATAAAATGTAAAAGGGGGTATTAGGTAGATTTTAGAGGGGTGTTAAAAGCTCCTCCTTAACACGAAGCCTTTCTCTTCTATAAAGGCTGGCCCATGAGCCATGATACAGTGACATGGGGCACAAGCCTCACCTCCCAATCTCGCATGCCATCTGCACAATCTCCTCGTCTCCTGGCACTAGGATGTGTCGCGCTATGATGTTGACATTTCAAAACTGACGTCCTCGTTCGTCATCGCAACATCAGTCCAGCTTGAAATCGTTTCAGTGAGGATCATCACCTCATCATCAGCCTCAAGAGTTGCTTTTTACTGCCAAAATTCGAAATTAAACAAAAGAACAACAAATAAA

>Cs1g23970

ATATACTTCCACAACATATTGGTGCTAAAAGTAAGCTCTGCTGTTGAGCTATATATATTTTAGTTTCGAAAATGAGCTGGGTTATAAGACTGTTGTATTTTTTCCACGAGTTTTCGGTTTTGTACGTTGGAGATAGATCAGTAAGATCCTATAGAATAAGCTTTTAATCGGTTTGTTTGAGGTTGTCTTTGTTACAAGCGTAAAGCCCAGCGTTCACAGCGGAGAGAACATCTATTTTTGAAGGTATTGTTTGCCCACTCCTCTTTGAAAAAGCTAAACTGATAGTAACAATAATTAAAGGTGGTGATTGGACGCAATGAGATAGGAAATGGTAAAAAGAATAACCAACTTAAACTAAACTAATGTTGGCAAAGACAGAAGTTTGACACAGAGAGAGAGAGAGAGAAAGAGGGAGAAAGAAGCCCTGCTTTCTCGCAAAGCCAGAGGACAATGACAGGTTTCCATTAGCTAATCTAGATTAGGAATTTCACCAAGAAAAGCCCTTTCTCTCTCTTCCCCTTTTGAGAACCCTTCAATCTTCTCCATTCAAGGTATTTTTTTTCCCTTTTTTTCCCTTTTTTTTGAATTTTCTTCTCGGACATCGGAATTCTTTAGCCATGCATATGCTGTAACAAAATAATCTTGTAATTTTTGACAGTGTTTTAATTTGGGACATCATTATTAGTTTAACAGATATAATTTATTAAGGCAAGAGGAAAACGGTTTGGCATTTGTTGATGATGGTGTTGCAGCAGGGAGAACAAAAAACAATAATAATTTATGTTATCTAGCTTTGTCATACTGGAAAAAATTTTATATGTCTACAGGCTAGTTAATTTCATTCTACATTACATGAATTGATTGATTATTCATTCAATGGTTCACAGTCTTTGAATCTAATCAATATCTTCAGGGTCCTCGAAAAGACTTTTGACACTGGCAGTTAATTAGCTAGGGAAGAGGTGGAGTTTCTTGGGGTGGAAGTTCTTTAGCTTGAACAA

>Cs1g09860

TACTTCCTAAAAAAGTGCAACATAGGTCACATTCTGACATCTCAAACAAGAAAGACATATGTGCCTACTTCTTTAGATCAAGTTCCATCACAAGACTCATCACCAAATGATCCAGAAGAGAGTGGCCCGCCAGCTTACATTCCCAAGGCTCCTTTTCCTCAAAGGTTAACAAAGGTAAAGAAAGGGACTTCAACAGGTGNNNNNNNNNNNNNNNNNNNNNNNNNNNNNNNNNNNNNNNNNNNNNNNNNNNNNNNNNNNNNNNNNNNNNNNNNNNNNNNNNNNNNNNNNNNNNNNNNNNNNNNNNNNNNNNNNNNNNNNNNNNNNNNNNNNNNNNNNNNNNNNNNNNNNNNNNNNNNNNNNNNNNNNNNNNNNNNNNNNNNNNNNNNNNNNNNNNNNNNNNNNNNNNNNNNNNNNNNNNNNNNNNNNNNNNNNNNNNNNNNNNNNNNNNNNNNNNNNNNNNNNNNNNNNNNNNNNNNNNNNNNNNNNNNNNNNNNNNNNNNNNNNNNNNNNNNNNNNNNNNNNNNNNNNNNNNNNNNNNNNNNNNNNNNNNNNNNNNNNNNNNNNNNNNNNNNNNNNNNNNNNNNNNNNNNNNNNNNNNNNNNNNNNNNNNNNNNNNNNNNNNNNNNNNNNNNNNNNNNNNNNNNNNNNNNNNNNNNNNNNNNNNNNNNNNNNNNNNNNNNNNNNNNNNNNNNNNNNNNNNNNNNNNNNNNNNNNNNNNNNNNNNNNNNNNNNNNNNNNNNNNNNNNNNNNNNNNNNNNNNNNNNNNNNNNNNNNNNNNNNNNNNNNNNNNNNNNNNNNNNNNNNNNNNNNNNNNNNNNNNNNNNNNNNNNNNNNNNNNNNNNNNNNNNNNNNNNNNNNNNNNNNNNNNNNNNATACTCAGCATGTACAGGATCCTAAGAAACACACCCCAGTTATTCTAGGTCGTCCTTTCTTAGCTACAGCTGATGCTCATATTAGTTGCAGGACTGGAAACATGCAATTGTCTTTTGGTAACATGACCA

>Cs1g19010

AGATGTGTTGTAGATATTCTATATTGTTAATCTATAACTCCAATACTCATAATTGCTCCCACCAAGATACCGGGTAATCTTGACTACAAGTGTGTGTCATGCCCATTGGTAACTCAAATGGAATATCAATTACAATCATAAAATCATAACTAACTCAAGATTAAGATTACAGTAAAATCAATGCCTATGAGATTTAATAAGTCTGACAATTATTACAAAGTTAATTAAATATCATATGTAATCATGTTCAATGTAGTCATACTACATTAATAAATTCATACATGATTAAGACAAATCATTCAATGAATTTATTACAGACTAAACATAAATAGAGCGCCCAACTCTATTTATCAACTGCGAACTTAATTTATTTAATCATAAGATAACTTGTATAACTTGTATTTATGTATTCTGTGAATCCATATGATGATCACATAAATACATATAATATGATTAAACGGACCTTATTCAAAATATTTATGCAATTGAAAATATTTCAAATATTTTATTAATCAGAAAATAAATAAATTAATATAATTTAAAGAGCATATAATCCCAACAGTATGAAATTGTATGAAATATAAAATTAATTTTAAAAATGAAAATTTTGAAAAAGTACTTTTTCCTTTTCAAAATTTCTTGTAAGATAAGATAATTTTACCAAAAGTGATATATGAAAAAATTTATCAAATATTAAAATTAACTTTAAACTTTTTAAAATTATTTTTGAGTCTCTTCAAAGTAATTTTGAATAGGCCGTTAATTGATTGACTTGATAAGATAATAATTATTAATTTTTTTAGTATTTATTCTATATTTTATTTTGCTAGTAAACAACTTCTTATACCCAGTACATATTTGAGAGTGGTTATCTATTTATCATATATATTTCAAAACAACAATAAAAATTATTAAAAACATGACAACCACACTCTTGATATAGAACTATTTTGACTTATATTTTTCAACTTACAGTTCAATTCCTACAGTACCGATATTCA

>Cs1g21340

TCTTAGTATAATATTTTATATATTTTAAACCAGGCTCATTCGAAAAATAAAGTGAACAGAAATTATTCGAGAATATTATATTATTTTATGAAAATGGGTTCCTAGAGTGTCTCTTGCTCATGCTTACTTATAGTGGGCAAATCTTCTTTGTTCCTGCCTAGAGATGGCAATTGTACCCGCAAACCCGCAAACCCGCCCAAACCCACCCGCCAAAACCCGCCCGTTGCGGGTAATTTTACCCGTTGCGGGCGGGTTTAGTATTATAAATTAAAACCCGCACATGGATGCGGGTGGGTTTGGTATTAACCTTGTATGTATGTGGCCCATCTCTTATTCTAAAGTCATAACCTAAAGAAAACTAAAGGCATTTCCCTTCGAATTAGTATTTGAAAATATTAATCTTAATTATTATTATAGGCATTGTGTTGGATGGGTGCTTATGGTGGTGAATGAAATGAATATCTTCCCTTGGATCTTGTTTTAAATGATAATATGAAATGTAATTATTATTTTTAGATACTAGTTTGTGTTTTTTAAATAGATTTGTGTAATTTATAATTAAATTTGAGAATTTGTATTGAATTGATGTGGAAATTTTAATTTTTCATTGACATTGTTTAAATATAAAATTGAGTATGCTATATGGAATTTGAGGTTATTGTTATAAATTTATATATTAAATATTTGTGATTTTAAATACAGAAACCCGCAAAAAATCTGTCGGATACCATTGCGGGTTTGGTAATTGTTAAAACCCGCTGCGGGTGGGTTTTTTTAAAAAAATTAAAACCCACTGCAGGTTGCGGGTGAATTTAATAATTTAAATTTTGTGTGGGTTTGAGTTTAGTAATGACAAACCCACTACGGGCGGTGCCCATTGCCATCCCTATTCCTGCCTGGACTCGAAGGCAAATCCAATTTGGTCGGGCCACACGTTTCCAACATGTCACAAAACGTTGTCGTCTCCTAAGTCCGCACAATCCGCTGTCAGACACACACGA

>Cs1g16500

ATGCCCAACGGCAGTAATCCTTCAAATATTATCCACGACATGATTTGGGTTCTTGAAATTTTGATTTGAGTATATTTTTATCTCTATGTTTTTTTGTTTGATCTGTGCATTGGGGGTTCCAACAATGCACCAGTAGTTGGTGGTTGTTTTTGCAAGGGGAATCCGTCGTGGTTTTAGTGTGTTTAGTTCAAATGACAATTTTAACCCTCTAATGTCAGCAATATTTTAACGAGGTTATGATGGAAGTGGACCAATGATAATTTTATTAACTTCAAGTAGATTGTTGTAATATATATATATATATATATATTTTATAAAATGTCAAAATCAAGTGGTCGGGAGATAATTTTTCTTAATTTATTTTGAAAGGAGTTATAATATGGGTAATAAAATTTTATTTCATCACTTTTTTTTTTATTACATGAGACTATTGCTCAGATTACAACTCATATTACATGAGAATATAACTGATATGTACAAATCAGACTATAACTGGGACCAACATACATCAAATCTAATGGAGCACTGCCCAGATTTTAACCCTCTTAAATATTCGAGGGATGTCTACTCCTCGCACTGGGGCAAGGAAGCAGACTGCCTTCACTCAATTTTATTTGAGGAAGGAAACACCCCCACATGCATTGTGGGGGTTCTCACCACTTTAGCCGAGTGGCTATTCATCACTTCTTAAAGTATATTTTATGCAAACATAATTTATTTATTTATATCGAAAACCCTGTTCAAATGACTAAAATGTCCTTTCGGTTGCCTTATTAGTTAATAGTTATTATACAACGCATTCTCTTGGATTGTGATGGTCAAAATATAATATTAAAAGGATGTTATGGTAAATTTAGAGTTTTAGTAAGGTTGGGTGGGGATCTATTTGATACAAGCGAAAAAACTATATCTTTTTTCAGGAAACAATAAAAAATCAGGGATTTGGAAGTTAGTATCCAATAGTATTTTAATTGATAAAAAATATTTTCTCTGAATTAACG

>Cs1g25310

GGAGGTATTTATATTTATTACAACTTAATCACAATGATAAATTAACCTAACTAATAAACAAATAATAACCACACATCAAAACTAAACAGGCAGATGTAGGAAAGGTTGGGCCACCTTCCTGGAGCATTTTAGCGGGGGCAGGGGCTCATGCAACCAAACATTAATGAATTCATTAGCATTCCTCTTCAGGTTCATTGGAGTCTCAGGTAAGGACTAGAGAAGGATGAGGAAGCAGCGCTCTGAACTCATTGATTCAGAGACAATATTTGGTAAATTGCAGTGCTTGCTGAAGGCGAGGTATGCTTTTAGGTACTTACCACATTACCACAGTTAGCACCACAAACTTGAACTACTTAAAGAAGCTTCCATGGGCTAGATTAAAGACTATTTAGGCTTTAAAGTTGTATGGATCAAGTTTATGATCCAACAAAAATCCAGTTTGGGTCTAAGATCAATGCCTAATCCTACCGTGAAGAAAGTAAACTGTCTATATGGTAAATCTTTATTTCAATTATATCATAGTTATAGAAGTCTATGAGCTTATTTCATATTCAAAAACTAGATAAAAAATTGAGAGTTAGTCTCAAATTTACATTATTAGTCTAAAATCAATTCTGAGCTCGTGTGGTTATTCCTTATCAATAGTGAATTCACCATTGTTGTGTGACTTCTAACTTCACCACTGTTGGTGTCTACGGATAATTTTTTATTTTTCAAATTTTATAATATAATTCTAATGCAATCACTTAAATAATTCTTATGCAATCAAGATCACCGTTACGTTTTTAAATTCTATTTTACTAGAATTTTTGTATACATGGAGTTTTCTAACAATTTTTCAAATACCAAATACATAAGTCACTTAAAGAGCAATAATTCTAAGGTAATAATGCAGTTACTTTCCCTTGTAGTGAAAAAAAAAAATGGTCGAGTATCCTTTCCCTTTTACAACAACTCTGGAGGGGAGATTTTTGAGAAAACATTTGGGGTTGGTGAAATAC

>Cs1g09210

ATCTCGCCTCCCACCTGAAGTGTCTCCTGAAATAGTCTCCGTAGATCTTTCCCCAATTCTGGAAGTTTCACCTTCTACCATTTTCTCAACGAACTATTTCCACTTGAAAAACTGTCTCTATGATGTTAATTTCACGTGAATAAATACACCAGGAAAGTTCCCCGGAGACTGGACGGGAACTCGGTCCCAATTAAAAACCAGAATCCTTAGACTCTTATCGTATACGTGAACAGTACCATATCCCCCAAGTGCGAACCGTCGGCTCTAATACCACTTGTTGTGTGCGGAATGCGGAATCAAGCGCACCAAATAATTATAGAAAAATAAAGGACACAAGAATTATGTAGTTCGGTAATTAAACCTACATCCACAGAGGCAAGAAAGAATAATTGTTTATTTAACAATTAATAGAGTACAATATTTGAGTAACGAATCTCACTTCCAAAAACCCAAATATACCCAGTACTCTCACACTCTACAAGAAAGATAAATTTCCCAGACACACTTCTCTCACTTCTCTCAAAAGCTTAGAAACTTGTAAGCTTAACAATTTTTTTGGATGCTTTACAAATGAAGAATGCATCTCTATTTAGAGCAAAAAACATTGCACTATTGCACCATCCGCTTTGCAATTTGTCAATTCTCCTATCTGTCAATTTGACAAATTTTGCCAACCTTTCTCATTTTTCCTCATTTGGTGAAGGATTGTCAAAAGCCAAATATTTTACAGCTTACAATTTGGTGCCGACTTTGAAAAACAAAAGCAATGCATGTGGCTCACTTTTCTTTTTCAACCAAAACTCTTTGACTTTTCAACACTTAGAGTGTTGATTCCAGTGGTAGTGTCGTGTTTGATATTATCCTCATGCCTTACTATTATTTATGCTCGAAGAATGAGATTTGCTCATAACTCTATGGATACATCACCGATGGAGAAATAATTTCTTATGGTTTCTTATGCAAAGCTAAGTAAGGCAACTAGTGAATTTGCATCGTCAAAC

>Cs1g15310

CACTTGTTTCATTTAAAATTTGTTTATAAGTCTTAAAAGGACAAAAATACCATTCTTGACGAATGTCTGACGAAAAAAAAAATAAAAAAAAGTTACAGAAAATTCATGAATTGTCTTGTATGTTCAGCATCTAAGGTATGAACATCCCTTGCACCTTGACCTCTGGTTGGGTTATCGCTTTAGCTTTGATGCATAGAGTGCAAATGGGTGTGATCTTTGTTCAAATGTTTTACTTGCAATTGGCTGGAAAGTATATATCACTGGAAGGTATACGGGATGCAGATCCTAGGCTTGTATAGTAGTTGCAGGCAGATCTTGGAGACGGATGCTGAGTTCATTGATTCACAAATGGCTTGGATGTGAACAAGTAAATACTTTAATACTTATAACAATAATAAATAAAAAAGAGTGCAAGTGAGAAGCATCATAATATTTCTTAATATTTTTTGAATAAAAAAAAGTATAGAGTGTTTAATTAAATTTAACATTAATTAAATCACAAAAGATTAAAAAACTGTAGTAAAATAGCCTAACCCTGTTTTTATTGTGATTTTGACAGGTAGATGGAAGTCGCTGAACCCCTGTTTTTATTGTGATTTTGATAGGTATACGAAAATAACTAAACTCTGTTTTTGTTGTGATTTTGATAGGTATACGAAAATAATTTTTATTGCGATTTTGACATGTATACGAAAATAACTTAACTCTGTTTTTATTGTGATTTTGACATGTATACGAAAATAACTGAACTCAGTTTTTATTGTGATTTTGACACGTATAAGAAAATAGCTAGAACGCTATTTTTATTGTGATTTTGACAGGTATACGAAAACGTATAAGATATGATATCATTTTAAAAACTTTAAATTGGGAGCTGGCTAAGTTACAGATTCTGTAACATACAGATCCAACTTAGCCAGCTCCTGATTGGTTAGCTAGCTACCAAACAAAAATAATTGCATACTTTTTCTTCATATGCAGGACAGGTGTGGTGTGGTGCA

>Cs1g16390

TTTTGTTTGAATATTAATAAAAAGAAAATAATTAAATTACCAATTTAACCTAAAAAAAATAATATTAATATTTAATTGTCAAAAATTGTATGCAAACTACCAACTGTGCAAATGGTGCTTGTAAAAAAATTAATATAATTTTTTTATTTTCTAGAATTAAATTAGTAATTTAATTATTTTCTTTTTATTAATGTCTAAGCAAAAAAAATTATTATAAAAGGTTGATATTGTAAAAATAAGCTAAAAGAAATAAAAAATAACGATATGGGTATCAATACTTTAATGACAGAGATATTTTAAAATATTTTTAAAATTATAAAAATTAAATAGATATTAATATTAAAATATAAGGACTAAATAAATTTTTTCCCAAAAAAAAAAGAGTGACTCATGTAATGTTACCCTTAAGGCAAATGCTATGTTACAATTAATGTAGCATACACACTCAACAACAACCACACAAATTGTGTGGGCCCATTATTTATGTGGTTATTGTTGAATGTGTATGTTACATTAATTGTAACTTAGGGGCTCCCCTACCCTTAAATAGATCCAAACTCCTGCTTCTTTTCCGTATTTCCCCTGTTGCCCTTTGTTCTAGTAATTTTATTCCCTACGTGTTGCGGATCAGATCTCCACGTGGCATTCGTTCCCATCGTTCTTATGCCACGTGTTGCTCCCAACAAAATCTATGTTTGCTCATTCATACGTGAACCTTTCGGATTCCTAAACGTGTCGGCTCCAAAAGACCAAATCGGACACGTATATACTTGAACTGGAATTTGATTTGTGGATAAACGAGGGATATTATTGTAAATCAAAATTTCCTCTGTCGCCAAATAGGTACCATGATCTTGTTATACACACCTGTGGGACCAGACGTAACCACGTGGACCAATGACAGAGCAGTTTTAGATGGAGAGCTCACAGCCACTAAACTGATTAACAATTTTCAGCCACAAGCGACAGCTTTGACTGCATTTCATCCTATAAAAGATTGA

>Cs1g26450

AGTGCGTGTTTGTTAATTCGCGCTTTCAAACAGCAAGAGATTCCATGCATGTCTTTTGACGAGGTTTAGTTGTTCAGTTTCTTGCATGGCCCGTCTCTTTTCATTACATGGATTCAAAAAACTTACTCTTACGAGTTACGTCATTATCAATCATCTCAGGACTCGATGATGATGGTGCGTCATCATCAAGCAGATGCAGTCACATGTAACTCCAGAAAGGCCAACGGCTTGTTACCTTAAAAAGTGGCAAAAGTTTCAGGTCCACGGTGGCAGAAGCGACCGGGGCATCTACGTTGAGGGTGAGCCGCCCCAACTTCACGCTCTTATATATGAATCTTGCTCCTGACCTGACCGCATCTGCTATATCTGGCCATCTGGGGTTGAATTAAGGAACCGATTGTATGCTAACTAATGGACGACGTTTTTATTTGAACTCCCCTACTCATGAGTATTGCCTGATTAATTTATTAACAAATTAAATATTATTTTGTTTTTATGATCAATCACTTCTTACAAAATTTACTTACTTGTTCGATCATCATACATATTATTAGATCAAATTACTAAAAAAATTTATACATTTGTTGTTTGCACATATATGCAACTTCTTTGTCACTTCTAACTATAATTAATGTTGGTTTCGTGGCCACTAATATGTGTAATATTATTTTATTATTCTTGATGTTTGTCATCAGTTGTGCTGTGTACTGCTACCACGTACCAGCAGTGAAGGTTAATTTACTGTCCGTTTTGTGCAGGCCACGTCTCTCGTTCGTCAGATAAAACACGCTTCATTTTTGCTCTATGTACGATTAATCATTATATATGTTTTCGATTAATATAATAAAATTACTATCACATTTTTATATAGACGCACACTAAAAATAACAACCAGTAAATGGGTGCCACGTATAAATTACTGAACCCTTGACGCGGGCAAAGTCGTCCTGCAAAGTTTCAGTAAATCGGTTGAAAAATAATATTCCGCCCAAAGTTCTCGT

>Cs1g11990

GAATCGAATTTTGCGGATTAAACATCAATCCATATCTGATCCACGATTTTACAGATTATAAATTTTTAATTCAATCCATGCATTTGTGGATTTGATGCGGATTTGACCCAATCCATATTCAATCCACCATTTTGCGGATTGGTTTGCAGGCTAAAAATATTTAATTTCGTCCTATCCACGAACAAACTAACTAAAAGAAAATTAATATAAAAATTATTTTTCTACTGATCAAATTCAAGATAAAATAATATTAAAATAAATTAATTGCTTAAATAAAACTAGAAAATACATTCAGAAATTTCAAAATATAGCACACCAAAAAGAAAAAAATCTCATTTTTGTAGATCAGTACATGAAAAATAACTGTTTAGGAAATTATATTTGTTTATTTAATTATTTTTATTTTAGATTATTATCGGATAAGATATAATATGATGTTAATGCAACTATATTCTAGCTTATTTATTTATTAGTAAGTACTAATACCATATTCACAAGTTTAATTTTTTATTAAATAAATTATTTTTTTTGCAGATTCGCTGATTTACAAAAGTAAATATCCAATCCACCGACTGCAGATTTCTAAATTTTTAATCTAATCTAATCCATCAATCCACAGATCGGATTTGTATGGATCAAATATATTGAGCAGATCGGATTGTATTCTGAACACCCCTAATATTTTGGTCATTAGTTAAAACCAAAAAAAAAAAGTCATTTTAACGGTGATTTTATCACTGTTTTTTTTTTTTTCACCCTCTTGTTGAAAGCACGGGTTCTAATTTGTATTTTATCTGCTTCTAACTCATTCCCTTGAAAAATTAATATGTCTCTTAGTCCAACAACATGTTACGAATCTTCCCTGTTTTTTCAAAGTCCATGTGAATAATCAGCACATGTTTACAACTCTTCCCTGCTGACCTGAAGTCATCCTTGGCTTTTTCCTAAATGCATAAATTATACACGTCGCAACATTTGGAATAATCATTTGATCGATCTCA

>Cs1g06440

GAAGAAAGAGAGTTTCAGGACGTTAATATCACTTAATCTAAGCTCTCTAAACTTGTAAAAATCTTACTTCAGATGCCTTTTAATCGTAATTTATTATTATAACTTTGTACCTTTATTCATTAATCATTCCCATGAATTTTTTTACCTACAAAAAATTCATGTTATGTGGAATATTATTCCAATTTTAGATGGGTCAAACTCCCCACCCTATCATTCTGGTGCACTTGAACCCATCGTATGATAACAAGTTGGAAAAAGTTGAGTTCTAAAGATGTTTTTTTCCATTTGTGGGGCCTACGTATATTCATGTAACTGGATCACATGGTTTCGCCTGGCGTCACTCACGGGTTTTATGATAGTCAGACTCAAGCCAACTCATCTAGAAGCTTACATCTAAAATTCTTTCTTCATTAATTTTTAAAAAAAATTATATGAGGTTATTGTTTAAATTATAATACATATTATATAATATTATTATTGATATTTATAATTAAATAATTAATAAAAATAATTTACATTAAATTTTATTAAATATTATCTAAATTTTTACACTCAAATATATCAAATAATTACTGAAATTATTTTATATTAAATCTTATAAAATATTATCTAAATTTTTACTTTTATTAATATCTAAAGAATATCCACCATCACTAATTATGCAATATAATTAAAAAACATTACCACCCAATACTTTTATTTGTGGGGCAGCAGCCCTGATTAGTAATTAATTATACACAAAATTGGACCACATGCGCTCATGCGCACACATTTTATCATGGTAAGGCAGAGCTCGTATAATATAAATTTACAAGTACAAAAAATTCATGTGACGTTGAAAAAATTGATTAAAAAAAAAAATTCATTTCATTTCGTGCCCTCAGTTTGCTTCGCGTTGCGTACAGAAAGTTTGTACAAAATATTTTGTGTGTCAGACCGTCCAGCACTGCTCTTTTATTGTTGTAAATGGCAGTGATGCAATTTTCATATATTGTTTGTCT

>Cs1g08450

CATTAACATCTAAATAGAAAAAATAAACTTTTTTTACTTTTATCTGAATGAAAAAAAGTCGAAATGACAAGTAAATATTATATTCCAAAAATATCTCTGTAAAATAATTTATTTTCTAAACTATAAAATCTAATCCTTTTTTTGTTTTAATTTACAAGGACATAAATGTCAAATAACTAAATTTAAGATATTTTTTATTATGAAAACAAATAAAATACCATTTATATTTAGATATTTAAAAAAGAAAAATAAACATATCATTCATATTTTTATAATCAGACTAATTCATACTTAAAAAAACTCTTACTCATTTAAATTTCAGATAAAAAAACAAACACTGTCTAAATTGAGTATTGGGTACTTTAGTAGATTATCAACTTGGATAGCCGTTAGATTAATGGTAGAGTTACTATACATATATCTGACTGTCTTGCTTAACCGAGTTACCAAAAGGCAAGTCATCCCAAAACCCAAATCGTTATAGTCAACAGTGTTTTCAAGAACTTTTCAAAAAAAAAAACGTGTTTTTAAGAAAACAAGAACCAATTCCAAGAATCGCAAAATAATTTCAAGAAACAAAAGAAAAAAAAAAACGTCTTTCTTGTTTTTAAGACAAATTTCCGAAAAAAAAATAAAAAATTCTTTAGTCTTGTCTGAGAAGAATCGCAAATTCGCAGTGGTCTCAGAGGATGCTGAATGCCGTCGTGATCATCAACTCAAACCCACTATTATCAAAATATTTACCCATATTGTTCATAGGTAAAATTAAAAAAAGAAAAAAGAAAAAAGAATGACTTGAATTGTGAGAAAGAAGAAAAATATAAATTTATTTCTGTTGTCCATGGTATTTGGTTTGCAATGTAGAAAAGTAAAATTTTGCATGAATCACAAAATCTCTACATATTTTATGTCTTAATAATTTAAGGTAGTTGACTTGTTGACATTACCGTTTATAAGATTCCAGTTCCAAGTTTGTTTCAAAAATCTACTTCACATGGTGG

>Cs1g21350

TAAAAATATACATTTTATGCTTTTTTTTTTTTAACTTTTGCTTAATTTAAGAAATTAGAATGAACTAAAAATAACATATTATATATTTTTAGAAAGAAGGATGTTATACAGAAAAGTTGAAAAGAGAAAATGGGAGGAGCATAAATAGGTGTAGATATTTGAAAATAAAATTTTGATTCGTTTCAGATTAATGAAGTGTTTATGAAAACTTCACATGCTTTTTTTATAACAAAAAAAATGATAGATAATCAATAAAAGCTAAATCACATTATTATTACTAGAATCCGTAAACACTAGGGAGCAATAACTAAAAAATAGAAAATTATACAAACGCAAAAAGATTTTGAAATTAGTATTTTTATCATAAAACGTCATCAACTACTCATATTATATCCTTAAAGATGATAAAATTTCAAAATAAAATTTTATATTTTTCCCCAGTATTAGAAATGAAGACCTCCGTTATTAATACACATTAGAAGAGAATACTAAATAAATTACATTTATTATTTATGATTAAGATTAGTAAGTACTATTAAGTATTATTACAAACCAAATCACACTAAAACTGACAATATATGTTTTCCACCTTTGAAATTTCAATGAATAAACTTAAAAACTCTCTAATGATGTTATAGTAACCATTTACATTGTCAATTGAAACGTGGAGCGATGTGCCCACATCAAGATTACGAATATAATTATATGTCCATGTATGTTTAGCCTAAAATAGTTGTGCATCACTTATCCGTTGCCTTAAATTTTGGTCATTTTCATTAACTGAGGGGGAAGTTTTTAGTAAACTAAAATCTTAAGCACTCCCTCAAAAAGTGAAAAAGTGAAAAAGAGGAAAGAAGAAAATCCTAGACCCAATTTGGGGGAGATTATTATAAGTTGAACTTATCCAGAAAATGACTAAAATTTTATTTTTAATTTTTAAAAAGAAAATAAAGGGATAAATATAAACTAGCATGGCAAGGCTTTTGAGTTGAGTTGTGAAG

>Cs1g25520

CACACAAAAAGTTCATGCACGAAAATATAATCAATAGCATTCATTGCGCATAGTCCATTTGCCGATAAAGTCATAAACGCAATCTTAACGTGTCTTACCCAGAAAATTCTCACAGAAGCTTCTTATTTGTTAAAAAATAAAAGCTTCTACTTTCCTTCCACATGCTATGTGCATATTAGAATGCACAGAGATCATAATTAATCAAATCCATGAAACACTTTCACTGATTTCACGCGAAGCAGCAATATCTTAGGCCTTTGATATGAGAAAATTTGGAGTCACTTTTACAGTCACTTTTACACAAGGTTCAGTACTTCAGTTCACTCATATAACTGGATCCAATATATAATTCATTCAAAGTACAAAAGCTAATTCTCAAATTAATGATTTCAACACAATGCTCTTTGCTGCCAACATAAAATCATGACTGTTAGCATGTCCTCAACTTAGCATCATACATATGAATTCCATCATAAAAAAAAAGTGGGGGAAAAATGGATAAAGCATTAAAGGCTGCTCAGAAGGAGTGGAGTAGGAGCTGATGGGGTTGTAGCCCAGATCAAAATCACTCATTGAAAGTCTGAAATCAATATGACAAAATCGATGTTATGGTTGTTTCAAATGGCGTTTTACACCTAATAAAGGGTTAGCACATCTTTAAATTAAAGAGAGAAGAATAGAGCAAAGCACAAGAAGCAGCAAAGAGAAGATGAGGTGTTCAATAAATCCTACTGGAATGGAAACAAAAACCTGTGTAGTGGAAACTGAAAACAGAGGAATTTAGCTAACCTAATGATTGCAGAACTTATTTAAAATATTACCCTAGATATGAGACTACAAGCAAACAGGGCATTCTGGTTACAACCCTAACAGCCCCTAGAGATATTGTCAAACTACAGCCCTGTATAAAATTACAAGTCCACCTGAGAAAATGTCAAGGTTGTAGACTTGTAGTGATTAGAAACCTTTCCTGACTTAATTTACCTTCACCAGTGAAGATG

>Cs1g14893

GAGAAAATACAAGGGAGAAGGTATGCAATCTATCAAAATAAATAATCATTAAATTATTCATTTCATTCATTCTTGGTATAATCAATAGGAAAATGATATATTGTAGTTCATTTTATTGTAGAGGAAAAGAAGCGGATGTCATTAGCCATTGCTGTGGGAGCGGCTTTATTGATACCTCTGTTGTGCTACTTATGTTATCTGATATTGAGAAAACTCAAGGCTAAAGGTAATCACTTGTACTATATATTATTAGTGTCACTCAATTACAAGAATCTGAATTATACCATCCACGACACTAGAAACCAATTTTATTATTTTCAATTATGGATTTAAAAAAATAAAAAACACATTTGATACCCCTCTTAATTAGTGGAAAGCATGGTGAATCGACAAAAGCTATTACGCGAACTTGGAGATAAATCGTCACTTCCCACTATATTTGGGAACAGGAAAACACAAGCTAATAAAGATCGAACCACGAAGCGTGACTTGAAGATATTTGATTTCCAAACAATAGCTGCTGCCACCGACAACTTCTCAACTGCAAACAGACTTGGACAGGGTGGTTTTGGTCCTGTTTATAAGGTTCTTTTTCTCTTTGTGGTGCTTATCTTTTTTTCAGTCGATACATTTGGAGTATAATTACAAATTAGAGACAAAGCCAGTATGATTTCAGGGGAAATTACTTGATGGCCAAGAAATAGCAATAAAGAGACTCTCAAAAAGTTCGGGACAAGGAATAGTGGAGTTCAAGAACGAAGCTAAACTCATTGCAAAACTTCAGCACACTAATCTTGTGAGGCTTTTGGGATGTTCTCTCCAGAAAGGTGAAAGGCTTTTAGTATACGAGTACTTGCCTAACAAAAGCTTGGATTTCTTTATCTTTGGTATGTCCCTCTTCAACATATACGTAAAGATTTTGCAACGATATATTAATTTCTCTCCCCACTAACCTGCTAATAGAAACATTCCATTTTTTTTTCCATTGTTCGATGAGACAG

>Cs1g19230

GGCAATGGAACTTTAAAATTTCATTTATATGTGATTTTGTGTTATTGCTTTCTTGTTTTATTTATTATTGTAGATTTATAATTTATAGTGGATTTCTAAACTATTATGTAAGTTTGTTTAGTGTCGAACTTTATTAAGTTGATTTATTTGACTTTATTATGTTAATTTGTAGACTCAAGATTGTTATTGTAACAACACTATATTTTTATTATGTTTTTTAAAATTAAATATACATGATAAAATTATTCGAATCAAACCAAATAAAACCAATTCAAACTATGGAATTGAACCGAATGGAACTAAATTGATTTGGTTCGGTTTGATCTTATTATAGTTTCTATTACTTGATCCGGTTTTGGCCGGGAACCGAACTAAATGGGATAATGCCCGGCCCTACGAGCAAAGTTCGTTTTACCCCGAGTTTGGTGAACTGGAAATCCGTCATTTCTCTCTCCTATTGGTGCTGTTGAAACACGTCCCTTTCCCTTACGAGATTGAGACATAATGCCACGCGTACAAGAGAAGCCGCATCTTAAAACGACATGAGCTGCCACACCGACGACAAATGTTTGAAAATTGTAATTAACCTTCGATATTTAAAAAACCTCGAAGTGTATAGTATTGATAGCTGTTAACCTCCGTTACGACTGAGTAGGGTGAGATATTAATTGAAGGACGACGAAGTCAACAAGAATGGGCAGCGGCCGCAGGGCTCTCCTTGATTTGAGTGGTCACCCCAATTTTTTAATTCGAATGGTCATAAGAACAAATGGATGAATCGAGGCGAAATGTGATTTGTGTCTGTGAATGGAGAGAGCGCAGGAAATTTGGCGACTGGCAGATGTTATCTGGTTAACGTCCTTGTAACGTGTCTATCCTGAAATCTAGTAAAACAATTTAATAATAAACAAAGCATATGATATTATAACATATCGATGACTAATCTATCTCCTGTAGGAAGAAAGCTTGTGTCTTAAAATACTCGAAACTAAATGCCAACG

>Cs1g05750

TTACGGATTCACGGATTTTTACGGATTTATAAAAGTAAATTCATATCCAATTCATTGACTGCGAATTTTAAAATTTTCAAATTCAATCCGATCCACCAATCAACGGATCAGATTTTTGCGGATCAAATAGATTAAGTGGATTAGATTGGATTCTGAACACCCCTAAAAAAAATAACACACTTAGGTAGTGCTTGTTCTTTTGTCTGAAATCTAAATGTGTACGGCTTTGATTGAAAGGTAAATGGATGTGAATATAATTATCTTAATGACATGCTTATTTTTTATTTTTTAATATCTTAATATAAGCAATAACTTGTTTGCTTTTATAAATAAAAAAGTTTAAACATTATTATTTAACATTTATGTCTTTAAAAGTCAAAATGAATAACGAGATTAAATTTTATAGTCCAAGAAATAAGTATATTTAATACGGATAATTTTAGGTTATAATGTTGGCTTCCCATTCAAACATTTCCCCATTTAGGCAAAGATTACAAACGTTCACTTTTGAGACGTTTAACAAGCATGCCCTACCTGATGAGTTATAATACTTAATGTATCACTTAATACTGCACATCACAATTATTTATATTTTCATAATATGCCTATAATATATACATACAACAAATATGTAATTAATATATTTTACTTATGAATGAAGATTGAAATATTACTTTCAATAAACTAAATTTTTTAAAGAAAATTTTTATTATTTGTAAGGGTAAATTTAGATAAATGATAATAGTCATAATAAGTGGAAAAAAAGAAAAAAATGAAGTTCAATTAATTATTATTATTATAAATTTAAATTTCAAAATTCGCAATAATGCGTTTTCTCTGACGTTACTTTGGAGGGAAAACGAAAACGAAAATCCACCCGTAAGTGGCGCGCACGCACCCCCCCTCTCGATCCCACAGCGCAAGTTAGCATAACCCAAAATTCAACAATATTAAATTAAAAATGATTCAAAAATTATAAAATTTAAATAAATAAATAAATT

>Cs1g23460

NNNNNNNNNNNNNNNNNNNNNNNNNNNNNNNNNNNNNNNNNNNNNNNNNNNNNNNNNNNNNNNNNNNNNNNNNNNNNNNNNNNNNNNNNNNNNNNNNNNNNNNNNNNNNNNNNNNNNNNNNNNNNNNNNNNNNNNNNNNNNNNNNNNNNNNNNNNNNNNNNNNNNNNNNNNNNNNNNNNNNNNNNNNNNNNNNNNNNNNNNNNNNNNNNNNNNNNNNNNNNNNNNNNNNNNNNNNNNNNNNNNNNNNNNNNNNNNNNNNNNNNNNNNNNNNNNNNNNNNNNNNNNNNNNNNNNNNNNNNNNNNNNNNNNNNNNNNNNNNNNNNNNNNNNNNNNNNNNNNNNNNNNNNNNNNNNNCCCCCCAACTTCGTGATGATTTGAAAATGTTAATTACTTCCAAGTTGTCAATGTTTCAAACATTGAGCTGGAGTTTGAACATGACGGAGAGCTGTTCCCACTCATTAATGATAGTGATTCCAAAGCGCGACTCATGAAGCTTTGTAAGTTATATGGACCATTAAGAATGACTATTAACTCAAATGCAAATGAGTGAAGGACCCATTCTTCAAAAGTTAGTGCAGATAAAAAGGATTAATTTAAGAAAAATATTAATTGATCTTTCCAGCAAAAGAGACAACTTTTTGGCTTCGAGATGGCCCAAATTCGGCCTACACGTTGTATTCGTTCCAAATGTACCTGAAATCGCTTCCATTATTGGTACTGGATAGGTCCATGCAAGAATTAGAATTTTTTAAAATAAATATATATATAAATAAAGAGAGAGAGAGAGAAAAGTTAAATACATAATGACGTAGCAAGTGCAGTCAAACAAAAGTGGGAAAAACTGATGTAGTAATAACTAGTAAGTGGGCTGATGAAAAGACTTCCATAGAAACAATGAGATTCATCTCAAAGAAATAGTCTAAAGTGTCCTGTTATTGATGTAAGTTATGGTTCAATTAAACTCCAGCTTTCACTGGGTTTTGAAATTGACGTTTTCTTAT

>Cs1g08660

TTAGTGGTTTAAAATACATCAACTAATTAATATAGGAAGGAAGGTTCCTTCATCTTTCCTCACCAATGCCACTAAGGCCTAATTTTAATGCCATAATCTCATATTTAATTTACAACCTTTTATTTAGTGTCTTTTGGCTTCTTCAAACTCTATAATTATTAAGTATTTAAGATTTGTTATGTTTGGATTTTTTTTATTATTTAACTAACATCCTCTTTAGAAATGTTCTTGTGTTTTTATTTTTTGACTTATTTAAATTTTTTGTATTGTGTGCTTCATGGAGTATTCTTTTCTTCATGAGTTAAACAATGATAAAGATAGTTGGATGATAAGTGTTAGACTTTGTAAGATGTGAGAGTCTGTTAATACCAAAAAAAATAGAGAGTTGATTAGTGTGAATATAATTTTTATTGATGAAAAGGTTGTGTACACTTAACTATACACATTAATATATATTTTTAAAGTTAACAACAAAATAATTAACTTTCATAAATAACATTAGTTTTGTAAGGCTGCGTCATCTTTAAAAAAAATTAATTCATATTTTTTATCAATTTTATCAAGTGTAAAGTAATAAAAAAAATAATACTAAATTTTTTTTTAAAGCCGCACGAAGTGCGGTTCTATTTACTAATTGCCAAATAAGGTCAAGGGTTTTATGTGGAGAGCAGCTAAGAACCTTTTGTCAACTGCAATGAACTTGTGGAAGAGAAAGCTATTGTAGAGCCCAATTTGCTAGCTTTGTAGGAGAGGAGTGGAGATCACATGCGATACTCTCATGGAATGTAAGCAGGCTTATAAACTACAGAGACATGCTCCTCTCACTATTACTAGAGCAAACGTGGGTGAGTTAAGTGTGATTCATGATTTGTCGAAGGATTTAAACAAGGCAGCATTTGAACCTGTGTTATTGGCTAGCTTGGAATGCAAGAAACGGATTTGTTTCGAGAACAATAAGATAGATCCTTTGATATCAGTGGCAAAGCCAGAAGCAGTGGTTG

>Cs1g24110

GAAGATAAATTATAATAATTTGACGTACATATTTAAAGAAAAATGCTTATAAATTAGAAGTATTTAAATTTAGGGAAAAATCAATTCTCATCCCTAGGGTTTCATACAATAGTTCATTTCATCCTTGCTTTTTAAATAATAACTCAAAGCATCATTTTATTAACTCACTATTAGTTAAATACCGTTTGTTAGTTATTCATTGAGGGTAAAAAATGATTTTCTATTAAAGAGAAAATTAAAATAATAAATAAAAGTTGACATGAAATACCGAAAAAAGATGAAAACTTCTATTTCAAACAATTATTTTGCACTACTCTCTATTTTGGCGGCATGAAAAACCCTATAAACCCGAGATTTAAGTATAAAAAAAAATCACAATTTTCATATTCGTATAAGTATACTCTTTAATTGCACTGTGAACCAACTTAAGGAGAGTGATAAGGACTTGAGGGAGATAATAATGGTCAAAGAAGAGTGGTGGCAAAGCTCAAAATAAAAATTAGTTAAAGATTAAAAAGATTTTCAGCACATGCAATCACATAAAAATTGGTTGTATTTTTGAGTTTTTCTTCCAATGATTTAAAAATATTGTGTTAGTTACAATTTGTGCTTATGGTGATTATTCAATGGCTATATTAAACTATTTCATCGTTGATTTCATTTAAGAAAAAATTAATAAATTTTACATATTTTGTAGTGTTATTTAATTGTATAAAAAAATATTTTAATATAATTTATACCACTTTAATAAAAAATTATATTTTACCTTCAATGAACTACTAATTAACAGCATTAAACTAATGGTGAATTAGCGAAATGGTATTTTAGGTCATAATTTAACAAATATGAATGGAATAGACTATTCCATGAAACTGGATGAAAGTGACTTTTTCCGTTAAATTTGACATGTACAAATACACAAGCCCTAACGACACTAACGAGCCATTTAAGGAAAGCGATATTTACTGGCATTTGCGGCGCATCCCAAGGTCATAAGAAAA

>Cs1g15080

ACCATGTCTATTTTTCAATTTTTTTTTTAATTTTTTACCCTTTAGCATTATCTTAGATTATCAATAAACTTGTGTTTTGTGTATTTTCTTAAAAACGACAGTCAATTTGGTCATGTGGAGTTGAAAATTAGGAGAGGGAGAACTAGTTTATATAAATAGTGAGAGGCGAAGTACAGATAATTAAAGATTATTATTTATGGTTATGGGGTTTATAATAAAAATAAAGAAACAAAACGAAAAGGCAGACGAATAGATATGAGGGGCATTTTTGGAATTTTGAGGATGTTATTAGAATTATTTTAATTTTTTGAATTTTTTTAATGGAATAACTTGTATAGCGGGGTTCTATAAGAAAATTTGTAGGGTGTGAAAAGCTTAACCCTTTTTTTTAACTCTCCCCAATTCTCCGGTTGAAGATGAGCGGAGCCCAGAATTTTCTACACGACGTCGTGTCGTGAATTTGAGCTCCCTTTGGTCCTGGATATGGACTCATTCTCAGTAGACCCACAGCTGTCCGTCGAACAAGATTCTCACATTTAAAAAAATTATTTTTTATAAAACATTTTTAAATATTAAAACAACAATCTCACGTTCTTTTCTGAATGAAAAAAATGTACTCATAAGCTATTGATGTAGGCGTAATACGACACTCTGATCTGAATCAATATCAATTACATTTCATACTTGCAAGGGTTAAGAAATTCACATGCATCTATGAGCATCCAAGCAGCAATTGTAAATACAAGAAAGAAAGGCTTCAAAAGGATCGTCTTCAATGAGAGTAGAAAATTTTTTACGATGAAATTATTGACCCAATCATTTTAAATAAATCTTTAAAATACAGAAATACACTGGGTGATTGGCCAAACTCAAGGGACTAAAAAGATAAATTAATTAACTAGCTATCAAAACTTATTGCATTTTTTGGTTTGGAGTACATGCTGTTTAGGGTGAGAAATATCGTGACTAGGAATGCTCGATCAGAATGAGCTCTCCTCGAT

>Cs1g03440

AAATAAATTTATATTTTCTCCGATCCCTTACCTTTTAAAAAAGATTAAATAAAAAATTAGTTGTGCTTTACTTTAGTCAACAACAAAGACTTAACAATGGGCTCATTATCATCTTACTAAATATCATACATCGTACTGAAGTTTAGTTTGCAAAGCAAGAACAAGAATGATGACCAATAATGACGTGGTCATAATTGTAGCAAATCAAACCTAGTCACAACAAATAGATAGAACCAGTGAAAAGCTCAGGCTTCAGAGAGCAAATCAGCAACATCATCAGATGAGAAGAGTCGAGGTCTCGTTGAAGATAGGTATAAAGTGCAATAGCAGAACAGAGGCCAAAATTGTAGATGCCAAGAATACCAAACAGAACAGAGGGCAAAACTATGGATGCAAAGTTTCTACCGCTATATATATATATATTTTGTTTGAAACACGAGGTAATCTTGAGTTGGCCTTAATTCTGGGAGGTACCTTTAAGTCCGTACCACAATTCAGACTAATTTCTGCTCGCACCGGATCGGCTTAATTCTGGGAGGTACCTTTAAGTCCGTACCACAATTCAAACTAATTTCCACTCGCACCGGATCGGCTTATAAATAGTAAAGTGCTGGTCAAATTGGTGACTCTATACGAGAGCGAGGTTTGAACCCCTGACTTCTCTTAAGGAGTGAAAGTGCCGAACCATTCACACCAGCCAACTTTAGTTCTACCGCGATATTTTAAATTTATGGTAATGTGAATTTATTGTATTGTACTGGGGCTTTAGGTTATAATATTTGTACAAGACAATTGCATTTTACACATAATCTAAAGTATCCCTACCAACGTGAGTATATCTTTAAGCAAGTAGACATGTAGAAAGAAGAAAGAAAGTCTAAATCCTGCACCTGCAGCATTAATGAGCTATCTATAAGCAGCACCAACGGAGGAAATATTTTAAACAAAAGAAAATAAAACCCCCGGCGCAGTGAGAAAATGCGAAAGCATCGAGTGATAAA

>Cs1g07060

TCAATTTGATTTATTCAGATTTGGCTGCATTAAGTTTATAAGTTAAAGAAATACAATTTTAGAACCAATCATGTGCAACTATTAACTTAATGAGAATAATATTTTTATTCTAGATAACGCCGCCTAACAAACGTGCCCTAAATACTAAACAAGACCTCCACCTAATAATAATGGTATTTGCCCCACACTTTTGATATTTACCAAGTAGGACTACTTTAGTTTCTCTATATCATTTTTTTTTTGGGGTTTTGTTGGTTTATTTATTTATTTATTATTTATTATTTTTTGCTTTCAGTACGATCCAATGCAATTCATCCTCCAAAGAAACATCTAAAAAGAGTTAAACTCCAAATATGAATTAAAGATAAAAACAGTGAGCAGTAATTTTAATGCATCAATTTAATACAGTACTTAAGTAAGGTCTCTTCAAACTGGACATTGATTATCAGGGGTAATTCTTTGCCCTTTAAACATAATTTCTGTGTTGGAACATGATTTTGAACATTATGACAGCTTTAAATTTTATTAATTACCCAGAAGAACAAATAATCATAAATAGAGCTTTCTCAAGTCCCAACTCTTCAATCAAGGTAGCTTATCTTTTGTTCTTTCGTATCTAACCATGGGTATGACAATAATATTCCAAGCTTTAGTTTCTCTTCTGGTCCTTGCTTTGCCTCAACTACTACTACAACTCCCCCCAATTGCGGCAAAAAGTTTTAGCCTGGAGCTCATCCATAGAGACTCCAAAGAATCCCTTTTCTATCGTGGAAACCTCACTTTCCGCAAAAGAATAGAAAGCTACGCCTGAATTTCTGAATCTCGGATGGCATATCTGAGGCCATTTCTGAGATATAACTACACCATATCCACTAGTCCTGAGATGATGCACCCGAAAGTTTTCCATCATTCAAACTCATACATGGTACGATTATTCATTGGCACTCCTAGCAAGGGAGTCCTCCTGATTCTTGACACAGGTTCTGCTCTAATGTGGGACA

>Cs1g19660

TTTATTTATAGGAATGGAGGGGCGTTACAAGCAATACAAATGATCAAGAAGGCTGCATATGATTGTGATTAGACGACAATCTATGCTTTAATCTATTTAATAATACATAAATGTATAGTACAATTAGTGATCGACGGATACATTGCTCCAAGCCCGTACAACAAATTGTACTTAATAATTGATCCCCTATTATTAGAGACGAATGCGTGAATGTATGCTAAACAATTTCGCCGAATAAAGGCAAATTTGCCAGACAACTTTAGATTTGTTTTCGATACTCGAATCAATTCCAGGCTGAAAGTAACTGAACCAGTTTCGGTTCTTGCAATCTGTTCGTTTGTTTTTTCGCCCCATGAGGAAAAAGGGCAGGAAAGTTGATACGTTTGTTTTTCTAAGAACTTGTTGGGCTTCTACAAGAACCTTTTTCTTTTTCGAGAAATTAATTTTCAGAAAATCAGTACAAATTTTTACTGAGTTCATAGTATTTTTGTCAAAACAAAAATTCATTTTCAAAATTTTTTTCTCTTTAATAATAATGATAATAATAATAACTGTCTTTTGTTTATACTTTAAATATAAATTATATTTTCGTTAATTAAAAGAGATTTCTTAACTCTAAAGATGAATAAAAATAAATAAAATTACTTAATTAATAATCACTTTGCCAACGATGTTTAAAAGATTTCGGCAAATGTGTATGCATAAAGAAAACTCTTATTGCTTTTTGTTTTAGGATGATGTAAGATACTTCGTGTTCAAATTGTAATATCAAATGCTTCATGTGAAATAAAACTAAATTATAACTACAAACTCTAACATAAATGTTGAAGAAATTTTAGAATATCTTTAATGTATTTTAATTAATTAATACATACATAACATGTATAATTGAATTTAATATAATCATTACTTATATGATAATTTTTTAAAATAAACACATACATATTACAATAACATTTGCGTATTGTATGATTGGTATGATATTCTAAAATTGGTAAGGC

>Cs1g14690

CAATAGTTGGGTGTGATCTTGTTGAAGTAGAAAGTATTTTCAAGCCACGAAAAATTCCAGTCTTCTTTATTCTTATTGACCACTTTCGGTAATTAACTCAAATACTTCTAATGAAATCTGGACTAATTACGAAACAGAATCTTACAAAACTAAAGAAAAATATTCGATAGAATTCCCCCCATCATTTTCTTGATTTGTTTATTTAATACCTCGCGTTTTAGTTTCTAGGGGCGCCACCCTATAATCTCACGAACATTTTGCTCTTCAATTTGACATATAGATAGACATATTAGGAACAAGTCCAATTAGAAAAAAGTGGCCACCTCATATATTAAGAATCATGAACTTGGTATACATTATTGGAAAATCATGATTCAGAAAAATGTATAATGTCAATGTCCAAATCATATGAGAAAGGACAAAGAACAACCAACACTAGAGAGAGAAATATTGTAGAATATGCCAAGCAAGTTGTTAATTAGATACTAATAATTTATAAATTTCTGTATCTATGACGTTACAATCGTTATTAAGAATACTAATAGCGTAGCTATCAAGAAAATTCCGCAATATTACTTGTCCAATTAGGATCCTATCAAGTTTGACTTAATGAAATTATAGAATATCTGTAGTGTTATCAAGACCATACCGCAATAGTACTAGTCAAATTACGATCGTACCTTCTAATTTGAACTCACCCAACTTTTTAGAAAACTTTGATTTATTTTTCGGGTTCTTTCTGGACAGATTTAACTGCTAATCTTTCATACACGGATTTATAAAATAAAATAAGCGCAGTAAGAGCATCAGCAAACTACGTTAGCAAATAATAGCCACCGTACGAGAATTACGCCGCGTTTCATTCTCATCAACAATTCCCTTACGTAATATTCAATTATTCTACTCGTTCACATTCTTAATAGTTATTTAAAAGGAATAAAATAAAAACAAATCCTCTCTCTCCCCTTCCTTTATATGCTCTCTCTTTCTTTCTCTGCTTG

>Cs1g13670

AGTATAACTTATGATATTGTGCAATAGTAATTTAGTTTTATAACTTACATAATCAATATTTTAAAATGAAATCCAATAATTTACAAAAACTGTATTTTATACGAAATAAAAAATTCTTGAAGTTAAAATTATTTATTTGCAGTTATTTTGGCACTTTATAATGGTAAAACAATATAATTAAACTAATATTAAATCAACCTTAATGGTAAAAGATGTTTAGTCACAGAAAATAAATAAAGAAAGAAGGATATAACTGATTAATTGTCTAAACTTTTTCTTTGTTTGAATTTTAAATTAATTTTATTAATTATTTTTAGTTTAGAGTTGGCAAAAATTATGGGGGAGGGGGATATCATCCCTGAGAAATTATTGGGCGACCACCTAAGTATTGGACAATATTGAAAAAAAAAAAGAAAAAAAAACACTAAAATTTTCATAAACATGGCACAAAAATACTTAAAAATTTAGCAAGTGTATCCCCAAATTACTTTTATGTTAACCTTCATTATTGTATTCCGACGTCACAAGGGTGAATCTGATCACCATCCGCAGTTCTAAATTTAGAGTGTTTAAACCTAAAAATTACGTGCATGCAAAGCAAAAGAGTCAAATTCGGATTTACCCACCCTGCTAAATCCTGCCTCTAAAAGGACAGTAACATTAGCGTCATTTTTAGGAATTCAATACGTTGAATCCATTATCTACGATAATGTAGAATAATAATTTTTCATTGTTATGTTGTTTTGTTGCTGTTGTTGTATAACGGTACGCGTTGACTTTGACAACAATTTTTGGACGGCCACCTATCGCTAGTTATAAAACAAAATGGCTCTTAAAATATGGTCGTCATACTCAAAATTTGGAAAGACGGTGCAAACTTCAGACAAAATTTTCAATCGCTCAAAAAGTTTTGAGTGAATGTCTTATGCACTAATATCGAAATTAGTTGTTTGCATAACCAGTTTAGAATAATTGTGGGTTTTGTTTGGCTTCAGTTGTAA

>Cs1g11940

TATGTTTTTATTTATTGTAGAGACTTAAAATAATATGAATGAATTGGAAACCAATCTTCACAAAGAAAGAAAAGTTAGCCTTCACATGGAAGTATGGAAGGAAAGACTAACCTTTATAAAGAAGGAAAAAGCAACCTTCAGAAGGAAAGAAAGACAAAAAGATGAAAATGGGGAAAAAAGTTATTAATAGTAAATAACACTAATTAATAATTAGTAATAGTGACACTTAATATAATAAAATTACATTAAATTAAAATAAAATTGAATAGAAACAAATACAATATCAATTATCACAATGCAATTAGTAAGTGTCTATTTGCTATGACTTGTCTAATAGTTTTAAGTATTTATTTAATCCCATAAGCTCTTAATCAAATTTTTTGTTATTGTTTGATTATTTGTTTAGTAGAATTTTTTTAATATTAAATAAGATATTTGGTGTCCACTATCAAAAGCTTAAATTTAGGACTTTTGAGAGTAAAAGTTGAAATTTTTTTTGTAAAATACTAAAATGTTTAAAATATTATTTACTTATTTGACAACTTTATTTCATTATTTTTCATAACCTAACTTTAAAAAACTTACATATTAAAATCATTTTTATAACTTTTAATAAAATATTGACAACCAAAAAATAAATTTTTTTGCGTAAAATATCTATTTGTAAAAACTCTGCTAATATAAACCCATTTAAATAAACTGAATTTAAAAAGCTACAACCAAAATACAACTAGAATTGTTTTTTTTAATATTTAATTGTCGGGCTGAAGGATCGGACTCAGACTTTCTTGATTAACCCCTAGACCGAGCCCGATCCGACTCGAAGCCTAAAAGTGGAGCCGAAGCCTGACTGGGCCGGGCGCTCACTTTTCTGCCATCCCTAGCGGCAACTCAACTCAAGCCTTGGCTGGATGGTCGTAGTTTTTGCGCGTTTCAGTTGACAGGCGTTGATACAGGCTACACGGGGTATGCGCAGAGTCTTAACCTACACCCACAACATT

>Cs1g24670

AGTCGCCTTTCCTGGTGATCGGTAGCATACATATATTCGCAATTGTCTTCTGTGAGCAGAGATAGAAAAAGCTCAAGACCGTGCTGAATGATGTGAATTTCCAATGAATAAATGGCATATGGATGCACTTGTGTATTTCGAGGCTCTTGTGATCAACTCTCTGTTTCATCAGTAGCCATGTCAAGAAGAAAGACCAGAGAGCCTAAGGAGGAAAATGTTTCTCTTGGACCTGCCGTTAGAGATGGAGAGCATGTATTGGGCGTTGCCCACATTTTTGCATCTTTTAATGACACTTTCATTCACGTGACTGACTTGTCTGCTTGTCTGGAAGAGAAACACTTGAGCGCATTACTGGTGGTATGAAGGTCAAAGCTGACAGGGATGAATCCTCCCCTTATGCAGCTATGCTTGCAGCACAAGATGTTTCTCAGCGATGCAAGGAGCGTGGCATTACTGCTCTTCATATTAAGCTCCGGGCTACTGGGGGTAACAAAACTAAAACTCCCGGTCCAGGTGCTCAGTCAGCACTTAGAGCTCTTGCTCGTTCTGGAATGAAGATTGGCCGCATAGAGGATGTGACTCCAATTCCCACCGACAGTACCCGCAGAAAGGGTGGTAGAAGGGGTAGAAGGTTGTAATTCTTTTCCTCACGCCCATCCATAACGTTCAAGACGATGCCGTAGTTACAGAGAGTTTCACTCTCTTCTGTTCGATGCAAGACTTATATTCCAATTTTGTTGTTTGGAATGTCTGTAGTCTCGGATTCCTTTGTGTTAGCCTTTCCGATCAATTAAGTTTATACAGTGGCAAGACTAATCATTTCTCCAGGAGTTTCTGAATTTTATTGTTTATCTACAAGTATTGTGTTTTAAGTGGTAAAGTATGATGGTAGTGGTTATCATTAGATGTTGAAAAAAAAATGGCATATGGATGCACGTTGCAGAACAAGGGACAGGTCCTCTTGTTGTTCTACTTCATGGGTTCCCAGAATTATGGTATTC

>Cs1g23330

AATCCACTTTGATCCTTAATATATATTTAACTAGATGTAGCTCTAATATTGAAGGGTGTTATTATTATTCTCTAAATATAGAGACATATTTATTTAGTAATGGCAATAAAGAGGGAAGGGGAGATCACTACGTCACTTTATTGGTCTTTAAAATGTTAATAGATGAATTTTTTTTATCTCTCTACCCCAAATAACTTACTATTTATGTTCAAAACTTAATTTAATTAAACTATAATAAGACCCCGTTTAATGAATAATGATTTGTTTAACTAAAAAAAAAAACTAAAATACACTATGTTAATAACTAATTAGAAACACCAAAATATCAAAATAAACTAAAGTTATTGATAATTTTTTGTCCAACTAAGATCAATGTCATTGACATATCTTACTTACGAGATCATCCAAAATTTAAAGCATCAAACATGGTTATTAACACAAAATAACAATGTTTTTAGCACCAATTCATTACATAAACCACCATATTTAACTATTCAACAAAATCATTCAAAGTATCTATGTCAACATTATATCATGAATTAAATGTCCAAAAGTATTTACTGTAGACAACTTTAGAATCAAAAGCATAACTATACTTGTAAGATTCTTCATAAATTTACATGAATAAACTTGTATCTATCAATTATGTTTTTATTAAAAATTTTAAAAATAAATATATTAAATATCAAATGAATAAATTATTTTATGTATAATGTTTATTAATAATATTACATAAGCTTATAAAAATACAGTGTATAGGAGAATAGGGAGGCGATCGGACTTTCTCTAAATAAGAAATGAGTATGAAAATAATAAATATATCCATCTCCTAATTAAATTTATGTTTCATTCATCATCTTGATCACAATCCTAAGTCCTACAGCGAAATAGACGTTTTTGATATTTCTGCCATTTCTTTTAATTTAAATATTAAATAAATAAAAAAGGGAAAAGGTCAGGGATCTATATGATAGAACCCCAAAAGCAAACTAACGTGTGCA

>Cs1g05350

ACTTAAATGAGCTTATGTATTACTCATTTTGTTATCATGCATGCATATTGTTATGTGATATGGATATGCCATATATTGTGTACACATTTGGTTGCGTATGGCATTATGTATAGAATGAGGATACGATGGTCCGAGACATTTTGCTTAGTCTCAATATGGACATTAAAGACTGAAGCATTATGACTTAGCTTCAGTGTGCTTTCTGGAGACATTTTGATTCAGTCTCATTATGATCATAAGAAAGCACGAATTCCATGAGATATCTTGGTATTAGTCTCATTATGATGAAAATGGAAACTCGCTTCTCGAGACATTTTGCTAGACTCTCATTATGGTTATAACGAAGCGATGGTCTAGGAGACATATTATTTTCGTCTCATTTTGCTAGACATGTGGACTTTATGGAAATGCCTTGGCCGTACAGCAGGTAAAATTTGAAATTACCTAAAATATGTGTTTACTTGGAAATGCACGATTAAGGGCATAAAGTTTGAGCACATTTTAGACTTATTTATGTTTTGGGTGATTGCTTGTATTTGACCTTTTGTTATAGTCACATATTGATTAATTCGGTTGTTTTCAAAATATTATTTTATTTTAAATTTATCATTAGAGTGATTTCTGTCGTCCAAGTGGGAGATTGTTAGATTTATTTCTCACATTATGTGGACTGACATAAATCATTCTAATTATTTTCTATATTTTACCGAATTAATACGTGACTATGACAGACTCATTTATTAAGTGATCAAATATGGTGTTAATGAGTCTAATTTTGACATACCCTGTACAGTAGTATATTATGGACAAGTATATCTTTAATTGTGTTATTTTAGCCCATAATGAGATGGGCAATTTTGTACTAACACATGAGGGGTCCCTGCGCTCAACCGCTGTATTTAAGTAACTTCAGCCCATTTTGGAAGTTAACTGTTGAAAAATCAGTTGCATGTGGGTGCAGTGCACCTCTCTCTCTCCCTCTCCCTCTCCCTCTCGTCTCC

>Cs1g13960

ATTCGGTGTTTGTGACTGTACAGATCACGTGTATACTTGTGGGCTCCTTTTATGATCAATTAGATTTCAATTTCTCTTGCTTTTCAGATTTTTTTTTTTCAAAATAAAATAATTGTTTCTTTTTTCTTCTATAAAAGGATTATCGATTAATTATCTGCCAGCTTAGACTACATTTTTTCAATTTCAATAAACAAACCTTGCATGATATGATAACATGGTTTAATTTTTTGAGTAGCGTAATAGCTACAGATTTTCATATAAATTATTTATGTATAAATTAGTGTGATATTATATAATTAGTTGAATGAAAATATAAATTAATAACAATAAATATATGCACTAAGTTATATTTTCATTCAACCACTCATATGATACCACAAAGAGTTTGTGGTTATATCATTATTGTAATATTTTTAACATAAAATTATGAAGATTATAAAAAATAATACTTTAAAAATGACAGATTTATTTATAGTAACTTACATTTTCAATTCTCAATATTTTTTAGTTTCGTAATTGAATTTTTACCCAAGTTAAATCTGGATAATTTTTTGTGATTTTCATTTTGAAGAAAAGAAAAGAAAAAATTCTTATGATTACAAGCAGAAGAAATTAGTGAACATAACGACAGACAGCGGAGAATGAAATCAAATCAAAACCTTTTATTTCTGCTGGGATCTTGGGGTCCACAATAATCAATTATTGTCCACACGCAATAATAACCCATCAACACAATAAGAAAATGTATATAACAGAATATTAGATTTTCCATTAAATTAAATTTTGGGGACAAAAAAAATCTCAAAGTCATGAAAACGACCGGCTCAGACCGACCAGCCAATGGGGCCCCGAACTTTAGGGCACTCAAAATCACACGCTCGCGTACGGTTACCTACATGGACAAATCATAACCGCTCTCTCGAGGACACGTGTTGAATCGGGAGTGGTTACGTAATTTTGCCGGCCACATACTCATTTTCTTCCCCACCCTTTATATATAC

>Cs1g08670

AAGCTTTTTGTCAGAATCCGATTTTGATGGAACAGGGAAAAAAGAGGAAACAGCTATAGTAGACTTTTGGAAATGGTTCTCTATTCTAGTCAGCTATTTTCCTATAGTATTTAAGTTAGTATTACAGAAATTATTCTGTTGGATAATACTACTTAAATTCGCATCAGTATCATTTGGCTTTGGGATTTTATAAGGTGAAGCTCTAATTTGGTCAGGAGGTTCACCGTGATCAACAATTAAACTCCGAAGAGGTGGATGCTCAGATTCTATGGTTCTATCACTTAGGGCAGTAAAGCCATACCTGTAACCATTCAGTCAAGACCATAGATAGACCATAAGTTCTTTCTTCATGGATCCTCTGCTCTGTAGAGTATCGTCCTTTTCTAGCTATTCTTCTGGGAAGACTAATGATTCAAAGCATGTTGTGAGTTCAGAAGAATTCGTTATTGAGAACTTTGATAAAGCAATTGATTGTTGGGAACTTCCAAAGATTTCTAAAGAAAAGATTTATAAAACAAAAAACTTTGATTTTCTAAAAAATGATTATGTAATAAAGACTGAAGAACGTGAAATAATTCTTTCAAAGCCATTTGAAACAATTCAATTGTTTTAAGAGCATTCATTAAAGAAATTAAAAGAAAAGAATTTTAATTATGTCCATATAAGATTAATCCAAGTTGGCATAAAACCATTAACCAAAGAAGGCTTCGATACTTCTATCCTCGCTGTCCTTAGAAATGGCTGATTTATCTCTTTTGATGATTTTTTACTAAGTAGTATTGAATCGAGTCTCTGTAAAGGCCCTATATCTTTTGATTGTTATCCAAACATAACAATTTCTCTTAAAGACAAAAATATTTTAAAGAGCATGATCTTACAAATCAAAACCCACAATTATCATATGATTGAGGGATCTGTCCCAGTTGCATTAATTTTTAAGATTTCTTATAAAGCCATGATTTCTGCATTTAGCACACAACATAAATTCCAGTCAAAAAGAG

>Cs1g20530

CTGTGGCTTCTTATCTCTTCATGTCTCCCATTATCTTTTTTTAATTAAGATCCAGTGATCCACTATCTTTGACTTTTAGATAATGGTAACCCATTATAGGATAACCTAAAGCCTAGGTTAAAATGGCCTGCCCCCATTATCATCTTCTTCGTTTTGTAAATTTTAACCTTATAGTTCCATTTCACAATAGATTTATAATTTTTTTTTTAAAAAAAAAATTTACCTTTTGGGATAGAACATTTATTATGTGTTTTCTAATAAGACATTTTGTCCGAAAAAGCATTTGAAGTCCGTGTTTAAATAAAATAAAATAAAATAAAGGAATGAAAGAGGGAACATTTTCAAATATTTTGACAAAATAAATACTTGATTAATGGGGAAGTGTAATTTACATGATGTTCATTGAGATGTTGATGATGAGAAAGTTCCCGTCACGCAGTCAGAATCATCCCATTTCAATATTCTTTTGTTTTTCTGAAATTATTATTATTTTTTTTTAAAAAAAAAAAGTCTTCCAACCTTGTATGACCAAATATAAATACACTACACCTATGACCTACCATCATGTTTGTCCTTATATGGACATTAGTAGGACATCAACTAAGACATGCTCTTATTAACGATTTATTTTTATAATTACCCACCATAACTAAATATATATATATATATATATAAATACTATAAATAAACTAAACAGGGAAAACACGACACGAAATCCATACCCACCCAATCGGCAATCGGCAATCGGCAATCGGCAATTGGGGAATTTCCTTGATAACAGAAATTATCGATATTTCAGCGCCAAAATTGGTTCATTTCACATCATAATAGACAACAAAATAAAATAAAATGATTAATTGATTTAATCAATAATTTGGTTCTGCTGCCTACTTGAAAAATGAAATGGATGTCGTGGGAAATTGGCAGCCCGCCAGTCCGCGGTCCCCACTCCCCATCGCGCTTCTCTTACTATAAAAATACGAGTCTAGTTATTACACAAC

>Cs1g03260

TATTAAGCATCAGTGTTTGGGTTATATATCTCTTTCTTGAGTAAGTTATGTCTAAATTTTATAATATTAAGCATCAATAGTTGGGTTATATATCTCTCTCTTGAGTAAGTTATGTCTAGACTTTATAATTTTTATTGACCATTTTCGTATTTAAAACCTCTTTATGGCATTCTTCGATCATTTCTTGGGTTAAGTTAATATTGACAATCTCTCTAAAGGAAATTCAAGTCTATCTTCTTGTAGTGCTATTTATAGAGGTCCTCGTGGAACTTTTTTGGGGTGCTTTGCTATGCTAATTGAGAATCAAACTTCCATTTATTTAGAACTTTATGCTACCATTTGTGCTATTCAATTTGCCTGCTATTCATTGTGGTTAGAATGTGATTTGACAACTGCTTATTCTTACCTAAAAATTTACATTCTTTCTCCTCCTTGGCAACTCTGCATTAAATTACTCAATTGCCTAATTACGATAAAGTCTATGAGATTTCATTATTCTCATATCTACCGAGAAGGTAACGGTGTTGCAGACACTTAGCTAATATTAGTTTGAGTTTTTCCCACTTGACATGGTGAGATTTTCCTCCATCAAAAACTAAAAAGTCTCTAATTGATAACTCTTGGGGAATGCCTAAGTAACAATTTTCTTAATCCCATTATTGTTAGTCCATTAGGATTTGATTTGGTTTAATCATTTTTAACTGAACTCCTGAATCTTTTTTTCTTCATTTAATATATTATACCTCGCTCAAGGTTTATTTTAAAAGATAAATAAATAAAGGAGAAAAAAATTACATATCCCACGCATACGAAACACGTGATTAAGTTGAGATAAATGAAATTTCAGGGAAGCACCGAGAGAAATAATAAAATAAAACCCTAATGGCATGTGCACTATAAATAACAAGAACCAATTCTCCAATCTCCACAACAATTTTGGTGATCAATAGAAATTAAACAAGAAGCGCTTCTAAAATCTTCCCAGCTTGAACTGAAAAAAA

>Cs1g12990

GGGAAGCCACTTTATACAGATCGGAGAATAAGATTTTTCTCTCTATTGTTGAGAGAAAAATTTTCTCGTGCTAGTTGCTTTGGTAGTGATAAAGGCGCCCACACGTCAAGTGCAGATCGAACCTGAGTCATAATCTGGTAGATTATTGGTGACAGTTCGTGATCTAACAAGCGTGGTGGTGACGGATCGTGATCTAACAGGAGTGGTGGTGGCGGATCGTGATCTAGGAGCCTGAATCACTTCAGCGGAAAAAGCCAACTCGGATTTTCAAGGTACGATTTCTAGAATACGAATTCTTATATATTATATGAGAGCGATCTTCAAAAGATTTTATAAAAATTTAAAAACCTGATTTTTCCCCAACATGTATGTCGTCGCCCATGAGCAATGGGTGCCATCGCCTGAGCGACGACATCAATCATTCGAGTGATGGCACCTATCGCCCATAATCTATGCCACTAGAGTCTGGGCAAGACTTGTGACTTTGCACTGCTAAGGGTTTAAAGACTATAAATTGAGCTCAATAGCATTAAGGTTTAATTAAAAGCGTTTGATACTTTATAATTCATTTTGAGGAATGTACGTTTAGGTAAGAATGCACATAAAGATTAAGATATTCGACAAATGTCAAGCACGTGCGGGTCTACCGCACTAAATAGTGTAACTTACAATAAGGGTTAGCCAAGGGCGTTTCTATCTAAAATGGCGTATTTTTTTGCTATAAAAATTAAAAGAAGTCAAAAACTAGCTAAAATTATGGATAGCTCCTAAATTTTGGCTATTTTCATGATATTTTCAACAAAATTTCTCCACTAATTACTGAAAAAAAAAATTTCCTAATTTAAGAGAGAAAACTGATGTTTGTATGTAACGGACACTTGTTAGTTACTATTTAATCAATCAATCAATCAAGATGGCCACATGAACTAACTTGCATATTTTACTGTTTCAACTTTCAATTCCCTCCATCACAACATTTTACAGTTTCAAGCCGTTTTTTA

>Cs1g15270

GAGAAAGTAGAAAGAAAAGAAAATGCCATTGATGAACAACAAGTTCTCACTTTTAAGCGTCTGGTGTCGAGGAGCACGTCGAACATGATGATATCGCATTCACTTCTCATGGTGAATTGGGAAATCCAAGTCGAATGAAGGGGGACGAGTCAAGTGCTCTGACGTCTGGTTTTCGACGGTTGATCTTGAGACAGGCAGGTTGAACATGACGTTATAGCGTGGCTGATCGGGAAACCTGTTGACGATGTTCCATTTCAAAATTAGTAATTTTAAATAAGAAAATCAGTTAAATTCTGTTATGCTGACGTGGCTTGACCCTTTAGCAGGACAGTTAAGTTAGTTTAGATTTCCGATTCCAGAAACGGCGTTTTGGGGCGGATTTTATGAGTCATTTCTAATTAAATCGAGAAAGAAAGTGCTTTGAGAGTCTCTGTAAAATTAATCTTCGATCCGTAATCTTTCTTTTTTTTCTTTTTTTTTTTGAATAACAAAGCTTTCATTCCTCTTCCTTTGAGTGAATGTAGGCTGCGAAGTGTGAAGCTTCCAAATCATGGCGTGCGTGATGGTGCATTCCGTAATGCCATAAGAGCTGGGCTGCAACATTGTTGGGCTAATAATTTTTCTATGGGTTTTTTCTAAGGGAATGTTCGGCTTTTATTATTTTGGGCTTACATTAAAAAGAAATCCTAATTTTTTACTTTGTTTACATCGGGTTGCAAATTACAATATTAATAAAATATTAATAGCTTCTTTTTCTATAAATTCTTTATTTTCTAGAAACTTTTGCTGTAAAAAATTATTGGATATGATATTTATTTCTCATTTAATTTTTTTTTTTTTTAAAAATAAATTTAATAACAAGCCTGAAATATATAAAATTTCAGATTTAAGGCCTTTAATAAACAAACCTCCTACTGTAGTCATTAAATTCTGTGCAAACAAAAATAATAACTGTTGGAGGTAACTTTGTGCTACACATAACGTTAAATAACGAACTTTCG

>Cs1g12610

GAGAGCAAGAAAAAAGAAGAAGAAGAAGAGAAGCCACCGCGGCAAATTCGGGAGCAATAATTTGGAGAAGTCAATGCAGAGATTGGAGAGAGACAGTTTCTTGAGTTTTTCTTTCTCACATCTCTTTTGTCTTGCTTGAATGTTCCTAATTAAATTCATGGATTCTCTTTATATTCCCATGAACTAATTTATTTTACTAGGGCTACGATGTAGCCTAACTATGAAGATTTAATTCCATAAATCTGTGTTATTTTTAATATATTATTCCATGATTGAGTGTTCATTATTGTGTTTAATGCTTTTAAATATCTGGCCAATATTTAAATGATTTGAGGATACATAGTGAGACCGAGAGGAGATTTATGTATTTTTGCTCTGTGTAATGAATGCCATGAGTTGAACGAAAGACAGAGATGTGCCAACATGATTCATGCAATCTTTCTAAGATTTTCCATATAGCTTAATGAATCTTTGCATATTTAAATTCACATAGAGATATAGTGGGTTAATATGTGGAAATATTTTTTATATTACTCGAGAGAGGATATTGAATAGATTAGAAAAATTTACTGTTAACATGGGTAATAAACTTTAATAGCATAGATGGAGGATTAAATTGGATTGGTTGTGGTGAAATCGGATGTCCTAGTGTTTTAATCTCTTGGTATTTTTTATTATTGCCTGCATCTTTATTTAATTTTTAGTTATTTGATTTAATTTAATTTTAATCTAAATTCGCTTATTCGATTGTTCAAATAAATTAGGGTTAGAATCATTTCGGTAGCTAATAAAATATACAATCTCTGTGGGACGATATTCTACTCTTCATTATATTACTTGTGCTGACTAGTACACTTGCTAATTTATGCATCAACGAGAGATTGAATGTGTTTTGTTTCATCTGTGGGCTTCTTGGTCATACTAAAAAACAGTGCTCGAAGTTATATAAATGTGCAGGCGAGGTGGTGAAACCTTATGGTCAATGGATGAGGGCATCGAAC

>Cs1g16710

AAATTAAAACCCTTTTGAATATGTCACTAAAACGATATGATTCTTCCTACCTCCTACGCCTTTCTTGAGAAAATTAAGGAAAAAATAATATAAAGTCAAACACAGACTTAAATATTATATGCAAACTTTCTATCAATCAGCTTTTAAATTCAACCATACAGTTGTATATAATTACCAAATGAAATTTATATGCCGCTATAAAAATTACTTTTTCTGCCTTGCAGCTTTCACATTTTGAATCAGCTTGCAGTTCTTCGGATTCCAGAACTTTCAGCCGAAATTTTTAAATTACTTTTCTTATCTGTGTATATACTGTTTTTTCCTTTTTAGCCATATATTGACAAATGAAAATCTTCCAGAAAATCCGTCTATATCTGGCAACTTCATGTGATTGGACAAATTTTTGCATGTGTCAATAATAAACTATAGTTTTGATAATTGGCCAGCACATTCCAGGGCCCTTCATCTCTTGGTCAGACTTAATTTTAACTCCTTGTTTAAATATGAATAAAATACTTAGGTCCTGTAATTTTAACACCTTATTTAAATCTAAATAAAAAAAATACCTACATTCTGTTTGGTATTGAGATTAGTTAATTGTAACTTAAAATTTTTTGGTCAATACGAATTGTTGTGACTTGAAAATTAAGTTGATTTAATCCACCACTTGTATGCTGAAAAAAAATTTATAATATCTTTATTATTTTTGTTAAAATTATTTTTTGAAAGCCATATTTATTAAACACCATTAACTTTTATTTTATAATTATAACTTCTTTTTTTCTCAACTAATTTATCTTGAAAGTTATAGTATATCAATATCAAATAATATCTTAATATTGTCGTACAAAAGCTTAATTAGTTATATATGTAGTTCAGTCAAATTAAAAAAGGAAAGAGAGACAGAGTTAGCACTTAATATGGGGCCATTTGCGCATTGCACGTTGTGTTGTGTGTAATGTGAACATATTGCCTGAAGAAATTAATTGAACCAGCAGTGA

>Cs1g05500

TACTAATGTAGAAAGCGCTTTTGTTGTTTAAGGAGGTAGAAGGGAAAAGTGCTTTTGCGATAAGGGAGCTTAACTATTTTTACTGGCTGAAATAGCAGTCAGCTACTCAGCTTTGCTTGTGATGGGGGGGATTTCCTGTTTTTTACAACATTTTAATTAAGAGGAATATTTGTTTCTTGCCTCGTGGTAAGTGGAGATGGGACAGTTAATCAATTTTTGCTTCTAGAAATTTATCAATATCCATCATCAACCAACCCAATTGTTTTCATTAACCTAATCTATCAACTACTGTTATAATTAATTACGTGCGAGAAAATGAGATGAAAAAGGGAATAAAAGGTAGAGACAATCAGTTTTTTTTTTTTTTTTTGACATTTCATGATAAATGTGTAATCTAATGCCCCCATCAAAAGTGAAGTTATAGTTCACCAGCAAGTGAGTTGAGAAAATAAAATAAGTAATGAAAGTCAAAACTTTAATAACTCAATTAAGAAAATAATTAATGGGTAATTATAAATTTATGACTTATCTTTAGTAAATATTATCAGTTTTGAGTACAATGAAAACCCAATAATTAATAACTTTTTTTTAATGATGAAGTTGTGGCTTTTGGCTTGTGGGCATAGAAAATTATCAAGCTTACCGTAAAAAAAGGAACTTTTTTTTTTTAAAATTTGAGCTAATCAATTCTTTTTAATTGGATATGGAAACACAACATACTTTTTTAGCTATCATTATGATTCACTTTGGATGATTAGTAAGATACTTCAATCAATGGATCTATAGTCTGACCTATAAATCCCTATAAGTAGGAATGATTTTATTTATTTATTTTTTTAAAATAAAGTATGAAAATGACAACAAGAAAATTGAAACTTGAAAAATAAAATAAAGGTTGTTGGTAAAGAATTACTAAAATGAAACTATCAAATTGAGAATTGAAATAGAAGTAACTCTGTCTATATTGAGTCTTGCTCTCTGCCTCTTTACTGTCTTGCCTC

>Cs1g08630

GTAAGACCCAACTGTATCCTAGTGTTGTGCATTCTCTGCTATATAGTCAAGATAATCTAATGCATCTTCAGGACTTTTATCCCTAAACTCACCATCACACATCATTTCAACGACTTGTCTGCCTTGGGATGTTAATCCCTCGTAGAAATAAGACACTAATCTCTATGTTTCAAAACCGTGGTATGGGCAAATGTTAAGTAGTTCTTTAAACCTATCCCAACATTTGTAGAGGGTTTCTCCTAGTTTTTTAGTGTGATTTGTCTTTTGAAAGAATTTATTCTAAGAGGTGGGAAGAATTATTTTAAAAATTGTGCTTGCATTTCATCCAAAGTTCTAATAGATCCTGACCTAAGATTTTGTAGCCAAGTTTTAACTTTATCCTTTAGTGAGAAAGGAAAAACTTAAGCCTAATTATATTCATGCTACAATTTTGATCAGCACACATGTTACAGACTTTCTCAAATTCCCTTAAATGCAAATATGAATTTTCAGACTCAAAACTATGAAAAGTAGGAAGAAGTTGAATGATACTAGGTTTGAAATTAAAACGTGATGCATCAGGAGGAAACACTATGCATGATGGTGACCCTAATCTAGTAGGATGCATGAAACTATATAGGGCTAGGGAGAGGGCAAGAATAGACATTAATGGAGATCATGCGAGGGGATATGAAGATTTATTTCAATATACTGCAATGATTCACAAATATGATCCTGGTGCAATTTGTAAAGTGCTTTGTGACGTTGTGACTAGGCTTGAGAAAGTTCTCTTTTAGAGATTCTTTATGGCATTTCCAGCTCAAAAAATGCCCTTAGCAATGGCTATAGGCCATATATGGTGGAGTTCTATTAACCAATGTGGGTATGGATGCTAATAATGGAATGGTCCCATTAGCATTAGTAGTGTGTAGATAGAGAATACTGAAACCTAGACGTGGTTTTTAGAAATTCTGCATTCATATTTTGATAATGGATTAGACCAAATTATATTTTGCACGAAT

>Cs1g05980

TTTTATTGAGGTCGGCCATTACCTTATTGAGGTCGATCATAAGCTTAACATTTAAGAAGATTGGAAAAAACAAGAGAATCCAAAATAGATCAGAAAATGATAATGTATAAAGTCAAAAAAAATTTATCCAACCATTTAACCTTCGACTTTGTGTCTTTTATACCCATTCGGAGTATTTGGCGCCCTCTCCACGATCGTTACTGGGTTAGTTACACCATCATGGGTGAGTGGGGGTTGCAACCGCCTTTGAGTGGATCTCATGCTCGGACTCTCAGGAGAAAACCCATGTCCTGATAACTACTGTTAGCCAAGACTTTAATCCAACTTAATCAGAGTCTCTCGTCAGATACCCCCAACCAAGTGAAGTACTTGGGCAACCAAAAGATGGCTCCTCGTGAATGAGCTCCCATGATGAGTTGCTTTTGGTCGATGAAAGCACCTGTTTGCTCATAGGATCTTGGTCATCACGCTTTTCACCCCTAACACGAGCCGCCTAGTTTCTAGAGTTGAGAGTGAAACGAATCAGGACTAGAAGCTCAATAGGTATCTCCTCGACCAAGTTGCTTTTTTTACTGCTCGACATTTTGGCGAAAGGCTGCTAATATTTGCTCTTTACTTCACAAGCTTGCTGGTATCTATCCATTCAAAGACTGTTGTCGTTTTTAATTCTAGTGACGTGGCATGTTTTAGACCAATCATTTGAATTCAAAACAAGTGGTTTGCATTTTTGGTTACTAGAAACAATTCACTCTCTTTCAACAATTAAATCCTTAAATCTCATCTTTTTCTCATTCTTCTAAATTTATCACTCATTTTCTCTCAAAAGCGAGAAGCATTTCAGGCAAGGCTTTTCTTTGCTATTGTTATTACTGCCATAGACATTTTACTAATTCTGAGTTGCTCCAGGTACAATTTTCTGAACTCATCCTTGATTGTTTGTTGATTAGGATTGTGTCGCTCTTTCTTGCTGGTTCTAGGGATTTTGTTTTCGATTTCTGCAA

>Cs1g12800

GATTTTTGTTTTAAGGTCCTAAGAAGAGAAAAGAGATAAATCTATAATGAGTGCATTGCAGGGGAGAGTTAAAGCCACTAATCAACAAGTTGAATTCTTCATTGACACATTAATTAAAAGATCCGGCTACGGTGAATATGCTTTACCATAAAACACTCTTTTATAATCACACGATTAAAATTACATAAATTTTTTTATTATTTATCATTTTGACTGTATAATTATAAAAGGGTACTCCATGGTACAGAATCTCCAAGATAGTTATTGCCTTAAATTATCAAATTGGGATACTGTTTGCACGAGTGGAAAAAGAAGAATCATTCAAACATGCATGCCTATGGATTTTAATATTAGTGATCATTATTTATCTAATCAAATAATATTCAAAAGTTTAATGCATATCCTCTTTGACATACATTATTATCATCGAATTTGTATAAGGTAGTGGGCGTTAATTGCAACTCAATCGTGGGACAAGAATCAGCTAAAGTAATGCAAAGTATTATTTCCCTTGTCCAACTTGAGAAAGTACTCACAAGTAATCTAATTAATTAAGGAACAAGTTGATGTATTTGGGACAAGAATTTAACATGGGAAAAATCATTTCATCAGAAAATAAATCATATGTAATCATGTATGCTTATTAGTAAGCCGTTGGGGTGAAAACATTGTGTTTGAAGTGCAATTTGAGTGAATGGAAAACGCATCATGCTTTAACAATCCTCTAACTCACTCATTTCATTCTATATCTTAACAATCTCCAAATTTAGTTTGAAAATTCAATGTGAGGACGTGAGTTCAACGTAACATATTTTATTTCATATCTTAAAAATATTGGATTGAGCATAAATATATTTTGGATAAAACTGTAAGTTTATGTATCTGGGGACTCCTAATTTTAAGTTGTAATTATATTGATAGGTTGTGTAATACAAATACAATCAATTTAATAGAATCTCATAACCAGGAATTTGCCTTACTCAGTATATAAACACAAGGCA

>Cs1g20960

ATTACCAGCCCGCTTGTTCTTGTTGTGAAGTGGCCTGATAGTAAGATGAGCAGTATCACTTGATAGAAAAACAATGTTGTAAATTGTTGATATAAAAGCAATTTTTGATTTATTTTTATCCTCATTTGCAGAATTAAATATCTCACGGATCCAGCCACTAGGCTAAAGCTTAGTGATCAAAATCCATTCCTTACAATTGTGAGGTCATGGGTTCGAGGCTTCGAGTCTCCGCAACGCATTGTGGAGGTTTAATTTCTTTCCTCAATTTGAGTGAATGTGGTCTTCTTCTTTTCAAAATGTGAGTGGAGTGAATCTCTCTCGAATATTAAAAAGGGTAAAAATCTGAGCTGTGCTATATAATATTTGTAAATTAGTCTAAGTAATTGTCCGATTGTAAATATAGTGTATTAATCTCATGAAATATGAGTAATGACCCGTTGAGTACTCGGCCCACGCATTCGGTCTTATAATTGGCATTTCTGTTTTCCGTGATTCCGTCAGTCCTGGGATTGTTTTTAATTCTTTTAATAATTTATAAATGGACCTTGCTACCTTAGTGTGTGTGAGAACAAGGTTGTAATACTCCTGTAATTTTCTTTTTCATAGTAAATTACATGTTACCTAATAGAAATCACAACGTGAAATAAGTGATTAGTGCTTAACAAGAAGTCATATAAATTGAGGCACTTCTTAAATTAAAAATGAAAATCATAATCCGCTGTCGGGGAGAGTGAACACATTTTTAGATTGATTTGTGTCATACACATTGACACTACTAAATGAACCGTGCTATGTGCATAATAAAAGGCCTTGATTTCTTACATAATAATCATGTAAGTTACTCACAATTATAAAGTTACACAAAACAAATTAAGTTTAATTTATTTAATATAACTTTGTGCTTATATGATGTATTATGTATGAGATCTCTTGCCTTAAGTGGAAAAGACATCAGACATGACAGAATATCCCGTGTGGGTGACATTGGAGGAATGAGGGGA

>Cs1g22930

TCTTCTACAATTATATTATATTTATGAGCGCAAGTATTTAGAAAAGGTGAAAAAGAAGAAGAAGTATTTTGATTTCATAGTCCACATTGCGCGGAAGAGCAAAAATCTCGTTAAATTAAACTAATCATAAAATGTTGAGTCAAAAAAAAAAAAAAATCCTATAATGTTACTTGTTGCTCCCAAAATTGTCTTGTATATGAGAAAAACTTCCAAAATAGGAGTTGGGACGGATTTAGTTTAACTATCTGTTGGAGAAATTCTAGAATGCAATATATAAATGGTAGTTATATTAAATTCAACTACACATGTCACACATGTGATATTTAGTTGGATTATCTCTAATAGTTTTTTTTTTCACTCACACTTTAAATTCCTCATAAAACTCTTTTTTAATTGAAATTACAATTACCCAAATAAATTCCTTTCTTGCTTTTGAATGGAAATTTAATTAAACATTTAAATAAATTATTTTTTAACAAATTTTTTTACATCCACCCTAATTCATAGTTTATATTTATATTTTAAATCCTAATTAGAAAATATTTATTTATTTATTTTTGAAACTTATAAAGGTCTAGAATTATGGAGATATAAACTAGATAATAAGATGAAACTAATTTAGAAGTCAAGATTTAGCTTTAGTGGCAAGAAACAACCTTCCTAGGGCTTAGGTCTTAGTTTCAATGAATGGGTGAGTTAATAATTGAAAAAAAAAAAAAAAGTGACACCAATTTATAGTAAAATAGTTGATTTTAATATATGGATTTATATTTTATAAATTAAAGTGATGCTTTTTAGGAAATAAAATCCAGAAGAGGGTTCTACAAGAATTTTAGAGGGGTGATAAAAGCTCTACCCTTGGACAAAAGATCTGATCGAAATTTCAGCATCAGCGGCCCAAGTTTAATTTATGTGCCTACCGGGCCTTGGCCCAACTTGACAAATAAAAATCTGCTTCCGCCTTCCGGGTTTGAGTCAAACATGTGGACTCCGTTTACAGA

>Cs1g02610

GACATGCATATTTTATTAATTAGTTTCGTTATTAATAATGAACAAGTGCTCAATTAGTTGTTGACATAAGAGATGACACATAAGTAAGTATTCCAATTGAGTGTTATAAGTATGTATCAGGACATTACTCTTTCTTATCAAGAACGTTGTAGATATGGACTAATACTATATGTATCGAAAATAGAATAAAATTTAGTACAAAGTCACATAAAGTACTTATATTAACTTTGTTGATATTATTTTTATGAATACTATGCTATTTATTATTTATTCCCATATTGCTACAATTTAAAACTTAAATGTCTTTAATAATTATAGAAAAAATAAAATAAAAATAAATTAAATAAATTGGCTCTTAAAATTTAAATTTTACTCTACTAACGTACTTAAGTACCCAATATGATCTTACTAAACAGAATTATTATTAAAACAGCACTTTTATATCGGTGACTCATCAAAATCTAATATCAAATTAATTCCATTACATAATTTTCAGCCCCTTTTTTTTTTTTGTTTTCTTCCACTTTTCCTCACCCACCCCCCGCGGCGCGGCGGGGGGCGGAAATCTCAGACATAAATTAATCATCAAACATAGAGAGATTAATGCAAGCTTTTAACCAATATGCAACTGCTGATGGTAATAACAACATACAAGAGATTGTATGAAAAGTTTGAAAAAAAGAAACACAGAAACTAGTTATTTTTTTGTCTGTCTTTTTCTTACATTAAGCCTGTCTATCAAAAAATATGAAAAAGAGAGTTTCACAAGCAGCAGAATTTCGAGTAGCCAGTGATTATTAGATAAGAATGTTTAGACATGACTTTAACGTTCAAGAAGGGAAGTAGAGAATAAAACTCTTTATCATTTAATTTCTTTTAATATGTCCATCATAATAATTTGCGTGATAAGTATTTTAGTAACAAAAATATTTTATTAAAAAGTGGATGTCTGAGTATTTCCGCTAATCTATTTCCACTCACACACACAATACTGAACTCAC

>Cs1g18370

TAATGATAACCAATCTTTGAATAAATTAAATTTATCCCAAAAGAAGAGAAATTAAAGTGAATTTAACTAATGGGTTAACTGTAAAAACAATTAGTTTATTTGAATTTTTTAAAAACAACTAGAACTTTACTATAATTTATCCTTTTGAGTAGGACTTTCACACTCCTAAGTTATTAATATCAATATTTAACTCCACTTCATCATTTATAGTCAACAGAAATTTTTTTTCTTTTTCTTTTTCTTTTTGCCCTTTTCTGAAAGAACTCAACTGGCAATTTGAATTGCAGGGTAATAGGGTATTATATTAATCTTCTAAGACGTTTGCCGCCATTGGAATCAAGTAGGCAACAAGTAATGTTTAACACTTTCATCATTTATTTAAATAAAAGAATTTTCTTTACCTTTCGAATACTTGAGTTTGAGATCATAAATTAAAAGCAAATTGCAAGAATATGTATGAACTAAAAGTAGCATCAAAGCAAAATTTTGCTGGAGGTAAAGATAAGGAATATATCGACCAGAGACTCGAGGGAAAGTAAATGCCATCCATACCTTGAATAGCCTGGCTTCTTGGACTCGAACAATAATTTTGAAGAGGAAACATCCCAAAACAAAAAGAATATATACTATGCGTAACAGCAAAGAAGATGTTAATTTGCCGAAAGAAAAGATCGGATCAGCGAAAATGTGATTTGGAAAGAAAAAGTACATGGAATTGCTTGAAGGAAACTACAGATATCTTCATTAGAAACAGAATTTTGCACAAAGTAATAATGAATCTTGGAATCAGTCGGAGGAAAGCAGATACCATGACTAGCATCAAAGCAATATATTGCTTCCATCAAGCCCTTCAAGTAATTAAAGGATTCCTTAATTACTGTCCTATCTATAAATAGCCAAATGGAAGATCGTGGGCAGAATTATCACAACCAAAGCAAACCAAAACAAATTCTTGAAATTACCAACTTCCTTGGATCAAATTCGGTAACCATCTTTGACCA

>Cs1g21650

TGCCCGTTATCACAGATATCTATAAGTACTTGCTTATGCCATCCGATTTCCCTCATTTTGGGTGCCGGATAAACCTTTTACCTCTCGTTCACAGCATGAATTTAAATAATATCAACTGTTATTGGACTGATTGATTCATGCATACAGCAGAATTACAATAGCTTTCTCTGCAACATTTGGGAAATGATTTAAGTGTGCAAAAGGCACACAAAAGGTAAGGATTAGTAGCAGAGAATATTAACAAGTAAATGCACGTTATATCCTTGACGTATTCTTAATGATAAATCCAGGGAACATTCAAGGTATGGACGAGGTGCAATAGGAGCTGCTGGATTATGATTATGATGCGAAGAGCATTTTAAGATACCATCTACTGATCTACATCTCACCAACAGAGGTTACCAAGTTCTAACATACAGGTTTTAGCCACCATTTAGCAAAAAGAAAAACGAAATAGTTCTTGCTTAGCTCAATTTTCTAATTGGCAAATCAGATATAATCTGCAGCGGTGATCATCACATGGTTGTTCCATACCTCGGGACTCAATGATGGATCGGGGATTTGAACTTTACTATTGCTGATACTTGAGTTAAATTTCTGTCCAAAATCTCCAGCTGCTGGCTTTTGTAGCAGATAAATGACGGAATACGCATACCTTTTCACATTTGCAACTGTGAAGGCAAGAAATATATCCCCAATTAAAATTCAAGTTTCAATTACAGTTTCTGATGAGTTGGAATTTAGTTTGGAGTAATCTGGATTGTGCAAGAAGGAGGTCATACGGTTCCAGAGTAATAAGCCGAAGGAATGCTTAGCTATGTTCAAAAGATGGGACTTTGAAAGAACCTCTGTGAATTGTGATCATTTCCTGGGTCCAGCCCAAGTTCCAAAATCTGCGAGTTGCAAGATGAAAATCTTGTGTGTTTTGGTTTGCACATCGAGAGCCTGCTATATTTTCATGTCGCAATTAATATCATCTAATATTTGGGCATGGCTAAGGT

>Cs1g15760

TTTTGCCAAGTCACATTATTCATAACTTGTATCAATTCAAAATCATTTTTTTTTGTATGACTTAATTATTACCTGTCGAACAATTAAGAGATCATGAAAGACTACTTTTAAGATAACTATAAGTTATTACATTATCAATTGCCAAATAGTAGTTTCTAAAATTTTAATACTTTATATATTTTCTTCCCTATTTATACTTTATGCCAGTTATAAATTTTATCCCCACCCTTTTGATCTTTTAGCTATATTACTGAATACTAAATATCCTCTATAATGAACGTTTGTTGTTTCTATGGTTTTTATCTTAAACGAGCTGTTAACGGTGCAAAATGATCACATCTAATTAATAGCAGTAATTGTTTATAATCTGATTAAAGTGTCATTTCCAGTGAGGAGATAACTAGAAACAATTCCCAATGGGGGAGAAAAAATGACGAAATTTTGGGGAAAAAAAATCATAGTAAGATTCACATATATGCTAATGAACCTTTTGATAATTTTAAATACTTATTAATTACTAATTAATTAACCTATCAGGTGATTGAGTACATAAAACGCTTACTAGGAATCTGCATTTTTCATCATTTTAGTTGAAGACATGATATAAATTCAGAAACAAATCTGCTTTTCTTTTTAACCCAAATACCGAATTAGAATCAGAATGCCAACCTAAGAAACTACTCAAAATGGAAAAAAAGAAAAAAAGAAAAAAAAAAGCAGCTTTATAATAAGATATTGAAATCTCCCTTCTATCCTCCACAAAGGTGTCCTCATGTATTTGCATTTTAATCTTTCATCTACCTTTTGTCAATAAATATATATATATATTTAGATACTTCTTCATTAATAATATTATTTATATATTCTTTCACTTCAGTTTCGGCTTTATAAATCCCTAATTGCTGCTGCATAATTTCACTCATCACATTAAATACATTTTCCTCTTGCAATTTGCAATTCCTTATTGCTTGCTCTCTGCAACATTTTAATTTTCTTTAAAA

>Cs1g25230

TATGAATGATTTGCAATGAATTTAAATAAGGCAGAGGCTGTGGAAGAGCTCAACAGTTAATGATCGGTCACGGCAAACCGGTAATGATCCCGAACTTGCCTCAAGTATATGTTAGAATAAAAGATCAAGATTCTCTCCTTATCTGATCATATCCTGAACAAATAACTCAATTGCTGATTGGTAGTTAATAAAAAATAAATAAAAGAAGTTAAATCTTACTCATACACTACTACTAAAAGATACATGACAGAATATCTTGAAAAGCTCAGAAGAGATCAGATCAGGAGAGAATCATTTTCCTATAATAAATATACAAAATCATGGGTTTAAGCCTAAAATAATTCAGCATCACAAACATGTGCAAGGGCCATTTAATTGTTCGTGGCCTGTAACATATATGTCCAACATTGTCAACTTTGATTCTTAATATAATTGCTCATGAATATTCAAAATCTCGCCGACATCATTAAAATTAAAAGATAAAGTGATTCACCATTAACTAGTAGGTAATTCTTAATAAACTTAATTAAACTGACAATCCCCGTTTTCAACTTTCTTTTCACTAATATCCCGTTGTCCCCAGCGGTTAATTACATGGTAAGTCCATGGAAAGCAAGATTTTATACAAATTGAATTCATTACTTAAAAAAAAAAATTCATCAAATAATCCAATTGGAAAATTAAAAATCAGCACAAACAATACTCTAATTTGAACAAAATTACTCTAATCCAATTTCAACGTAAACATGGTTTGAACCTTTAATTTTCAACTTAGATATTTGAATGTAAATATAAGTAAAAACAGAAAACTTCATGATAATTAATTATAATTTTTCTTTAAAATAAAATATCAATAATATTAGTGATCCCAATTTATATCATTATGTGTTGTTTATATATATCATAACTATTAAATTATTATTATACGATTAATGGATGAATGTGTTAGATACAAATTAGGATCTTTAGCTTCAATCGGAATGTTTTGGTAACTTTGAAGA

>Cs1g08460

TGGGAAAACTTATTTACTTGTCTCATACTAGACCAGACATCGCTTTTGCAGTCAGTATAGTAAGTCGCTTCATGCATCATCTTCGGGAAGAACATCTTGAAGCTGTTTACAGAATTCTGAGATATCTAAAAAGTACTCCAGGAAAGGGACTTTTGTTTAGAAAAACAGAGAATAGAGGGATTGAGGTATACACTGATGCTGATTGGGCAGGTTCAGTTATAGACAGGAAGTCTACTACTGGCTATTGCACTTTTGTATGGGGTAATTTAGTGACTTGGAGGAGTAAAAAACAAAATGTGGTTGCACGAAGTACTGCTGAAGCTGAATTTAGAGCTATGGCACATGGGATTTGTGAAGTATTGTGGCTGAAACAAGTTCTAGAGGATTTGAGAAGACCTATAGTTCTACCTATGAGGTTATACTGCGACAACAAAGCAACAATCAGCATTGCACATAACCCAGTGTAACATGATAGGACCAAGCATGTTGAAATTGACCGTCACTTCATCAAGGAGAACCTTGAGAATAAGATTATCTGCCTATTGTTTGTTCCAACAAAACAACAAACAGCAGATATCCTAACCAAGGGACTCCTCAAAACTAACTTTGGACATCTTGTCAGCAAGTTGGGAATGATTGACATCTACGCACCAACTTGAGGGGGAGTGTCAAGAAGTTAGGATTTTTCAGTTAGTTTGAAAGTCTTATCAGAATAAAGTTTTAGATAATTAGTTTAGTTTTAAAGTCTTATCGGAATAAAGTTTTAGATAATATTTTTTAAAAAATTCAAGTTTAGATAAGTATCCTACAGTTAGGGGATATCTATTAGGAGTTTGTTTTCTACATTCTATTTAAGTGTAACCTGTAGAATGAATGGAATAAGGAAAATAGCATTTTCAGACTCTAAATATTTACATGCACAATTGGTGATCCAAAGGATAGAAACAATACAAGAAGATAACTCTCTAACAACAGGACCAAACTTCAAGTTTAGTGGGGAG

>Cs1g08350

ATTTTTTAGAGAAGTCAGTCCACATGCCTGAAACAACCAACCAAGGCGCAACCCGAGAAAGCAGAGCTTCAACTTCTTAAATTATATTTAGATATTAGGTAAATTTCATCCTACCCTCAGTTAAAATGGCAATCTTCAATTCACTTCACTAAAAGTATGAAAATTTTAATTAATCTCCAAAATATTATTATTTTTAATAGTTGACCGACGTTGACTTAAAAATACAAAAATGTCCTTGGCCCTAACATCAGTCAAATTGAACAGTAACAAAGTTAGTTGGTTCGGATTAGGGGCATTTTCATATTTTTATAAGTCAATGTCGGTCAACTATTAAACATAACGGTATTTGGGGGGTAAATTGAAGTTTTCATACTTTTTGTGGATAAATTGAAGTATGTCAATTTAATAAGGGGTTAGGAGGAAATTTACCCTTAAATGTATCATAATTATACTATTAGGTAAGCTAATTCAATTTAGTTACGTGCTTCAGTATGAGGAGAAATTCTAGAATACATTAGAAATATCGTGTTAATCATATAATATGTAAATCATACTATGAGCTTATGATATGTGTGCATTTATTTTGAAAAGCTATCACACAAGTGGGCTTCTATCAAATCTAAATACGCATGTCATATATGCGTTAATTAGTTGTATTACCTTTAATGTATCTAGTATTTCTCCAATATAAAAAACTAAAGGGAGGCTTTTTTTAATTATTTTTTTTTTGCCGACCTCGACACTACAGGGGAATTTAATAATTAAAAATAAAATGGATACCTAGTCATGTGTCATAATAGTCATGTGACATAATGAATCTTATCCCTCATGAAATGCATAACACTACAAGAATGCAAGCAACTAGCCAAACAAAAAATTCTTCCCACTTCCCAAATTTTAGTTAAATAGTCTTTGAATATTCATTGATCACGACACTTTGCAAACCCTTTAAATGACTCTCGGATCATCTTTCTTCTTTACCTGCAAAAGTGCTTTCTATT

>Cs1g08920

AAATGGGTGGTTGGATTTTGTGGTGAAGGTGACTTAGATTATCCTAAAATTGGAATTGAAATGGCAAGAATAAATTGAAGAGAAATTCTATTAAATTGACGCACTTGGGTATCTGGATCCGTATCAACATGAATCATGGGCTAAATAATCCACATTAATGCCAATTAAATCATGAGGGGGGAATCCACACCTCATGAACCACGCTTTAATCAATATGGTGCTAAGGGCTTATCGTGCCAAATAATAATAACCTTGTACTAGGGAGCCGGTGAAACCAAGGCGGTACTAGACAAGATTAATATTATTTGTCAACGAGAAGTCAAAACATCCACAAAAACTAGAGGGGAAGAGAAAATAAATTTACCAAATAAAGCCCATGACACATGTTGAGACTTCACCTTCAACCCAAGCTTGAAAGAAAATTAGCCACTCATAATTGAACTAGGGGCAAAATGAGAATTTATTAAAATACGAAGAAAATACAAGATGAAAAGACGCGGTTTGGTTGAAAATAATCCTGTGTGATGCATGTATCGTACGCGAGATTTTTCGTCCACTGATATGCTGCCACGTCACCATCAACAGTACATAAGAATTTTAGCCCAACAGGATCGTGACACCTCATCAGTCAATGCCACATCATCGGTCAATGCCACGTCATCGTCCGTCTATATGAACAGTGTCATGAACAGTAACGTATACAGTACTGTACACGTGAATAGTACCGTACATGTGAACAATGCTATTTTTCCTTTTATGCTCCTCCTAAGGTTTTCGACCGTCTTGAGTTCAAAAGTGATGTCCATTTTGGCATCTGATCTCTCGTTTATTGTGAAATGACTATGATGCCCCTAAAATACATAAAATACTTAATTAAAATAAAACAAAAGGAATTAAATCAAAAGAAAGTTAAATTCATAAAAGTAAAGGTGTTAGTAAGACTTGAAATGTAAAATGACAGTTTTCTCCTTTATAAATATGCATTTTCTAACACTCAACAA

>Cs1g23740

TGCCTATTAAATCTTGGATAGAAATGGTTGAAGAACATGGTGCCCATTACAAAACCACTTCTTCTGATGACCAAGTAAAACAATGGATGAGTTCCATTACAAAGTCCCCTGAGCTTATGCTCGCCTTACAAAACCTTTCCCAAAGCCAAATTTTCTCAAAAGAAGAAAAAGAAAACCCTATCTCCAAAGAAATCTCAAAACCCTCTTCTCAAAATGTGATTGTCTCTGGTGAAAGCTCTTCTTCCCAAATTGTGCTTTCCCAACCATCACCTTCAAAGAAAACCTCCGATTGGTTTGATAAAACCCATTTTCAAAATATTCTTTCTTTAGAAGATGGATTTTACCATGCTGATCCTTTCCAAGCAATTTCAAAGTTTTTCCCTAAGGGCTGGTTTTTTAAACCATGGGATTTAACAAAACCCCAGTCCTACTATCAAAGCATTTTAGAAATCACTGATTCAGTTAAGTTCAAACATTTCTTTCTCAGTGAATCACATTCAGAACCAGCCTATTCCACGGCTACTATTTTAAAAGTTTTGAGCCCAAACCAATGGGGTGACCTACTCCATAATTTTAAAACTTTCCCTTTAAATTTTCAAACACGTTTACCTCATTGTCGGACTTATTCCTACTGGGATTACCAACAAGCCTGGTATAACACCTTTTTTATCCAAAATCCCAAAAAATCTCATTCTTGGTTATTTTTCTTTAACCCAAAAATAACCGTTCAAAGCCTTCCAAACTGGTTCCATCATTAGTGGAATACTTTTGGCCCAATACCATAAATTTTAACCCCGAATGCTACCCATTGTTTAAATCTCTTCAAAGCCCACTATACTCCTTCTGAATCCGAGAAAAGGTTTCCCCCATTTCTCTGTTTATGTACAAATTTCTTCCTTCCATGGGTATGGATGTGGAACCTTCGGCTTCATACCCAAGATTCCCAACTCATCCTTCAAAGAACCTTTAAAGTAAAATGGTGGTCTAAATTTGACGAATAG

>Cs1g07510

TGTTATAGTTCTTGGATTCCTTTACTCGAGGGTTGAAGATTGTTTGACCATATTCATTCATTTCCTGCAGATTACACTTTTTTTCCTTTTCCACACTTTTGTTGTTGAATTTTCAAAATTAGCGGACAATTTTTATATACACACACACACACACATGCATGCATGTTATGACTTCAGTAGATATAGATAGGTACATGAATGTCATAGCGCACCCGTTGTCCTAGACTCTACTAAACAGTAGATGAGAGTAGGTACCATTCGTCTACTCCTGTGTTCTATGTAAGAGAACCACAGCCACTTAATTCAGGATGAAAGTCATAAAAACTTGGAGGGTCACCCTTCATTTTAACCCAAAGGCTATGAGACTCAGTAAACTTTCCCTACACCAAAGCATGGTTTAATTATTTATGGTGAAAGGACTAATGGTGGCCATGAACAGACTATCTGTCTACTCCTGGGGCCAAGCACTGGATATATGAGCCCTTCGACTTGAGCTGGTCCAATCTTGCATAACTGTGAAGTCAACACCAACGGACAAAGAGGCATTAATAGTTCAACGGGAAGAACAAGTCTTATAATCAATTTTGGCCGACTAAAATGTATTGATGATCATAAAGATTAACATTTTAGCCTCCAAATTTAGCTCGGCTAATTGTGAGTGCAATCACATGAGTTTTAGTGCTAATAGGAGAGGAGTTGTGATAAGTGTGTGCTTAGTGAGTGAAGTTTTTCATCTCATCCAAGCCCCTAAATATTAAGAGTTAATAGCTCGAGTGAAATACGTACCGTATGATTATGAACAAATTTAAGATACAATTGAAACGTGTATCACACGGTTGTGCGAAGAGACACATGGGAGGCTCCAATCTACCCCACAATTGACACTACATTAATTTCAATTGCATGATCAACGTTCACGTTCTCTGATTTTGCCACCTGTAAAAAGAAACAGAAGACCAAATACTAGGCGTAGACTAATAAGCAGACAAACAATAACTTCA

>Cs1g25770

GGGAGGAAATAATCATGGGATGTTTATACTTTGTTGAAAGGTTGGTTAGATTAAATCATATGATGGGTTAAGGCGACCCACTAACGCAGTAGTGGATTTTGATAATTGTGCGGTCATGTCACGGAGTTAATGAAGCAATTTTGTTAATTAATGCATTTTCAATGAGTTCACAGTCCAATCCAATATATTTTTGGGTTATAAAACATCTACGTAGTTGTATTAATTTATTTATATTATGATAAGCTTTTATAATTGCCTTAACTATGGTAATGATACGTATAAATAATACACACAGATCAACAGATAACGGATTATACAAAAGATAGCCCCCCGTTAATTAAACTTTTTCGCCAAACAGACGTTTTTCTTTGTATGATCAACGTAGTTTGTTTGTTTGAGATTAGGCCAACAATTGTCAACCAAATAGCTTGGTCTCTTTCATGATCATGAATAACACCAACTAGAATTCGTGCATTGTTTTATGAGCAAAACAAATCTTATACAAGATTCATACAAATAGCTTACGAGAGACTGTATTATTAATTTTTTTTATATCTGATTTACGTTATTAATTTTGTATCCGTAAATAAAGGAAATTCTGATGCCACGTGTTAATACAATACAAATACAAATCGTTACCCGCCAACATCGAGTTTCTGTTAGATGATTGGACTTGGTCAGCATCCAAATGAAGCACCTTGAACGAGCAGTGCTGACATGAAAGAAGAAACCTGAACCAAACGATGAAATCAATTTAAAAGCAAATATTTTATTGCACTCAACGCAAACAATTTCTCCGAAATATACTCTCAAGCTGAAAAGATTTTCACTCCACTGTCTCTGACACTGTCACTATGATTCAGCACATCGTAGCTCCATGAATGCTGCAAACCAACTTCATTGATTCGGATTCTGACTGAAACCCACATCCCCAGTTTTGATTAAACACCACAGTCTGGTCTGCTCCTGTTAAGTAGTAGTAACACACTGACAGTACCACT

>Cs1g10430

TGAAATTACCTCAACATTTATGCGTGAGGGTACCACTTTCTTTATCTTTTGTTTACTTTTCTTACAAATAGTTGACTTTTTCCTCTTGCAAAGCAAACAACCTTATTTCAAGTTGCTACTGGATCATGTATGTTTGCAGAATTTTCGATATTGACAGTACCACTTTTGTGCCAAATTATAGTTGGCTAGTTGGCTCTAGAACTTACTTTTTTTTCTGGTAATGTCTTCAATGTGTTCTTGTCCAATACAAAATCATATTACAACTTTCTACACATCCTAAACCAATGCTTGCAACCTCAATAAAAAGTAGTCAACATTTTTTGTATAGCTTCTATTCTTTTGTGGAAATCCTTTCAAACATCTTTCCGCCATCTTTGTAGGATGTACTTTTTTAAAAGTACGAAAATATTATTTCGAGTCAAGACTACAATTGAATGTCTGCAAACAATTCCTCTAAATTTAAGCATTGAAAACTACGGCAGCACTTAACTTACATTTGATGTTATTAATAATACCAGAACATGAACCGTTGGATTCCTTAATTTGCAGATCCAACGGGTGTGGTGAAACATAACGTAAAACAAGGTTCAGGTAGTGGTTGGATATTCATTTTTAAAAACAAAAGAATGGCTACCTGCTAAAACGGTTGTTGAAAAAAGGAAGCAAAGGGGTTAGCCGGTAAAGAAAATTTGAGATGGAAATGATGAGTGACGAGTGGTCGCTGGAAAAGCCAGAAAATTGAAGAAAGAAAGTACAGTATCTAGGTTTTGAAAGAGGAAAGCATTAGAAGCAGCAAACGAGATAACCAAATTCGTATTTCATTATTTCATATCACAAATTAGGGAAACCAAAAAAATTGCAGCCCCATAAAATCGAAGCTACAGGCGCTGGAATGTGGCAGAATTTGGTAAAATGTGAAGCATCATCTTGTGAAGTTGTCTTCACCATATAATTTTCGTAACATCATTCCTTGTTATGGGTGGGTCGTGATTATTGGAACA

>Cs1g19940

CCTTAAAAGATTTAATTTTGATGACTTATTTCACCTAGATTATTAAGAGCAAAAAGGTGGGAGAAAACATGTTTGTTGATTTAACGCCATATTGTGGCCTAATTTATATTTGAGCAGGTGATTTGTGGCATATTATTTCTCTACATTTACATGCCAAAGCTAAATGCTTGCTTAATATTTTCGAAAGCCTGTTGGCAGAATATAGAAGGCATTAAGGAGGTATTTGAGGGCAGAAACGGAACTTAATTATTTTATCTCTTTTTAGCTGGACTATTGGCACCCCTCTAAAAACTCTTTATCACTAGTATTCCACTTAATGCCTAAATTACCCCTTGTTTCTAATTTATTAATTTTTCGATTTCCTAATATTTTCTCACTACTAAAATTATTTTAGAGTCTTATTATATCAATCTCCACACAAAATTTATGAGGGTGATAAACAAATTAGTGAATTAGATTCAATATCTTTAGAATTATGATGCAAAAAGTTTTAGTATGATTATGAAGCAAATCCGAATGAAAAATTGTGGAAAAGATAAAAAAGAAAACTCCAAGATTCAAACAATTCATTAAAAAAATACCAAGATATATATTTTATAATTTGTTTGATGATGAGTTACAAATTTTTAAGAAGTTGGAAAGGGAATAACAGAAGATACTGGAACTAATGAGGTGGAATCAGAGCATACTAAAGGAAGTCATTAGAATTGTGCAGGATGCTGTGTTTTAAGGGAATTTTGGAGGGTTGCTAATAGTCTCTCTCTCTCTCTCTCTCTCTTTATTTATGAATACCGATCAAAATTGGACCTCGATTTGCGGCATATTAAACCTATAACAGGGATTAGAGTTGGTTCCCTTAGACCATGAAAACACACAGACATTTGGCATTTGGGGAAGGACTTGGCGGGACCCTGTTAATTTGATTGGTGTCCTCTGTCCTCACTCACTAATACTTCAAGAGAAATAATGATATCCCTGCTGCCTTTGCCAGAGTGTTAATC

>Cs1g25780

AGTGCAATAAAATATTTGCTTTTAAATTGATTTCATCGTTTGGTTCAGGTTTCTTCTTTCATGTCAGCACTGCTCGTTCAAGGTGCTTCATTTGGATGCTGACCAAGTCCAATCATCTAACAGAAACTCGATGTTGGCGGGTAACGATTTGTATTTGTATTGTATTAACACGTGGCATCAGAATTTCCTTTATTTACGGATACAAAATTAATAACGTAAATCAGATATAAAAAAAATTAATAATACAGTCTCTCGTAAGCTATTTGTATGAATCTTGTATAAGATTTGTTTTGCTCATAAAACAATGCACGAATTCTAGTTGGTGTTATTCATGATCATGAAAGAGACCAAGCTATTTGGTTGACAATTGTTGGCCTAATCTCAAACAAACAAACTACGTTGATCATACAAAGAAAAACGTCTGTTTGGCGAAAAAGTTTAATTAACGGGGGGCTATCTTTTGTATAATCCGTTATCTGTTGATCTGTGTGTATTATTTATACGTATCATTACCATAGTTAAGGCAATTATAAAAGCTTATCATAATATAAATAAATTAATACAACTACGTAGATGTTTTATAACCCAAAAATATATTGGATTGGACTGTGAACTCATTGAAAATGCATTAATTAACAAAATTGCTTCATTAACTCCGTGACATGACCGCACAATTATCAAAATCCACTACTGCGTTAGTGGGTCGCCTTAACCCATCATATGATTTAATCTAACCAACCTTTCAACAAAGTATAAACATCCCATGATTATTTCCTCCCCTCTTATTTAAATGGATAATTACCTAATGGTCCCCATAATTTACAACAATAGTAAATATGTCCACAAATTTAATGTTGATTTCGAGTTCGTTCCTAACCCAGTAAGCGCAAGCAAACATTTAAAAAAAAAAATTAAACAAAAGAAAAACTTAGGAGTATTTTGTTATTTAAAAAAATAAATTGTAGGGACATTATTATGTAATACAGACAATGACTGTTTAC

>Cs1g25170

AAGTTGGTTTTGTTTTTGTAATCGTGTTATACAGACATATTTATAATTAAAATTCTAAACTTGTAATTAAAATTAGAAGATGAACAAGAATCTATTTTATATGATATATCAAGTTGGTTTGTAGTGAAAATGAGAAAACAGATATTTAAATCTTGTGCTCGAACGTAATAGACAAACAAAATTACCTTCTATTAATTTAAAATACAGCTACAGAAATAATGGCACGTGGATGGTTTTTGTACAAATTGATTACTATTATTACTATTATTATTAGGTTAATGCTAAGTTACATGCATGTACCTTACAACCCTCTCACATGAATAGTGTATGAATACTATTCATGTGAGAGGGTTGTAAGGTACATGCATATAACTTAGAGGGATCCTTATTATTATTTTGATAAGATAAATTGAATATTAAAATATACAAATTTATTTTTTTAATGCAAAAGTTTGTCCTCATTTTGTCAGTTCAGTCCACTGTGGTTACATTAAGATATCCTGGTAACTTTTCAGTTGAAGAGACGTATCGTAATGGATTTTAAGCAAATTTAGAAATTAGTAAATGATGGAAGAGCTTACAGGGCTGTACACGTTTCGACCTAAGCTACGTGCCAAATCAGCATCCACTTTAACTAGCTTCATCTTTACCGTTAATTGGCATCACGGAAACGCAAGACAAAAACATCGACGGTTGGTGATCAGCGATTAACGTCCACATCAAAATTTGATGACGTCAGCGAAAATAGGAGTAAAAAAAATTTGATGACGTCAGCGAAAATAGGAGTCATCAAAATTTGATGACGTCAGCGAAAATAGGAGTAAAAAAAAAAAAGTTCGATGGGTTCGCTGGAGTTGGTGCCCTGGTGGTGTCTTTATTCATTGTCTTACCATTTATCATTGGAGCATCTTTTTGTATTTCATTTCTTGTGGTTCATTTTTTATGAGCGAAAAGTAACCATTCGCTTTATTGTTCCCTGAAATCCTTTTATATCTTTTCAC

>Cs1g02850

AAAACTCATCTTGTTCGTCTGGCTTCTTCCTCTATTAAATCATGGTTGATTAATCGGTGAAGGAAAAAAAAGAATAAAATTGGAAACAAATTGATTTGTAGGAAAATAAATTCCTGGGCTATTCTTTTTATGAGTAGAGAAATGGGGTTCACCCGCGGGCGAGAAAATGAATCTAACCAAATTTGGTCCATGGACGTGAGCTTAGTTCATACAGTCAGAATTCCAAAGGCAAAACTAAAAGTAAAAAATAAAAAATGGTTATAGATAATTAGAAATTCTCATTTTAAAACTCTTCATTATCTTACATTAAAAGGTAAACTAGCGTTATTAACTTTTAAAATACAAATATCACTTACATATTTAGTAAAACTTATGCTTAAAATAGTACTACCACTTAACACATAATTTATTTGTTTATTATTTCTAATTATGTATGCCAAATGCATATTATTGTTATTATTTTTTAGATATAGAAAACATGAAATGATATCAAATGAGTACTTAGGACTTAAACTAAAAAGACAACAACTCCAGAATACTAATTCTCTTTCACATTTTTGTTTTAAGTAAATACCAATTCAAAAACAATATGTCGAGTTTCAAATAGTGTTTTGGATATGGGTAAATTTCGGATTATCTCATCCTAAAATGATAATTTTCAAAATATCCTCTAAATTATTTTAAACTTCAATTTGCTCTATTTTTTTTCAAAAGATAAGAAGAAAATTAGGGGTTAATTAATGATTTTTTTTTCATCTTCTCTATAAAAATGAGGATAAGTTGAGGTTTACAATCATTTAGAGGGTATTTTAAATATTATTATTTCATGAGGGGTTATTCTAAAAATTAACCCTTTATATATTTTTTTTAATATTGTGTATAAAAAATATTAATAATAAATCTATTTATTTAAAAAAAATGGTTTCGAAAATTGAGTTACAGAAATAGTTACTGAGCACTAAAGCCTTATTATAAAGAATATACTTACTGGGCATTATTAT

>Cs1g10800

CAATTGGGTTAAAAGAATAAATATATTGTTACTGTACTTGAAAATCATAATGTTGGTTTTAGAAAACAATTTTTTTAGTATAATATAAAGCTGAAAATCGTTCATCTTTAATATTAATAATATTCTTCTTTTGTTGATTTATTAATTCTTTATAAAAAAAAAAAGTGTTTGCTAATATAACTGAAAATACATAATGCTCATCTGCACTGCATCGTGATTATGGATTGGCTGGAGCAAAGTTGCTGCAGCTTGAGAGAGATGAACGTTGATGATGACCATGCTCTTGACAAAATTCAGTTTAGTTCTGCGAATAGCTCAAGTTTCCATCAAAAGATTAATCAAAGAAACTTCCCCTGGTAAATTAAAGCTTATTAATTTCTTTTAATCCTTTCATTAAATTTGATCATGTTATGGATTTTAACCATAGAACATAAACTACAATATACATGGTATGATTTTATTCCCTATATATAAATGCAATTAGTAATTGGGTGCATTGAGTTATAAGCGGCTGATTGCTGAAATTGCACTCATCGATTAATTTGACGGGCTTCATTTTGCTTTGAAGAAAACACATAGCAAAGATGAACTCACTAGTTACGTCAAGTGTAAAACTATATAGTTAACTTATTTGATGTGGCCGGATCGCCTAAAATTTATGTGGATTTAATGATAAGGATTGAAGATGTCTCAACTCTTAGTAAATGGAGTTGAAGAGTGCAGTCGTGTACCTAATCCTTTATAATTATGAAAGGATTATTTTGTAGTAGATTTTGTAGGTCTATCCAATATTATTTTGTGATGATATGTCTATACAATAGTACCTTGGGGCTGCTCTTTTGCAAGGTAGGTTAGCCTTATTATCATAAGTATATTAAAAATTGAAGGTCTTGCTCTTTTGCAAGTTTCGGTTGTCTTGCTCTTTTGCAAGTTGGCTTGGTTTCATTTAAAATTCGGTCTCGGCTGGACTGGTGTTTTATTTCTGCTCACCATAAGAATTA

>Cs1g23380

AAAGAAATACTTTGAATTTTGATCTTTTGTTTGGGGGTTGAGTTCTCATCTTGAATTTGTTTTTCTGGGTTTTGTATGAGTCTCATAATTAATTAAGGACCAAAATTTCTTAATCAATTTGGTAACCAATTATTTGTTCGCACCTTAGAACCTTCTCACTGGCTAATTTTGTTCCACAAAAATGTTAGTTGTTAATTAACCAAGATGTGCCAGTCTATTTGTTTCAGCACATGCCCGTCTTCGGTCATAAGATTTAGTGATTAAATACTTGACAAAAGATCGGCGTACGTTACCCGTAGTCTTGGCATATGATTATGACATAATATAATTATAAATATAATATATTATAATCCGACATTATTGTATAATAAAATATAACTTATATTATATTATATTATAAAACAATATAATATAAAATTTTAATTTAATTATAAAGTAACTTTCATTTGTTGATGTCGAATATGATTATTTAATTTAAATTTAGTATCTTGGTAAATAATAAATGATTAAATAATTAAAATACTTTTTACATGTTTCAAGTAAAATAATTAAAACAACTTTATACAGTAAAAGTAAAATAATGTATTATCATTTATTTGTTAAATGTCCAGGTTTATTAATATTTTACATCATTATTTAAAATCAAATTTTATTTAAATATAAATATAAATTTATTATAAGTTATTGAATAATAAAAGTAAAATGATTAAAGTTAAAATATGAACAACTTTAATACAATATAGTTTAAATCATCCCCACATGGATTTATTTTATGCTCCTTTCATTATACTAACTACTTTCTTAATGCAATACAATGCAACTAAAAATTATAATAAAGTATGAATTTATATAAAAATAATGTTTTAACGCAATACAATATAGTATAAGGCAAGCTAGCAATGGCACCATGAGTTTATTATATGATTTGTTTTATAAAAAATCAGGGTTTAATGAATAAAAGCCATCGTGGGCCTTAAATTGAGTTAGTTGAGATGAGTGGG

>Cs1g13840

TTTTTTCTTTTTCTTTTTTTCATAGATGCTTTTCCTTCTTAAGTACTTTCTATTGTTTGTAGAGGACAAGATGGTCCTTTCAGTTTTTGTTCTTTCTCTTGTCCAATACAAACACTCACTCTCCTTTCATTTCTTGTCTCCTTCCATCCACACTTCTCCCAACACCACTCAACATTTAAAAGAAACCCACCACTCTCTTCTTAGTCTCACCCACATTGTCCCCATCTTCTCCATCATACTCTCCTCTTTAGTCCCCCTTCTTTCTTGTATGCACATACTTGTCCTTGATTTGGTTGTTCTCTTTTCTATTTTTCTTGTATATTCTAGATTTAATTTATATTTTGTAGCAACTAGGATAATGATAAATAAATTATATCTTCCTTAATGAATACCTATGACGTATGTATTATCCTATCACTCAGAATCTCATCTTTCCACATTATTTCCAATCTTTTGATTCCCTAAATTTGAAACCATTTTTTAATGATCAATATTATTTTGTTATGTATTATATGAATATAAATCAAACTCCAGGTCCCTTTAAACATCTCAATTTTTTTTATTAGTTTAAGCAACTAACATTCTCTCATGCATGTATGTGTGTGTATGTATATATATATATATATAGAAATTATTCAATCTAAAATTATTATAGGTGTAACAATTTTTTTGAACTTCAAAAAAGTATATGATATGATTCTGGGCCAACACTTATATATATATACATAATTCTCACCCATTTTTTTTTTTTTTTTGAGGCGCGCCTTCCTCTTTCTCTCTCTTATGATCGTCTCTATAAATACTCTACAAAAACCATCATGTATGTCATCTCATATTCTCCCTCTCTACTCATAGTCATCCAACTTTTTGCTTACTACTATTGCTATTTCCTAGCTAGTACTACATACTAAGCTTTTATTGCTAGCTCCTATTCCGTAAAACATCTAAAATCCAAGACACACACTTTTTTCTTTTTCCTCTTCAAACTTATTAATTTCATC

>Cs1g08790

AACAAGCTACTTCTTTTTTATTTCTTTTGTTTCTTTTTTTAGTAGACTGTGCTAAGAAAATAATTTGACAATTAAAAGTAAACTTTCAGAACTAGCTAGCTCGTTTGTTTGGAAGTAGTAGTAGTGCTTAATATGAACCATATAGAGATCAATTAGATGATAATCTGTTTGCATAAATTTACAGAACTATCCAGTACAAATAAATGAGAAATATAGCAGACATATTGTTAGAAAAAGATTGATGATGTTGTAAAACACTTGAAAGCTTTTTTGAATGGGAAATAATGCAGTACCGACAAAGAAAATTAAGAAGCCAAATCACTATTACAATCAAATAATTCATAATATGTCAGTATTGACACTACTGTTTGTAGAAATGTTAATTTTTTTATGTTGGCTTTATTCTCCGATCTTATTATAATTCAGAAACTACTTAGAACTAGTCTCAATATTATTATATATTAAGAGCATCCAAGCCCAGTACTCTACCTTACGGTTTGATATAGGGAAAGATGAGAGATGAAACGGGAGATTGGAAAGTGAAACAGAGATCCCAGAAGAAAGATGAAAGATAAAGAGTACACTTTTTGTCACCTGTGGGCAATGGCGAAGTCCCAAATGGGTGTTGTTTCTTTATCTGACTGCAAGCTATTTGCATATGCTCTGCACTCAAAAGAGATTCCCCAAAAATCCTATTATTTTTTTCTCCCTTTCTCCCCGTTCTCTATCGTAAGCATTTTTCTCTGGTTTCATGTTTTAAAAGAAAATTTCCGCGTTTGTGCAAGCAAATTCTGAGAGATGCAACTAATGTTATTAGCCAGAATAGGTATGGGTATGCATCTTCAATTAATGAATCCATCAACGATCCATTTCCGGTAACTACGTTACAGATTTATAACTTGAATGCTACTTGTCTACTCCCACAAACAAAAAGAGAACATGAAAGGCAAGCGAAGAATAAATTTGGTAATAAGATCAATGAAAGCCAAATATATATTAAA

>Cs1g02550

TCAGAACAAAAAGGAGAGTAATATCCATAGGCTGAAATAGAAATTGGGAAGAAAGAAAACGGCACCAGTGAGTTCCATTAAGATTGATCCTCTCTATCTAAGTTGTTGAACACTTTTGATTTTCTCTGTTGTTCTTCCTTATCTTTCAATTGTATTTGAAATTCGAACTAATTTGGGTAACAAAAAAAAAAAAAAATGAAAGTAAATCGAGCCCTCTCGTTTTGAACATGTTTAGTTGTTTACTTCCAATGACCGGTTGAATTTTTAAATTAAATCGGCGTCTCTCGGTTACTTCCCCCCATTTTACTCTAGCCACTTGATTTTCAGTTATCGTTTAAGAACCTTCCACGTGGACATTATAATAGGATATGACATGTTCAAAGACGTGTTCATGTCATTTCATATTAGTGCGCACAAGTCCCAAAGTTCATGCCGCCTGATTTGTCCCACGTTACTTTTTCTCTGCCAAGTTTATTCCATGATGATTAAAATCTCTCTGGATTCTTCGTCTTGATAATCAATTTCTAACTTATCTTATCTGACTATTGCATCAGGTTATTAGTTTACTTGTCCTCGTACTCCGGGCATAGAACGGGCAATCCAAGGGACTAATTGTTGCATCTATTTTCCTGAACACTTTTGTTGGGTTCAAAAACCTAATATGATCATAATTGAAATATCATTCTTCGACTTTGATTATACATGTGTCGTATCCTCAAAGTGTTCCAACACGACATGGATTTAGAGAAATCTCCCTATAAGAGGTCATCCTGCTTATGAGGTAAGGTCATTAATCAATAATCAGGCATCCTATTTATTTTGCACGTTAAAGGGCATGTACGTTTAAAGAATTCTTTAAAACTATTTCAACTTTTAGGTAAACCAATGATCCTCTTTATGTGTTGAGCTTATGTATTATCAAGGACCTAAATCCTCTCTTCGCGTGAGCTTTCGCATCAATAAATATAGATATGTGATTGATAGTAAATAACGGAATAGTT

>Cs1g15960

TCATTATTATGTTCAAAACACATTATATATATAGTACATACCCTGATTTTTGTTGCGGGCTGTCACGCACTCGCACGATGGGCCAAGGAATTTTCAATATAACCTCACTACAGTCTGGATGTGTTTCGTGATATGCTATCTTCTGAAGTACTAGAGGTATCATGTCTGCAACAGACATCAATTATCTCACCAAAATTGAATCCTTTGTTAAATCTATCATGGAGTCATAAATGGAAATCTTGCCCCCAAGTATATCGACCACACCGAGAATCCAATGATCATTCATGAATATTGGCAAATACACCTATGAAATATAGACAATATTCTTAACAAGTAACGGCAAATATATCTACGAAATGCGGTAACTGTTTTTATCTACTTGCAGTGCATATGATGTAAAAAACTAAGCATACAATGTGAAACTTACAGTGGAAACATTTTTCCAAGGAGTGGCACAGTCCAATTTTGCACCATTAGCATAATCAATGCAGCAACAGAGAGTTGTTCCTGGCCAAATCCAGGAAATAAGAGGAGAGCAAAGCGCCAATGGCGCGCGAAGCACATGCAGAACGGGCACGGAGAAAAATCAGTGTGGAGGAGAAGCATCCGAGCTCATTCCCTTCGCTTATGGGGCCAAAGCAGTGCAGTTGTTCCTGGCCAAATCCAGGATTTGGGGCTTCTTGCTACACCTAAGAAGAGCTTTGTATTCACTCGGGCAGAGAGAGAGAGCTTTGTATCTTTGAAGAAGAAGAGACAGTAATCAATTGGAAGAAGAACGAACGGTAATCAATTGGAAGAAGAAGAGCTTTGTATTCACCTCTAGCTTCAACTTCAGGGATGGGTGCATGTTTTGTGACTGATTGTGCAGGATAATTTGGATGATTTAAGTTTTTAATTAGGGTAATTTCGTAACTGCACATAAAAAAAGGCATATATGAGAAATTTTAGAGTATAGAGGTGTTTTTAGATAAATACCCTTTTTTTGGGGTATACCATAGACA

>Cs1g09800

TGAAATAAATGAAAACAAAATAAATGAACACAAATAAGTGAAAGACCACTATTAAATTTTTTACAGCGTGATCAATTGTCAACAGTTATAAAATAAAAATAATAGTAAAAATAAATTTAAATTAAATAAATATTGTTGTAAATAACATAAATTTTTTTTCTTATTTATAAAAATAATTCCTCCAACATTGAAGGATCATCATAGAAAGAGCAGGATACTCTACTAAGAAGAAATTTATTATTTATCTTATAGTAAATTTTTATTTGTCATTCAAGTTTTCTTATAATAATTTTTTTATCATTTGACTAATAATTTTCTTATATATTTATTATAAGAAAATTTTCACAAAATAAATAAGTATAAAAAAATTTTATTATTAATTTTTTTGTTCATATCAAAACATATATTTCTTATTTTTAATGCTTTATTTTTTATTTAGTTTCTATTCATGTGATTTATTTAATTTAGAATAAATATAAATAAAAGAGTAGTTGAAAATAAGGGTAATATTGTAAATTTATACTATTATGGGGGTTTTTGGCCATTATAAAAGCAGAAACGTTCTTATTTATTGAAAAATAATAATAATTTTTGTGATTAAACTTTATTTTGATTTTCTAGTATTCTTTAATTTAAGGATTAAGTTATTGTAAAATTTATAATTTTTATCCACTCTATTTTTTATATTTTAATATCAATTTAATAATTAATAACAATTAATAATTATTATAATTTTGATATAATTAAATTTTATTAAAATAAAAATTATTTTGATAATAAAATTCAAACCAAACCCAAATGGTTTGGTTTGTTTGGGTTAAAAATATTTGGGTTGGTTTGGGTTGGATCCAAATTTTTATGGTTTGGTTTGAGTTGACTTAAAATCCAACCCAAACCAACCCATGCCCACCCCTAGTCAGAACCATTTGGAATAGGGGACATCACACAACAAGCTTTTACACATGCATCATTTTAGGTACAAATTCTCAACGTACCTATAA

>Cs1g15780

CCACTCGTAAAGGATTTCTTGACATGCTAGAAGAGGCTTCTTGTAATGAAGAAAATTCGGTGCTTGATGGTTTGAGATGACAAATGTTTCTCTTTTCGATGTTCTGTAAATGCGGGTTTTTGTTGTATGATTTTGAAGTCTCGGAAAATGTCTCTTGTCATTCTACCACATTGCAATAGTAACTCACTAAAACATACACATTGTGGAGCTTTAAATTGAAATGTCCAATCTTAATAATCTAGCTTGCTGCTTTTGCCTCTGCCCTTTTCTTCCTTTTTATTTCTTCACTAATCTGTTTTTATTATGAATTAAAATTCTGAAAGAGATCTTTTGCTGTTGATGCTGTTGTGGTAGTTATGATCTTTGATTCAGAATGGTTGATAAACTTTCTAAATAAACATTGGTCAAACTCTTTGCAAAACTCTCTTTAATGAACAAATCAATTGTGTTCGACGAAATACTTGGGTACTTTTTTTTTCAATTGTGTTCGACGAAATACTTGGGTACTTTTTTTTTTTTTACTCTAATATTTTCTTTTTAATCTTTTTCACGGGATTACTAAAGCATTAAATTAATTTAATTTTCTAAATGAATATTTATCATTTTGTTTTAAGTTTTAACCAGGTATTGGAAACCAAAATTCATAACCAAATCGCAGAAAATTTGAAACACAAAAATCAAAGAAAATATGAGAAACTAAGCACAGAAATAAAGAAAACTATGAGAAATTATAACTTTCTTCTCAACATTTTTTAAGATTAAGTCTAACACACACTCAGTTTGAATCTTAGATGTCTCACCTCTTTTTATATTTTTATTTTATTTTTGATTAGATTAAATATCGCAATTCCAATATTTCAACCACCACTAGAAACCAAAACCCAAAATCATTAATCAGTAATCAAGGTGCAAGAAGAACCAATGACAAGAATTATGTAGCGTATGAGAAATAACAAATATGCTTGGGGTCAATTCACAATTTATATAACTTCCTTCCCAAA

>Cs1g11510

AAACAAATATAAATTAATAAAACTATTTAATTATTTAATTAATTTTTATCAGATATTTTAGATATATTTTATTTTATCAAAACGTTAGCAAATAATGTATATATAAAATCAATTATTTACTTGTATTATTAATTTTCATGATTATTTTTAATCTTACATTGGTGATTATTAAATTCAATACAATCAATTATTGTAACAAATTATTAAATAGTTAATTTTATTACCATAATGATACCAATTTAGTTAGACTATAAAAACAAAACAAAAAATAAATTCAAGTAAATCATGAATAAAATAATTAATTAAATTTTAGGTTACAAATTAAAAATAGTGTTATTTATGAAAGAAATATGAATAAATTTTTTTCTCTTATTCTAATCCCATCTAAACCCACTTATAAATTGGGATTGTTAATCCCATGATTTGAGGACTAAATTTAAATTTAGTCCTCCCTAATTCAATCCAAACAAAGTAGCCAAACATGGGATAAAATATTTAATCCCAAAGTTAATCTAATCCCATACTTAATCCTAACTCCCAAACATGCACCAAGTTTGACAAGCTCTTAAAATAAACTTTTAGAGAAAGCAGTAGAAAGCAATATTATTAATTAATAGAAAGCAACCACAAAATGATGCATATTTGTTGAATGTATTTTTCCAAAAAAAAAAAAGGTCTATACATAGTATATATCCTCATGACAGTTATAAATTACACACTCTATGAAGAAAATCTATACGACTGGAGAAGTATTCTCACATAAATATGATCAATAACGTTTTTTTCTTTAAACGAGAAAGATTTATATAGGAAAGTCACACCACATTAGCAGCATGCACATTTAGCACAATTGGAATAATGACAGTGTCTAGTTAACGTTTAATTACTTACGCCGCTCGTTAATAATCAAATAATAAAAAGCAGCCATTTTTAGAATTTAAGAATTAAAAGTTGCTTAACAAAATAAACTTGGCTTCTTTATCTCTCAAATTAATCAAACA

>Cs1g19090

CTACGTAATTGTATGATTCCTAACTTTACCTAGCATACAAACAAGATCAGTAGGAGAATAAATACTTGACCCAAATGGTGAAAATTTTCATCAGAAAGTTAAATAAGAAAAAATATAAATTAATATTGAAGTTCAACAATGGATGATAGGAATGATAAGAGTACCAAATGCAAATCTGTGTTCATAATTGTCTCTTACTTGGATTCTAGTAATACCGACCAACTAATTTCATGTTTTTAAAAACCTTGACATGGGCTATATTGTAGTACAAGTTTAAACTCATGGGATAGTGTTAAAACCAAATAACGACGAGCGTTACTAGATAATATGTATCAAAGTCAACTAATGCTCAAGTGAGGCATACACACCCTTTTTCACATGATAAATTTTAGATAATATTGTCGACCTACGCATGATGGAGGTTTTAAAAAAGTATTTTATAGTCTCTTCAAGTATTTAAGCTTGGCACAATCTTTAACTATATATGAGCAAACTTTTGAGTAAGAGTTGAGTACATAAATTTCTATATAATATTAGAACTAATTCATGAGCTAATTAAAATATAAACCATCCATAACCATGTAATTAATGATAAATTAAGCTGCATGAATGATGAAACCACATTGGAGATTTTGTTAAGGCCGAGTAAATTAGATAACGAGGTGACGCATTCAAACTATACAGTTCATCCAACGTAAATTTTATTGTTAGTAAAATTACCAATAAAAGAAAATTTTTACACGAGCCATTGGATGAAAATAAGTTTAGAATGTCTACATACAATTACAGTGAGGATCCATTATTTTGTTAGTGTTGGTTTCCTTTTTAGAAAATGATACAACGACGGATATCCAAGCATAATTAGTAAGTTAAGCACTTCCAATTATTCTTAACTTATCTATAAAGAACAAACAAAACCGATTGGTGAATAAATAGTAACGCCCAATGGCCCAATTTTATCACAAGAAAATTAAGTTCGCAAAAAAAATTAAAGTTCAACA

>Cs1g16800

ATAGTCAGTAATGTCCCCGACAACTACATTTTTATGCAATTTAACTATAATTAAATAATCTTTTTATGTTTAGCCATAAAATGGAGTCATAACTTAACTAGCCGTGAATACGTTTACCATGCATGTGCAATCACTCGAATTTAAATAATTAATAAGAAAAAAGGGTGGATGAAGAAGCTTCTCGTCGCGTCTAAAACTTTTATAAGAAAATACTTTCCAGTTCATCTCTCACTGAAAAATTTATATTTCTATAATTCTTTCCTATATCATGCTATTAAAATAAGTTTGAGATTTTTTTTATATTAAAAATCTACATCTAATAATAAGAAGAAAGATTGACCCGATACCTATTAACTTATAAATACTACAAAGGAGGAACAAATTATCATGGGCTCTCTTGCCCGGGGAATGGTCCAAGCGCCTATGACTTGGTCTTTGATGCGTGAATAAAAACCTCAGATAATGCACAAAATATGGAGTTCTGTCAACCATTTATTAATGTTTTTCCTTTTTCTTTTTTAATAGAAACCATCTAATACTGCCGTTTACACTATACTGATAGAGATCGGAAATATAATAACTATATTCCAATAATTGCATTGCATCAGAGGCTAAATTTCCATACTCAGTACAAAAAAGGCATCATCACAGTACAAAATATGTACACTTTGGTCAATCATACCATTATAAAAAATATCGAGAGTGTGAACAAATGAAACAACCACGTAGAATTGTAGATCCTTCACCTAATGGTTAATATATAGAAAGTTTGTCATTTTGTAATATAGATTAATGGAAGATGATTAACATAATCCGACTGCATTAATCAACTTAGCGGTCAAGGCATCCTACCCTGAAGAATTTTATATATGAACACATCACTATCTTGAACCTAGGCAAAAAAAAATCCTTAATCAAACTCTAATTGGAGAATTACGCGTCCCTATACTATCAATTAACGCCCTTAAATTGAGATTAGATGCTGCTTAGAGAGGCCAATA

>Cs1g06850

CAAAAAGAAAATGAAAAAAAATGACAGGAGCCTCAGTGTTGGGGGTGAATATGATTTACAGGTCGATTGCCTTCCATTTCCCCTCATGGTTAAATGTGTACAGAGAGATATTGGGTGAGATTTGTAATATTAGTGAGTTCCATTTCTTCTTTCTTTGTGGTTGTGCTCATCGGAATGGAACACATGGATAGCCATTCTGATTGAGCATTGAGAAAAAAAAAATAGATTCGTTAAAAAAAATGAAAAAAAGGAAGAAATCTACACCGAGAATTTAGAAATCAATGTATTTCCGGTATCGGGAATTGTAGAGTTATGAGCATGGCAAAAGCTTATTGGATTTAAATGAAATTCTTTTGTGATTCATTTTGCCAATCTTGTTTGTTACGCTCATCAGCAGGCTGCTGGGAATGGAATTAGGTGTTGATATAAAAAAAATATGGTTACTGGCAGAGCGGCACTTCAAGTTTAAGCCTCTGTGTGTGCGAGCAAGAGTTTTTAGGATCAGCTGTAGCACTTAATGCCTAAACCTTGTGCGCAAGTATTGGCACCAAAATTATGTAGAATGGGTTGAAACTGAACCATACTATGTCATTGTAACATTAGTAGTCATATTGTTTGGGACACTTTGTACAAAGTAATTTAAAAATTTTGCTTTGAGATCAGTTAGTTATAATCAGTGTTCCGTTTACAAGTTTAAATGTTCGGTGTTCCGTTTACAAGTTCAAATGTTTGAATCTTGATTTCACCAATGCATCAATTTGTCCTTGAAGCCAAGTTATGCCAACGCAAGCTCAAAGCCTAAACGTGGAATGAAATTTTAACCCATCGCTACTATCCGTTTCAACTTATTCAGACTCTCAGCCCATAACTATTAGGAGTGAAACCTTTCCGAGGTTGGACCAAAATACTCTCATTGGCCCACCAAGAAGGTTAAATAAAATATGGCATACAATATCGGGAAATCCAAACCTTGAATTCTCATTGGTGGCTGAGAATTGATG

>Cs1g08205

TTAGTATTAAAAATTGTATTCTTTTCTTCAAACTTTCATTATTATTTAATTTTAAAAAAAAATATCTTAAAAAAATAAGAAAAACCTTAAAAAATAATAAATATTTTATGGGTTTAATATGATTTTATCGTGCCTATAAACTTTGTGTGCAACAATTAGTTGAGAATCTTTAAAAAATTACTTGTAGTGAGATGAATTTTTTTTAAATTTATTTTCTCACTATGTTGTATCCTAATTTTGTTACTATGTATATTACAAGAATATTATTTTGACTGAGATGATTACTTCTTAAATTTGGATTATTTTAATGATAATAAAAAAAATTATATTGTGCAAGTAAAATAAAATTGAAGAGAGAAAGAAAATATAAATGGGGTTAATCCTATTTATATTTGCTCTTAAATGTAATTGTAATATAACAAGAGTTTTATAAGATTAATGAAGGGGTACTAATAGTCCAGATCTTTTTTTTTTTTTTTTATATGTTTGTTATACTTAATATCCTCATTAAATTTCTTGTCTTTTTGGACCCATTGATAGTTTGAGATTTTTTTTTTGAAATTCTTCCACTTTTATTCCTTCTTTTCCTTTTAATTAACATTATTTTGTTTTTATTTTTTAAATATTAGGGAGATACTAACCGGACCATAGTACAGTTGAAAACATTAATTAAAAAAAATTATGTTACTCCATTAAAAAAATTAAGGTATTTATTTTGACATTTTAAATTTTAGTTTATTAATTTTTAGATGTCAAAATTTTGAATAAATATGAATTTGAAAAAATAAAAAGAGATTTTAACAGCATAGAAATTTATGGAAAATATCTGTTATCACAATTTTCAATAAATTTGAAAGAAGTTTCAAAATTGTTCAAATTCATCCAGACTTGAGGCTCCTATAGCGCAATCCACGTGCGGAGGAGATGCCACGTGTGGATCGGACCGTTCAAAATATAGTGAATATTAAGCGCGGCAAATCGCTTTTATGTAGCGGACGTTG

>Cs1g23040

ATTAGATAGAATATTTATTTAGTATAATTTTTAAGAAAAATGTTTACTTAATTTTCAACCACAAGCCAATAACCTATTGCTTTGATCAGCACCTCCTTTCTTTAACTCTAAAATAGAAAGTTTGATTCTCCTTAATGATTAAGCTTCAATAGGCTTGTGTTGTGGTGCTTCGGCTGAATTATATACAAGATTGAAAAATAATAATAATCACAAACTTTGGTTAAAAAAATAAATACATTTGCTGAAGACGTGACTCTTGTTAAATAAAACATGCAATATGGCAAGAAATTTTCTTTTTTAGATAATTAAAACATCTTAACAGAGATTTGTACAGTAAAAAAATAAAAAGTAAAAATTCAACCACAAACTAACGTCACATATCAGACCACAATTACGATCAGTTGAATGAGTTAACAAAACGAGATTGCCGACAACCAAATTTTTGGTAACCATTCAAGTTGAAGACGAGGACAGAAGCCACCATTAAAAATTAAAAATTGTACGAAAAATTCCAAACTCACCGACCCTTTGCTCTTCCACCATCCAAGTTCATCAAGTTGACCCAATTATGCGACGCCTCGGAAAGTGAGAAAGAGAGAGAGAGAGAGAGAGAGTACGGAAAATTGGCGTATTTGCAGTGAATCTTATAACCTTCCCTTTCAAGCATGAATACATGTACACATCCATACATACATAAACGTAAACTAGTTTTTATGTGCGTCATCTGTCGCTCTTATTTATCTTGGAAGTTCATTAATTAATTCAGACACAACAATAGAAAGAAAAAAAAAAAATGGGACTGAACTTGGATTTGAACATGGCTTACGTTCCAAAAACAACCAAGGTGTCAAAGCTCCATGAGTGTATTAAAAGATTGGAGGAAGAGAGGAGTTAATTAATTCAGACACAACAATAGAAAGAACGTAAACTAGTTTTTATGTGCGTCATCTGTCGCTCTTATTTATCTTGGAAGTTCATTAATTAATTCAGACACAACAATA

>Cs1g03840

ACTGGAGAGTGTATGTTTATAAAGGAAAAAAATCATCATTTTACATTTCAAGTCTCACTAACATCTCTACTTTTTATGTAATTAAGTCTTTTAAACTTTGATCGTGCGTGTTTTGTTATAATTTGAGTATTTTATATATTTTGGGATATTTTGATCATTTCAAGATTAACAAGAAATCGAACACCGACACCAACATCATGAACGGATTTAGAAGCCCTAAAAACCAAAGGAGCGACAGAAACCAAGGTTAAAATGGATTCTAATTATATAATGTATCGAACTGATGCACACTCCTAGCTTTATTTGCAATTTGGAGAGACATGTGTAAAAAAAATATTAGAGAAGCCCATTGAATACTAAGGTTTAAGGGATGGACAAAGAAAAAAGAGATTTGAATAGGTGTAGAGAATAAATTATTTTATTTAATTACATAATTTTATTCTTAATAGTAAATTCATTCGTGGACATAACAGTAATAAATATTTTTATAAAAGTATGGGTTTGTAAAGTATTGCGGATAGTTCAAATATCCTTACCCCATCCTAAGGTGCCGCTTCACTTGCTTTTATATCCACGTGATTTTTTAACTTAAGTTATATGGCAATTTTGTTTTAACCCCAACTTATTTTCTTTAATTACTTAAACATCTAAACTATTGGAAGCTTTGGGCTTGGGCCACCTAAATGAAGTGAATCCAACACTTTTCATGAGTAAAATAGGAAAATAAAATATAAATGGAATAAAAATTTTAATTTTTTAAACAAAACCATATTTTTCATCAAATAATGTAGCTTGTTTATGGTATGATAAACGCTTTTTGTTTGATACGAATCTATCTGAGTTAAAATTTCAATGTCAAACGATGCCATTCTTATCAATAATGCAGTACTTTTATTATGCCGCACACCACACATTTAAAATTAATTTATCAGAGTAGTTGGTATGATTGTATACTGATACATAAGGGTTTAATCTCTTTTGCAATTTGCACATCAGCAAAA

>Cs1g04990

TTTACTTTGTCACTATTATTATTAATATTATTAACTTTTCTGGCATTGATCTTAAAAATGGGCGCCTTCTTTGGGCAGCCATTTGCTTTGTCTACATTCCTGTTGTGCTCATTAAGGAGTGGGGTTCCTTAATCGATTTTAATTAGTTGAAAGAAAATATATCAGAAGAATGGACTCACCTGTGTTGCAACCGTTGCAACATACGACTACTTTTGTCATTTTGTAGTTTGATTACACCACACCACCTTGTATTTTCTGTAAAATTAAATAGGTACTAAAAGTTACAAATTTTTACATATAAATCCTTTTTTGATAAAATAGCATTTGAAAAGAATTTTAATGATGGTTATTATTTATCATTAATATCATTATTATTATCTAATATTATTGCTATTAAATTTTATTATTTTAGGTACAGCAGTAATTATTATCATTATTAATATTATTATTGCCGTTACTTTTATTGTTGTTGTTGTTGCTGTTATTATTATTATAATTAATTAATTTTTTGTCTTTATTGTTATTATTATTTTAATTATAATATATACAAATAATATTTAGGACATAATTGACAAAGGACATTTTCGTCTATTCTGATTCTAAAATAAAGAAAATGTTATTAATTGTGATTTCAATTTCTAAAGTTCAAGTAAAATAATTTATTCTGATTCAAGTTCCTTATTATGATTCCAGTTCATTTCTATTTATGTTTAGTAAATGCACAATTAAAGTGTGTTTGGAACATAGTATTATCTAGTATTATACTATTAGAGTCAGCTTTTAAAACTTAAATTAGATTCACAACGATGTGAGAGGAAAGAGGGGTTTCTTTGTTTACTTATAATTGGAAACATTGTTTATTTAGAAAAGAGTATTTTCGGTATTATAATTGAAAAAGGGGAGCCCCTATGTTACAATTAATGTAACATAAACACTCAACAACAACCACACAAATAATGGGCCCACACAATTTGTGTGGTTGTTGTTGAGTGTGTATGTTA

>Cs1g16030

CCTCTTTAAATTGGGACCTTTTATTTCCACTTTAAAGATTTTACGAATTAAATATCAAATTGCTATATAGGCGCAACCTCAAGTCGAAGTATAATTTGCACGTTTTGTAAATACAAAGTCAGCGTAGAGAGCGAATTTAGTTTTGTTTCTTAAAAATAAATATAACAGTATAAATTTATAGATATTTACAAAAAAAAATCAATTGATTCTTTTGGATCAATTTTGTGATGCCGAATCACAATTGTAGAAAAATAAAATGAATGAAAAGCCCCGTCAATCCAAGAGTCAAAGCTATAAGTTTTTTCACCCTGTCTATGGGGAAAAAAAAAATCAATTGAATCTTTTGGATCAATTTTGTGATGGCACTATTCAAATATTCAAGCTTAAATTAAATCAACTTTACTTTCAAATACTAGATAATAAAAGAAACTCGAACCCCCTTCTAACATTTGGGTTCAAGTTCCATTATATAAAAAAAATTTATAAAAACCACATGAATATGACTCAGAAGAAAATGATATAATAATAATAATATTACAGATACAAATAATTGTAAGTTAGGAGATGAAATGATTAGTTGAAAAGGAAAAGAAGTTAACAAAATTATCATGTGGACCTATGATTTTTTTTTTTATTATGCTAATATCTTCGTGATATTTAAATCGAAGAATCTGATTCGATGGCCGCATAACATATGGCATTATAGTATTAAGTAAAAAAATATTACTTTTATAGTTACTTTTATTATTATTATGTCAATATTTAGACTGTTATTTTCCTCTTTTCATATATATTACGTTTTATTGTCAACTCAATCCGTACAATTTTTGTGATACTATCATAATTGAAAATAAAATTTCTAAAAGTAGCTTTTTTTTTTTTTTTTTCAGTGTCGTGTAGTTGCGACTTGCATGGCATTCGTCTTCCTTAAATATCCTTGCTTTTTATTATATTTTGTGTGATAAAATAATACAAGTAAAGATAAATATGGAAGTATCAAT

>Cs1g21210

TTTTTTTTTGCTTCAAACGGTAGGGTTCCATTAGGTTTGTCAAGATCGAATTAACATAAATGGATGACTTAAGCCGTATGGCTTTAGCCAATTTAATTTACAAATATACATTCTGGTGCAAACTCTTGTGCGGCAAAATCACATCTTTTAATTTATTTCCAACTTGAGATAAACGTTTGACTTCAAAATTCAAATCAAAAGCTTGCTCGCCTGCTTGCTTTCTACGGTCAATCAACACAAACAAACATTAAGTGAAAAAATAAAAATAAAAACAATTGCAAGAGAGAGAAAATGGCCGACATGGGCTAGATGGGCCACGTAGCACGGGGAGCATCTGGACCAGGGTGCATAGTAGAGGGTCCAAATTAACATGGAATTTGTGGCCTTGTGGGTCACGCTAGCCACTTTTGATGAGATTTGCTGTCGGTTTGGCTTGGTAAACACGACTACAATATCTTTTTCTGCCAAAACATTAAAAAAAATTTAAAAAGAGGTTAATTTTTCTTCGTTTTTCACTTACCACAACACTCGAAAACGTTATGGCACATGAGTGATGCACAATTACTGTAATCATTCCACGCGACTTTGAAGCTAGTGAAGCGTACTTATTAATTGGATAAAAACTTGGAGTATAGAAGTGTGTGATTGTTTGGAGGGACGGTGGATAAAGCGCGTATAATTATCAATTTCCCTAATAATGTAAATTTTTAGCAAGGTAAAAAAATCCTGTCTGGATGGTCAAAAGTTTCTTTGTCAAGCTGTCGTGGCATTCTTTACTTACAAAACAGCTATATATATGCCCACATTGTAATGGACTTCTCCAATACAATCCTCCAAATCAAGAGTCTTCAAACTTCAAATTAAATTGAGACTTCATAATCTTTTCTCTCTTTCAATTCCCTCTCAAAAGTTCTTCCGGCACGTTCTCGCTCTCTGTCTTTTGCTTTGGCACTAGATTCTATATTGTACATCGTGAAACACTTGTGGACACACTGATTAAA

>Cs1g25340

ACTACTAGTAGTACAAATAGCCTGCTCTTGTAGTCTTTGAAGGCACATTTGACTTCAATACTTTTTTCAGCTTTCAAGACTCTATGTGAGTTTTTTAGCCTAGTTAATTACTGGCATTATATGTAGAATCAACGAGTCTTTATCCTTAGGTTTATGGTATTCTTTCGAATAAACATAAATTCAGACAATTCCTTTATTGTAGTATTATCAGGATTTAACGGAATACTAGAACATTAAACTTCCGCTACATAGTTTGGTATCAGAGCAAAAGCCTAGTTCTTAAATGTCCTTAGTTCTTAAATGTCCTTAACACCTACTCATTTTTTCCCCTAATCCTATTTAGCTTTAAAAAAAAAAGGATGTTTCAGCCTCCACGTTGGCCGCCATCCATGGCCACCGATATTGATTTTTTGCCTCATGTCCGTCGATCTACAACTCCATCAGAGACAGAGAAAGCAAGCCAACAAAACCTAGCCATTTCCAACACTTTTCCACCGCTGCAATCAGCTAAAAACCACCAAAAACTGTACATCATCATTACCTTCGTTCAATGGATTTTGCTGCTCATTAGCTAACACCACCACCATCAATCAACTCCTTTATACCAACACAACATTTCTAATTATTCACCAGTGTATCGGTCATCAAAATAAAAACCAACAAAAAGGTTGCCACTCATTTCAACTTTCCAACCAACCATTGTTTTTTTTTTTAAACAAACTACCACCATTATAATATCATTGACCTTAAAGCTTTTAGATCTACCCATCTTTGCCTGATTTCCAATCACCATCGCTGGTAACCTAAACTTTTGCTTAATTAATATTTTATCCAATAATTTTACTCAATTTTCAGTTTCACCCACAATTTTTTCAAACCCACCCTCTCTTGCAATTTTCAATTAACCTTACCATATTAAGAACATGCCTCCAAGAAAAGCCCATTGGTAATATGCCCTTCATGCAGCAACTTATGATCAGGATAATGTGTAAGACGATGCC

>Cs1g18880

ATAAATCACCTACAGTAATGAAAAATATAGTGTCACTAATTGTATTTTTAAACGTCTAACTTGATCATTTTGAATTACATTTATGATGTGTTTAATCTAAATTTAAGATAAATATTTTACTTAAAAAAGCATACAATTTTACGTGATGATAATAATTTTGACCGTTAAAACCTAATATGACACACTTCCTATGATTAACGTATGACGATTTATTTTGTGGCATGAGAAATATAATGCGAGCAATTTTTTGTCTTCAATTATTAAAATTTTGGTGTAAAATATCATTTTTTATAATATATAATTATATTGGGACGACCCAGCTCACTTGAAAGTGGGAGGACTATCGTGGGGCTCCGATCAGAGGCTCATAAACTTTGTTCATTCTTTTTAGGGACCGCTCTGTCAAGGAACATTCATTGAGGACCGTTCGTCGTATCCATTCAAGTAAGGGCGAAAGTCATTTGGAGAGACAAATTATTGAGTGTTTATCTGAACGACTTTTTTGGTATAAACAGGAAGCAGGTGTTGGTGTTTACTATTTTGATTTAATGGGTCATCTCCATCTCCATCCAAATGTTAGAAGGAGAAAAAAACAAATAATAAACACTACTAGACTTGTTGGCAATCTAACTATCAGAATGGTGACTTTAAAATGTTAAATTGAATGCTTGTATTAATGACTAAAATAGGTAGCCAAACCCCAATGAGTGGAATTGTAGAAATGAACCTCACCATACATGATGATTTGGAAAAATACTCTATCAGCTAAAGAATATAATTAAAGTTATCTTACTTACTCGGAATATATACCACTCTGATGTACACAACTATAGAGCTTTTAACCCAAGTACTTCAAGCATACTGACTTTATGGAGGCAAAATTGGGGTCAAATCCCTCCTTTGTTTGGAGAAGTATATTATGAGGAAGACAAGTGTTGCACAAAGGCTTAAGATGGAGAATAGGTAATGGAAACCGGGTCTTAGTGTCCAGCAGGAATTGG

>Cs1g09830

TGAAATTTTTCTGTCATTTAGTTCAGTTTAGATTGAAAAGAACCGAAAATTTTTGAAATTTTTTTCTCCTTTTCTTTTTTTTTTTTTTAAATTCTAAAATAGAATAGAATTGATTTGGACTAACCTAAAATTTTTGTTGATGATGGCAGTGGATCAAAATGTTGGATTTATTATTTCGTATTGAACTTTTGTCTCTCATTTATTTTGAGTTTAGATCGAAAAGAACTAAAAGAAAAAAATTGTCTCAATTTTTTTTAATCAAATAGATTTGAATCATACTATATTAAAATTTTCAATTCTTTGTTATCATTTAATTTTATTAGACTTTCTTAGGGTATAGGCATTCAGTTTGGTTTTCCCAAAACCAAATCGAAATATGTTGGTTGGTTCAATTCAGGCAATAAGAACGGAATTGTTCAAAAAAATATGAACCAATTGATTTTTTGGTTTAAAACGAATAGAACCAAAAAATCAATCAGATCAATATCAAATTAAAAAATAATTTATTTTTATATTAATATTTACATGAAACATAAATGATTAGTGATTACAATTGAATTAGAAAAAAAAATGATACAAGTAAAAAGTCTAAAAAATTTTCCAACATAAAAAAAATATTGAATTTCTTGAACCGAATCATATAAACCGTACCAAATCCAACTAAACTTAAGGAACCAAACCAAATGATTTAGGTTAAATTCCTAAGTTCGGTTCGCATTCGATCATAAAATTTTCAAACTGATTAAGTTAATGCCAACCCCTAAACTTTTTCTTTTTCTTTTTCTTTTTAAAATAGAGAAAGAGACACTCGTGTTGAAATAGATGATGTCAACACGGAAAAAGAATTATACTGTCAAAACAACTTCTTTTCTAAAAAATTATTAATTCGTAACGTATCAAGTATCAACGTCACTCTTCAAACAAAATAAGTACTAATAATAACTTAATGAAATTTGATCAAACAAAAGGTCTCTGCATGAAATTTGCCCATTAATACGAGT

>Cs1g04235

ATGCAGACATGGATTTTTAGACTCAAAATTGTGAAAAGTATGTAGAAGTTGAATGACACTAGGTTTGAAATTAAAACATGATGGATTAGGAGAAAAAAATTATGCATGATGTTGCTTCTATTCTAGTAAGATTCATGAAATCTCTAAGTGTCCTAGGTTGATCATGAATATTTTGTAGTGCTTGGTTATCCCCTTGCACTGAAAAGTTATCAACTTCGTTGTCTATCATGTCTAAGGGTAGTGAAGGTGATCTTCTAATTAATCTGTCACCATTAGTACGAGACCAACATCTCATACAATGCTAATGATGTGTGAGTGTGTGTCAAATTAAGTGAAGACAATGATGTACAATCAAATGAGTAATGCATGAAATATGAAATAAAAATTGCACAAAATTAAAAAATGTAAACAAACTAAAAATTAAATAAGCAACAATAAGAATCTACAAAAATAAAAATAAAAAATAAAATGAAAATTATTAAAATAACTCAACAAGCGATATTGCAAAACTAAGAATTTAAATTAATTAACTAAACATAAAATAATAACACATCCACAAAATAACAATGTAGGCGGTATTATAAAATCAGTCATTAAAAAAATAAAATTTTTTAAAAAAAGAAATTATAACGAGAAATAGAAGGATACACTTACCTCAAGATGTAAAATTGACTTTTAAAAATTATCAACCAATTAAGCAATCAATTTAAATTTAATCCTTAATTATATGTTTCAAAGTGGAAAATCCAAGTCCATTTAGTCCACAATCTCTAAAAAGTTCAAGTTCATTTAGCCCACAATGTAATATACGCACTTGGACTTTCTTAATAAAGTGCCGATTTTTATTTTCCTCTTCTCTATTAATTTTTAATTTCGTCACTATAGTTTAATTGGGATAAATTGAGCTCGATTACACGCGTCGATGGCCGGATTTTATTTTTATCACGGGTCCGAATTGAGCTTAATTTGTATTAAGCTTAAAAAAAAAAAAGGGGGCAGAA

>Cs1g06350

ATAAGTTCATGTTTACATGAAATAGGTTCTTGTTTTAGTTCATTTTCTTTCTGGATATATGGGAATTTTCATTTTCTTTCCTGTTATATAGTTTGGCGACACATAAATTAGCTTTGTTCTGTGCATTTGAACTTTTTTCGATCTGCATCAAGCCTTATTGAACTGCCATTCTGTAGTGCCCAATGTCTGTTCCAAGCCAAGTTGATTGATTATACAACATTAGACCAATTACTGCAATGCTGAAAATTATTCGTGCCTTATTGAGAATATATTTTAATAACCGAAAAACAAGAACTTTCTGAAAATTTCTTTATCCGTAATGTTTATGGAACTTTCAGATTCTTTTAATTATCCCACGAAGAGTCGAACTTTCAGATTCTTTTAATCATCCTTCCAAGAGTAGAGCTTCAAGTCACATGCAATATATGTTATCATCAGATTTTTGGGTACCACGAGTTTGTTTTGGTTGTTGTAGCTGAAGAGCTCTGGTAGGTGTAGGTGCACTGATGATCTACGTGCTATTTATTTATTTTTGATTAATTTAAGTGTAAACAAGATGAAAAATTTCTAGGCAGCAAGCAATTGATTCGCCTGGTTGAATCACGGTGTACCTTTTTTTTTTTTGGCCCAAATTTCTTAAAATAAAAAATTACTGTTAGCTTAAATGCTGTTAAGTTTCTCCCAATCACAGACATAGTAGATATAAATTCTTAGTTTTTCAAGTTAAAATTATCCCTTTTGCAAGTTAAATATTTTTACTTTGAATTGCTTTTTCCAATTAGTTTACTGTTTTGTCTCATGGGAACTTTGTCTCAATTTTATAGTTCATCAGCCATCTTTTAGTCTTCACTTTGAACATAAGAACTGTGTCTGTGTCTAGTAGTACACTGTCAATTGCTAATATAGTCTTTGAATATGCAGAGAGAGATAGCTAACAGAGAGGGATATCAACTGCTTACAAACATTTTCAATTAGCTATTAGCTACTCAGTACTGACACTA

>Cs1g23580

TTTCAAACTCACAGCAGCCGTCATAGCATAGGTTACCAAACACCAAGCTACTTTTTTAATCAGCAGCTTATTTCATCTGTACAGTACAACCAGCTTATTTCATCAACCTCTGCAATCTCAAACTGGCCCTAAATATTGAAGAGAGAGAAGCACTATCTCATTTTAAAAATCTTATTTAGAGAGTTTTTTAAGAACTCTTTTTATTAATATAATTTTTAAATAATTAGTAAAATCTCATTTGAAGAGTTTTTAATAATAGCTTCGGGTGTAAATTTATATTTAGCTTTGTATAATACTATAATATGACTCGACTAATTTCTCGAATAATATGATATTTGGGAATCTGTTTCAACGTAACTACGGAACTTCACTGCATGCATCATGGTCCAAAGTCAAACTTTTTGGCTGCAGTTGAGTTTTGAGGACATTTTCCACCTATATGGTAGAGGTAGACAGCAATAGCAGCGGTACCAATTATGAAAGATATATCTGGGCAATTTACCTATTTGGGTTGGAAATTGTCTTATTGTCATCAATATCCAATTTATATATGTAGATACAATTCACCAGTGTAGAAGCACTTGCTGTACTGTTTTTTTTTTTTTTTTTTACATGCATTTCAAATTCCGTGTCTAATTATTTATTTATCAGTTGAATGTTAAGGCTGTATCTGCACGTCCGATTTCTCAATTTCGTTTATTTTCGGAATATTCTTTCATAACTATATATAAAGAGAAAAATAAAAAAATCATTTTCTGAATACGGACTTAATCAAACACATAGATAACACATAGATTTATGTGCTCGTTCAAACATGTTATATCATGATGGGAGAGGGTCAACGGTGCTGTCCCCAACTCCCCGTCATCCTACTCTGTAAGAATGATTCTTCTTTGAAATAGAATTCCATGACCATGACTGGCCGCTAAATGATACCCTGGATATTTCCTTTTAAGCAACGTTTTGGCGTACCCCCGTTTGATCTTTCTTCTAATTTCATC

>Cs1g07690

GACAAAGCCTTCTCATATTGCCCACCCCAGAGGAGAGAAACAACAGAAGTGGTTCCCTCCCCCTGAAAATATTTTCAAAATTAATGTGGATGTTGCCATCAATACTAAGAATCAGATTGCAGGCGTGGGAGCAGTGATTAGAGATTCTAATGGAAAGATAATTGCAGCCGGTATTAATCAAATTCATCTTAAGGGACCAGTCAGTTTAGCTGAGGCAGAGACTGTGCAATGGGGGCTTCAGTTGGCAAAAGAAGCAGATTTAACTTCCTTGATCATTAAGTCAGATTGCTTAGAGGTGGTTCAACTTGTAAACAACACCAAGAGCAGCAAAACAGAAATTTTTTGGACAATTCTGGAGATCCGAAATCAGTTGAAAGTTTTTCAAAAAGTTGTTCATCACATAACAAGTCAATGTAATGCTTATGCCCACTCTCTAGCTAAGTTAGCTTTGGGAAGAAATTCTTCTTCTATGTGGCTAGGAACTATTCCAGTCGAGATTCAAGTTGTATTTGAGGTGTTGTGATTTATGAAAGATTTACTTTCTTTTCAAAAAAATAAAAGTATTGGATATGCCTATTTATCCTCTAATAATTACTTTAAATACTTTATTGTTCTACACACTCATCTATTAAATTTAAAAAATTAAGTTATACTTTGTATGGTCTTTAAAATACTAAAAATCCTTAAGCATGTTAAATTAATCACATTTATTTACTCAAAACAATACTCAAAGAAAATTCTTTAAAACAATTAAGCATATAATACTGAAGTTTTTATTGAAAAATCGACAAATCCTAATATAATTTATTTCTATCCCCTACCTCTTCGAAATTTAAACTTGGAATGATTAAGAGAATAATAATAATAATAAATTGTTTAGAAAACCACTAACCAAGGCCTATATCATCATATCATATCATATGAATTTTGTTACAAATTAGTTATTAATATTTTGATAATTTAGTGACCCTCGTTCAACCGAAATTGGATAACTTTTTGCA

>Cs1g18000

TAATTTTCTTAACATGGTACTAAATTAGGTTTTGTGTATGTTTTTTCACTTACAAAATCGTTTCAACTTTTAACTTACTGTTTTGATCTCCGGGATCTAAGAGACTTTAGAATTAGATTTGTTTCACATTATAAGAGGGGTAGTGCTATATATTTCATTTTTTTTTATCCCAAATAATTAAGGCATTAATTAATTAATTTATTTCTTGGGATAATACAATTAATTCATTGGATTTTTGTAAAGAGTTATCAACTAATTAATGAATACCACATCATTTGAGATAAAAAATTTAAGATGCCTATCATTACTCTTATAAGGGAGAGTTGTTTTTGTTGGCACTCTGGTTTGATCTCTCTAGACCCACACGAGTTATTCGAAATCAAATATTCTATTATTTATATAAAAGGAGAGAATATTTACCTATTAACTTAAGTTTTGAGATTAAAGATTTCTTTAATAAAATTTGATATCAGTATATGCACGCTCTGTAAGAAAAAAGATAATTTTAAATGAATTTCACTTGGGACATAGAAAAACTGTTTCATAAATGATTATTGCAACACAATAGTATATAAAGAACCAAGAGGGAAATAGGGATTTAGATTTTAGAATCCAAGTAAAAACCCTTTCAATGGAAAAGAAAAAGAAAGAATCCAAGTAAAATCTGTAAAGAATGACAGTAACTACTTCTGTTGTATGAGTACACTTGTCGAAATCTCAAGCATCCAAATATAATCCTATTTTTGGCAAAAGTGAAAAAGAGACAGAACATGAGCGGGTAAATATGAGTTAAGGTTTGAAGAACTTGCCACGTACTTATTGTTATTTTTATTTTTTCAATCACATTGGTTCTCATTTCCTCCTAATAACGAAAATATTTAATTGAGTTTCTGATATTCCAGGTAATTAATTAATTAAGTGGAAAAAAAATCCAATGAGAAAAATGAGTGTAAAAATATAATAAATAATAAATAAATGAAGAGAAGGACCCCCGGAAATTC

>Cs1g13460

CTAAAAATAATAATTATGACAATAATGTTAATTAAAACAAAAAATTAATAAAATTTAACAACAATAACAACAACAACAACAACAAAATTAACTGAAATAATAATATTAATAATGATAATAATAAAAATAATAAAAATTATTAATAAGTTTTAATAATAATATTATTAATAAAAATAATAACAACAATAATTAATAATATTTAAAATAAAAAAGTTAATATATTTTAATAATAACATTAATTTTAATCATAACAAGATAAATATTAATATTAATGAATTAATTACGGTATAATAACAATTAAAATAATACTGCATGACTTTTCAACATAAGCTCATCAATTGTAAAACCAAGTTAATTAACTAACATTGTAGTGCTCATACATTCTCATCACATTTCTTCATTTCGTAAATAGTTGAATATTGAATTCTATACAAACTATGGGAAACGTTTGGTAGCCATTTGAAATTTTGCATGCATTTTTGAATTTCAATATCACGTGCATGACTTTTCAACATAAGCTCATCAATTGTAAAACCAAGTTAATTAACTAACATTGTAGTGCTCATACATTCTCATCACATTTCTTCATTTCGTAAATAGTTGAATATTGAATTCTATACAAACTATGGGAAACGTTTGGTAGCCATTTGAAATTTTGCATGCATTTTTGAATTTCAATATCACGTGCATGACTTTTCAACATAAGCTCATCAATTGTAAAACCAAGTTAATTAACTAACATTGTAGTGCTCATACATTCTCATCACATTTCTTCATTTCGTAAATAGTTGAATATTGAATTCTATACAAACTATGGGAAACGTTTGGTAGCCATTTGAAATTTTGCATGCATTTTTGAATTTCAATATCACGTGCATGACTTTTCAACATAAGCTCATCAATTGTAAAACCAAGTTAATTAACTAACATTGTAGTGCTCATACATTCTCATCACATTTCTTCATCTGTTTAATAGCTGAATATTGAATTCTATACAAACT

>Cs1g08380

ATTATTTTAAATAAATAATTGAATAATTTTTTATGTTTAATATTACATACTAATTTAGATAAATTATGGAATTAAAAATTTGATATTCATTCTTAATTATATATAAGTTAAAATAAATTATAAAATTAAAATTTTCATATTTAATTTTGAAAGAAATACAAATTAAAATAAATTATTGAATTAAATTTTAATGTTTACTTTAAGATTAAATACAAGTGAAAGTAATTATTGAACAAAATTTTAATATTTGTGGCAGATTTATATTATAATAAATTATTTAATCCTACTTAAATTCATGTAGAAATGAGCACTATTAGGGCATCTCCAAAATTTTTTATTAATTTTACTTTTTAAATATCTATTTACTTATTTATGTCGTAAAAAAGAGAGTGAAAAAATCTATTATTTTTCAAAATACTCTTTAAATAAAAATATATTAATATTATTTTAATTAAATAAATATTTTTTAAAAGAAAAATAAATAGTAATTAAAACACTTCTTTCTTTCTCCAATAAAAAGTAATAAAATATAAATTAAAAAGGGAGAAAATAACTCTATAAAATTGAAGAAAGATAATTATTTCTTATTTGAAAAATTTTAATTGAAGTATTTTTTGGATCTTAAATATTGATTGACTCATTGACTCTTTAAATAAGATTTATTACTTATTTGAAGAATTTTTTGAAAATACTCTTAGTTAAATTCGGATTATATAATAAAATATTTAATCCTTGTGAAAATAAGAATTAATAGCGTCATGAATTTTGAACTAAAATTTGAATTAATTATTATAGTGACAATTAATAATAATAAAGAAAATCGAAAAATCATGGTATCGTAATCGTTTCAGAGATTGTTGTCTCATGTCTGTATCCCATAATTTACGCAATGCTGACTTCAACACTTCATCTACTCGTCTTCTTTAGTTTCTATAAATAGCACAGTACAGTGAAGCCAAGAGGAGCTGTACTGCACTTCACTCACTAAAACTCTGTAAAAG

>Cs1g22230

ATAATTACCGATCTAGTTCAATATAGGCAACTAATCTCCTGGATCCGTAAAGCATCTTTTGCAATCTCCTTAAACCTCCTCGCCTCTTCCTCATCCTCCACAGGGTCGCCGTAGTACCGCTTCCCTGCAACCATTCTCAACATAATATTAGAAGTAAACTCCAAAAACAATGTTTTCAGCTCCATCTTCGGAAACTCTTGGCGTCAAGAAGTCGACAGTTTTTTCAATAACCGATTGATTTCATCTCTTCTAATTGATATAAACACGTTAAGACGATTTGATGAGAATACCCCCGATAAAAAGACTATTATTTTAATATCCATCATTAATCATCTTAACAGTTGGCAGTATAATTTAATTATACTTACTATTAGTATTTTAAATTCCAGGTAAATCAGTCCTTTTATTTATTAATATTTTAATTAGTCTTTTACAGGTCAACGTATGCAATGAGCCTTGATTTTTTTTTTATAAAAAAAGCAAAGGTGTAATTAATTTTTTAAAGGTCAACTAATAGTCTTTAACGAAGAAATAATTAAATCTAAGAATTTGTAATTATTTAGTAGTAAAATGTTAAGGCACTGTAGTTGTAATTAAATGTTATTCCGCAATAATTTTAGTAGTAAAAAGAAAATAAATTTAATAATTTATCATATGTAGAAATGTTATTATTTTATTGATTTGTGTCCCAATTATGTTCCTGCTTTGGTACAGCTATAATATATCATTTTTGAGTTTTTATTTTACAGTATTAAATTCTTTGTGGTCATTGCAAGTGTTTAGAAATAGGAAAATGATAACTAAACTAAAGAACGATAAAATAAAAACAAAAACGGCAGCATATTACTACCGTTTAATGGTTTATAATTTGCTTCTCCTTGGGCCCACGGAGAGGACAACAAAGAGAGGAACCGTTTCTGTGCATTATTTTGTCGTACAGCGACTGCCACGTGCATTATTTTTTATTGTATATTTTGCTACGGATAAGTGTTTGACGACAG

>Cs1g23990

GTAAAACAAGTAAATTAATATAAGAGCTATTGAAAAAAAATAAAAAATTACAAGTGTAAAATAAAAACTCATTATAATAATGGTTACCTTATAAATTTCCTGATTTTTTTCTAAATTAAATAATAATAGTTTTAGGTTTACATAATACATGTGGTAATATATGTCAAATTTTATTAAAAAAAATTAATCTCAAAATTAAATTTTGAAAAACTACTATTCTCATGTTTTCAACAGATGTTTGTCGGGTGATTTTGTATCCTTCTAAACTTCTTCGACAACAATCTCAAATGATATTTAAGTATAAGTAAAAAGTAAAGTTTCAAATTACATAAAGTATGTAAAAAAGCAAAAAATAAATATAAAATCGTATATGTCTAGTAATATAAGAATTTCTTTTTCTTTTTCTTTTTTTGGTCTTGATCCACATCAAGAAAAATAATTTATTTAATTAACATGTGTAGTAAATAAACACTATGATAGAAAATGAAAAAAAAAAAGTTATAAATATCCTATAATGAACATAGAATTTATGGTTAAACCAATTTAATAAAGTTATTCCAATTGCTTCAATACGATTTTTTGTTTTCAATTGATGAGTTTTCTTTGTTTTATTAAATTAAATGAATAGTTTTTTTTTGTTAAATTAAAGTTTAACATTGATATACAAGATTTATATTATTTTTATAAGTGTATGTTTTGTTTTACAATTTTAAATATCAATTTACTACTCTTGTATTAATCTATTTATTTTACCTTATATTTACTAGACTTATATATAATTTATAAAATTTATATTTGTCACAATATTAATACCATATTTTTAATATAGGCATCGTAGAATTTTCCTACATGGTTATAGAGTTCTTTTTTCCTATTTCTCAAGGTTAACCAAAAAGAGAAAAATAAATATAAAAAAGAAACAGAAGAAAAAAGTAAACAAAGATTTCGAGCCCCCTTTAATTTGAAAATTAGCGATCCATATCATAGAGAGAGAAAGAGAA

>Cs1g06740

GCCTGATGGGCATTCCAATTTCAGACAATGAATCATTGACACGATTTTTTTAATTATTTTTTTTATTTTCTTTCATACTTTCAACCATAAATAAATAAATGGTCTAAATTATATATTTGGGTAAATTCTACATTAATAATTTAATTCATTAGACATAATTGTAAGTATATCTAGCGAGTAACAAATATTTACTATTTTTAATTATTTTAAGAATTTCAAATTTATATTTAAATAAATTAATCTCATTTTTTTAATCATATTAGTTCTTTAGAATAAAACCAATTGAGCTACCAATAATTCGGTTGATAATAAGCATAATGTGTGCTTAAATATTTTGTTATATAATATGAAATGTATTCGCATATTTGCACCAATGATAGTTTTTCTTCTTACACACAAAATTATTGTCTTTTTAAAAGTAGTTATTAAACATGCAATTAAGTTATAATTGATTAAGTTGTTTACCTTTTCGTCTGTATTAAAAATTAAATAAAAAATGAATATAAGCTTTTTAAACCTTTTACCGTCATTCAAGGATATATATGATTATTAATATAAACAGTATTATTAGTAGGAAAATTTGAAATATAAAAATCACAATTTGTAATATTCCGAATAAATTTCTCTCACTTCTTTTCTTTCAATTATATAAAATTAAATGTAATATTAAAAAAATTGTATAAAATCAAATAAATAAAACTTGAGAAAGTTACCGAACCGGCGTCGTGTCCCCCGGCACGTGATTTAATAAAAGAATTTTGTTTTATGAAGCAAGTCATTGGCTTTCCTTATGCGGACTGCATTTGTTATCTGAAATCATCATCGAATACCACAGAGACAATGATCTCATGTTTCATACGCAAAATAATAATCCACAACCAATATACGGTACTCATTCAACATCTAAGTCAACGTCAAATGAGAGAATCTAAAACATCATCTTAACCGTCCATTTCGTGATTTGGATCAAAAAATAAAACTCTATCATACACTATAAAATA

>Cs1g18190

CTGCTTTTATTTTGGGCAGATGATGGGGCAACCGAGTAATTTATAGGACAATGATGCATCATCTACCTAATTGAATTCCACATGATTTTAATAAAAGACATCGTTTGATTTTAACAATTAAATAAAATAATAAATGACTTTAATTATTCATATATTTTTACATATTTGTTTTCTCCAAGACGAAAAATAAGTTATTTGTATATTTGTATCAAAATTAAGGGAAAAACTTTAATTTAAAAATCACACTAAAGTGCGCATCTTTGTTGAAAATATATATTATATACTATAGTCCGGGCTTTATATAGAAATATAAAAATATGATCTCTTGTTATTTATTTCTACTTTACAGGGCAACGCATCATCTTTTCTTTAATTTCACATGATTTTATTAAAAATAAGAGATTTTTTTTTCTGTTTTAACAATTACATGAAATGATAATCTATTTTAGTTGTTCATATAATTTTACATGTTTTTTTTTTCCTGGCACTGAACGTGTGTTATTTGTATATCTGGTAAAATTAAGATAAAAACTTTAATTTAAAGATTATGTCTATCCTTTGTCTCGTTGGCTGAATACAACAACCAGCATTCAATATTGTCCCAAAAAAAAAAAAAAAACAACAACAACCAGCATTGAACCAATAAAAAATTCATATAACTGTTGCTTTATCTAAGCCGCCAATTTTATTTTTATTTTTTCGTTATTGAAAACAATTCGGTTTCAGTTTTAAGTTGTTTCATGTGGTATTTGTTTTTGTTTAAATGTAAAATTATCTTAATAAAATCAATACTTTTTTTTTACTACTCACTGAATTTGGATGTATTTAATATTTACTTAACTTGCACCATAAACCATATTATTTTACCTTAATAAAATCTTACTTGTCGATTGGCTGACAGAGTGATGACAAAATATTGAGCCCAATTTACTATTTTCAAATAATAAGAGGGATATATGAGATTTTGTGTACCATAATTTCTATCAGCGTCTCCTTTTATT

>Cs1g16430

AATATCTGCTATAATAATTGTTGTAAATATTTATATAATACAGTAATATGACGTGTAATCCGTATTAAAAAAATAAAAAATAAAAGCTGTCCGCTCACGGGTCACGTCGCTATCCAAAATTGTAAGCGCAAAAAGTGACCTCATGACAGCTTAACTTGGCGAAATAAACGTGATAACATGTAGCCTCTATCGTGCACGTATTTTACGCGCACTATCACTCACGTGTATTTGTTTCAGAAAAAGTTTGAGTTAAAAAAAAAAAAAAAAGCTGCATGGCTCTACTGAACTTTCAGAAGTTTTTGTCCCATGTATCACGTGTGATTTACGTGTTTATTATTGATAATGTATGATTAAAAATAAATTATTTTTATAATTTTATTTATTAATCACTAATTACTCATGTGAATTTTTAATTACTAATAAAAAAAATTAAAAAACATATGCTTCAATTTGGCAGGCCAAAGCTAAAACTCAATGTTTTGAGTTTGATCTATTAGAGCATAATTCGAGCATATATTCTTTCAAGCAAAGAAGTTTTTTTTGGTGAATTTTTCTATTTAAAAAAAATGCTGATATTAGTCTGTTAATTGGGAGAATGACGCTCTAGTATCCATCTGATATCCTAATTTTACCACTTTAGTTCATCATAGATATTTTAATACTCCATTTCATCCATTGTGTTCAAATTATATATAATAAAATAGTTCATTGTTGAAAAAGGTGTTACTTTTTAATAGATAAATGAACTAATTCTAATTTATGAGATCTTAGCTAATCAATTTAAAAGTGAAAATGTCTCGTAATATATATTTTATCAGTATGTTTTTTATGTTGTAGTTATAACCCCAAGATGAAATATTGTATTAAAAATCTATCAGGGACTAAAATGATAAAAAATAAAAGGACTAAATTGTCATTCTTCCCCATTAATTTAATTAAACATTGCCATGCCAGCCAACCAGCTCCAGCTATATATAAGTGTGTGCTGGTAGCCTCAGTTT

>Cs1g17050

TTTAAAGGCAAATTAAAAGTTTGTCGTGTACTTAACTTTTAGACTTTGAAATTCCATAGACACTCAAATTTGTCGTCCACGTGTTCCTCCCCCACATTAGTCGGCTGCAGCGAGCAAAGATTTCTAAATTATTACTCATATAGAAATTTACGCATGTTATAATTTAATAATTTTTAGGTGCGAACATCCACATATTATACACTTAATGCTTTAAAATATTGTAATAGATTTTGATAATATAACATTATAAAATCTTTTTACAACACCGACGTTAATATTCATATTTTGATAAGCAAGTTTACACTAAAGAATGACTCTTATACAATTATATAATTTAAACACTCAAATTATAAATACAGTGTGACAAAAAAAATTATTAGAAGATAAAGACGGAGAAATCAATATTAAATCTAAATTTCTAACGATTTCTCCAATGACCATAGGCACCAGGTGCTCTCAGGCTCATTCGAACTATGCCCGAGTGAAGTTAGTAATGTCGAAGTAATTGTAAGTAACTTGCAATACAATAAAGGATATTTTAATATTTCTATTTTCTTGTGAAAACTTTGTAACGCAATTCTTTTTTTAATCGAAGCAGCATTGTTAATTAGATAATAATAAAAAGGGAGCTGCTACCTTACATGCACGTAAGGTACAGCCTCTCACAAAACTCAGATGTGGATTTCACTTGGGCCCACCAGCGAGAGGCTGTACCTTACGTGCATGTAAGGTAGCAAAAGCCTAATAAAAAATCTAATCAATCAAAACAATCCGCCGAAACGATTTAATTATATAATTGATCCATTTCTGCACAAGCTTCAAATTTTCAAATCCTCAACGGCCATATTTACTTTTGTTTTTGGTCGAAAATTTCCTTTCATTCTGGCAATTCGATTTCAAAAAGCGGAAGCAGGTTGTTACGGTTTTTACCTTAAAAGGTTTCTGCGGAAGAAAAATAAGAATAAATAAAACCAGAAGAAAGCAAAAGAGTAGCGGATCAA

>Cs1g15900

TCACTCTCTAAGTAATTATATTTGTATCCTTTTTTGTAAGTTTCCTTGGTCTGGCAAATAATTTGATACAACTTAATTCGATCCGATATAAATAAACTCAGGTTTGGATCGGATTGAATTACTTATTCAATTCAATCGGATGACAGTTTATGCATCCTGGACAGATTATGATCGGATCAAGTTAAATCCTATGGGGTTTGGTTGACATTAAAAAAAAAAAAAAGTTAAATCCAACGATCAAAGGAGTCACACTCACACGACACAGCACAACACTCACACCACACACACACACCCAATCTAAACCTAGTCCATCGTGACTCACTCGCGCGCTGCACTCACCAAACAGGCAAACGCACATAGAGATCGCAGATGGCGGGACGCCAAACGCATACGGCACGGCAGCACACACATTAGCCAGCAATCACAGACAGCACAACCGCGTTAAGCCAAGCCATCGGGAGTCCGCGATATGCCGACGCAATCTGCTGCAGCTCGACCGCTGCTGTTGCTTGCTCCACTGACCACCGCATTTATCTGAACGCGATTATTTTTTCATGGTATATTACTATTGAATTTTTTATCCGCGAATTATAAGTTCGTGTCGAGTTTCAATGATGTTTTTCCAAACCCGATTACTCCGAAAGCCGATTGCATTTATCTGAACGCAATTTTGCCCGCACCTAATTGAGTGTATAAGAACATACATCTACATGATTTTAAGGTTAGAAAATTGTCATGGTTCGATGTAACAGAATGGAGGCCCAAAAATTAATGGCAATGGACGATTACAAATAACTCCATCATCCAGGCAAGGTCTTTACTCTTAGACATCAAGCATGCGATGTAACAGAACTGAGTAATCCATTGTCATGGATACTGAATTTACCAGTGTTAACATTGTACGCTGGTCAGTTGTCCTATAAACCCCTACACGGTTTGTCGTCTGCGACTCCAAATACCAAATGTCTTGCTGTTTAAATAGGGTGGGTAAATCATATAAA

>Cs1g21430

GCATTTACCGTGGACAATACTTTACTCGTTTCAACTTTTTTCCTGGTAATTTCGGAATCTCTTTAATTTGCATGTATAAAATAAATCTAGTGGTTTCTTTTAATTAGTACGTACCTAGCTTGAAGAAATAAGAAACAGTTAGAAGTGGGGAATCATGGAGAGCTATTTGCAAGAGAACTTTGGGGTGAAACCAAAACACTCTTCAACGGAAGCGTTGGAGAAATGGAGAAATTTATGCGGAGTTGTCAAGAATCCGAAACGCAGGTTTCGGTTCACTGCCAACCTCTCCAAACGTTACGAGGCTGCCGCTATGCGCAAAACCAATCAGGTTCCTTCATACTTCACGATCGAAGTATTAATTGTCAACTTCTTATCATGCATGCGACTTCCCAATATTTTCATTCATATTTATGAACTTATTAAAGATCATTTGCTAATCATGATCGTTCATCATATTGGTCGTGCCATCTCATCCGGTGAGGCGTGAGGGCTGCAATAATATTTCTTTTAGTAAAACTAAATTGTCATTCTGATATAGTCATATCATGCACGATACCAAAAAAAGGAAAGGAAAAGAAATATATTCTCCTTCATCTTTGCCACTTTTTTAAAACTAGATTCTCTACCCTGCTAGCAAAAGATTTGTTTATGTATACAGTATATATATATATCCGGAGATGAATTATTTATTTATTCGTTTATGACTGGTGCTTAATTACAAATTCTATATTTCACAAATTCATCTCGCTTGATTATTTATAATTCCCCTGAACTTGAAATAATTAAGCAGCAAACTCTGCTTGAGATAAATGCCGAATGCATGAACAATCGTCAGTGGTAGAATGGGCATTCTGAGGGCTGACATGATCTCGATCTTTGCAAGTGACTCAACATATCATATCACACTTCTTGTAGAATCTAGATTCTTCTATTTAGCAACAATTCCCTGATGATGATGTAACTCATTTTTCAGAGCAGAGCAGTAGAGAGATCATTGCTTT

>Cs1g08480

TAACATAGAAAATTCATACTTTTTCATGAAAAAAACGTAACCATATACACGAATCCAACACAAAAACAAGCTCTAATATCAATGAAGGAAAATAAAAAGATTCAAGAGCCTACCTTTCTTCACAAATCAAGCAACGAAATAATCAAATGTTAGATTACGTCAATGGTCGGAAGGAAATCATAGATGGAAATCCTCCTCTTGATCACAGTATAAATGCCTTTTCAAAATGGGTTATTCAAATGTTCTTGGTTCAGTATTAAGAAGAAGAGAAGAAGTTGAAGTCGAAGAAAAATCTGTATTCGATTGGGAAGAACGTATTCGACTATTGGGGAAGTTGATCCAAAATCAACTTTCCTTTGAACAACTCCCAAAAACCGTACGTAGTTACCATGTATGGGTTGGGTTAGGTGATGGGTTGACCCACTTGTCCAACATTTCTAAACATATAGTAATATTAATGGACAAGGACACTCTCTTTATCTTTTTTTGCTCTAAGTCTCTCCTACTGAAATAAATTAGGGCAGATCAGTTCATGACCAATGAGAGCACAAGTTTGGCCAAAGAATATAAATTTTAAGAATTAAAGAGAAAATTACTTGAGTTTACAACTAATCCCCTTTAAATCTATTAAAATTACCTCAATTAAATCTACAAAAGTAATTCCACAAACCCATAAATTTTAATTTTTTACATCTTATTGTAATAGAATTAAACTCTTCATAAATCGTTTTTTTTTAATTTAAAAATAAGTAGTCAAGATGAAGTCTTAAATTTGATGCTGACAATTAAGTCGAAATATTTCACTTTATTTATAATTAAATAATTAAACTTATTTTTTCAACTGGATATAAGATATTGTGAGGAAATACATAAAAACAAACAAACAAACAAACAAACAAACAAGACAATCCCTAACATACATTTAATGTACGTTACTCAGTTACTCCACTCGCATGAACAGTGAGTGGAGGAATCCAAATGAGAAATGACTTGTTGGTATT

>Cs1g22910

AATGTCATTGTCGAGCGTTTCAGGCATCTTTGACAAATAAGTTGTTAAGATTAATTTGTTAGCTATGAACAAAATAAAATTTTCACATACGAGGGCGTATATCAATTTGTATATGCAATCAAAGCTTAATTTGTGTTAAGGGGAAACTGCATAAACATGGAAATATGGTAGAAAGTTAAGCGAAGAGGGAGCAATTAATATGCACATGAGTATATTGAACACCCCCCTCATCGTGTTGATATTAAGGCTCCTTTTTATATTACGGTGTTGAGGCTAAAAAGTTACCCTTTCTTGCGAAATCATTGAAATGATAACTTTAACAATGAAATAAAAACTTTAATTGCGGAAAAAAAAAGTTAATAGTAGTGGATAAATGTGATTCTTAAAGTTGACAGTTTAAAAAAATTAAAATATTAAAAATCTTTATCAAATAATTATATGAAAATTTAATTTAATTTTTAAACCATAATTAGAAGTATTTACTAAACTGAACACCAGTGATTGTTCTAACCTAAAATCTGAGAAAAATAAATAAACTTAAATTAGGAAAAACTTTGGTAAGAGAGAGTTGGGCAATTATGAATATGTGTGTGTATAGTCATTCTTTAGTAAGGGAATTCTCATTTTTATTGAGGATTCTAATAAAATAAAATAAATTTTCATATTTCAATATGCTAACAGATAAAAAATATAGTTTTTAATGTATAAAATTATATATTTTTTTTATATTTTAGACTCGTGTATGTATATGTATACATACATACATATACACACACAAATACGTATATAATCATTTTTAAATAAGAAAATTCTCATTTTAACTGAGAATATTAATAAAAAATAAAAAATCACATTTTAATATGCTAACAAATAAAAAATCTATTTTTTAATGTATAAAATTTTACATTTTTTAAAATATTTTAGTGATATAATTATTTTAACAAAATAAAAATATACTCATTAATGAATTACTCTCTCTATATATACATGTATAGGGAGGC

>Cs1g18220

CGAAAAGACTCGTTGACTAATACAATCCACCTCCTAAATATCTCACGCGATCGTCTTCTAATGAGGCCAAAAAGAAAATTCTCACAAACACAGCACCAACCCGACATGGGCAGAATTATTTATTTATATTTCAATAACCCTAATTTTGATTGATGTGCACGTTCAAATAATATTTCTTAGCTGTGGTTGGCTCGAATGGCGCCTGGGGCTGGGGACAAGCTAGCTCCGAAATGCATGCACATGATCAAGTAAATATTATCAACTTGACAATATATTAAATCCAACGAACGAACCGTATATATATGGACTGTGCATCTGTATAATGTTTAATTATAAATTTCACGGCCAATAATCAACCGGCTTACCTAGTTCTAAACTTAACAAGCATTGTTAATAAAAATTATTTAACATGTATTATTTTGGGATCAATCTGGCCTAGCTAGCTAGTTGCTTTTCAAAGTTAACAAGAAATTAATATGGCGCTGAAAAGGGATGGAAGCACATGCTGACTTAAAAAAAAAAAATTTTAATTGTTTTTGTTTAAATAATTTATTTATCATTAATTATCCTAAAAGTCTTTTTCCAAAATATATGAAATTTTTAAAATATGAAGATAGGCACTTCTTTACTCTTTTCTATTCTAAAAAGTTGAGATTTATAAATTGTGTCAAAATATTTTTTTTTTCTTTTAAGGTAAGATAATTTTTTTCCAAATCAAATTTTTAAAACGCAGACTTGTGTACTTATAAAAGTTTTTGCACCTCCATGTTATTTATTTATATACATATATACGAAAATTCTCTCCTACATACCTTGGCGCACACCAAAAAAAAAAAAAAAAAAGAGATTCAACGTTTTTATGATATAGTCAAATAAATTTCATCATTATAAAATGATTAAGGATAGTGTAAAACACTAAATTAGATTTATTTCTCTCGGACATCGCTAGATTGTATATACACACCGAAGAAATTAATAAAAATGCATATTGACTTTCTGAC

>Cs1g19170

CAAGAGAAATTCTATGGTTTGGTTACGGCAATACTACTTGGAATATATGATCCATATAACAAGAGAAACGTGTGTATAAGTTGAGATGATATATATATATATATTAAATGGGTTCGGAGTTCCATTTTTCAAAATCTGTTGTTAAGAAAAGGTAATTGCTATACCCTAATTTAAGTTATCTGGTCGTTTTCAGCTTCATAACATTGCAACAAACCTAGAAAGCAATTTGGGTGCGTAGCATAACCCTTTTTTTTTTAAGCAATCTTTAAGAAAACCAAACTCTCTCTACCTTAGAGAGATACATTATCACGGGACCCCTTGAAAAGCCAAAAATTTTGTTTAAGTGTGAGGATTGTTCAATTTTATGTTATTAGTCCAGCAATTAATTATTTTCAGTCAAAATGAAATTCTCTTTTTACCACTCTCGTCATGTATTGTAAACCAGTCATTTAGGCAAGTTTCTGTCACATGAAAAGGTACAGTAGTCTCGGGCATTGGTCAGCTTGATTCCCATACCATATAAAAGGCTATAAGCTTAACTCATACTCTGAGTTAGGATTTTGAGTATGAGTTCGACCTATGAGTTTTTATAAGTATTAACTAATAATAAGTTAAAATTTTTAATGTGAAAGTGAATATTTATATTAGTAAAAATTATTGAAAATTGCTAAACACCTAAAACAACCAAAACACAAAAAATGGCACATATAATGTAGTTAATCTAACAAAATCCGAAAAAAGAGAAATAATGAAAAAAGTTATAAATTGTCAATGGATCACACCTAACTATAATAGAAACATCAAACCAACTTTATTCGACCAAAATTAAACGTCATTGATTAGTAAACTAGCCTGTTAGCCCACCAACCTCTATCTCTCACTCTCACATAATTCAAGTACTCCAATGACAGCATTATCGGCCTTGCCTTTACGGACACTCAACCTGTCCATATACCTTATGTGATGTTTGCAATATTGTTCTCAAGTACAATCATGACCAC

>Cs1g18480

GGATGTACTTCAACGAGACCAGCTGGGTTTAGCAGACATCTGGTAGTAGGGTTCCACGAACAGGTATATCTTGTAAGAGTCTAGTAGCAGGGGAACAGGAGCAGGAATATTCCGCTCGTCCACGAATACGCCATCCGCGAGGGCGGTCTTCTGTTAGAAGTATAAAGCCATTCCTGCTAAGGGTTGAGGCGAGGAGACCCGGTCAACGAGAGTTGGCGAGACGTGCTCTCCAATCCGAGAATCAAGGCACGAAGGTCATGCAACCATCCACGATTACAGAAACGGAGCATTCACTTTCGAGAGGTGGGAGGATAACGGGCGTGAATGGAGGGAAGGTCTGAATTCAGGAGAAGCCTATAAAAAGGTAGTCAACGAAGGTAAAATTGTTATAATTTTTGACATTGAAACTGAGATAAAGAGAGCATTGAGTAATCGATACCAAAAATAAGCCATAAAATTAGTAGCTAACGTTCCCAGGAAGACAAAACCTTCATTTTTGACTTGGGCGTCAGAGGGTTTAGGCCGAGAAAACAATCGGCGTGCTCTGACTTATTTCTTTGTACGCATGAATTTTTGGAGAAGAAGTCATACGAGGAGAGATCCTGATCGTGGTCGAGTTGATTGAGCAGAGATCCTAATCGTGGTCGAGAGGGTAACCAGAATCTCGCATCAATAAAGAACATATCTTTAATTAAAATTTAAAGAATAATACAAATTATGAAATAAGATAGTGTTAAATTTTTTTTCAAGGAATAATAATATTTAAATATTTACTTGAATTTCTTTGGGATAAAATTTTTAAAATATTTATCATTATTCATAAATTACCTCGATTATTTAAGCAGTTCGGAAATTTAAAGGGGGTGTCAAGAGTCGAGACTCAAAAGCCTATTTGATGACAAAAGATTGCGTCATCCGAAGTTGTCAGGTGTTAGATTTGCGTACTTTGTAACAAACTAGTTCTTCAACTAACAGATGGAAGTACATAAAATGGATGGAAA

>Cs1g07290

ATGATGATGATGATAACAATAACAATAATAATAGCAGAAACAGAAACAACAACAAAAAAAAAATAACTAAAATAATAAAATTAATAAATTTTGTAATAATAAAATAACAATAATAATAATATCAATAATAGGCGGATGTACGGTACAGCAGAAATTAATAAATTTTGTAATAATATAATAACAATAATAATAATATCAATAATAATAGTGATGATGATGATGATGATGATGATGATGATAATAATAATAAAATATTAAAATCAATAAAATTTAATAATAATATTATTAATATAATATTAGTGACATTAATAAATAACAATAACTATGATCATGATGATTATGATGATGATAATAATAATATTACCCTTTCTTAATTAAAATAACTAAAATTTATTAATGGGAAGGGGTTATATGTCTTTTTTTTAAATATTAGGAAATTTTATATCCCTTTCCCCTAACGTTGGGGTCCTACTAGTTATTTTTTTATGTATTTTTTATTGTAAATGTACAATATTATCCTTTTTTATTAAAATAAACGGAATTATTAACCGAAAAAGGACTTGCATTTTTAAAAAAAAAAATTAGGGCTTTTCATGTTCTTTTTTCCAAACATTGGGGACCCAACAGTCATTTTCCCTATAATATATAGAAACATAATATTAAAATAAATATATAAAAATTTAAATTATTTTTCATATTACATAAAATAATTTTAATATTATTAAATCATGAGATATATTTTATTAACTGTAAAATCTATTTAACACAAAGATTTATTTAAAATTTTTATAATAAACACAAATGTATTATTAAAATAAATTTAATAATTACATTTTAAAAATTATTTTATGTATAAAAAAAATTCTAAAATTGTTAGTCTACTAGACAAATTAAACAATTTAAATTATATTTATATTTATTAAAAAATTTGAAGGTCAATTTTTTTAATTACTATTTACAAAGAGTATTACAATCAAAAGAGGGAGTTTAATAAATAGTAA

>Cs1g08260

ATTAAAAATTTTCAAAAGCCCTAAATTTTATATGTATACCACAGGCAGATACATGAGTAGGCAGGGGATAAACATGCATTCAACTATAGTAGCACAATCTTTCATGCATTTGCATCTCAATAGGTGAAAACAACATATTACTCCCTAATTTATCATTACCCCCAACAAATTTGTTACAAATCGATCTAATCTATATTCTAGGTTTCTAGTAACAGTGATTAACAACAATCAACATACATTGTTTCAAAAACATTGTTTCATGTGAAAAATTAAATGAAAATAAATAAATAAAATGAAACTTACTTGATTGTAGCTTTAAACACCCCAAAAACCAAAACGTCGTGACTTCTTAACAACTAACCACTTCCGTCAATGCCTCTGAATCTTCGAATTAGCAAAAGATTTGAAACTTAAGTGTGGTGCATTGATGAAAATGCATGGAAACTACAAGAAAAATGGTGGGATTTTGTTGCTTCTTCATGCATGGTGGTGGGGAATAAACTATGGCTGTTTTCGTTGCTTACTGGGCTGGTGGACCACTATGCCTTCTTTTTTTTTTTTTGCTTGTTACAGATTCTAAACGACGTTGTTTTCGAAGCATATGTGAAATTATCATTTTATTCTTATGACGTCAGTAAAGTCTTAACGATTTGAAAACAGAGGTGGAGAGGTGTAAAATAATATAAGTTCAAGTGGAGCGCAGCAAAGAAAATAAAGAATTATATATTTTTTAAAATGTGAGAAAAAAAAAGGTGGACGGTAGATAATTTTCCTTATTAAAATATGCTTAGTAATGGTAACCTTGAAAGGTAAATCCAATAGTATAATTTTAAAAACTAGAATGGTTAATTTGTTATTTCAAAAGATTAGGGAGTATAATGAAATTTAACTTGTATATATTGTATTAAATTTGAGACGTTTTGATAAAAAGGAAAATGGTTAAAAGTGCTAAAATTTGAACCAAGTGATGAGGGTGGTGAGGTAAGGTGAGGTTTGTATAA

>Cs1g22990

CACCCCTCTAATATTAGATCAATGAGTTAATGTATGGCGGTAGTCCCCGTTATTTAGTATGATTGTAAATATTGTAATTGTAATGTCTGATATAATATTAGTAACAATCTGTATATAACAGAATTCAAAAAAAAAAATTGTCACAAAAGTGGCTATGCTAATTAGCTTTGATCTTCAAGATGACTTGGTTTATTAACCAATTACAACAAGAGGTGACTAAGCGCTAGCCAAACTAAGTAAGGGGTAACGGTGGATATAGACACTAATACCACAACAATATATATATATAAACGAAAAAGTGGGTGTTTATATAGTTATTCTCATGTGCGAGTATACTCTTTTTTTTTTTAATGAAAACACATATTATTAAACTGTATAATTTAATTAAGTGAATTTTCAATACACTATAAAATTATGAAATTAAAAAATATATCCTTATAAAAAAAATTAGGACGGTACTAGAAAAAGGTGACACTAAGTCACTAACACGCGCACACACATCAGATATGGTACAAGGAATAGGTGGAAGGTATCTGGCGTACCAATCATGTAGATATTATATTATATTTTAGGGGAGAAGATATAGAGGAGCCTCGATTCGATTGAATTTGCTTGTCCTCCTCCCATAACGGAATTCACACGCGCCAGACGCAAATGTAACAGAAAATAAAATATATAACGGCAAAACGACGCTCTGCAGAAAGAAAAGAGAGGAGGAGGAGGGAAGAAACGAAGACCAAAAAAGAAAAGAGGGAGAAAGGAGAGGCTTGACTTTAGTTATATTATTATTTGCCCCCTCAAAACGGAGAGAATAGACCTTGGTAAGACCCCGCTTGGGCGGGTAACCGAACGGAGTACGTTGTAAACCGCGTTCTTGAATCAAACCACCTCCTCCGCATCCAATTCCAGTCGTTACAAATTCAAAAACACAATGCGTATCATTTGCTCCCATTTTCTTCCTCCTCCCTCGTAATTGAAAGAAAAATAATGATAGTGTCTGG

>Cs1g08900

TTCCTTAAAAAGCACGCAGATAAACAATCATAATATTATCAATTAGCACATTAGAAAAGGGCTAACAATAAAATATACCGCTATTGGCACATTAATAAACAGACACAAGTTTTATAACCTAAAATTAAAATTTCAAAACACATTGAAAAATGCAAAAGTAATTAATTAATGGAATTTGCAGTCAATCAAATGGTTAATTTAGGACACAACAGAAAAAATTATGAATTAAAAAATTTTTTAACTTTAATAGATGCCACGTCAACAACACCAACACCAATCACCAATCAATAAGCACCGCTTAGAGAGTTAACGGCAACGCAGCGCAGTCGTGCAGTGGAGGCAAGGCGAGCTTCGTGCGGGCGTGCGGTGAGGATCTTACTGCAGCGAGCAACAACAAAGTGGTGAGGCCTAGCTTCCAGCCGCAGCGAGCTTCAAACAGCAGTGACGACGATGAGTTCAAAATTTTAGAGAGACGAAGAAGAAGAGTGCCGAGCTTTAAGGGCTGTTTTCCCTAACTGAACTGAAAGGGTTTTAGTTTTGAAGGAGGGGCGATCTGTAAATTGCAATTTGTTGACTTGTTGTGGATCTATGTAGAATGTGTTCTAATATTTGTATATAATCGTAAATAGGAACAAAATGGGGCGATTTCAATGTTAATTACCGAATTTACAATTTCGGAACTTGCTTCATACAAATTAACAAATTAAATGTTTATTTTCAAACTTATTAAAGTTCAGGGATCTATATCCGATCTAAAATGTCGTATTTTTATTTCTACTTTTAAGTTAAGTTTAACAATATTGATATTTAATTCGAAATTCTATCTATCATTATTATTGAAAGTAAAATTTTCTAGAAATCAAACCAAAATTAGGCAGCCAGATAGAAAAATATTTGGTAAATTTGGTTTAACGAAATTTCGGATTAAATATCATTTTTCTAAATTTAAAATTATAGTGTATTTATTATAAGAATATACAATAAAAAGAAAATAACGGTAC

>Cs1g24210

GCCACTAACCACTTCCCAAATAAAGAGAGGAGAAGACGAGGAGCTAGCTAGCGTTTCAATCTCCATTTTGATTTGTCTGGTGATATGATTCTGGAATTTAGAGGCTGCTCGGAAGACACAGGGATTCTCTCAAGGTTGCCACTTGTCACCAGTCAACCACCCAAAAATAACGTCATAGTCTCAAATTTTCTTTTTTTTTTTTTGCCCTTTTTCACTGCAAGAATGAAACCTATTCTTAGCTCCTTGCTGAACTTGGCACGACAATGAAGATTGAAGAGTGGTTGAATTTTCTCTGTTGTCTGAATGGGCTGTGTGGCCCATCTGATGTCGTTTCTCTTTAGATGGGTGCTAATGTATGTTGCTGCCATCCCTTTCATATCCTCTTACTACCCCCCAACCCATTGCCCTTTCTTTTTTCAGAATTAGAAGCCAGCCCCTTTCTTTGTTTAAATAATTTTAAAAAATAAAAATCACCAGGTACTTTAATTTTATAACCTAATTAAATATACATCTTTGTAATAAATTTTAATTTCCAAATACTGTCAATACAATAGAAACTGCAAACAGAGCAATAATACTGTCATATAGCTTTTTACGATTTAAATTTAGTTCCCATAAATTTTGTGTAACAGTGAGTAGTTAAAGCCAATTGATAAGGCAAATAACATTAGAAATCGTTCGAATAACATTTTAGTCAACGTGATTAGCTTACGCTCTAATTGAGACGAAATTTGTTCTGATCTAAACGACTCGTGAGGAGATTTTGATATTAACATTTTGCAGTGATATTAGATGATACTCTGATTCAAACGACTTTATAAAAGAGATTTTGATATATAATCCTAAAAGAGGAGTTTTTATTAGTCAAAGACTTTAGAAAAGCAAAAACAATTATGTATATATTTTTAATAATTATTTTAAAAATTATAATATTATCAAAGGAAATTATAGGTTTATTTTTAATCAACTAGAAACCTGCAATAACTTTTTCAATAGAATTT

>Cs1g26060

TTTATGAGATCAGAATTGATTTTCAGGTTCATAGAATGCGCTTTAATCATTTAAGCACTTTACTTGGACGCCAAAACTTTTTTCGGAAGTAGATACCAATTATGCCCTCACAATTTTTTTTTTTTAATTGGAGCACTGTTATCAAATTTGACAATTAAATCATTAAGTGCACATCTAGATACTTAATTATAAATTAGAATAAATGAATGAGTTTGATTTCTTGGCATTACAAGTTAATAGAATTCTCGAATTACCACGGAAGTGAATATATGCTGGAAAGGAAATAAATGCAAAGATAAAACAGAAAGGGAAAAGCAAAAAAAAAAAACAGATCGAAATGGATTATTTTCATGTTCGCGTAGATGAGTTGATTATTGATAAGACGATTGCTTTTTTAATTTCTTAAAACTACTTATGGGTTCCCGTTCTAGTGTCCTTCTTCTTCACTTGATTTGGACAAAGTCAATATGTGCTTTTTGTGTTTACGAGTCAACAAATTTTAGACCTCGGTCTACTTTAAGAATCCTGCATCTATTCATGGTGATGATTTTGGAGCGAAATTTACTTTCTTTTTTCTTTCTTTATAATATGCTAAAACAACAAGTTGCATTTAATTATTAATCCAGTCTGGGAACCAACTAACGCACCCTTGGACTTATCTGGGTCATTATATGAATAAACTTATTTTATTCTTTTGAACTAAGACTCTCTTTTAATTGGATGCTCTATGCGCCAATCCGTCAAGCCTTGTAAAAGTTTATTTAGAGTATCTTTGGCATTGAAAGTAATTGGAGTTAATGAATCCGCGTCTTCAATATGCGCTCTTGGTACTTCTTGAAAATTTAGCTTACATATCTGCCAGTCAAAATCTGCTAGACAAGCTCTTCGCTTGCCAAGAAGCCACCAGGCTCAGCATTCCATAGTTGAAAGGTAGGTTTGATTATTAGCTCTTGAAGGGTTTGCTTTATAGTAGTCTCTACATAATATTAATCTTTATTTCT

>Cs1g17170

TCTTTTTAGTGGACACATCAGCATCATTAATGCCGTGGTGTTCTGGAATTAACACATGTAGCAACTCCTTCACTTGTCGTTCCAGACCTAAAACGTCTGTCTCGTCTTCTGCACAAAAATAATCTGAAATTCTTGCCCAACGCTGCTGCTGACTTCTATGGGTACTGTAGCCTCCTTGGTAAGGGCTCAAACTTGGGCCAGTATCGTTGACCTCGACCAGTTGCTGCTGATGCTCATAGGAGGAGGAGGAGGAGGAGGAGGAGGAGGAGCCCCTGATTTCGAGTTCGCGCTTGAATTTGTTCATCCCGAAGCTGAATAAAGACCGTAAACGTAAATTCTCAAAGCTTGAGAAAGGGAAAGTAGTGACCGTAAGAATGGTTACCCAGACAGGGTCAATTCTCCACATCTGGCACACTTCCTCTCTTATGGCCAAGCCGTTGATGGCATCCTGTGCAGAGTAAAACGCTTGCATAAAACGGGCTTCATCATCAGCGGCCTCTCCTCCTGCGGAGGAATCCAAGCATGGCTTCTTGTTCTTGATCAACAATTGGAGACTTTCAATAGCTTTCAGTACTCGTTTTTGAGTCCACGAACTTATCGTTGCTCCTTCGTCTGCAGCAGGGAACAGAGTTTTAAGTTTCACTATCAAAAGGGACACTGAAGATTCAGCATCCATGGATTAGAAACCAGGCCAATCAATAAAATAAAACAAAATTACGTGTTTTAAGCTGTATTTCAGAGCAGAAAATTGATGATCTCATGTATGAATTAATATAGAAGAAGAAGAAGATATAATTTCCCCGTGAAGAGTTTTATTTAAATTTTTTTCGAATTATTCTTAAAGCAAAGGGACTCTGCAAGTGGCCGACACTTTTTGTTTTGTTTTTACGTGGCGGGGGTGGATAAAAAGTAACAAATTTTCTCTCACAACAGCGACAACGCTGAATAAAATATTTTTCCATGCTCAAAAGCGATGCCGTTTGTTATGTAAAAGGAAAAAT

>Cs1g19820

TAAAAGTAGAGTGGATATTCTGACTTAGTTTTTTACAAGTCCGAGTTCGAGTTGCAACTTGTTTAGTTTTAAGAATTCAACTTAATCAGGTCCAAAAGTTGGTTTGGTACGGTTGAGTTTAATCCGGATCAACTCAAATTCAGTTTAAGTTAAGGTTATAATTTCAAGTCAAAAATTCGTTGTATTAAAAATTTTTATTAGATTTAATTTTTTTTTTAAATGTGATCCGATCGAATTTGATGTTTGGTTCGATTTTGACCCAAATTGAATTTAAATCCGATCGATTTCTAATTATCAACCCAATTGATTTTTAAGATCAAACCAAATCGTACGGACCGAAGACCGTTATTTTTATTCATGCCCACCCCCAGGTGAAAGCATCTTCATTTTCAAACGGCCTCGTGACTTTATTTATTAACACGTAATATAATAGCTCAAAAATTTTAGTTCGGTCATATTTCTCAAACATTTGTTTTACAATTATAACTTCCAGTTCAACCATTGTGGCCTAAAATTCTTTTTGCCCATGGAATCTATTAAGTTAATCGTCTATGTTATTACGTGACAAGCAATCTTTCAATTAACTATTTGTTCCCTCCTTTTAACCAATTTTTGTTCCAATATTTTATTTTCTAATAAATTGTAAATACTAGTTCATTCCATGATATACCCTATAGATGAATTATTTAAAATCAGAAAGTTATGATAGTATTAGGTATCTCAATAATCATAACATATATCTAATTACATGTTTTATTGAATAATAAATTTAAAACGGAAAAAATTATTATTATATCATCTAATAAATGAAATTGACATAACTTAAGATGCATATTGAGATTCGAACATTGAGGGATATTATAATTTTATGGAGAGAAAAAAAAAATAGTTTGAACGAAATTACACGTAGGATTTTGAGAAGGTTTTATTTTACGGCGCGCGCGAAGTCCACCCCTTTTATCTTTCTTAGGCGCACAATAACGCAAAACAGCATTGTCAAA

>Cs1g14940

ATTTACGGTGAACAATTGATACACCCTTAAAATTTTGTAGTATTTTTAATAACATAACAAATTTACTGTACAGTGTACACGAAGGATAATTTTTCTTAATTAATTATTAATCAAAGACGATTCTATGTTACATCACATCAACGCTACATTTTATCAAAGCTTTTTTTTTTTTTATACAAAGCCAATACTAAATTAAAATCCCTATAACACTCCTTTCAAATGTTCATTTTCAAAATCTTCTCGGTTTTTGTTTGGGTTTGATTTTTTCAATAACGTTTGAAAATATGGTCAACCCCAATCTACTCAAAACTCATTAGTATTGAGCTGACCTCGTAAGTACGACCTGGAGCCTATGTTGGCAATCTGGTTTACAGATACAACCTGAGATTTATGTGAACAACCAGATTTAAAAATACATCCTTGAACTTACATGAACGATAGGAAAATTCACCAGTCCCACCTGAAAATGAAGAAATCCGACCTCAAAATTTATTTGTCCGACTCGGGAATTAGCTTGACATGGAAAATTATTGGTTTAAATAACGTGGCCTAATTTTTAGGGAAGTTGTACGCCACGATTTGTTAAACCGTCTCCAATTATTAACAACTCGCAACTGCTGAAGAAGGTGGGCTAAAGTTAAGGTGGTAACCACTCACGACCAAGGGCTATTTAAGCTGAAGAGGGCAACAGAGAAAGGGGTTAGCAAAAATTAACCAAAAACTCTACAAGTGACCTATCTAAGAGCCTTAACATTCTCTACTAGATTTTTAGTTCCTCTATCAGAAAATTCCTTTATCTCGAATTCATTCACAATTCTTCCCTTTCATTTTTATCTCTTCAGATAAAAATTCTGTCAACGCTTTACCTCAGACAATCTTTTCTGCAAAATTGTTAACTTCTTTGAAGCTATTTGGTTGTAAGTTGGAGCAGCCCTCACATTGTGCAAATCTCCAATCTCTCAAGAAGCTTTCTCTAGATGAGGTGTATGTAAATGATCAGA

>Cs1g15050

CCAAGCGACACATCTTTGTAAGTTTATTATATTCATAAGGCTGGCGCTAAGTCACGTTGAGTGTAATTTGCGCCTTTCACAGCAACAGTACTCAATCTCATTAGTGGTATTGTTGCTGTGAGAGGCCGGTAAATTACGTGTGCAACCCAATTGATTTTTCCATAGTTGTGTCATGTGTGCATAAAATAATAATAAGAAAAAAAAATCAACAGTTACTTAATTCATTTACTTTCACAATTTTTTTTAATAATATTATAAAAATGTACACTGAACTCAGATTTAATATGGCACGACTTAGATACTTAAGCTCATGATACGAGTTGAATGTGTTTTAAATAACAAATTGCATTGGGATCTCTTGCATTATTTTTGATGTGGTGTTTATTTTTAATATGGTGCAATATGAATGTATGATTTCTAGTAGTTGAAATTTATTTTTAAATCTACCGAAGAAATTATTCTCCACAAATAAACTGAAAATATTAATTAGATAGGTTAAAAACATTATTGGTGTCACTCTTTAATTTGAATTTATTATTTGACAAGTGGATGTAATTTATGCAATGACCAATGAATTTGATTCATTTTGGATTCAACGAATTAATTTTATTGGTAACTTTAAATGTTGATGGGTGATCTAATAGGTAAAACTTTAGCATTAATTATGTACACTATAATTATGGTATCTAGAATACTAGATGGAATCTACCTATTTACAGATAAGATTAGATTGAGATTAGAGAGTCATAAATGTAAATCGCATTTAATTAAGGAAACCCATTACACAAACCCTTAAAACGGAAACAAAAAGATCAAATAATTGAAATATAAAAGATGATATCCAATAAAAGTTTTCCACGTGGAATTCAATACTAGAAGAATCCGCACGTGGATTCACCGTGTCAATAAGCAGTCAAAAATTCCGCGTATGAACAGAAAATCCTTGTCCATTAAGAGAGAGAATGTGAAAAACGACATCATTTTGAGTTGTTAAAAAAAAA

>Cs1g26000

TGCTCCATTAATAAACTCAAAATCACTCTTATGTTGTGTTTGATATATTTTTTATATATATTTGTTTTTATTTTCCATGTAGTCATTAAATTGAGCAGTGAAGTTTCAACCAAGCTGCCGCGTGTGTTGCCATGAGATGTTCTTTCAACAGCAATTCATGAAATTACTGCCACTTGCTTATTGAATATATGGACACGTATCTTGTATGCTACAAAATGGACACATGGCCACTCATGTCACACTTGATTTTAATTTATTATTTATTATTATTTTCAAAGTAATCCGATCTTTGAAATTTGCATTAGATGTTAGCCAAAAGCAGTAAGGATAAAAAACACACGCAAGACGTCCATGTTCTTATTATTTTAAATAAAAAATTGCCCATCAAATATTATTCTAAATTTGACATTTTCTTTTTCAGAAAAATATTGCTTTCAGATGTCAATAAATCTAAGTGTATAGATTTTAAGGGCCATTTTCACCTTATTTATGAAAGTAGAAAGAAATCAAGATTGAGTACTTGAGTTCAATTTATGCTTTCTATAAATTAACCTGCTTAACTCAATTTATACATAAATTATTTAATATTACAATTCTCTAATTAAATATCGTAATTAATTATCAATAAGAATACACATTATATCTCTGAAAGAACTTTTTTAAAAACAACACATAATTACAAATAAAATGCGTATTTTCTAATTTTAACTATTTCTTCACCATCATTTCGTGCTTGATTACAATAATTTTAAATAATTTCATGTATGCAGTTTGTTTTTTTCTTGTTATAAATAAATTAATATTATAATGGGGTGCGTCCAAAATCGAAAAACAAAAATGGAAATAGATTTATTTAAGGATCAAAAATAAAAATAAAATATTTTGGGTGTTTATACTTTCTCTTTCTTTGGCTACAGGTGGAGCCCACAGTTGGCCATTTTGCATTGCTGGCTTTTAATCCATGTTCGTTTTCTTTTTTATAGGCCTCTGTTTTGTCTGCT

>Cs1g21580

TTAAATAATAATTTTAATAAAATTAACATAAATATAATATATTTTTTACCATACAAATATACATTTTTAAACTATATCAATTAATATTTATTAAATATTTTAATATTGTAATTTAAAAATTATAGTTCTCCAACCTTAATTTTGATCGAAACTTACATATATGCTGATAAATATTTCAATCTTTCCATGTTTCTGGGTTAGGTAATTATACGTTTGCCAATGGAGAGAATGGGGGTCCGATATTTTCAATATATTTGGTTCCAGCTATTATTAGTTATTTTATATTTTGTGAAACGTTATATCATGAAACAGTGAATCTTTTTGGTTTCTCTTTGCAGTGAATTTGGTGATCCGAACTTTTCCCGGGAAACAGACCCCTTTCATTCTGAAACTATAATTTACATTTTCTCAACTTGTAACATAATTGTATTCGGTGGATTTGATTTCCAATTATGATTCTTCAACTCGACCGTCTAATAAATAATATTTCTTTATCTCATTTTATTAATTATACAAGTTCATAAAAAACTGACACTTGGTTGGGTTCAATAAATTAAATGTTTTCGAATATGATTTTAGAAAGCATTTTAAAAAATTTTAGTTGGTTTTCTAATAATTAAATTGCATTCAAGTATTTCCAAAATCTTTCTGAGCTTCAGGGTTAGATATTTATGCCTTTAATTGAAAGAATTTGGAATACTTATCAGCATATATGTACTGCCCAGTGAGGGACTCCGAACAACTGGACCATGGCTGGTACTGGCGCAACATCAAAGAGAGAGTCATTGGTCTAAAAATGGTCAACGCATTTTGTCGCCATTATTAATTTTTGATTCCACGTCTCTGAGCTCCGCCACCGTTTAGCCGCGTCTGATATAAGATGATAACTTGCGAATCATCCAGGCCCCCCCTGTCACTGGCCTACCTATCACTGTCGCCATCGTTTATTTATATTTAACAGATTAACTTTTCTCTTTTAGTTCCAGTTTATCATTACATTA

>Cs1g18670

ACTAGGACATGTGTGGGAAAAAATCCTATGCAACTTGTCTACCCAAATGATACGACTTCTTGGTTAGACAGTACAATACATGCAGTTTCAGATAATTAAGAAACTAAGAAGTTTGATTATTATCGAATTTCATGCCACGTCATCAGAAATATGAATAGCTGTTTGGATGCCATTATACTATTTAGAAGATGATTGATGTTTGTGTTTGTACATTTAATTAATTATTTAGCAAATTTGAGTAAATTCATTGACAGTCATAAATGGGATAAAATACAGAAAAATAATCGGCAATTATTCGCGTTGAAAAAATAAGAGATTATTCCAACAATTATTTGCTTAACAGCATGTGTTTCAGCGTTTACATAACAGGTTTAAATTATTTATCGTTTATAATCAAATTTAAGCATCCACTAAATTACCAAAATTTTAAGAGTGGAATGGTTTTGCAACAATTCTATTCTTATAATATATAATGTCAATACAAATTTAAATATATCAATTTAATGTCTGAATTGAGGTATTATATCATCTATGTGTGAAATAAAATTTATTTTGTAAACAACAGAAATATTTAATATTAACGTGTGCCACGCGTCATTAAAGATTCCTAATAAAATCTCATTATTTATTATGTAAAATATATTGACATTAACGAATATTCAATGAAGTAAAGAATGAATCACGATCACCAATCAAAAAATTATTGAAAATATTATGTTATCATGTGTCATGTAAAATTTTAATTTATTATATTTTAGAATATAAATTCAGTGATCTTACATGCATAAAGAAATCCAAAGTTATCAAGTAAATTCAAATTGTTGTTTGGCATGAATAAAAATGTAAAAATAAACTCATGGAAAGAAATTGAATAAAATTTATGAAAATAAATAAACTTAATAGTTGTATATTAAAAACCGTACTAAAGAGTGAGAATGCCTCACTCGCTAGGCTTTACTCTTTTTAATATTTTTCTTCTCTCACCCAAACGCATACGCGCG

>Cs1g03420

AAGTCTAATTGTGTTTGCAGCGACATGCCCAGCTGCTACTTTTGATGCCTCTGAATTGCTATCATCATCCTGTTAGATAACAACACAAAACTATTCATCTAACCTCATCAAGGGCTCGCTTGATAGTAGTAATTCTCACAATCTGCCAACTCTACTTATCTATCTGATACAAAATCAAGTTCCCCAATAACAAACATGTGAAACAAATATATAGGTGAAAAGGTGACTAATACAGTAACTGACTTGAAATTTCATAAAATGAATGTTATAGAAGTAAAGTAACCACCTTTAGAATCAAAGCGGCTATTTGTTGAAGCGCATTTTCAGCATCAGAAACCACATTATCATTATCTTTAGAACTTATAATAATAACACGCTCTTCATGTCGCTGCCAAAAGAAAATCATCAAAGCACATCATTTATTCACCAATCAATGATTAAAAAAAAAAAACCCTAACTTTCCAAAAACAAAATTAGCCCAAAAAAAGAAAGAGAACTTACAGCAATAGCATCAGCGATTTTGATGGTGGCCTTAGTCTCTTCTCGAATCTTCTGGATTCTATGTCCTTCTTTCCCTATGACCTTTCCAATCTGTCTTGAAGGTACTATTATTCTGAACAAGACGTCTTGTGCTTTCGCTCTCCGTTTCGGATCCGATCCTTCGATTTCTCCGTCTTCGCGGCGGCGCTTTCCGGAGACATCGTGCCGCGGTTCAGGTTCGGTGACGACGGACACGGTAGGGTTTACTGTAATTTCGCCTTGTTCGGCCATTAATGCGTAATAACGGCTGAAGTGGAGTGAGAATCAACGAAGAGATTTATCAACATCGAGTCATTGTTGCTGCTCAAATTACTTCTTTATCCTCCCAATTAAAAAGGCCAAAAAAACAAAGTAAAAGTTTTAGCCATATTTTATAATATCACTGAAATAAAGGATTAGCGTGTTATTATCCATTACCGCCAGCCATTCCTATTATTTTGTTTTTAAGTTAGAAAATTAAG

>Cs1g06830

GTGAATTTGGAGAAATTTTAGAATATATTATAGGTATTATGTCAGTCATATAATAGATAAATGATGTTATTATATATGTGTATTTATTTTAAAAAATCATTATATAAGTGGTGGTTATATTAAATTGAATTACATATATCATATATGCATCAATTGATTGTAATACATCGAAGGTATTTTAGAATTTCTCGTGAATGAGAGTGTTTAAATTGGGTTTGTAAAAACATGTTTTCAGAATCGATTTTTTATAATTTCCAATTTCGAAGTATTTTACTTTTAATTTCTTCGAATCCTCTACTCTTTTCTATGTATATTTTGGGAAATTGATAAAGAATTAAGATGGTGATAAAATGACAACTTAATAATCAGTGATTAGTGTGGATTATAATTTTATAAATAAAATGTTTAAACTTACATCTGATTACAGTTATTTTTATCACTATCTGGTCTAATTTTTCTATCTAAATATTAATGCTTTATATTTTAGGTAAATTTATTTTCTTATTTTATCCCTCTCTTTTTTTATTATTTTTTGAAAAAAAAAATAATTATCTAGTGCATAAATGAAATAGTTTTGTACTTAATGTTCATGTCAATTTCAAGTTGATAAGAATAAAAAAGATACATAAGGTACTTGTGCTTCACTAATTTCTTTACTAGTACGTAACAATTACTTGCTCTTATCATATGACCATCATCAAATAATTTATCATTTATTAAATAGTTAAAACCTCATAACATTTACATTGATCGCATTAATGTTCTTCAAGACATGAGTTCTTTTGAATTTTGAGAATTCTTCTACCAAATGTGCTCATTAAGAGAGGTGTATGATAAAAATCTAATTAAATTTGCTTTATTATTTGCAAAACTTTTGATCACATTTGCTGTTTTTTTTTTTTTTGAAGAAAAAGCACACAATTCACGTCCCGGGTTGCGAATTCATTGATAAAATGACAATTAAAAGGAGGTGCCTTCTCATTTTTTGAAGTAAATATTTC

>Cs1g15280

TAGACTTCTTTTTCTGCACTTTCAGATGGGGAAAAGTAGCACAACACGTTGCATTTGAATTTGGAGAAAATACACAGATGATTGAAATGTTTTAGTGAGTAAGAATGCAAATTGTGTATGACACAACATATACACATATGACCGTAAAAAATAAATATAATTTATCAACAGAGAGCAGCTGCATATGCCCAGAAATAACAGACAAAGAGCAGCTGCAATTGACTCAAATCACAAATCTCTCATGCCCAGAAATAACGAACAAGAAGAGTGCATAAACAGACAACTGAAGATAAAATACACCTTACATTAATTCAAAAAAGAACAGAGAACAGCTGCAATTGACTCAAATCACAAATCAGAAATCTCTCATTTCTTCAACCTCACAAACCAGAAATCTCTCATCATCATTCATCCTCAACCTCAACATTAGCAAATTCAAAAATGTACCCAAATCAAGTGCGAAAGGGAAATCAATTGCAATTTCCCAAACAAGTTTCGGCTACCAGCGTTTACGGTGACCGTGGTTGTGAATCCGTCGTGGTGGATCCCGAATGGCTGGACCTCGGTCAGGGAAGGAAAGTAACGACGGTGACCGTGGCTGTGAGTCGGTCGTGGTGGATCCCCAATGGCTGGACCTCGGACAAGGAAGGAAAGTAACGATTTTTTCAGTAAGGAATCCCAGGTGAAACGAATTCTTCGGTAAGGAAAGTAACGAATTCTTCTATCTGTCTCAGGTGAAACGAAGATACAAAGAGGAAGAGATTTACGGTGAGTGAGATATTCTATCAGAAACGGCAGCGTTTTGGAGAAGTAAAAAAGAAACAAAAAAGAAACGAAACGACGCCGTTTTATTTATTTTCGGGGTTTGACCCGGGGCAAACATCATTTTCCTTTTTCCTGACAGGTGGAGAGGTTTAACTAATTTTAATTGGCATTTAATTGTGGGTTATCTGTCTAAGCTGTGGCATCTTTTTGGATTTATCAGCTCATTTGTGCTTATG

>Cs1g21660

CTATGCATTGATACAGCTAACGTTGGATTTGTTTGTCATAAATTTACAATAAAAAAATTTTTAAATTAAAATTTTGACTATAGATCTACAATGAACGAAAAATTCCTTTGCCGATAACAACTGTACAGTCCTAGCTTAAACAACAAGAATTTTAATGGTACAAAATATAGTACAAATGACACCAAAATATGATAGCGGCCAATAGTGAAAATCATTAATTTTAATTAAAAAAATTCAAATTATTAATTGAAGATAACTAACAATAAAATATAAATGAATCTTAAATTATTAATTTAATATAAATGTTAAAATAAAAAAATATTATCTTATATAATACACTCCATACTACACTAAACATATTATTATATTAGTACTTAATACAATATAATACAATGCAACTAATACAATAAAATATAATAGGTTGAAAGCACCCATAATGTGTATGTATATATCATATATGCATGTGTGTGTGTAAAATCAGTTACCTTATTTTTGTGGCATGGGTGTTATTCGGATCATGTTTTTTTTTGGTCGAATAAAATCAAACCAAATTTACTCATGAATGTCTGCTTCTTTGAGTCATTGCCTTATAAAACTTCATTGCTGTTAATTCGCTGATAATTACAAAGTTTATTCGGTAGTTAGCTTCAGGTTTTTGTTGCCCAGCATGACGATGAAAATGCTAAAAACGCTATGGAAGTGTTTTGGTAATTATACGAACTCATCAAATACTAATCTGTGTTTATCATAATAAAAATAATAGGCTATACCCTGTGTATTAAATTATAAATAACTCACTTAGCAAAGTCATGAATTCATCGTTTGATCGCAAATAAAGACCATTTCCATTGCTTTGGCCTCGAATATTTTGTATTCTCATTTACGTCTCGTCGTTTATTTTAATTCGCAATCCGGCCGCAGTAACGCCTGACAATTTCCGCTACAGATATATAGAGTCAACAGTTGAGTCCATTTATAATTAACGACTATTTCCCCTCGTG

>Cs1g18600

TGGTGCTCGGTTCTCAAAGAACGTTATGGAAGGACAGGAGACTGGCGGTACAAGGTTTCTCACATCCCTAATATCAGAGATTGAATAATGGCTATGTGCCAAGAAAAAGCCCAGAAATTCTGAGACTGCCGCAAGTGCATTTGTCCCCCAGGTAACTTCTTCCTGTCCTCATTTTTTATCAGTTAAAATATCGCTTTTGTTCAATGCAAAATCTTGCACACAGCAGTCACCAAGTAATTCAAAAGTATCACATTTATTTTAGGAACTGTTTTGTTGAGCGCCAGCTAAGTCTTCCTCATTCCCTAGATTATTGCAAGATTGAAAGTATTTTGGACTTTCCGGAGCTGACAGTCGTTAGATTTGTTGAAAAAATTAAAAACTTAATTTATGTCCTCATATTTATTCTATTCTACATTTGCACTTAAAAAAAGTCAATGGCACAATTATAAAAGTTAAAATAGCTAAGTCCATGTTAGCAATATATATTCTATCAACTACTCGTCCACTACACGGTTCAGGCGTTGGATTAGGTTAGCTTCAGGCCACATAATTTAGAGTTTAGCCTCAAAATGCGACTTTCTTATTGGGAACTAGTGGTTTGCTTGAGTATTATTATGATTAAGTCCACTAAAATTACTAATGCGGGGGTGACATGGCATGCATAATCAGCGGCTAATGCCAAATATTGACTTTTTATGTTACAGAACTAATTATTTCTCAGTAACTAATTTTTCGGTTTTTGTCTTGACAAATTTATCGCCACGTTCCATATATTATGGCTAAATATTTGATTAAAGAAGTGATAATTTCAGTTTATATACAATGCTGTTAATTCGAAGAGGATGGTCTTCAAGTGGGGCCCGCTGGCGTGGGGAAGTAGCCGAAAGGGTCAAAGGAAAGCCGCGTAGTGGGAGGCTAACAAATTCTTCAAGCACTTCAAAAGCACTTCCTATAAAAGCAGTTGGTCAGTCCCTTGTTTGGATCACTCAAAAAGCCAAGAA

>Cs1g25110

TTTTGAGATACATATGTAAAATAAAGACAACACGCAATCTGAACATAATTCGCCTAAAACTAGAAGAATATGAACAAAATTTGTTTGTATTATTATAAGATAAGATCTAAAAATGTAGACAAAATATCTTCTACACGTGATAATGTTCGATACCCTGCTCCAAAACATTCGTTTACACTCGAATGAAGATGAAGGGTGAAGGTTTGAGATAAGGATGATACATAATTTTCATTCAACAGTACATACTGTGTTCCCAAGTCGAAAATTGGGCCTAGTTCTTGGAAAAAAAAATATTTGATCGGCCCAATAATATGTCTACTAAACTGGCCTCTATAACTAAGAGCACCACTTCATGATCTTTATAAAACCCCACTATAAGTAAAGTTGTTTTCAAGAATAAAAATTAATTTAATTACATTCACTTATATTTTTTTTTCCCCTCTCCGTCACTTTTTATCTTAACCAAAATTGATTTACAAAAATTTAGAACAATCTATTAATCTTTTCTTATATCTTACAAATTAAACATTTATGTCAAATTGTACATATATTTTTTTAAAACCTGTCAAACTATGATTTTAAATATGTGAGCTATGAGAGATAAAATGGGGGCCAGCTAGCAAATCCATCTAAGCTTCTACTTAAAATCCACTTGCAGGCCCACCTTTTAGAAAAAAAATCAGTTAACTTAATTTAAAAAATAGAAAAATAAAAAATACTCTCAAACTGAGAGCTGTTGGCCAATCACATGATTAGACTTGTAATCTGTTAATGTAAAATCTAAAGTACGGAACGTGTCCAATTGAATGATTGATGGAGAATATCACATAATTAGCATGCATTTATAGGCAATCAAAAAGCTACATAATCCGTAACTAAGAGCCATGCATTTTGATTCGTTGGATTTAGAATAATGGGTTTTTCATTAACATAATTAAATGAGGGTTGTCCACCAGTAACCCAACAGGTTAACGACACAAATTGAAATTAGCAGCCGTTGG

>Cs1g24240

AAAACAACAAAATGTACACGTTGCCATCTGCATTCACTCAAACTCAATTCGCTTAACCGGGCCATATGCTGCCGATGCCAGCCCCTACACTCTCGCTGGCTTCAATTGACATCGGAATCGACCCTCCAGCACCACTGAACGTGATTTCATTTCATCACGTGGCTCTGCTTTGATTTGATCCTAACACTTGGCTTCTTCTTTCTTAGGTGAAAATAAATTCAAACAAACAGCATTTGAGATCAGTTTCGATCGATCGTTCGTGTGGTTCCCATTTTGAGATCAGTTCCAACCCACCAACAAAATAAAGCACTAAAAGTGAAAAGACCTGTAAGATCAATTCCAGCAAGTGTGTTTTTCACAAAAGTACCCATTATCTGACTTAATTGATATATAATTACAATTGCTTATCCGATAATATTTAAGTTAAAATTGTCGCAACAGGTAAAATTATTAGCCAGTTGTTCAGAACTAATAGTACAGCTCAGCTCAGAAGCAATTAATTAATCAAAGAATAGCCAGCTGTGGAATATATAATAGTCGATTATATAGCTGTTACATGGTAAAGTCTTGTGCTTCAATCACGCACTCGCTTTACTGTTTATTACAAAAGTACGTGAAAACTGAAAACTTTTGAAACAGCACCTGCGATCTTTTCCCTTAAGAGTCAAAATCATTAATGCACACGCACCCAACTTTATTCGAGCCATCATTCTCATTACTGCAGAAAAAAGAAAGCATTTACCAGTAAATTACAATAAAATCATCAGTGTATACTCTGTATGGTAGTATACTAGTATATGGTAATACAGAATAGAAACAAAACATCAAAAGGAAAAAAAAATAGAAAGAAAGAAAGAAAGAAAATGCCATGCCACGTGTGTTTTAATCGACGCGGTCGACCGAAACAGCGACCAGACTCTGAACTTTATCCATTAGTAGAATTATATATCGCAGCATCTCTTACAAAATGTTATTTTATTATATTACTTCCTTAACTTACT

>Cs1g08940

TATTTTTTAACAAGTCCTGCGTTCATAAAATATATTCTTCCTTGCAAGTTGATTTTTAGACTTCATCATGGGTGACTAACTTAATATATCATTTTTTGTTTAAAGGCAAAGATAAAGCTGATAAATCAATGAGTTGATTATATGCATTAATTTTGGGACAGCTGAAGTGCGCTTAAGAGTTAGGAAAGCAAACCTTTTTGGATTGCAAAACTGATTAATCAAAAAATGGCTCTGACTCTCAAGCAAAGAATGTCTTAATCTCTTTTATCTTCAAAAATCATAAAAGAGCAAGGACTGTATATTTCCAAAGACTCTTTATTTTCCTTTCTTTTATTGGAAGCCAAAAAGCTCTCCTGCAGAAGTGTATGAAAGAACATGGAGTTGCCCTCAAAATTTTAGTTTCCTTATGTCTCTTTGCCCGTAAAAAAGAAGCTTTTAAGCAGTGAATGAAATGTGTTTTTGTCTATCCAATTTCTGCACAAAACAAACCAAGATGGGAAAGGACTCATCATTAAAAGGCCTTCTTTCTTTCTTTTTTTTTGTTTCTTTTGTGAAATAGTTTCTGTGTTATTTATTCTTTGAACCAAAGCCAAAGTGAAAAATTTGGATTTTATTAAGGAATTTCTTGGTCTGAAAAATGATCTCAAGTGAGATTTACAGATTATTACTTTGTTTGCAGACTGATAAGTTATCATTTGACCCAAGTTTTAAGCTGTTGGAAGGGGTCCAAAAATTACATTTAAGCGCTTAACATCCCTCATGTAGGTAACAGAAACAAGGACAAGTTATTGTAATTTAATTAACAGTGAGGTGATTCAAACACGACTTCTTGGTCAAGTTGGCCGTGGAAAGTTACTGCTTGACTCAAAAAGGTTAAGCCATCAGATAAGGGTCCAAAGATAATGCTTTAACTCAGACCAGGAATGAGAACTTGGCCTAATGTCATGGAGAGGGAAAGTTAGAACAGTTTTGAATGGTTGTATGGGAGAGGATGTCTTCTA

>Cs1g04300

AGAACTCAGTAAACCATGCAATCTATAGACTTATTTACAGAATAGACAAGCAACAGTCAAGGCATCTGTAATCTTCTTATAGGCAATAAAGTGAGCCATTTTCAAGAATTGATCCACCACAACAAATATTGAATCCATACCCCTCTGAGTACGGGGTAATTCCAAAAATAAATCCATGCTAATGTCGGTCCAAAATTGTGTTGGAATTGGCAGTGGCATATATAACAAACACACACCCGCCCATATACACAATCACACTAATCACCTAGCACATCATATATATATTTATGTCCTTGTATATAATTCAAATTTCAACAAAAGTATCAAAAGTTAAATTCATATTCCATCCTATATACATGAACCATCAATTAAATAATTTTATTTCCTAGGGTTGGTGGGAGAGGGAAATGATCATGTGACCTCTTGTTTTCACTCTAAGGGGACAAACCAATTCGACTTATTCCGTCAGAGATACAAAAAGTTACTTCTTGCTAATTAATATTGTGGTGACATTAAAATAAAATAGTAACTAGCATATTTATGGACAAATCTCAAATATTTGTGTTATTTTCATAAAATATTTAAGTGTTGTGCAAATTTTAATAAATAAGATATAGATGCACAGCTTTCGGGTTCATGGAATTTGCCAATGGACTTTATTTTATTTTATTCATTAGTTCCCCTTCTTTCTATATATTAAATACATGTTCTTATATAAGAAGTAACAAGAATTATTAGAATTTGATATATCTCAATCTGATCTATTGACCCGGAGAGCGACAATTTTTTTAATATTGTCTATTATTGAAATCACCTCCATTTGTTGACTTTGTATATTTTCTAAGGGATTCGCTTTGATTTTCTTTATTATTTTGGAGATCCGATCTTTTCAGAAGCAATTATGCAAGACACTTATAATAAATATGATGAATTTGGCTGCACTACACTATATGCCACTCAACACTCACTTTGGTGAATAATTTCTCAAAGCCAGCAAAAGA

>Cs1g18360

GCCAGTGGAGTATCCAGCTTAGCCGACATCTTTCTCTTCAATATCGTTAGCTGCTAAGACACTATCCTCGAACGAGGAGGAGGACGAAGAACAAGCAGCAAAGAAAGAATAGGAAAGCCCAGTTGGTGGCTTTTCAAGGCGAAAACGAAACGGAGAAGTGGGTGATAATGATGAAGAAGGTGAAAACTTTACATGGGTTAATGGCTACTGCTGGTTTAGAACTATATGAAATTAGGGATTTTTACGTTTGTGGCTTTATGCTTTTATATGTTCTTTTTTCTTTTTTAATTGCACTGGTGTAACTATGATCGGATTATTCAGATCGACCCGAATTTGATTAAATTCGATTAGACGACACAAATCATATCCACTCAAATTTAAAGCAAAATCCAAAGCTGATCCTAATTTGTACGGATTGAATTGGATCGGATGATTCAGATTTATTCAAATCCTATCCAATGCCTAGGCCTAATTAAGATTTATTGATAAAAAATTTGTTGTTCATCAAGTGCGTGCAAAATTATACTTGAAAAGTTAAATTAATTTAAGTATCTAACTATTTCATGAAAAATCTACATTATCCTTAATAATTCGTTTTTATACAACTTAAAAAATTTGAACGCTAAACATTCATTTATTGTTTATTTTACCGTTAATAGCTTCAGACCACATCGCCTCAATTCCATTAGAACTTGAAATTGGTGGGTTACAATCGTGTTGTTCTTTATGTATCAAAGCGGCCCATTTTTCGGCTTTAACTAAGTACATTTCTAAAATTACCCTTTTAAACCTAATCATTTTTTGGGCGTAAAGGCAAGGTGAAAAACTTCTTTGCCGTGTAAGCTGTAAGTAACTCAAATAATGGCCATAAAAACCAAGACATTAATTATATAATTTATGCTTTTAATCATGCCACTCCGCCCGTACAATAAATAAACATTCTTTCTTTCTCATTATAAATAACACATAAACTAACTCCAAAACTTTATAAAATCATCTTC

>Cs1g06470

CAACATTGGCAACATTATTTAAACATTTTAGAAATGAGCCATTATAACTTTTAAATTATAAAATCTTCACGAATTAACATTAGTCAATTGGTGTTGGGAAATTTTTGCTTTTTAAATGCCACGGACACCAAGTAGCAGACCTAGGGATGGCAATGGGCACCACCCGCAGCGGGTTTGCCATTATCAAACCCAAACCCGCACAAAATTTAAATTACCAAACCCACCCGCAACCCGCAGCGGGTTTTAATTTTTTAAAAAAAATCCACCCACAGCGGGTTTTAACAATTACCAAACCCGCAATGGTATCCGGCGGATTTTTTGCGGGTTTCCGTATTTAAAATCACAAATATTTAATATATAAATTTATAATAATAACCTCAAGTTCCATATAACATATTCAATTTTATATTTAAACAGTGTAAATGAAAAATTAAAATTTCCACATCAATTCAACACAAATTCTCAAATTTAAGTATAAATTATACAAATCTATTTAAAAAACACAAACTAATATCTAAAAATAATAATTACATTTCATATTATCATTTAAAACAAGATCAAAGAGAATATATTTATTTTATTCACCACCATAAGCACCCATCTAACACAATGCCTAAAATAATAATTAAGATTAATATTTTCAAATACTAATTCAAAGGGAAATGCCTTTAGTTTTCTTTAGGTTATGACTTTAGAATAAGAGATGAGCCATATACACACATACACAACTTTGAGCCTTTTGCTATTTGGTAATTTGATTAATTACATTATTTTCTTTATATATTTTTATATTTAAATTAAATTAAAAATATTTGAAAAAAATTATTGCGGGTTTGGTGGATATCCATGCGGGTATCCACCGGGTATAAGGTTAATACCAAACCCACCCGCATCCATATGCGGGTTTTAATTTATAATACTAAACTCGCCCGCAACGGATAAAATTACCCGCAACGGGTGGGTTTTGACAGGTAAATTTAGGCGGGTTTACAGGTTTGCGG

>Cs1g03530

CTATAGGATAAAGTGAGTTTACTAAAAATAAATTTAAAAAAAATTTACTAAACACCAAAATTATCTTTTAACTTTTCAAAATTATTTTTTAACTTTAAAAAATCAAATCCCAAATGAACCTAACTCATTCTTATAGTTCTCATGTTATGTTTCAATTAGCCATTGCATTCATTTTTTCCGATAATAACTAATAAAAGTAGGTAAATAATCATCTATGATTTGTATGTACACATTTGTGTGCGCACACACGTATGTGTGTGTAATTAATAAAAAATAAATTAAAAGATAAAAACACGTACAATCTATTAATAAGAAATTGACATGCATCCTATTACGTGTATCCGATCAATATACAATCTATGAGTTCGTACAGAGCGAAACTCTTATTATTAGAGTGAGACTATTGACACCCCTCCAAATATTTATTAAGACGATTTTAATAATTTTACAATTTTAACCTTTGTTAAAGTTACATAATATGACAATTTCTAAGTAAAACCCTACTCAAAATTCAAATAATTTTTTTCCTTAAAAAATCTAATATTTTAGAAATTAAAATTTTATTTTTTCTTTTAATTTTTAGGTGAGGAATTTACCACAGTTAAATGCAATAAAAAGAAGAGAACTCGTTGTTTTTTTTTTTTTTTGAATGAAAAGTCCTATTGTTGAAATAGGTTTAATAATAAAAATAAACAAAAAGTACAGAATGGGGTTTACTCTAATAAGTTTTAAGTAAGAATATTTTTATCATTAACAGAGGTATTAAAAGAATTTTATAATTTTTTAAAATATTTTAATGATGTTAATAAAGAATATGGGTGCTGAGAGTTATTTGGAGCCACTCTTAGTATTATTATATTCACTTTAAATTTTATGTCATCTTTGACCTCGCCCGCCGTTACGCGTTCGGCGAGGAAAAAGAAGTATAGTTGGAACTCGGAACCGAGGAGTATTATAAATTTAAACTGTAACGAAGTAATTATACAGAAACGACATCGTAC

>Cs1g25210

ATAATAATAATAATAATTGAAAATATTTACTCTTTATTTTGCTAGTGAACAAATAACTAAGGGCATTTTAATAACTTTAACAATTCATTCTAAATTATAAGATAAAGTAAACATATTAATGAGAATATATATCATTTCAATTCTAAATCTCTCAGTTCAAATAAATAATTTATTATGAATTTCACTGCTTATTCCGTTTTCTGCCCATTCTAATTTCGAAAAAGTAAATGCACCATAAGTGCATTATTAGACTATGCACTAACAATATCACACAAAATATTACATAAACTTGCAATCAGTAGAAAGACGAATAATTATTTGATTTCAAATCACCATTTATCTTTGAGAGGTTGGAAGATTCAAGTGAAACTAATACATATTTAAACAAAATTTATGTGTGAAGAAGATGTACAAGTAGAGTAAGAAAGCCCAGGCACGGGCCCGCGGGGAAATACTAAGCCCATATCGTTCATTCACAGAGTAGGCTTTTTGACCCCCCCGCCCCCCCCCTTATTTCCGTATAAAAGTTTTTTTTTAATAGAAATTGCCTAAATAATTAATGATAAGTTAATTTCCTTTATTTTGAATGAGAATTCAATTAAACACTTAAATAAATTATTTCTTAACAATTTCTTTCACACCTGTCTCATTTCATAATTTATATTTATATTTTAAATTTTAATTGAAAATATTCTTTTTAGAAGCTTCTAAGAATTTACAATTATATAGATAGAGATTGCGTAAGAAGAAGAAAATAAGTTAAGATTTTAAGATTTATAAATATTTTATAAATAAAAACTTAAAATAAACTTTTTAGGGTGAAAATTAATGACAAAATGATTAATTTTAATTTTTAGCTTAATCTTCTGTAAATCAAATTGGGGTATTTTAGGGAATAAAATTTAAAAGGGGTGTTCTAAGAATTTTTGAGGGTTGTTAAAAGATCCACTCGCATTCGTAACATTCACATCATACAGCTCAAAGCCCATTAAGGCAGCAGG

>Cs1g10270

TAAAGAGGGTAATGCTAAGTTACATACATGTACCTTACATCCCTCTCATATGAATAGTATATGGATACTATTCATGTGAGAGGGATGTAAGGTACATGCATGTAACTTAGAGGGATCCATATAAAGAAATCGACTATTCTACTCTACGACAAGATCTTATTACTTCATAATTAAAAAATAATAAAATAAAACAATAACAAATCAACGGAAACCAGACCCGGCATGAGTAATAATGAGTTATAACGAATTGACCTTTTAATAACAACATAAATTTAAGCCAAATCACCAAAATCTAAATCGACGTAACAAAATAAAATTTACACATATACTTAAATGTTACGAGTAAAATATTATATGATTTAATCAGCTATCCTGTTTATACATTATTTACTTATTAAGTTTTAACGATCATTATGTTTAAAGAAATTATTTGATATTTTAATGTTATTTATTTAATATATAAAAATTGTAATATAAAATAAATTAGAAATTTATAAGACTAATTTGATTGTGAATTAAAACTATTTCTCAACTGCACCAATATTTAGAATGTTTGAGTCAAACATAAAACAACTCCATCTATTTAGTTTAAATTTTAAAGCCTATTTATTATTTTTTTAGTATGAGATAATTATTTAGAATTAGGATACGATGAATGTAGAATTTTAGGAAATGTATAATAAAATTCTATGATAAGTTTTAAAGAGTTTCTGTATTTAAGAAAATATGGAAAAATATAAGGGCTTTTCGAGAGATTTCGCTTTTTACTAAATTTGGTTGTATCTAATTTTACGTGGTAAAATCAGAACCCCACATCATGACTCAGTCACCACGTGCAAAAGGACACTCAAGCACGGCGTCATGATACTTGCTCCCAACCCATCATAATCAAAACACGTGTCTCCCTTGAGTGACGAAAACCAAGAAAAAGGAAGTCTAAGAGCTAACAATGTATATGCCCGCACCTGATCAGTCCCTCCCTACACCATTAAAAAAAAAAA

>Cs1g03360

TAATATTATGTGTAAAAAATAAGAGTGGTAATGTTAAAATCATGCTCACATGAGTGGAGAGGGAATTAGATTGTTATTATCAAACTATAATTAAAAAAATAATAATGTTGAGTTTTTTTTTTTAATGATCGGATTATAATATTGAGTGTTGTTATACCATTAATCACAATTTCATATGTATAATATATATTATGCAATAGACATTGAAGGCTAAATGAAATAATTTTTAACATGTTCAATTTCTATGAGATAATTTTACATGCATAAGAAGTCGTATGCATATTACTCAAAATATCATAAGAAAACTCGAAAAGATGGTACTTTATGTTTATACATTGTTACTCAAAAAATCAAATTAATACTCCATATGATGATGCTTTATATTATTTTCTACAAAATTAATTTTGTTCTATTGATTTTATTAAAAATTTGATGAGTAATTATAAAATTTGTTATATTAGTTATAATCGGTTTAGGATGAATTGGATTTATAAAAGTATAAAAATTATGCTCATTAATATTAACTTTCCTTATTCAAATAAAAAAAAAAAAGGGCTAACGGTTCCCATTTTTATTTAACATTGTTCGTGTTACACCGAGGGGGTTTGATAATTTTCTAATTTAAATAGCTTTATTTTGAAGTTAGGTAAAGAACAAGTAGCCATCTGATAATTGCCATTGGCTTTGCCATATAATCAAATAGTTCACAGCCCGCCCGTTTCGGTAGCCGGATTTAACTGCAAAATGTAGTGACCGTTGGATTGCCTCATTAGAAATCCAACAGCCAATGAATTTTAAATAAAAAATTCAAATGCCGCTGTATCCTGTATGTGACACAGCGCCGCACGGCAAGATACAAAAGATTAATAAATAATTTAACTGTTAATTGATAGTTAATAAATAAATAAATAATTAATCTTATTTATATATCACCACGAATGAATCAACTGAAGTAATGATGTGGTTTCAATGATATTTATTATTATTATATTCAACAAGAG

>Cs1g10660

AAGGACCAATTGTGAGATTTGATGTGATATCTCGATGTTTAGTTTTTTAATTTATCATTAATTAGTGGTCAAATATTTGTTACATTTTAAATGGTAGAAACACTAAAATTTTATCTCTGAGTCAATATCATTTTTTTTTTTTTNNNNNNNNNNNNNNNNNNNNNNNNNNNNNNNNNNNNNNNNNNNNNNNNNNNNNNNNNNNNNNNNNNNNGTGTGGTCAATTGGGGTCCGACCACAGGTCCGACCACAGGAATACCTTATCCTATCACTTGAGTATGTTAAATAAATTGAGGAGAAAAAGGGGTAAAAAAAAAAAGAAATAAGTAGAAAAATACTTGCGAATGTATTTGATTGTTTAATTTTAATAATTTAGAAAATTTATTCAAAAACTAAAAAAAAATAAGAAATTAGATAAATGATGAAGGGTATTATTGACATTTCAGCAAAAGCAAAAGTCATTTCAATTACCTAACCGAGAGTGAGAGAGAGAATGAGAAACTTGTCTATATCAAATCATAACACTTTCGGGTGGAGTAGTAGTGTAGTACTTCTAATTATCATCAACAATAAATTATTTCATCTTTTCTATTATCCAACAGGTTTTAAGTGAAAAGAGAGAAAAATTAAATAGTGCATTTTTAGAATATCATATTTAGTCCAAATAGTTTTATTTTTTTGTTCATATGAGTAAAATTAGAATGTTAATAAATTACACTTTTCGATGAATGAAAGTATTTTGTGACCTCATTTTTTAAGTCAAGGGTTCGTGTATCATATAAATATTAATTTTTCTTCAAACTTTAAAAAAATATATTTATTTGATCTCGCGTTCAGGTATTCAAACTATGTCATTATCAAATTATTCTTAAAGTTAAGAATTTAAATCTTATTTTAAAGTAAATTTTCTTTTAAATATTTGAAACTCAATATGGGTAATTTAGGAAATATTTATAAAATAAATAAACCAAAAAATTGTAGTTGTAAATGAAATAAAAAAGAAA

>Cs1g21790

GATTTGGTTAGATGCTCTCATCACCGTACAGTAAAATGAATTTTATATATATATTATCGCATACGAAAGAGAAACTTCAGTTCTCGCTTCTCGCTACTCGCCATTGGCTTTTCTTCCATTTCGCTCCTTCAATAGTTTGGTCAGTTATTAATAAAGCCAATAATTTTAGGTCATTGTCGGCGGAACAAATATTGATTATTAATATCAAACGGTTCTTATGTTATGATGGCGGCCCCACTTCCCCGCCTTGTAAATGATAGAATAATTAATTAATTGTCAATAATAAACCCAATTTAATGCGACGTCTCCCCATTTCTTTCTTTTAAACCTCAATCATTGTCAAAGCCGTTTGTGGCTCGCAATTAATTAAGCACTTCCAACTTTACACAAGTTAAAAGGGCGAAGAGTCACGTGTTTCCGTGCTCCATCATCAATGACGCGGATCCGTCATAATTCATAAACAAAATATTTTCTTTATTTCATAACCAACCCAGCCACCCAACAGCAACTATGATTTTTTTATTTTTTTTTTGTAATTTGAATATAAATAAATATGAAAGATTTATTCATAATTAAAAAATTCTAAGTATTTCCAAATTTTTTCAAATGTTATCAAGTATATTCTTTTTACAATTTGGTTAAAAGTAATTTTATTAAGAAGAAAAGGAGTGTCACTTTTTGTTCAATCCTTCTTGATTAAGCGGAAATGTGCTTTTTGATCAAGTGGCGTAGAGTCACGTTCCATCACTGATTTCCACATAAAAAGATGATTGATGACATTTCAAAAACAAAAAGCAGTCTACAGGGTAGATTCAAATATTCAATTCAACAGTGCTGTTTCCGCTGCTGTTGTGGTTGCTGCCCACTCACAATGGGCAGGGGCAGCCCTGTGCTATCATCGCGTAGCACTCATAGTCTGATATTGTTTCTGACTCTGTTGTATCATAGCATCGTGATGAAGTCACCGAATCTTCATCGAGCTACCCTTTATTGAGGCCATC

>Cs1g14180

ACTGCTGTACGGCCAAGGCGGATGTACGGTACAGCAGTCAAATGGAGAAGAATCAACGATGATGTGGCATCTCAAACTTGGCCACATGTCAGAACAAGGCTTGAAGATTTTCTCTGAGCGAAAATTGCTTCCGGGGCTCAAATTGGTAAGTTTACCATTTTGCGAGCATTGTGTTACAAGTAAACAGCATCGATTAAAATTCAGTAGATCTATTGCTAGAAGTAAATGCATTCTAGACTTGATTCATTCTGATGTTTGGGAATCACCGGATATATCCATGGGAGGTGCAAAGTACATGGTGACTTTCATTGATGATTACTCCAGAAGATGTTAGGTGTATCCAATTAAGAAAAAGTCATATGTATTTTCAGTGTTTAAAGAATACAAAGCACGGGTGGAACTTGAATCTGGTAAAAAGATCAAGTGCTTGAGGACGAATAATGGTGGAGAGTATACAGACGGCGAGTTTCTTGGTTTCTGTAAGCAAGAAGGTATTCAGAGGCAGTTCACGGTGGCATACACTCCTCAACAAAATGGAGTGGCAGAGCGGATGAACAAAACTCTTACAGAACGGATAAGAGCTATGTTGAGGACTGCTNNNNNNNNNNNNNNNNNNNNNNNNNNNNNNNNNNNNNNNNNNNNNNNNNNNNTTTTGATGAATTATTTTTCTGATACTTTATACAAAAAATATGATGAAAATGAATTAGTTTAGAATATCAATAAATTTTTATTACATTCAAGATAAGGAGAAATTTGTATAAAAGTCGTGAGAATAGAACTTTTGGTAAAACAAAAGTCGCGGGCTTATTTAGAAAAAAGAAAAAATTACATTCTATTTTCCAATTACTATGGTCAGCCTTAACACATGCCGGTCTTTTTCTCCTCTACTTTCTAGAAATTTTGGTTTCACTATAAAAACTCGGTATATAACAAACTTATGAGTGTTCTTGCATTACCAATCCAACTAAGCACACGCTTACAATCTAAAATACTCTCATCTC

>Cs1g16190

AATTTTGTTAAGGATTGAGATATTTCCACCCCACTATTTTTGTAAATCTCCCCCATTTTTAGGAAATAATTACAATGAGTTGACAATATTTTTAAAAAATATAAATTAATAATAGTTTTGTACATATAATTAATTAAGTCCTTCTATTAATTTTTTTCTTAGATTCTTTGACCATCTTTAATCATAAATTTTAATATAAGCTTTATTTTATTATTTTCTTATTATGATATAAAAGACAAAATTACCATCATAAAACGTGAGTTATTATATCTAATAATTTAGGTATTTAATTGTACGACCTATAATTTTGCAACCTAATAATTTACATGAGAATTATGCTATCAAGACACTATGTTAGTTTAATTAAAAATAATAATCATACTAATAAAGTAGTATTAGAAACAATAAGTTTAGTACAAATTGGAAAAATATTGAAGTTAGTGTAAATTCTTGATAATTTTATTTTACACGAGTAAAATAGTTAATTTAAAGTTATGTTAAAGCAATCTTTGTAATCAACTATACTAAATAAATTTAAGTCACAAAAAAATAAATTAAAATAGATATAATTAATTAATTCTCCAACTTAATTTAAAATTTTTTTAAATAATGTGTAATTTTTAAAATTATTTTTTAAAAATAGGGTGGATTCATAAAAAATATGGGGTAAAGATATCATCACTCTTTTGTTGAGGATTAGTATGGGTTGAGTTGGATTAAATTTTTCTCAATCTACACTGTTTAGTCAACGGATTGACCTCAGTTTTTATCTTAACCGTTAATTACTTTCTTCAATCGTTGTATCCCAAGTAAAAAGGGAAAGGTAAATTGCCAATTTCTGATTCCGGAAAGGTTTTAGTTGTAGCTCCTGACCAGTCGCTTAATTTTCCTAATCCTCACGACCAAACTGACCTCAATTTTTATCTCCAACAACGCAGTTACAAATTGCTATCAACTAAACCCTGCTAAAACCTCATATCAAAATCTCTCTCTGCTGAATA

>Cs1g07740

AAAATATTGTCGGGAATCCTATGACTTTTAGATAGGTGACTTATAAAATTTTTGGTTCATTTTTCTAACCCCTTCCTCAGATGCCCTTTTTAGCTTAAATAGGCCCTTGGTCGTGGGTGGTTACCACCTTTAACTTCTGCCCATTTTCTTCAACAGTTGCACGTGGTCATCAATTGCAGACAGTTTAAATGGATTGTGGCACACAACTTTCCTAGAAATTAGGCCACGTTATCTCAATCGATAGCTTTCTATGTCAAGCTAATTTTTGGGTCGAACAAATAAATTTCGAGATCAGATTTCTTCACTTTCAGGTCGGACCAGTGAATTTCCTGGTCGCTCATATAGGCTTAAGGTTGTATTCTTAAATCAGGTCATTTACATAAATCTCATATCATACTTGTAAACCAGGCCGCCAAAATAGGATCTAGGTTGTACTTACGAGATTAGCTCGACGACAATGAGTTCTGAGTAGATTGGGGTCGACTATATTTCTAAATATTACTAGAAAAATCCAACCCGAACAATGTTGACTCTTTCTAGAGGTTATCAGAAAAATCCAACCCGAACACCAGTCTCATAGTTTTCAACATCAGGAAGCTATTTTCGGTGTTGGACACTAATTAATGAAAAATATAAAAATTCTTATAAATTGAACACCAGTCCCCTAGTTTTCAGTACTGAGAAGATATGAACGGTATTGGAAACTGAAACAATGAAGAAAGCGTAGTATGGTCTCCAGCAGAACAGGGGTAATGTGAAGTAGAACAAGGATAGTGTTGCGTTAAAATAGGCTTAAAAGTAAGAATTTGGCAAAAATAATAATAAAAAATAAAAATAAGGAATAAATAAAGAATAAAGTAAATAAATAAAAAAGCGAGACCATGGAGAAAAAGCATAAGCACGTGACTTAAAGGCAAATTGAAAACATACATTTTGTCTTTTCTCAATACCTTTTGGCTTCTGTTTCGTTTGGGCTTCTGTTTCGTCCTCATCCTCCACAC

>Cs1g01400

TTTGTCAGATGTTTATTGGTATCTTGCCATTAATTCAAGTCGATTTTATACTTAGAGAGGAGGTGTACAAATATATAATATTCAGGAAAATTCTATTTCCAGATGTGGATAATTAAGAGAACCAAATAACAAAACTCGTGTAAGAAGTCTGCTCTTGAGCTAAATGAATTTTTTTTTTTTTGGGTTTTATTTTTTGGATTTGATATGAAAGTGAAGCATATACTAGCAGTTGGATCAAGATGTATGATAATTGTCTATTCATGCTTAAGAGGATAAAAATTGACACGGATCGAGAAGAAATTGAACTTTGAACACTTCCATGATGTTGAAACGAAGGCATACTAGACAAGTCTATGCCCAATTTTACCTAACAGCCTCAAGTTTTTGCTTAAATTTCAGCTTTTTAATATTTCAATCCATTTTTGAAAACACGTTGTGCAAGCTTTTGCGACATGTTGGGCTTAAGGATCACGGATGACAGATTCACAGCCAGGAGGCTGCTGCTAGGGTTGAATTTTCAAATGGGATTATTATTATTATTTGAGTTTTGGGATCAAACATTTTTAAAAGTCAAGTGGTCCTGGTGATGATGTTAAATAATTTTGTGGCAGAATTTGACTATTGACTTGTCTTCCAAAATTTTCCTCATCCCAGGATCCAAGGCACTGACCAACTGATCACCATTCAGCAAAATGTTTGACAAAATCGGAGCCAGCTTTTATCTGTGTTTTATCTTAATTTAGTTCGACATTTTAACTCGGGACAGCTTGAGGTAATTAGCAACCAGGCCAGTTTGGGAATTGGGATGAGCTTAAGTTTGGGTCCAAAGCAGGCCCAAAATTTCAAATGAGCCCAAAAGACGAATAATCACTTAGCTTGATGAATCATTACATCAGTGCTTATATTACCCAAACACGTATGCACTTATGCAACCACGTGTCGCTTGCCATGCAGAAGCACTAAAAATTATAAGCAAACTCCAAGCTTTCGGTGATTCAATC

>Cs1g21390

AATGCTTTCAATTGAGACAGTTATTAATGTCAGACAGAGAACATGTCAAGGTGACTTAATTAATCACGGTTCATTCTTCAACACTGCATTGAAAAGAAAACACAAGACGAATTCTAGAATCTAGATCGCGGCTAATATTTGGGAAATTTATTTCTATGAAATGTCTCGAGATAATGATTCAAGTCTAAAGATAATTTGTTTTTCTTCCTGTTAGAAAAAGATGCTTTATTTAAATACAAACTAAAATTGGTAAATACGGTAAGTCTTAATCGGACATTATTAATTTGTTGGTGATAAAATTAGAGTAAAATAAGTTTATAAAATTTGTATTCTAAATGATATTATACTTAACGTGTCATTCATCTATTAAAATTATAATGTTATATCATTAATTATAATAAAAAAACTCAATAATTACTAAATACATGACAGTAAATAATTAGTGACGTATTATTTGAGTCAATTTTATAAAATTATCATTATCCATGATAATATTATAAAAGGTTGTTAAAAACTCATTAAGAGAGAATCTAATAAACTCTCCACGTAAATAATGATTAATTAGTTTGTAGAGCAGCCAAAGTCAATTACTAGATAATTAATTATTAAGTATATAATAAAGATTAATATTTGATTTAACTAAACTCTTTCGCCCACCAGTTAGCGTACTAAATCCGAAACAACGTCCGGCCCGCTAATAGTATTTCATCTTTTCATTTATGATGCATATATAGCCATAATCACTGCTATGATTAATTGATTGTAATTAACTACTAACAGATTCTCACCCTCTTTCCGTACTCCAAATTAAGCCGGCTATTTCTAAAAATCAAAGGCACCCAAAATAAGCCCTTTTTAGAATTTCTTGGGTTTTATTCTTATATTTTCTTGTTCCTAATTCCTAATTATATTTAAAACGCTAAAATTGCCGTCTATAGTTAGGAATTGGGAACAATAAAACAAATAACATATTAGAAAAACAAAGGACTCCCCCAAACTTC

>Cs1g22965

CAACAACGAACATAAAATATGAGTCCCTCCGTAACAGTGAACATGAAACATAAAACATGAAACATAAAACATGAAACATAGAACATCAAACATGAAATATAGAACGTAAACATGAAACGGGTGCAAAAAAAAAAATTCCGGGGTATTTTGAAAAATCAGTACATGAAACATTGCACAACCAACATGAAACATATCACTTAAAATATGCAACAACGAACATAAAATATGAGTCCCTCCGTAACAGTGAACATGAAACATAAAACATGAAACATAAAACATGAAACATAGAACATCAAACATGAAACATAGAACGTAAACATGAAACGGGTGCAAAAAAAAAAAATTCCGGGGTATTTGAAAAATCAGTACATGAAACATAAAACATAAAATATGAAACTTAAAATGTTAAACATGAGATAAATTTCATAGAAAATGAATAAAATTTTTGTCTTTTTGTTAACACACATTTTATTTTATTTTTTAAAATATTTATAATAAAAAATATTTTTTTTCATTTTTACTTTACTTGTCATTTTTTACTTATTATTATTATTATTTTTTAAATATTATTCGTTTTAAATATTTTTTATTTTAATTTATTTAAATAAAAAAAACATATTAATGGCAGCAACCGGCTGTAAAACCGGTTGCTGCCAGTCAAAAAAGTTGTTTGGCTGCCAGATGGCAGCCAAACAACAAAGGGGTCAATAAATTGACCCCCTCTATGATACAGGGACTTTGAGTCCCTGTATCATAGCCGTGTCCTAGTGTGAGTGTCCTTCTGGAATCTGACTTATTAGTTTTTATATCGATGGCTTTGTGAGAAGACATCGGACGGCTCCGAATGATGAAAAATATGGAGACAGTGAGCTGAACCCAAATCGACGTCATAGAATCAAAGCTCATCATTTTTAGGTCAAATCAGGCTGTATCTCATACAGGCCTTTACTATAAGAAGACACCTTTAATTTAAGGATCACATTGATTATGGTCAAATCAGA

>Cs1g09360

AAGTTAATTTTGGTGTTTGGTAAAAAAAATCAAAATCACTTTTGCCAAAATCAACTCCTCCTACAGCTAATTTTGAAAAGCAGGGAAGGAGTAACTTTTAGATTCTGATTTTGAGAATCAATTTGATCATTTAATATAATTTCATAAATATCCTTACAATTATTACTTAAACCCGAAATTATCCTTATTTAAATTGAAAACAAAATCAATATTTTAATAATTTTTTATTGTAATTAACCCACGCCTTTCGTTTACCCTATTTCTTCATATTTTGTGTTCTATCAAAACTTATCTAGTGCAACAAAGCAAGATCAAGTACGTGTTCCATCACCATATTCAATAACAGTAAGTGATTTATCACCTTTTTCATGCATATTCAATGTAATTTTATTATAATATTTGTTGTACGTGTACATTTTTATATGTATAAGTAAATTTCATCTTACATTATGTATATTTTAGTCATTTATTTTTTTTACAGCAGTTTAACAATAAGATTTACCAAACACCCATAACTGCTTTAAAAATTTACAGTACTTTTGAAAATAAAATTTACCAAACACTTAACTGATTTTCTTCACAACTAATTATTTTTACAGCACAACTAACATTAATTATTTTAAAAACTGCAGCATTACCAAACTGACCCTTAGTTAAAATTGGGTAACTATGTGATAATTACTCTTGATATTTGCCAGTTCCCACGTCGAATTCTTTCGTCCGGTGGCCGGTTTCGCCTAAAATTATAGAAGCCTTGGATTGCTTTCGCAATAATTTTTTTTTTTAAAAAAAACGGCTATATGACGTGCAGCGGCTGAGTTATAAGTGGACCCGGGTTGAATATTTTAGAAAAGAAAACTTTTAGAAAAGTAACATTCTTTTGTTTGTTTGTGATTATCATTTTCTGAAGTTGTTCACATGAAGACCGCAGGTCTGTATGACTCGGTTCTTGTGAACCATGTGACTCCACGTAGTCACGTTCGTTGGAATGATTGAATTGG

>Cs1g26330

ATGAATATGCTTTGATTATTCGCTCATGATAGCTCAGCTGTTTTACATTCTGTAACTCTTTGATTTTAGGTGTCATGTATGTAGCATGCTACCTAAATTTTTATTATATTATTATTTATTGATAAATTTTTATTAATCACATTATCGTTGCAAGGTTTTGTGTTTTAAAAATGATATATGGTGATTTCATAGTCAATGCGATAATATACAATATTTGCATGTATATAACTAAGAAGAAGAAGAAGAAGAAGAACAGGAGGGAAATCATTTATAGTTTCATGTTTTTTAATCATTCCCTTGTTTTGTTAAATTTCTTTAGTCTTCATTTAGGTGTGACTAATTCATGTTTCTAGATTCAAAATAATTAATAATTTTTTAAAAAAAAAAAAATGAACGAGGTTTAAATTTAAAACTCTTCAAAATTATCCGGACAATTTTAATACTGAATTGGAGCGCCTATCACAAATTTAAAACAATTATAAATCGATAAAAATTACTCTTCAAAATTTTCTAAAGCCAGCTGGTCCAATACTGACCCATCGTTGGTGTCAACAACAATTTATGTTTCGCAAATAAAGAGATTGATTATTGATAAATTGATAATCAACAGCGTACTGCATGTGATTCCCCGCAATCTTCTCGGCATATATATTAACAAAGATTTTGATATCTAATGTAATAATATAAAAAATTTCCGTGGAGGGTAATCATTAATTGTTTCTCCTTAGACTTTTCTTGATGCTCTCACCGATCTTATCAACATCCAAGAATAATTCAAAGTAGCTGATCATATATATTTCCTTTGGACAGAAGTCTAGCCGTGTAGCCGAAGGACAACGTTTATTATAGTCCTTTCCTCTCTGCAAAAATTTTGCCGTGTAGCCGACAGACAACGTGTATCGCCATCTAATGTTGAATAGTCAAATTAATAAAGAGTTAAGATAATGGGATTGAGAGCATGCACATGTTGGTACACGTTACGTCCAATATGTAGCTAGCGG

>Cs1g18320

TTGTGGCATATATATAGATGTTATGTTTGTTTGGTAACCTGCATATTAATGCCATTACAAGCACAGATGTTAACTCAATACAAGCGTTTGGTTAATTGCAAAAACCATATTTGAACCATGCTTATATGTTTTATGTAACAATAACTTTTGCTGTTAAAACTTTATGCAGGAGGCATTATACAATATTGCCCGTGCATATCATCATGTTGGACTTGTATCTCTTGCTGCATCATATTATGAAAAGGTGCTTGCTACGTATCAGAAGGATTGCATCATCCCTGGATTTCCAGACCACATGGAGGATTGGAAACCGGGTCACTCTGACCTTCGCAGGGAAGCAGCTTATAATTTGCACTTAATTTACAAAAAATCAGGGGCTGTTGATCTTGCTAGGCAGGTCTTGAGAGACCATTGCACTTTCTGATTAATTGTTTTCACAATGACATCAATAGCTTGTATATGATGATAGTGTATTTTATTTTATTTATATCAATTTTAAATTCATTAACCGGCTTTGGTAATGCATAATGATCAAGGTTTTTCTCTTTTCATTTTTTGATAAATTTATATATTTTGCCTTACTTGGAGAGAATTATATAATTGAAACATATAAAAATGTGTGTGTGGAGAATGTTAGGAAGTGAAAAAATACTCGGTACCTGACACTGGCTTATTTGTCAAGGATGGCTCCCTAACAAACGAACTAATTGTGGCTTTATATGTAGCTACTTTATTTGCTAAGTTCGAATTTTGTACATACATACACATGAGGAATATTACATATGCATGCAAAATAGAGAGTTTTGCCAAGTTCATGCAGTTATGTAAAGTTGAATTTTTTTAAAGAAAATATGTGCATGCAAGGAGTCATACATTATACACATTAGTAGTCGTTTTTATGTCCCAAACAACATAAGTGTGAAAAGAGTCATACATTATACACATTAGTAGCTGTTTTTATGTCCCAAACAAAAATAAATGAGGAAAAAAAATTAACATAC

>Cs1g14780

NNNNNNNNNNNNNNNNNNNNNNNNNNNNNNNNNNNNNNNNNNNNNNNNNNNNNNNNNNNNNNNNNNNNNNNNNNNNNNNNNNNNNNNNNNNNNNNNNNNNNNNNNNNNNNNNNNNNNNNNNNNNNNNNNNNNNNNNNNNNNNNNNNNNNNNNNNNNNNNNNNNNNAACCGAACAACAATTGAAGGATACGAGACAAGCAAAATCCCCTAAAAAATACTAAATTTTGAAGCGAATCCTCGACAATTGAAACCCTAGAGAGGTTAAATTGAGATTTTGAAGGATTGGGACCTAGGTTTAGCGATTCTTGAGAAACCCTAGATTGTCAGGGAACGAGGAGAATTTTTTATAATTTCCCCTTTTAAATAAAGACTAAGGATATGTCGTTATCAGACATCAGTTGAATGGAGGACAGTATTTATATGAGTAGAGTGAAGGTAATTATCCTGACTTGGTGAGGGTTTCTTTGGAAATATGAAGAAAGTTCGGGGATTGGTCAGAGAGTGACACGTGGAATGGGAACATTGTTTAGCTTCTGGCGTAAGATAATAAGATATGATACGGTCACTAATCACTATGAGGTAGTACATTTGCTTGGCCACAGCCAGTCAGCCACAGGCTTATTAACGCTTATTATATTTGTTTTGTAGTTGTGTTTGCTTCAAATTGAATCTTGAATCTTGAATCTTGAAGTTGAATCTAAGGCGTGAAGAAAAATGGACCAATGAGATTATGGCATAGAAGCATCTAAAAAAATATTATAATGTGTTTACTTCTCGAATTGGAAATCGAAATTATTAACATCATTTTACTTCATAAATAAATGTTCAGTTATGGTGCAACTATGGTACTATACCACAATTACTTTTAATCATTATATCAAGTCATGATGATAACTGTAGCTGGATCCAATGGTACTACAGTTGCACCATAATTTTGTCTCACAAATAAATATAAGAATTAGAATCCAAATAATAAGACCCACATCAAATTAGAAATAGAAA

>Cs1g05450

TGGACTTTAAACATTCTTCACAAGAAAATAAACTCCACCCTTGCTCATTTCATAGCTGTAAAGAGAATAATCAATACAAGGAAAAAAAAAAAAGTAACATCTTTAGTCATCAGCAAGAACTAACCCGTGATTTCTGATTGTGTTATTCGATCCTCCGTTGGCAGTAAAGTGTGCTTCCTTGAGCTTCACATTGCTTGAGGAGAGAAAAAGAAAATTAGAAGAACTATCTTTTTGTTGCGTTCATTTAAGCTTTACGCATTGCATAGAGTTTAAAATCAGATGCCATATCACGATAAAGTTAAAAAAAAGAAAAAAAAAAAAAGAGAAAATGATAACACACTGATCTTTTCCACTATTCAATAACATTTTCCGACTAATCTTAAAGATCATCAATCTCCTTTTACTTTTTCTTCTTCTCCCCCCCCCCCCCCCCNNNNNNNNNNNNNNNNNNNNNNNNNNCATATTTTTGGACCGATAATTTTCGTCCATTTATCTAATTTCCCAACAAAAAGAGTGAGAAAAATACACCGAAAACAATGTCATTTTGAGTGTGTGTTTTAAAATTAAATTTAAAAAAAAAAAACACCACCAAACAATGTCGTTTTGGATGTAATTTAATTTTCACCCTTTTGTCAAAGTTTTATTTAAATAGTAGTGCTTGAAATTGGTAGGTGAACCCTCCAAAATCACTCTCAATATTTTGGCTCACATAAGATACAGCCCAAAGTTGCCCCAAGCATCTGGGAACTTAATTATATGTAAGTTTAATTGCTTTTGTTAATTTGCCTTCTGGTTTCTTCAGCAGATATTTCTTGCTTCACTTATAATTTTTTTTTTTTTGGCCCCCTCCTAGAGGTTCTCATATTTACCTTCTTTAAATAAAAGAACACTAGAAAAATGCCAGCTTCTTGTTATATTTCCTCTTTTGTTAGTACATAACATATATTTGTATTCACAATTATTACTGAGAAACTTTTATGTTGTTTCTCATAAGAAAACAA

>Cs1g12290

CCCACACATGCCGTGTCGTGGGCCGTGCCGTGCCTAGGAAAAATAGCCCATTAAGCTTTTTTCCTTTTTTATTTTTTTAAGAGCGCATGCCAAGTTGTCTTATTTTTGTCAATCTTATGTACTTTTTTTTTTGAATTTATGTGCCTTTATTTAAATTTAATAGAACAAAAAATTTAAAACTTAAGTCCATATTCAATAATAATAAATTAATAATAATAAGAGAATGTAATATTAGTTGCTAAGTTTGATTGTATGGTATTAGAATTCTTTTTTTGTACTTAACAACAACAACAATAATAATTTTTTTTAAGGGTTAACTTAATTGTTAACTTAGAATTACGTAGAGTCATATATCATAATTTATAATAATATTAATACATTTTTATAAAATCATAATAGTTATAATTTTATTTATTAAAATCATGATATCAATTATTTATTTAATAAATTATAAACATAAATCAATTATAAATTTTTTGAAACTAAGAATTAAATAAATTTAAAAAACTTGAGCCAATAAAATATATTAAAAGATTCAATTTCAAGTTATTTATAAAATCATAAAATTTTAAGTTTACTTTCATTAAAAAATAAAACGTGCTTATTGCGTGCTTTTTCGCGTGCCGTGCTTTGGCCCATCTCTAATCGTGCCGTGCTTTTAAGGTCCATGTGCCTAAAATCTTAGTTCGGCACAGCCCACATGCGTGCCGTGCCGTGCCGTGCCACGTGCTCTACCAAAACGTGCTCGAACAATGCCATGCTGTATGGACACGTGCCATGGCTCGTGTCGTCCGGCCCATTGACCACCTCTAATTCAATCTTACTACATCAATTTGTACAAATATTGTTTTCAATTTGTACAATATTATTTGTACGCTTGTGGCTTAAATTATTGTAATGAAAATGAACAAGCTACTATCTTCACCCATGAAAAAATAAAAATTACGATTTTTCTTTTTGACCGTTGTTGTTTAAAAAACAAACCCTAAAGATTTTTGTTC

>Cs1g21360

CAGACTAATTCAGGTCTATTCAGATTTCAGACAAAAAAACAAACGCCACCTAAGTTAATTAAGTCATTAAGTAAAAAAACAAACAGCCCTTTAATTGATTGAAATTAAGTCAATTAAGTCCTCAACTTAAAAAAATAAACGCTGCTTAGTTGAAGTCGTGCCTCCAACAAACGGTAGACTTCGGCCGGTAAAGAAGTTGAAAATGATCATGCTTGGAACATAAAGAATCATTCGCTTTTCGTAATGATCATTAGCTTTTTTATTCCAAGCACTTAGAATTTTTTCTAGACTATACCTACCGGGAATGAGGACATTTTAAGTTACTTTATCAGTTTATTTTATTTTATTTGTTTTAAGGGGTCATCTGAGACTTCTCTGACTAATTTCTATTCTTCGTAAGCATTCATGCAGGCTTTAGCTTATAATAACAAATGGATCTCCGCAACAAATTAATTTACTAATTTATGAAAAATCTAAAATTTTTAGGTCAATGGTTTCTTGTTCTGATGACTGATGGTCAAGAGGTGAATGATCTGTGTAAGGAAAATGTTTTTTTTTTTTTGCCCTTTAACCCCTTGTTTAGTTATGTACAATATATAGACCTCAACGAATCTATCATTACAAAAATTTAGACAGTCTATCATTCGAGGTTTTAGTTCATGCTATAATTGCATGCCTGTAATCCATCCAATATATTAATAATGCATCTTCTGAATACAATATGTATATAATTACCTTATATTATGACATTTTACCTAAAAGTAACTTAGTCAATGGAGCTAGTGAATCCAATGAAATTTAATTAAGGCTTTGGATACACGCCTTATCAACTTACTTGCCAATTATGGGCATTAGCAGACCTAACGAATTGAACCTGCTCTTAGTTTTCTTCTCTTTAATCATCTATTTTCCTTAAATTCATTTAAAAGCAAAACTATCTTCTTCTTCACGTTTCACTTGCTTCCCTTGCCGTTAGGGAATGTGAAGAGCATGAAATTAAA

>Cs1g05470

CTTGCAATTCAATGCTTCACACACAAGCTTGGGTTGACATTTGGCCATACATTAGTGAAGAAAGTCTGACAAAGCGGTGGTGTTCAAACACAAAACTTCATCAGTTGACTCAATTTCCAATTGTGAATGCACGGAAAAATGTTGCGTATAAATTGACTTATGTGAAGTCTTGGTCTGTGATTCCTGCTCTAATTTATAAGTCAAAGAACGACTTGCACTGCGGTGCCTCGCAAGTCTGTGTTGATTTTAAAATTCTCTTAACAAGAGGTAAAATTGTAAGAGATTACCCCTCGAGAAAATTATTATCTTAAAAATACTTTTAAATTATTATAAACCTCATTTTATCTCTATTTTTTTAAAGAAGATAAAAAAAAAAAAATTTCATTACTGCACCCTGATTTTTTCTTTATTTTTTAAATAAAAATAGAGTAAATTAAAATTTTACAATAATTTAAAATATTTTGAAGATTACCATTTCAAAAAGGGGGTAATTCGACCTTTTTTTTTTTTTTTTTTCCTTTACAAGAGTCCATTCTATGCCCGTTTAGAAAAGAATTTCAATGTCGGAGTGTCCAACGTTGTTAAAAAAATAAGCTCAATATGCGATTCTAACATTTTAAATTCCCATGAGCTCTCGTTCTTAAGGGGATTTTTGCTGTCGAAAACCATACTATTAAGAATTGGCCATATATAGAAACCACATAATCAACATTTGTAGTCGTTGACTAATTAACAAGAACAAATAAACAACTCAACAGATTTTATTATCTCAATTTTTTTTTTGTTATTTATTGTAATCAATTTTATCGCTGAACTCTGATTAATTTTTTATTAAGTGTATGTGTGATTTAAATTTTTTTTTATTGATTTGTAAATTGCTAATTTCAATCAAACGTCACATGAAAGGCATGCAATAGAAACTTTACGGAGGTTATTTTCGTCGTTTTACTCGTTTTCCCAAAACGGCTAGTACAAAAATCAGCTGAAAGGAATGCCTCAAC

>Cs1g10425

AAAAGACATATTTTTTTGGTGCAAAAATTTCCCTCCAAAAGTGAATATTTTGTGACAATATTTCACTATTTATGACAAAATAAATGGTCACGAAATATAATTATAAAAGACAACTATTTTATAACTAAAAGAGAAGTGTAAAATTGGCCAAACAATCTGCAACACAAGATATTAGTCTTCAAAACTATTGTAGTTTTGGCTAATTTTAGGACCATATTTTTTGTCATATATAATTACCTATGATGACAAAACTAAGAAATAGTAGTTGAAAGTTTTGTCTTGATTAATTTTATATTTTATAACTAAATTTGTTCCTTTTGAGACACATTACTTGGTAGCTAAAAAATAGGATAAAATATTTTTTCTTTTAATAGTTGCAAAAAGATTTTTTTTGGGGCATAAAAAAGGGTCCAAAATTAGCTATTTTGGGACAACATGTAATCTTTTATGACAAAATATATAGTGGCAAAATATAATTATTTTGATAAAACTATTTAGTAGCTAAAAAAGAAGTACAAATTCTATCAAACTATTTGTGACATAAAATATTAATTTCAAAAACTATTATGCTTTTAGCTAATGTTAAGACCTGTTTTTTGTTGTTATGTATAACAATCAATCTAAATAAAATTAAGAAATAGTAGTCAAAAGTTTTGTCTCAATTGATAATATATTTTGTGGCCAAAATATTCTATCTTTGTGACACTTTACTTGGTCTCTAATTAAGCTTTTTTGTGATGATCAATAGAATTACATAATAATTACTAAGTTTTTTAGTTAAATTATATATTTACTATTAATTAAGTTGTTGGCAATAATGACTAAATCAATACTAATTCGGAATACCATCAACAACCATAATACTAATGAAACAAAAAATTTTAATATGTAGTGAAAAAATTTTAACAACTGTCCCTAGGATTTAGAATTATTTTGACTAATTATTCTAACAATTTTATTTAGGGATAAATTTAATAATATAATTGCTCCAATTTTAATTG

>Cs1g11770

ATAAAGAATAACAATTAAAGCTTATGCATAGTTATAGATGAGGAGGGGAGAAATCCATCCTAATTGTTATACAAATATTTACAATAAAAAATATATTCAATCTTCTTAGACCAATTTTATAATGATGTATTGTGAATGTGTGAGTTAGAAAATGTTTATATATACTTCTGAATATTTAAAACAAAATTAATTTTTGTTAATCTTCTACATACATGAAGACAAAAATAATTTACTACTTGAATGATATAGCAAAAATATTTACTTCTATATATGAAAACAAATGTTTGAAATTGGTCTAGAGTAAATCAAACTCTTGCATGATCATCACCATGCAAGAGGTTCCTTCGAGTAGGAATGGCAAAAGTTCGGGATCAAAAACATTTGAAGCCCGACTCTAAATAGATCCGAATATTTTATAATTAAGGTCAGGTTAGCTTCAACTCGAATCGGGCCAACATCAGGTCGGGTCCAACCCCACCTTACCAAAATAGTTTTCATATTATTTTAATGTTAAATTATTTTTTAACGTATTGTTTTATATTCATGTTATTTTAATATTTTCTTCTATACATGCACCTTAATTAACAAGATAAAGGCAAAATATACAATATAATTAATCACAAAATAATTCACAAAATAACCAATTAATAGGAATAATTTTTTAAATTCAATTCCATAATCAAACTTTAAAAACACAATGTGATTAAATTAAAAAAACACGATTGAATTCCACAACAATTTCAACCCCACACAGTCACACACAAATCAATTTGAAAATCTAACATGAAAAACACACAATGCAATTAAATTAAAAACTCACAATGTACTTCAAAGAGGGTCGGGTCAAAATTTATTCGGGCCTAAAAATTTTAACTCGACCCTAACCCAAGACTCAAAAATTCGGATCAAACCTCTAAAAATTAGAGTCGGGTCAATCGAGCAAAACACAAGGTCGGATAAAATTGTCAAGCCTACCTTGGAGTCCCTTTAAGGGTCTGATG

>Cs1g10030

ATAAAAATTTATTTAAAAAGATATACAGAACCACCTTAAGACTTTGTTTGGTACAATTTTTCAAATAGAGCTTTTATTAGTTTTACCAAAACAATTTAGTTATTTGGTTGTTAATATAAGATTTTATTAAAAAAATTATGAAATTACTTTAATAGGCAAGTTTTTTTAAAAAATTATGTTATTAAAAAAATAAATTAAGATTGTAAAATAAGTAAATGATATTTTAAATATTTTAACATTTAAAAAGAATTTCAACATCCTCTCCCAAAAGCGTAAAACTTTAGTTTTTACAAATAGAAGCAAAATAACTTATTTAAGCTTAAAAAACTCTATTAGAAAAATAATTAAATAATAACAAAAATTATGATAAGAGCTTATCGGATTAAAGGTTCGTTGGGGATTGAGGTTGTGCAGTTGTTATTTGGTAAACACTAGTTGATGTACCTTAAAAGTTATGTTAATATAATTTTTTACTTATACGATAAAAAAATTTATTTTATCTTTAATAATTTTATTAAAATTATTATTTAAAATTTATATTTACTATATACTATTAATTTATATTTCTGCTGTACGGCCAAGNNNNNNNNNNNNNNNNNNNNNNNNNNNNNNNNNNNNNNNNNNNNNNNNNNNNNNNNNNNNNNNNNNNNNNNNNNNNNNNNNNNNNNNNNNNNNNNNNNNNNNNNNNNNNNNNNNNNNNNNNNNNNNNNNNNNNNNNNNNNNNNNNNNNNNNNNNNNNNNNNNNNNNNNNNNNNNNNNNNNNNNNNNNNNNNNNNNNNNNNNNNNNNNNNNNNNNNNNNNNNNNNNNNNNNNNNNNNNNNNNNNNNNNNNNNNNNNNNNNNNNNGTATATACTATTAATTTATATTTCATAATTATAATTTTTTTTCTATAACAGTTATAAATTAAAATATACACTATCTCAATTTTAAATAAAATTTAAATAAATATTTATAACCATTAAATAAGTTTCACCAAATTTATTTAAATTAAAATCATTTTA

>Cs1g26180

GTATTTGTCATAAAGAAATTAATTAAAATGTATATATAAATGGTGACGTCGGTTGGATAAGTAAACATGGAAAAGGCTATAAATGGATCACTTGCATGAACAAAATCATATCTTAAATGAGGAGTCTTGATTGATTTGGATGTTTCACAACCCTAAAATCAATTACATGCCAAATAATATTTAATCATTGTACTTGAATTTTATATCGCGAGGGAGGGTTTCAAGTTCGGCGAGCTAAAAAAAAAAAAATGGAATTGAAATGATGCTGCCACTTCTTTGCTTCTTCCTTTTGATTATATTGTTTGTTGGTAACTTGTTATTCTCTAACACAAGGAACAGAGAGAGGGGCAAAAAAAAAAAAAAAAGTTGTCAACCAACGAAAGAATCATAATATATATGCGTAATTTTTTAATGTACGTATCATAATTCTATTGGCTTATTAAACATATTTCCAGAGTACAAACTTTTTTTTTTTTTTTTGGGGGGGGGTATGTTTTAAAGTGGTGCTATTTAGACGCCTCTGTTAAATTTATAAATCTTTTAGGCATTAGAAAATAATTAATCCATTTTCTAACATTTGTCATCCATTTTGGATAACTCCGACTCTGATTCAATCCTGAAACGGGCTCCCTGCTACCTCTCCCTGCACCTTTTTATTTATTTATTTATTTATTTATTATTAAAAAGTAATATTACATATTTCAAAATATGAATTTGTATTTTTATGTCAATCAACCAATATACATACGGTACACAAAGTTTAAACAATTATTAGAAAAATAGATTGTTCCTAAAGATTAAGCTAATTAATTGATAAGCACGAAATTTTTGCTGGCATGGTGGCCAATTTGTTTGTTTGTATGGTAGCATGTCACCTCCCTCATATATATAATGAAGGGGGTGGTAGTAATTTCCAGTTGCAAGAAACAGTGGGGTATTAAAAGAATAATGAAGGGGTGGTAGTAAAACCATTAGAAATCTTAGAATTAAAATTGGGCTTG

>Cs1g05900

GCTAAATTGTTTTTTGCATGATTGTGTGATTGAAGTGTTAATGTTGTTGTCATTACTTGAGGACAAGTAATAGTTTAAGTTTGGGGGTGTGATAACTCTATGAAATGAGAGTTATCATGATATTTTTATACTTAAATCAATATTACTTAGAGAGTAAATGATGTGTTTTTATTACATTTTAGTTACATTTAGCATAAGTATCCTTTTAGTTTATTTTTAATAAATTGAGTTGTTTTAGATCATTTTGTGCTTAGATTATATGCATGATTGTAGGTGACCGAAAAGCTCAAGTTTTAGCGATGGAATGCTACTGCAACTGACCTACACAGATGATATAATAACCTGGATACAAAGGAGTTAAAACAAGTCTAGAATAATGAAGATGTTGTAACCGTTGCTGCAGTAATCCTAGAGTAATGAAAAAAAATGGATTTTGTGTGCAATAACTAAAAGATTGCAATTTGAAGTTTCTAAAAAATAGAGGATACGCACCACAAACTTTCGTTTACCCTAATTCTAAGTTATAAAAAGAGTACGCATGAAAAGAAAAAGAGAGAGTTGTCACCCAAAGAGAATAATAGATAAAAACAACAAGGAAATAAAAAATTAAGATAGAGATTTGAGAGAAATAACCCAATAAGTTTTTGGTGCCGTTGCTGGGGAATAATTGTTCATTGTGATTTATGAAATTAATCTTGATCTATGCTAATTTTTAATTTAATTTTAATTTGTTTATTTTGTTGATAGAAGTGCTCTAATAGCCTTTGATGTGGAAATATAAACCTATTGAATTCCAGTTTAATCTAGAAATTTAAAGAACTACAAAGAGAATGAGAAAAGAGTAAAGAAATTCAAAACCTGTTATAGCAATGGATGATTTGCAAGGCACGAGGAATTTGAATTCTCGAGGAGAAATATAACCAATAAATGCCCAAGGAGGTCAAGAAGGTCAGAATGGGCAGATTATTTATGGGCAAATAGGGAATAACAATATTATTCAT

>Cs1g08500

ATTTCAGTTTGCGCTATTGTTTCACAACTAGAGCTGGCAAAACGGGTTATCGGGTCGTGTTCGTGTCGTGTCAACTTCGTGTCGTGTCAAATTTAGGTCAACCCAAACCCGACCCATTTAATAATCATGTCAAAATATTCAGACTCAAACCCGACACAGAAAAATAATCGGGTTACCCAATAACCCGCTTAATATCTATATATTAAACAAAAGTTACATTAAATATTAACAATTTATCATACATATGTATGCACATTCATACATCCATACATACATACCAAATTATTTTAAAATAATCGGGTTATTGATCGGGTAAATGGGTCAAGAATTTTAACCCTAACCCGACACGGAAAAAAATTGTGTTGACCCGAACCCGACCCATTTAATAATCGTGTTAAATTTGACGACCCATACTCATTTATTTCAGTGTCAAATTTTGCCAGCTCTATTCACAACAAGCGTGTTACCGTGAGATGAGATATGTCATGTTTAATATAGGAAAAATAATAACTCTACAACGTTCAAGTTATAAAAAATGTAAGATAACTAATTACTATTCAAAATAAATACTAAATCAAGTTAAATAAATACCATAAACTTGATAATATTTTCTATAAATAAGGAACTTCTTCCTAAATTAATAATTGGTTAATTTATCAGAATATTAATATTTACTAAATTAATAGATTTTTTTTTATAGTTTCAATGTTATTAATATATCAAAGTTTTACTAAATAATTGACTATAGTAAATAAAATATGGCTCTATCATTCCTTGTTTTTTTTTCTTAAAATTAAAATCTATATTAAATCTCATCTCTTATTTTTTTTATATTGCATCTAAAAGTTCTAAAATTACAACAAAAAATTGTGAACCAATAATTTAAGTGATTCCTTGTGCATGAAAACTATTATGATTTAAATGTATGTTTATTAAATTTTACATAGACAATATACATTAATGCACTGTTACTAACAGATATTAATTTACCGAGGAAACAA

>Cs1g06550

TCAGTTTTTTTAAATTATTATCTTGTTTTTGTTAAATTTCTTGTCTTTATTTATGTATGGCTAATTCATGTTTATATAATCAAAATGACTAATAAATTTTAATTTTTTTAAACAAAATTTGAAACTCCTCAAAATTATCTAAATAATTTTAATAATTTTAATATTGAACTGTGGCACTATCACAAATTCAAACCAGTTATGAACTAATGTCCTACCTGGTCCTATACTGGCTCTGCAGTGTATGGAAGCTGTTATGATTTTGTCAGAGTGAATATTATGACTAAATAGTATTGTAGTTGCTTTGATACGGAAGGTCTATTTTTCTATTTACTAAACGGACATAATCTATCGAGAGGCAAATGCCCTGTAAACAAGTTGGCCAGCCATGGTCCTAAAATGCAGAGAGCAATTTTCCCATTAATCATTAATTTTGATCACAAAATTAATTCTGTGTATGTCGTCGGTTGATCTTTTAAGCTCTTTTGGAAACATACATACAAATCTTTGTGGAAAAAGAGAAAATTAAGATTTTTTTCTTAAAAGCCGATGATACACAAAAAGAGAGAAAATAAGAAGAGGATACAAAATCTGTACATGTGATGAATTGTTAGACATTAAAAATATAAAATTGTTATCTTGAAAATACAATTTATAAGGAGTAATATTCTAGGATATAACGGTGTAAATTAATAAGGCCTCAATTGACGGATGATTAACCAACAAATAAATGAATCTATAATCTTTCCCTAGCAGCAAGTCCACTTTAGAGGTGTTGAAGACGCATTGACTTGTTGACGTCGCGTTGATTCTAAATTTCTAACTCGTTTGCTCTTGTGTGTAGTTACTATAAATTTGGCTTTCCGTCTGTTTCTTTGTACATAATTCTTAGATGTTTATTTCTACCTTGTCCAGCTAATCTTTAATAATTCTTGTTGTTCTCATCTTTACTTGTTCTGTGCTGTTTCTGCGACTACCAAGTCTCAGCTGATTTCAATTACGGA

>Cs1g19290

TTTTGTCTCTTGCTTTTTTGAAATTTCAAAATATTAAAAAATATTAAAGGATAATGAGAAGAGGGAAATTTAAAATTCATGTTCTAGGTTTTTTTTCAACATATATCGCACCACGTATCTAACTATAATTATTAATTAGTTGGTGTTTGCACATTAGATTATACATGAAACCTGAGATGCATTGTAGAATTTCTCCATTTGGGGATATGTGGTTTGGTCGTCGACCGCAAGTAACTTCTAGAATATCACAATTTTCTTCTCCTCCAACCAACTTCATAGATGATCAAAAACAAAATATTAGAAGCCAAAACATTATCAAAGATGTAAAATTATTTATGCTCTCCATTTTCTTTTTTTTTTTTTCAAATTTGGACTACTAGGAAATTAAATTACAATACATATATATATATCTATGGATATAAGAATGTGCAGTTGAAGAGCTACTTAATTATATAATAGAAATAATACTTTAATCAGATATTTAATTGATTCAGTTAATGGAGTTTGACGCTCTGACTATGCTAGCAAGATGAAATATTGTATACATGTGTTATGGATGTGTTCATACTAACACTAAGGATTGTATGAGATAATTTATGAGTAACTAAAATTAAGTAGTGGTTATGGTGGAGTTGTTCCACCGAAAAGCATTGATGATAACCGTTAGATCATAATAATAGAAATATAATTCTGCTATAGAAAGTATGGAATATAAATCATAACATACTAATAAGAAAAATTTAAAAAAATAAATTCAAAGAAATGTGAAATTGAGTATTTAACTTTTAGAATATGAGAATATAGTGAGAGAATACAATTACAAAGTAGAAAAAATTAATAAAGGATAATTTCAGATTTTTTTAATTTTTAATATTTAATAATAAATTTATTTTTAAAAATAAATATAGAGAAAATTTTAATAATCTCAAATACCACCGCCAGAGGAAATGATGTATAAGTCTGTAGACAACATTAAACGCAGCCAAACGTTGACCAACTAC

>Cs1g03680

TGGGACCAACTGACATCAATGCTAATGGAGCTCTGCCCAGATTTTAACCCTCACAAATATTCGAGGGATGTCTACTCCTCACTCATGGGTAAGGGAGAAGACTACCTTCACTCAAATTAATTGAGGAAGGAAATACCCCCACAATGCTTTTGTGGGGGTTCGAACTGCTGCCCTCCAAGTTGGAGGGCAGCAGCCCTGGCCACTCGAGCTGAGCCGAGTGGTTAATGCTAGGAGATAACTAAACCTTGTGTGTATTTTATACATATTTATTTTGAAATTGAATGTCATAAACTGAAGAGTATTGGGTTGTTATCAACAGCCTTTCCTACAAAAAGAAGAAACTTGAAGTGGGAGTGGTTGAAGAGTAATAACTGAAAGGAGGTTGAAATTGTTTGCGGAAAAATGACAGATAAAGGCTATTTTAGGAATTGAGGAGGAAATAAACAGAAAAGGGGTTTGAAAATGATATCGGAGGGGTGCCGATAGCCTCACTCCATTCTATTTATTTTGTGCAATCTTATGTACAACTTCGAGTGCAAATAACATTTTTCAAATAAATTAAGATACAAATATTAGCATTGGAAAGTTTTATTCTTATTTAAGCTTTATTTGCCACTGCTTTTATTTTTGTTTATTTTAAACAGTAATTTAGTCAAGTGAATGTGAGAGTAAGGTTCGAGTCTTAAAATTACAATATGAAGACTTAATTTTCTCTGTCAATTGAGTGAAAATAATTTTTTTTTCTTATAAAATGTGAGTAAAACAAATATCAGATACTCATAAGTTTACATTAATTTTAATAATTATTTGATTTATAAATATCAGTAGCTGTCTCAACTACTATAAATAAATATTCAGGTAAAAAATCTGGACCCTAACAATTAAAATATATTAACTAATTCTAATAATTGTGTCTGACCTATAGCATCATTAATATTCTCATTGTAATATCAGTATATATAATTTTTTTTAAAAAAAAAATGACAAGAATTGACTAAGAA

>Cs1g19830

CCTAAACAGTTAAAAATAGAATTAAAGTAACTTTTATTAAATTTTGAAACATTTTTTATAACCAAACGGATAAAGATTATCTCTTTATATGAGCTCTCAGTTTTTAATGATCTTGTGCCATGTGTATTTAAGTGATAATGTATGGATAAAATTTAACTTTTTCATTTTCTTTATTTATTAACCACCAATCACCCATGTGTATTTTTACTCAGAAGAAGATCAGATCAGATCATGAGAGAATCCTAATCCATAACAATAATTTGGTGTTCTTTTAGTAGATATCAACTTGAATTAACGAGTGAGAATCAAGATTGGACAAAATAAATAATTGAACAAACAATTATGACCAAAGAGATTGGAGCAGAATTCTTAACTCATTATATTTATTTTAAAACAAATTTTTATTTTTCGGGTTTCCCCACATTTTCCAACCTACTATAACTCTAGCTGGAGTCAATTAAAAAATAATAATAATAATCAGATTAGGAACAAATTCACATTTATTAACAAGCCCCGAAAAAATTGCTTTTATTTTTTGAAGTACTTAAAAATTATTGCTAAGTTGAAATTTTATGAAGCACTAAACTAATAATTACTAAATGACAAACATAAAAAATAAAATAAAACAATTCTGACGGAGATTTAGAAGACAAAAGGGGGGGGTTGGGTTGGCAATGAATTGAAGAATTCTATATAAGTGGCGCCAGAAGTAGACTGGTAGGTGAATGAATAATCAGAAGTGTAACGACGTAAAGGTGGACCAAACGACGGGCGACTGAGCTGATATCATAATCGCCACTCATTTGCCAAAATAGGCGCATCTGGCTATTCCCATGTGCCTATTATAACATAACCAGATCATAACTCCCAAAATAGCCCCCACCCCATGTGACTTGTCCCTCTCATCTCATATCGACGACGACGCATTTTCCAAAAGACCACTCTTACCAAAATACCCTTCAACGAACTTTACCTCTCCACCGAACACAAACTCTCGGTGC

>Cs1g09510

GAATTTAACTCATATATACTCAAGAGTTAACTCAATAGTTGAAAGTTACCTCAAGCTTATATTATTAGTCCAATGTTATTCTCAACTGATGAGAGATCTATATTTTCCAATATCATAGCTGAATTTTATTTTTGCTTGATGTTATTTAGATACATAGCTTTAAGTTGGAAAATGCTGACTCGCCCAGTAATTTGCACTGAATGAACATAGAAGGTGCTATGCATCCTAGCCATAGATGGCATGCTGATGATGTTATAAGAGAATTAGTACAATATGTTTATTAATTTATATGCTCCATCTCATTCCTAAGATAAGATCAAACCCCCTATCCTTCGCTCTGTTATAATCATTACAATTATTAAATTAAATCGCTTATAATCATTACAATCTACTGCTTTCTTCTACCATCGTCCAATATTTTTCACTTATTGACATTCTAATTAATTGCTTATGGTCGGAGATAACTACGAACATAAAACATTTTATGTGAATAAAGATGCTTTAACGTGCTAGTGGAGAGAGTAAGTTTGACATTGTTCTATCTTAAATATCAAATTAATAATTATATATCAACATGTATTCTCCAAAAATTATTTTCAAATATAAATATTCTTAATTTTTCTATTACATACGGATTTATTTTTTAAACCCAATTAAAATAATTTGATATTCAGTTATCATAGTTATATATAAGTTATATTTGGCAGGGGAGAATACACTGAAGTTTTTAATGTTTACTATTTTTACCATTTAGTCCCTAATCTATTTTAATTATCCATTTCAACCCTAATTTACTAACTTTTTTATTGTTGCCAATCTAATGTAATCTAATGTGATGTATTAATCAAAAGGTATGATAATTTTCAACAGTACACTCTGAACAATAAAATTAAAACATCAAGAAATAAAACATCAAGGAAGTAATGGATAACTCAAATTTGTCGATGATGTAAGTGATAAAAGCGAAAAACAGTAGGGCCCAAAGGTCATTTTGTTTCATA

>Cs1g25570

ATTGGTTATTGATACTGTAATAGCAGCATTCGTAGATTCAGGAACAACTAGATAGCTGATACATAAAATCCCATCCCAGACATATGTGGAGGGGAATTGCTTCCGGCAGATTGTCTCTGGACTTTTGAGTGTGCCCGTAGTGTTCGGGGCTGCAGTAATGCATGCCACCGGCAACTGATCAGTTTTCTAAGTAATTGAAATTAGAAGTATATACCAATGGCATTCTTTGGCTTATTATTGATATCAATGATTGATATGATATTTCTAGGCGCAGTCCTCAGAAGTCCAACTTGGGATGAATGCCCTATTTATCAGACTAGCATTATAGTTTGTCTTCCTGATATAAACCTTTTTTTGAGTTTATGCCCATTATTTCGTCCCGTACGGCTCTACTAGACAGTAGACATAAGATAGAGGATTGGCCATAGCCGCCAAAATAATCCCGGGGTTCACTCAGTTTTGGAGGCTGCAACCCCTGTAGCTCAATGCTGCCAACATTTTCTTCATTATCGTCTGCTCGTGTGGATTTTGACATCTAAGATTGTTTATTCTTTTCTCTTACAACTTGGATCACCCACTTGATCTAACACACATATAATGTCCGGTTATCTAGTTAACCTTGGTATTTAAAAAGTAACGTCTGTAACATAACTTGTAACCCGTCCTTATGGATTTGGTTTATGTCGAGATATTTTCAACTTCTTCATGGGGTCCCCGGACGACCCAACTTGGTTTTTTCTTCTTCTTTTTAAATGTGTCTCTAGATATATTATATTCAGATAACTTCCTTGATCCTAGTTTCTGTAGTACAAAGTGACTGGTATTTTGAAACCATGGTCCGAGCAAATTATTACTTAAACTTTAGAAGCACCACAATTCATAATCCACGCAATACAAACCTTATAAATAGCTTACAGTTTCTGCACTGACCAAAAACTCAAAGCAAGCAATTCGAATTCTCTGTTGTTCTCTTCCTATCTCCTCCTTAGTTAGTTCATAGT

>Cs1g09120

GAAATGATCAAAAGTTTTCTGATGCACAGGATCCTTAGAAAAGGCAAGTGCAGGTACAGCAGAACATGAAATTTTCAAGGATGGGTATGGCTCTGATACCAGTTATGGTGAAGTACGTGGCGTTGTTATACCAGAACTACAACAGTTAAGAACGTTCTTTGGACCAGTAGTCCGCAAGCTCGGGTTTCCAGCCGATATACCTCTGTAATCAAAAACCTTTAAAACATTTTTCTCAGGTTATACAACCCAAAACAGACTTTCCTTTAGTCTAAAACAGGGTACCAAACCAGATAAGAAATGATCAAAAGTTTTCTGATGCACAGGATCCTTAGAAAAGGCAAGTGCAGAGCATACTTCCTTAGGTTTTCAAAATGAACAAATTTAATTCTTTATTCTTTTTCTTTCTTAGGACTTCATTTGCAGAGAGAGAGAAGAGTTTTAGGGATGGAAGTTTGTAAGAAATTTTATAATTAAAAATTATAATTCTTTAAATAAGGATAAAATTGTAATTAGAAACTATTTACTCCCAATCTAAGATAAAGTAAACACATAAATTGGATTCTGATCTCCATTCCATGCTTCCATTCCAGTGTAAGTAAACAACCTATTCCCACTCCACACTCCCAATCTTAGGATTCCCACTCTCAAAGATTCCCAATCCAATTCAAAAAGTAAACGCTACCTTATTTTCATGTTGAAGCAATCGGTGATGGAAAAAAGTTGCAAGTTGAACACTGACAGTGCCCTTAGTGCCCTACCTCTGTTTTTCCATTTGGAAACAAGCCTTAAAGTGGTGCTCATCCGGGTTCAAAATCCGGGTTTCAGAAACCCGAATTTTTCGGATTGAACTTGGACAAACCTGGACACGGACACGGACCCGGATTTTGCGGATAAGGAATTTTGAGGGTTGTACTACAAAAAAAGTTGCACGACATGGGGCTTATGGAAATTGGGCTTCAATTCTGACCCAACAAAGGCCTAATTTTTTTGAATGGGCAACC

>Cs1g26600

TTTTTCGAGTCTATCGGTGTCTAATTTCTTTATCATTTCAATAGACGAATAGAGGGTTTAGGTCTTTTAATAGACTTTGAGATTTTATTTCGTTGCTTTTGTGTTGGAGTTTTAATTATGAACTTCTATAATATACTTTAATTGTAAATTTCAATTGGAATCGTACATACTTTTTCTTTTTCCTTTTTTTTAAACCCATCATTTTTCTTCTTCTTCTATAAATACACGATTGATTCTAAGAATTTCGCAAATTGAAATTCACATATTTTCTTTCATTCAATAAATTGTATTTAATTTTCTTTTGAAGAGAAATTCATTAAAACAGAAAAACGATTACAAATAATATTTAATTTTTAATCATTTGATTATATATAATATTGTTACATGGTACTCATAAAATTATGATTACACATTTATATTTTGATGGAATGAATTAAAAACGTGCATTGAAATAAATAAATAAAAAAAGATTCTTTTATGTGATTCGCTTCCAAATAAAATGCCATAAAAACAAATAAAATGCCATAAAAAAATAAACTACCATATTAAGAGCAAAATGATAGATTAAATAAAATATAGATGAAATAGCCATTTAAAACTGCATCTCAAATTTCATCATAGATAATATTATTAATTTTAATTTACTTAAAACATGTGCAAAATTATAAATCATTTTATAAATAACACATCACATATCTTATCATTTAAAATAATATTACGTAATAAAATTTGAAATGCCTTATTATATTAGAGATCCGGAGGTGCTCTAGAAATAGTTTGTATGCCTATCTCATATATATTTTATTTAATTTATTATAAACCACTTTATAAAAAGTTAAAACTACATAAACATGCCATAGGAGCTGGCTACGGTACAGATTCTGTAACATACAGACCAGGTTAGCCAGCCCCCGTAAAAAAAAAAAAAAAAAAAAGAATGACGGCGTGTTCGTAAAACGATGCGTCTCGTCGCTGAAATTCAAACGGTGTCGTTCCGGACA

>Cs1g22510

TTTTGCCAAGCGAATTTTTAAAGGATTATTCTTCTATTTTTTTTAATTAAAAAGAGTTAAAGATTAATTCAAGCTTAAAAATGAAAAAAAGTAGTTTTACTATACAAAATTATATTTAATATAAATTAAAGTTGGTTGGTGTCAGTAATTCGACACTCTCACTTCTTAAAAGAGATCAAGGGTTCAAGTCTCACTCTCGTATGAAGTCACCACTTTGACCAGCACTTTACCTCTTATGGGCAGACCCGGTGCGAGCGAAAATTAATCTGAATTGTGGTACAGACTTAAAAGCGTCTCACATAGTTAGGGTCCACTCAGGACTACCTCGTGGTTAAAAAAAAATCATATTTAACATACACTTTACCTCTTATGGGTCGATCCGGTGCGAGCAAAAATTAGTCTGAATTGTGGTACGGACTTAAAAGTGTCTCACATAGTTAGGGTCCACTCAGGACCACATCGTGGTTAAAAAAAATCATATTTAACATGACAGAAAAATTTGTACGTTTTTTTCAAGACACGACAATGTCAAATAGTGGGTTTTCTATTTTTTTTTCTCTCTCTCTTTCTCTCTTCTCTAATCATACATGTCAAAATTGAGTATTAAAATATTTGTTAACCCATCACATTATAAGTTGTTATTTTCCTTTCATTCACTCGAAATTCGAAAATAACTTTTTAAACCAAATCACGAACGACCCACCATCTCAATGCCATATGTATAACTTATATTTTGTTATTTAGGGAAAAGCGCCGTCTCAAGTCTCAACAAATGTGTTCGCTTGCTTCCTTAATCCTTATTAATTATTATTCTCTCTTCCTTTTTTTAATAATATAATATTAGGCTTTTTAGTTGTCGGACACAAATCATTAAAAAAAAAAAGACAATTTACGAGAGTTCTAAAACCAGAAAGAAAATAAAAGAAAAATAAAAGAAAAGGAGAAAAAAATAATAAGGTAATTTTGTAAGTGAACAAAACCAAAAAACGACAAAAGAAAAA

>Cs1g21770

TCTTTGAAATATTTTTGTACAACGCACAAATTTATTTACCATATGAATGATTTAAAATGAAAAAAAAAAGTTATATCTTGTCAATTGTTCATAATCCATGTTTAGATATCTTTAGTTCTGTATTAAAAATAGTAATAATATTATTAACATACTAGATTCCTTAGAATGAATCATTTATTAATTGAGTGTCTTCATTTTCCTTTTTTATCCGGACACACAATTAAATTATAATTAATTTTTAATTTATTATTAAATTATACAATTTAATAAAATTATTTTAATTAATTATATTAAATTGTATGATTAAATAGTATGTTCACACAAAAAAATATATAAATACGTATATGAAAATGATTATATAAAGAGAGGGGGGTGCTTTAATAAGTAATGGGGATATTTTAACGTAATATTACAGCGGAAATAGAAAATGTATAAATGAAAAAAGAAGGTAGACAAAAGAAAAGTGGCCAAGTTCGAACTGAAAAGCCATCACTAATCTAAGACCATGTGATGATACCCAGAATCCAGCCGAAGGTCCACTTAATATTGTAATTGAATGAGCCGATAATATCGGCAATCAATCATATTATCGCGAGAGAAGAAAAACAGGAGGCAGCAACTAAAGTTGATTTTTGTGGGTGGTTTGATATTTTCATTTTCTAAGAGAGGTTAGTGGTTCAAGTCTCACTCTCGTATGGAGTCATTATTTTAAGCAGTACTTTACTCCTTACGAATCGATCCGATACAAACATTGATTAATTTAAATTATGATATAAATTTAAAAATATCTTTAACAATTATAGCCTATTTAAGACCACCTCGTGATTAAAAAAAAAAAACAGGAGGCAGCCTTAGTAGCAAGTATCTTTTACTCTTTTAATTTTCAAAATGTTTAATTTAATAAAGAAGAAAAATGAGATGGATGGCATTATTATCAAAGTACGCGCGCGGCGAGAGGATGGACGTGGATGGGTCGTTTTCTGGTACACGGGGGCTGTTTC

>Cs1g01030

GGGCTACATCTTGAGTTTGTTGCCGAGAACGAGCTGCATTTTGAGTTTGCTGCAGAACCACAAAAGGTACTGACGGAAAGGAGAAGTGAGAGCAAGGTTGTAACGCCCCTTGTGGAAATTGAGGATAATAAGATATCTGGGGGGTTGGCAGACACGCAGGTGATTGATTTAAGTGATGGAGAAGAAAATGATGATCTGAGAGGTGATAATCAGACGCGTGAGACTGACTTACGATGCCTGGCTTGGCATTATACAGATCCCCAAGGAGATATACAGGGCCCCTTCTCTATTGCATCGCTAAAACGTTGGTGGGATGATGATTATTTTCCTTCAGATTTCAAGGTTTGGAAGAGTGATCAGGGCCAAGAGAATGCTGTATTATTATCTGATGTTCTTCAGGGGTCGTTTCCCAGTTAATTCTGGAAGATAAAGATTTTAGTAGTCAAAAAGAGTTGCAAGTTGTTCTGTAACATACAAATTGAAATCTGTGAATAAAATTTTGGAGACTTTTCATTACAGGGAATCAAAATTTGACAACTGAAATAGTTACTCTTCTAATTTTGTGTTTGGAGCTGGAGATGACGAATCCATTGAGTTTGGAATAATATTGACTTGGGAAAAATAAGATGGTAAAATTAAGAAAGAAAGAAAAATTAAGCTTTTAGTGATGAGATTTGCTCTCTTTTGGGAAGTGTAGCATTAAGTATACCATTCAGAATGAGCAAGCAGTCCTTTTCTCCTAAGGGTGGGGTCTTGTTTGTTTTGTTTTGTGTTTTTTTTTTTTCAAATATCATTCGTTAATTATCCAACAGCAATAATTACCCGAGAGAAATAAAGTGAGTTTTTAAAGAGTAAACAAAAAAAACTGGTACGGCTATATTTTTAAAATAAGAAAATATATATAAAAATAAGAGAATAATTAATGGAAATTAAAAAAAAATGAAAAAGGAAACTAATAGGCGCAAGAGAGAGGAAGGTAATTTGATTTGCTGGGACCTGGG

>Cs1g26080

ATTGTCAAAGTAATGGTTACTTTTATATGGGTCAAGTTGGATTGGGTTGGGTTAGTCCATCATGAGTGACTCGTTAGACAACTTGTCCAAGTCTAATTGTTCAAAATAATCATATTATTATTTTGAATACAACATACTACACGTGTTAATAAATATAAAAAAGGTGGTTAAATCTAAAATGTCCTATATGTTTTTTATTTTAATTTTTGATAGAAAATGTTCTATATGTTTGTGGAAATAATATTGAATAAACAAGAGATCTTATCTTATTCCTAAATGATGCGGGATTCAAACTAAAGTTGTAATAAATTAACTCAGATTTAATTTAAGAAGCTTTGATGAAGTAAGATACATGCTTTCCTATTTTATTATTATTATTTTTTTAAACCCCTTAAAAGTGTTAAAATGAGTTTCTATAGAGGATTGAAAAATCCATTCCCTGGTCCGTTTCAAAGCAACACCTTCTCCAATTTCCGTCTTCTTTTGGGCTTTTGGCCGCTATCAAGGCAATTGCAAGAGGGTATTTTAGTCAGTCCACTTAAACAAGGTCGGTCTCGTGACCTAGGCGGCTTGTTTCGTCTATAAATATGCCTTTGGGGATAGCACTTTCACTCTTTCGATCGCAGAGGATTCAGAGAGACCTCTATCGAATCAATATCCCTCGATCTCTCTGTGTCAAATAACAACGGTAACCAACCCAAAAAGTTTTTACGAAATAGAAACTGAGAAAAGAGGAAAAAAGCAACAACATAAGTTATTATTAAGTAAAAAATTTCTTTTTAAATAATTAAACTATACTTCCCAATTTTTCAGAAACCCTAGAATAGGGTTTTCTACATTCGATCGCCATGGATACGCGATTTCCATACTCCCCGGCGGAGGTGGCCAAGGTCCGCATGGTCCAGTTCGGCATTCTCAGTCCCGATGAAATTGTAATTTCCTCTCGAATATCTCTTTTGTAATTCCTAGTAGTTTGTTTGGTTGCTGAGGATAAACGGGAA

>Cs1g02720

TCGACAAGATACCAAATGGAATACAAACGGCAGGGGAGGTCTTTAAAATTACGGTCATACTTTCTATCTAAGTGTCAATACGCCAAATCTCAGGGGAGATCCCTAAAAATTACCATTTCTTGAAAACATTTTTGTAACGTTACATTCCAATTCCAAAGATGTTGGAGACAATACGAGTTGGAATGATATAAGAAGAATGCTAAGGGTAGAGAAAGATGAGAAAAATAAAAAAAGAAAAGAGTAGAGAGAGAGGGTAAATTTATCTCATCTTTATCTATATTAAGTACAACCCATAATATAATTAGTATCCAGACACACTTCTCATACTTTGTTACCCACTCCTACATCACGCAACTATTGGATTTGAATTATTGACCTAGCTAACTACAAATGTCGTAGACTTGGACAAAACAAAACTGTTTCTATGGGCATTATTTGAGGTGTAAAATTTATATTCTACATCTAATATTAATAAATGGATCACACGTCAATTAAGACTCGATTGCTAAAATTCCTGGCATTTACTCTTTTAACTATTTGTAATTCGTTTTCTATTTACTGGTCATGGTGTATAATTATTATTATCATTGGGAGGGGCAAAATTTGCAGCTTCGATGGAGTTGGTTGGAGATATCTGCATTCTACAATGATCCTTTAGGAAGGTAGCTTATTTATCATGGAAGAAGATTTTAGATCGTAGCAGAATAATTTTCCAATTACAGGCAGCAGAGTGAGTGATATATAGTAATCCGAAAAAAAGGCAGAGAGAGAAAGAGAAAGAAATCAACACAGGATGTCCACTACAAACATGTGAAGAAATCACTCACAAATTAAATAGCTAAAAGTAAGGCAACGCGTAACAGGAGAAACGCTATCAATAAAAAGTGTTACCATCTATGTAAAGATATTAAGTCCAGGGTTTGTAATTCCAGAATTTATTACTAACTTTATCATGCAGTTCGCTAACCCCCCTACTCTCCTCGATAATTAATGATACTCGT

>Cs1g14000

TTACAAACGATTAACTCTGAAGCGTATGAACAATGATTAGTGAGTCACTGATTAGAAATGGAAAAATCTTTAGTTTGGTCATTGTTATTTCTAGAGCTTATTTTACGGAAATAATTGAAGAATAGTGACGTCATATAATATAGAATTTTGCTTTCAAATGTTATATGTTATTAACCATATAATATCTTTAGTTTTTGTCATACTAAACATATAAAAAAAAAAAAAAAAAACTAGGGCTTTAGATGTTAGGGCAAACAATTACCCAACGATTTCTACTTTCTACTTGCAAAATAGTAAAAATTATAATAAAAATGTGTCCAATAGCTAGCATATCAAATTAATGACGTAATAAAATAAATGAAAGCGGAAAAAAAAGATCTTAAAATAGAATAGTTTGCTTGAGAAACCCAAGTCAAGCCAATTACACAAACACCCCTGCACGTAAAGTCACATTGAACCAAATTTGCGTCCATTTCCTTCAGCTGCTAAAATCAGTTCGTCAAATTATTTCCATTTTTATTATTATTACTTTAGTGCAGACATTTTTTTTTGTATTACACAATTCAATACTAGAGATATTTTACCATCACGAAGTTATTCGATCAATCGATATTATTATTTTTATTATGGTATGGTACAAAATATTACTTCTATATCTTGAAGAAATTTATTCTTAAATAAAATAAAATCCTACACTCATATTAAAGAGAAATTATAATGATTCAAATTCATTTTTTTATTCGTTACGGGCAAGTGAGTCTTTTAACATTAGGTTATAGAACCTAACAGCTTTTAGGGCACACATTGAATTATTGAATTTATTTTGATAAAATTTAATTAATTGGACTATTTATTTATTGTTTTTAAAATTTTCCAACCAAAAAATAAATAAATAAATAAAAAGAAAAAGAAAAAGAAAATAACCCCAGGAAATGCCAAGTAATTACAAAAACAAGGGAGAGAGGGCGCTCACTTTAGCAATTAGCTCTGAAGTCTCCAAA

>Cs1g14030

TTAATAGATGTGTGTGGTCTATAGTAAATTAGTCATTTTTAATGGATGTGATTCATAACATATTGACACATTGTCACGATCCTGTTAACACTTTCATTTTGTGGCCAAACTTCCATTGGGTTTTGCTAAACTTAATCAGGGTGATGATTGTCGACGTTAATTTTCCCGTACAATAACTTTAATTTTTCTTTTTTTTTTTGGACGAATTGTGTGTTTTGGTATTTAAAGTAATTATATTATACTTGTTAATAATAAATTGTCTTTGATAATAAAATTTTATCATTATTCTTTTAATAAATAATTTTATTAAAATAAGAATATTCTGAGTAACTTTAGGGTGTAAATAACCCCGTCATTTTATCATACAGTTGTATGTTATAAAAACCTTATATTACCTTTACTAGTTTTGTACAAAGTTATTTAAAATTCATAATTATCGTAAACAGTCGACTTCTGTCATATTGTCACAGTTGCTTTTAAGTCACAATATCTTGATGCACTAAATAAACCTTTTATCAAGGAGGGTAGAAATTATTATATATTCTCGAATTGGAGCACATGGCTTTACACATGGACATTGCTTAATATAAGTGAGAAGTTCCATAGGAGTTGGGAGATCTTATTTCTAGCAATTAATTTCTTAATAACAACCATGCAGTATATAACCTACTCAATTAATTTCTTAATAAAAATTTCATCAAATTTTTTTTTTTTTTAGCTGAGATGGTTGTTTAAAGAATAATACTGTGCTTAGTATATAGACACTTAGTTGCAATATTTATTGATGTGTTATCAATTATTTAATATCATGTGTATAGTACTAGTTAATTTTATTATTAATTATTAATAGATTATTTTTATAACCTACTTAATGGACAAAATATAAAATAGTACATCAATTTGTATCTCGATTGAATATCTACGTGAACAAGCATAATATTATTTTCTTGATGGTTTTTTTTTTTAAATGATAGTTTTACCGGAAAATGAGACTCTTACAA

>Cs1g01280

TGATAATTTGATGCATTTTGTCGTCGAAGCGCACGTCATGTGTAACGATGAGGTAAACATTGTGTTATTTGGTAAGTGTATTATTACTTAATTTTAAATAGAAAATCTTAAAGAATATTATTATAAGTTTTTTGTTTTCTGAGAAAAAAATAACTATAAATTTAGTTTCAAATAACTCCAAGTTGTTTTTCAGATTATGGAACTATATAAATTAAATCAAATTTCTCTCTATCCTATGAATTTAAATCCTAAAATCTCTCTAACCATTGAGAGATCTTAGGAATTGTAAATCAGCTAATAATCCTTCCAATTAAATACTAAGACCAACTACATAACAACATCACATTAATACTATGATATAGAATTTATGAATTATAAATCTATGATACGAGACAGGCTTAGGACTAATTTAGTAATGTTGTAGTTTTTGAAATAGCTATTGTTAATTTTGTTGTATAAATAATCAGTTGTAAAAGCAAATTAGTTAAATATTTGGTTTTTAAAAGTACTAAGAATTTAAAAATTAATCATATATATTTAATAAATATTATCATTAAATGGCTATAAGTATATAAATGGTTAAAATCAATGTTGAATAAAATTTATTTACGTATTTTTAAATGTGTATAATATTTTTTATTGTGTACATATAATAATTGACGACTATAATAATTTTTTTTTTGTAAATAAAAATAATTTCTTTAAATTTAATAATTTTATTTCTTAATTAAATAATGATAATTTAAATTTTTATAATATACTAAATATAAAACTGCTGTACCGTACATNNNNNNNNNNNNNNNNNNNNNNNNNNNNNNNATTCATAAATTTAAATTTTTATAAAATATTAAACATTAAAATTAATTTTAAATTTTTTAAAAATTACTTCATAACGAATGATAATACTATAATGATTTAGTTGACGATATTGAGTACCATATAACAACGTCGAATTAAATATTAATCTATAAAAATTATCATTCATGTAAATCGACTCGATG

>Cs1g03410

AATACTCTTATTCCTACCCATTAATTCTCCCAACTTACCCTTCTCAAAACGAAAATCATAAAACACGCGAATCTTCCTACAAATATCATACCAAACCTCCACATTTCCCTCAACAGAACTCATCAGACCAAAATCAACAAAAACCGTTTTCAAATCATCAAACAATGTGGCAATCTCTGTACCCCATTCATCTCCATTACCAGCCAACACGTGAGGAAAACCCAAACAAATGCCGACCAAAATAACATTACTAAACCCATAATAGTTTTTCAACCTAGATAGCTTAGCTGTTAAATCTTGTGAACTCTGACTAAAAATCGAAACTTCCTCTTTGTATAACTTACCCAACTTGTCCCAAGGAAACCCAAAACTGGAAAGCGCACAAGCAGCATTCAACAAGCAGGAATCCTCCGAAAGGAAAAACTTATTAGCAGGCAAAAAGCAACTAACTTCAGCATGATCAATACCAATGCTTTCAAAGAAAAACTCAAATTCGTTAATGGGATGGTACCTGAGGAATCTCTGGAAGGTACTAGAGAAACTTGGGGCTGAATAAGAGACGCCAAGATCGGCAATGAGCTTGGAGAGAGTGTTTAGAGAGTGCTTGCTAATGTGCTCCGCATAAGTGAAAGGTATTGACTTTGTGTAGTGGAGATACTCAGTCACTGCTTGTTGCGCTTCTTTGATCGCTCGTGGCCTGTACTTTGTAGGGATTTTGAGTTTTGCCGCTGAACTCAAGTGGCGGGAAAAGATTGTGCATAGAGATTTAGGGTTTTGCATTTTTGCTTCCTGGGTTTTAGTTAGGGTTTTCGGCTTTGTGATTTTTATTGCATTTGGTTTTGGGGCTTTCGGTAAAACTCCTTAATTTTCTAACTTAAAAACAAAATAATAGGAATGGCTGGCGGTAATGGATAATAACACGCTAATCCTTTATTTCAGTGATATTATAAAATATGGCTAAAACTTTTACTTTGTTTTTTTGGCCTTTTTAATTGGGAGGA

>Cs1g20820

CGGGGAGGGGATGAGGATTCATTTAAGCAATTCTGACGGGGACGGGGAGGGGACGGGGACATAACAAAATATCGGGGATGGGTACGGGGAGATTGGTCCCCTCCCCTCCCCTCCCCATTGCCATCCCTACCCCCACCTTTGACTTTTGATTTGCGCACTCGCTTAGTTTTTTCTACCCAAGCAAATTTCTGTATTCCAACATAAATGTAAATAAATGAGTATTTTGACCTTTTCACTATTTGATTATTCAAACACAAAAGTATTTGATGGTTTTTTTTTTATTATCCACTTGAACTAAACTGAAATAATGTTAGGAATATAAAAGCATTTAAAATTAAAAAGAATAAGAAATATAGTTTTAAATTAAGTATTAAAATGTACTTATTTTTTAAAAGCTTTTATTTATTTATTTATTTTTTGCCAACTGATTTGTCGTTATGGAAAATAAAAATTACAAAGAATAGTATGAGAATTGTATTGCGCAACATAAACATTAGTAAGGCATAATTTTGCATTTTTGAAACTGAAAAGGCATCTATGCATAATTACGATGCCGTCCTTGAGTCTTGAGAGATAAATAATGTTGTAAATAATGACAAATGATGGGCCGCTATCAATTCCAAAGAAAAGGCAAAATTTAGGGTAGTAAGTAAGTGAAGGATAACCACTTGGGCTAAGAGCCACTCCCTCACAATTGTGAGGGAAGTGGTTCGGGATTCCGCAGATGAATTACGAGAGTTTAATTTAAATATTTTTTCTTAGAGTCTTAGAGCTTGAGTGAAGGCAGTCTTTCTCTTAGAATGTGAGTTGGGCGTCTCTCGAATATTTGAGAGGGTTAAAAATCTGAACGATGTCATATCAAAATTGATATAAATGAGTCTCAATAATTTATATGGTGTAAATATCATGAGTAATAGTTCTCTAAAAAAAAAAGTAAGTGAATGATGATGATGAAAGGGAATAAATAACTTTTTAGAAAATGTTAAATAAATGCAATTGAA

>Cs1g02295

CATTTAGTTAGATTTCTTAAAACCCCAATTTCGATTTTAAAAAAAAAAGAAGAAAATTTGTTGAATTTTTTTCTTTCTCTCTCTCGCTCTGAGGGAGAGAAATTTTCAACGTAAGTGACGGGTTTTTCTGGTGGCAAAAGAGAGCATCAGGATTTGTTTTTAAAAAAAAATTAATCAACAAAACAAACAAAAAGTTCCTACGAGATGGTGGAATTATTGAATTTCCATAACTGCCCATCTGTTTTTCACATTTTGGCAATTAAATGAACTCTCACACTTACTGAGATTCAAACACTCTTTTTAATGGGTTAAAAAATAATAGTCATCATTTTATAATTTATTACATTATATATACTTTTTTTCGAATTATACTTTTTTCATCGACAATCACTATCTACATCCTCAAAATCAAAACATTTATTTCTTTAATTTTAAATTGAGTTAGATTTTAAATTTTATATGTCAAAAAAAATTTACGTATGATACTCTCATTATAGATAAAAATTAAAAACTTTAATTATATTTAAATTATCATTTACGATACTACCCATTTATATTTAGATTTCTATTTACTAATTATGAATTTACACACAATAATTTCATCTAATAGCTAAGGAGCTATTAATTTTAATTCTAGTCTTATTGCTCTTGTAAAGAGATACAAATTTAAGAAGAGAAATCAAAATTTTTCATAACATAGAAATAAAATAGTAAATGACTCCAATCTTGAAATTGACTTCTGCTTCCGCGCGTGGGAAAAATACAAGGTAGTTGAAGATTTGACGTTAACAATTAACAAGGCTTGGGAAAAAAATTAATAAATGAAAAAATAAATCAAATTAAATTGTCTGAGCCCGGGTTCGAACCGGGGACCTCTAGTGTGTGAGACTAGCGTGATGACCAACTACACCACCCAGACGATGCCTAATGTTTGGCAAGTTTTTAGTTTTCACTTTATTTTGTATTTTAAAGTCCCTAACCCGTTAAAAGCAACACAACCC

>Cs1g25190

CTTGGTTGTTCAAGTTGTGAGCAATTGAGCCGGTTGATTGATTGTCTTGTTCATCCACATTTCATTGGTTAAATTCATTTCTGCCTTGTAGTTGTATCAAATAATATGTTGTTGCTTCTGTTAGAATGATGTACATTGTACTTTAATCTGATGTCTTCCTTTAAACTGAGGTATCTTCCGGATGGCGATGATCGATTGAATATCAGGAAATTGGAGACAAAGCAATATTGGCAATGAAAGAATTTTACTTTCACCATGGCATTCAAGGATGAATATTACAGCTGTTTTAATGAATTTTCAATTTTTCATTATTCATAATGTCATTGTAGTTATAATGTATCTCATGAGGGTTTAGCAAGTTCACGAACTATGCTTGCTTCAAAAAACACTCATAAACTCAAACCGCTCTGTTGGTTTACTGGTTGGCTTGTCCTCAACACAAAGGAAAGTCTCTCCATGTTTTTGTGAGAGAGTAGAATATATTCTAGTTCACGACTTCACGGTGGGCCACTCCATTGTCCATTCTTTATAACATAATATTTTTTTTAAAAATATATTAGATATTATAAAAATAAAAATAAAAATAGAACACTCATAAAATTTGTAATATCAATGGACTTGAAAAAAAAGTGAACACAGATATTTTATATTTAATGTAATGCAGTGTATATTAAATTATTTTTTTTGTTTTTAAATTTGTTCTTAACTTTAAAATGGTCAGAAAAATAATTTATATATATATATGAATACTTATCTAGGTGATAAATTTTATAGGGTATTATAAATCAGTTTACAATATTCTAAAATTTAATTTATGATTTTGTAAATACATCTTAAAATTTAATTCCAAATTAAAAAATTTTCCCTATACATGACTAATTAAAACTAGTAAATCAGGTTTTCAAATAATAAGATTGTATTACAGGAAAAATTATGAGTAAAATAAATATATTAATTTGTCTATAAAAGAGAAATGAAAAATCAAAAGATTCGAACGCCAA

>Cs1g14680

TATGAAGACTTAATTTTTAGTTCGTGATCACTGTGATGGGAAAGAATTAATCTTCGTCTGTACATATCTTTGCTTTTCCATTAAGAAAAAGTGAATTTAATTCATGAACTATTCACGTCAAACAATAAGCCAATTATAATGGACATTCAATTTAATTCATTTCTTCTAATCCTCCGTCCTTTACAAGATTGACTTGGGTATATTAAACGACTGAGCTTTGGGATTTGACAGAGAAAAATAAATGAATGAACCTTGACCGTACATTATTTGACTTGTCAGTTACAGACGTGGATTAGGTACTCACATGAGTGTGTAATTTGTAAATGGCAAGTCAATTATCTAATATTTTCCCGTGTATATTAACACATTTCTGGTTATTTTTATGGGTGATGGATTGGATATGCATGACAGCTCAATAATTACGGTTATGGTAAGAAAATAAAAAACGTGTTTAATATTCATCTATAATTAAATCATGAGGAGGAAAAAGAAAGAAATTAAAATATTAAAAAAAATGCACTCGATGTGTTCATCTATTTCATTAACTCTTCAAAAAATAAAAATATATATACTTTCTTCCAATTGTTATAAAACACTTTATTTTTATTTTTATTTTTACTTAATACCATATAATACCCATCAAGTAATTTAAATATCAAGGACAAGAATATGAAATTAATTAAAAATAATAATTCTTTTTAATTATCATGAACCTCAGTTTTACTCAATCCCTAATTAATTTATTTAAACTTAAAATTCATGATCATTCCACCCTCAATGAAATATGTCTTAATGAAATAACGAAATTCATGAACATGCCAAATCAGCTAGACCGCTCGAGAAGACTAACAAGTAAACTGTTATGTACTATTCGGAGTATGTTATTAATTATGAAAGATTCGGATATTGATTTCTTGAAGAATTTGTCGTACGACAGCTTCCTGATTTCTTTTAGAAATAAATGATATTTTCATAGCAATTAATTAATATCGATCTTATCC

>Cs1g09980

GCCTCTCAGAAATGAGTTTTCTGCATTTGTGCATTTCAAAAGATATTTGCCGTCAAGATTAGCCATTTTTCTTTGTAGTCAATGAAGGATTTAAGCATAAAGCATTTGTGTTCATATCTTTTTCAATCCTAATTCTTTGGCGTTAAGGAAAAAAGCACACTTCTGTATTGCTTATTTATTTATTTTTTCGATCCCCTGTACATTTTGTAAAGAGATTTTACTAGTTGAGTCTTTAATATATTATAAACTCTCTAGTTTTTTAGTTTTTTTTTTTTTGACAAGTGTGAAGTTTTTTATCAATATGTTTTTTTGTTAACACTCTAGCATCAAAGACGAAAAGTTATTTTAAAATTTTAAGAGGGACCTAAAAAGCAAATAAAAGACCTAACTGTCAATATCTTCAAACTTATGATTTACACGTGAAAATGTTACTTAGAAATTTTGTAATTCCTCCATTAGTTGTTAGGTATTTACTCTTATAAAGATAAAATTTTGGAACACATAGCAACATATATATATATGTCTCATCTCTCTCTCCTCGTGGGTGTTAGCTAAGAGCTAGTTTGTTCACAAGCTCCATTTTTCCCGTAAACAACTTTTGAATACGTCCACCCTTCAATCCATGTATCATCGTTCAACATCATCAAAAGCAACAACGTACAGGGCGCGGTCCATCACATCATCATCATCATCATCATCATCATTCATCATCATGCATACTTTTTGATCTTTCCCTTTTGCCATTGTTACAAACTTGATTAAATTTCTGTCTTTGATCTTCAAACCAATACCCATTATCATCCCCGCCATGATTAGAATGGACCAAATGATCATAGCCCTACAGCAGAGTCGGTGTAATCTTAACATTATTGTTATTATTATTTGATGATATTCTCTCTCTCTGGGTTTTTTTTTTTTGTATTATTTGATGATATTCTCTCTCTCTGGGTTTTTTTTTTTTGTTTTTGGCCCCTTCAATAATATAAAATTCCAATATCCAA

>Cs1g25350

ATAAAATTTATAAAGTAAGATCATGATCCAATGTACAAAATTGAAAAAGAAACGAGGGAGTTCAATATATGCAGGATCATTAAAAAATGTCCATTTAATAATTTGATTGTTGGGATTAAGTGTGTGTGTATGTAGATGTATGTGTGTGTGTGTGTATACAAAATTTTATTCCAAATTGAAGTGCTAAAGCGGTATCCTGGTTTGGCCCCTTTTTTTAAGTTTATTAATAACAATTTGTGTAAAACAGTTTGTGTAAAGACTTAAGTCTATTGATTGATTCTTCAACCACAAATACAATTGGAATCTTAGCTTGGAAATAATAACCAAAAGAAATTAATTAAAAATGACGGAAACATGTTAAAAAAATATTTCTATTCTTGCATACCATTCAAATTATATTCATGTGCTCCAACTACTTCCATGAACCAAAATGAAAAAGACCAGCAAAATTAAGAATTTACATTTTAATAATTTTATAAAATTAAATTAACTTGAATATATGATTGATATTTTTACTGTTGAAATAATGAAAAATCTTAGATTAATAAATCTTTTAATTTTTAAAATTGATTTCGAGTCGGGCTCATAGGCCTTATTAAATTCTCGAATCCGAAGCTGAAATTTTTTGTACCACGGAATTGACAAACTATTCATCATGTATCTATGTCTGCTATTCTTGTAATAAAGATCGAATTTCCATCTCCAACCAGTGGAATTGATAAGTTAAAATTTTTTTATGATTGTCAAAACTCAAAAGTCAGAACTAAAAGTCATAATGATAGACAATTGAAATTTTGTTTTACAAAATTTTAATTTTATTATTAATTGTCGCAGGAAAAGGTATTGATTTGGTTTTGAAATTGCTGTTTTACAAATTTTTCATGACTGTCAAATGTCATAATTAGAAGTGATAATGATAGACAAGTGAAATTGCTATTTTACAAATTTTAATTTATTATTAATTGTCGCAGGAAAAGATATTGATTTGATTTTAAAAAAAA

>Cs1g19510

TAGAGGAAAAGTTTCATTGTAATTTTAGGATTTCTCTTTAGCTGAATTTTTCTTTCTTTCTTGTTTGATTGATTAGTTCTTCTTGCTCTCACATAAATAGTAACAGAAAAACCAATGTGGTTTGAAGAGGAGGGTTCATTGAGGGTTAGTGTTGGTATTAAGAGGGGTGTCAACCGAATTTTTTTTTGTAAGAGTTAATTTTCAAATATTTAAAATAATAAAACAGAAAAGGGGTGTTAAGAGATAGTGAAGGGTGGATGTAGCACTACTCTTTTTTTCGAGGGTCCAGGGCCTCCACTGATAAGACTTGGGCCAAGTCCAAGTACATATTCTTGTCTTAATTTTAATTTTTTTCTTATTTTTTTAATAATAATAATTAATCTTCAAGTTCGAAAAATTATACTGATATACTTTCAGTTCTTTTCTTTTCTTTTTGGTTTATTGAAAGCTTCATTCATTTATTATTATATAATTCTTTGTTATGCGTGTTGTTATTGTAGAGTCGTAGATTTCATTTTCTGCATTGCATTTTTTTTTTGTTTTGCTATTGAAAATGTTTGCTAGAAAACTGTATGATACCTTCTTCAAAAAAAAAAATTGAATTTATTTTAAAATTATTTTTATCTCTTTAGTGGCCATTAAAGTGTATTTTTTTTACTCTACCAGCAACTCAATTCCCCAACGCACGTGTGAGCGAGATAAAACCACATACATCTTGTTTTTGTATAAAAATTTTTTATTTCAATTATATAAGAAATTTTATTATTTCAGTTAGATGACGCTTGTTTTTATATACCAACTAGTTTTTACTGAGTCAAAAAGGAGAAGAAAAGTATGGCAAAGTTAAAAAAAGGCAGAAACTGAAGAACTGTTGCTTTTTGATCAGAAACGTTCGTCACTTTTCTAAAAGAAAAGCATATTCCCCATGCTTTACGGTTACCTCTACGGCATCAACATCCTATAAGCTCCACCTTCCATTTGATAAATTAAAGAGGGGAAGA

>Cs1g09070

GAGCTGCAGTTGCAGGCTGAACACTACTAGTAGCAGAAGCAGAATTGGAGTCTTGAGTTTTGGTCTGCTTTGGCTTTTCTTTGTCAGCTTCAGCAGCAGCTTTTATGGGTCTAATGGATGAAAAGCGAAGAGGGTTTTTTCTTGAGGATTGAGTGAATGAAGTGAAGCAAGAGAATGAGCTCTGTGAGACTGTTGCAGACAACGCCATGGAAGAGAAGAAGAAGAAGAAGATGATGATGAAGAGAGCTCAGTTGAATGTAGAAGAAAGACTGGCTCTTTAGAGATAAGAGATGATTGTACATTTTCTTTTTTTACTTTTTATTCTCAACGGCCAGGTTTCAAAGACAAAAATCCGTTGGTTCCCGGTAAATCCTTTTATTTTCTATTTTTATGTACTTAATATTTTTTCAACTTTCTTTTAGAAATTTTGGTTCATTTATTTTGTTCAATTTTTTGTTTCAAAAAACTTCATAAAATATATTCAGAATAGATACATAATATTTATCGAAATGTCACATTACTCTAACAAAAAAAAAAAAGAAGCGATACCTTCCAAAAAAATTTAATTATTTTAACTTACTATCTTCTTTATTTTGAAATTTTAGTCAACTTTTATATTTTTTAAAGACGTATTTTGGTCAAATTAATTTTTTTATAAAAATGACATTTTAATTTCTCTTAGAATTTGTTTTTACTCCATACTTATCGAACTTAAAACTAGTGAATTTTATATTAAAATATCATATCTACTCTTCGATTTTCAGTTTATATTTTGATATAAAAAATAAAATAAGGTAACTTTCAAACGGTAATGAGGTAATAAAATTTTAGAAACAAATTCAAATAGTTAAAATAATTAGTGTTATTATCCTAAAATTTATTCACACTTTGTATTAAAAAAATTTGTTTTAATCTCAGGTAATTATTTTAAGTCTATAAGAGTAATATATTAATATTATATAATAATTTAAAAAAATAGTAAAACGAAATACAACATACAA

>Cs1g21400

TAATTGCAGCCATTACCTCAAACAAGTATTGTTTATGCACTGAACTAGTCTATACATATAAAGTTTGGCAACTAAGACAAATGAATGCAAAATGATAACCTCATAGCCTGCACCCATTAGAATGACATCCAAATGGCTAAATCTCGAATAGATGCTCTAGGAAGAGTATCTGAAAATTTATGTTTTGTGAATTGCCTCAGCAGAATGTAAAAAACTCTATCTGCATACATTATAAAATTGTGATAACTGAAAACTTTCTAGTAGAAAAAATAAGACCTTGCAATCTTAACTAAAATTATTGAAGACCCAAACCCGGCCTAGGCTTATTTAGGCCCGACTTGTTGAAGTTTGGACAAGCTTTCAGAAGATTTGGGTTTATTACCAATTACACAGGCTTGGGCCTAGGTTTAGAAAAGCTCCGCCTAACCCCACAATTTACCAACCCTAATCGCGCACACATAATGTGTTGAGAGCTTTTTATTTTTTTCCTTTAAAAAACTAATATTTATTATCATAGTACTACTAAGACAAACTCCATTTTTTTATAATGCTGTCTAAAATTATATTATACGATAAATATTTTTATTTTATCATATATTATTTTAAAATGATTTTATCTTATTTTATTTCTTAAAAGGAGGAAAAGAGTCTCACTAGGGGAAAGTGGGGGACTCAAACCACTTTCCTCGAATTCGTTCGTTTCCATGATGGAAATTTCTTTAACATAACATATTATTGACTAGACTAACCACAAAGAAAATTTTAATTTCTTGTGCATTTGCGGTAACATTTCAAAACAAAAAAAGAAATGAGTGAGTACAAAAAAAAAAATGCATATGAAGTTAAAGGAATTATTATTATCATCATTGTAATTGTTATTATTAAAAAAAAAGAGGTACTATAGATTTTTATAAAAAAAAAAAAAAAAAAAGAAACTCGCGGTCTTTTTTGTGTTTTTAATCTATCTTTTAAGAAAAAGGAAAAGAAGGTGAAAGAGAAGG

>Cs1g04930

TTATTATTTTTAAACTAATTAATCTTACTAGGACCATCATAGTCTTTGTCTTTTGCTACAGAGCATCTAATTCCATAACCATCTTTTCAACTCTAACTATTCCAGTTTTCTTTTTTTTTTCTTTTCCCCTTTGAATGGATGTACATATCTGGCAATTTAACAATCCCCACGACCCATGACGCTGCAAGTCCACTAACTTTTGCTGATCATTTGAATTTATATTAATTATTAAAAAATTAAACTATATTAAAATAAAATGTAGATTAAAAATTGACTTGTACAGTCAATCTGATAATGGACTAGCTCCAAATGGATTTTCATTATTAGGCTTGATTTAGGTGCTTAATTAGCCTACATTTGGCGGTATTTTAGTTGATGACAGAAATGCAATTTTTCGCTGGTCAAATGACCCGGATTGGGTATTTAGAATTAGCTTCATGCAAAATATTCTGCAACTTTTCCATCTTGGATAAAGAAATAGCCATGATTGTTGTGGGTATGAGTTTCTAATTTGTGATTTTTCTTAAAAAGATTAAAAAAATAATTTTAGTTTTCTTAATGAAGAATGAAGGTTGATTTAGTTGAAAAGAACATTAAGGTGAGAGTGAAAGCTGAAATTTAATTATAATGAATTATAGGCACTCTACATTTCTCGATTTATTTTTCATAGTTATCATATATTACTCCCCCATTCAACAACAAATTAAATTTATCTCCCTCGACGTAATCCTTTTTTTTTTTAAGTAAACATTGTATATCATTTAATTTTCGACTTTCTATTAGTACTAATCTAACAATAGAAACGATGGATGAATTGACTTTATAACATACTTCTTTTGACTTTGAATATTTATCGCTGCACAACGTGGGATCAACGGTTCAAATTTTTTTTATAAAAATTTAAAAAGCCGGCCACCATTTGTAACTGTATATCCTCCCCCCTGCTGTACCATATCCAGTATCAATTCTCTTCATCTGCTTTCACCTCCCACAATTAATCA

>Cs1g01080

GGGGTGTTCGCGGATCGGATTTTACGGATTGAACATCAATCCATATCCGATCCATGATTTTACGGATGATAATTTTTCAATCCAATCTAATCTACGGATTGATAAAATTCAATCCAAATCCAATTCATACATCTGCGGATCGGATGCGGATTTGACTCAATTCATATCCAATCCATACATCTGCAAATCGGATGCAGATTTGACTCAATCCATATCAAATTCACCATTTTGCGCATTAGTTTTCGAATTAAAAATTTTTAGTTCTCTCCAACTAATGAAATAAAAAAAATCTAATATAAAAATAATTTTGCTACCAATTAAATTTAAAATTGAATAAAATTTAAATAAATTAATTATTTAAATAAAGCTAAAATAATACATTTAAAGTTTCAAAATATAACACATCAAGACTAATTATTCAAATAAAGCTATAATAATGCGTTCAAAGTTTCAAAATATAACATACCAAGCTTAATTGTTCAAATAAAGCCACAATGAATTGAAATGGCGAAGTTTTGGAAACCAAACACAGTCTGATCCTTCACCAGGGCTGTTGCGCCTGTCGAAAAGTGGTGATTACGGAGCCGTCCACAATGGGAACCAGGAGAAGCCATACGGGTGGCGATCTTGAAGGTTGTGATAGTGAAATTGTTCAGCTAGAACTAGTGAATGTGAAGTGGCTCGATGAACTTGGGAAAGAAGATGGCTTACTCTCGCCGGAATCGCGCGGTCAATGTCCTTCAAATCTGTGTCGAGAGTGTATCCAACCAGTATTGAGTGTTGCTAGTGTGTGTGACGGCGGGAAAGTTTAGGATTTTTTGTTTGTAAATTGTTGGTGACTTGGCGTTATGTTACCTATAATAAATTTTTAATTATATATATATATATATATATTTTTTTTTTTTTGTACATCCGCCTTGGCCGTACAGCAGTAACTGAAATGATTAAAGGTGGGGTTTTGTGATTCGACTGTTATTGTATTTTGTTAATTCTTTGGATTC

>Cs1g10320

GGTGGGATGATCGGAATTATTTTGTAAGGTACTGATGATTTTGTCTCTGAGATTCTTCTTTCACATTGGAATCATGCTTTGTCTCTCACTTTGTAGTTTACTGGTTTTACAATCTTGTTATGATAAGGAAATCTAAATATAATTTAAAAAATGTAAAGATAAATATTTTTTTAACAAATTTTTATTTCTTGATGTAAATTTTGTAAGTTGTAAGCAGTATTTTATTACTCACATTTTTAAGTATTTCAAGCTAGGGGAGTATGGTCTTTTTGAGTTAATAACAGCCTAAGTTAGAATTAATTAATTTTTGAACGGAAATGGGGGCACAGTGCACATGGTCGAAAGATATGGGCATGCGGTGTAAAATATTGAAACAAATGGGGGCTGACACTAAATTGCCCTAAATTTAATTAAAAAAATTATTGATCTGAAAAAACGAACAGAACTGATGGTTCCATTTTTAAAAGCAATCGGTTCAAATGAAAAAAAAAATCAAAAATTGAAAAATTAGAAAATACAAAGGAAATGATAATTTAAAACAAAAACAAAACCAAACGAATTTGGATTCGAAATATTATTTCTAAAAAAATTGATGATTCAGTTCGATTCGGAACCGAAACGAATTGTTGCTCACCCCTACTAATCATAACTATGTATTTTTTCTTAATACAAGGTTGGATTATTGCATAAAATCCAAAATTAAGGAAATGGTGTTGTCATTGCCGTGGTACTACCGTATCGATTGAGACTGAAATACAAAGACATCAAGCATTACTCTTTTTCATTTTTTGAAGTAATTGACAGCAACGCTCTTTCATTTTCACATGTGATTGATGACAAATCTTTACACACACACATACACTTCTAAGTTTCTTTGGCAATGGCAAGGAAGCGTAAATAGTGGCATCTCTTAGTAATGATTTTGAGCTTGTACTTCAAATGGGTTAATGGAGCAGAGCAAGTGCCACGTTACTTCATTTTTGGAGATTCATTATTTGACA

>Cs1g20980

GTCCTCAATTTGAAATAACTTCCAGCCCAGATTTCTACAGAGAAAAAGTGACGGGTCATTGTGAAACAACCCGAGTTGCTGGGTACAACCCATTGGAGCAGAGCTCTAGCATACATTGCTCGAACCCTATATCTCAAAATACTGAATCCTCAAACTGCCCGAACTTATCTTGCATTCCTTCATTTGCTACCAATGGGTCAATTTTTGTTTCCCTTAGCCCTGAAACCCAGGGCCACTCCCGATTTGTATAGCTGTCCTGTATTCTACCTCCTAGTGTCTTTCCTCCTCTGCCTTGCCATTTATTTATTTTTTCCCTTTTTCATTTTTCTCCATCGTAAATATGTGTAATTTCGGCATACTTCCTCTTTTAGGTGGAATCGGCGTGTATTTATTTATTATTATTATGATTATTAGTATGTCCAATGCAGAGGAAGTAGAGGGAGAGCGGGCGGGATTTTAGTATGTGACTAGTTACATAGAGACATTCTGACACGTCAAATTTCCGATGAAGTGTATTTGTGTTGCCGTGCCTGTATATATTCGCCAAGATTACAGATTAGTTTGGACGGTAAAATCAGTTGCAAGAAACTGCCTACTTTGTTGCTGTTTACTGTCTTTTGCAGTTTTCCAAGTATCAAAGGTATTCACATAATAGAACAGTGCGGATTGCAAAAAATTTTTAAATTGAACCATTCCCGAATATTGGTTTGGTATGTATATGTATTTATGTATCTGTTGTTGCCAAACCAGTTGACATGTGTAAAACTCTCAAAATTGATCTTCAATTTAGTCGTAGGTTTGTGGTAAATTCAGTAACCGATGGTAGCTGACTGTGCTTAGAAAGTATTAAATTTTATTTTATTATTTCTTAGTGATCTGAAATAGTTAATCAATTGTAAAAATAAATGAATGGTATAATAATATACAGAATATTTAATAATTGGTCTTTGTTTAGGATACATACAAGGGTCCAATATAAACCCCCAGAACTTTATACTAGG

>Cs1g11710

TCTCCATTAATTCCCAAGATTATGATTATGTAAATCCTACAATTTATGCCATTTTGTATTTATTTAGTTTCTTAGTTTAGCTTTAGGTAAGCCTATTTTATAGGCTCATTCTTTGTGTAAATAATATGAAGAATATGTAATAATAATATTTCATGTTCATTTTCAATTATATTTGGTTTAGATATTTTAAATATCAATAATTATCACTACAATATTAAATGATATTAATACTTTGTAGGAAAAAAAGGAAAAAAAATTATGTTGGATTACACTTCATTTTTTTTTTTTAACTTCAGTTAAATTTGTATAAGAACAATTATGGTTAATGTGAATATATAATAAGCAAAAACTATTTTACTACTCTTCACTTAATTGTGGAACCCTGAAAATTGTTTGCTAAAAATATTAAAATTAAACAGTTGATGTCTCGTTAGTCAATAGTCATTAATCATGAGAACAAAAAGTGCATTTTCTACACTTCTTTGTAATTAGTAAAAAAACAAGTGCAATTGTTTTACAGACTGAAAATTCCACTAGTAAAAACGCCAATCAAATGCATTTTATCATTTTTATTTTTTATAGTCATTGAATGTTTTTTGCCCGCTCATACTCTCAACCATCTCAATTTTTATGCCTTAGTAAAACCAACACTTCTTTTGTTTTTTTTCCCCTTGTGTCTTTCCATTTTTGTTTTCGTCCGGCGAACTGTAATATTCACATTTTGTTTGTAAGCAATAATTTAGGAGTCTCACATCGATCCAACCCCACCTTATTAAAATTATAAAGGAGAAAACTTACGTTTTCATTACATCAATTTTCTTTAGTCCAGAAACCAAATGCAAATGCGTCATGCATGTGCGTGTTTCCACTATAATTTTCATTGAACGTCATTGTGGGGACCTAATCACAATTATATCACCGCTTATCCATATACTTTGATTTCCGTCGGGTTGAGTGGTTGGTATTCCTCAGCCATCGAAATTGCAAGGGGTGTGATAAAC

>Cs1g18270

GCATTCTGTTGAACATTATAATTATGCCTTAATTGGTTGTTAGATTTTTGAATTACTGGGATTGATATCGTCACCCTTGCATGTGTTGCAGATATGGAGGATGATCGATTTGATTTACCGGCCTGAAGAAGAGGTTTTGGCTGAGCTTGACAAGTTCAAATCTCATATTTTTGGTTGTGATAAAAGTTGTTAGAATACCCAGCTAGTGTCAAGTTTCCAAACTCTTGTACAAATGTGTTTGTTGTGTACCTCTATCACAATTATCCATCGGATTTGTTTTCCTCCTTTTGATCTTACGAGGTGCAAAACTTTTAGGAAACTACAGTGTTCTAAAAGAAAATGGCACTCTTGAGGAATTTTGCTAAAGCAATGAATACTAGTATTTTATTATCCAGCTAGCTGACTGAGCTTAACTACGGAAACGGAAGGTAGTTAACAAGAAGTGTTGTGTCAAATTTTGTTTTAGTTGAAAAAAAAAAGACTGAAGTGTTGTGTCAAATTTTGTTTTAGTTGACAAGAAGTGTTGTGTCAAATTTTGTTTTAGTTGAAAAAAAAAAAAGGAAAATCATCTGTTTAAGACTTATTTTTTTAGTGGAAAAAAGGAAAATCATCTGTTTAAGACTATGTTTGGCAAACGTTATTTATTGTATAACATGTTTGTAACTCAATTAATATTATATTCGCATCATTTGTTGTATGTAAAATATATTTCTTCATTACAATTTTAACATTACATCGCAAATTTAATTAAAATGTTTTCAAGGTTGTTATGGGGTTCGTTGGTAGTGTTAGCGTCATACGCCCAAAATATTATTACCCGGTGAGACTTTTAGCTTTGCGTGAAAAGACGGTTAAATCATATTCTAATATAATGTCTATATTCAAATTAAGGAAGAAAAATAATAATAATAATAATAATAACGAGGAGGAAGGAATTTGGAAATAGCAAAAATAGTGAGTCGTGGCAATAGCAAAACGAAAACCAATGAATGACCATGAGA

>Cs1g02250

AACTAAATTTCAGAATATTTCGGTATGGTTTGAAAATTTGATTTGATTTTGGTTCTATTCTTAAGAACCGAACCTTATGGTGCCCACCCCTAGATCCTTGTATATGAATGATAAGATTTGCTTCAAGATTTCTATCCATGATTCCTTCAATTAATAATAGTAATAATAATTTACACTAGAATAATTTTATATCAATTAAATAAAGACTATATTTTTTCATCTCATCGTCAGGAGAAGAAAATGCCAACCAACTCAATGGAGGGGTTATCCTCCATCCCACATAAATATTAAATTTTATTTGAATTAATTATTTTTCTCAATAGTAATCTATCATCATTTGTTTCAATGGTTAACAATAGGGCAAAGTTTGATGGTCATTCAGTCAAAGAATGAGCAAAAGTTATCCTTCATAATTCACATTTCTAACCTAACAGAAGAAAAAAAAAATTTGCAAGACTTTGGTTCCTGAGTCATGAATTTGGATTGGAAAATGAAAAATAAAATGACATGGTCATCGAAATTTTATGTTAGCCATGGATTAAAGTAGTAAACCGTCATTTTCTAAGACTTTAGAAGCATCCCTTGATTTCACAAAGCAACAAACTAATTAGAAAAGTAAAAAGTGTTTGAATTTTGTTATATCTTAGACGAGTCTCTCCCTAGGTATGGGAATTATTTGTAAATGTAAGAATTCAGAGACTCAAATTAATGAAATTGGGAAAAGTAAAATATATTTTGTTTGATTTATAAGCTTGTCCTTAATAGTACAATTACTTTATAAGTATTTTAGTTATATAAAATTTTATTAAAATAAAAGTGTTTAAAGCATTTTAAGGGTATATTGCCAATTTTGTAATTATCTTATAATCATTGACTAGACGAAACTTTTACTGCAGACCTTACGCGATATGCTCTTAAATTATATCTATATATCAGACATTATTTGAAGTAACCTCTTCATTCAGATCAGAGTCTCAGAGCCAGCAAAAAGCAACTAGAAA

>Cs1g14880

AAATTGTGTGTGGCAAGAAGAACAACAAATCCTATCATACTGAACGCCCACTCAATCTTGTAGGAATTGTAAGTGTGGTCAGTCAAATTGTGTATGTGAATTTCATTAGTAATTGCATTCTTGACTTCGACTGCTCTTATTCGGTATTGCAGGCATGGCAACTGTGGAATGAAGGTAAAGATTTAGAGCTAATAGACCTTACTCTGGATGGATCATGCCCTCCTAATGAAGATGCATTTTTGTTGGTCTCTTGTGTGTGCAAGAGCAAGCAGCAGATAGACGTACAATGTCCAATGTTGTTTCCATGCTTACTAATGAATCTCTTGCCCGGCCTATACCTAAAAAATCTGCATTTTTTATTGATGTTACCACCGAAGAACCAGAAGCTACCGAAAACAAGTCAGAAGTTTGTTACGTAAATAAAGAATCAATTACAGAGATTGAAGCAAGATAAAAGTAATACTGGCAGTGAAATAATTTTCTGTATTGATGAAGCAAAACGGCGCAGAGATGCATAGAGGCGATGATTTATGTTGATTTTGTTTTTCATCATACTACTGATGTTATGAATCACTTTATAATAATCCAAATTCATAAATGGATTACTTTGCAAACGGCAGAATGTTCCATATATTCTATATATGTAGATTGAGTGGCAAAAAAATGTTTGAAAGTGACAGTTATAAACGTAGAGGGGCAAGAGTATTTGTTCAAATTTAGGCCATTGCTATTTAAATTTTTGCTATGCGTTACTGTCCTTATCCAATATTGAATTGGAAACAAAGTCATAACCAATGTCATTAACAAATCTAGTCCGCTCCAGTCTGGCATGCACTGGGAGACTTTTGTGACATACCACAATTGAATGCTATTCAAAAGATTTTCAGTACAAAGTAAGCAATTAAGAGGGCCTAGCCTAAAGTCAATAGTCAGTGTCGACGGCGGACTGCTTCTCATTGATTATATATAACCACTGCTACTCACAAAAGCCAACAAAACAA

>Cs1g09660

ATGTTAGAGGGGTAAAATTGATATTTCATTTGAATAATTAAAATTATGTTGTATTATGCTAATTTTTTTTAAAAAAGAATAAAACGACGTCATTTAACGTGATATGAAAATTAAAAAAAAAATTGATGTTGTTTAACTCAATTGAAAAAGAAAAATAAATACAAATTTTCTAAATCTAACAAGTAAGAAATACTATTTTCCTCTAATTTCTGGTACCTACCTTTGCGCTTAATTCTATTGCTTGAAAAAATTTAAATTCAGTTAATTTATCTTTAATTTTTATTTATTTTGAGTTAAATTATAATTTTAAAAGGTAAAAATATCATTTCATTAACAGTCTATTAAATCAACTAACGGAGATCACACAGAAGTGAACGAATACAATAAAAAATAAATTTTAAGTAGTGTGCTGAAAATTTTTCTAAAATAAAAGTTTTCCTGTACGTGACTAGCATTTCCGGTTCTTTTTGGGTTCGTAGTTGATCATATGTTGACCATTAGAATCTCATTATCCAATCCAAGCAAATTACTTCTTGGAAAAGGGCACACCTTTGTGAGGTAGCGGGGAAGGTCGTAACTGTAAAGCAGAGAAGGAATCAGAAAAGAAAAATGGTAGTGCCATCGTGTGGGGTCTTTCTTTCTTTCTTTCTTCTTTTTAAATTGTATTAATGATGCTGATAAACAACTGGCAGTTAAACAAATTGTGCCATTGTACACGTTATTATACGCGACGAGTTATATGTTATGATTTTATTGGTAGGTGTTCTCAATTTATCAATAAAATATATCGTCCAGGGTAACGTATTTCACGGTGCGTACTTAATTACTCTTAAAACGATATAATTTTAACATCTGTTGTTTATTATTATTTTTTCAATTAATCTGTAAAATCATATCGTAGCGAGAGAAAAAAAGTCGGGCCGTGGGGGATAACATGACGTGTCAATGCTTTTGCCTTTATAAATAATAATAACATAATATGATCGCCCTCTCACCCTTTG

>Cs1g25330

TTTACTTACTCGAAATATCTTCTAAAAATACAATAGGAAATTGAATAAGAGCTTAACAGCTTTCACGAATCAAGACTGAAGTTGGCTACATGATTTTTGATGAGGTGATTTGTGATTACTTGATATATGATCAAGAAAAGAGGCGATTCTTTTTATTTATAGAGTTCTTCCTAGCTACTTATCCTTATCTTCTACTGCTTTCTTATCTTTTACTTTCTACGTGTGTGGACAAAAATGCTTCACGTGACTAAAAGAAGTGTTAGTAGAATTATCACCACAATTTTTTTTTAAGTTATGCTTTAAAAAAAGAAGTGAGTGAAGGGTGACAACTGAGAGTGGACCAGTGCTAAGTCATGCTTCTATCATGTTGTCAAGGTTGTGGGCCTCAACATCATTTAAATTGTTGTGGTAATCTCTACCATCATTTTCTTCTTTTTCTTCGCTTGATTGATCTTCCATGAGAGTTTTGTAAGCCTTGACATCTTCTTTATATTGTTCACGGTAGAAATTTATAAGTTGATTGTTTGTGGTCCTGGACAAAAAAAGAACTTCAGGTATATTCTGACGAGAATATATTTTTTTAGTTAAAGTCTTCAAGGATCAGTTTTGGATGATCAATAAATTCTCTAATCTCGAATTTCTTTGCTATTACTCTGCTTCTCTAATGTCTAATTTGTTTAGTTAAAAAATTATTAGTGCAAATCTAAAGAATGCTGGAGCCTTCCAAGTCGTCACCTAATACACCCTCTCCGCCGAAGAACAAATTATCAGGAGTTTGAATCCTCCTAAGAAATCATTTAGACGACCCCCAAAATTCACTAACGAACCACCTCCGGATGTCCAATTGCCATCACCGCGAATCAAACCAAACCACTCTCATACAGACCCTCCATCTCAAACCCAACCCCATGCAAACCAACCAACAACGTCCACTGCCGCTATCCTCTCGAAACTTCAGCAACCTTCACTCACATCTAATGCCGTAAGTGAACAAAAAGAAA

>Cs1g14450

GCGGTTTGCGGGTTTGCAGGTACAATTGCCATCTCTACCTACCAGAATCTTTATCAAAATTTTAAGAGTAAATAAATCATATTTTAAGTTCTTACAAAGATAATTTACCTCATAATTTGTCTAAAAGCTTTTTGAGGGTGTAAAGAATTGTTAGGAGCTTAAATAAAGTGTGATGAAAACAAATCTTCAACATTATTCATAAAGATCCTTCCAGCAAAGAGTATTCAGTTTCCCTCACATTGTATGTAATATGATCATTCTGCTATAAGAAGATTGGTTTTCAGACTCATGTTCACTACTACAATTTTTCCCTTCTCATTTTGGTTTTTTGGTATTAATACATGGTACTGATTACAACTTCAAATGTGTCTAAAAGAACTTTTCTACGTGTGCCACTCAATTAATCCTGATTGAAACTAGATGGCTCTAAAACGTTTGAAGCTACTTCACAACCGGGATCTCACTTTTTTCAATAAAAATTAGTGTTATTAATCAATTATCAAAGGACTACTTCACTTATGATGATAATGATGATTCGTCCACAAAAGTTACGCAACAGGATGATGTATGGATAGTGAAATGCATGACCATGTGATTCAGATTTCGGTACAGCACTCTTAAGATTTTTCAACCCAATGCACTGAATTAAAGGATGCAATTCAACAGAGTTAGTTCACGTTACACATGGTTTCTGCAATGCCTTGAACATTAATGATCCGAATGTTTTCCTTTTGTTTCGTTTATTCAAATATTAATCGGATGCATTATATTTCTGAGCAGGTTCGCATCAAAATGTTAACGAACATGTTGTAGAGTCAACTGAGAGACAAAAGCCACAGAAGAGATTTCATATGGCGTCGCCAGTGCAAGGCACGTGATTTCTCAAACGATCAAGTAACCAAGTTATATATAGCAACTGATGGACGGTTTAGATTGAATCCTGCAATCTCATTTGTGTATATCTTACAAATCTCACCCCACGTCACAAACCTTATCTTCTT

>Cs1g17850

AACTTTTTGCAAAATGTCCTGTTGGTGTGCTCTATCGTGAGATTAGAAGACTATGAAATCTGATGCACAACTGACTTCCAAAGAAATCTTGATTAGACTATAATAGGCTCAAACTCTCAAAATGATGTGGCTATCATGCAGAAAAATGAAGTAAAAAGACATTGCTCAATTCCAGTCCTGTTTTGAGACATATGTTCATCACCAAGCTGCTGCAGTTGATTTGCTGGCTTGTCTTTGTTATTATGTGAATGAAACAGTCGTCATGGAATCATGATGCCCCTCGAATTATTATTTAGTAGTATGAGACTTGTCGCAGGAGATTCCTGGTGCTGTAACATTACTACTGATTTTTATGAATAAACTTGCTCACTTATCCTGAAAAAGAAAAGCCAACAAAATGATAAACATGGTCGGCTTATAAATAGTTATTTAATCTCATAATTTTTTTTTTAGAATTTTTTTTAAGATTAATAAATTATTTTGTCTTTACTAATAATGTTCAAAATTTAGATTTTTATAATTAGAGATTTGAAAATCTTTTTCAAAATGTGAAATATTATTTACTTATTTAAAAATTTAATTTTATTCCTTTCAAATATAACTTTAAAAAATTTACCTATTTTATTAATAAAAATTTTACTTAAATAAATTTTATTTGAAAAATTATACGCATTGATCTTAAATAAGCAAATCTAGGCTGCCTGCATTCTTCCTGTTGAAAAAGAAAAAGAATGATAATTCCATGAATAGCTCCAAAATATTTGTTTATGCAACCATCCCCCCTATACAAGAGCAAAAAATCCCATAGCTCTCGAAAGCTAAGTCAACCAGCTACGTGGGTCCCTCCATCATGAAAAAATTAGAAGAATACAAAGTAAGTGACAAAAATCAGTAAGTGACAAAAATCAGCAAGTAACTGTCATTAACGTCCCTCTCTTTCTCCGGCCTGCAAAATAAAGCCTCATGCTCTAATGAGCTGGCTGGAAGCAGGGGAGTTTTCT

>Cs1g05270

ATGAAGTCTGGTTTTTGGCTGATACAAATTCACCCCAAAGATCGATATAAAACAGCTTTTACTGTTCCCTTTGGACAATACGAATGGACAGTAATGCCTTTTGGTTTGAAAAATGCACCCTCAGAATTTCAAAGAATCATGAACGATATTTATAATCCCTATTCTGAGTTTTGCATTGTTTACATTGATGATGTGTTGATTTTTTCTCAAAGTATTGATCAACATTTCAAACATTTAAAGACTTTTTATCTTGTTACAAAAAAGGCTGGATTAGCCCTTTCTAGTTCAAAAATCTCTTTATTTCAAACAAAAGTCAGATTCCTTGGCCATCATATTTCTAAAGGAACAATCACTCCAATCGAGAGATCCCTTTTGTTTGCTGATAAATTCCCAGACAAAATTCTGGACAAAACCCAATTACAAAGATTTCTTGGTAGTTTAAATTATGTTCTTGATTTTTGCCCTAATATCAATAGGATGTCTAAACCTTTGCATGATAGGTTGAAAAAGAATCCTGTTGCATGGTCAGAAGAACATACCAAAGTTGTTAGATTAATAAAGCAATCTGTGAAAAATATTCCATGCTTATTTCTTGCAAATCATGCTTTACCTAAAATTGTTGAAACTGATGCATCTGATATAGGTTATGGAGGTATATTAAAACAAAAGGAAAATGACAAAGAACAGATAGTACAATATGTTTCTGCACATTGGAATGATTACCAGAATAATTATTCTACTATCAAAAAAGAAATTCTTTCTATTGCTTTATGCATATCTAAATTCCAAAGTGATTTACTAAATCAAAAATTTTTACTTAGAATTGATTGCAAAGCTGCAAAACATGTTTTAGAAAAAAATGTTCAAAACATTGCATCAAAACAGATTTTTGCACGATGGCAAGCTATTTTAAGTGTTTTTGATTTTGATATTGAATTTATTAAATGTGACAAAAATTCTGTTCCTGATTTTCTAACCAGAGAATTTCTTCAAAACAGATA

>Cs1g16000

GAAAGTCATGACAAATTTGCATATGGAACAACAAGAAGGCACACAAATATTTGTGAACAACCAAGCTGCACTTTCAATTGCTAAGAATCCAGTGTTCCATGGCAAAACAAAACACTTCAAGATAAAAGCTTTATTTTCTAAGAGAGGTGCAAAGAGAAGGAGAATTGCAGCTGATCTACTGCAAAACAGAAAATCAAAGTGCTGACATCTCAACAAAAAGCACTTTCAAAAATGAGATATGAATTCTTGAGGCAAAAACTTGGTGTATGCAGCTTCAGCGTCAAGGAGGAGAATGTTGACCGGAATGACGCATGAAGTTAAATTAGTTTTATTTGTTGTTATTTTTTAGGATTATGACCACTTCTTCATGCTCTTTTCTTGCACTTAGTTGGTATGCTCAACTTGTAGTAGATTTTTCTGCAATTTAGTTTTCATTATGCTGTATAAATAGAAGACTTTGTTGCTTAATAAAATATAACTTAGTAGTCTCTAAATCAGCCTTCTCTCATTATCTTTAAATCCAACAAAAAGCCTAGGACCACATTGCTAGTAGATTAGGGTAGAAATGTAGATCTATACATAAAAAATTGAACTCCTTTTTGGTCATATGTTTTCAGTTTCTCGATTTTACCTCTGACCTGCAAGATTCGCAGAAATTGGTGTGGGAATAGAAATTTATAGTCGTAATATATTGAATATCGCATATAAACATGGATGATGGCGCACATTTTAAAAACATTATTCAGTAAAGATATGCACCTAACATATCTGAGCAACTAGTTAGATGTTAGAAGCAATATGGCAACAATATATTAACTGAAGCACATGCAACTTTCTCATGGCTCCAACTCATAATGCCGCCATCTATTTATTCATCTCAACTACCAAGCCCCCCTCATCACCTACATCCAAGAGAAATCATTAATATGGTTTCTATACATTGTCTGAGCATGAAAAAATATCTGAAAAGGAAAACAACGAAACAAGATACGGAGCACATG

>Cs1g11640

TCATGCATATAGTGATGATTCGTTTTGGGAAAGTCATGCTGTTAATATTATTGTTAATTATAATTATAGAAGAAAATTTCAAGCTATTAAAAGGTCTCATTATACACGCGTATGCATATGCATGATTTATAAACATTTCTATCTCTTAGTTAACTTTTCTTTTCTTTTCTTTTTTTTTTTAAAAAAATCATTATGAAAAACTTAAGCCTTGGTGAGTATTTGGGTTAGAGATCAGGTTAAATTAAATTAAATTTAATTTAATTTAGTCCTTATGAAAAACTTAAGTCAGGGTTTTTGCACACAACTTTTTTGAGATATTGAAGTAAATTAAATTAAATTTTAAATGATATTTGATTAATCAGATAAACAAAAAAGTGGGAAATGCAATTAGTCAAAAGCTATTAAAACTTAAAAATTAATTTTTTTTTATGGGAAAATAAATAAAATAAGCAAGCAAGGAAACAATCGTGAGAGGAACTGAAATTTTCTAAGGAGATAGGAAAGCTTCTAAAAGATTAGTGGTGATATGGTTGGAATTTTACGGATTAAAAGATTAATAATGATATGGATGAAAATTTATGGATTTAACTCTCGGTGAATTGAAATTGAATTCTCTTTTAATCAGAGCGGTTACGCTCATAGTTAGATGTTGAATTCTTTTTCACCTACGAGATTTAATTTTTTTGAGTAGTGGCAGTTGTGCACATTTATTAAAATAAGAATATGATATTTATTTGGAAATTTAACCATCTAATATGTATTAAAAAAATGGAGATATTAATTTTTTTAAAAAGTCATTCGTTATATAACTAAGAAGAAACGCAGAGACAAACAAAAGCAAATATATATATATATTAAACGCAAGCATATTAACTTGAAATTAATTTATAATAAAACATATTTATGGCATTACATGTGGCATGACATTAACTTTATTAAATCTGCCTTGTATCAAAAACACTTTTAAACAAACAACGAACACATCTACTCTGCAAACCACA

>Cs1g03590

CTTTTTTTTATTTTTCATGTTCGCTGATACTTCGGGTTCAATCATTAAATAAGAATAATTTCATGTAGAAACTGGAGTCGGTGCATTATTTGAAAATAGCTACGTTTTTCTGCTGTGAAATTCAGTATTGTACTTTATGTCTTTGTACTGGTAAATTGAAGATTGTCGCTCTAGGTGGGGGGAGTAAATCAAAATTTACCTTTATTTGCCTCATCTATATATCCATGTCTTCTCTGTTGTTTGCTGACTCACTACTGCGTTACTTCTTCGCAGAACGCACCATGTGATCCGTGCCTGGTGCATTGTTGCTTGCACTGGTGTGCTCTGTGTCAAGAGCACAGGGAGATGAAGAACCATCTATCGGAGAATGCTTCTACAGCAATGACCATTGTTAATCCTCCACCAGTTCAAGAGATGAATCCCGGTGAGAATAAGGAACCTGTTCCATCAGAATCAGCTTCTGTAAAAGACGAAAGCCCCAGTTTGGGAATACAGCCTCTGTAGGATTTTATGATTGCAACTCTCTGGGAATCCAAATTGTTACGACTTAAACGGGGATCATCTAATAGCTGCTAGGCAGTGAGGTTTTACATTTCATCAAAACTTGCTTTTTACGCAAGCAGCAAGATGATGAGATTTAAACATCAATTAATTAAGCCACTGCTTAGCTCAGCCCATTTCAGCGATCTGTACTTAACATATGATTGCTTCCAAGGTTTTCTAACTATGTAATTGATATTTCACCACTCATACCATTGGTTGGTTTGGTGGTAACATGGGGAATGAGCTGTAAGTTTTCAGAGACTCAAAATTTCAATATCTTGTTTTCATTTGTGTAGATTTTCAACTATATTATATAATTCTGATGATTTAGATCTTCAAATAGACCCTGTGAAATGTGTGGGCTGTTAATTTAAGCACTCACAAATACAACTTAATACGTACAATACCTATTAATTTAAAACATTAATACGTATTGGCATCAAACATAGATGACTGGA

>Cs1g13033

TTTTTTCAAATTTACTAGTGGTACTTTCATTTGGTAAACTATTCTATATATATACATTCTAATCAATTATATTGGCTTATTTTCCTTTTATTTTGATTCACGCTGCTGGTATATATAAAATTTATTTCAATTTTGGTGAAAATTTTGTTTTGTTGCACACAGATAATAAACCCAAATACATTCACATGCATATCTGGGGTTTGTTTTGTCTTTTAGGAGCAGCATTACTTGTCAAAAGTTCGAAGAGCTCTATGAACTTAAGCATTCCGGGTTGAAAATGGACGAGATATATGCAGTGGAGCTCATTGGATGAGCTACTATAGTGCCAAAGTTGCAGGTTTGCACCATCCGCTTTGTTTTGGTAGTTAACCCGAGATTAAATTCTTCAGGCATGTAAAGTAGTAGACTTTAGCCAATTCAAGAGTAGAATATAATATATTTACTTGTTTTGATCTTAATCTTTAATCACTTAGCCTGAATAAGAAATAACTTACTTTTAATATGTTATTTGGTGTTAATAGCTTCGTTTTGTTACTCTTTTCCGCACAAATTCTTGATCTCAAAAGAACTTTTATTTTGAATAATCTGCATAAGGACAATCTTAGTTAATACCATCAGTGATTTTTTATTTTTTCTCTTTAGTTTTAATATGTTTGATAACTATATAAATTGATATTGATTCACACGACTCAAATTAAATTGGTTAATTACTCTAAAGTATAAATCTAGCAGTCAATAATTACATAAAAATAATTGCAAAAAAAAATAAATTAATAAAAACAAATCATGTGGGCCAAGTGATATTTAATTCAATCAATCTTATCATGCCACATCAATTTATACAAAATAAATTTGTACAAAAATTTGTGGCTATATCATCACTCTTAATTTAATAGCAGTTCAGCAAGCGTAGGTTCCGAAAATTTCTTAGTCCTGATTAGGGTTTCACTAATTCCACCTGAAAATTGATCTCGCGACAGCAATTAAGACATAAAATTAGA

>Cs1g25990

CTCATCCCCTCCCCGAATAATAACAGGGGATCCCCGAGGGTTCCAGATCCTCGAATAATTAATATATTTTTTTTGTTTTCGATTTTGAGTTAATCATATTAAAATAAAAAATTCAAATAAAAGTAAAGTTCGAAATATATCTTACATTAATATCCATTACAAAAGTCACATACATTAAATTAGTAAGTAACACAACATGCAATGGATACAAACTATCATGAACAAATTAATAGCCTAATACAAAATTGTTACAAATAAAATCAAATTTAGATTCAAAATTAACTTTTCAATGGTGGCATAGGCACTTTATAAATGTGGACTATTCCCTTTACAACACAATAGTTAAGTCGAAGCAAATTAAGAGCAAATTATAAATGGAGATTCCATCTCATCCTCATCCCCTCTCCGAAGAAAAAATTAGGTAAAAAAATTTCTCTGTCCCTTCTCCGAATAAGAAATTAGGTATGAAATTATCCTCATACTCTCCCCAAATGAAAAAAATCTCTGAAAATATCCATCCGGTAAAAATTTTTACCATCCCTAATTTTGACAAGTTGATTATGGTTAGAGATGAATTTGGGCATAAAAACGAAACTACTAGGTCTGGCTGTGAGCCGGTTGGGTGAAAAATCGGAGCCTGGCACACATGAAATTGTGCTACAGTATACAAGTTCCAAAGCCAGTTGCGTGTGTGCATTTTAATAGTGTACAAGTTCCAAAGCCAGTTGCGTGTGTGCATTTTAATAGTTTTTTTTAAAATATTAAAATATTTAAAATATTTTCTACTAATTTAACAATCTCATTTTTTTTTTTACAACATAATTTTAAAAAATTTATTTATTAAAATAATTTTATCATTTTTAATAAAACGTTACTATCTTAGGTCCTTATTCTATAATTTTAATAAATAAAAAAAATGGGGTCTCATTAGAGTTAACCTCTACTCGGATGTTGGAAGTGGAACACAATTTGTTGATAAGTACAGTAGTATATAAGCTAAC

>Cs1g15540

CATTTCTACCATCTTAAAACCCCAATTAGCATTCTTTTTTTATGTTATGCAGAAGTATCTTTTTAGATAATTGACATAATCTACATTATTAACCCGTTGTGTGTATTTTGGTGTTCCTTCCTATCCCCCGACTTTGTGTTTTCAACCCCCCTAATATATATTGTAATACGTACAATAGCTAATGAAAAATGAAAATTTTATAGGGTTGGTCTTTTAAGCCCCCTTCAAGCTAGGATGACCAATCGACTTGTCTTGGGGTAACGGGCTTCCTCCACTTTTACTTTTGTTTACTTATTCTTAATGTTTGTACATAGGAAATGAGGCTTAATCCATATTTACTGCAAAGAAGGTGTTTTCTTTCACTCACATACGACTATCTAATTTCTTTTATTAACCTAAAGAATAAATAAATGAATAAAAAAATGAGGATTTCTATTTAAGTTCTTTTTTTTTTCCTAGAACGAAAAGTTTAGGTTTTAGCGAATCGCATGTTGAGATATTGATAAAACACATAAACTTAATGGTATTTCATAATTAATCGATTGAGTAGGATCTTCCAAACCACTAAAAAAAGGAATATTTATTATTTGATCCATATCCAGACATAAGAAGTCCGATAGAGGTAATACTTGAAATGGCAATCCATAAAAAGATATCGATATTGAAGTCAGCTAAAACAAGGTTATAACTAAAAGGAATTACTGAGTAACTTAGTGGAATTGCTATGACTGCTATAGATGGACTAATACTAAATAAACTATTATTTCCTCTAGATGGAAGAAGGTTTTCTTTGAAAATGAGTTTTGTCCCATCAACTAAAGCTTGAAGAATCCTCAAAGGGCTGGCATATTAAGGCCCAATACGCTGTTGTGTTCCTACAAATACTTCTCTTTCTAACCACACAATTATACTGATTGTGATTGACAATATATAAATTGTTCTTCACATAGAAATTTTTCATTTATATTTTACTAAAATTTATTGGAGAATGAAGTGGAAAA

>Cs1g15350

AAATAATCTTATCAATCGTGAAGGAAATAATAATCTTGAACCATTGAATTTTATCAATCATTTAGGAAATTGTGATTTTGAACCATATTCATTTTACTATTTTATGCTCTATATGATGAATATCGCATCACCAAGACATTTTTTTTTTAATAAATCTTTGAAAAAAATTATTACATGATGTGTTATTAATATTACTTATAATAGTGCATTAGTAGTATTGTTCTTTATTAGCAAAATGTCTTTGAATCTATTGATTTTGATATGATGTTAAAATTACAAAATTTAAAGTTTCCGTCAAATATTCACTTATTTATAGTCTGCAGTTGAATAATACGTATTTTTATGTAAGTTATTAAACTGATAATGCCTATAAGTATATTAGATAAAAAAAAATTTATTTAAGTTAGGGATGGAAAAAACGCCCAAGTTGAATTTTAGCTCGGTTGACTTGACTTAAATTTTTTAGGCTTTAACTCGAATTTAAGGATCAAAATTTGAGTTCGGGCTTAAAAATATATGTCCAATTAAGTTCAGGTCAAGATTTGACTATAGCCAACCCAACCCAACCCAATTACTTAATAATAATAATAAATATATTTTTATTTGAATTAGACATTTTTTTTTGTTTATGTTTAGTTATTTATATAATTTTGTGGTTGGGATGTTAATTAGATTAATTTTTATGTTTAATTTTAATATATTAGAATAGGTAAATTTAACTTATTTAAATATTAAAAAATTAAAAATATATTTAGTTGGGCTTGGGTCCTGACCCAATTAGAGTCGGGCTTGGTGAAAAATTGAAATTTTTATCGGATTCGAGCCCTTTATTTATGCTCAGCCCAAGTTCGACCCAACAAACTACCGTACCTAATTTAAGTATTTCCCCAAGAATTTGGCCGAATAATTTTAAGTAATTATTTTAGTAATGAGAGCAATAATCAGTTTTTTTTTTTTTTTAATTATATCAGAAATAAGTTTTAAAAAAATAGTAATTAATA

>Cs1g09080

CCTTCTTTAATTTAGAGATAAAGAAACATGGAAAAATTGTTTTGGTATAAAATGTTAATATGCCCAAACAATACAAATGTGAACTTTGTTAGCCAGTGTGGTGAGAAACGAGATGAATAGGACTTGGAGGTGGTGGGGTGTTGGTGCTGATTTTTCAGATTGTTTGAGGTTAAGATGGGATTTTGGAGGAGTTGGGGGAGTTTTGTGCAAGGGTGGTATGATGTAATTGAATAATAATTTAAGCTTAGATTACAGTAATTATCCTAATAAATTTTTGTCTGAATTAATATTTGCTGTTCAAACCCCCTAACCTTACATAGTCGCTCCGGTCATTAATTATTTATTAAGCAATTATTTATTACGTATGATGTCAAATTTACATAAAATTAGATGATGATATAATAATCAGTTTCAGTCAAATACACAGGGGAAATAACGATTGGAGTCCCTGAATTTTGACAAAATCACAAATATAGTATACGTTTTCTTTATACGTCCCTACTAATTTTGACAAAATCACAATCACAAACGTAATACTAATGGCGATTTATGACACGGCTGGTTAATTTGAAGTCAGCACAATATAAAAATTAATCTTTTCAGATTATAATATGTTGAATTCGTTTCATCTATTTAATAATTATGTCGAGTTAACCAATCAACTTTAATGTGAGCTAATTTGTGATAAAATTAATTATTTAAACAATTAATACAATATCTTATCTAAAAGAGTTATATTTTATTTTATTCTTTTTAAAAATACTTAGTTATATGTGGACGAAAACTTATTAAAGTTTGAATAGATAAAAGTTAAATTAATTAGAGTAGTATCACGTGAACATCGTTTAAGAAAAGAGAGGGGGGGGGGGGGGGGGAGATAAATAACTAATTCACTATGGTGGCGTTTTAGATAATTTTTAAAATATAATGGATCAATGGGTAATTATTTCAGACTACATAGGGTGAAGGAATATTTTACCCCAAATTGTCTTTAACGTATG

>Cs1g08810

TTCAGCCGTTGGATCCACGACTGAAAAGCTTATTGTTGTTATTATTTTTTCTGGATTATCCACCTTATTCTTGAGGCATTCTTTGAAACGACGACAGGGGAAAGCTACTCATTTCTCACCTCTATTGGATATTATAATAATTATGATTATTATTAATTATCTGGTAGGAAAGTCTATAATACAAATATATACTTTTCAAAGCACAGACACGCGTTCCCACTTAACAACCACTAATTTATATGATATTAACTAGTGTCCCTGTTGAGCAATGTTGATGGCACCGGTTTGATGGAATTACTTTTCTTCAGTATAAAAATACAGAATATATATAAATTTTTCTTTAAATACTATTAGTGGAACTAAATTTTTAAGTTAATCTCCATAGATTTTTAGCATATATGATAAATATATGTGTTTAGTCTCAATAAACCTAAAATTGTAATTTAAAAAATTGCGGTGTTGGACAAAATTCATGAGTTTTATCAAAATTTAATATAAAATAATTCAGTTTAATCCCAAATTATTTATATATAATCGTTCATTTAATTATCAAAAAATAGGAGTTGAATTGATGGACTCCATATTCTAATTATACATGTTACATCTAAAGCAATTTAAATAGCCAAAGAATCATAATTGGAGAAGATCATGGCAAATTCATGCTGGCATGCTGCTTCCAATTTCCAAAAAGAAAGGAACGAATTATGTGAGTCATAGGAGCTGATATGATGGAATTTAGATCCTAAATTCATTTTGTCTTTGATTTATGTTTTTGATATCCATTAATAGGTATTCGATAAAAAAAATTAATAATATATAACTCACTAAATTACACATAATTATAAATGCAAATGAATGATAAGATTCTTTGGAAGCAAGTAAATATAAAATATAAAACGAGGTGAAGTCAATTTCGTAGTCAATCAGCTTTAAAAGATAACCTAACATTAGTTGAGTGGCGTTGACATGCAACTAACAAGATAAAGATAGTTAAATTCGAA

>Cs1g05520

ACAGCTGGCAGAACTTGAAGAAATTCGCAATGACGCGTACGAAAATGCCAAGATTTACAAGCAACGAATGAAAGTCTTCCATGATAAGCAAATTATGAGAAAATCATTCACTCCAGGTCAGAAAGTGCTTTTATTCAATTCTCGCTTGCACCTATTCCCAGGTAAGTTACGCTCTCGTTGGTCTGGCCCATTTATTGTTCATACTGTTTTTTCACATGGGGCAATTGAAATTGAGGACCCAAAGAATGGTGTCACGTTTAAAGTTAATGGTCAAAGATTAAAGCCATATCTAGAGTACCAACCACATGACGAAGACACCGAAATAAATTTGAGTGACCCACCAAATTTGAATTGATTTTTTTTTCTTTTCGTTGATTTGATTTTGTTTTCTTTCTTTATATTATTCTTCTGCTAATTGAAATTATTTTTGCATAAGTGTGTTTGTTTAACTATTAAGTTTTCTTTTATCATTTTTACTCATGAGTACCTTACTTAGACCGTTCCTTCTGAACATTCTTAAACCACTTTAACATGTCAAAACTCATCTCAAAAGGCAGATTCAATTTTGTAAAACACATCGCATTTGGGATGTACAGCCACCAGAGTTAGTAGCCTATGTTGAGGGTTTAGAAAGTCAACTCAGAGACATTGAGAGAAGTGTTTACGACATCTAGTTGGAGCTTGAGGTAAATTCAATAAGAGGACGATTTTAATTTTCTTTGTGTGTTTTAATTTGTTTTTCTGTTTGTGTGCTTTAGTTTATTACCCTGGTGAAGTGGCGGATAACGGTACTCTGTGACAATCAAGTCGGTTACTTCAATTTCCCATAATAACTGATATTCTGAAGCGAAGGTATGGACAAAAACAAGACTTTAGCGAAACTTCACAAGCGATTCCCTTCACTTCCTCAGAATGCCCTCCTTACGATCTATAAAGCTCGGTCCGAACGCATGCGATTACTCATGAGGAATAACATACCTGCTGAAAATTTATTGCATACA

>Cs1g11980

CTAAATTAGCAGTAATCTCAAAAATCTGTCCTTACTAATGTTGCTTTGGTGGAAATCGACCTTATTTAAAGAGAATGAGCCCCATGCCATTGGGCTACAAGCAGAAGCGGCCCATAAATAGTAGAGTATCTAAAATAGTAAAATTATATCATCTCCATTGTCCAGAACACTTTATATATCCATTTCCAGTTTTCCACTATATATGCATCCCTAGTATACTTTAAGCGTTGAAGAGGTCTGATGGAAAGAAAAAAACAAATTGATTAGAATCCTTCGTTTCTAGGGGATCATAAAAGGCTTTATTATTTTTCTTTTTCGAGTGGTGCTTTTTACAAATAAAAATGCACAAAGCTAGTAAAAGATGAATCTATTCACTTTATCAATTCAATAATGATATAGTACAAATAATTTTATATAAATTGATATGTACAAACTGATGTGGCATAAAGAAATTTATTGAAACGTGACACATTATTTTTTTAATTATGTGAATCCACGATGGTTTTATAGAACCAACCTTCGATTTTTTAAGATTGTTAACAGCAGACGGGTGATATTCAAAATTATTGATCGAACAGGTATAACTTATTACCTGATTCTTCTTTAATTACAAGAATTCTGATCATCGAAGTTTTTTGAACCTTCCCAAATCAAACTTGGCTGTGGTTGGACTGATAATTTCTTTTAGTATTCTCACAGTAGATCACTTTCACAGTTAATTGGAACTCCACTCGTGAGTCGCGGACGCGGTAAGTGTAGACTTGGGAACTAGCTAAAGTCTTAGTATTAGGACCTGTTTGTTGTTATATATGATAATCAACACAAATCAGTCTAATGATTCAACGTTTATGAGTCGGCACGCAAGACTTAATTGGATAAGACTATTTTCAGCGTCAATCATATTTTATACGTTCTAAAAGAAAAGTCTTTGTTACTTTCTAATTAGTAAATTTCCTTTAAGATGAACTGTATCAACTGGGGCTAGTAATCAGTTGAGCTCA

>Cs1g05340

CCCTATTGAAGGCAAATAACGTGGGTTTTATTTTTTGAATTATTTGGAGCCTAGAGTTTTCACTAAAGGGTGATATAAAAGGCTTATTTTCTCTAAAGAAAAGAGGGAAGCCACTTTATACAAATCAAAGAATAAGATTTTTCTCTCCATGATTGAGAGAAAAATTTTCTCGTGCTAGTTGCTTTGGTAGTGATAAAGGTGCCCACACGTCAAGTGCAGATCGAACCTGAGTCATAACCTGGAAGATCATTGGTGACAGTTCGTGATCTAACAAGCGTGGTGGTGACAGATCGTGATCTAACAGGAGTGGTGGTGGCAGATCGTGATCTAGAAGCCTAAATCACTTCAGCGGAAAAAGCCAACTCGAATTTTCAAGGTACGATTTCTAGAATACGAATTCTTATATATTATATGAGAGCGATCTTCAAAAGGTTTTATAAAAATTTAAAAACCTGATTTTTCCCCAACAGTTAAAGCTTGAGATGATAGACGTTGGAGACAATCAGTTAATAGGAAAGTTCCCAGATTTCATCGCAAATTTTTCAGCTCTCGAGGCAATTGATATTTCCGCGAATATGTTAGGCGGGAGAATTCCCGATAGTCTTTGTCAACTAAGAAGCTTAAACTACTTAAGCATAAGTGAAAATAATTTCTCCTGTAAGCTTCCTCTTTCAATTTGGAACATCTCTTCCCTTGAGATAATTTCTCTACATTCAAACAGATTCGAAGGAAGTCTTCCACTTAACATAGGTTTTAATATTCCAAACGTGAATTTTCTTAGTGTAGGTCAAAACAATTTCACAGGTTCTCTCCCACACTCATTTTCCAATGCTTCAAATCTTCAAGTGCTTGACGTTTATAAAAATCATTTCAGTGGACAAGTGAAAATTGATTTCAACCGTCTCTCGAATCTGTCCAGGCTTTTTTTAAGTAAAAATAATTTAGGAACTGAGTCAACTAGTGATCTTGATTTCCTAACTCTTCTCACAAACTGTAGCCAA

>Cs1g16026

TATTTAGGTTTGATTAGGTCCAACCCATTTCTCAGCACATTGACCAGACATTTGACTTTAAAAAAAGAAAAAAAGCAACCAAGATCCTCAATCTTTGCTTAAATGTCCGTAGACTGCCTTAATTTCGGCTTTATTTGGTATTGAGGTTGGACAACTATAATTTTTAATGTACAATATTAAAGTGTTTAATAAACACTAACTATTAGAACTTAAAAGTTAAATTGATTTGGTCAATTATTTTTATGATGAAAAATTTATAATATTTTTACTATTTTTGTCACAATTATTATTTAAAAGTTATGTATAATAATATATTATTAGCTTTTATTTAATAACTATAATTTTTTTCACAGCAACTGAAATTTAAAAATTATAACATCTCAATACCAAACATAACTTTAAGGTAGTCTAACAATCTCTTTTGCATATAAACCTTAGGATTATTGTAATTAATTTTGAAAAAAGTTTATGTGACCATTTTTGAGATTAAAAAGATCTTTGTGTAAGGTAAGGGCCGATCCGAAGATAGTTACAAAATCCAATAGCACAAATAGTATAAAACAAAACTTGCCCTAAAATAATTACAAACTGACACCAGTGGGGATAAAATATTGACGACGGCATCGTTTTTCTGAAACAGTGTGCAAGGACACAAGAATTTGTCAACTATGAAACCTCATCAAACTTTCATTTTCGCTGTCGTATCAAGTAAAATTGTTTATGCCAAAGCATTTCCCGGAAGACACACACACGCACACATTATACAAGTAATCAAACAAGTGGAAAGCGAGTGGCATGGCAATAAATTCAAATAACATTAAAATTGCATGGTAAAAGTCCAATTTTGCTGAGGTGGCTACTTGAAAATTTCAACAAATTAAGACAATTTGGGCTTCAATTATTTTTATTGGTGGGAATTGAACCTTTTTTATTCACACTGTGATCAGTTCTTACAAGCATAAATATGTCTCCTTTGCATTGACAATCTAAGCAAAAACAGT

>Cs1g15230

AATTCTTTTCGATATTCTTCACACTCTCGCTGAACTTGTTCACCGCCCCAGCAAGATCTGGGAAATTCCCCAAGGACACTTTCCCACTAAACCACGCCATCCCCGAGCTACAACACAGAGCTTAAATCCACTGATCCAAAAGCATAGCATCCAAAAATCTGCCATTTAAACACACAATTCAGAACTCGACAGACAAATTAACCAATCATTGAGAAAAATCTACGATTCAGAATGTGCTAACTCTAACACGTTAATCATATACGATAATCTTGATTAACCAAAATGAAGCATTCACATTTGTAATTAGCACGTGGAGATAAAACCTATATACTTGGAGATCCAAATTGAATAAAAAGGGAAAATAATATAAATTAAATTAAATTATAAATCTTGCACAATGCAAATCCTAAAAAACGATGAAGTCTTTATTCGAGTTCATTTTATTTTCGTACCGTTAAAGAGATTGGAGATGACGATGAATGCTATGAGATTGAAACCGAAAGCCGAATCATCACTCTCTTTTGTTTTCACCTCTTAAGCTGTTGATGAATAATGATATCGACGTATCAGATTTGAGCGTAATAAACTTTTGTTGAGAGAGAGAAACGAATGAACGATCAACGATCTTTAAATTAGTATCGGTTTGGAGAAAGATTCGGGTTGAGGGATCGATCTGATTATTTTCACTGTGAATTTTCATGGGGCTTGCTGACAAGTGAGACGTCGTCGTCTTGATAACTCTTTTTAATATTTTGTTTTATTCAATTTACAAATATAATAAATTTTTAAATCAATAAATACCAAATTAATGTAATAAAATTGAAATAAGTGCATTTCGAATAAGAATTCCAACCATTTCTAGGATCCGCCTGCCGTACCACTTCTTTTGTCTTTTGTGACAAATTTTCATACAACTCCTTTCACTCTTTTTTATTTAAACTTTACTTTACAAATATTAAGTTAATTTCAACTGCATCCATGTGAACATTACCGAAATACCC

>Cs1g15020

CCAGAAATAAAGCATACAAAATCCATTCTTACACAGAGCTAACGATCAATCGAATTGCAGTAAAGTATCTCACTTCAACCAAGAAATCTCACTTCAACCAAGAAACCAAAACAGCTAAGAAACCAAAACAGCTAAAAAAAAAAATCAACATCCAAATGCATAAACAAACACAAACACGAATCTACTGATAAAAAAATTGCTTTGAATGTGCATATAAAGAAGAAAATTTAAGAAAACATACACGTTCTCTTGCGAGCAGCGGGGTAAGCACAAGCAGCGCAACGACTCTTCTGAAGATGGAAGCTGCGACGGCCACATCGCACACACAAAGTGTGTGTCTTGTTTCTCCTCTTACCGAAACTCCCTGTTCCCTTACCCTGTCATAACCCAAAAAAAAAAATCCACTCACAAAATCAATAAATTTCAGTTTTCGATCATTTTAACTCACGAAAATTGCGCAGAAATAGAAACGAATGGTACATGCAGAATCAAAAGAGCAAAATGGGAGACGGGTTGTGATTTGTTTTTACCATCGAAGCGTATGCGAAGCAGAGGAAAGCCTTCTGATGAGTGAAGAGCGAGCGGCGGAGACGAGAAGGTTTATATTGTGGCTTTGTTGCGTTAAAAAGGAGGGTTTTTTTCTAGGGTTGGCTGATGAGGGTTGAGTGAGGGAGGCTACGTGTCAAGATCTTGTTGGGTTTGATTATAATGGCTCTACTAGTCTGATTGGGTGATTCATTAACTGGGCCTGGATGGGCTTAATTTTTGCCCAACCATACAAGCCAACCCAGCCCATCTTTTATTAATATTAATTTGTTCAGTGTATTTTTTTTTTTCTTTCGCGGGAAATTAGAAAACATTTAAGAAAAAGGGGCGCCAAACAAACACATCGATATACACCATTTAACTTAAAGCAAATAATCAACAAGAACCCTTTTTACTTACAAAATCAAGCGCCAAAAAGAAAAGAAAAAAATTGAAGCAAGATCAGAAAGAAGAAG

>Cs1g06370

TGAAATGAAGTCAGCCAAGAGTGGAGAAGTGGAGTGGGAAGATAACCTTGGGTATTTCATCAAGAAGGTCATTTTTTTTCTTGCTGTTACAATTGTGCTGTTTATTTTGTCACTCTTAAATTTTTACTTTTGTTGTTAATTGAATGATTTCTTGGTTTCTCTTTTTGTTTCTTAGTGGTGGCAATGAATTTTGTGTGTTAAATGAAATTTTGGAGTTATTACATAGACTTTTTCCATTAGTTAACGTGACTGATTTTATTGGGTAAAATGAAGGTTTTAGAGAGCTTCTGCAAGCTTTTAAAAATTGGGTATTTTACCAAGGGGTAACTGAGAGTAAAACAAAATTTTGTTCATTTTATATTTTGCTGGCTTTTTGGTTATTTTGTTAATTTTGGCTATGTTAGAGTGGGTTTGAAGTTGTGCTAGTGCATATAGACGGGTTTGAACTCTTGTAATTGGTTTTATTTTCACATTTCATGTGTGAATTTGAACTTATGAGCTTTAGAAGGCTTTTCAATGCTTGATTTATTTTTATGAATTATTGAGCATTGTAACTTAATCTTGGGAGGGTTTTTAGCTCTGTAACTTATATTGGACCAAGAGAATTTTTGTTCTATATATATGCATGTGATCGGTTTTGTTTCTTTTTGTATTAGGAAGCAGAAAATGTGACCTTAAAAAATGGGTAGGACATTGGTAATTGCTGAAAAATTGGATCGTTGCTATTGATTTCTACCACTAGTTTTATTCTGTGGTCGGATATAAGTTAGGAGTTGATAGTTTATGTTTGATATAGTTTGTCACCGTAAGCTGTAAACTTTGTTTTCTAAATGATGATTTTGAATTTTGTTTAGAAACTGCGTGTTTGGTGTCGACTTGAGGATGGAAAGTGGGAATCTGGAATGATACAGTCCACTTCAGGGGATGAAGCATTTGTTTTGCTCTCCAATGGAAATGTAAGTTCATAAGTTTTGAAGGTCTCTAAAAGCCATACAAAATTC

>Cs1g05150

GTTCCTCACCAACTGAAAGAAACAAAACTTATTAGCTAAGATTCAAGCTAGCTCACTGTTAGGAATATTAATCTAGGCAAAAATACGCTACCTGGACATTATTCCTACCAGAGAAAGGTGGGTAGCCATTAAGAAGCTCAAAAAGAATTGCCCCAACGCTCCACATATCAACCTACAAAGAAAGTTTTAAAACTTTCAGTACATTCTGATGCATTTTAATAGCACAGAATGATTATCTAGGGGGAAATAAAGAAATTCAGCTTCTCATCATATCTTTGAAATTGAAGAACTTCAGGAGCCATGTATATTGGGGATCCACAAACTTTCTCAGCATAATGGCCTGGATGCAGGGTGCTGCTTAAAATACGAAGCAAACAGTCAATTTTAAGCTCCAGACTTTTCAAGAATGAAACTTCTGCATAATGTTTACATTTTAAAACTACAATAGAAACAAAAGAACAGAAAAAGTCAACATTCAAACACACTTCCAGGACATTGTCAAAAATAAGAACTATCCTCACACAATATATTTGTATCCTTCTACTAAAAGTTTACCATGAAAGACCAAAATCTGCTATCTTGAGCATCACATCATCATCTAGGCCGAATAATAAAATGTTCTGTTCCACCAAAAGCAACAGCAAAGTCAATTTAAAAACTTACAATCTCGAAAACATACAATGTTTTAAAATGAAAACATATAAATTAAAATGAAGAAAATTCATGTGTAGAAACACTGAAAATTTGGCAGTTTAGTGACAACTATACAATCGCTGACGACATTCAAAGTTCCTTTGAACTTCCCAAAATAGGAGTACAAAATAGTAACTCATCAAACTTTAAGCTTGTTCAAGATTGTTCATTTTCTCGATTTCATCACTTTCTTGACAAGCACACTAAAGAGCAAACCATATAAAGCAACCTCAATCCTCTAGGGAACTCCATCCTCTATGATTATCACCAACCAAGACTCTCATTTAATTATTCATATTATACTTCTC

>Cs1g08760

ACACACACACACACTTCTTGTCACCATCTTCGACTGAAAATGAGACACTAGCTTGTATCAAAATCTCTACCATTACAAATTTGCTGCACCCTCCACAAGTTCTCTTTACAGTTCCAAAAGCTTCATTCCATGTACGGACTTGTGTCTTGTAACCCAAAACTTGGAAGGGCAATGATCACATTGAACATTGTAACAAAATAATAAAAATTGGACATAGTAACAAAATCATAAAATTTGTTATGGCTATAATATACATGATGTAAGATAAATGAACCATTTCCCTACATGACCACAAAGGGATAAGCTAATTGACCATGGGTTATTTTTACATACCTCCCTTGAGGTTTCACATAATATCAGTTTAATAGAAAGTATCTCCATATTTTTACATCCCTCCCCTGCAGTTAGTATTTCCGTAATATCAGTTTAATAGGGATAAGCTAATTGACCATGGGTTATTTTTACATACCTCCCTTGAGGTTTCACATAATATCAGTTTAATAGAAAGTATCTCCATATTTTTACATCCCTCCCCTGCAGTTAGTATTTCCGTCAGTTGACCGTTAGTGGACTAACAAGAGGACGAAAATACCCTTTCAAGGTTGACAGTCTTCTTTCATCCAACAAAAACTCAAATCCCCTGTACCACCTCACCAACAAAACCACATCCTTGTTTTCTTAAAATCAAGCTTTCCATTTTTGATATTTTTCCCCAAAAATTCAAATAAAAAATCAATTCGAAAAAACTAAACCTCATAACATGTCCAACGTCATCTAACTTTAGCTTGGTATCCATAACAACAGCACCAAATGTTGCTATGAAAGCACTGTCGACTAAACTTCAATTTATGTCCGCGGCACAACCAAAATAATTTATATCAATTAAATTAGCCAATTCTAAAAGCAAATAAACACAATTCGTCCCTCTGCCAACAACAACCTGCACCAGTGAGAAAAGAAGAAAATAAGATCGGTTCCACGAAGAAGAAATAAAAAGAAAA

>Cs1g09363

TGGAGATCTTTAATTAATGATTTCTTTTATTATTATTTTTTATAGGACTCTGTCATATCCCTTGTAGCTGATGTACGTGTTAACTACTATTGACAAAAATATCAGTTTAGTTCATTAACCTATAATTAATGGGAAATTTACAAAAATAGCCATACAATTTTAGCGTTTTTCAATTTTAACCCCCCCAAATTTTTTCTATCAACATTAGCCAAATCACCAAATTGTGGACGAAATTGCCCTCATCATTCTCACGTTTCCCTCAACCAAATTCCTCCCCCTGTCACCAAAATCTCATCACCGGAATCCCGTATACATCGTCAGTGGCATAGATCGGCAGCGGAGGAAGTTGTCGGCGGCTGAAACAAGCTAAATCACGTTGAAAATCTTTGGAGATCTCCAAAATCAAAGACAAAAATTGGAAACCCTAGGTGAGTTGTTATTGTCGTTGTTGTTGTTGTTGTTGTTTGGGCTGTTGTTGGTGTTTAATGGCGGGGGTGGGTGAGATGCATGTGGGAAAGGAAAGGGAAGAGAAAGTGAAGAGAAAGGGAAGGGAAAGGGATAGGCGCATGCCGCGCGCATCTCGCGTGCAACAGTTGCTGCCTCGTGCGCGAGATGCGCATGCCGCGAGCGAGCTGCGTGCCAGCCTACGCGCGCTTGGCGCAGCCTCTTGGTGCGACAAGCACCCCGTCGCACCAAGAGACAGCGCGAGTGGTGCTGCCCTTTTTTTTTTTTGCTTTTCTGCTGTACCGTACATCCGCCTTGGCCGTACAGCAGAAGAATAAAGCGTAAAACTGAAGCATACATCAAGACGTTGTTCAAAGAAAATATATTTAGGCATTTCCTACTGTAATACCCTAGACCCAATTTTTTGACCCTCCACCCAGATATACATATAGAGAGAGAGAGAGAGAAGAGACGAGAGAGAGAGGAAGAGAGTGTTTGACTTGGTTTGACCCGGATCCGACCCGGAAACCCGCGACCTTGACCCGGCGATATCTCCC

>Cs1g21680

TATATCCACTAGGAGTTAGGATCTGGTTTAAGTTTTCTTGTAAATCACCGGCATAGAAGATTGTAATCAGCACACTGATTATCAAATTGAATTGGATGGATCCAAAATAACAATCGATTGAATAGACTTGTCCGATATTAGACCAAACTACAAAATGATCTAAATTAGCATTTGCTCTGAAAATGAATATACTGCACCAACAAACGCGTAGATCAACTAGCATGTGGTTGTTCTAGCTTAATCAAGGTCCTCGGTTCGAGTATTGGGAATGCAGCTGCGTTAAATACTTATTGGGAGAGCTTTGCCGCCCTAGTGGTCCTACCCGGCTCGAAGTTGGATTAGTCGGGACCCAATGTGATTTTCGAATACCAAATGATTTAATACACCGAAAAATATACTGTAAGAATTCTTTTTTTTTTTTCAGTTCTGTTTGACTTTGACCCTTCAGATCCAGAAAAATCCTTGAAGTTTTACACCCATCGTAGCTTAATTAAATGAAATGCAAGCAAATAAAAAAGGTTTTGCACCCAATGGAACAAAGAAACAAACGTATTGCACTGCAATGAGAAGCAAATAGAAAATGGTTTTTGTTATCGTAATAATGAAATCTGGTAGCTAATTAACGTTCAGTTGCGTTAGCTCGGTCAAAGGGCACACAACGAGTCGCGACATTTATCTTCAATTGCACGAGTTGGATTGTGTCGCTGTTAAGAGGCGGCTATAGCACCTGGACACATTACCAAAGCGATGACCGAAGGGAATACGAATTAATAGTAAAGCGCACCAGCTGCGGAGACTATAGGGTAATACTACGGATGGGACCCTATATTTTATTGTTCATCAACAAAATTGGTCAAAAATTGAGCACTTTGACTACGCTGTTGTACCATGTCAGCAGTCACATCTGTGGCGGCCGGTCTCAAATTTTAATTAAAAATAAAAAAAACTGATATTTAAATTTATGGGTTGCCTTGCCTTCTCAACGGTTGCATTAATATCTC

>Cs1g22850

CCATCAACAAAAATAAAAAATAAAATAACTACCATTGCCAAGTGGGTTTTATGATGGCACAGAAGAATCCCTATCTTTTTGGTGCTTTTCGGTGATAGTGGAGGATCCCAACTTTGCTCATTTTTGTATTCTGTTATTAAAAACTAACTACGATTAGCTTTAAAAAATAAAAAAATAAAACTAACTCTCCTTGGCTTTTCAATCGTACGTTTTATGTAAACATAGAACCGATTAAACAGATTGCCGACCTCGCAACATTCAAACTACTATAAGTTAAGTAACACCCAGCATAATGAAAGGTCAAGGAGGGGAAATAATCAAAATACCTAAATTGTCTCAGTGAATTAATTTATTTCTCTACTTTTTTGTAAACTTAATAAAAAAAGGAAAAAAGAAAAAGAAAAAGGTATTGAGTTGATTTTTAAATTTTTTTATCTTTTAGACTTTAACTATTTTACCTTTGACGTAGTAAAACTATTGATCAATAAAATTTTTTTAGGGGTAAAGTAATAAAAGAAACAGAAAAGTTTTAAATGCTCCTAAAATACTATTTTATTATTTTTGAGCTTGGTGGGAGGGAGTGGGCGTGTGTGTATATATATAAAATAAAATATAAAATCAACAGTTAGTCAGTGTCACTGTGTCAGTCTCCCATTAAAATATTGTTATTAACATATAAAACTTGGGAGATAGATAATGAATGCAAATTGCACTAAGTTAGAATGATTATATGCAAAAGAGGGATAAAGACAGATCTTCGAAATATACAAAGTAATAGAATAGGGAGTTTTCAAATTAAATTAAAGCTTCTGACACATACACCGTCCATTTGATAGTGGTCCGCATTTTTTGTTATGAAATAATTAGCGGTTAGGATACTTAATGTGCATACATTTTAAGATTATAAATACCTGAGGAATCAAGAGCTTGTTACCATCGTCTTGTAGCTTTCTATCTTCAAGTATTTCTTAAGCCTTTCATTCGATATCCTTCTCTAACAA

>Cs1g26375

CAAGTTAATTTATTAAGAGTCTAAGTTCATATAATTATAATATGTATAATTAATATTGTGAGTCTGAACGACTTGAAAATTTGTATTTAATAAAACATGCTCTATCAAATACTTATAACTATTTATATAGGGATGTGAATAAGAACTAGAACTGCATTTTTCTAAAATTGGAGGGGAGGGGTTTATATTGTCGATATTCCAAAGACTCTATTATAAATTACAATTAAGAGATTTTGGAAAAAAAAATAAATTTGGTTTAATAGTTCAAATTTGAAATTGAAGAATATTCATGTCGATCGATTATATTGAAATAGAAGTACATAAATCTAACCTACATAGTCTGGCACATCCATTCTCACTACATAAGAAAATTAAGGAGTGTCTTAAACTTAAAATTTATTTTTATTCATGTAAAAACTTATATGTATTTTCTTAAAATACATCAATTATTTAAGTTCTTAACTCTATGAACTTAACTAAAATGCATGTCCCCATAAGTTAAAAAGATTAAATGTATATCCCTTTGAACTTAATAAAAATGCCACATGAATTAAATTTGTAATTATTTATTTCAAGTTCATAGTTAAGTTGCACTTATAAACTAAATTTCAAGTTTCGTCCCTGTCTGAAATGAACTCTTCTAAATTTTTTAAAATTCAATACCTCGTTCGAAGATATAAAAACGTCCAGGAGGATACCTAATTGTAAATGTTACACAGGAATGATAAAAAAGAAGAAAGTGTTTAAATAAAAAATAAAATGAAAAAGGAAAGAAAGAAAGGAGATCGAAGAGAGTAGTTGAAGACTTGCACAGTAGCCTGTAACCATGCAACTAGCTATGCGAAAAGGCCCAAAGGGTTGAACCAATCACAAGCAAGCAAGTATAATTCCATTAGAATCACTCCAGAGCTAAAAATGAATCTGAACCGTTGGATAAACCTCACCAGCAGTGGTAGGGTAAATGATAATGCCCCCATAACGACATCCATTCTTCCACACAC

>Cs1g09030

TATCATAATTTTCTTGGACCCCAAACTCATAAGTAAATTATAGCAACTAAAAATTAATAAAGCACATACACCCATGTAGATTCTCTCTTTACCCCTTTTCCTCTTGCCAATTTTTTCTTTTTTTTTTTAATTAAATATTTAAAACATCACCAACTCCAACTACATGTACTCGCACGCACACGTAAACAACACCCACTCTCTTTTTCGGATTTATTTATTTATTTATTTTTTAACAAAATCTCTCCCTCACAAAAAGAAGGAAGAAAACGGAAAAAGAAAATAAAAAAAAATAAAGTGACACGTCATCCTCTTCTCTCTATTAAAAAGTGCCACGTCATCAGCCTCCCATTATCCAACCACCGCTTGCCGTCCCCTTTCTCACACAGCCTCCCTTAGCTGTTCCTCACTGTAGCCAATGGGCCCCACTTCACTCTCTCTCTTTCTCTGTCCGAAGCCATTTCTCCATTTGGGATAATAACATGCTAATCCCTGTTCTTTTTTCGCTCTTTTCTCACACTTGCATCAAATAAATCCAAGAGAAGAGAACTATCTTAATGACCAAATTGGGCCTGATTTGACATTATAGTTAGCCAACTGTACTTTCTGAAACTATCACATTAAAGTATTTAATAACACTTATTATTATAATTTTAAAAATTAAATTAATTTTATCTCACATTTTTATTTTAAAAAATTATAATAATTTTATTATTTTTGTAAAAAATTTAAATAAAAATCATTTTTATCGCATATTATTAAGTTTTATTTCGTACTACGAAGGCGTTGTATTGCTAATTACTAACTGGGTACCTTAAAACCATATAAGACGTTGCACAATTGAATTTGACTCGACTTGTTTGATACAGACATAGAAACTATATCAATTTCTGAAAAAAAAAAAAGGAAAAGGGGTCTCAACATTATTAACCCATTTCTCGTTTCCTTCCTCTTTCCTTCCTCATATATAATCTGCTGTCAGAACCCAAATCAAAACTTTCCTT

>Cs1g02620

TTTCTTTATTTGCAATATATATATAATTGATGACTATGAAACCAATTACACCGTCCAGAAATACTTGTTTAAATGTATAAGATTGAAAAAAAAAGGCTAACTTGAGAGCTATAGGATGCATATAAAAGGGCAAACACCATGGTCAATGGAAACTAAATGGCATGGACAATATAAATACATACATACATACATGCACACACGCGCGTGCGCACATATACACACACATATACATACATACATACATACATACATACATATATATATATATGAGTTTTCAATCAATTGACCCGAACTGAACGCCCACCCCAACCTATAATAGAAGATGTGGAGGAAAGAATTAAGGAGATGGCATTAAAAATAGATAAATAGAGATACATGGAAAGCAAAACTTAAAAAAGCCAACATAATTATTTGGTGGGGCTAATTTTTATTAATTTTTTCATTTAACACTAATATTGGTTATCAGATTAATTCTGATTTAAGGATTGAAATTTTATGTAAAAATTTGTGTTAGAGAAAGGGGCTCCAGATCATGACCGTGTATATGTATGTATGAGTGTCTATATATACACACACAGATGCATACATACATACATTCATACATATACATATACATATACGTCCAGGCTCAGACTGTTTTAGCCTCCTGGACGTGCAACAAGATCCATATATAGATTTTGACGTAAAGTTCATACATTGATATTATATTATTATTTATTTGTCATCATTGATATCATCGGCGCCTCGTTAATTTTGTAGGTATTAGCTAGGAAAACTGAACCGTCTTCCCCGCCAGCTGCACATCACATCATCTTTATCCTTCATGATGCCGTGATCTGTCCGTAGTTATTATCTCTTAGCTAATTAGCAAGCAATAGCCAGTGCTGATTTTTTTTTAAAATTTACTTTCATTTTATTTTATTAAAAGAAAGAAAAGAAAATAAAAGAAAAGAGAACCCGAGTGTGTGTCGGGGACACTTAGTCAGCAAAATTATGCAATT

>Cs1g06520

AATATTGTACTAATGTTTTAACATATAATAAAAAATAAACAAAAATAATAAAATATGACTATTTGATGGTATGGATATTGTAAAGGACAATAAATAAAGTATATAAAATTATTTGTGAGAAAAGTTTAACATATTCTTACTTTAAAAAGTCCTCTCATGATTTTGATCTTTTAAAAGTTTATTGACATGTGACGTTACTAAATTTGATGGCTAATTATCAGGAGAAGAAAAGTTGATGATGAGAGAATAAATTGACGGGAGCAAAAATTATGGAGTGAAAGCGGGAAAAATCAGTCATGACGAAGAGTTTTAAGGAATTAAAATGCATCTTTTATAACTAAAGTTAGTTGGTGTGAATGGTTCGGCACTTTCACTCCTTAAAAGAGGTTAAGGGTTTAAGTTCCGCTTTCGTATGGAGTCACCACTTTAGCCAGCACTTTACCCCTTACGGGTTGACCCGGTGCAAACGGGGATTAGTCTGGGTTGTATTACGGGCTTAAAGGTGTCTCCCACAATTGGGGCCCACTCAGGACCACCTCATGATTCAGACAAAAAAAAAAATGCATCTTTTAAGAGGTTAGTCAAAATAATTTAAAAACAAATCCTGACCATTGAATTAATAATATCATATGTCAATTGATTTTAAAAGAAATCTCACAGTGGTTAGGTCATAATAAAAAAATCAACAAAAATAAAATAATAGTAAAACTTATTTACCTAATCACTGTTAAAATATTTCTTTTCTATTTTTTCAAAAAATTATAGAAATATTTTTAAAAAATAAAAAGAAGAGAAATATTTTAGAACTTCTTAAAAAAATAGAAAAGAAGAGGATAAAGAGTTAACTTTTTTTTCCATATTTCAAAAAAAACCCTAATGAGTTCTGAGAAAGTCTCTCCATCGCCATTGGTGAGCGACAGTTTCACCAAAAAGGCAAAGTTTAGGGCTGAAGGGGATGATCGAGACAACCCTACACCCTTTTTCCTACCGTGACATAGCAA

>Cs1g02760

TGTTATACTGAAGATTTTATTTGTAAGCAGATCTAAGCCCTTTTTGAATCGTTGAAAAAATTGGAGCAACTTTTTGTTGGGTGGGTATTTTTGTTGGGGAAGAATGAAAGTGGTTTGAAGAGTTATTGATGAGTAATGGGTGAGAGTGGATTTGAGAGTGAGGATAATGGCCATTGATGAAATGGCGAAATGGAGTGAGTTTAGTGAGACAAAGAGGGAGAAGGCGTGAGGGCTCGAAATGGATGGAGGAAAAATTGAGGAAGAGAAGAATGAAAAATGGATTGAAGGATAAATTTGTAATTTAAAAATGATATTTTTATTTTTATTTTTATTTTGATCACGAGATATTTTGGAGAGGTTCCCAACTGTGGGAGACACATTTAAGTTCGTATTACAATTCAGATTAAAAGTTTCTACTTGAACCGAGAGGTCGTACCACAATTCAGACTAAAAGTTTCTGTTTGAACCGATAGAAACAAGTTCTCCCAATAAATGTGATTTTTCTACGACTTAAACTGAGAAATAAATTTAATCAAACCACTTAAGAGAATTCAATTACCAATCGAGCCAACACTTTACCAATCGAGCCAACACTTTATTGGAAAGAGTAATGATACAGTCACAAACTCTTGTATAAACTTATTTTGTACAAACTGATGTGGCATTAATTTATTGGTTGAATGAAAATATAAATTAATAAAAATAAATCATGTGGGCCAAGTGATATTTAATTTAACCAATCTTATCATGCCACATCAGTTTGTACAAAATAAATTTATACAAGAGTTTGTGGCTGTATCATTACTCTATTGGAAATGATATTTTTTTATAATGAACAAAAGTTTGGAGTCTTACGGAACTAAGTCGGGGGGAAATTTTTAAAATAATAAAATTAACACGTGTTTATTTATAGAGCGGCGACTGACTGAGACACTCGCGAGTGTCCGTAGCAATCCTCATATTTAATAGGCAGGCGCTGTAGACTAAAGAGCCTGCCCAAC

>Cs1g21840

GATTAGTCTATTTGTGGTGGGGTTGGGATTGGACGGGGAGAACAAACAAGGAAACGAAAAAAAAAAAAAAAAAAAGTAAATCACGTGTAAGCTGCAGGAAGGTCCCATGTGTACAAAAGAGGAATACAAGATATTCCATGCATATACATATGGACCTGTTCCAAGGATTGATTTGCATGTGTCCTTCACATGTAGAAATGTGCATATAAATCCTATAGCTTGTATTGTTGTCCCTCTTTTCAAATATCCTATATATGATTATCCAAAATGATCCTGGATAAGATATATAATACAAATAACTTTCTAGAACTAGAGAACGTTGAGAACTTTATATTCATTTATTAGAGATCAGATTATTGGGAAGATGGAAGCAGAACGAAATTGTTATAACATTTCATGATCAGCTAGCAAACTCATGGTTTCTAGTTTTGAATGAAGCTTAGTATGAAATTTTAAGTAACTGAAAGTAAAAAGTTTGGAGTTCAAATCTTGTTCATATAGATGAAGAAATTTAAGAAAAAAATTGGATAACTTAAAAAAATAATAATTAGAAAGCCCTAGCTTCTAGGGTCAGTGTTACAAATAATTATTAATACATGCCGTGATAGAAATGAAGATTTCATCGAATTATTCTTCCATTGTCTTGGATAATGACCGTCCATTTGTCTTTTGGAAGCATGAATGTAGAGATTTTGGCTTTCATATATGAGTCATTTGCGGGGCCCTTACTCTCATTTCTTTGTCGCCATGAATTATCATATATATTCTGTAGTGATTTCAATACCCCACCGAGACGGAAAACTTTAGTTATAAGGTTTAAAACATTAGAGAAATGGGCATAAAGATGATCTGTTCATGATATAATAGATGATGGCCCGTGAGTAGAGTGGGCCAAAAAGCTCAACTGCCTTCAAGGTTCAATCCCCACTACTCAATACATGACAAAATTCCAACGCCCACATATAGACAACTCAATGGCCGGCTATTTCACATGCAACCAA

>Cs1g06240

ACAAACTAGAGTTGAATAAATGAAATTTACATGTGATTTGAATTATTCGAGTTGAATTAAATTGTGATACTCATGGATAACAAATTCAAATTGAATAAGAAAAGTGAATGATTAATTCAAATTGAATGAGAAAAAGGTCTATATAAGAGAGACCTATAAAATCTTTAGTAATATCTATTACGACAATAAAAAGAGAAGAGAACAATTCGATTATTTTAATAAATGCTCTATGCTTTTGTCTTGTATTTTCCTTCATCGATTTTTCTTTTTGTTGTTTAAACCGTCAAATTTGGAGTGCACTTTTTATAGTAATCGTCGTTGAATCTTGAAGACCAGATCGAAAAATCTTCCTTTGAGGCGTGAAAATTTATCTTAAGGATACGACTTGACAAATATATAGTTAATGTGTATTGGGTAGGATAAGCAATAACAATGAACAGTGATGTAAACATTACCAACACTAACTATCCATGAAATTTTATTCGCCACGTTCTATGTTTGTCTCGGTCTATTATTAATCAATGTTTATTGATTAATGTTGTTGTTTTGGTTGGTTTAGGAGTTTAAAGAAACAATATTGCCTTATAAATATGAGTCCTCGTCTTTATTCATCTTTCATCGATCCTCATATTCAGAAAGTTAATTTTTATTTGACAAATCACGGAATCCGTGTAAATATATTGAAGTTGGCTTATGCAAGTGGATTTTAGTGGAGGCTCAACTCGATTCACCAAGGACCCTAAAGAGAGCAAAACCAAGCCCAGCGAGAGCTGGACACTAGAGCAGACATGAGGGCCCTTTTGTAATTTTGTCTCAAAGTTTTTTATTGGGCTAGACCGATTCGGCCCAAACTTTTGATTTTTCTTTTTAAACTATTTCGGCCCTGCTCGCATATTCTTTGACGCGTAAAAGAGAAAAAATAACACATGGACCACTGCTTGCCAACACAAGGAAGCAAAAAGCCTCACGATTTTCAACAAATTAAGTAAATCAGAAGAAAC

>Cs1g09680

GATATGGATATGGGTATTTTAAAAAAATATCCAAATAAAAATTGGATGGGTATGGATATCCAATTAATATCTTATTTGAAATTAAAAAATTAAAATCGTAAAACTAAAATGAAATAAGCAAAATCAAATGTCTGGATAGCAAAACCTAAGATTATCACATCGCTCGCTGGCCGGTCTTCGCTTACCGACGCTCTTCCCCGACTCCGACGCTGCTTTCCGGGGGTTCCGTTTAGGTGCGCCGGCGCTGTACCAATGGTTGATCAGCCGTAACTGTAGCTTCCAGCTATTCTTGAGCGCTTTTCTGCCTCGTTCCAGCTGCCATCGTCTCTGTCGTCGCTACCACCGCCGTCGTCCGTCCGCCTGTTTGTTGTCTCCCTCGCAAGGTAAGTTTTGATTGCTGAAGCTTATAAGCTGTTTAGTAGAGTGGGAGTTGGCAAGAGTAGTATGTGAGCGTTTGAAACCCTTCTATACTATGACTGAGATGTTTTCTGGCACTAAATACCCAACAGCTAACTTATTTTTTCCAATAATTTGTGAAATTAGATTATCATTGAATGCTTGGCTTAATTCTTCATGTGATGTGATAAAAAAATATGGCGAAAAGTATGTTAGAAAAGTTTGGAAAATATTGGGATGAAATCCATGGTGTAATAGCTGTTGCTGTTGTATTGGATCCTAGATATAAAATGGTTCTAGTGGATTATTTCTTTCCTCAGATTTATGGTAGTGATGCATCAACTCATGTTGATAGAATCCGTACTCTCTGTTCTGACTTATATTCAGAATATAAAAAAAAGAGTGTGGTTGGATCGAATTTGGCTGAAGGATTTGGTGAGTCTAGTGTTGTTTGTAATTCTAATTCTAGTTCAGTTGTGATGGGTATGTGGGATGTGCAAAAATTTAATGCATTCAAAGCCAACATATTGGTAAACGAGTTAAGTCGGAGTTAGATAATTACTTGGAAGAAGAGGATAAGACTACTCCAGACTTCGATATTTTAA

>Cs1g20110

CCCCGCGGGGTACTTATTGCACTCCCGCTAATTAAACGAAAATATATGTATAAGAGACCTGTTGGTGGTAAAATATGGAGAAAGAAGTATTAAGCGCATGACATGTGGGGAATATGATTCATGACTTTTGAAGCCCTCCAAAGGATGAAGGGGAAGTTGAATAGGCCTCAGTAGAATGGGAAGATCTCGACAAATTAGTATCAACCACCTAAATTATCTAAAAATAATAAAAAGTATTTATTACAATATATCTAAGTTGCCACGTGTCAATATGGGACCACATAACAGAGACAATCTCACCAAGAGCAGGGCATGTCATTACAGCACAGTCCCCACCTGAATTATCTCTAAAATATCTCCTTACAAACCACTAAAATCATATTTCATAAAGAGGACCACATATACAACTACATGCATAATCTCTAATGAAATTAGGTGCTCCTCAGCCCAATTGATTCATTTCAAATGAGCCAATTAGAATTAAAGATATATTACCAACAAAAACTATCCCCCTAGAGGACAGCTCTCTAACCTAAGCAAGCTCAAAAGCCTCAACAAAAAGGCCAAATTACTCTTAAATCCCTAAGGAAGAAAATAACTAATGGTGGTTTGCCATGCTATATAAAAGGACATAAAGAGGTCCTCGGGGGGGCATTCCATCTTCAAAAGTCAGGTTGATTTCACAAAAATATTAAGTTTACTCATTCACAAAAATACTCAACTTTTCTCCTATTCTAGCGTTTCTTGGAAAACCTCCCCTCTTTCAACCTCCCACTGACTTGACCGTCAGAGTTTCATCATCGGCCACCAACGTCATTGCCTTTAATGGCTATTCCTTATGATTTCAGGTTCATCTTTGTCGCAATGTTCCCCAAAAAGACTGTTTCTAGGCACCTCCATCTCCATTACTGTCAGAAATCACCTTCTGTAGAGAATCTCTCATAATGCTTTGTGCTAACTAGAATTGGACCAAAACAAGACCCAAAAAAAGACGAAGAAGA

>Cs1g05380

TTTAGTAATTTATTTCTCTTAGCAGTTTAACAGTAAAATTTACCAAATGTTCATAATTGTTTTTAAAACTCACAGTACTTCTGAAAATAAAATTTACCAAATATTCAACTGATTCTCTTCACAGTTGATTATTTATACAGTACAGCTAACAACAATTATTTTAAAAGGTACAACATTCCCAAACTGGCCCTAAGATCGATGTTTAAGTTTTTGTATCTTTGAATATGTGTTGTGGCAATATATTTTCTTCTTTATAATGTGTATATTTTCAAATTTACCAGCTTCTACTTGGATGCATCATAATAGTGAGATTCAGAGAGATTTATAATGCATCGATGAGTTATATAGCCCAATACTTTTGCATGAACCAATGATATTCTCGCAACTATATTAGAATCATTTGCTTATTCTATAATTGGAGAAATTGTAGAATACATCAGAGGTAACATGTCAGTCATTCAACAAACAAATGATGCTATGACATATATACATTTATTTTAAAAAATCATCATACAAGTGATGATTGTATTAAATTCAATTATACATGTCATGCATGCATCAATTGGTTGTCGTAAATTAGAAGTACTCTATAATTTCTCCTATAACTGATATGGTACTTTAGACGTATTATAAAATTTCTCAGCAACTTGATTGCCGTCAATAGATGCATTTGCTATTGGCTTCGTTCTACCCCACGCAAACATCTATCAATGAAGAAAAATCTACATTTTTGTGCTTGAACAACCCTCGATAGGAAATTGAAACAATCATCCACACGTTTTGAGAGAAGAGCCTCAAACGTCAAACATGCTAAATTTCATTTCCATTACCTACCTTGCCACTTCCGTTTGGTGCTTCGTCTTGTTTTTGCTTGATTCTCATAGTTGTTTTGGCCTTCATTCAAACGAGACAGACCGCCTCGCTTATCTAGCCATAAAGTCACAGCTTCAGGATCCACTTGGGGTTACAAAGTCATGGAATAATTCTATAAGCTTGTGCCA

>Cs1g06770

TGATTTAGGTCTCGACTCGATCTGAGCCATCCTCACTTAAAAAGAAAAGTTTTGGGATGCTCCTATAGCAATATTCTTTTTCCATTCACTTGCTTACCTGAAACTTTATCACCTTAGGGCAACTCTTAGTTCTACCTTTACACTTTTTAGTTCTACTTTAAATTGCTCCATTGCTTTCTTTACATCTTCGCCAACATTAATATGTGTAAAAAAAAAAAAATACATACTATAAAAAAAAAACTATGAAAAAGTTTCACTACATGACAAAATTTCTTTCATTTTTGTGCCTTTTGCATATTTTCAATATATGTAACATTATTCCATCTTCACAGGACATTTTGTTCAATCTCTTCTTTGGAAAACTGTCGACTTCCCATCTGTCCAACCATTTCTCCAACCAGTGTGTAATAAAATTGACCTAATATTTGCCAAGATGCTCTCACAATCCTATATTTAGTTGCATTTATTGTACTCCTTGATCAGATAAATCGTAAATTTATTATGATAGATTTCATCATAGCATGTTTAACATACAATTTCATAATTGAAAGTAAGAAAAATGTTGAGAGTGATTGGATGTTGTAAAGACAGAAACAGACCTTTATCTCTTTTATCTGATCAGCGCTCAACTTTGCGTCACTCTCGAAGAGACAAATATAGTAGCCCAGTAACACACCTCTATTAGGAATTTTAGTATTAAAGATAATATCACTAATTAATGCATGTAATTAAATTTAATATAATTATTATTTATATAATATTTTTAAAAATATATAATAATAATAATATCATTTATTTATCGTATGACAGACATGATACTTTTTAATGTATTGTAGAATTCTCCTCCATTATTTAGCCTTTTTCCTGCTTTTCTTTTACGAGTGGAGAACGCTAAATGACTAGCTTTCCTCACAAAAGTTATAAAACAACAAAACCATTAAAGTGGCTTTGGAACTGGTACATTCAATAGCACTACAGAGAAAATTCTTCTGCTGTTTAAC

>Cs1g07500

CATTGTCTTTAATAAAATCTCATTTCCAACCCGAGAGAGCATCCTCGTACTCCAACTCTGAAGCCTTTGCCAGAGCCTGTCCCGAATATGCCTGAAAACTTCTTTCTTTTTTCTGCCAATATATGATGGAAGACCAAGGTAGGCTCCATGATTAGTGGTCGTGTTAACCGCAATAACATCACATACCTGCCGAGTTACAGAGTCAAGCACATTAGCATTAAATGAAATTGAAGATTTATTGAAATTAACCTCTTGACTAGAGGCTCGACCATATACTGCCAGTAACTACTTGATTAAGGAAGGTTCATTGTTATTTGCCCTAAAGAAAAGGAAGCTATCATCGGCAAAGAACAAGTGAGACACCGATGGAGCACCTCTCACAATTCTAACACCATGAAGCAAGCCATTCCTTTCTTGCCTTCGAATTAAAGAGCTTAATCCTTCTGCACAAATAATGAATAAATACAGAGATAATGGGTCTCCTTGACGAAGAACCCTACAAGGAACAATCGGACCAACTTCCTTCCCCTCCCGTAGCACACTATATTAGATAGTTGATACACGAAGCATAATTAACTCCACCCACTTGGCATCAAACCCCAACTTAAGCATCATAGCTTGAAGAAAATTTCACTCAATCTTGTCATACGCCTTAGACATATCAATTTTCAGAGCAGCTACACCATTTTTTCTTTGCCGCTTTCACTTTAAGAGGTGCATAATTTCAGCCGAAAATGATTATATTATCAGTGATAGCTCGACCTGGGACAAAAGCACTTTGAGAATCTAAAATAACCAAATTAAGAATTGATTTTATGCGGTTAGCTAACATTTTGGATATGATCTTATGCAGAACATTGCATAAAGCAATCAAGAAATGATTCCACTCAACTGGATATTCTTTTTCGTAGGCATTTTTATCTTGTTAATTTCACCTTCTTGAGGTATATGCCCATATAAATTACAAAAAGTCCAGTTTACCCTTAAAGACACGTCAAGGA

>Cs1g09770

GGCTAACCAGTTAGAGCGTAAGCAATTTTTCCTCTCTGGATTTTATCGATTAACTGTTTCAAAGAAAACGATAGACCGTTTATTTCCAGAATCGCTTAGCTAGCCAAATATTGCAGGGAATCATTTTTCAATTTGAGTTTAATAATTATCCATTTTTCATGAATATAATTGGAATGATTATAAGGCTTATGTTTATTAATGAATAGCACAACATTTTAATCAAAGTCATTTAAAGTTTCTTATCATCAAAATCAATATTATGAACATATACAAGTTAACATTCTCCCCCTTTTTAATGATGACAAACTTCTTTCAATATTTTGCTCCCCCTTAATATATGCTCCCCCTAAATGTATGGCCAATTTAAAAATTGTTGAATAAACAGATTTTTACTTAATAAAGTGTTTAAGGAGATTTATTTCAAATTTAAGTTTTATACTTATTATTCTCTACCTCCCCCTTCTTTATTTTATATTCAGTATACATTATCATTATTCTCTCCCCCTTCATCATAAAAAAGAAAAAAAAAACCTAAAACATAGATTTAAGGAAGAAATTACCCTGATTAGGCGAGCAAATTTTTTTGGCAAAGTTCCCCTTAAAATGGAACAAATCCTCAAATACAAAACAAGATTAAAACAATTCCAAATATAAGAAAAAACAAATTTCTATATATCAACAACTCCATCAAGCATCAGTCCACACACATAGAATCATCCAATATCAAAAGCATCACATATAAAGAATTCATCATCAAAATCAAATAAAAGTAGAATGTATGCCAAACAAATTAATAACAACACACAAATAAACATAATATGAAGCGTATGTGTGAAAAGATGAAGAATGAAACTAAGATGGAGGTGGTGAAGATGAACTGCCAGGGTCATAGCCAAAATAACGAAGGAGTGTCTGTTGTCCAGCAATAAGCTCAAGCCGCTGATCAATGCTCTGCTACTGAAAAGTCTGAAACTGAGAGCGTAGCTACTACTAATCTCGAG

>Cs1g09140

ATTTTAACAATTGTCATTTTATGAGCGTAAAATCCAAAATTAAGTGCTTTTATACCTCAGGCCTCAGCAATGGCATGCCATAATACTGTTTGAGTAATAAAAGAGCAAATTTAATATCAAGTTGATAAGAAATTTAATTTGTGGTTTCGTTTCCATGCGCGGGTGAATAAAACTGGATAAGCTCTTAGGATGCAATAATAACAAATAATATGAATAATGCGCTTTTTGAGATAAATACTGTAAGTAAATTGATGAATGAAATCAGTGTTTGGACGACTAGGCCTAATAACTCTTCAATCTTTATATTTTAAAAAATATCTCAACACAGTCTTAGCGTTAAATATGTTACAACTTTATTATTACTTTTTTCTTTCTTTTTACAATAACATCATTGCAATAATCTTCCTACCTTCTTGAGAATATTAAAAAAAAAGAAAATAATATTAATTTATCAAATTAGCATTAAAATAACATAAAAATTTATGGAAATTTTTATGTTATTATTTTACTTGGAGGATTAATTTTGGTAAAATTATTTTTTATATAATAACTATTAGTAAAATTGTTTCAAGGATATTAAACATTACTTTAAGTTAGTACATAAATTATTAGCAAGATTGTCATACGAGTATAAAAAATAGTAGTTCTCTCATATTGATATTTATTGGTTAATTTGCTAACTAAAATTTTTGTAGATAAATTAATTACGGCCAAGAATATTATTGTAAAAGAAAAGAAAAAGAAAAGATAAGAACGTAACTTATTTAATTATAAAGATATTTTGAGGTATTTTTTAAAATATAAAGATTAAATAGAGTGAAAATATAGAGAATAAATACAAATTGTTGAATGAAATTTTATGTTGTTGAATGAAAATTTTTCTTGAACCACGTATAAGTTAATCATGTTGTGCCAAGGGACTTGTATAAAAATAATTTACAGAAGAATTTGTAGGTAGTATTACACTAAACATATAACAAAAATGTTGAGGTGAGATTACT

>Cs1g05440

AGATATACAAGCCATTTTAAAATAAAATTGTAAAAAATAATTAAATAATATTTGATTTTTTGCATAAATTTAAAATTTAAGAATAACTTAATAAATAAATAATAATAACACTGAATAATATATATAATATATAACTAGGACAAAAAGAAACATAGATTTCAGAGGCATTAGATTTTTTTTTTTTATCTTTTAGCTTGTTTGGTCAAGATAAATAATTAAATAAATAATCAATCAGTACCATTATTTTATCAAAAGTAAACACAGCCTCTTTCATTTACTTAAAAAGAAACACAGCAAAGATATAAGAATACAAATTTAATCTATTACAGAATAAAAAACATTGAATCAAAGCGTGCGTCTTTTTATCTTTTAACTTGTTCCTGATTTTAGTATAGTCAATGATTCTATACCATTTTGAATCTATTTAATCCAAGATCGTTCGATATTTAAAGTGATGGGAGTCTTTAAAGTTAAAATTGTTATGAAAAAAATAATTGTAAAATAAAAGTTAATTGTGTTTGGTAAATTTAAGTACCATAAAATATGATCACTTACACCACATCGTATGTAGTAAGGTTGGCAAAATCAGATTTAGATATGAATGTAAGTGCATTCGGGTCCGGATATGCATTTACATTTCTATATCCATNNNNNNNNNNNNNNNNNNNNNNNNNNNNNNNNNNNNNNNNNNNNNNNNNNNNNNNNNNNNNNNNNNNNNNNNNNNNNNNNNNNNNNNNNNNNNNNNNNNNNNNNNNNNNNNNNNNNNNNNNATTCGAATGCGGGTAATATCCACAACGGATGCGGGCGGATATTCCGGATCTGTAAAAATAAAAATCAAATAAATTAAAAATTCAACAATTCAAATAATCATATCAAATAAAATTCAATTATAATCCAACAATTAACAATTAAACATAAATAACATACAGCAACATCATAAATCCACAAATAAATATATCATAACATAAATTTGAAAATCTGAAATTGATTTGTTGGAAACT

>Cs1g02110

GAAATATTTGCACTATGATTGTCAACCTTAAACAGCTCATTGTGATCTCAAACCCAACAATGTTCTTCTTGATGCTGACTTGACTGCACATTTAGGTGACTTCGGCTTAGCAAGATTCCTTCCATCCACTCATAAGAAAACGAGCACTATTGGTATCAAAGGATCTATTGGCTATATTGCTCCAGGTATTAGCTTTTATATTTATCTTTTTTGACACGTAAATTTTTATGATACTTAATATAAATTGAATGAATTGATTGTTTGGATGGCATCAGAGTATGGCTTAGGGAGTGAAGTGTCAGCATATGGAGATGTTTACAGCTATGGGATACTACTGTTAGAAATGGTGACAAGTAAAAGACCCACAAATGTTATGTTTGAGGGAGACTTGAATCTTCATAACTTTGCGAAAACAGCCTTGCCTGATCATGTGATAGATATTGTGGATGCAGTAATTCTAAACGATGACGAAGAATTGACTGCTACTAACCAAAATCAAAGACAAGCAAGAATCAACAACAGAATTGAATGTCTCAAATCTATGGTTGGAATTGGAGTTGCATGTTCAATGGAGTTGCCACAAGATCGAATGAACATAACAAATGTTGTCCACGAATTGCAATCAGTCAAAAAAATTCTTCTTGGACATTGAACTATATATGTTCAATAGGCAAAGAGGTAATTGATAGTTCTTTGTCTAATCATATGGAACAAATTCAATTGTACATTTTGGCTTTTGTAATAAAGAAGGTTTGGTGATCACACTGAACAGTGCCGAAAAATCGTGGTGGTGAGAATATTTCGTGGATTTTTTTTTCAAATAATATAAACTATGTTCAAGGGGCATGTGAAGAGACTAGCATCATCGCCACTATAACCACTCCCAAATGAAAGCAAAAGCTTTATAATATTTTAAGAGAAAGAAAAACATTAGCACATCAACTTTATCAATATTTTATAATTTATGCTGCGATAAATAAGGAAAAGAAAAGAACAAATTA

>Cs1g03810

AACTACAAAATGACATAGGCTTTATTCTCGAGTGGGGCAAATCACATGCATGTAGCCGTATGTTCACCATCTTCGAATTGGGGATATAGTTATTATTTTTGTATTCCCTCCAAGTGTTTGATAAAAAGCCTGAATCAATCTTGGCGCTTTAATTATTGCAGGCCATTTCTACCTATGAGGAGCCAAGGAATATGAAGCTTATTATATTCATTCGGAAAGCAAAGCTTAATGAGGTGTTGTATTTAATCTTGCTTATGCTTAGTCTAGGTGTACAATTCATCTGTCACATATTGTTCAATTTATGTTGACATAATGTTGCTTTGCTTTTTGAAGGACTGGGTTGTGTAAACTTACAGGAGATTCAAGGATTGTGAGAATCTAAATCTATATTTTATATTTTATATGAATTTGGCTTATTGAAATTATATTCCAATTGAATAATCAATGAATAATGAAATTTACTTTTGTGATTTTAAAAGATAGATGGAGTAAAATTATGTCAAAGGCTTACACATATTTCTTCCAAGATACAATTTGGAACTTTGTGCTTTGAAGATGAATTGTAAACTTTATTGTAATTTCAAGAATTCAACAGGCTTCATTGCTTTTTTATCTACAAATGATGCACTTGAGCACTAGTTACAGAATAGTAATTAGCAGTATATCTTGATCTTTGGCAGTATCTACAAGGCTGATGCTAGTGAAGCTCCAATAATATTACCGCCTTAAAATATTACCATTTTCACTTTTGATTTTTTCAGGATCTGGGCTCTGTGCAGGAGAGTATGTTACTCTAGGATGGAATAAAGTAAGTGGTTGGTTAACTAGTGGGAAAAAATTACACATCTGGGAATTTTTTTTTAAATTTTAATTTTTAAAGGGAAAAAATCATTTATGAGATCAATTATCTATCTTGTTGACCTTTATTCTTGTAATTAAGGTCCATCTGAAGTTGATTGCTTGTTTTTCAGAAAGATGATCTGAAGGCTGTGATTGATTAC

>Cs1g11750

TAAGATTAAGAGCAAATATTGTTAGGTGTATTTAGTAATATGATTTCCCAAAATTCACACCTTTCTTGGATCAACAAGTTTGTGGGCCGAACCCAACCATTGTAAACCTTTCATAGTTTTTATTTTTATTTTTTTTAAAGTAAACCTTACGTTGGATTTGTAATATACATTTGTTTTAGTAAAAATTTTATTAAAAGAAAGCACCCTTATACACAGACACAGAATACTTGAGTCATCCACTTTAAGAACTAAGAGATGAGGTGTGCACCACTTAATTAAAGAACAATTTGTAATTTATTAAACGTATATAACCCTTATTTATATTATATGAAAGTAAATGCATTTAACAATAAGAATGACCTTAAGACCTCTCTATCTATTTTAATGTTAGATGGTGGGTGTACACTAAAAAAATTAGTTAGAATAAAATATTATTATTTTTTTGGAAAATTATCAATCCTTTATCCTAAGAATATCCCGTTATAAAAAATACTATAAAAACTTGAATTTATTCGATTTTACATTCATTATTTATATTTTGTATTACTTGTCCACTCTACCGTTGGGAATTTATAGTCATTCACTAGATTAAGACTTTTTTAAACTATAGATATATCGTTTGCCACCATATATGGTTGATAACGGGATAATTTCAAAGTTGACGCAAATAATTTCTTAAAAAAAGGGGAAATTATTAATCTTGTTTTTTTTTCTAAATATTGACAACAAATATAGATTAAAAGAATGTTGGAAAAGAATATATTCTAATAAATAATGGAGAAATCTACTTACATTTCTATCATTAGTGAAGAGGCCACTCTCACATGAAAGATGGGAGAATAAAAAGAGGAGAGTAATTGTTAACTATCTAAATATTTAATTAGTGTAAATTATTGATTAAGGACTTTTTGTTCACATAATGTTGTTTGTTTTGGTTTGCTTAAACTGTTATAATTATTGAAAAATTAATTAGTTATTCAAGAAATCTTAATCCGGTGTAT

>Cs1g07210

AGTCTCCCTTAAAAATTAGAACCAATTCCATACTTCAGATACCGCCACGTGTCCACTAAAATCCTAAATCCTTCCTATATGAAACCCGTCACAATTGGCTCCCAAAAAAATTTACAGAGAACAAAAACGACTAAAACTTCCAATCAGTCCACATGGTCAAGTGTCAACTGGGCAACCAGAATATTCCGGAAAAACGGCATGTCGTTTCCGCAGCTTATCCGCAGCCGACTTGTCGTGCATCCACGTAATGAATAAATCCTATTGATAAATTGCTCTTTATGTTTGTGTGTGCTTAAATTTAAGTAACCAAACTCTTCAAAATATTTCCGCATAGCAAAAATCATTTTTTGTTTAAATTTCACTCATATATTTCAATTACCACCCAATACAAGTCAAACAATAAGATATTCTAAAAATTTTGACTAATAAAATTAAGGGTGCGTTTAAGAACAAAGTATTCTAACTTTTAAAATATAGATGTTGGAGAGTAAAATGCATGATTAAGAAGTAGAATTGAAGAAAAAAAAAACTATAAAACTTCCAAAATCCCATTATAAGGTGTTACTAAACACCTTGGTGTATAAGCTTTGGCAGCTCAACTATTACACATGACTTCCAAATGCACCTTAATAGCACTTAACATTTAAAAAAAAAAGATATGGAATTAATCATAAGTCTTAAAATTAATTGCCATGTTTTCTTTTAAGGATACATTTGTTGCCACTTCACTATGATTAATAAACTACAGTTATTTGAATTAGAATCTTCTCTTAAATCGAATTTTCCATTTTAAAAATTCTATATCATGTATATTCTTGTGATAATATATAAATAAAATTTAGTTTTTTTTTTATTAACCCTTTGAGCCTTAACAAACACTGTTGACAACGCCCGTAACTCACGTGGCACATACTTACGGAGTCTCTAGAACTAACTCCACATTTCTGAAGCAGCCACGTGTCCTCTAAAACCCTAAACCCTTCCTATATCACAACCCCCCC

>Cs1g04750

TTTTTAAGAACGCATGTCAAACTATTAGCATATTTTTTTTCAAGCCTATATATATATATATATATATAGATTTATATGCCTTTATTCAAATTTAATAGAATAAATTTTTTTCAATAATATTATAAATTTTTTAATACTTAATAACAATAATAATAATAATAATTTCTTAAGGGTTAACTTTATTGTTAACTTGGATTTATATAGAGTCATAAATCATGGTTCACAATAACATTAATGCATATTTATAAAATCATGATATTTATAATTTTATTCATTAAAATCATGATATTAATCATTTGTTTAATAAATTATAAATATAAATAAATTATAGAACTTTTTTTAAACTAATATTTAAATGAATTTAAAAAATTCAATTTCAAGTTATTTGTAAAATCATAAATTTTAAATTTACTTTTATTAAAAATAAAATGTGCTTATTGTAAATTTTTTTTATCGTACCGTGCTTAACCCATCTCTAATCGTATCGTTTTTTTAAGGCTCGCGTGCTTAAAATTTTAAGTGCAGCTCAACCCACGTGTGTGCTGTGCCGTGCCACTTACCCCACCAAATTATGCTCGTGTTATGCCATGCTCTACGAACACATGGCGTCTGACCAATTGGCCATATCTAAATATGACAAGGTTTACCTCGAGTCTCCCTCCCTTAAGATTTGGGAAACCTCGACCACATATACAGAGACCTATCCTCTTATGCTTTTAATTTTAAAAAGAAAAAAAAAACGCACACAGCCAAAATTTTAACATAAAATATGAAAAAGAACAAAATTTTTTAATGAGAAAATAACAGAAATCATGCTTAATGAAGAAAAAAAATAATTATGAATTAAACTATTCCAACCAAACAAATAGGACAAGGCAGATACGTATATAATAATGCAGGCATAATTAAGTTAACAAGACAGATAGAGGAATCAATATCACAGATCCTGAGCTTTTCAGTATGAGCTTACAACAGAAGCAAAACATTTTTTACATGTTATT

>Cs1g22960

ATCAAGTTTGTTCAGCTGCAACTGGATACACATTTCTTGTTTAATAACGAACCTCTTAACCAGTTGCACAAGTTTGATCACTGAACCAAGCAGTGCTTATACACGCAGCAATCCAACTTGAGGATGCGACAACTTAAGTGACAGATTGGAATGTGCATGCCAGCGAATTGTAATCACAGAACATATCGACACATTTACATTAATGCACGTAAATAATACATGTTCAGATGCAAGTAAAAATCCTCTTTTGAGTTGACTGTTGAGCCAAAGTCATTATTGGTCTATAATAATTAATGACGCTAGTAAGTCAACGCTGTTATAAAAATATATATAAAAGAAGAAAGTGAAAACTGAACTGAATTAATTTGCTGTGTTGATTGCCTCTTTAATCGTCAATATATCCATCACTTTTCTTGCAGGGCACTGGCTTATTGTATCGAAGTCTTCGAAGGATCAATTTCACAAGGCACTTTCGGCATATAAAGAAATCATCGCTGCCTAGAACTTACCTCAATCTTTGAACTATCTGACAAGAAATGGGCGAGGGAAACTTCTCTCAAGTGCAGTTTTTCTAAGATTTAAGTCAAACTTGAGAGTATAATTACATTGTCCATTGTTGTTAATTTCTCAAGAGGAATACATCATCAAGCCACTCAAAGCTATTTTAATTTTTCATCAAGTGGGGTTAGCTGAATGCAAAAACAAAAACCCAACAAATTAACAATAGATTCAAGCTTCCCATTACATTACAAAACAATTTTGCATACCAATTTCTTAGAAATGGAAACGCTAAAACCCAAATCAAATCTAAAAAAAATGGAATTGTACCGAACTCACTGAGAGTGACATCTTTAGTGGCTCTATCACTCTCCAATTTTAGAAAAAAGAAAAAAAAAAGAAATTAATCAAAGTTAGTAGGTAGACTATCTTCATTTCAGTCTATAAATTGCCTTAAAACACAAGTGAAGTGGTGAGCAAGCTAACCTATTTGTTTTAAAGAT

>Cs1g13070

TGCACATTATATTTTCTTGACTAATGCTTAACATTTGATTCTAAACCACAAAATAATTGTGTGAAGTAGTCCACATTTCACCCATTCTTAGCCCTTATCATCAGAATTGCTCTCCTTAGGCCCCATACCTCAGGTTCAAATCATGGGCAATTGAGCGACAAACATGATAACACAATCTAAACTTTTAGTGGCTTGAAGGAACATGGGATGTTTCAAAATGAACAGTAATCTCCACATCTCCGTTTAAAAAGTTTTTCTACATCTCCAATTGTATTTCACACAAAAGAGTGATTTTCACCGCATATGACTTAATGCAATGTAAATGTCATTGATATAATTCAATAGTTTTCATGTCAAAATAAGTGTTAGCAAATGCAAAGACATTTAAAATCACTAATTGCATTAAGAATATTTTGAAAAAAAAAAATCTGTGAAGGTCTAGAAGTTCAGATCAATTTACATAACATGAGAATTCATACTCTAGAGATCTAGATTTCTAGAAATCTGAAAAATGTTCCGAAAGCAATCGAGCACCAAACTCAATTATTAGGAATCAACATCATTAATCTCATCTAAACATGACATGTTACATGTTACATGTACATAAAAGCATATTTCATCATATTTTACCTCAAAGTCTTACTAAATCAAATACACCCAACATTTTTTTAATCCATATAAACATATAAAAAGACCTAAACTCATTTATTGAAGACAACAAAATTTAATTTTCAAAAATTATTAGCCTGGAGAACGGAGATGATAATTTGAAAATGGAAAGGCTGGGCTTTTTCTTTGGTTCGGGCCATAACCAGCCTTGGCCGACACCGGACCCGGCCCGACCCATTCCCATCCCATCACACATTAATGGAACAAATGTCCAAGTGTTTTATGAACTTTGTTGAGGTAGTGCTCTTGTCATATCATTTGTAGTGTAGGCTCGGCCTGATTGGAACGACTACCAAACGAGTACCAGCTGGAAGGCTGATAGGAAGGAAATT

>Cs1g10840

TTATTTATTTCATTATATGTTATTGCTCTTGTATTAACTTATTAATCTCAATGATGTACTTATTTATTTTAATCATTTTCAAGATTGTAGGAGATGGCTGAAGATGTTAAGTTCACTCTTCCGGAGCTTGAAGGAAGACCAAAGAGAAGAAAATAAACAAAAAAGAAAAGCTTGCAAAAAGAAGAAGTTAGTCATGAAGCTTTAATTGAGTATAATTCTTATGGTGTTCCAGTTGGAAAGGGAAGAAATGATCTTAGGAGTTACATTGGAGTCATTATACGTGAAACAATTTCAATTTTACTTGATGATTGGAGGCGTGTGCCACTAGAGATAAAGGAAACTTTGTGGCTTCATTTTCAGGTTTTGTTTACTTGTTTAATAGTATGTTTAAAAATTTTATATAACTTCTAGATTTGTTGAAAGCTGTTTCTAATCCATGTTATTAGTTTACAGAAAAAATTCAAATTGAGCTTGAAATGCAAAAGCCAAGTGTTAAAGTGGATGAAGATTGCATCAAGAAACTTTCGAAGTGAACTGGCAGCTGAATTCGTTCTACCTAACAAGGACAATCGAAAGTCACTAAGGTTGCCTCCTATTGAATATCCGAGCATTAAAAAGGAATATTGGAAACTTTTGTTTGACAAAGTTTATTTTGAACAATTTTAGGTGTGTTGTTTGTTAATATTACCTGATTTATTTGTTGCTAGTTACTAATAGTCAGTGGTGACCTTTTGTTCTTCGTCATACTGTTTGTGCAGGAAAAAAGTAAGAAGGCAAAAGTCAAAAGAGCAAAGAATGTCTACAACCATCGCTTGGGTAGCACAAGATATGGTGGCATGTTGTATAGAAAAGTATGTGTAGTAAATAATCTAATATGTGTAGTAAGTAATCTAATACATATAATATAATAGAATCAATTATTTATGTTTTATGTCACCTTGTAACAGAAAAATAAGAGTGGAGTTTCTGAGCGGGAGATTGACCGCAGTGAAGCTTGGTTA

>Cs1g17680

CATGCTTTGTTGAACTTTAAACTGGTGCAATAACTTGAATAATCTTCTCGTGTCCTGAAAATCAATGTGTTAAATGGTTTCTTTCTGTGGTAATGTAGATGGGTGCCCGGAAACATGCATCTTCAGTGCCTATTCCCATGCCGGTACTGCTTCGAGTGAGAGAGCTTGCGGAAATGCAGTCACAGTTTCCACCAAATCTGAGCTCAAGCAAGCTTACTGGATCTGCTGGAAGAAATGTAAATGAAACAAGATCGGGGTACTTGTAACACTGTAAATAAAATAATACATACCCAGTTGCCTAGAGTACTAAAATAAAGTTTGCTAATTGCTAGTAGCTTTGTTGGTTGAACATTAGTTATTCTGACCTAAATTTAGCCCTTTAAAAAATTATGGTCTTTTAGATTTAATTTTATGGTGCCGCAATCCCCCTGGGATTAAGTAGCTGGCTGCTTAAGTGATATCTTTACCGAAGATCTTTGTTAACCTAGATTTGTATTATTGGAACGAGAATTGTACAATTCGTCTTGTTGCTAGTCTCCTGTTGATGTTCCTTTTTTTCTTTTCCTTTTAGTAGTCTGGAGTCTCTCGGTTTCTAGTAGTCTAGAAAGGTTTTTTATATCGTAATTTGTATTTGCGAAGGGTGTGAAAGGACGTTCAAATGGAGTAGAGGCATACGTACTCCGTAACGTCAATAATCTAATTGTTGGACTCGAAGCAGATCAGAAATTTGTCGTTTTTCAATTAAATATGAAGAAAATAAAACAAATTGATTGTGCGATAATTAATTCACTACTCGAATCCCCTGTAATTTAAATTTTCTTATTTCTTTAAAATGGAAAAATGAAAAAACTGTATATGAGTCTGAGCTGGGCTTGAAATATTTAGAATCTGGGCCCCTAAAACCCAATAAATACGGCCTGTAAAATTAGCCCAATCAAAAGGGCTTACAAAATTAGCCCAAAGGCCCTCTTTTTCAGCCCTTTTCATGTCACCCGCTTCGC

>Cs1g07600

TTAAAATCAAATTTTTTAAAATTGTTTATTTATTTTCAATTAAATTAAAGGAAAGGAGACTCCAATCTCCATAGTGACATTTTTTTATAAACAAAAAAAGATATTTCTCCCTTTTTTTTCTAATTAACAAGGAACGTTCAACCTTATTTTCATTCCGAATAAGATAAATTTTATATATATTTAATAATTTATTTAAAAGAAAACGTTATAAAACTGTTTATTATTATTTTTTTTCAAAAAAGATGAAGAATAAAAAAAGGTGGGTTTGGTGAGTTCACATGAACTAATAATACATTCTCTTAATCTACGCCATTTGGCATTTGCCCGTGACGTCCGCCTACCGCCGCTCACTCCCACCGGTTATAGCCGGTCATCCCTTGGCCCTTTCATGTTCATCGTTTTTCGCAATTTCACTTGCTATCCAAGCGCGTGCTTCCACCGCCAGCCACGATATACTACTATATTTTCTGCTGCTCCTTGCATTTGGTGTCTCCATGTAAGCATGAAGGTTACACACATTTTGTTAATGGTTTTTCATAAATTGAATTGAGAGATTTAATAGCATGAGACATTTATACAATTTTTAGTCTTTTTTTTTTAGGATTTCACATATACTTAATCTACTCCCACCCCGTTCACAAATGAAAATTAAATAATTTCTCTTTCAAAAATTAATATGAAATGAAATCAATCCCTTCAAAGAAGAATCTAACACACTCTGCAAAGCAATATGCTAATATGTGAGTTTAAATATTTATTTTTGTTTACGAAGTAATAAATAAAAAAATAAAAACATCCATGAAAGAAAAATTAAAAAAATTAAATAGTACTTTAGCCACAAAAAAAATACTTTGGATAGCTTAAAGCCTTAAAGAGAAAAAAGAAATAAACATAATATTGAGCTAAATACAACAGTTTTTTTTATGTATTAAATTATTTAGTATTCAAAAATTATATTAGACAAAAGAGTAATTAATTTACAATAATATATCCTGAAAAGC

>Cs1g04000

GGCCATATTTAATTGCTTGATCGAGAGTAATTTTCATCCCATTATGATCATGATCTTTAGGATCAAGCATAGATTCTATGATCTTCATTTTATTCCTAACATCCCCTCTCAAACTAATCTCAAGTCTCCATTTTGTAGTTAGGTTGTGAAATTAAAGTGGGTAGCTATGAAGAATCTTTGATAAATATCAATTAATAATATTACTATTGGCAAAATACTGAACATAATATGAATTTTTAATTGAAAAAAAAACTGTTTACCAAAGCCACTTTTAACTTATAAATTAAGGGTTAAAAATTAAGGAAAAAAACACCTACATCCCGAAAGTATGAAAAATGGTCATAAAAACTTTTAAATTTTCACTAAAGGACAACAAAATGCCTTTTTGACTGTAATGTCTTTTGTATTTCATAATTAGAAAATTGAATTTAAATTTTCTTAAAAATAATTTAAATTTAAGACAAATAACATATTATATCTAGTAGACTAATAATATTAAATTATTTTTAAGATTTTAAATTTTAAATTATTATACTTATTTTAATAATATATTTGTTTTTATTATTTTCATTATTATTTTTATGAAAATTATATTAAATTATTTTTAAGAAAATTTAAATTTAAAATATTTTATAATTATAACTTTCAAACTATTGCTGCTAATTAGTAATATTAAAAGTAACCTCACAGGAGGTAAGTAATAATTTCCCATTTTAAAAATGGATGACGAAATAATTACGATGGGCAGATAGAAATTTACAAACATATCTGGTCTCAGATTGAGGCTGAAATTAAAGGAAAGGACAAACAAGTAAGTTCACTTGATAAAGGTATGTAACTTCCACCTATAAATTGCTTGCAATTAATGAGATTCTTATGCAAAGCACATCTCTTTGTTGTAACAAATAATTTCTCTGGAAATTGCAAGGCAATGAAACCAACCACGAATATTACTTTCTCCTCTCTTTTTCTTGCATTGCTTACCATAGCATCAATCAACA

>Cs1g13480

AGCTTTTTGAATTTGGGCCCCGTTTTGTTTAACCCAATAAAGTCATAAGTGGGCTGTGTAAGTGACAATTGCGACGGCTTATTTTGCAGTCGCAAGAGCTGTCGCGAAAGACACTTTCTTTTGTAGTGTGGATTCATTTGGTTTTAAGGTTGGACACGGTTTATGATCCAATCATCCAATCTACCAAAGGAAATGCTGATTTTCAGGTTCGTTTTCGAGCATTTGCATTTCATCTTCTCAGATATTGAACGGGTTCAATAAATATTTACATCATAATTTTTATTTTGAAACTTACATCATAAGGTTAGTAACTTGATCTTAATGACTCCAACTTATATGCGCCACATGACCAATATTAGATACATAGATATTTGTCAAAAATCTATATTTTCAAAGTTAATAACCGTAAAGATTACGAAGGTTTGTTCGCATTAATTGAAGGACTTGAAAAAACATTACGTTCCTTCAATATATTTGGCTCAAACAATCACATAAAAAGTCCAAGTCTTATTCGGGGACACCTATCACCCAGTGATGGTACCTTCTAAGACTAAAAAATTGACTTTCCTAGTAGAAACATATAATTGAATTTTTTTTTTTTTTTTGATATTAATCACGAGGTATCCTGGGGAGGGCCCCAACTGTGGGAGGCACCTTTAAGCCTATACCACAACCCAGACTAGAAGTCCCCGCTCGAACCGGGAGGCACAGGTTCTCCCAACAAATACGACTTCCCTGCGACTCGAACTGGGGAACAAACCCAGTCAAGCCACTTAAGGGGACTCCATTGCCAGTGGGGCCAACACTTTGTTGGTTATAATTGAATTAATACCACCCGTCTGGTAGAGAAATTTCTAAGATTCCTACATTAGCTTATTCCCTTTATAGTTTATCCTATAAAAAGAATAAGTTCCATCAATTAACTAACATTGCAGTGCTCATACATTCTCATCAGATTTCTTCATTTCCTAAATAGTTGAATATTGAATTCTATACAAACT

>Cs1g12870

GATATTATGCAGCGAGCAAAATAAGGTGGGCAATGAAAAATGTGATGTCCATACGCAAGATAAGAGAGATAAAAAATCAATTTAGTTTTTAAGCAATAACAGCTAATATTTATCGAATATTAATGCTGTAGCTTCTCCAACTTTAGTACTAAAGACAACCTTAATTCATTTAGATGCTGTGATTTGTTTCTGATTTTAATTGTTAATTCGGATTCCACAAGTTCAATTTCCTAGTGTTATTAAATATGATGGATTATGTTGATCATCTTTGATATAGTTCTCTTCTTTAATTTTCAGAGCTTGATAATCACTTTTCAGAATGCCATCATTGTTCACCCCTCTCAAAATTTCATTCTTTTGCCTGCTGCTCAAATTTTTTTTCTATATTCCTTCGGAAAATCCATTTTAGCTGCACGAAATAATTTGATTCTTGACTGTGATATGAATGAACGAAAAATACAATGTAGAATATTACCAATTTTATGTAAATATATTTTGGTTTTGAGTTGTAATTTCTGGTTTCAAGACTTCGATTATTTCGTTCCAAGGGGTTTTTTTTTAGTAGTTTGTTGATTTTAAGACCCCAAGAATGAATTGAATTTTCAAGAGAAATTTCATTACAGAGCACGAGTTTGGTGCCAAAGTTTGTGAATGAGTTAGTCGCCTTTCCATTTGACACGTTTCTTACACGTGTTAAGTTTGTGGTGTTACAAATGTCTTTAGACGCGTGATGCCACCTGTGATAGCCCCGTTCTCGACACAGCTCTAACGTAAACTTAAATAAGAAGACGACCCATTCATCTCAAAATTAAAATATCGCCTCTCGAATAACCAAACGATTGACGTCAAACGAAAAACAACAACCTGTCCAGCAAAGAAAATAACACAAACACAAACACTGTTACTTAAACGGCACCGTCACCCAAAAAAAATTTGAGCGCCAAATTTGAAAAGCTTCAAAAACTTCCCCGCCAAAAGCGATGACCAGAAGTTAGATCACA

>Cs1g07240

TTTTTAAAAAGATAATGAAAAAAATTGGAATTGTGACCCTAAAAAATAATCAACTTGAAAAACTAATACATATTAATATATTTAATAAATTATGAATCAAATGTGAAAAGTAAGTAAAAAAAAAAAGGTACATATATTTGCTTTTTGCACTGGGATTAGTATTCGTTGTTAAGCCCGGCTGGTGTTAAGTATTGAATTAATGGGCAAGAGTTGTGAAATATGTATCCAATTAATTTCTAACCATTGATTTGGTGCTTAGAACAAGTTAAACTGAGCACCGAATTCATTTCTTCTTATAAGTGGGTAGAATGAGACATCTCATTAATTATTATCTCATGGTATGTAGGCCATCCATTCCCTTGTCTACCCACTTGCGTGAGCAAGCTAAATTACCATTTCTAGTTAGTAACATTATGGTAAAGCCGCGTCCCATTGACTAGTTTTGTGCCTTCATCATTTATAGTAGCACTAGCACATGAAATCCCATTCATTTTTATCATTTATAGTAGCACGAGCTGATGACATCCCATTCCCATCTTCAAGCTCACAGAAGCTACACTTTGTAAATTATATAATAACCTTCGGCTTCATTGTTTCATTTCCACACTGTTTCCTTTGCTTTTCTGGTAGATAGGAAAACTTGAAGATCAAGGGAACAGTACTGGAAGATCTCTGGCACGTTTTCTGTCTGTAGCATCACTTCTCAGGTATGGAATCAGCAACTTTTTTTTTTTTTACTTTATTTTTACTCTTCTGTTATCAGCTAAAGAGAATGATTTGAGTTTTCACTTTTTAAATTTTTATTTTAATCCTTTGAAGCTCTGGAGATAGCTACAAAATTTTTAAGTCTCAAGCAGTGCACTTGGATTCATTTTTATAGTTTTACTAGACTTTGAAATTTTTAATACTAAAGAAATTTTTCAGACGTCCCTAAAAATTTACAGAAAGATGAAAAAAATTAAAAAAATTAATTTGTAATTTTATTAAAGTTAAACAGTCGG

>Cs1g21270

GTATCCTGATCAGCTTCCATTTTCTTGCCATTCTCTTCATCTTCCATTGAATCGCCGCAACATGCCATCATTCTCGTGATATTTTTCGCCCCAAAGAGTTAACAGACTTATAAAATTGATGTTCACTGTGGTTTTAACTGAATTTGGAAAATTGGAGCCAAGAATCTGGAGGGGCGTCCCCTCTTTTTATAGAGTGGCAAAGATACTCATTTTGATGTGGAAAATGGTAACGGCCTTCGAGTGAACGGTCTGCCGTTAGATTTAGTGGGACCAGTGTTGAAATGGATAGCAGCGATTGCGCCCTGGCCGGGGACTGATTGATAGCGGCTGATAGAAAGGGACGACGTTTTTCAAGTCATAAGACGACTATCTCACAGAACGGCAGTGGTTCGGCTTGTCGTTTATATTGAAAGTTGAAGCCGGTAAATGACGTAGTCTGGCATTGCCATTTGCATTACGTACATTCTTATCGAGCAGGCAAGGAAGTCAAGAGAATAATTAATGGTTTGTGCAAAAAAACAAAGCGAATATGAGAAATAAAATGAATGACTTAGCGTTGGTGCCGTGTTAAGCAGAAAAGTTCTCATGATTCAGACGTATGCAGTTCTCTAGAAGTAGAGTTATTGTGCTGCCAATCATGACATTTAGATTTTATACTAAATGAGATTGATGGGCGATGTAGACGACAAATACAAAATAGAGAAGACAAAATTTGTTTCGATGATTCCATGATCATTTATTTGATTGATTCATATATACTCGAGTTTGGCGTTTGCTAGCTTCACCTTGGCCTTGGGTGATGGAATCAACCGAGGGGATAGCGAATCGAGGTAGTTTTATTTATTTATTTATTATTATTATTATTATTTTTGGTGAAAGGTTTGGAAGTAGTATTTAATTTGCCAAAAGCTTTTATTTTTATAAACAAAATTTATTTACGAAAAAAATGGAAAAAGACAGTACCCATCACACGCACGCGTATAAAACAATAGCACACTACA

>Cs1g07430

TTTTTTTTGATTATTGTTGTGAAGGTTTCGACTGGTAATAAGTAAGCTTGGCAAAAAAGCACGTGTCCTGCGGACATAGACTCGATCCTTCACATGAGGGCTTGCAAAAAAAAAACTTAAGGAAAAAGAAAGGCCAAGTCAGGCCGAACCTCTCTTTCACTAATATAAATTAAAATAACAAAAATTAATTGAAAAAGAAAAAAAAAAGAAAACATAAATCTCTATTCATGAATCACTAATGATTCAAAAAATTTACGATTATAACATTAAACTCTCCCTTGGTCTTAATTAATTAATAAGATACTTGAATTAGAGAGTTCAGTACTAAAAAATTAAATATATAATTTTTTATTATTATTTTATTTTAAAATATTTATTTTCTTTATTCATTATTTATTATAACAACTTATTAATTCATTATAAACTTACAATAATTTAAAAAATAGTTTAAAAAAATATGTGAAAGTTCATCTAGACGCTTACACTTTTCTTAGAAAGAGTTTCAGCTTAAGCTTAAAAAAGCTTGGCCCAAGCATGCAACTTGCTATCATATTCATAAGGAGAACTTATTAAATGACATTGATTTTGGTGAAATAATTTATGTTTTTCGCTTACAACTTGCAGATTAATTATTTTCATTTATTGTCAAGGATTTGACAGTAAACTTTCAGGTCGTTCTAATTTTTTTTCTTTCTTTTATAAGAACAGAAAATTTCTCTTCCACTTTACTTAAAATATAATCTATTCAGTTTTTGTTGATATGATTTATAATAATTTTTGTTCGAAAAAATATAAATATTCTTTTGAAAGAAAAATACAGTTTAAAGAGACCATATAATGGTTTTTATTATTTAATTTTAATAAAAATAAGTTTAACTGGTTTGACATATGATTAATTAAATTTTTAATAATTAAAATTAAAATATTATCATAATTAATTTCTAATTTTATAAGCGGTTATATATTTATCCCAATGTTGTAATTTTAGAAGAGCATTGTAG

>Cs1g11180

ACTTTCGTACGTGAAAATGAAAAGTAAAGTTGATCAAGCGCCATCTTCCCGTTTAATAAAAATGTGCTTATGTGCATGCCCTTTTCTCGTATATGTGTTGTGGACTTGTGGGTGCACATAATGGGGTCCAAAATTGAAACCCAATGGGACGTTCCCACGTGTCGTTTTGTCGTCTTGTGGTGACGAAGCACCTGGGGACGGCGACAAGTGGCCGACAAGTAGATGGGGAAGTTGATGTGGAGAGGCCCCTTAGTTGCCTCCCATAGTGCTTGTTCATCGCAAACTTTATCATGAATTATTATTCATCGGCATTGTTCGCAACATGGTCTCAATTCTCATCAGGTCTTTTGAGATAAGCATCAAAAGTCGAACTTTAAGTTGATAAAACAGAAGATAAAAAGGGACCGATGTCATTGATTGTGAAAGCACTTACCATGTTTGAGATTATCTGCTTTGTCTATTGGTGAGAGAGAAATTAATAAAGCTAATGAGATTCCTTTGTTGGGTTTGCCTTTGCTTTGTCTAATCAAATTGGACCCCAAATCGTGTTGTTAGATTCCTCTGTCCATCTGTATCATGTTGTCAGTAGATTGGAACTTGTTTCAAGAGAAAAGCCATAAAGTCTTTTGCATCACTATCTCAATTGATATTCGCTTCCTCCTAAAATTTATCATCAGAACTTGCATATATTCCTCTTCTGTGCACAAGCACAATCATGTTTAGATACCACATGAAAGATATGGCTGCTTAGAATTAAATGTTTGACTTAGGATTAAATAATTACAAGTATCTATGGGTCAAATAGTTATTGTCAAATATTTACATTATATAAGTATGAATTGACTCAATGTGGGAAACCCCTTGGGATAGTTTTTTTTTTTTTTTTTCATTTTTTTTCTTTTTCAGTAGAAGATCGGCAAAATCCAATTTAATTTTTTTAACCACTACCCATCTCAAGATTAGATCCCTGATGGACCCTTCTCTTAGTTCAGTAGACGAGG

>Cs1g10630

ACACAACTGTAAGGTTGTATTCTAAGCAATGACAATTTAAGTAGCAAAACAAAGAAAATTGATGATAAAATATGAGAATAAAGTAGAGAGGATTAATTATAACAATTCATCTAAATTTGGGACATTAGTATTTTGAAATATCAATAAAATTATAACGATATATTAGGACAATATTACTTGTGTTGCATAAGTTAAAAAAGAGTATATCAAAGAAAATATAACCAAATACAACGTCTAATTAAAATTTTTCTACACTCATTTGCTCCAACGGAATTAGAAAAATGATATCAAATAGGTACAATACACTAATAACTTCTATAGATCTATTTACCAAATCTTTACTGACATAAGCTTTTGAAAAACTAGTACGTAACATTAGCATGCGTTGGCTCTGCAGATTATAAAATTGACATATGTTATTTTAAGGAGGGAGGTTTTATTCAGGTGTACTAATCAAGTCCAACACTACTTTTAATATCATAAAAATTATATGTAAAAACTTAATTACGTATATATTGATCGGCTCTTTTTTTTTAATTAATTAAGTATTAAGTTATTATATTATACATATATTTATAACTGTTATGATAACAGATCATTACACTGATATTTATAATCAGATATATATATGAGACTTTATTATATTTTTATTTTATGAAGTACATGCCTAGCCAAATACTCATTACAAAATAAAAGGATATCTCTACTTCACTCGTATTTTGCAAGGAAAAAAAAAGTTACAAGTAATTTCCACACAATTATATTTAATTAAGCGATGTTAAATTAATGGGAACTCGAATCATTGTCCTCATAGTCATGCGATCGGCCCTTGTTGTATGGGACTTAAAGTGTGGCATTTTATTGTCAGGGGTATTTTAGATTTTTCATTCTTGAATAATTGTCATATTTGTGAATGAAATGACTGCCGTGCCATATGTTTGCAAATTTTAACCACCTACGCAGCCGGTGCAAACTCCGTCATTACAGTTTTCCTTTTCCTC

>Cs1g24600

TCATCCGCTGCTGCAACCACCACTCCTAGTGGTTATAGTGTTACCGGGGAGGGGCAGATGCGCCACCACGAGAAGAAAGGAATGATGGAGAAGATCAAAGACAATCTTCCTGGACACCGTCACTAGACTACCTGCCTGTACTTGTCTTTCGTCGACGACACATACAATACTATATATACTAGTGCTTTTTATTATTTGCTATATTTTACACGAGTGCTTTTGTAATATGTCCATAATATTCGTAGTTTGGCAAATGCTACATTACAATTAATGTAACATACACACTCAACAACAACCATACAAATGATGTGGGCCTATTATTTGTGTGGTTGTTGTTGAGTGTGTATGTTACATTAATTGTAACATAGGGACTCTCTCGTAGTTTTATATATGTCCATCTCTTTTTACTAGTTATGCATCTTGGGATAGTATTATGTAACACATACAACAAGCTGTCAAGCTATTTGTACTAGTGGGTGGGCTGTTCCTTTAGTTTTGTGTATTAATACAGCTTTCTATTGTATTTTGTCATATATATATATTGAACTAATTAATAAGAGTTCTTTTGCTAAACGTTTTTTTATCGATTTTCCACACTACACCGCACCTTAAGCCTGCTTATTCGTATTACAAACTTTCATTTCTGAAACTAGATAGTATCCTGATTCTTACAGCTTATAAGACATGCCAACGGACGGCTTGTCGGTATGCTCTTTCGTTTCAATTTAAATGACGTACTGGAGGAGAAACAGCGGAGGACATCTCTCATAACGAGTATCTGTTATTGGGGATTTTGATAATATGGTGAAAAAACAAAAAGAAGTAAAGAACTCAGCATCAAAATGTGAGAAATTGGTTAAACCTCAGTAATAGAAATTTGGTTAAACATTCAACCAATTTAAGACAAAACATCTTTTAACAATCCGACGTTGAGAAATAGAAATTTGCCTTTGGCATCACGGATGAAACAAAAGCCATTAGTATGATACCGGTACCCTTTC

>Cs1g07480

ATTATTTTAATTTAAATATTATCAAAACTCTAACCATGAACAACAATAATGCCTCGAAGCTGAGAGTTTTTTGTAATTTTATTTTATATTATTTACTTCGAATTGTAATTAAACTATCTTATCCTCTACTTCTCTGTTTTTATTTTTTGCCTTTAAAAAGTAATTCGATAATTGATTGGAATTTGGATGTGTGTTTATCCTGGAGAAACAGGTAAGAGAGATTGATCTGTACAGCAGATTTATAATTATAATAAAATTATTATTTTATTATAATTATAATTCATTTAATAATTATTATATTTTTTTAATTAAATAGAGGTTGTTTTTGGAATTTAAAATTATAGGGGGTGAATTAGTACTCTAGAGTTTCAATAAAATAAAACAGAGTACAAGGGGTGTTACGAAATTTTAAAATTCAAAAAGTTTTATAATATTTATGAATAAATTATAAATAATATAGAGTATAGGGGGCTAAAAATAAATTTACAGGGCAGCTATGTAACTTAGGCAGGCCCGGGCAAAATTTATGATTGCACCTTGAGAAACCAAATTTTTAAAAAACACTGAAATCTTATATATTGAAGAGCAAATACATAATTTTGGATATTTTCTATTTTAATTTTTAGTGATAAATTTAATTTAAATCAATTTTTAATATTGAGTTACACATTTGGACTAAACTAGATCAAATTTTAAATTAGATATTAGGCCTTATTTTAATGGATTCCGAGTCAAGCAACTAGGCTTATAAATTATAAATGAAAGAAATATGTGTGTGATTTTAAAATTAATTTTGAACTTGAATTTATACATTTAATTAATTCAAAGAGGTTTGGAATATATCATCTGAAATTTTTGGATGGGACTTGGTTAATGGTTGTTGGTTAGGGACCTATTTGACACATATGGACAGCATTTATCATAATTTGGTAAATCATAAAGATTAAGGATTGGACGTTGTTACCGCAATCAGAAAATAACTTTCATCGCAATAAATGTGC

>Cs1g10380
[truncated: 117,417 more chars]
