# Supplementary material for: Consequences of adaptation of TAL effectors on host susceptibility to Xanthomonas
Source: PLoS Genet. 2021 Jan 19;17(1):e1009310. doi: 10.1371/journal.pgen.1009310 (PMC7845958; doi:10.1371/journal.pgen.1009310)
Supplement: S3 Fig — Schemes represent the alterations observed in the adapted dTALEs compared to their parental dTALEs and the predicted recombination events, which led to the adaptation. Repeats that were likely to be subjected for recombination or deletion in the parental dTALE are underlined. Repeats in the adapted dTALEs that were altered as a result of recombination are underlined and marked in blue. Repeats that were deleted are marked in purple. A. Alteration observed in dTALE2A1 compared with dTALELBM2. B. Alteration observed in dTALE2A2 compared with dTALELBM2. C. Alteration observed in dTALE3A compared with dTALELBM3. D. Alteration observed in dTALE5A compared with dTALELBM5. E. Alteration observed in dTALE7A compared with dTALELBM7. (PDF) [file pgen.1009310.s003.pdf]

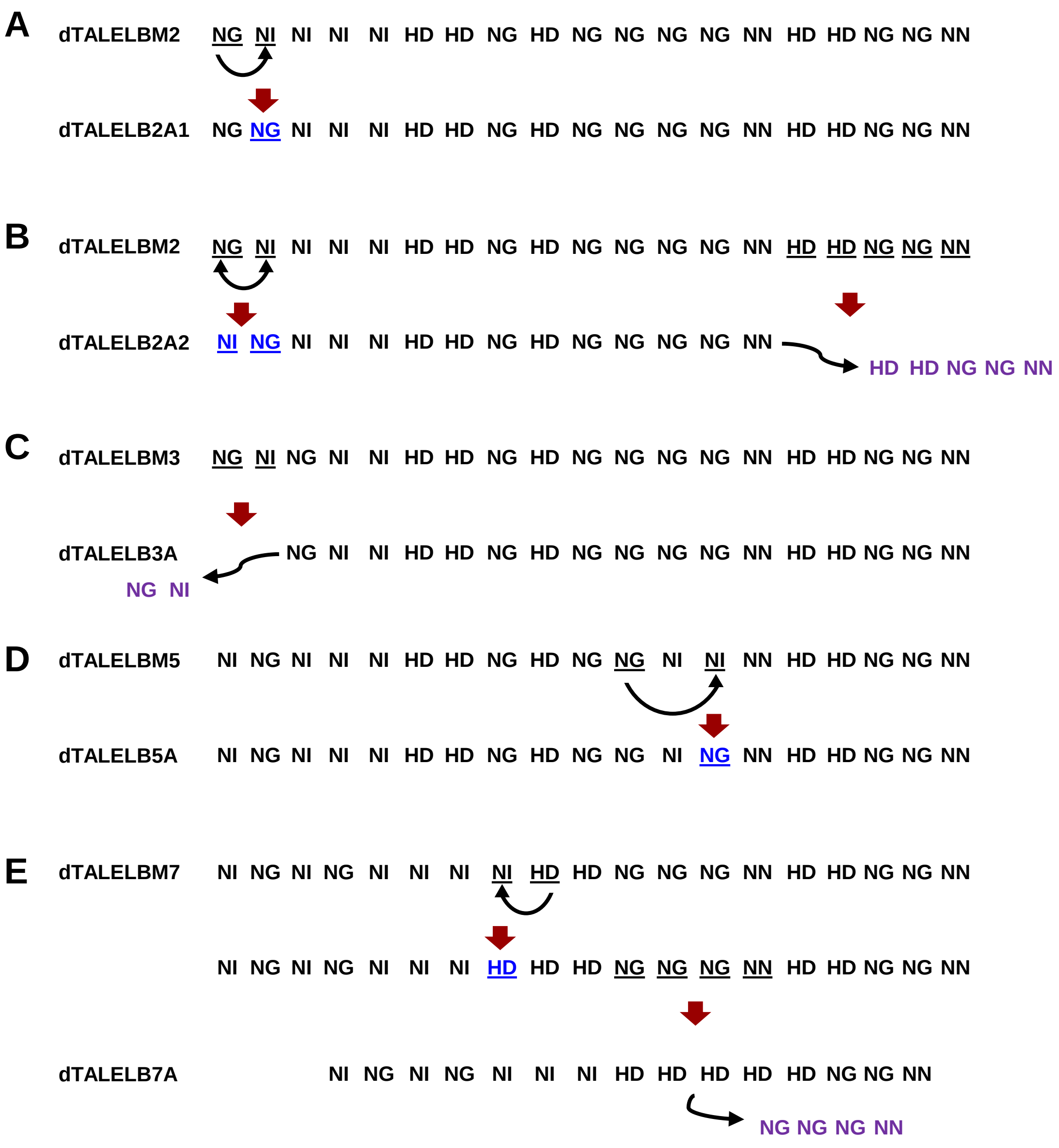

S3 Fig. Predicted recombination events that occurred in adapted dTALEs. Schemes represent the alterations observed in the adapted dTALEs compared to their parental dTALEs and the predicted recombination events, which led to the adaptation. Repeats that were likely to be subjected for recombination or deletion in the parental dTALE are underlined. Repeats in the adapted dTALEs that were altered as a result of recombination are underlined and marked in blue. Repeats that were deleted are marked in purple. A. Alteration observed in dTALE2A1 compared with dTALELBM2. B. Alteration observed in dTALE2A2 compared with dTALELBM2. C. Alteration observed in dTALE3A compared with dTALELBM3. D. Alteration observed in dTALE5A compared with dTALELBM5. E. Alteration observed in dTALE7A compared with dTALELBM7.
