# Supplementary material for: Consequences of adaptation of TAL effectors on host susceptibility to Xanthomonas
Source: PLoS Genet. 2021 Jan 19;17(1):e1009310. doi: 10.1371/journal.pgen.1009310 (PMC7845958; doi:10.1371/journal.pgen.1009310)
Supplement: Attachment — Submitted filename: Teper and Wang.pdf [file pgen.1009310.s010.pdf]

In this well-written article entitled « Consequences of adaptation of TAL effectors on host resistance against Xanthomonas » Teper and Wang investigate the potential and the mechanisms of and through which Xanthomonas TAL effectors repeats evolve to adapt to incompatible DNA target sites within promoters of host susceptibility genes. Here, based on the analysis of PthA4 which is a major virulence TAL effector in Xanthomonas citri (the causal agent of Asian Citrus canker), the authors evaluate the capacity of PthA4 variants harboring from 2 to 9 mismatching repeats preventing for the induction of the PthA4 matching S gene CsLOB1 to evolve new repeat arrays allowing for virulence recovery. The relevance of the project is particularly high knowing that deployment of resistance strategies based on TALE-mediated loss-of-susceptibility has mobilized major interest over the last decade in several economically important crops threatened by Xanthomonas species relying on TALE effectors.

Overall I like the results and appreciate the hard work which was elegantly designed and achieved but I am worried about the "too artificial" conditions of the experimental evolution. Because such approach is artificial in essence, a detailed section in the discussion taking a step back on the limitations of the strategy would be more than necessary. I am personally not convinced that TAL repeat changes observed in this assay would occur in a field.

Major comments

As illustrated in Figure 2B, the authors repeatedly alternate phases where the bacteria are grown on synthetic medium versus phases where the bacteria are in the plant leaves. This means that bacterial populations are also evolving on NA medium. How does this influence the overall strategy ? Can one exclude that some adaptive mutations occurring in planta were counter-selected when bacteria bearing them where grown on plates ? Or that mutations have appeared during multiplication on plates and that they were next selected in planta, thus resulting of an adaptation to an artificial medium and not the host tissues ?
I see this as an important conceptual gap.
I recognize that the experiment is carefully conducted and that results are very interesting and worth being published BUT they should be interpreted with more caution, taking more this strong bias in consideration.
In the same line of idea, the assay is based on over-expressing a dTALE from a high copy plasmid which probably favor intra versus inter TAL repeats recombination. I am actually surprised that no modification veseem to has taken place at the pthA4 locus leading for instance to a loss of Tn5 : has this been verified ? Having included as negative control Xcc pthA4:Tn5 bearing an empty vector in the experimental evolution assay would have helped to exclude the potential for other sorts of extra-dTALE modifications to be occurring).

The authors should at least show that they are aware of these caveats and be discussing them. They also should rationalize their choice : why did they have to multiply bacteria on plates between each passage on plant ? Was it on purpose ? For technical aspetcs ?

Minor comments

- Specify in the title that this article is dealing with loss-of-susceptibility mediated resistance.

- Figure 1

Fig 1A-C : To complete the analysis about PthA4 variants highlighted in Figure, a phylogenetic analysis among the 13 allelic variants would be useful and give more weight to this interesting analysis depicting the relative affinity of 13 unique array variants to the pLOB variants generated from a diverse set of Rutaceae plants. A bioinformatic tool called DisTAL could be useful for this as it evaluates phylogenetic distances between repeat arrays: https://doi.org/10.3389/fpls.2015.00545.

Fig1D-E tend to experimentally validate the predictions reported in Fig1C. Fig1D is useless because signals are quite weak overall (even for PthA4 on sweet orange), thus requiring a quantitative analysis which is shown in Fig 1E, albeit without support of a statistical analysis. Please do it.

- FigS1 : The authors miss to compare the performance of Xcc pthA4:Tn5 (dTALEWTLOB1) to the WT strain. Would be good to know the full level of complementation.
- FigS2 : why so many protein bands on the left membrane ? Why not on the membrane to the right ? It should at least be indicated which band is expected to correspond to the TAL analyzed.
- In the M&M section (L385-389) the authors refer to evaluating potential re-arrangements when bacteria are grown on plates but I missed to see the results of this experiment. Is this reported or just mentioned here. If so, can one clarify what has been tested to show that no ".. repeat alterations in the dTALEs isolated from bacteria grown in artificial media" has been observed ?

Other minor remarks/typos :
L49 : not all TALEs are vital for bacterial pathogenicity, please correct.
L59 : one ref is not edited correctly (Kumar Verma et al 2018).
L61 : better refer to a review here.
L67 : earlier pioneer article that would better fit here.
L78 : either cite one or two complete review articles or refer to all original articles but mixing randomly both is weird
L260 : point mutaions
L266 : suggetsed
