# Supplementary material for: Consequences of adaptation of TAL effectors on host susceptibility to Xanthomonas
Source: PLoS Genet. 2021 Jan 19;17(1):e1009310. doi: 10.1371/journal.pgen.1009310 (PMC7845958; doi:10.1371/journal.pgen.1009310)
Supplement: Attachment — Submitted filename: Response to Reviewers R2.docx [file pgen.1009310.s011.docx]

Reviewer's Responses to Questions

Comments to the Authors:

Please note here if the review is uploaded as an attachment.

Reviewer #1: This is a very interesting study on host adaption of a bacterial pathogen. The authors used a unique system (PthA4-LOB1 interaction in citrus canker disease) to show host adaption in Xanthomonas can be achieved by mutation of a specific TAL effector. The authors first examined the natural variations of PthA4 and LOB1 EBE sequence, which suggested they determine specificity of host-pathogen interaction in the disease system. The authors then designed pthA4 TALEs that had low binding affinity to LOB EBE, thus cannot promote disease after being introduced into the bacterium. Through many cycles of re-isolation and re-inoculation, some bacterial strains gained the ability to cause disease on the host and it was found that designed pthA TALEs in those strains mutated to a form with a stronger binding affinity to LOB EBE. This study provides the first experimental evidence that Xanthomonas pathogens can gain the ability to cause disease on a specific host by mutation of a single TALE. This may also give us an insight into molecular mechanisms of the evolution of bacterial pathogens, particularly for Xanthomonas. The experiments are well designed and executed. The manuscript is well-written and organized. The following is the list of comments and suggestions for the authors to consider.

1) The study can be strengthened by using a natural bacterial strain that has a low binding affinity pthA4 TALE, like RVD11 in Table 1. The study could also explore the possibility of PthA4 wild type TALEs to gain virulence on genetically modified LOB EBE resistant plants, which could provide support the authors’ argument about the potential durability of genetically modified plants.

**Response**: Excellent point. We agree that using a natural TALE with low affinity to the EBE of LOB1 or the native Xcc strain against modified citrus lines could have been a good model to explore in parallel or instead the path we took. As of now we only have one homozygous line that contains one single nucleotide deletion within the EBE of LOB1. As a tree crop, it is quite challenging and time consuming to generate homozygous or biallelic mutants for citrus plants. Therefore we could not take this route right now. However, we are in the process of generating multiple lines of mutants in the EBE region. We will do so when the plants are ready. Using natural TALE with weak binding affinity to the LOB1 EBE (if available to us) could have been a potential route to choose but it would not mimic a “disrupted EBE” scenario and therefore we decided to proceed with designer TALEs instead.

2) The experiment was conducted solely in greenhouse and laboratory. The authors should discuss if the process described in the study could occur in nature. How are the authors going to test?.

**Response**: Excellent suggestion. We believe that introduction of citrus homozygous lines that are disrupted in the LOB1 EBE into the field and monitoring them for canker in area where Xcc is prominent would be a good way to test our model in a more natural setting. We added this suggestion to the discussion as a downstream study that could be conducted.

3) The authors stated that dTALEs carrying seven mismatches did not have any sequence change after 30 infection cycles. If TALEs tend to have high rate of mutation because of the repeats, why the bacterial strains carrying seven mismatch TALEs did not mutate at all, but those carrying less number mismatches did.

**Response**: This is an excellent question. We think mutations can occur to all constructs including dTALEs carrying seven mismatches. However, the probability for strains carrying less mismatches to overcome the mismatches via mutation is much higher than strains containing more mismatches. The mutated constructs that overcame the mismatches will enable higher fitness for the strain, thus led to takeover of the population. For the mutations that did not bring increased fitness for strains that carry the dTALEs containing more mismatches, it is just difficult or impossible to detect such mutations at such low percentage. We discussed this issue in the discussion section of the manuscript.

4) Introduction should include some published information about host adaptions by other pathogens, like what other mechanisms underline host adaptions.

**Response:** We dedicated a paragraph elaborating known mechanism of adaptations to specific hosts by various pathogens covering studies conducted with different methodologies (population analysis in the field, reverse genetics and experimental evolution) to the introduction.

5)

A. Fig 1D labeling is not very clear.

**Response**: We repeated the GUS staining to achieve better labeling.

B. Fig 3C has so many colors, which is not easy to match with labels.

**Response**: We separated Fig 3C to four graphs, each representing the original TALE, the adapted TALEs and the controls. We hope that makes the data more friendly to the readers.

C. Fig 4A, it would be good to show the DNA sequence for each mutation.

**Response**: The DNA sequences of all the dTALEs used in this study are now available in supplementary file 1. The DNA sequences coding for the RVDs are color coded for convenience. In addition, we modified Fig 4B so it will show the affinity of each RVD to each nucleotide in the EBE of LOB1.

D. Table 3 last two columns need more explanations.

**Response:** A more elaborate description of the last two columns is added at the bottom of the table.

E. Fig S2 needs to explain why there were different sizes of bands in each well. It looks like some had more bands than others.

**Response**: The top bands matched the predicted full length TALEs and the other bands appear to be degradation products. We modified the protein extraction and running protocols to avoid protein degradation and repeated the gel running. The new western blot (S4 Fig) contains only the full-length bands.

Reviewer #2: This manuscript reports on an interesting study of adaptive evolution of artificial genes encoding transcription activator-like effectors (TALEs) in the bacterial citrus canker pathogen Xanthomonas citri subsp. citri (Xcc). Starting with a mutant in the tale gene pthA4, which is unable to induce the expression of the LOB1 susceptibility gene, the authors followed the emergence of new bacterial clones that re-gained the ability to induce LOB1. This ability was acquired over time from artificial pthA4 gene variants that were not able to bind to the corresponding promoter element of the LOB1 gene, called EBE[PthA4]. All in all, repeated inoculation into and isolation from sweet orange for a duration of 30 cycles allowed obtaining adapted, LOB1-indicing tale genes from constructs that had two-to-five mismatches with the EBE, but not so from constructs that had seven-to-nine mismatches. The observation and characterization of such novel tale gene variants that evolved in about 1000 generations or less is important information for attempts to generate disease resistant crops against TALE-dependent pathogens.

Despite the general interest, this study can only be considered a first proxy because the artificial tale genes were cloned on a small high-copy number plasmid (pBBR derivative) whereas natural tale genes are encoded on the chromosome or on large conjugative low-copy plasmids. Therefore, mechanisms and speed of evolution can be expected to vary dramatically between the experimental setup in this study and the situation in the natural environment.

**Response**: Excellent comments. We agree with the comments and revised the manuscript accordingly in the discussion section.

Another criticism concerns the conclusions about affinities of natural and evolved TALE variants and their potential to induce the downstream gene, which are merely based on one computational algorithm (TALE-NT 2.0). Only two variants were characterized experimentally, which is by far not enough to speculate about adaptive evolution and enhanced affinities/activities of the time course of the experiment. At this point, the study needs to be more quantitative and the different TALE variants should be tested experimentally with respect to reporter gene activation. One may even wonder whether or not high affinity is required at all or if a threshold induction level would suffice for symptom formation?

**Response**: We thank the reviewer for the insightful comments. The experiments depicted in Fig. 3 used evolved TALEs that were reintroduced to the *pthA4* mutant and quantified the effect of the TALE on the expression of the *LOB1* S gene using qPCR. This is a quantitative experimental evidence that supports that the evolved TALEs has higher affinity to the EBE of LOB1 when delivered *Xcc* in their native infection system. We did not observed any induction of LOB1 by the non-adapted TALEs when delivered into citrus.

The TALE code was deciphered more than a decade ago and was validated by numerous studies. Therefore, the prediction analyses are appropriate. The analyses described in the first part of the result section regarding the variation in the TALEs and the LOB1 promoters were conducted to assess variations found in nature that are interesting as standalone observation.

The threshold level of induction that is required for symptom development could be an interesting independent study by itself. We think it is beyond the scope of the current study we present here.

Additional remarks/corrections:

Line 59: Problem with Ref. Kumar Verma et al., 2018.

**Response**: The reference was removed.

Line 83: AvrXa7

**Response**: corrected.

Line 148: It is not clear where the Tn5 is exactly inserted, and the reference does not help either because 50 random Tn5 mutants in pthA4 were obtained in that study.

**Response**: The Tn5 mutant of pthA4 has an insertion at poison 770 bp in the ORF of pthA4, which corresponds to clone 320E11 (Yan and Wang, 2011). We verified the insertion site by amplifying and sequencing the area. The information regarding the Tn5 insertion site and the transposon orientation was added to S1 table.

Line 213: It is not clear why PthA4 is not mentioned here? Codons from pthA4 should be present in the strain, even if Tn5 has disrupted the gene. In fact, the study would have been more instructive if the authors had chosen a deletion mutant in pthA4 – or even better a gene replacement with the artificial construct containing codons normally occurring in pth genes – instead of a transposon mutant. The question whether intra- or intergenic conversion leads to new tale variants is indeed very interesting and could have been much better addressed with such an experimental setup.

**Response**:

A. “PthA4” was added to the sentence.
B. We thank the reviewer for the insightful comments regarding that it is more ideal to use the dTALE mutants in the background of pthA4. We could not do that because of technical challenges in making such TALE since it’s impossible to use PCR based substitution/deletion methods when the area you wish to substitute harbor 16 other identical regions to it. Manufacturing dTALEs with identical codon usage to the one of PthA4 requires de novo synthesis of all the TALEs used in the study, which is very costly that we could not afford.
C. We agree with the reviewer that the question whether intra- or intergenic conversion leads to new tale variants is indeed very interesting. We think this mechanistic understanding of such issues is beyond the scope of this study.

Line 222: That’s the place where one wonders about real affinities (GUS assays, see above).

**Response**: We monitored the expression of the LOB1 transcript in the host during *Xcc* infection by using qPCR with all the dTALEs used in the study as a marker for the activity of the TALEs in a natural system.

Line 231: This is again speculation based on predictions. Why not performing transcriptome profiling using RNA-seq, or at least study these candidate genes by qRT-PCR?

**Response**: The two canker associated genes mentioned in this sentence are reported to be induced by PthA4, presumably downstream to *LOB1*. Since LOB1 itself is induced by the adapted dTALEs, it is difficult to determine whether their expression is directly induced by the dTALE or mediated by *LOB1*.

Line 252: It would have been instructive to see a scheme where one can easily follow the mutational events that might have led to the new phtA4 variants.

**Response**: We added a supplementary cartoon to clarify this section (S3 Fig).

Line 266: suggested

**Response**: corrected.

Reviewer #3:

In this well-written article entitled « Consequences of adaptation of TAL effectors on host resistance against Xanthomonas » Teper and Wang investigate the potential and the mechanisms of and through which Xanthomonas TAL effectors repeats evolve to adapt to incompatible DNA target sites within promoters of host susceptibility genes. Here, based on the analysis of PthA4 which is a major virulence TAL effector in Xanthomonas citri (the causal agent of Asian Citrus canker), the authors evaluate the capacity of PthA4 variants harboring from 2 to 9 mismatching repeats preventing for the induction of the PthA4 matching S gene CsLOB1 to evolve new repeat arrays allowing for virulence recovery. The relevance of the project is particularly high knowing that deployment of resistance strategies based on TALEmediated loss-of-susceptibility has mobilized major interest over the last decade in several economically important crops threatened by Xanthomonas species relying on TALE effectors.

Overall I like the results and appreciate the hard work which was elegantly designed and achieved but I am worried about the “too artificial” conditions of the experimental evolution. Because such approach is artificial in essence, a detailed section in the discussion taking a step back on the limitations of the strategy would be more than necessary. I am personally not convinced that TAL repeat changes observed in this assay would occur in a field.

Response: We thank the reviewer for the encouraging comments. It is always nice to know our colleagues appreciate our work. We have added some discussion regarding whether TAL repeat changes observed in this assay would occur in a field.

Major comments

As illustrated in Figure 2B, the authors repeatedly alternate phases where the bacteria are grown on synthetic medium versus phases where the bacteria are in the plant leaves. This means that bacterial populations are also evolving on NA medium. How does this influence the overall strategy ?

Can one exclude that some adaptive mutations occurring in planta were counter-selected when bacteria bearing them where grown on plates ? Or that mutations have appeared during multiplication on plates and that they were next selected in planta, thus resulting of an adaptation to an artificial medium and not the host tissues ?

I see this as an important conceptual gap.

**Response**: We conduct the experiment in the current settings mainly because we encountered technical issues. We initially started the experiment using plant system alone but encountered many problems with contaminations and strain purity that led us to discard the data and start again in the current settings after a few months.

We found that without selecting the bacteria between cycles using antibiotics some lines were lost due to outside contaminations of opportunistic fungi and bacteria and some Xcc lines lost the antibiotic selection were identified as well (which could be a resulted from the loss of the plasmid, the Tn5 insertion in pthA4 or outside contaminations of Xcc).

We agree that it is preferred to conduct experimental evolution studies by keeping the bacteria in the same condition and many traits are affected differently in the host when compared to artificial media. However, our focus was only on TAL effectors that are not known to have any positive or negative contribution to the bacteria outside the host. We did not observe any modifications in the TALEs when bacteria were cultured in NA media alone and therefore we feel confident that TALEs were not subjected to selective pressure in artificial media conditions. We also note that all the downstream analyses conducted with the adapted dTALEs (described in fig 3) were done with bacteria that were complemented with the dTALEs and therefore any TALE independent adaptation did not play a factor in our final fitness tests.

We speculate that if we would have been able to sustain the bacterial cultures only in planta we would have observed more TALE independent adaptations such as enhanced growth or stronger water soaking symptoms without canker. We are not certain how the alternative experimental settings will affect the kinetic of TALE adaptations. On one hand, using only plant environment will create more selective pressure on the TALEs to adapt; on the other hand, NA media might select against TALE independent plant-adaptive traits that could make TALE adaptation less competitive.

I recognize that the experiment is carefully conducted and that results are very interesting and worth being published BUT they should be interpreted with more caution, taking more this strong bias in consideration.

In the same line of idea, the assay is based on over-expressing a dTALE from a high copy plasmid which probably favor intra versus inter TAL repeats recombination.

**Response**: We agree that our simulation was conducted in a very artificial conditions and we added further discussion on the matter in the discussion section of the manuscript.

I am actually surprised that no modification veseem to has taken place at the pthA4 locus leading for instance to a loss of Tn5 : has this been verified ? Having included as negative control Xcc pthA4:Tn5 bearing an empty vector in the experimental evolution assay would have helped to exclude the potential for other sorts of extra-dTALE modifications to be occurring).

**Response**:

A. The bacteria were selected on NA media containing kanamycin (for the Tn5) and gentamicin (for pBBR1MCS5). By this way we selected against bacteria that might have lost the Tn5 insertion. We should also state that in our experience Tn5 insertion is usually not lost that easily. However, by subjecting the bacteria to selective pressure such phenomenon might have occurred if antibiotic selection was not used between cycles.
B. We agree that using the pthA4 mutant harboring empty vector could have been a good control to be added to the experiment. However, we note that we did not observe any adaptation in Xcc lines that harbored a large number of mismatched repeats, which by themselves can act as internal negative control.

The authors should at least show that they are aware of these caveats and be discussing them. They also should rationalize their choice : why did they have to multiply bacteria on plates between each passage on plant ? Was it on purpose ? For technical aspetcs ?

**Response**: We thank the reviewer for the excellent suggestion. The reason why we isolated the bacteria on NA was mentioned above. We added this in the materials and methods section.

Minor comments

• Specify in the title that this article is dealing with loss-of-susceptibility mediated resistance.

**Response**: the title was changed to “Consequences of adaptation of TAL effectors on host susceptibility to Xanthomonas”

• Figure 1

Fig 1A-C : To complete the analysis about PthA4 variants highlighted in Figure, a phylogenetic analysis among the 13 allelic variants would be useful and give more weight to this interesting analysis depicting the relative affinity of 13 unique array variants to the pLOB variants generated from a diverse set of Rutaceae plants. A bioinformatic tool called DisTAL could be useful for this as it evaluates phylogenetic distances between repeat arrays: <https://doi.org/10.3389/fpls.2015.00545>.

**Response:** As suggested we added phylogenetic analysis of the Xcc/Xca TALEs. The analysis is shown in S1 fig and data is described in the result section. As far as we can tell, there are two distinct clusters representing that Xca TALEs (RVDV12 and RVDV13) and the XccAW TALEs (RVDV5, RVDV6 and RVDV7) that appear to come from different lineage from the rest of the LOB1 targeting TALEs.

Fig1D-E tend to experimentally validate the predictions reported in Fig1C. Fig1D is useless because signals are quite weak overall (even for PthA4 on sweet orange), thus requiring a quantitative analysis which is shown in Fig 1E, albeit without support of a statistical analysis. Please do it.

**Response**: Statistical analysis was conducted on the data presented in Fig 1E. We have redone the GUS assay to improve the quality of signal.

• FigS1 : The authors miss to compare the performance of Xcc pthA4:Tn5 (dTALEWTLOB1) to the WT strain. Would be good to know the full level of complementation.

**Response**: The comparison between Xcc pthA4:Tn5 + dTALEWTLOB1 and the WT strain is presented in fig 3B and 3C. Complementation of dTALEWTLOB1 does not match 100 percent to the WT. While significantly contributing to the accumulation of the LOB1 transcript and growth in planta Xcc pthA4:Tn5 + dTALEWTLOB1 is not as pathogenic as the WT strain.

• FigS2 : why so many protein bands on the left membrane ? Why not on the membrane to the right ? It should at least be indicated which band is expected to correspond to the TAL analyzed.

**Response**: The top bands matched the predicted full length TALEs and the other bands appear to be degradation products. We modified the protein extraction and running protocols to avoid protein degradation and repeated the gel running. The new western blot (S4 fig) contains only the full-length bands.

• In the M&M section (L385-389) the authors refer to evaluating potential rearrangements when bacteria are grown on plates but I missed to see the results of this experiment. Is this reported or just mentioned here. If so, can one clarify what has been tested to show that no “.. repeat alterations in the dTALEs isolated from bacteria grown in artificial media” has been observed ?

**Response**: We added the NA media streaking of the bacteria to the result section (lines 219-225). In brief, the strains were routinely streaked on NA with antibiotic selection in parallel to the plant infection for the duration of experiment. Plasmids were extracted from three randomly chosen clones isolated from each of the strains. We did not observe any modification of the repeat arrays compared to the parental dTALEs.

Other minor remarks/typos :

L49 : not all TALEs are vital for bacterial pathogenicity, please correct.

**Response**: corrected

L59 : one ref is not edited correctly (Kumar Verma et al 2018).

**Response**: This reference was removed.

L61 : better refer to a review here.

**Response**: The reference was changed to two review articles: Muñoz Bodnar A, Bernal A, Szurek B, López CE. Tell Me a Tale of TALEs. Mol Biotechnol. 2013;53: 228–235. doi:10.1007/s12033-012-9619-3

Perez-Quintero AL, Szurek B. A Decade Decoded: Spies and Hackers in the History of TAL Effectors Research. Annu Rev Phytopathol. 2019 Aug 25;57:459-481. doi: 10.1146/annurev-phyto-082718-100026. Epub 2019 Aug 6. PMID: 31387457.

L67 : earlier pioneer article that would better fit here.

**Response**: we cited the following pioneer articles:

Yang B, Sugio A, White FF. Os8N3 is a host disease-susceptibility gene for bacterial blight of rice. Proc Natl Acad Sci U S A. 2006;103: 10503–10508. doi:10.1073/pnas.0604088103;

Antony G, Zhou J, Huang S, Li T, Liu B, White F, et al. Rice xa13 recessive resistance to bacterial blight is defeated by induction of the disease susceptibility gene Os-11N3. Plant Cell. 2010;22: 3864–76. doi:10.1105/tpc.110.078964

L78 : either cite one or two complete review articles or refer to all original articles but mixing randomly both is weird

**Response:** we changed all the citations to original manuscripts

L260 : point mutaions

**Response**: corrected to “point mutations”

L266 : suggested

**Response**: corrected to “suggested”
